# How does diagnostic subtype affect the quality of primary care for people with dementia? A retrospective cohort study in 1490 English general practices

**DOI:** 10.1093/ageing/afag101

**Published:** 2026-04-20

**Authors:** Charlotte Morris, Pearl L H Mok, Dame Louise Robinson, Darren M Ashcroft, Tom Blakeman, Evangelos Kontopantelis

**Affiliations:** Division of Population Health, Health Services Research and Primary Care School of Health Sciences, The University of Manchester, Manchester, M13 9PL, UK; NIHR Greater Manchester Patient Safety Research Collaboration, The University of Manchester, Manchester, M13 9PL, UK; NIHR School for Primary Care Research, The University of Manchester, Manchester, M13 9PL, UK; NIHR Greater Manchester Patient Safety Research Collaboration, The University of Manchester, Manchester, M13 9PL, UK; Division of Pharmacy and Optometry, School of Health Sciences, The University of Manchester, Manchester, England, M13 9PL, UK; Population Health Science Institute, Faculty of Medical Science, University of Newcastle upon Tyne, Newcastle upon Tyne, NE1 7RU, UK; Population Health Science Institute, Faculty of Medical Science, University of Newcastle upon Tyne, Newcastle upon Tyne, NE1 7RU, UK; NIHR Greater Manchester Patient Safety Research Collaboration, The University of Manchester, Manchester, England, M13 9PL, UK; NIHR School for Primary Care Research, The University of Manchester, Manchester, England, M13 9PL, UK; Division of Population Health, Health Services Research and Primary Care School of Health Sciences, The University of Manchester, Manchester, M13 9PL, UK; NIHR Greater Manchester Patient Safety Research Collaboration, The University of Manchester, Manchester, M13 9PL, UK; NIHR School for Primary Care Research, The University of Manchester, Manchester, M13 9PL, UK; Division of informatics, Imaging and Data Sciences, The University of Manchester, Manchester, UK; Division of Family Medicine, Yong Loo Lin School of Medicine, National University of Singapore, Singapore 119228, Singapore

**Keywords:** dementia, dementia subtypes, health inequity, prescribing, primary care, older people

## Abstract

**Introduction:**

Diagnostic subtype has been suggested as a determinant of inequity for people with dementia; its impact on primary care provision is underexplored. This study investigated the association between dementia subtype and likelihood of receiving guideline-consistent primary care.

**Method:**

Retrospective cohort study using Clinical Practice Research Datalink (Aurum) database, 1.1.2006-30.06.2024. We examined potential inequity with eight dementia subtypes: Alzheimer’s disease (AD), Lewy body dementia (LBD), vascular, frontotemporal, unspecified, other and two mixed categories. Six outcomes were examined: care plan or medication review (both within 24 months of index) and four indicators of potentially inappropriate prescribing (PIP) (high anti-cholinergic burden drugs, z-drugs, benzodiazepines and anti-psychotics). Cox-regression models were used, adjusting for: age, sex, comorbidities, deprivation and ethnicity.

**Results:**

A total of 571 663 people were included and 72.1% received a care plan; 79.4% received a medication review within 24 months. Compared to AD: people with mixed dementias were more likely to receive a care plan [hazard ratio (HR) 1.29, 95% confidence interval (CI) 1.26–1.32 for mixed including AD/LBD, HR 1.37, 1.32–1.43 for mixed non-AD/LBD]. All other subtypes were less likely to receive a care plan. Individuals with mixed AD/LBD (HR 1.28, 1.26–1.32), mixed non-AD/LBD (HR 1.35, 1.26–1.45), vascular (HR 1.05, CI 1.04–1.07), LBD (HR 1.02, 1.01–1.04) and unspecified (HR 1.02, 1.01–1.03) were more likely to receive medication reviews. Compared to AD, all other subtypes were more likely to experience PIP across all four indicators.

**Conclusion:**

We found greater likelihood of PIP in people with non-AD dementias, a novel finding. Further research is needed, especially with new AD drugs potentially widening disparities.

## Key Points

- People with mixed dementia were more likely to have a care plan than those with Alzheimer’s disease.
- People with mixed, vascular and unspecified dementias were more likely to receive medication reviews.
- Patients with non-Alzheimer’s disease subtypes were more likely to experience potentially inappropriate prescribing.
- Diagnostic subtype is a newly identified driver of inequity in primary care for dementia.
- This research directly quantifies how dementia subtype influences provision of guideline-consistent primary care.

## Introduction

The number of people with dementia of all types is set to increase drastically [1]. The World Health Organisation (WHO) recognises this, identifying dementia healthcare as a priority [1, 2]. Guidance suggests post-diagnostic dementia care should be provided in primary care, where most healthcare for people with dementia is already being delivered [2, 3].

Dementia is an umbrella term, encompassing different pathologies, resulting in a clinical syndrome of impaired cognitive functioning severe enough to interfere with daily life [4, 5]. The main dementia subtypes are Alzheimer’s disease (AD) (60%), vascular dementia (VaD) (15%), Lewy body dementia (LBD) (5%–10%), frontotemporal dementia (FTD) (2%–5%) and other causes (3%) [5–7]. Some people never receive a formal subtype diagnosis [8, 9], meaning people live with ‘unspecified’ dementia. This represents potentially suboptimal healthcare, as there is great variation in clinical picture, treatments and prognosis [5]. Diagnostic subtype is often a clinical best-guess rather than definitive biological fact [10–12]. An unspecified diagnosis is sometimes the most medically appropriate choice; forcing a specific label can be futile [11, 12] failing to reflect the complex nature of underlying pathologies [10]. Despite this, considering the most likely subtype, particularly to understand eligibility for anti-dementia medications [4, 13] and likely trajectories [14] has clinical value. Primary care guidelines are not subtype-specific [4].

In the UK, primary care guidance for dementia, first published in 2006 (updated 2018) [4], recommends annual care planning, medication review (including deprescribing) and avoiding anti-psychotics, anticholinergic drugs, z-drugs and benzodiazepines [4]. These examples of potentially inappropriate prescribing (PIP) are highlighted in Beers prescribing criteria, with specific guidance for dementia since 2012 [15]. Broad guidance is that these medications should be avoided wherever possible, with strong evidence of harm from their continued prescription [16]. However, in some clinical situations, these medications are required; the term ‘potentially inappropriate’ is used to capture this nuance. Quality of primary care for people with dementia is multidimensional [4, 17]. In this study, we focus on three important aspects of high-quality primary care for people with dementia: care planning, medication review and avoidance of PIP.

Interest in inequity for people with dementia is growing, with evidence of disparities based on patient and system-level characteristics [18, 19] and commissioning [20]. Studies have shown inequity in receipt of guideline-consistent primary care based on age, sex, socioeconomic status and ethnicity [19, 21–23]. While diagnostic subtype is recognised as a potential factor in disparities in primary care [18], no studies have yet explored this. This study examined a large electronic health record (EHR) database in England to examine six indicators covering care planning, medication review and PIP in relation to dementia subtype.

## Method

### Study design, data source and linkage

This was a retrospective cohort study using the Clinical Practice Research Datalink (CPRD) Aurum. CPRD Aurum is comprised of coded EHRs from English primary care practices, covering around one-fifth of the population. Data are broadly representative of the population [24].

A comprehensive list of dementia codes was developed [25, 26] and compared to that from a relevant paper [16]. Codelists are provided in Appendix 2. Study start date was 1 January 2006, ending on 30 June 2024. Patients with a dementia code entered on or after the study start date, up to one day before the study end-date, and aged 18 years or above, were eligible. Follow up began at index-date, defined as the date that the patient first had a dementia diagnosis, after registering with a CPRD practice. We did not impose a minimum practice registration period.

Those first diagnosed after study start date but before their CPRD registration date were included (even if they did not have a subsequent diagnostic code), with their index-date being taken as the date of CPRD registration. Censor-date was defined as the earliest of date of death, registration end-date, last practice collection date or study end-date. Those with censor dates before their first diagnostic code were excluded.

We obtained patient-level linked data for Index of Multiple Deprivation (IMD) (2019) and ethnicity. IMD is a small area-level (average 1600 people) deprivation score, calculated as an aggregate across numerous indices within seven domains: income, employment, education, health, crime, housing and environment [27]. The cohort was linked to patient locality IMD with data provided at patient-postcode level [28] with data provided in quintiles. Only patients with available linked IMD data were eligible for inclusion. The CPRD-algorithm derived ethnicity record was used [29], classes were Asian, Black, Mixed, White, Other or Unknown.

The study protocol was approved by the CPRD scientific committee, study reference 23_002686 [30].

### Selection of indicators/outcomes

We selected six indicators of guideline-consistent care. These indicators reflect two different aspects of quality: receiving a recommended review and avoiding potentially harmful prescribing.

- **Guideline-consistent if present:**
  - A dementia-specific care plan or review coded within 24 months of index-date.
  - A medication review coded within 24 months of index-date.
- **Guideline-consistent if absent:**
  - Prescribing a high anticholinergic burden drug ≥3 times after index-date.
  - Prescribing a z-drug ≥3 times after index-date.
  - Prescribing a benzodiazepine ≥3 times after index-date.
  - Prescribing an antipsychotic ≥3 times after index-date.
  - Receipt of any of the 4 PIP medications ≥3 times after index-date [15].

Guidance recommends everyone with dementia has an annual care plan and medication review [4, 31]. We chose receiving a care plan or a medication review within 24 months as the outcome measure, as this is consistent with guidance for reviews at least annually [4]. We chose ≥3 prescriptions as a pragmatic proxy for PIP, rather than this being a defined guideline standard. High anticholinergic burden (ACB) drugs (scoring ≥3 on the ACB scale) [32], z-drugs and benzodiazepines should be avoided due to associations with adverse health outcomes [15, 32–36]. Antipsychotics, which carry significant risks of morbidity and mortality, should only be used in exceptional circumstances [4, 16, 37].

Each indicator was analysed as a binary outcome (e.g. received a care plan or review within 24 months of the index-date = 1, did not = 0; received 3 or more issues of a z-drug = 1, did not = 0). We defined PIP as three or more issues of each PIP indicator to increase robustness. This threshold was chosen because it indicates a sustained PIP and a departure from guidance. A single prescription, or even a second, might be clinically justifiable for short-term use in specific situations.

### Dementia subtypes

We used subtype at index-date to classify patients. If no code was entered at index-date (i.e. those who entered the study at CPRD registration date, with a diagnostic code entered before this), their first diagnostic code was used to classify baseline subtype. In the UK, diagnosis of dementia is usually made by a specialist [4]. Therefore, the earliest code is likely to be the diagnostic code entered from the specialist letter, arguably more accurate than later codes entered when the focus of encounters is not diagnosis.

To identify subtype, the dementia codelist was split into subtype groups. These were AD, LBD, VAD, FTD, Unspecified (no subtype specified) and other dementias (e.g. Huntington’s dementia). Separate datasets were created for each subtype. For patients with more than one subtype coded on the same date at either the index-date or date of first diagnosis, we classified them as having mixed dementia. We subdivided this category into two groups. The first, mixed (including AD or LBD), contained cases where one of the recorded subtypes was either AD or LBD. The second, mixed (non-AD/LBD), included all other mixed dementia cases. This distinction was important because anti-dementia medications are indicated for AD and LBD but no other subtypes. This categorisation allowed more detailed analyses of anti-dementia medication prescribing to be published separately.

### Comorbidities

**Figure 1:**
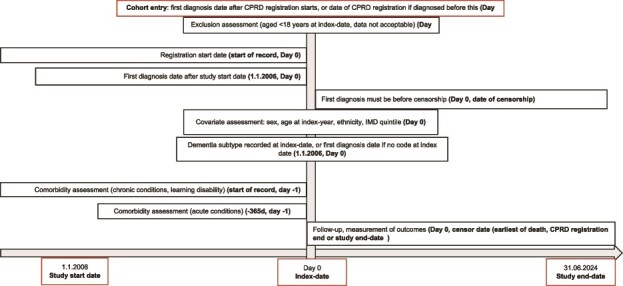
Description of the cohort study design. (Abbreviations: CPRD, Clinical Practice Research Datalink; IMD: Index of multiple deprivation; d, days).

Figure 1 shows when/how covariates, outcomes and follow-ups were examined and handled in the analyses [38]. An adapted version of the Cambridge Multimorbidity Score was used to account for comorbidities [38]. A detailed description of how comorbidities were handled in the cohort is presented in the supplementary materials (Appendix 1) [38].

### Statistical analysis

Analyses were conducted in Stata MP version 18. For all six outcomes, time-to-event analyses were used (Cox regression modelling) to explore the relationship between dementia subtype diagnosis and likelihood of outcome. Cox regression modelling allowed us to account for varying follow-up times from index-date until receiving the intervention, or censor-date whichever was first. Receiving a care plan, medication review or a third issue of PIP was classed as the ‘event.’ Within each analysis, people were followed up from index-date until censor date or until receiving the intervention. For analyses looking at outcomes within 24 months, follow-up was capped at 730 days. Minimum follow-up was 1 day. If a relevant code was not present, this was deemed to be absent, not missing.

Models were run for each of the six outcomes to examine how subtype diagnosis affected the likelihood of receiving guideline-consistent primary care. Models were adjusted for sex, age in index year, modified CMS score at index-date, LD, IMD quintiles and ethnicity. Standard errors were clustered by practice. Covariate selection was determined *a priori* based on a causal framework. Age [39], sex [39], ethnicity [40, 41], socioeconomic deprivation [42], multimorbidity and LD [43] are known to drive significant inequalities in timing and accuracy of dementia diagnosis. These factors are independently associated with variations in healthcare use [19, 22, 44, 45]. By adjusting for these, we aimed to minimise confounding from factors affecting dementia diagnosis and guideline-consistent primary care.

**Figure 2:**
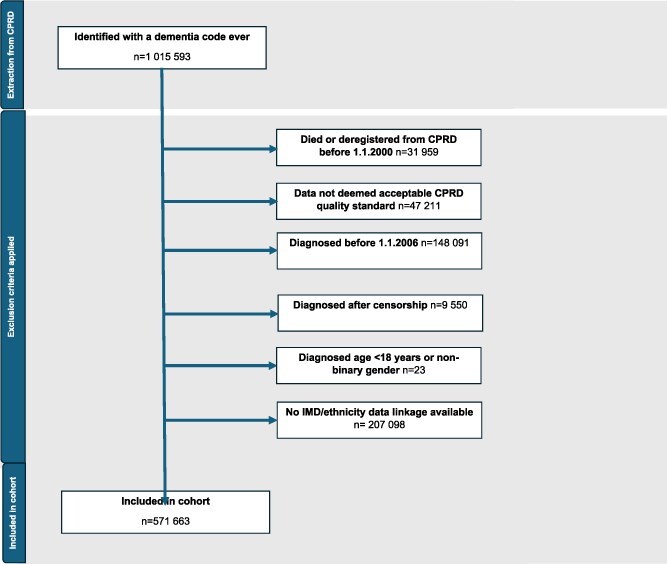
Study flow diagram.

There were few missing data, after applying inclusion and exclusion criteria, only ethnicity had any cases with missing data (*n* = 149) these were excluded (Figure 2). All other covariates and outcomes were complete. Complete case analysis was conducted.

Detailed sensitivity analyses are described in the supplementary files (Appendix 1).

#### Patient and public involvement

Our study design was directly informed by a public involvement group, whose insights shifted the analytical focus from end-of-life care to ‘living well’ outcomes. By prioritising functional status and quality-of-life indicators, the group ensured the research remained aligned with patient-centred values and the lived experience of dementia. Group composition varied at meetings. Overall, it comprised of people living with dementia, current and former carers, former healthcare workers and people with no direct link to dementia but an interest in research.

## Results

**Figure 3:**
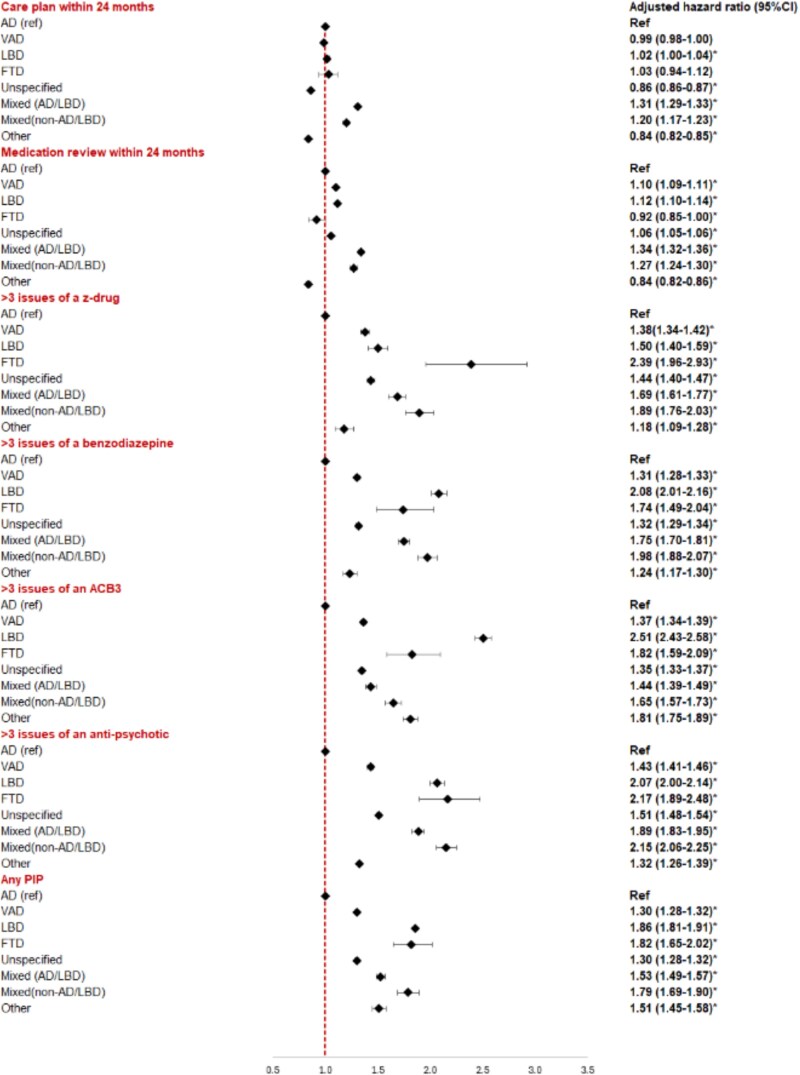
Hazard ratios with 95% confidence intervals of outcomes for each subtype of dementia Adjusted for age, gender, index of multiple deprivation quintile, ethnicity, comorbidity score, learning disability diagnosis, standard errors clustered by practice.

A total of 571 663 people were included; Figure 2 describes application of inclusion and exclusion criteria. Table 1 shows baseline characteristics for the whole study population and by subtype. Table 2 shows the number of people receiving care plans, medication reviews and indicators of PIP overall and by subtype. Figure 3 shows results of adjusted analyses for each of the six outcome measures and the dementia subtype.

**Table 1:** Baseline characteristics for the study population by subtype

	Study population	AD	VAD	LBD	FTD	Unspecified	Mixed (AD/LBD)	Mixed (Non-AD/LBD)	Other
*Mean age at diagnosis [range]*	83 [18–112]	82.5 [19–107]	83.1 [27–108]	79.3 [30–102]	71.0 [26–95]	84.0 [18–112]	83.3 [38–106]	83.3 [21–106]	79.3 [18–104]
*18–50*	1461 (0.3%)	340 (27.0%)	110 (8.7%)	17 (1.3%)	31 (2.5%)	537 (42.4%)	20 (1.6%)	12 (1.0%)	394 (30.9%)
*51–65*	16 928 (%)	6102 (36.0%)	2832 (16.7%)	656 (3.9%)	250 (1.5%)	5276 (33.8%)	419 (2.5%)	159 (0.9%)	1234 (7.3%)
*65+*	553 274 (97.8%)	197 746 (96.8%)	123 385 (97.7%)	17 711 (96.4%)	688 (71.0%)	177 512 (96.8%)	18 996 (97.7%)	4404 (96.2%)	12 832 (88.7%)
*Female (n, %)*	356 179 (62.3%)	132 070 (64.7%)	73 059 (57.8%)	7628 (41.5%)	475 (49.0%)	120 254 (65.6%)	12 203 (62.8%)	2722 (59.5%)	7768 (53.7%)
*IMD Quintile* *(n, %)*									
1 (least deprived)	125 934 (22.0%)	47 130 (23.1%)	26 115 (20.7%)	4707 (25.6%)	221 (22.8%)	39 866 (21.8%)	4075 (21.0%)	927 (20.3%)	2891 (20.0%)
2	128 534 (22.5%)	47 143 (23.1%)	27 772 (22.0%)	4236 (23.1%)	209 (21.6%)	40.693 (22.2%)	4262 (21.9%)	1062 (23.2%)	3157 (21.8%)
3	114 055 (20.0%)	40 079 (19.6%)	24 943 (19.7%)	3727 (20.3%)	186 (19.2%)	37 449 (20.4%)	3776 (19.4%)	913 (20.0%)	2982 (20.6%)
4	104 426 (18.3%)	36 418 (17.8%)	23 577 (18.7%)	3007 (16.4%)	184 (19.0%)	34 065 (18.6%)	3651 (18.8%)	812 (17.8%)	2712 (18.8%)
5 (most deprived)	98 714 (17.3)	33 418 (16.4%)	23 920 (19.0%)	2707 (14.7%)	169 (17.4%)	31 252 (17.1%)	3671 (18.9%)	861 (18.8%)	2716 (18.8%)
*Ethnicity*									
Asian	12 819 (2.2%)	4222 (2.1%)	3168 (2.5%)	347 (1.9%)	44 (4.5%)	4172 (2.3%)	399 (2.1%)	88 (1.9%)	379 (2.6%)
Black	12 073 (2.1%)	3863 (1.9%)	3105 (2.5%)	333 (1.8%)	16 (1.7%)	3867 (2.1%)	418 (2.2%)	114 (2.5%)	357 (2.5%)
Mixed	1763 (0.3%)	603 (0.3%)	407 (0.3%)	42 (0.2%)	<10 (<1%)	569 (0.3%)	66 (0.3%)	<20 (<1%)	52 (0.4%)
White	538 097 (94.1%)	193 286 (94.7%)	118 411 (93.7%)	17 497 (95.2%)	890 (91.9%)	171 804 (93.7%)	18 372 (94.5%)	4308 (94.2%)	13 529 (93.6%)
Other	319 (0.1%)	128 (0.1%)	48 (0.04%)	10 (0.1%)	<10 (<1%)	116 (0.1%)	6 (0.03%)	<5 (<1%)	10 (0.1%)
Unknown	6592 (1.2%)	2086 (1.02%)	1188 (0.9%)	155 (0.8%)	13 (1.4%)	2797 (1.5%)	174 (0.9%)	46 (1.0%)	133 (0.9%)
*Mean comorbidity score [range]*	5.8 [1.8–37.0]	5.3 [1.81–33.5]	7.01 [1.81–37.0]	5.6 [1.81–31.6]	4.9 [1.8–23.8]	5.80 [1.81–32.3]	3.8 [1.81–28.7]	3.5 [1.81–30.7]	7.0 [1.81–33.8]
*Learning disability (n, %)*	2903 (0.5%)	1236 (0.6%)	252 (0.2%)	28 (0.15%)	<10 (<1%)	1269 (0.7%)	33 (0.2%)	<10 (<1%)	76 (0.5%)
*Median follow-up time (days), [mean, range]*	585 [835, 1–6753]	703 [935, 1–6753]	545 [804, 1–6740]	534 [737, 1–6039]	527 [782, 2–6472]	523 [787, 1–6751]	498 [714, 1–6656]	464 [680, 1–6376]	416 [663, 1–6689]
*Number who died within 2 years of index-date*	169 129 (29.6%)	47 596 (23.3%)	41,135 (32.6%)	6065 (33.0%)	152 (15.7%)	62 466 (34.1%)	7054 (36.3%)	1912 (41.8%)	2749 (19.0%)
*Total n*	**571 663**	**204 188**	**126 327**	**18 384**	**969**	**183 325**	**19 435**	**4575**	**14 460**

Those with *n* < 10 are masked to ensure the anonymity of CPRD data is not compromised, as per CPRD guidance. To ensure complete masking sometimes numbers are presented as <5, <10 or <20.

Abbreviations: n - number, IMD - index of multiple deprivation.

**Table 2:** Number of patients receiving each indicator of high-quality primary care, by dementia subtype

	*Total (n)*	*Care plan within 24 months*		*Medication review within 24 months*		*>3 issues of a z-drug*		*>3 issues of a benzodiazepine*		*>3 issues of an anti-psychotic*		*>3 issues of an ACB score 3 drug*		*>3 issues of any PIP*	
		*n*	*%*	*n*	*%*	*n*	*%*	*n*	*%*	*n*	*%*	*n*	*%*	*n*	*%*
*AD only*	**204 188**	157 294	77.0	166 008	81.3	12 951	6.3	28 018	13.7	27 409	13.4	32 097	15.7	67 842	33.2%
*VAD only*	**126 327**	89 876	71.2	99 765	79.0	9138	7.2	18 087	14.3	19 253	15.2	23 459	18.6	46 321	36.7%
*LBD only*	**18 384**	13 280	72.2	14 694	80.0	1230	6.7	3684	20.0	3738	20.3	5179	28.2	8130	44.2%
*FTD only*	**969**	688	71.0	690	71.2	103	10.6	168	17.3	227	23.4	214	22.1	397	41.0%
*Unspecified*	**183 325**	121 646	66.4	141 487	77.2	13 786	7.5	27 015	14.7	30 095	16.4	32 099	17.5	66 653	36.4%
*Mixed (AD/LBD)*	**19 435**	16 327	84.0	17 169	88.3	1405	7.2	3400	17.5	3601	18.5	3082	15.9	7311	37.6%
*Mixed* *(non-AD/LBD)*	**4575**	3963	86.6	4152	90.8	363	7.9	892	19.5	964	21.1	795	17.4	1895	41.4%
*Other only*	**14 460**	9034	62.5	9769	67.6	710	4.9	1606	11.1	1780	12.3	2910	20.1	4524	31.3%
*All*	***571 663***	412 108	72.1	453 734	79.4	39 686	7.0	82 870	14.5	87 067	15.2	99 835	17.5	203 073	35.5%

Abbreviations: AD - Alzheimer's disease, VAD - Vascular Dementia, LBD - Lewy Body Dementia, FTD - Frontotemporal Dementia.

### Care planning

In total, 412 108 (72.1%) people received a dementia-specific care plan or review within 24 months of index-date. The biggest differences in the likelihood of receiving a care plan were seen for people with mixed (non-AD/LBD) dementia (HR 1.37, 1.32–1.43) and mixed (AD/LBD) dementia HR 1.29, 95% CI 1.26–1.32), compared to those with AD alone. All other subtypes were significantly less likely to get a care plan.

### Medication review

A total of 453 734 (79.4%) people received a medication review within 24 months of index-date. Those with mixed dementias were significantly more likely to receive a medication review, compared to those with AD; mixed (AD/LBD) (HR 1.28, 1.26–1.32); mixed (non-AD/LBD) (HR 1.35, 1.26–1.45). Those with VaD (HR 1.05, 1.04–1.07) and LBD (HR 1.02, 1.001–1.04) were also significantly more likely to receive a medication review within 24 months. Those with FTD (0.83, 0.77–0.89) and ‘other’ (HR 0.80, CI 0.78–0.82) dementias were significantly less likely to receive a medication review than those with AD.

### Potentially inappropriate prescribing

#### More than three issues of a z-drug

A total of 39 686 (7%) people received 3 or more issues of a z-drug. Receiving more than 3 issues of a z-drug was strongly associated with dementia subtype. Those with all other subtypes were significantly more likely to receive 3 or more issues of a z-drug compared to those with AD; those with FTD were most likely (HR 2.56, 2.09–3.13).

#### More than three issues of a high ACB drug

A total of 99 835 (17.5%) people had received high anticholinergic burden drug 3 or more times during the study period. Dementia subtype was associated strongly with the likelihood of receiving a high ACB drug at least 3 times. All subtypes were more likely to receive high ACB drug compared to those with AD, with the biggest differences seen for LBD (HR 2.48, 2.40–2.56) and other dementias (HR 1.95, 1.84–2.06).

#### More than three issues of a benzodiazepine

A total of 82 870 (14.5%) received at least 3 issues of a benzodiazepine after the index-date. Non-AD subtype was strongly associated with an increased likelihood of this outcome. The largest differences were seen with LBD (HR 2.07, 1.99–2.16) and mixed dementia (non-AD/LBD) (HR 2.02, 1.83–2.22).

#### More than three issues of an anti-psychotic medication

A total of 87 067 (15.2%) people received 3 or more issues of an anti-psychotic medication after index-date. Those with non-AD subtypes were significantly more likely to receive antipsychotics, with FTD (HR 2.26, 1.96–2.58), mixed dementias (non-AD/LBD) (2.15, 1.99–2.31) and LBD (HR 2.04, 1.97–2.13) being most affected.

#### Any PIP

A total of 203 073 (35.5%) people received any PIP after the index date. Those with non-AD subtypes were all significantly more likely to receive any PIP. Those with LBD (1.86, 1.81–1.91), FTD (1.83, 1.65–2.02) and mixed (non-AD/LBD) (1.79, 1.69–1.90) showed the greatest likelihood of receiving PIP.

Detailed sensitivity analyses are presented in the supplementary files (Appendix 1). Overall, these supported the primary Cox modelling.

## Discussion

### Summary of results

The likelihood of receiving a care plan, medication review and PIP varied by dementia subtype.

- **Care plans:** 72.1% received a care plan within 2 years of index-date. Patients with VaD, LBD, FTD, unspecified and other dementias were less likely to receive a care plan than those with AD. Those with both types of mixed dementias were more likely to.
- **Medication reviews:** 79.4% patients received a medication review within 2 years of index-date. Individuals with VaD, LBD, both types of mixed dementias and unspecified dementias were more likely to receive a medication review than those with AD; those with other dementias or FTD were less likely.
- **PIP medications:** 35.5% patients received any PIP; 7% received ≥3 prescriptions of z-drugs; 14.5% received ≥3 prescriptions of benzodiazepines; 15.2% received ≥3 prescriptions for anti-psychotics and 17.5% received ≥3 prescriptions for high-ACB drugs. All non-AD subtypes were significantly more likely to receive PIP medications across all four indicators.

### Results in the context of current literature

Qualitative studies suggest individuals with rarer dementias often perceive suboptimal healthcare or lack of knowledge from professionals [46], our findings support this. It is unclear why people with non-AD dementias were more likely to experience PIP. This might be due to specific symptoms, or because specialists initiate these medications for people with rarer dementias and they are continued in primary care. Regardless, current guidance advises against these mediations for people with any dementia subtype; this should be followed, unless new evidence-based, subtype-specific guidance emerges.

Care planning is financially incentivised in UK primary care [47]. To achieve this indicator, primary care clinicians must record a care plan for 70% of their patients with dementia. It is possible some of the variation seen is because those with AD or mixed dementias are more likely to have their care plans undertaken in primary care, whereas those with rarer dementias only may have these completed in a secondary care setting. Evidence suggests that high rates of annual reviews are completed, but that quality varies [48], and patients are not always aware these have taken place [48–50].

Patients with VaD or LBD often have comorbidities monitored in primary care. This may explain why they receive medication reviews more often, perhaps opportunistically. The results present a paradox: non-AD subtypes were generally more likely to receive medication reviews, yet they were more likely to experience PIP. This could indicate suboptimal review [48] or be confounded by more detailed subtype diagnoses correlating with better guideline adherence at the practice level. An alternative explanation is patients with non-AD subtypes experience more severe neurobehavioural disturbances. This results in more frequent reviews, but as clinicians have few evidence-based alternatives, this results in PIP.

### Strengths and limitations

This study addresses an important gap. We employed robust methodology, analysing a large cohort, adjusting for multiple covariates. Our PIP definition was robust based on three medication issues and four indicators. Sensitivity analyses showed similar patterns over different timeframes and analytical models.

One limitation is we used baseline dementia subtype codes, which might not capture later, more specific diagnoses, though these initial codes are arguably accurate as they likely originated from specialist letters or hospital records. We created mixed dementia categories for individuals with more than one diagnosis code on their first diagnosis. This avoided overlooking diagnostic complexity, such as VAD and AD diagnosed at baseline. The mixed categories made up only 4.2% of the cohort, suggesting a minimal impact on overall findings. We included all people with coded dementia aged ≥18 years, but did not consider early-onset dementias separately due to small numbers. We did not use a minimum registration period due to the short follow-up of many patients, as this would have meant meaningful data (e.g. care plans coded soon after diagnosis) would have been lost. Generalisability is limited to people with coded dementia.

We defined PIP as ≥3 prescribing events for each drug following the index-date; this pragmatic proxy does not fully capture the clinical nuance of guidelines, which emphasise lowest effective dose and frequent review [4]. This definition may result in false positives (e.g. clinically justified short-term courses) or false negatives (e.g. long-duration single issues). If these prescribing patterns vary by subtype, HRs could be biased in unpredictable directions. Given these constraints, our findings should be interpreted as a measure of repeated prescribing rather than a definitive assessment of guideline non-compliance. HRs may be influenced by temporal overlap if latency between symptom onset and formal coding differs across subtypes. However, we only included prescribing events occurring after the index-date. Since a new diagnosis should trigger medication review, any continuation resulting in ≥3 prescriptions remains a pragmatic PIP proxy.

While we developed detailed comorbidity codes, our methodology may have underestimated comorbidities in these complex older adults. The small number of FTD cases, reflecting its low prevalence, resulted in less precise estimates for this subgroup [51]. A large number (*n* = 207 098) were excluded due to missing IMD/ethnicity data, which may influence generalisability of findings. Missing cases were predominantly from practices not contributing this data for any of their patients. The number with missing IMD/ethnicity data from practices which did contribute IMD/ethnicity data was far smaller (*n* = 149, 0.03%). Systematic evaluations demonstrate the linked CPRD population remains representative of the English population [51]. Consequently, exclusion of these patients is unlikely to have introduced significant selection bias.

### Implications

Further work examining inequity in healthcare provided for people with dementia with diagnostic subtype is needed; this study suggests significant inequity in basic primary healthcare. Considering the newer expensive, but promising treatments for AD [52, 53], inequity is likely to worsen. Qualitative work with primary care clinicians is needed to understand knowledge about managing non-AD subtypes.

Clinicians should be aware of these findings and consider if they are prescribing suboptimally for people with non-AD dementias. This may be a learning need, influenced by symptomatology, or due to specialist direction. Nevertheless, these prescriptions should be regularly reviewed to ensure ongoing safety and suitability. PIP may be a patient-centred option of last resort. Our findings could signal inadequate resources or therapeutic alternatives to PIP for those with more complex dementia presentations.

## Conclusion

This study is the first to show how primary care for people with dementia varies by diagnostic subtype. High rates of care planning and medication reviews were seen overall; those with mixed dementias were most likely to receive these. We found a consistent pattern of higher rates of PIP in people with non-AD dementias. The results signal inequity in primary care provision with different dementia subtypes, requiring urgent further work, especially as new AD drugs may worsen existing disparities.

## Supplementary Material

Supplementary_material_afag101

## Research Data Transparency and Availability

Not applicable for the CPRD data. Codelists shared in the appendix.
