# Supplementary material for: How does diagnostic subtype affect the quality of primary care for people with dementia? A retrospective cohort study in 1490 English general practices
Source: Age Ageing. 2026 Apr 20;55(4):afag101. doi: 10.1093/ageing/afag101 (PMC13092812; doi:10.1093/ageing/afag101)
Supplement: Supplementary_material_afag101 [file supplementary_material_afag101.zip › aa-25-3645-File002.pdf]

How does diagnostic subtype affect the quality of primary care for people with dementia? A retrospective cohort study in 1490 English General Practices

Supplementary materials:

Handling of comorbidities 2

Sensitivity analyses: Method 3

Sensitivity analyses: Results 3

Figure S1: Adjusted hazard ratios for care planning over time 2006-2011 (n=133,155), 2012-2017 (n=220,971), 2018-2024 (n=217,537) 5

Figure S2: Adjusted hazard ratios for medication reviews over time 2006-2011 (n=133,155), 2012-2017 (n=220,971), 2018-2024 (n=217,537) 6

Figure S3: Adjusted hazard ratios for PIP over time 2006-2011 (n=133,155), 2012-2017 (n=220,971), 2018-2024 (n=217,537) 7

Figure S4: Adjusted hazard ratios for competing risks* models for care plan and medication review indicators (n=571,663) 11

Figure S5: Adjusted hazard ratios for competing risks* models for PIP indicators (n=57,166, clustered for n=1481 practices) 12

Figure S6: Adjusted hazard ratios for incident dementia cases only (n=526,107) 13

Figure S7: Parametric regression models (Weibull distribution) n=571663† 14

Table S1: Supplementary Table 1: Accelerated failure time model with lognormal distribution, coefficient and time ratios for each outcome (n=571663) 15

Handling of comorbidities

Comorbidities were classed as present if they were recorded at least 1 day before the index-date. The list of relevant comorbidities was obtained from the Cambridge Multimorbidity Score(CMS)[1] ; detailed codelists were developed based on this. In total, 19 comorbidities were examined (supplementary file, box 1), as one of the 20 comorbidities in the CMS is dementia. We classed 3 conditions as acute and 16 as chronic. Acute conditions were constipation, depression and anxiety, and people were coded as having these if they had a relevant code in the year before index-date (i.e. from day -1 to day -365). Anxiety and depression were classed as acute to avoid lifetime prevalence inflation that can occur with acute conditions within CPRD data. Patients were coded as having a chronic condition if they had any of these codes entered prior to one day before the index-date. The CMS applies different weightings to each comorbidity, with weights dependent on the outcome examined[1]. As we were exploring indicators of guideline-consistent primary care, the primary care consultation outcome score was used. Learning disability (LD) is an important comorbidity for people with dementia not included in the CMS score. This was a binary indicator determined in the same way as chronic conditions.

**Supplementary Box 1: Comorbidities included in our comorbidity score (adapted from the Cambridge Multimorbidity Score)**
Dementia
Anxiety/Depression*
Painful condition
Hearing Loss
Irritable Bowel Syndrome
Asthma
Diabetes Mellitus
Coronary Heart Disease
Chronic Kidney Disease
Atrial Fibrillation
Constipation*
Stroke and TIA
COPD
Connective Tissue Disorder
Cancer
Alcohol problems
Heart Failure
Psychosis/Bipolar Disorder
Epilepsy
*Treated as acute

Sensitivity analyses: Method

Detailed sensitivity analyses were conducted examining how associations changed by year of diagnosis. Competing risk (CR) regression models were run for care planning and medication review outcomes, with death as the CR. Visual inspection of proportional hazard (PH) graphs confirmed the PH assumption was violated, as is common in large complex datasets[2, 3]. To account for this, sensitivity analyses were completed using a parametric model (Weibull distribution)[4] and an accelerated failure time model. Competing risk models for PIP indicators were not computationally possible for the full dataset, so were run on a random 10% sample. A further analysis including only incident cases was conducted.

Sensitivity analyses: Results

Overall, patterns for care plans, medication reviews, and PIP indicators were consistent over time (figures S1-S3). The CR models for care planning agreed with the main findings, the model for medication reviews showed a change in relationship direction. In contrast to the main

model, those with VaD (HR 0·94, 0·92-0·95), LBD (HR 0·94, 0·92-0·96), or unspecified dementias (HR 0·92, 0·91-0·93) became less likely to receive a medication review compared to those with AD (figure S4). CR models for PIP indicators were run on a 10% sub-cohort for computational reasons (figure S5). These models showed consistent directions but attenuated relationships, especially for smaller subgroups. Analyses for z-drugs were non-significant, likely due to small sample size reducing statistical power.

As the index-date was defined as either the first date of dementia diagnosis or the date of CPRD registration if diagnosis was made before CPRD registration (n=45,556), a sensitivity analysis was run including just the cases where index-date was the date of first diagnosis (n=526,107). Results were broadly similar to the primary models with no changes in the direction of associations seen (Figure S6).

The parametric models (supplementary figure S7) yielded similar results to the primary cox-models supporting their robustness. The cox-modelling was also supported by accelerated failure time (AFT) (lognormal) modelling. Higher time-rate ratios were seen for outcomes with lower HRs in the cox models, and vice versa, with only slight disagreement for medication reviews (supplementary table 1).

References:

1. Payne RA, Mendonca SC, Elliott MN, Saunders CL, Edwards DA, Marshall M, et al. Development and validation of the Cambridge Multimorbidity Score. *CMAJ*. 2020;192(5):E107–E114.

2. Bellera CA, MacGrogan G, Debled M, de Lara CT, Brouste V, Mathoulin-Pélissier S. Variables with time-varying effects and the Cox model: some statistical concepts illustrated with a prognostic factor study in breast cancer. *BMC Med Res Methodol*. 2010;10:20.

3. Sjölander A, Dickman PW. Why test for proportional hazards—or any other model assumptions? *American Journal of Epidemiology*. 2024;193(6):926–927.

4. Carroll KJ. On the use and utility of the Weibull model in the analysis of survival data. *Control Clin Trials*. 2003;24(6):682–701.

Figure S1: Adjusted hazard ratios for care planning over time 2006-2011 (n=133,155), 2012-2017 (n=220,971), 2018-2024 (n=217,537)

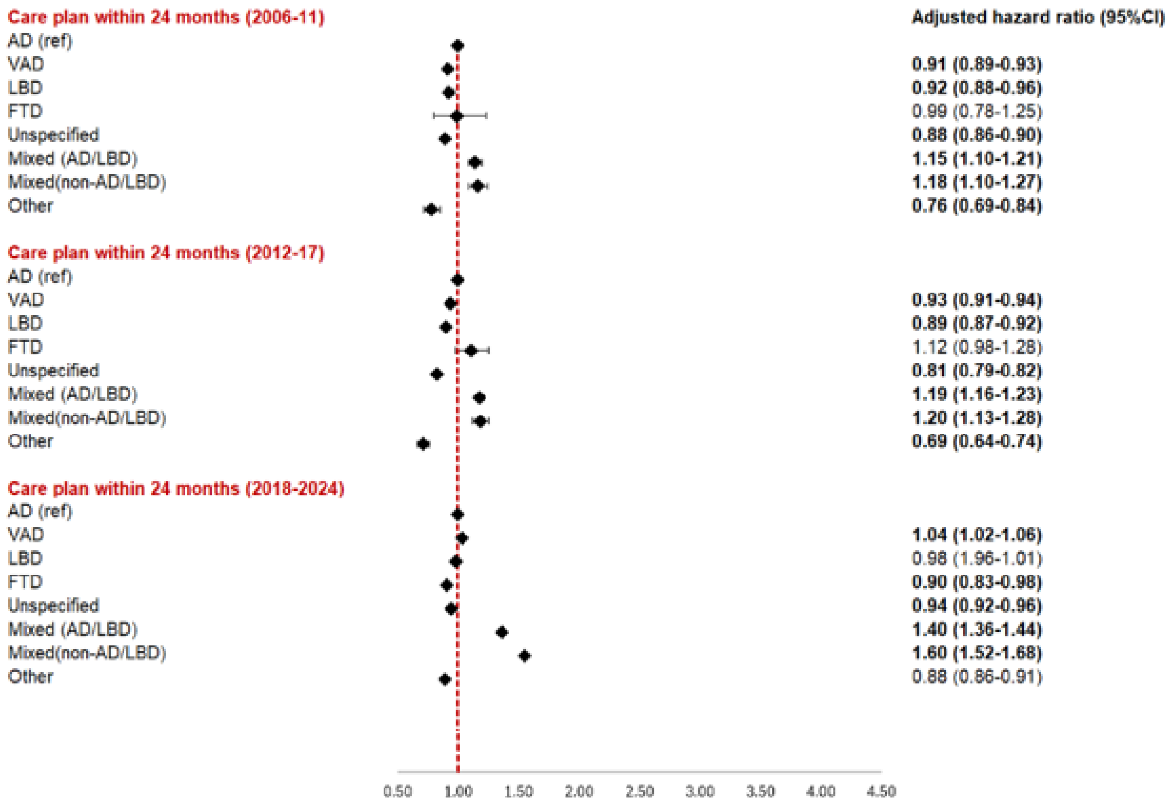

Figure S2: Adjusted hazard ratios for medication reviews over time 2006-2011 (n=133,155), 2012-2017 (n=220,971), 2018-2024 (n=217,537)

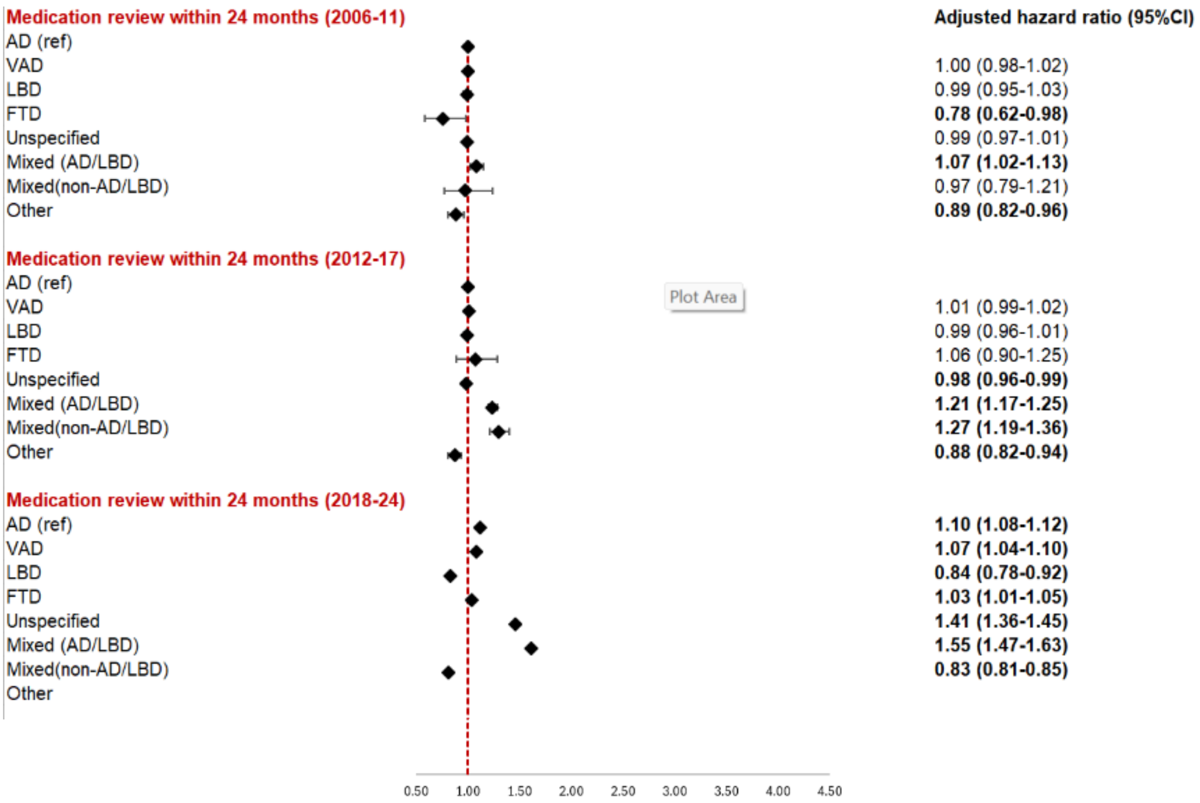

Figure S3: Adjusted hazard ratios for PIP over time 2006-2011 (n=133,155), 2012-2017 (n=220,971), 2018-2024 (n=217,537)

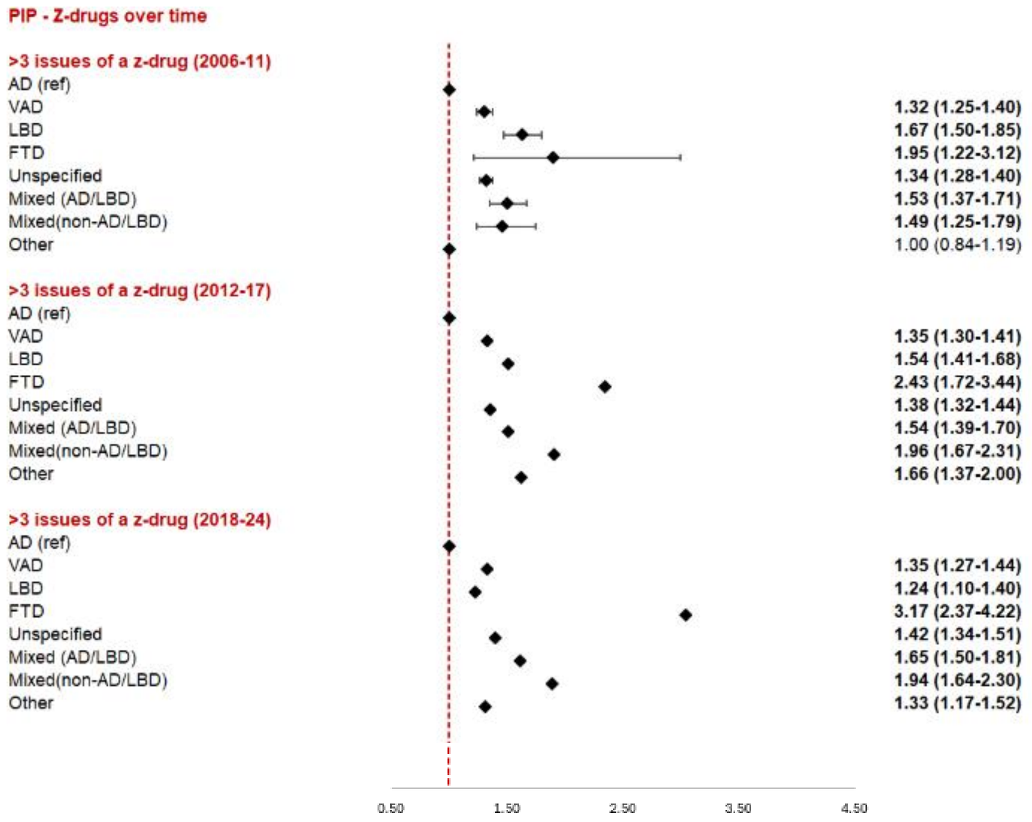

PIP - Benzodiazepines over time

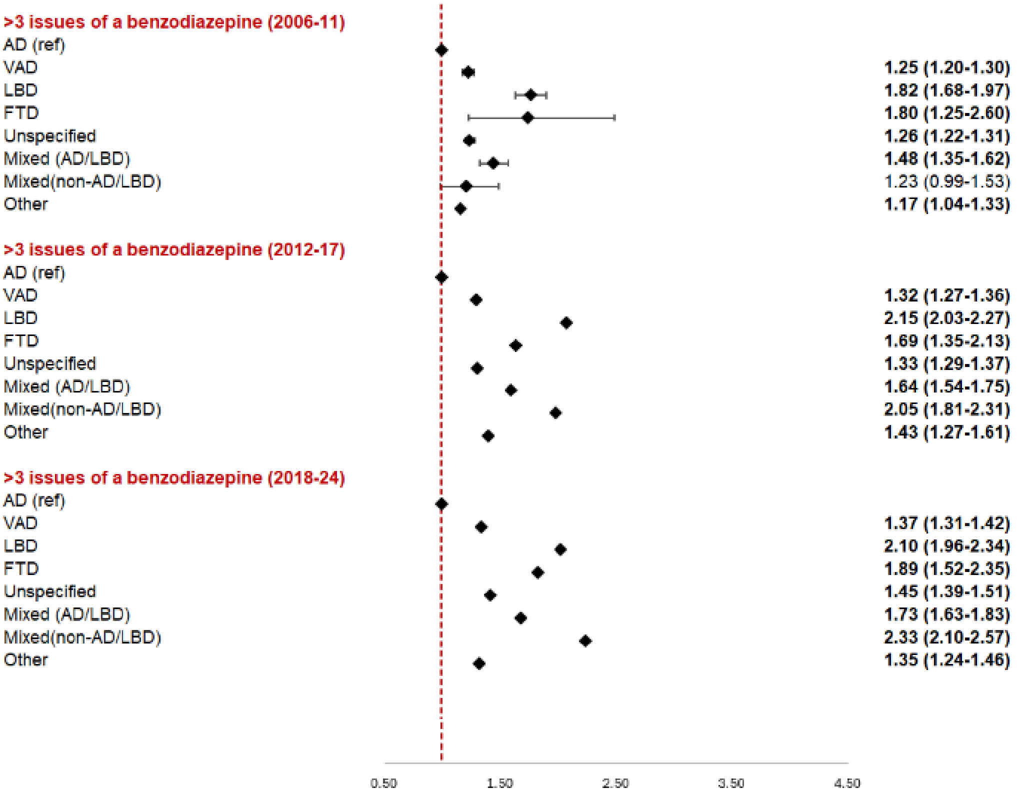

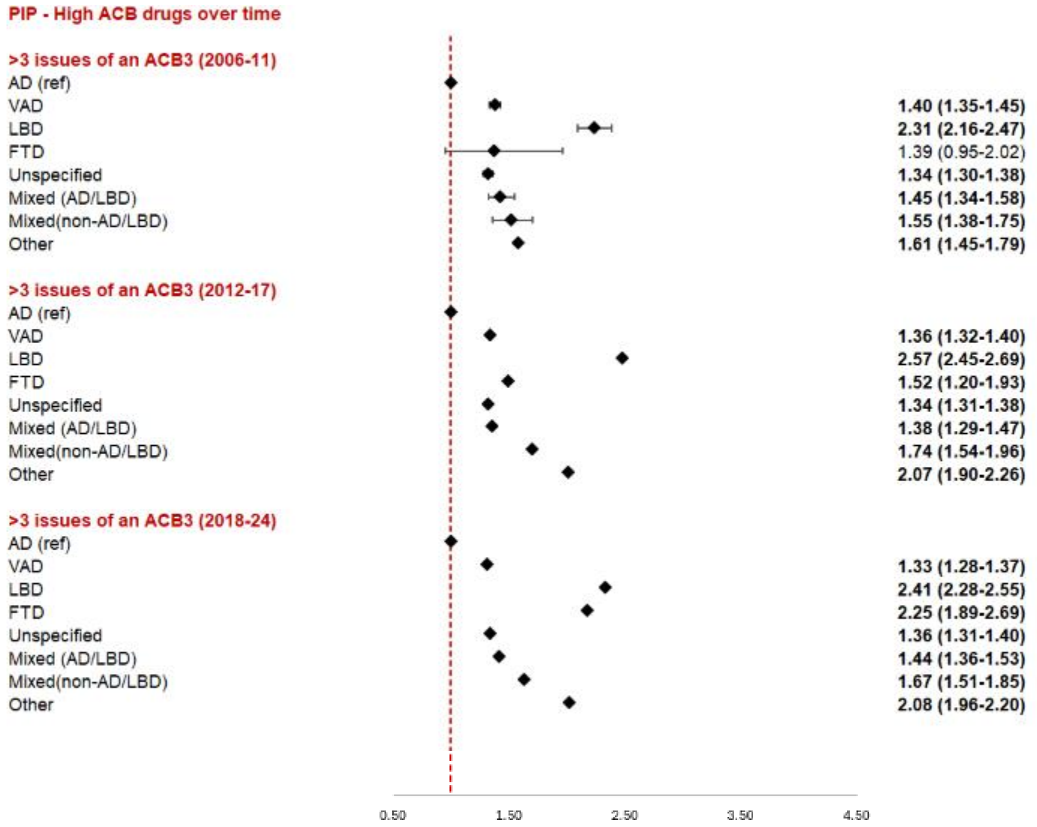
PIP - High ACB drugs over time
>3 issues of an ACB3 (2006-11)
AD (ref)
VAD
LBD
FTD
Unspecified
Mixed (AD/LBD)
Mixed(non-AD/LBD)
Other
1.40 (1.35-1.45)
2.31 (2.16-2.47)
1.39 (0.95-2.02)
1.34 (1.30-1.38)
1.45 (1.34-1.58)
1.55 (1.38-1.75)
1.61 (1.45-1.79)
>3 issues of an ACB3 (2012-17)
AD (ref)
VAD
LBD
FTD
Unspecified
Mixed (AD/LBD)
Mixed(non-AD/LBD)
Other
1.36 (1.32-1.40)
2.57 (2.45-2.69)
1.52 (1.20-1.93)
1.34 (1.31-1.38)
1.38 (1.29-1.47)
1.74 (1.54-1.96)
2.07 (1.90-2.26)
>3 issues of an ACB3 (2018-24)
AD (ref)
VAD
LBD
FTD
Unspecified
Mixed (AD/LBD)
Mixed(non-AD/LBD)
Other
1.33 (1.28-1.37)
2.41 (2.28-2.55)
2.25 (1.89-2.69)
1.36 (1.31-1.40)
1.44 (1.36-1.53)
1.67 (1.51-1.85)
2.08 (1.96-2.20)
0.50
1.50
2.50
3.50
4.50

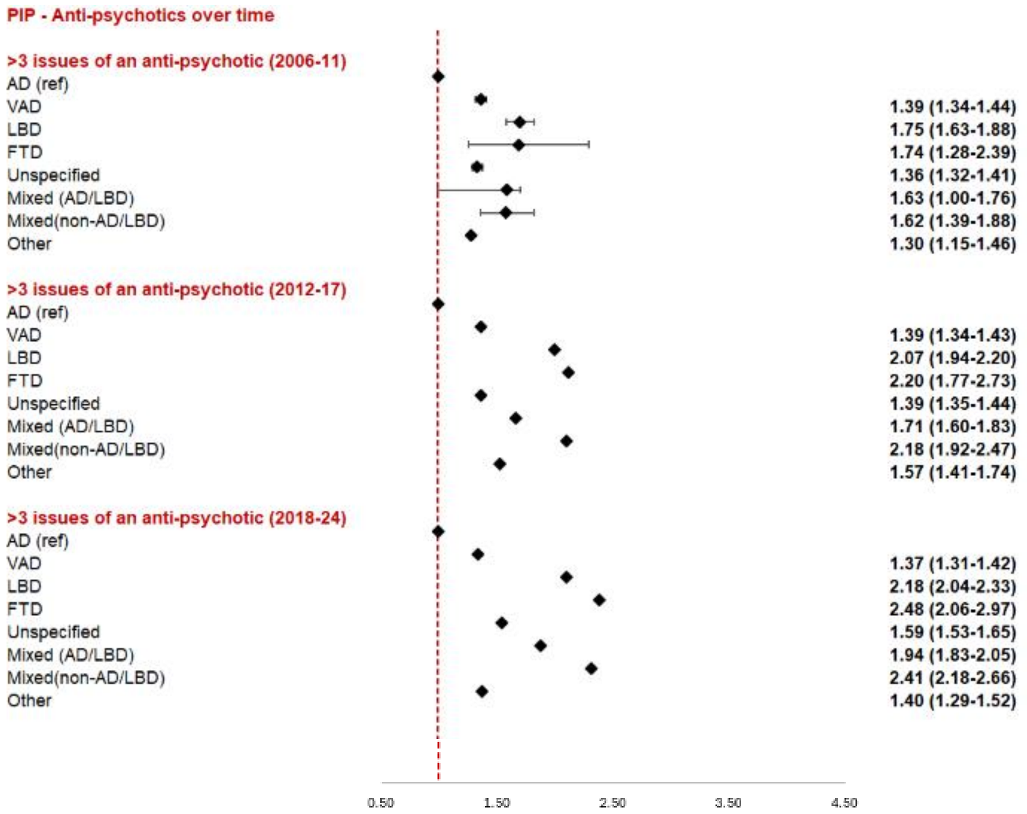
PIP - Anti-psychotics over time
>3 issues of an anti-psychotic (2006-11)
AD (ref)
VAD 1.39 (1.34-1.44)
LBD 1.75 (1.63-1.88)
FTD 1.74 (1.28-2.39)
Unspecified 1.36 (1.32-1.41)
Mixed (AD/LBD) 1.63 (1.00-1.76)
Mixed(non-AD/LBD) 1.62 (1.39-1.88)
Other 1.30 (1.15-1.46)
>3 issues of an anti-psychotic (2012-17)
AD (ref)
VAD 1.39 (1.34-1.43)
LBD 2.07 (1.94-2.20)
FTD 2.20 (1.77-2.73)
Unspecified 1.39 (1.35-1.44)
Mixed (AD/LBD) 1.71 (1.60-1.83)
Mixed(non-AD/LBD) 2.18 (1.92-2.47)
Other 1.57 (1.41-1.74)
>3 issues of an anti-psychotic (2018-24)
AD (ref)
VAD 1.37 (1.31-1.42)
LBD 2.18 (2.04-2.33)
FTD 2.48 (2.06-2.97)
Unspecified 1.59 (1.53-1.65)
Mixed (AD/LBD) 1.94 (1.83-2.05)
Mixed(non-AD/LBD) 2.41 (2.18-2.66)
Other 1.40 (1.29-1.52)
0.50
1.50
2.50
3.50
4.50

Figure S4: Adjusted hazard ratios for competing risks* models for care plan and medication review indicators (n=571,663)

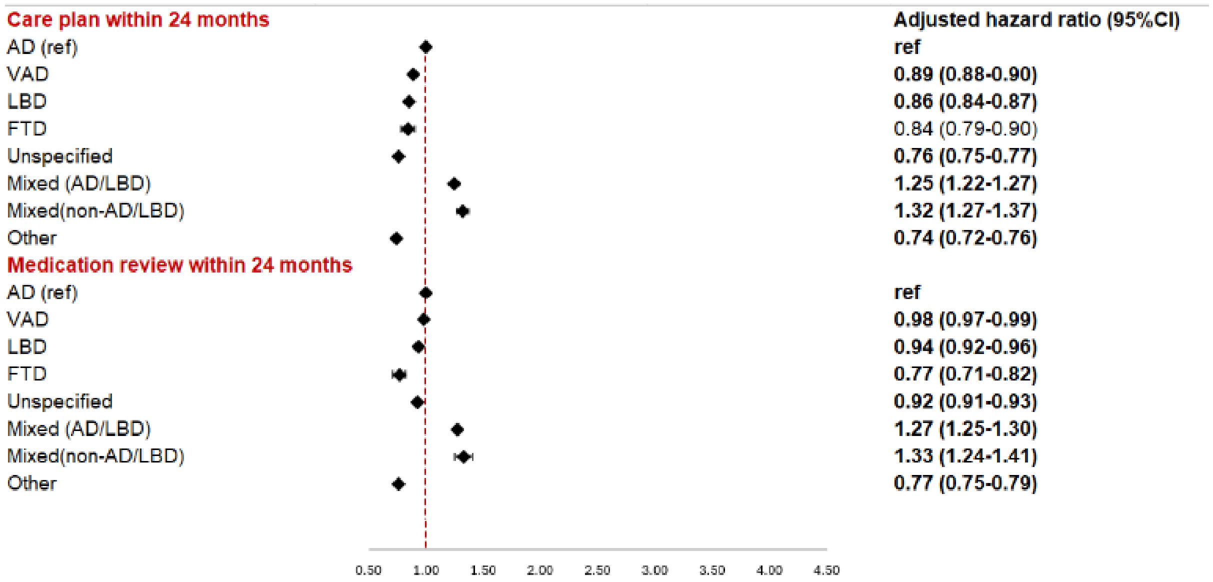

*competing risk was death.

Figure S5: Adjusted hazard ratios for competing risks* models for PIP indicators (n=57,166, clustered for n=1481 practices)

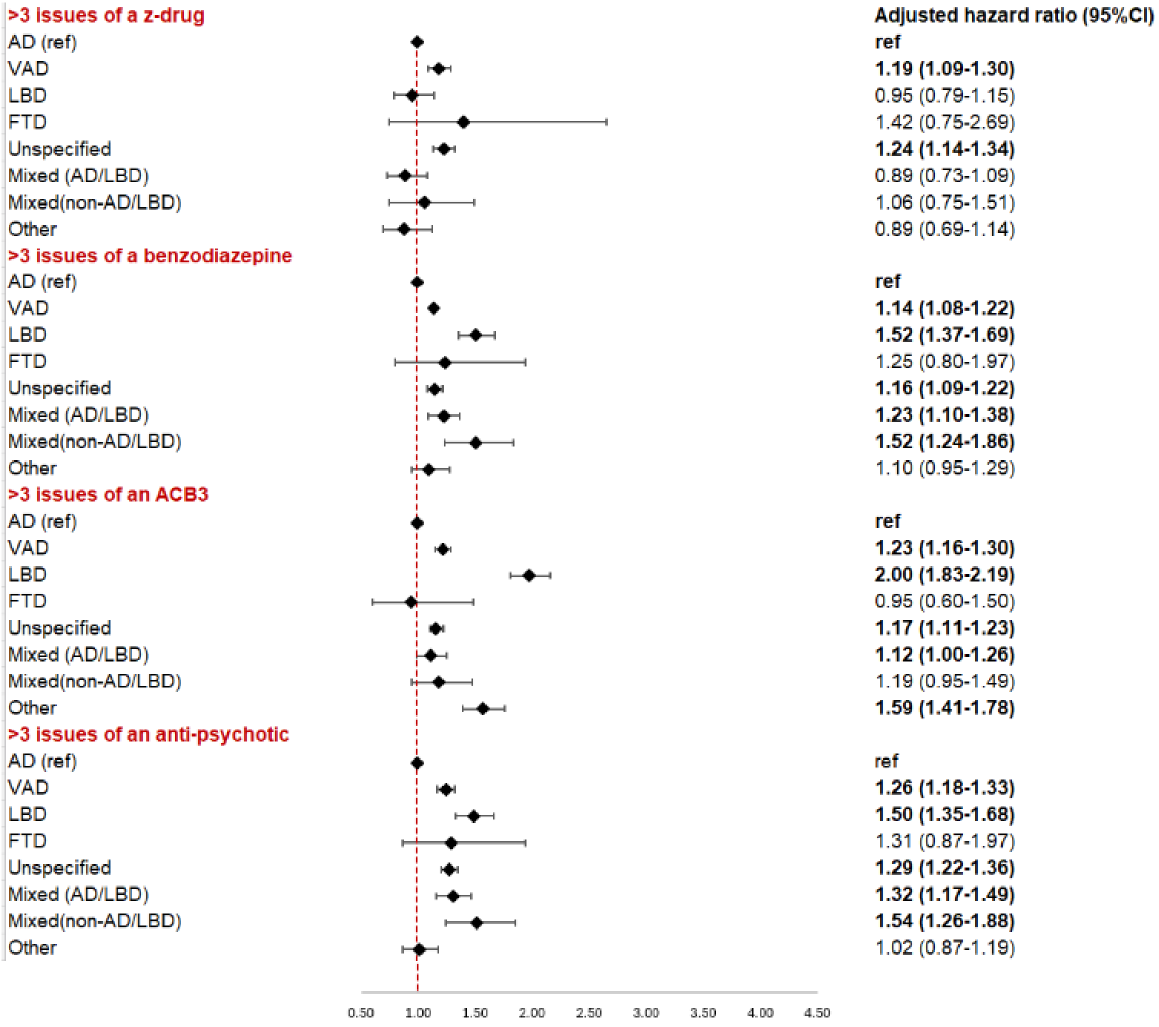

*competing risk model was death.

Figure S6: Adjusted hazard ratios for incident dementia cases only (n=526,107)

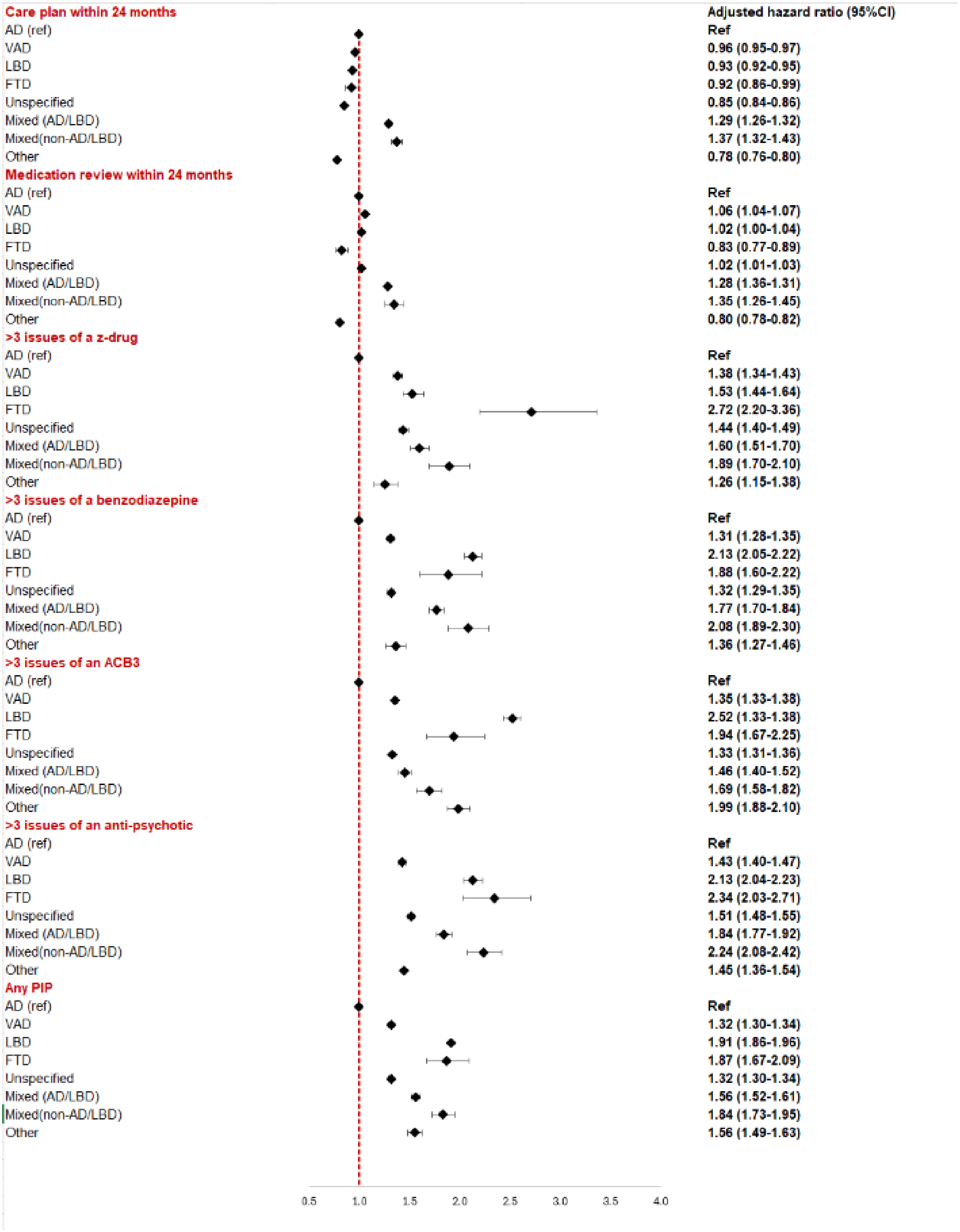

Figure S7: Parametric regression models (Weibull distribution) n=571663†

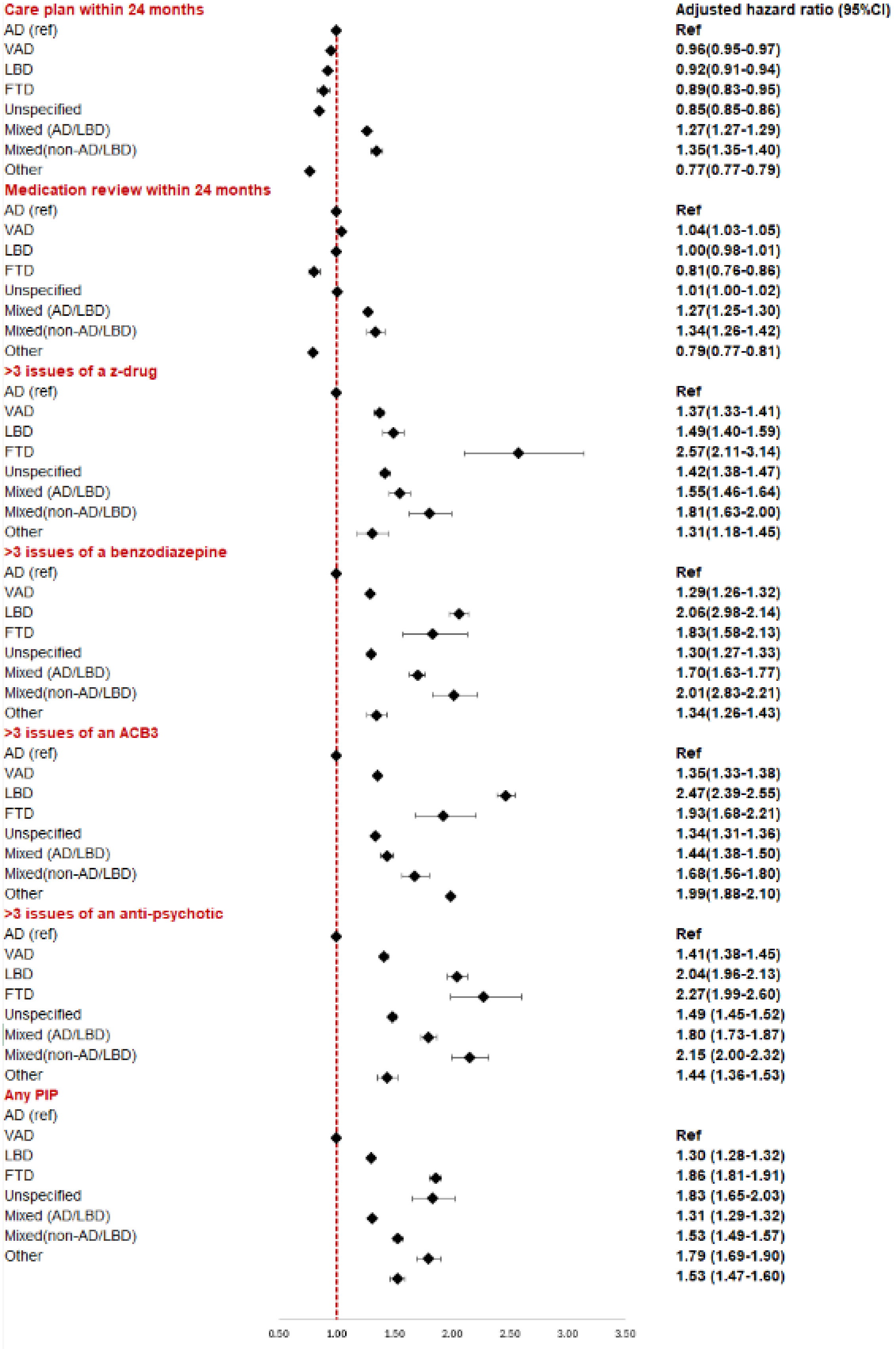

†Adjusted for age, gender, Index of Multiple Deprivation quintile, ethnicity, comorbidity score, learning disability diagnosis, standard errors clustered by practice.

Table S1: Supplementary Table 1: Accelerated failure time model with lognormal distribution, coefficient and time ratios for each outcome (n=571663)

Supplementary Table 1: Accelerated failure time model with lognormal distribution, coefficient and time ratios for each outcome (n=571663)

	Coefficient (B)	Time ratio (e^B)	P	95%CI [lower]	95% CI [upper]
**Annual Review**					
AD					
VAD	0.11	1.11	<0.001	0.09	0.13
LBD	0.26	1.30	<0.001	0.23	0.29
FTD	0.37	1.44	<0.001	0.23	0.50
Unspecified	0.30	1.35	<0.001	0.28	0.33
Mixed (including AD/LBD)	-0.62	0.54	<0.001	-0.66	-0.57
Mixed (not including AD/LBD)	-0.79	0.45	<0.001	-0.88	-0.70
Other only	0.59	1.79	<0.001	0.54	0.63
Med Review					
AD					
VAD	-0.03	0.97	<0.001	-0.05	-0.01
LBD	0.12	1.13	<0.001	0.09	0.15
FTD	0.48	1.62	<0.001	0.35	0.61
Unspecified	0.05	1.05	<0.001	0.03	0.07
Mixed (including AD/LBD)	-0.65	0.52	<0.001	-0.70	-0.61
Mixed (not including AD/LBD)	-0.82	0.44	<0.001	-0.93	-0.70
Other only	0.51	1.67	<0.001	0.47	0.56
**3 issues of ACB3s**					
AD					
VAD	-0.28	0.76	<0.001	-0.29	-0.26
LBD	-0.72	0.49	<0.001	-0.74	-0.69
FTD	-0.52	0.59	<0.001	-0.64	-0.41
Unspecified	-0.27	0.76	<0.001	-0.29	-0.26
Mixed (including AD/LBD)	-0.31	0.73	<0.001	-0.35	-0.28
Mixed (not including AD/LBD)	-0.42	0.66	<0.001	-0.48	-0.36
Other only	-0.55	0.58	<0.001	-0.59	-0.51
**3 issues of Anti-Psychotics**					
AD					
VAD	-0.33	0.72	<0.001	-0.35	-0.31
LBD	-0.58	0,56	<0.001	-0.62	-0.54
FTD	-0.69	0.50	<0.001	-0.82	-0.57
Unspecified	-0.37	0.69	<0.001	-0.39	-0.35
Mixed (including AD/LBD)	-0.52	059	<0.001	-0.55	-0.48
Mixed (not including AD/LBD)	-0.67	0.51	<0.001	-0.73	-0.60
Other only	-0.30	0.74	<0.001	-0.35	-0.25
**3 issues of Z-drugs**					
AD					
VAD	-0.27	0.76	<0.001	-0.30	-0.25
LBD	-0.28	0.76	<0.001	-0.33	-0.23
FTD	-0.69	0.50	<0.001	-0.86	-0.52
Unspecified	-0.30	0.74	<0.001	-0.33	-0.28
Mixed (including AD/LBD)	-0.35	0.70	<0.001	-0.40	-0.30
Mixed (not including AD/LBD)	-0.44	0.64	<0.001	-0.53	-0.36
Other only	-0.21	0.81	<0.001	-0.28	-0.13
**3 issues of Benzodiazepines**					
AD					
VAD	-0.24	0.79	<0.001	-0.26	-0.22
LBD	-0.58	0.56	<0.001	-0.61	-0.54
FTD	-0.45	0.64	<0.001	-0.58	-0.32
Unspecified	-0.25	0.78	<0.001	-0.27	-0.23
Mixed (including AD/LBD)	-0.45	0.64	<0.001	-0.48	-0.41
Mixed (not including AD/LBD)	-0.61	0.54	<0.001	-0.69	-0.53
Other only	-0.25	0.78	<0.001	-0.30	-0.20

**Any PIP**					
AD					
VAD	-0.27	0.76	<0.001	-0.28	-0.26
LBD	-0.50	0.61	<0.001	-0.53	-0.48
FTD	-0.50	0.61	<0.001	-0.59	-0.41
Unspecified	-0.28	0.76	<0.001	-0.29	-0.26
Mixed (including AD/LBD)	-0.39	0.68	<0.001	-0.42	-0.36
Mixed (not including AD/LBD)	-0.53	0.59	<0.001	-0.58	-0.47
Other only	-0.36	0.70	<0.001	-0.40	-0.33
