# Supplementary material for: How does diagnostic subtype affect the quality of primary care for people with dementia? A retrospective cohort study in 1490 English general practices
Source: Age Ageing. 2026 Apr 20;55(4):afag101. doi: 10.1093/ageing/afag101 (PMC13092812; doi:10.1093/ageing/afag101)
Supplement: Supplementary_material_afag101 [file supplementary_material_afag101.zip › aa-25-3645-File003.pdf]

These are the codelists used in the study. Medcodes are provided in all instances - these are the unique identifying codes used by CPRD Aurum to identify each term. They are provided for reproducibility of the study within CPRD Aurum. In some cases (for the Comorbidities) readcodes are also provided, these are for transparency around the modification of the CMS score, which initially used CPRD Gold codes and also conditions identifiable with readcodes.

1. **Dementia codelist**

A comprehensive codelist of dementia codes was developed to identify cases of people with dementia. This was developed from an existing codelist (1), which was then modified to include further CPRD aurum specific codes. The ‘pcdsearch’ algorithm(2) was used to search the aurum data dictionary for the terms ‘dementia’ ‘alzheim*’ ‘Pick’ ‘frontotemporal’ ‘lewy body’ and additional codes identified to form the final list. This list was compared to a codelist used in a recent paper using a cohort of people with dementia in CPRD aurum to ensure no important codes were missed(3).

Creating the codelist consisted of: searching the CPRD Aurum data dictionary(1, 2) and comparing to a relevant codelist from a 2024 paper(3). The final list identified 302 codes and was reviewed by 2 clinicians (CM/TB), only diagnostic codes were included. Any patient with one of these codes in their EHR was extracted from CPRD and the exclusion criteria applied

medcode	readcode	description
1235534016		binswanger's encephalopathy
1270228100000611 1		degenerative brain disorder
148381000006115		senile/presenile dementia
149347010		binswanger's disease
1823871000006112		dementia confirmed
1949621000006118		dementia stage at diagnosis
1949631000006115		dementia stage at diagnosis - early (mild)
1949641000006113		dementia stage at diagnosis - mid (moderate)
1949651000006110		dementia stage at diagnosis - late (severe)
1949661000006112		dementia stage at diagnosis - undetermined
1949671000006117		dementia stage at diagnosis - not known
1971401000006111		"dementia in alzheimer's disease with early onset without additional symptoms"
1971541000006114		"dementia in alzheimer's disease with early onset other symptoms predominantly delusional”
1971771000006112		"dementia in alzheimer's disease with early onset other symptoms predominantly hallucinatory”
1972021000006119		"unspecified dementia without additional symptoms”
1972041000006114		"unspecified dementia other symptoms predominantly delusional”
1972061000006113		"unspecified dementia other symptoms predominantly hallucinatory”
1972071000006118		"unspecified dementia other symptoms predominantly depressive”
1972081000006115		"unspecified dementia other mixed symptoms”
1972131000006115		"dementia in alzheimer's disease with early onset other symptoms predominantly depressive”
1972141000006113		"dementia in alzheimer's disease with early onset other mixed symptoms”
1972171000006117		"dementia in alzheimer's disease with late onset without additional symptoms
1972181000006119		"dementia in alzheimer's disease with late onset other symptoms predominantly delusional
1972191000006116		"dementia in alzheimer's disease with late onsetother symptoms predominantly hallucinatory”

Appendix: codelists used in the study

1972201000006118		"dementia in alzheimer's disease with late onset other symptoms predominantly depressive"
1972211000006115		"dementia in alzheimer's disease with late onset other mixed symptoms"
1972231000006114		"dementia in alzheimer's dis atypical or mixed type without additional symptoms
1972251000006119		"dementia in alzheimer's dis atypical or mixed type other symptoms predominantly delusional"
1972291000006113		"dementia in alzheimer's dis atypical or mixed type predominantly hallucinatory
1972311000006112		"dementia in alzheimer's dis atypical or mixed type other symptoms predominantly depressive"
1972341000006111		"dementia in alzheimer's dis atypical or mixed type other mixed symptoms
1972371000006115		"dementia in alzheimer's disease unspecified without additional symptoms"
1972401000006117		"dementia in alzheimer's disease unspecified other symptoms predominantly delusional"
1972421000006110		"dementia in alzheimer's disease unspecified other symptoms predominantly hallucinatory"
1972451000006118		"dementia in alzheimer's disease unspecified other symptoms predominantly depressive"
1972471000006111		"dementia in alzheimer's disease unspecified other mixed symptoms
1972481000006114		"vascular dementia of acute onset without additional symptoms"
1972501000006116		"vascular dementia of acute onset other symptoms predominantly delusional"
1972521000006114		"vascular dementia of acute onset other symptoms predominantly hallucinatory"
1972541000006119		"vascular dementia of acute onset other symptoms predominantly depressive"
1972571000006110		"vascular dementia of acute onset other mixed symptoms
1972601000006115		"multi-infarct dementia without additional symptoms"
1972621000006113		"multi-infarct dementia other symptoms predominantly delusional"
1972641000006118		"multi-infarct dementia other symptoms predominantly hallucinatory
1972661000006119		"multi-infarct dementia other symptoms predominantly depressive"
1972681000006112		"multi-infarct dementia other mixed symptoms"
1972711000006113		"subcortical vascular dementia without additional symptoms"
1972731000006119		"subcortical vascular dementia other symptoms predominantly delusional"
1972751000006114		"subcortical vascular dementia other symptoms predominantly hallucinatory
1972771000006116		"subcortical vascular dementia other symptoms predominantly depressive"
1972791000006115		"subcortical vascular dementia other mixed symptoms
1972821000006112		"mixed cortical and subcortical vascular dementia without additional symptoms
1972831000006110		"mixed cortical and subcortical vascular dementia other symptoms predominantly delusional
1972871000006113		"mixed cortical and subcortical vascular dementia other symptoms predominantly hallucinatory
1972911000006111		"mixed cortical and subcortical vascular dementia other symptoms predominantly depressive
1972931000006117		"mixed cortical and subcortical vascular dementia other mixed symptoms
1973221000006118		"other vascular dementia without additional symptoms without additional symptoms"
1973271000006117		"other vascular dementia other symptoms predominantly delusional
1973341000006118		"other vascular dementia other symptoms predominantly hallucinatory
1973381000006112		"other vascular dementia other symptoms predominantly depressive
1973401000006112		"other vascular dementia other mixed symptoms
1973461000006113		"vascular dementia unspecified without additional symptoms
1973501000006113		"vascular dementia unspecified other symptoms predominantly delusional

197353100006117		"vascular dementia unspecified other symptoms predominantly hallucinatory"
197355100006112		"vascular dementia unspecified other symptoms predominantly depressive"
197683100006111		"vascular dementia unspecified other mixed symptoms"
21256010		presenile dementia
22408016		pick's disease
2366171000000112		sporadic creutzfeldt-jakob disease
2366181000000114		sporadic cjd (creutzfeldt-jakob disease)
2386018		jakob-creutzfeldt disease
2502971000006115		dementia associated with alcoholism
2502981000006117		alcohol-induced persisting dementia
2510951000006114		creutzfeldt-jakob disease
2510971000006116		subacute spongiform encephalopathy
2510981000006118		cjd - creutzfeldt-jakob disease
2510991000006115		jcd - jakob-creutzfeldt disease
2511001000006119		transmissible virus dementia
2511011000006116		creutzfeldt jakob disease
251625013		h/o: dementia
26545010		senile dementia
2707661000006119		pick disease
2707671000006114		picks disease
2748441000006111		sd - senile dementia
2931231000006118		ad - alzheimer's disease
2931241000006111		alzheimer disease
2931251000006113		alzheimer dementia
294635013		uncomplicated senile dementia
294637017		uncomplicated presenile dementia
294638010		presenile dementia with delirium
294641018		presenile dementia with paranoia
294642013		presenile dementia with depression
294643015		presenile dementia nos
294644014		senile dementia with depressive or paranoid features
294645010		senile dementia with paranoia
294646011		senile dementia with depression
294647019		senile dementia with depressive or paranoid features nos
294648012		senile dementia with delirium
294652012		uncomplicated arteriosclerotic dementia
294653019		arteriosclerotic dementia with delirium
294654013		arteriosclerotic dementia with paranoia
294655014		arteriosclerotic dementia with depression
294656010		arteriosclerotic dementia nos
294660013		senile or presenile psychoses nos
294718018		dementia in conditions ec
295668011		[x]dementia in alzheimer's disease
295671015		"[x]dementia in alzheimer's dis atypical or mixed type"
295672010		"[x]dementia in alzheimer's disease unspecified"
295680015		[x]other vascular dementia
295681016		"[x]vascular dementia unspecified"
295684012		[x]dementia in other diseases classified elsewhere

295685013		[x]dementia in pick's disease
295686014		[x]dementia in creutzfeldt-jakob disease
295687017		[x]dementia in huntington's disease
295688010		[x]dementia in parkinson's disease
295690011		[x]dementia in other specified diseases classif elsewhere
299325013		[x]other alzheimer's disease
299641000000112		[x]lewy body dementia
3341641000006113		dementia paralytica
3341701000006116		paralytic dementia
3350441000006115		organic dementia
3414231000006117		mid - multi-infarct dementia
3414251000006112		vad - vascular dementia
3414261000006114		multi infarct dementia
346929012		alcoholic dementia nos
359081000006118		[x] presenile dementia nos
359091000006115		[x] presenile psychosis nos
359101000006114		[x] primary degenerative dementia nos
359141000006111		[x] senile dementia nos
359151000006113		"[x] senile dementia depressed or paranoid type"
359241000006119		[x] unspecified dementia
362941000006113		[x]alcoholic dementia nos
363021000006113		[x]alzheimer's dementia unspec
363031000006111		[x]alzheimer's disease type 1
363041000006118		[x]alzheimer's disease type 2
363791000006112		[x]arteriosclerotic dementia
376531000006119		[x]dementia in alzheimer's disease with early onset
376541000006112		[x]dementia in alzheimer's disease with late onset
376571000006116		[x]dementia in human immunodef virus [hiv] disease
3802621000006119		lewy body variant of alzheimer's disease
3802631000006116		sdlt - senile dementia of the lewy body type
3802641000006114		lbd - lewy body disease
3802651000006111		dementia of the lewy body type
3802661000006113		dlbd - diffuse lewy body disease
3802671000006118		cortical lewy body disease
3802681000006115		clbd - cortical lewy body disease
3964591000006117		subcortical leucoencephalopathy
3964611000006111		binswanger's dementia
3964661000006114		subcortical atherosclerotic dementia
398571000006112		[x]mixed cortical and subcortical vascular dementia
399031000006111		[x]multi-infarct dementia
423221000006117		[x]predominantly cortical dementia
423351000006115		"[x]presenile dementia alzheimer's type"
423381000006111		"[x]primary degen dementia of alzheimer's type senile onset
423391000006114		"[x]primary degen dementia alzheimer's type presentile onset
425901000006116		"[x]senile dementia alzheimer's type"
428201000006119		[x]subcortical vascular dementia
431681000006117		[x]vascular dementia
431691000006119		[x]vascular dementia of acute onset

45046017		alzheimer's disease
4539871000006116		history of dementia
481281019		progressive multifocal leucoencephalopathy
497559016		arteriosclerotic dementia
499946014		alzheimer's disease with early onset
500317011		alzheimer's disease with late onset
6897211000006117		"primary degenerative dementia of the alzheimer type presenile onset"
6897221000006113		"primary degenerative dementia of the alzheimer type early onset
6897241000006118		dementia of the alzheimers type with early onset
6897251000006116		"presenile dementia alzheimer's type"
6897271000006114		dementia in alzheimer's disease - type 2
6900181000006114		"primary degenerative dementia of the alzheimer type senile onset
6900191000006112		"primary degenerative dementia of the alzheimer type late onset
6900201000006110		"dementia of the alzheimers type late onset
6900221000006117		"sdat - senile dementia alzheimer's type"
6900241000006112		dementia in alzheimer's disease - type 1
6973421000006116		dementia associated with aids
6973431000006118		acquired immune deficiency syndrome-related dementia
6973441000006111		aids - acquired immune deficiency syndrome dementia complex
6973451000006113		adc - acquired immune deficiency syndrome dementia complex
6973461000006110		acquired immune deficiency syndrome dementia complex
6973471000006115		dementia associated with acquired immunodeficiency syndrome
7043651000006119		dementia associated with parkinson's disease
7043661000006117		dementia associated with parkinson disease
7103601000006116		dementia due to creutzfeldt-jakob disease
7263011000006112		dementia due to huntington disease
7263021000006116		dementia due to huntingtons disease
7699251000006118		sporadic jakob-creutzfeldt disease
8009521000006114		dementia due to picks disease
8009531000006112		dementia due to pick disease
8009541000006119		dementia co-occurrent and due to pick's disease
882171000006115		dementia
882181000006117		other senile/presenile dement.
882191000006119		senile and presenile dementias
882201000006116		senile dementia - simple type
882211000006118		senile dementia-acute confused
905791000006115		[rfc] alzheimer's disease
914921000006117		[d] vascular dementia
914931000006119		[d] dementia with lewy bodies
914941000006112		[d] dementia
914951000006114		[d] dementia in alzheimer's disease
939491000006118		[rfc] dementia
97154016		niemann-pick disease
8024201000006111		Mixed dementia
1237065100000611 2		MVAD - Mixed vascular Alzheimer dementia
2575731000006115		Primary degenerative dementia of the Alzheimer type, senile onset, with delirium
2575741000006113		Dementia of the Alzheimer's type, with late onset, with delirium
2575751000006110		Dementia Alzheimers type, late onset with delirium

2603271000006110		Primary degenerative dementia of the Alzheimer type, presenile onset, uncomplicated
2603281000006113		Dementia of the Alzheimer's type, with early onset, uncomplicated
2667431000006115		Primary degenerative dementia of the Alzheimer type, presenile onset, with depression
2667441000006113		Dementia of the Alzheimer's type, with early onset, with depressive mood
2667451000006110		Alzheimers dementia, early onset, with depressed mood
2929931000006112		Primary degenerative dementia of the Alzheimer type, senile onset, with depression
2929941000006119		Dementia of the Alzheimer's type, with late onset, with depressive mood
2929951000006117		Alzheimers dementia, late onset, with depressive mood
3384371000006119		Primary degenerative dementia of the Alzheimer type, presenile onset, with delusions
3384381000006116		Dementia of the Alzheimer's type, with early onset, with delusions
3392881000006115		Primary degenerative dementia of the Alzheimer type, senile onset, with delusions
3392891000006117		Dementia of the Alzheimer's type, with late onset, with delusions
3392901000006118		Alzheimers dementia, late onset, with delusions
3558401000006117		Primary degenerative dementia of the Alzheimer type, presenile onset, with delirium
3558411000006119		Dementia of the Alzheimer's type, with early onset, with delirium
3575041000006117		Primary degenerative dementia of the Alzheimer type, senile onset, uncomplicated
3575051000006115		Dementia of the Alzheimer's type, with late onset, uncomplicated
3894371000006118		Alzheimer's neurofibrillary degeneration
3894381000006115		Alzheimer's neurofibrillary change
5005501000006111		Familial Alzheimer's disease of early onset
5005511000006114		Familial Alzheimer disease of early onset
5005521000006118		Non-familial Alzheimer's disease of early onset
5005531000006115		Non-familial Alzheimer disease of early onset
5005541000006113		Familial Alzheimer's disease of late onset
5005551000006110		Familial Alzheimer disease of late onset
5005561000006112		Non-familial Alzheimer's disease of late onset
5005571000006117		Non-familial Alzheimer disease of late onset
5005581000006119		Focal Alzheimer's disease
5005711000006119		Progressive aphasia in Alzheimer's disease
5005721000006110		Progressive aphasia in Alzheimer disease
6548441000006111		Family history: Alzheimer disease
6548451000006113		Family history of Alzheimers
7515121000006112		Primary degenerative dementia of the Alzheimer type, senile onset in remission
7515131000006110		Primary degenerative dementia of the Alzheimer type, presenile onset in remission
7718421000006113		Assessment using Alzheimer's Disease Assessment Scale
7718441000006118		Alzheimer's Disease Assessment Scale score
7813831000006118		Familial Alzheimer-like prion disease
7834071000006116		Non-amnestic Alzheimer disease
7969281000006110		Dementia of the Alzheimer type with behavioural disturbance
7969291000006113		Dementia of the Alzheimer type with behavioral disturbance
8030791000006119		Altered behaviour in Alzheimer's disease
8030801000006118		Altered behavior in Alzheimer's disease
8030811000006115		Altered behavior in Alzheimer disease

8030821000006111		Altered behavior in Alzheimers disease
8032911000006111		Early onset Alzheimer's disease with behavioural disturbance
8032921000006115		Early onset Alzheimer's disease with behavioral disturbance
8032931000006117		Early onset Alzheimers disease with behavioral disturbance
8032941000006110		Early onset Alzheimer disease with behavioral disturbance
8044911000006116		Delusions in Alzheimer's disease
8044921000006112		Delusions in Alzheimers disease
8044931000006110		Delusions in Alzheimer disease
8044941000006117		Depressed mood in Alzheimer's disease
8044951000006115		Alzheimers dementia with depressed mood
8044961000006118		Depressed mood in Alzheimer disease
8044981000006111		Alzheimer's disease co-occurrent with delirium
9896131000006110		Behavioural disturbance co-occurrent and due to late onset Alzheimer dementia
9896141000006117		Behavioral disturbance co-occurrent and due to late onset Alzheimer dementia
1576281000006119		Cause of Death- Alzheimer'S Disease
5005601000006112		Frontotemporal dementia
5005611000006110		Pick's disease with Pick bodies
5005631000006116		Pick's disease with Pick cells and no Pick bodies
5005641000006114		Pick disease with Pick cells and no Pick bodies
7559631000006110		Frontotemporal dementia with gene located on 3p11
7560531000006110		Frontotemporal dementia with parkinsonism-17
7560541000006117		Familial Pick's disease
7560571000006113		FTDP-17 - Frontotemporal dementia with parkinsonism 17
7750421000006118		Right temporal atrophy variant frontotemporal dementia
7755121000006115		Behavioural variant of frontotemporal dementia
8024631000006118		Altered behavior co-occurrent and due to Pick's disease
8089241000006117		Vascular dementia with behavioral disturbance
8089231000006110		Vascular dementia with behavioural disturbance
2664621000006110		Multi infarct dementia with delirium
2722941000006116		Vascular dementia, with depressive mood
3653681000006111		Vascular dementia, uncomplicated
8033251000006110		Multi infarct dementia with atherosclerosis
2912331000006111		Vascular dementia, with delusions
9902261000006115		Vascular dementia without behavioral disturbance
9902251000006117		Vascular dementia without behavioural disturbance
2664611000006117		Vascular dementia, with delirium
7840631000006118		Ischaemic vascular dementia
3653691000006114		Multi infarct dementia, uncomplicated
7515061000006117		Vascular dementia in remission
2912341000006118		Multi infarct dementia with delusions
7840641000006111		Ischemic vascular dementia
2729791000000114		Predominantly cortical vascular dementia
745381000006119	F116.00	Lewy body disease
3195111000006118		Lewy body
8042791000006116		Lewy body dementia with behavioural disturbance
6024061000006111		Senile dementia of the Lewy body type
3195101000006116		Lewy bodies
8042801000006115		Lewy body dementia with behavioral disturbance

318953100006118		Diffuse Lewy body disease with spongiform cortical change

2. **Dementia subtypes codelists**

Note that at this stage some codes appear in multiple lists, e.g. mixed vascular and alzheimer's dementia. Patients with these codes will have been tagged in the AD dataset, and the vascular dataset, and as such coded with a 'mixed' subtype.

Alzheimer's Disease

Medcode	Readcode	Description
1971401000006111		Dementia in Alzheimer's disease with early onset, without additional symptoms
1971541000006114		Dementia in Alzheimer's disease with early onset, other symptoms, predominantly delusional
1971771000006112		Dementia in Alzheimer's disease with early onset, other symptoms, predominantly hallucinatory
1972131000006115		Dementia in Alzheimer's disease with early onset, other symptoms, predominantly depressive
1972141000006113		Dementia in Alzheimer's disease with early onset, other mixed symptoms
1972171000006117		Dementia in Alzheimer's disease with late onset, without additional symptoms
1972181000006119		Dementia in Alzheimer's disease with late onset, other symptoms, predominantly delusional
1972191000006116		Dementia in Alzheimer's disease with late onset, other symptoms, predominantly hallucinatory
1972201000006118		Dementia in Alzheimer's disease with late onset, other symptoms, predominantly depressive
1972211000006115		Dementia in Alzheimer's disease with late onset, other mixed symptoms
1972231000006114		Dementia in Alzheimer's dis, atypical or mixed type, without additional symptoms
1972251000006119		Dementia in Alzheimer's dis, atypical or mixed type, other symptoms, predominantly delusional
1972291000006113		Dementia in Alzheimer's dis, atypical or mixed type, other symptoms, predominantly hallucinatory
1972311000006112		Dementia in Alzheimer's dis, atypical or mixed type, other symptoms, predominantly depressive
1972341000006111		Dementia in Alzheimer's dis, atypical or mixed type, other mixed symptoms
1972371000006115		Dementia in Alzheimer's disease, unspecified, without additional symptoms
1972401000006117		Dementia in Alzheimer's disease, unspecified, other symptoms, predominantly delusional
1972421000006110		Dementia in Alzheimer's disease, unspecified, other symptoms, predominantly hallucinatory
1972451000006118		Dementia in Alzheimer's disease, unspecified, other symptoms, predominantly depressive
1972471000006111		Dementia in Alzheimer's disease, unspecified, other mixed symptoms
2575731000006115		Primary degenerative dementia of the Alzheimer type, senile onset, with delirium
2575741000006113		Dementia of the Alzheimer's type, with late onset, with delirium
2575751000006110		Dementia Alzheimers type, late onset with delirium
2603271000006110		Primary degenerative dementia of the Alzheimer type, presenile onset, uncomplicated
2603281000006113		Dementia of the Alzheimer's type, with early onset, uncomplicated
2667431000006115		Primary degenerative dementia of the Alzheimer type, presenile onset, with depression
2667441000006113		Dementia of the Alzheimer's type, with early onset, with depressive mood
2667451000006110		Alzheimers dementia, early onset, with depressed mood
2929931000006112		Primary degenerative dementia of the Alzheimer type, senile onset, with depression

2929941000006119		Dementia of the Alzheimer's type, with late onset, with depressive mood
2929951000006117		Alzheimers dementia, late onset, with depressive mood
2931231000006118		AD - Alzheimer's disease
2931241000006111		Alzheimer disease
2931251000006113		Alzheimer dementia
295668011	Eu00.00	[X]Dementia in Alzheimer's disease
295671015	Eu00200	[X]Dementia in Alzheimer's dis, atypical or mixed type
295672010	Eu00z00	[X]Dementia in Alzheimer's disease, unspecified
299325013	Fyu3000	[X]Other Alzheimer's disease
3384371000006119		Primary degenerative dementia of the Alzheimer type, presenile onset, with delusions
3384381000006116		Dementia of the Alzheimer's type, with early onset, with delusions
3392881000006115		Primary degenerative dementia of the Alzheimer type, senile onset, with delusions
3392891000006117		Dementia of the Alzheimer's type, with late onset, with delusions
3392901000006118		Alzheimers dementia, late onset, with delusions
3558401000006117		Primary degenerative dementia of the Alzheimer type, presenile onset, with delirium
3558411000006119		Dementia of the Alzheimer's type, with early onset, with delirium
3575041000006117		Primary degenerative dementia of the Alzheimer type, senile onset, uncomplicated
3575051000006115		Dementia of the Alzheimer's type, with late onset, uncomplicated
363021000006113	Eu00z11	[X]Alzheimer's dementia unspec
363031000006111	Eu00111	[X]Alzheimer's disease type 1
363041000006118	Eu00013	[X]Alzheimer's disease type 2
376531000006119	Eu00000	Dementia in Alzheimer's disease with early onset
376541000006112	Eu00100	Dementia in Alzheimer's disease with late onset
3802621000006119		Lewy body variant of Alzheimer's disease
3894371000006118		Alzheimer's neurofibrillary degeneration
3894381000006115		Alzheimer's neurofibrillary change
423351000006115	Eu00011	[X]Presenile dementia,Alzheimer's type
423381000006111	Eu00113	[X]Primary degen dementia of Alzheimer's type, senile onset
423391000006114	Eu00012	[X]Primary degen dementia, Alzheimer's type, presenile onset
425901000006116	Eu00112	[X]Senile dementia,Alzheimer's type
45046017	F110.00	Alzheimer's disease
499946014	F110000	Alzheimer's disease with early onset
500317011	F110100	Alzheimer's disease with late onset
5005501000006111		Familial Alzheimer's disease of early onset
5005511000006114		Familial Alzheimer disease of early onset
5005521000006118		Non-familial Alzheimer's disease of early onset
5005531000006115		Non-familial Alzheimer disease of early onset
5005541000006113		Familial Alzheimer's disease of late onset
5005551000006110		Familial Alzheimer disease of late onset
5005561000006112		Non-familial Alzheimer's disease of late onset
5005571000006117		Non-familial Alzheimer disease of late onset
5005581000006119		Focal Alzheimer's disease
5005711000006119		Progressive aphasia in Alzheimer's disease
5005721000006110		Progressive aphasia in Alzheimer disease
6548441000006111		Family history: Alzheimer disease
6548451000006113		Family history of Alzheimers
6897211000006117		Primary degenerative dementia of the Alzheimer type, presenile onset

6897221000006113		Primary degenerative dementia of the Alzheimer type, early onset
6897241000006118		Dementia of the Alzheimers type with early onset
6897251000006116		Presenile dementia, Alzheimer's type
6897271000006114		Dementia in Alzheimer's disease - type 2
6900181000006114		Primary degenerative dementia of the Alzheimer type, senile onset
6900191000006112		Primary degenerative dementia of the Alzheimer type, late onset
6900201000006110		Dementia of the Alzheimers type, late onset
11914891000006110		[X]Dementia in Alzheimer's dis, atypical or mixed type
12370651000006112		MVAD - Mixed vascular Alzheimer dementia
6900241000006112		Dementia in Alzheimer's disease - type 1
7515121000006112		Primary degenerative dementia of the Alzheimer type, senile onset in remission
7515131000006110		Primary degenerative dementia of the Alzheimer type, presenile onset in remission
7718421000006113		Assessment using Alzheimer's Disease Assessment Scale
7718441000006118		Alzheimer's Disease Assessment Scale score
7813831000006118		Familial Alzheimer-like prion disease
7834071000006116		Non-amnestic Alzheimer disease
7969281000006110		Dementia of the Alzheimer type with behavioural disturbance
7969291000006113		Dementia of the Alzheimer type with behavioral disturbance
8030791000006119		Altered behaviour in Alzheimer's disease
8030801000006118		Altered behavior in Alzheimer's disease
8030811000006115		Altered behavior in Alzheimer disease
8030821000006111		Altered behavior in Alzheimers disease
8032911000006111		Early onset Alzheimer's disease with behavioural disturbance
8032921000006115		Early onset Alzheimer's disease with behavioral disturbance
8032931000006117		Early onset Alzheimers disease with behavioral disturbance
8032941000006110		Early onset Alzheimer disease with behavioral disturbance
8044911000006116		Delusions in Alzheimer's disease
8044921000006112		Delusions in Alzheimers disease
8044931000006110		Delusions in Alzheimer disease
8044941000006117		Depressed mood in Alzheimer's disease
8044951000006115		Alzheimers dementia with depressed mood
8044961000006118		Depressed mood in Alzheimer disease
8044981000006111		Alzheimer's disease co-occurrent with delirium
905791000006115		[RFC] Alzheimer's disease
914951000006114		[D] Dementia in Alzheimer's disease
9896131000006110		Behavioural disturbance co-occurrent and due to late onset Alzheimer dementia
9896141000006117		Behavioral disturbance co-occurrent and due to late onset Alzheimer dementia
1576281000006119		Cause of Death- Alzheimer'S Disease

Vascular dementia

Medcode	Readcode	Description
294652012	E004000	Uncomplicated arteriosclerotic dementia
294653019	E004100	Arteriosclerotic dementia with delirium
294654013	E004200	Arteriosclerotic dementia with paranoia
294655014	E004300	Arteriosclerotic dementia with depression
1972521000006114		Vascular dementia of acute onset, other symptoms, predominantly hallucinatory

1972571000006110		Vascular dementia of acute onset, other mixed symptoms
1972751000006114		Subcortical vascular dementia, other symptoms, predominantly hallucinatory
1972831000006110		Mixed cortical and subcortical vascular dementia, other symptoms, predominantly delusional
1972871000006113		Mixed cortical and subcortical vascular dementia, other symptoms, predominantly hallucinatory
1972911000006111		Mixed cortical and subcortical vascular dementia, other symptoms, predominantly depressive
1972931000006117		Mixed cortical and subcortical vascular dementia, other mixed symptoms
1973501000006113		Vascular dementia, unspecified, other symptoms, predominantly delusional
497559016	E004.00	Arteriosclerotic dementia
295680015	Eu01y00	[X]Other vascular dementia
294656010	E004z00	Arteriosclerotic dementia NOS
3414251000006112		VAD - Vascular dementia
8089241000006117		Vascular dementia with behavioral disturbance
8089231000006110		Vascular dementia with behavioural disturbance
2664621000006110		Multi infarct dementia with delirium
2722941000006116		Vascular dementia, with depressive mood
3653681000006111		Vascular dementia, uncomplicated
8033251000006110		Multi infarct dementia with atherosclerosis
2912331000006111		Vascular dementia, with delusions
428201000006119	Eu01200	Subcortical vascular dementia
431681000006117	Eu01.00	Vascular dementia
431691000006119	Eu01000	Vascular dementia of acute onset
1972501000006116		Vascular dementia of acute onset, other symptoms, predominantly delusional
1973381000006112		Other vascular dementia, other symptoms, predominantly depressive
914921000006117		[D] Vascular dementia
9902261000006115		Vascular dementia without behavioral disturbance
9902251000006117		Vascular dementia without behavioural disturbance
2664611000006117		Vascular dementia, with delirium
7840631000006118		Ischaemic vascular dementia
1973551000006112		Vascular dementia, unspecified, other symptoms, predominantly depressive
1976831000006111		Vascular dementia, unspecified, other mixed symptoms
3653691000006114		Multi infarct dementia, uncomplicated
7515061000006117		Vascular dementia in remission
2912341000006118		Multi infarct dementia with delusions
363791000006112	Eu01.11	[X]Arteriosclerotic dementia
1972541000006119		Vascular dementia of acute onset, other symptoms, predominantly depressive
1972791000006115		Subcortical vascular dementia, other mixed symptoms
1972821000006112		Mixed cortical and subcortical vascular dementia, without additional symptoms
1973341000006118		Other vascular dementia, other symptoms, predominantly hallucinatory
1973461000006113		Vascular dementia, unspecified, without additional symptoms
1973531000006117		Vascular dementia, unspecified, other symptoms, predominantly hallucinatory
7840641000006111		Ischemic vascular dementia

398571000006112	Eu01300	Mixed cortical and subcortical vascular dementia
295681016	Eu01z00	[X]Vascular dementia, unspecified
1972771000006116		Subcortical vascular dementia, other symptoms, predominantly depressive
2729791000000114		Predominantly cortical vascular dementia
3414261000006114		Multi infarct dementia
1972481000006114		Vascular dementia of acute onset, without additional symptoms
1972711000006113		Subcortical vascular dementia, without additional symptoms
1972731000006119		Subcortical vascular dementia, other symptoms, predominantly delusional
1973221000006118		Other vascular dementia, without additional symptoms
1973271000006117		Other vascular dementia, other symptoms, predominantly delusional
1973401000006112		Other vascular dementia, other mixed symptoms
1235534016		Binswanger's encephalopathy
149347010		Binswanger's disease
1972601000006115		Multi-infarct dementia, without additional symptoms
1972621000006113		Multi-infarct dementia, other symptoms, predominantly delusional
1972641000006118		Multi-infarct dementia, other symptoms, predominantly hallucinatory
1972661000006119		Multi-infarct dementia, other symptoms, predominantly depressive
1972681000006112		Multi-infarct dementia, other mixed symptoms
297013012		Cerebral degeneration due to cerebrovascular disease
3414231000006117		MID - Multi-infarct dementia
3964611000006111		Binswanger's dementia
3964661000006114		Subcortical atherosclerotic dementia
399031000006111		[X]Multi-infarct dementia
12370651000006112		MVAD - Mixed vascular Alzheimer dementia

Lewy-Body Dementia

Medcode	Readcode	Description
745381000006119	F116.00	Lewy body disease
3802621000006119		Lewy body variant of Alzheimer's disease
3195111000006118		Lewy body
8042791000006116		Lewy body dementia with behavioural disturbance
3802641000006114		LBD - Lewy body disease
3802661000006113		DLBD - Diffuse Lewy body disease
6024061000006111		Senile dementia of the Lewy body type
299641000000112	Eu02500	Diffuse Lewy body disease
3802651000006111		Dementia of the Lewy body type
3195101000006116		Lewy bodies
8042801000006115		Lewy body dementia with behavioral disturbance
3802631000006116		SDLT - Senile dementia of the Lewy body type
914931000006119		[D] Dementia with Lewy bodies
3189531000006118		Diffuse Lewy body disease with spongiform cortical change
3802681000006115		CLBD - Cortical Lewy body disease
3802671000006118		Cortical Lewy body disease
295688010		[x]dementia in parkinson's disease

Appendix: codelists used in the study

Frontotemporal Dementia

Medcode	Readcode	Description
22408016	F111.00	Pick's disease
2707661000006119		Pick disease
2707671000006114		Picks disease
295685013	Eu02000	Dementia due to Pick's disease
5005601000006112		Frontotemporal dementia
5005611000006110		Pick's disease with Pick bodies
5005631000006116		Pick's disease with Pick cells and no Pick bodies
5005641000006114		Pick disease with Pick cells and no Pick bodies
7559631000006110		Frontotemporal dementia with gene located on 3p11
7560531000006110		Frontotemporal dementia with parkinsonism-17
7560541000006117		Familial Pick's disease
7560571000006113		FTDP-17 - Frontotemporal dementia with parkinsonism 17
7750421000006118		Right temporal atrophy variant frontotemporal dementia
7755121000006115		Behavioural variant of frontotemporal dementia
8009521000006114		Dementia due to Picks disease
8009531000006112		Dementia due to Pick disease
8009541000006119		Dementia co-occurrent and due to Pick's disease
8024631000006118		Altered behavior co-occurrent and due to Pick's disease

Unspecified dementia

Medcode	Readcode	Description
359151000006113		"[x] senile dementia depressed or paranoid type"
1972081000006115		"unspecified dementia other mixed symptoms”
1972041000006114		"unspecified dementia other symptoms predominantly delusional”
1972071000006118		"unspecified dementia other symptoms predominantly depressive”
1972061000006113		"unspecified dementia other symptoms predominantly hallucinatory”
1972021000006119		"unspecified dementia without additional symptoms”
914941000006112		[d] dementia
939491000006118		[rfc] dementia
359081000006118		[x] presenile dementia nos
359101000006114		[x] primary degenerative dementia nos
359141000006111		[x] senile dementia nos
359241000006119		[x] unspecified dementia
12702281000006111		degenerative brain disorder
882171000006115		dementia
1823871000006112		dementia confirmed
1949621000006118		dementia stage at diagnosis
1949631000006115		dementia stage at diagnosis - early (mild)
1949651000006110		dementia stage at diagnosis - late (severe)
1949641000006113		dementia stage at diagnosis - mid (moderate)
1949671000006117		dementia stage at diagnosis - not known
1949661000006112		dementia stage at diagnosis - undetermined
251625013		h/o: dementia
4539871000006116		history of dementia
3350441000006115		organic dementia
882181000006117		other senile/presenile dement.

21256010		presenile dementia
294643015		presenile dementia nos
294638010		presenile dementia with delirium
294642013		presenile dementia with depression
294641018		presenile dementia with paranoia
2748441000006111		sd - senile dementia
882191000006119		senile and presenile dementias
26545010		senile dementia
882201000006116		senile dementia - simple type
294648012		senile dementia with delirium
294646011		senile dementia with depression
294644014		senile dementia with depressive or paranoid features
294647019		senile dementia with depressive or paranoid features nos
294645010		senile dementia with paranoia
882211000006118		senile dementia-acute confused
294660013		senile or presenile psychoses nos
148381000006115		senile/presenile dementia
294637017		uncomplicated presenile dementia
294635013		uncomplicated senile dementia

Other

Medcode	Readcode	Description
2366171000000112		sporadic creutzfeldt-jakob disease
8024201000006111		mixed dementia*
295684012		[x]dementia in other diseases classified elsewhere
294718018		dementia in conditions ec
346929012		alcoholic dementia nos
362941000006113		[x]alcoholic dementia nos
2502971000006115		dementia associated with alcoholism
7043651000006119		dementia associated with parkinson's disease
295687017		[x]dementia in huntington's disease
481281019		progressive multifocal leucoencephalopathy
295690011		[x]dementia in other specified diseases classif elsewhere
376571000006116		[x]dementia in human immunodef virus [hiv] disease
295688010		[x]dementia in parkinson's disease
2510951000006114		creutzfeldt-jakob disease
2386018		jakob-creutzfeldt disease
2366181000000114		sporadic cjd (creutzfeldt-jakob disease)
423221000006117		[x]predominantly cortical dementia
97154016		niemann-pick disease
295686014		[x]dementia in creutzfeldt-jakob disease
359091000006115		[x] presenile dementia nos
2510981000006118		cjd - creutzfeldt-jakob disease
2502981000006117		alcohol-induced persisting dementia
3964591000006117		subcortical leucoencephalopathy
6973421000006116		dementia associated with aids
7263021000006116		dementia due to huntingtons disease

*this code was classe as other, as our mixed subtype codes must have specified either AD/LBD or non AD/LBD - as no subtype is specified it is technically an unspecified code.

Covariates

Atrial Fibrillation

**Medcode**	**Readcode**	**Description**
300130013	G573.00	Atrial fibrillation and flutter
300132017	G573z00	Atrial fibrillation and flutter NOS
3299911000006116		AF - Atrial fibrillation
421235014	G573200	Paroxysmal atrial fibrillation
5669591000006113		AF - Paroxysmal atrial fibrillation
636701000000115	G573500	Persistent atrial fibrillation
636721000000112	G573400	Permanent atrial fibrillation
82343012	G573000	Atrial fibrillation
9988012	G573100	Atrial flutter

Alcohol addiction/problematic alcohol use

medcode	readcode	desc
106878015	8BA8.00	Alcohol detoxification
110629012	E231.00	Chronic alcoholism
11911671000006113		[V]Personal history of alcoholism
11912461000006111		[V]Problems related to lifestyle - alcohol use
11922951000006112		Alcoholic psychoses
12031691000006112		Alcoholic steatohepatitis
12054431000006113		Chronic pancreatitis due to acute alcohol intoxication
12456651000006117		[V]Alcohol rehabilitation
12721711000006110		Alcoholic liver damage unspecified
12730491000006117		Other alcoholic psychosis
12878014	E23..11	Alcoholism
138527014	G555.00	Alcoholic cardiomyopathy
14103017	F375.00	Alcohol-induced polyneuropathy
15243013	E010.00	Alcohol withdrawal delirium
1894171000006111		Alcohol dependence resolved
1972161000006112		Mental & behav dis due to use alcohol: acute intoxication, with trauma or other bodily injury
1973701000006119		Mental and behav dis due to use alcohol: dependence syndr, currently abstinent
1973811000006113		Mental and behav dis due to use alcohol: dependence syndr, currently abstinent, but in a protected environment
1973871000006116		Mental and behav dis due to use alcohol: dependence syndr, currently on a clinically supervised maintenance or replacement regime [controlled dependence]
1974021000006112		Mental and behav dis due to use alcohol: dependence syndr, currently abstinent, but receiving treatment with aversive or blocking drugs
1974071000006113		Mental and behav dis due to use alcohol: dependence syndr, currently using the substance [active dependence]
1974111000006117		Mental and behav dis due to use alcohol: dependence syndr, continuous use

1974141000006118		Mental and behav dis due to use alcohol: dependence syndr, episodic use [dipsomania]
1974191000006110		Mental and behav dis due to use alcohol: withdrawal state, uncomplicated
1974231000006117		Mental and behav dis due to use alcohol: withdrawal state, with convulsions
1974301000006117		Mental & behav dis due to use alcohol: withdrawl state with delirium, without convulsions
1974521000006117		Mental & behav dis due to use alcohol: withdrawl state with delirium, with convulsions
1975141000006117		Mental & behav dis due to use alcohol: psychotic disorder, schizophrenia-like
1975181000006111		Mental & behav dis due to use alcohol: psychotic disorder, predominantly delusional
1975291000006117		Mental & behav dis due to use alcohol: psychotic disorder, predominantly hallucinatory
1975341000006119		Mental & behav dis due to use alcohol: psychotic disorder, predominantly polymorphic
1975371000006110		Mental & behav dis due to use alcohol: psychotic disorder, predominantly depressive symptoms
1975411000006111		Mental & behav dis due to use alcohol: psychotic disorder, predominantly manic symptoms
1975461000006114		Mental & behav dis due to use alcohol: psychotic disorder, mixed
1975511000006112		Mental & behav dis due to use alcohol: resid & late-onset psychot dis, flashbacks
1975541000006111		Mental & behav dis due to use alcohol: resid & late-onset psychot dis, personality or behaviour disorder
1975591000006119		Mental & behav dis due to use alcohol: resid & late-onset psychot dis, dementia
1975661000006111		Mental & behav dis due to use alcohol: resid & late-onset psychot dis, residual affective disorder
1976071000006119		Mental & behav dis due to use alcohol: resid & late-onset psychot dis, other persisting cognitive impairment
1976111000006110		Mental & behav dis due to use alcohol: resid & late-onset psychot dis, late-onset psychotic disorder
2358251000000115	2126C00	Alcohol dependence resolved
2467301000000111	8BAs.00	Alcohol relapse prevention
2474678017	66e..00	Alcohol disorder monitoring
2485391000000114	8BAw.00	Alcohol twelve step programme
2502971000006115		Dementia associated with alcoholism
2502981000006117		Alcohol-induced persisting dementia
251626014	1462.00	H/O: alcoholism
2549849017	1B1c.00	Alcohol induced hallucinations
2612481000006116		Alcohol hallucinosis
2612491000006118		Alcohol-induced psychotic disorder with hallucinations
2612511000006112		Alcohol induced hallucinosis
2614651000006115		Alcoholism
2626141000006112		Alcoholic polyneuropathy
2626161000006111		Alcoholic peripheral neuropathy
263572010	63C7.00	Maternal alcohol abuse
2658601000006116		Acute alcoholic liver disease
2724201000000113		Late onset alcohol-induced psychosis
2740361000006119		AA - Alcohol abuse
2740831000000114		Harmful use of alcohol
2806371000006116		Alcoholic myopathic syndrome
2806381000006118		Alcoholic myositis

2819381000006112		Alcohol rehabilitation and detoxification
283559019	8H35.00	Admitted to alcohol detoxification centre
2885451000006111		Alcoholism counseling
2911271000006113		Acute alcoholism
294662017	E011100	Korsakov's alcoholic psychosis with peripheral neuritis
294664016	E011z00	Alcohol amnestic syndrome NOS
294668018	E012000	Chronic alcoholic brain syndrome
294669014	E013.00	Alcohol withdrawal hallucinosis
294671014	E014.00	Pathological alcohol intoxication
294672019	E015.00	Alcoholic paranoia
294673012	E01y.00	Other alcoholic psychosis
294674018	E01y000	Alcohol withdrawal syndrome
294675017	E01yz00	Other alcoholic psychosis NOS
294676016	E01z.00	Alcoholic psychosis NOS
295126010	E230.11	Alcohol dependence with acute alcoholic intoxication
295127018	E230.00	Acute alcoholic intoxication in alcoholism
295128011	E230000	Acute alcoholic intoxication, unspecified, in alcoholism
295129015	E230100	Continuous acute alcoholic intoxication in alcoholism
295130013	E230200	Episodic acute alcoholic intoxication in alcoholism
295131012	E230300	Acute alcoholic intoxication in remission, in alcoholism
295132017	E230z00	Acute alcoholic intoxication in alcoholism NOS
295136019	E231000	Alcohol dependence
295139014	E231100	Continuous chronic alcoholism
295141010	E231200	Episodic chronic alcoholism
295142015	E231300	Chronic alcoholism in remission
295143013	E231z00	Chronic alcoholism NOS
295144019	E23z.00	Alcohol dependence syndrome NOS
295251015	E250000	Nondependent alcohol abuse, unspecified
295252010	E250100	Nondependent alcohol abuse, continuous
295253017	E250200	Nondependent alcohol abuse, episodic
295254011	E250300	Nondependent alcohol abuse in remission
295255012	E250z00	Nondependent alcohol abuse NOS
295765017	Eu10.00	Alcohol-induced organic mental disorder
2969641000006116		Alcohol-related disorder
297011014	F11x011	Alcoholic encephalopathy
2979071000006110		Alcoholic pellagra
299061000000117	136S.00	Hazardous alcohol use
299081000000114	136T.00	Harmful alcohol use
303399018	J613.00	Alcoholic liver damage
303464018	J61yz00	Other non-alcoholic chronic liver disease NOS
3062791000006112		Alcohol-induced anxiety disorder
3062801000006113		Alcohol induced anxiety disorder
3073921000006112		Alcohol rehabilitation
315091000000117	Eu10800	Alcohol withdrawal-induced seizure
3161691000006118		Alcohol-induced sleep disorder
3161701000006118		Alcohol induced sleep disorder
3165681000006118		Alcoholic liver disease
3165691000006115		ALD - Alcoholic liver disease

3182581000006111		Alcoholic psychosis
3182591000006114		Alcohol induced psychosis
324488015	SM0y.00	Other alcohol causing toxic effect
3315041000006111		Alcoholic steatosis
3315061000006110		Alcoholic fatty liver disease
3374881000006119		Alcohol-induced mood disorder
3374891000006116		Alcohol induced mood disorder
3402361000006113		Alcoholic ketoacidosis
3402371000006118		Alcohol induced ketoacidosis
346929012	E012.11	Alcoholic dementia
3493491000006118		Alcohol-induced psychotic disorder with delusions
3493501000006114		Alcohol induced psychosis with delusions
3527541012		Alcohol abuse surveillance
353587018	J617.00	Alcoholic hepatitis
353594015	J612000	Alcoholic fibrosis and sclerosis of liver
353595019	J613000	Alcoholic hepatic failure
353687012	J670800	Alcohol-induced acute pancreatitis
353698013	J671000	Alcohol-induced chronic pancreatitis
356292013	C150500	Alcohol-induced pseudo-Cushing's syndrome
362921000006118	Eu10211	[X]Alcohol addiction
362941000006113	Eu10711	[X]Alcoholic dementia NOS
362951000006110	Eu10511	Alcoholic hallucinosis
362971000006117	Eu10513	[X]Alcoholic paranoia
362981000006119	Eu10514	[X]Alcoholic psychosis NOS
3630141000006115		Korsakov syndrome - alcoholic
3630161000006116		Amnesic syndrome due to alcohol
3630171000006111		Alcoholic amnestic syndrome
3649911000006110		Alcoholic amblyopia
3688931000006118		Alcohol-induced persisting amnestic disorder
370961000006119	Eu10712	[X]Chronic alcoholic brain syndrome
370971000006114	Eu10212	[X]Chronic alcoholism
3777351000006111		Alcohol-induced sexual dysfunction
3777361000006113		Alcohol induced sexual dysfunction
3794201000006119		Alcohol induced paranoia
3859091000006114		Dilated cardiomyopathy secondary to alcohol
3859111000006117		Dilated cardiomyopathy caused by alcohol
3890681000006118		Uncomplicated alcohol withdrawal
3903681000006118		Non megaloblastic anaemia due to alcoholism
3903691000006115		Non megaloblastic anemia due to alcoholism
3927031000006111		Megaloblastic anaemia due to alcoholism
3927041000006118		Megaloblastic anemia due to alcoholism
3927051000006116		Megaloblastic anaemia caused by alcoholism
3927061000006119		Megaloblastic anemia caused by alcoholism
397621000006111	Eu10500	Alcohol-induced psychosis
397791000006118	Eu10600	Alcohol amnestic disorder
397801000006117	Eu10200	[X]Mental and behav dis due to use alcohol: dependence syndr
397811000006119	Eu10300	[X]Mental and behav dis due to use alcohol: withdrawal state
397881000006114	Eu10100	Alcohol abuse

401760017	E012.00	Other alcoholic dementia
401797010	E250.00	Nondependent alcohol abuse
405461000000114	66e0.00	Alcohol abuse monitoring
451124014	J617000	Chronic alcoholic hepatitis
4525015	J153.00	Alcoholic gastritis
453265010	G852300	Oesophageal varices in alcoholic cirrhosis of the liver
4533451000006113		Excessive alcohol consumption
4533461000006110		Excessive alcohol use
4539891000006115		History of alcoholism
460276013	ZV11300	[V]Personal history of alcoholism
460926018	ZV57A00	[V]Alcohol rehabilitation
461112017	ZV6D600	Alcoholism counselling
4723831000006118		Admitted to alcohol detoxification center
4763141000006111		Korsakov alcoholic psychosis with peripheral neuritis
476361000006114	E011.00	Alcohol amnestic syndrome
476611000006116	J612.00	Alcoholic cirrhosis of liver
476701000006114	F394100	Alcohol myopathy
476741000006111	E01..00	Alcohol-induced psychosis
4768901000006117		Alcohol induced encephalopathy
478024019	F25B.00	Alcohol-induced epilepsy
478046010	F144000	Cerebellar ataxia due to alcoholism
4978041000006118		Alcoholic
4978851000006112		Tolerance to alcohol
4978891000006118		Psychological tolerance to alcohol
4978971000006113		Persistent effect of alcohol
500478011	E23..00	Alcohol dependence syndrome
5012711000006112		Alcoholic coma
502017010	E23..12	Alcohol problem drinking
5063221000006116		Alcohol-related macrocytosis
5063691000006117		Alcohol-related sideroblastic anaemia
5063701000006117		Alcohol-related sideroblastic anemia
5086741000006114		AH - Alcoholic hepatitis
5087751000006113		Chronic pancreatitis due to acute alcohol intoxication
5110231000006116		Alcohol-induced hypoglycaemia
5110241000006114		Alcohol-induced hypoglycemia
5111821000006113		Alcohol-induced pseudo-Cushing syndrome
5111831000006111		Pseudo-Cushing syndrome due to alcohol
542611000006114	F11x000	Cerebral degeneration due to alcoholism
5612351000006115		Alcoholic macrocytosis
5689771000006115		Persistent alcohol abuse
5689781000006117		Chronic alcohol abuse
5886961000006112		Abstinent alcoholic
5887581000006113		Alcohol-induced cerebellar ataxia
5965471000006114		Alcohol injection of liver metastases
5980911000006115		Alcohol-induced flushing
5980921000006111		Alcohol-induced flushes
5981291000006110		Alcohol withdrawal-induced convulsion
5992721000006114		Esophageal varices in alcoholic cirrhosis of the liver

6047161000006116		Alcohol related optic neuropathy
606991000000117	136W.00	Alcohol misuse
6221451000006112		Alcohol-related fit
6221531000006118		Alcoholic cerebellar degeneration
6221541000006111		Alcoholic cerebellar degeneration syndrome
6282271000006115		Tolerance to alcohol - finding
6354581000006114		History of alcohol abuse
6769881000006115		Alcohol abuse control
6769901000006118		Assess alcohol abuse prevention
6769911000006115		Assess alcohol abuse control
6947461000006115		Alcoholic cirrhosis
6947471000006110		Alcoholic liver cirrhosis
7240381000006117		Alcohol abuse cessation behaviour
7240391000006119		Alcohol abuse cessation behavior
7309181000006113		Alcohol-induced pancreatitis
7309191000006111		Inflammation of pancreas due to alcohol
7309201000006114		Alcoholic pancreatitis
7309211000006112		Inflammation of pancreas caused by alcohol
7518451000006115		Thrombocytopaenia due to alcohol
7518461000006118		Thrombocytopenia due to alcohol
7518471000006113		Thrombocytopaenia caused by alcohol
7518481000006111		Thrombocytopenia caused by alcohol
753331000006118	E011000	Korsakov alcoholic psychosis
7613321000006119		Alcohol reduction programme
7613331000006116		Alcohol reduction program
7700651000006119		Chronic alcoholic liver disease
7703061000006115		Acute on chronic alcoholic liver disease
7706231000006114		Mild alcohol dependence
7710581000006113		Severe alcohol dependence
7725141000006111		Moderate alcohol dependence
7791891000006116		Disorder caused by alcohol
7791901000006117		Alcohol induced disorder
7798481000006113		Alcohol relapse prevention programme
7798491000006111		Alcohol relapse prevention program
7798531000006111		Alcohol twelve step program
7821631000006114		Fibrosis of liver caused by alcohol
7821641000006116		Alcoholic fibrosis of liver
7840361000006119		Gastric ulcer caused by alcohol
7840371000006114		Alcohol induced gastric ulcer
7848901000006118		Duodenitis caused by ingestible alcohol
7848911000006115		Alcoholic duodenitis
7849261000006118		Perceptual disturbances and seizures co-occurrent and due to alcohol withdrawal
7849271000006113		Alcohol withdrawal with perceptual disturbances and seizures
7849281000006111		Psychotic disorder caused by alcohol with schizophreniform symptoms
7849291000006114		Alcohol-induced psychotic disorder with schizophreniform symptoms
7849301000006110		Mood disorder with depressive symptoms caused by alcohol
7849311000006113		Alcohol-induced mood disorder with depressive symptoms
7849321000006117		Mood disorder with manic symptoms caused by alcohol

7849331000006119		Alcohol-induced mood disorder with manic symptoms
7849341000006112		Mood disorder with mixed manic and depressive symptoms caused by alcohol
7849351000006114		Alcohol-induced mood disorder with mixed manic and depressive symptoms
7858031000006112		Neurological disorder caused by ingestible alcohol
7858041000006119		Alcohol-related neurological disorder
8016431000006116		Gastric haemorrhage due to alcoholic gastritis
8016441000006114		Gastric hemorrhage due to alcoholic gastritis
8030711000006112		Thrombocytopaenia co-occurrent and due to alcoholism
8030721000006116		Thrombocytopenia co-occurrent and due to alcoholism
8041851000006114		Degenerative brain disorder caused by alcohol
8041861000006111		Degenerative brain disorder due to alcohol
8042671000006110		Circadian rhythm sleep disorder caused by alcohol
8042761000006112		Cerebral degeneration due to alcoholism
8048151000006113		Chronic pancreatitis due to chronic alcoholism
8088731000006112		Disorder due to alcohol abuse
8088741000006119		Alcohol induced disorder co-occurrent and due to alcohol dependence
8088751000006117		Alcohol dependence with alcohol induced disorder
8088761000006115		Perceptual disturbance due to alcohol withdrawal
8121241000006115		Seen by counsellor for alcohol misuse
8220581000006118		Alcohol related harm
8242881000006116		Finding of alcohol drinking risk
8243961000006112		Alcohol rehabilitation and detoxification care management
83834016	J610.00	Alcoholic fatty liver
8458711000006115		Ascites due to alcoholic cirrhosis
8458751000006119		Ascites due to alcoholic hepatitis
8458771000006112		Hepatic coma due to alcoholic liver failure
8462171000006115		Hepatic ascites due to chronic alcoholic hepatitis
852381000006110		Alcohol reduction-maintain abstinence
882221000006114	E01y.99	Other alcoholic psychoses
882531000006110	E250.99	Non-dependent abuse of alcohol
886501000006115	J613.99	Alcoholic liver damage NOS
899431000006116	SM0z.99	Alcohol NOS - toxic effect
9322521000006111		Alcohol dependence in pregnancy
9324921000006113		Alcohol dependence in childbirth
9344021000006112		Unhealthy alcohol drinking behaviour
9344031000006110		Unhealthy alcohol drinking behavior
940781000006116		Evidence of alcohol withdrawal
961571000006111		Excessive use of alcohol
970401000006116		Misuse of mixture of Alcohol
970411000006118		Misuse of Alcohol unspecified
991151000006114	SM0..98	Alcohol NOS - toxic effect

Anxiety disorder

108724018	E260100	Psychogenic torticollis
117876016	E201200	Hysterical deafness
1210253015	E200100	Panic disorder
12127531000006113		Recurrent mild major depressive disorder co-occurrent with anxiety

Appendix: codelists used in the study

12127541000006115		Recurrent severe major depressive disorder co-occurrent with anxiety
12127551000006118		Recurrent moderate major depressive disorder co-occurrent with anxiety
12127561000006116		Recurrent major depressive disorder co-occurrent with anxiety in full remission
12127571000006111		Recurrent major depressive disorder in partial remission co-occurrent with anxiety
1216885017	E206.00	Depersonalisation syndrome
121871000006118	E284.00	Stress reaction causing mixed disturbance of emotion and conduct
1230451012	E292400	Adjustment reaction with anxious mood
1231321013	E202700	Animal phobia
123751014	E290000	Grief reaction
12703831000006115		[X]Organic anxiety disorder
147404016	E201100	Hysterical blindness
147537017	E201400	Hysterical paralysis
1495057015	Eu45300	Somatoform autonomic dysfunction
1495439012	E211.00	Affective personality disorder
158091017	E261500	Psychogenic aphonia
1774451016	E20y000	Somatization disorder
1780506015	388N.00	HAD scale: anxiety score
1861181000006114		Breathlessness causing anxiety
1976491000006113		Mixed anxiety and depressive reaction
199101000006113	E278.00	Psychalgia
199321000006118	E262300	Psychogenic cardiovascular disorder
199341000006113	E262.00	Psychogenic cardiovascular symptoms
199441000006117	E264.00	Somatoform autonomic dysfunction - gastrointestinal tract
199531000006114	E260000	Psychogenic paralysis
199581000006110	E261.00	Psychogenic respiratory symptoms
199671000006111	E265100	Psychologic vaginismus
215891000000116	Eu45000	[X]Somatization disorder
2162219012	E262000	Cardiac neurosis
223631000000113	E202.00	Phobic disorders
223641000000116	E202.12	Phobic anxiety disorder
2287071000000115	173f.00	Anxiety about breathlessness
230091000006118	E21..00	Personality disorder
23211011	E210.00	Paranoid personality disorder
232501000006118	E26..00	Physiological malfunction arising from mental factors
249869011	1288.00	FH: Anxiety state
2500131000006115		Anxiety disorder of childhood OR adolescence
251630012	1466.00	H/O: anxiety state
2525391000006119		Sedative, hypnotic AND/OR anxiolytic-induced anxiety disorder
2593211000006113		Anticipatory anxiety, severe
2674981000006112		Anxiety diarrhea
2675001000006115		Anxiety diarrhoea
2682511000006110		Anticipatory anxiety, mild
269821000006113	E203.00	Obsessive-compulsive disorder
2742251000006114		Hallucinogen-induced anxiety disorder
2742261000006111		Hallucinogen induced anxiety disorder
2777721000006112		Organic anxiety disorder
2832021000006113		Inhalant-induced anxiety disorder
2832031000006111		Inhalant induced anxiety disorder
2848291000006116		Generalized anxiety disorder
2848301000006115		GAD - Generalized anxiety disorder
2848311000006117		GAD - Generalised anxiety disorder
2907991000006110		Social anxiety disorder
294953016	E200000	Anxiety state unspecified
294960010	E200400	Chronic anxiety
294961014	E200500	Recurrent anxiety
294963012	E200z00	Anxiety state
294964018	E201000	Hysteria unspecified

294965017	E201300	Hysterical tremor
294968015	E201500	Hysterical seizures
294978017	E201A00	Dissociative reaction unspecified
294991016	E202000	Phobia
294992011	E202100	Agoraphobia with panic attacks
294993018	E202200	Agoraphobia without history of panic disorder
294994012	E202300	Social phobia, fear of eating in public
294995013	E202400	Social phobia, fear of public speaking
294996014	E202500	Social phobia, fear of public washing
294998010	E202900	Fear of crowds
295004011	E202E00	Fear of pregnancy
295015018	E203000	Compulsive neurosis
295016017	E203100	Obsessional neurosis
295017014	E203z00	Obsessive-compulsive disorder NOS
295026012	E20y.00	Other neurotic disorders
295029017	E20y100	Writer's cramp neurosis
295031014	E20y200	Other occupational neurosis
295032019	E20y300	Psychasthenic neurosis
295033012	E20yz00	Other neurotic disorder NOS
295044016	E211000	Affective personality disorder
295045015	E211100	Hypomanic personality disorder
295048018	E211z00	Affective personality disorder NOS
295342013	E260.00	Psychogenic musculoskeletal symptoms
295346011	E260z00	Psychogenic musculoskeletal symptoms NOS
295348012	E261000	Psychogenic air hunger
295349016	E261100	Psychogenic cough
295350016	E261200	Psychogenic hiccough
295351017	E261300	Psychogenic hyperventilation
295355014	E261400	Psychogenic yawning
295357018	E261z00	Psychogenic respiratory symptom
295360013	E262200	Neurocirculatory asthenia
295362017	E262z00	Psychogenic cardiovascular symptom
295363010	E263.00	Psychogenic skin symptoms
295364016	E263000	Psychogenic pruritus
295366019	E263z00	Psychogenic skin symptoms NOS
295374018	E264200	Cyclical vomiting - psychogenic
295381013	E264400	Psychogenic dyspepsia
295382018	E264500	Psychogenic constipation
295383011	E264z00	Psychogenic gastrointestinal tract symptom NOS
295384017	E265.00	Psychogenic genitourinary tract symptoms
295387012	E265200	Psychogenic dysmenorrhea
295388019	E265300	Psychogenic dysuria
295389010	E265z00	Psychogenic genitourinary tract symptom NOS
295391019	E267.00	Psychogenic symptom of special sense organ
295392014	E26y.00	Other psychogenic malfunction
295393016	E26y000	Bruxism (teeth grinding)
295394010	E26yz00	Other psychogenic malfunction NOS
295395011	E26z.00	Psychosomatic disorder NOS
295460014	E278000	Psychogenic pain unspecified
295464017	E278200	Psychogenic backache
295465016	E278z00	Psychalgia NOS
295474019	E280.00	Acute panic state due to acute stress reaction
295475018	E281.00	Acute fugue state due to acute stress reaction
295476017	E282.00	Acute stupor state due to acute stress reaction
295477014	E283.00	Other acute stress reactions
295478016	E283000	Acute situational disturbance
295483012	E283z00	Other acute stress reaction NOS

295498014	E292100	Adolescent emancipation disorder
295499018	E292200	Early adult emancipation disorder
295504018	E292z00	Adjustment reaction with disturbance of other emotion NOS
295505017	E293000	Adjustment reaction with aggression
295507013	E293100	Adjustment reaction with antisocial behaviour
295508015	E293200	Adjustment reaction with destructiveness
295510018	E29y.00	Other adjustment reactions
295511019	E29y000	Concentration camp syndrome
295512014	E29y100	Other post-traumatic stress disorder
295513016	E29y200	Adjustment reaction with physical symptoms
295517015	E29y500	Other adjustment reaction with withdrawal
295518013	E29yz00	Other adjustment reactions NOS
295519017	E29z.00	Adjustment reaction NOS
296224013	Eu40.00	[X]Phobic anxiety disorders
296236019	Eu40y00	[X]Other phobic anxiety disorders
296237011	Eu40z00	[X]Phobic anxiety disorder, unspecified
296238018	Eu41.00	[X]Other anxiety disorders
296239014	Eu41000	[X]Panic disorder [episodic paroxysmal anxiety]
296245018	Eu41300	[X]Other mixed anxiety disorders
296249012	Eu41z00	[X]Anxiety disorder, unspecified
296251011	Eu42000	[X]Predominantly obsessional thoughts or ruminations
296252016	Eu42100	Compulsion expressed as ritual
296253014	Eu42200	[X]Mixed obsessional thoughts and acts
296254015	Eu42y00	[X]Other obsessive-compulsive disorders
296255019	Eu42z00	[X]Obsessive-compulsive disorder, unspecified
296271019	Eu43y00	[X]Other reactions to severe stress
296272014	Eu43z00	[X]Reaction to severe stress, unspecified
296273016	Eu44.00	[X]Dissociative [conversion] disorders
296277015	Eu44300	[X]Trance and possession disorders
296289018	Eu44700	[X]Mixed dissociative [conversion] disorders
296298015	Eu44z00	Dissociative disorder
296340014	Eu45z00	[X]Somatoform disorder, unspecified
296341013	Eu46.00	[X]Other neurotic disorders
296358015	Eu46z00	Neurosis
296722013	Eu93000	Separation anxiety disorder of childhood
300681000000118	8HHp.00	Referral for guided self-help for anxiety
304838011	E200.99	Anxiety state
3062791000006112		Alcohol-induced anxiety disorder
3062801000006113		Alcohol induced anxiety disorder
3070671000006111		Anticipatory anxiety
30729012	E207.00	Hypochondriasis
3090721000006114		Anticipatory anxiety, moderate
3110021000006119		Anxiety disorder of adolescence
3142901000006117		Cannabis-induced anxiety disorder
3142911000006119		Cannabis induced anxiety disorder
3265831000006118		Adjustment disorder with anxiety
3287741000006111		Anxiety
3287771000006115		Anxiety reaction
3334381000006111		Cocaine-induced anxiety disorder
3334391000006114		Cocaine induced anxiety disorder
33475016	E202800	Claustrophobia
3357961000006113		Anxiety disorder due to a general medical condition
3357971000006118		Anxiety disorder due to medical disorder
3357981000006115		Anxiety disorder due to general medical condition
3367191000006114		Anxiety disorder of childhood
3409261000006113		Phencyclidine-induced anxiety disorder
3409271000006118		PCP induced anxiety disorder

342665019	8G94.00	Anxiety management training
345691000000118	Eu45500	[X]Globus pharyngeus
3497371000006119		Moderate anxiety
3514292010		Anxiety in pregnancy
3527472014		Anxiety disorder caused by synthetic cannabinoid
362441000006118	Eu43000	[X]Acute stress reaction
362691000006113	Eu43200	Adjustment disorder
362871000006111	Eu40000	Agoraphobia
3630001000006116		Anxiety hyperventilation
363641000006114	Eu41y11	[X]Anxiety hysteria
3636421019		Anxiety disorder caused by stimulant
363651000006111	Eu41111	Anxiety neurosis
363661000006113	Eu41z11	[X]Anxiety NOS
363671000006118	Eu41112	[X]Anxiety reaction
363681000006115	Eu41113	[X]Anxiety state
3636814011		Anxiety disorder caused by synthetic cathinone
3649131000006119		Caffeine-induced anxiety disorder
3649141000006112		Caffeine induced anxiety disorder
3654681000006114		Mild anxiety
370048011	E202A00	Fear of flying
376681000006119	Eu46100	Depersonalisation-derealisation syndrome
378101000006119	Eu44000	[X]Dissociative amnesia
378121000006112	Eu44500	Dissociative convulsions
378131000006110	Eu44100	Dissociative fugue
378141000006117	Eu44400	Dissociative motor disorder
378151000006115	Eu44200	[X]Dissociative stupor
378291000006117	Eu51511	[X]Dream anxiety disorder
3810661000006112		Severe anxiety (panic)
3839481000006118		Amphetamine-induced anxiety disorder
3839491000006115		Amphetamine induced anxiety disorder
388071000006116	Eu41100	[X]Generalized anxiety disorder
3883581000006112		Separation anxiety disorder of childhood, early onset
389371000006113	Eu45200	[X]Hypochondriacal disorder
3950381000006113		Anxiety about blushing
398351000006110	Eu41211	[X]Mild anxiety depression
398561000006117	Eu41200	[X]Mixed anxiety and depressive disorder
399841000006110	Eu46000	[X]Neurasthenia
399981000006111	Eu4..00	[X]Neurotic, stress-related and somatoform disorders
400321000006113	Eu42.00	[X]Obsessive - compulsive disorder
401778010	E201600	Conversion disorder
401779019	E201z00	Hysteria NOS
401780016	E202z00	Phobic disorder
401783019	E20z.00	Neurotic disorder NOS
401804015	E264300	Psychogenic diarrhoea
401810015	E28z.00	Acute stress reaction NOS
401881014	Eu41y00	[X]Other specified anxiety disorders
401885017	Eu44y00	[X]Other dissociative [conversion] disorders
401886016	Eu45400	[X]Persistent somatoform pain disorder
401887013	Eu45y00	[X]Other somatoform disorders
401888015	Eu46y00	[X]Other specified neurotic disorders
403931000006116	Eu05400	Organic anxiety disorder
4196231000006110		Dream anxiety disorder
419841000006116	Eu34114	[X]Persistant anxiety depression
420641000006116	Eu93100	[X]Phobic anxiety disorder of childhood
423021000006114	Eu43100	Post-traumatic stress disorder
424471000006119	Eu43.00	Stress and adjustment reaction
427051000006113	Eu93200	Childhood social anxiety disorder

427071000006115	Eu40100	Social phobia
427131000006117	Eu45.00	Somatoform disorder
427231000006111	Eu40200	Specific phobia
429711000006118	Eu45100	[X]Undifferentiated somatoform disorder
4391041000006110		Separation anxiety
441512015	E292000	Separation anxiety disorder
4432121000006110		Death anxiety
4539991000006111		History of anxiety state
459711000006119	E283100	Acute post-trauma stress state
461701000006114	E29y400	Adjustment reaction due to hospitalisation
461761000006110	E294.00	Adjustment reaction with mixed disturbance of emotion and conduct
461801000006118	E293z00	Adjustment reaction with predominant disturbance conduct NOS
461821000006111	E292.00	Adjustment reaction, predominant disturbance other emotions
4767501000006113		Childhood phobic anxiety disorder
478089014	E29..00	Adjustment reaction
481154010	E200200	Generalised anxiety disorder
484681013	E264000	Psychogenic aerophagy
485870016	E202B00	Cancer phobia
488201000006114	E200.00	Anxiety disorder
488211000006112	E200300	Mixed anxiety and depressive disorder
493878012	E201.00	Hysteria
4944401000006116		Anxiety about treatment
4944411000006118		Anxiety about forced dependence
4944421000006114		Anxiety about loss of control
4944431000006112		Anxiety about resuming sexual relations
4944481000006113		Anxiety about not coping with parenthood
4944501000006115		Anxiety about making mistakes
4944511000006117		Anxiety about getting it wrong
4944521000006113		Anxiety about altered body image
4949001000006110		Anxiety reduction
497343017	E292y00	Adjustment reaction with mixed disturbance of emotion
500650019	E28..00	Acute stress reaction
5024071000006118		Anxiety depression
5024101000006111		Anxiety hysteria
506324018	E293.00	Adjustment reaction with predominant disturbance of conduct
5248991000006117		Anxiety and fear
5249061000006116		Anxiety about body function or health
5249071000006111		Anxiety about health
5249261000006112		Anxiety about behaviour or performance
5249271000006117		Anxiety about social functioning
5249281000006119		Anxiety about behavior or performance
5549571000006113		Depression anxiety scale
5605191000006116		Anxiety about losing control of bowels
5605201000006118		Anxiety about wetting self
5605211000006115		Anxiety about vomiting in public
5605221000006111		Anxiety about having a fit
5605231000006114		Anxiety about choking
5605241000006116		Anxiety about swallowing
5605251000006119		Anxiety about collapsing
5605261000006117		Anxiety about shaking
5605271000006112		Anxiety about sweating
5605281000006110		Anxiety about dying
5605291000006113		Anxiety about going crazy
5605301000006114		Anxiety about going insane
5605311000006112		Anxiety about going mad
5605321000006116		Anxiety about losing emotional control
5605341000006111		Anxiety about becoming fat

5605351000006113		Anxiety about obesity
5605371000006115		Anxiety about fainting
5605381000006117		Anxiety about having a heart attack
5605411000006119		Anxiety about appearing ridiculous
5605421000006110		Anxiety about saying the wrong thing
56251019	E201B00	Compensation neurosis
5628441000006118		Performance anxiety
5886461000006119		Parental anxiety
5886481000006112		Anxiety attack
5935111000006118		Castration anxiety complex
6025541000006118		Counseling for anxiety
605901000006114	E211300	Cyclothymic personality disorder
613781000006118	E211200	Depressive personality disorder
626101000006114	E2D0z00	Disturbance anxiety and fearfulness childhood/adolescent NOS
626121000006116	E2D0.00	Disturbance of anxiety and fearfulness in childhood and adolescence
6358151000006115		Episodic paroxysmal anxiety disorder
65032014	E202C00	Dental phobia
6707631000006112		Anxiety level
6707891000006110		Self-control behavior: anxiety
675881000006115	E20..00	Neurotic disorder
675921000006111	E2...00	Neurotic, personality and other nonpsychotic disorders
677321000006115	E205.00	Neurasthenia
694811000006118	E201900	Multiple personality
7046013	E201800	Hysterical fugue
7137751000006110		Anxiety about loss of memory
7567751000006111		Mood or anxiety disorder clinic
7859081000006118		Anxiety disorder caused by opioid
7859091000006115		Opioid-induced anxiety disorder
7860011000006112		Anxiety disorder caused by methylenedioxymethamphetamine
7860021000006116		Anxiety disorder caused by MDMA (methylenedioxymethamphetamine)
7860031000006118		Ecstasy type drug-induced anxiety disorder
7860271000006114		Anxiety disorder caused by dissociative drug
7860291000006110		Anxiety disorder caused by ketamine
7860301000006111		Ketamine-induced anxiety disorder
787521000006112	E201700	Hysterical amnesia
8010321000006110		Anxiety disorder caused by drug
8103671000006118		Anxiety about lethargy
8103681000006115		Anxiety about mood
8113001000006117		Stranger anxiety
8297491000006114		Education about anxiety
853241000006119		Phobic anxiety
9322671000006111		Anxiety disorder in mother complicating childbirth
9322681000006114		Anxiety in childbirth
97986013	E202600	Acrophobia
980191000006111	Eu40300	Needle phobia
9901121000006113		Illness anxiety disorder
9901161000006119		Mild major depressive disorder co-occurrent with anxiety single episode
9901261000006114		Moderate major depressive disorder co-occurrent with anxiety single episode
9901281000006116		Severe major depressive disorder co-occurrent with anxiety single episode

Asthma

medcode	readcode	desc
104872017	H330000	Extrinsic asthma without status asthmaticus
1139131000000117	9NNX.00	Under care of asthma specialist nurse

1190365100000611 6		Life threatening acute exacerbation of asthma
1190385100000611 5		Severe asthma attack
1192285100000611 5		Acute asthma
1192467100000611 7		Extrinsic asthma with status asthmaticus
1192637100000611 6		Intrinsic asthma with status asthmaticus
1208954011	663t.00	Asthma causes daytime symptoms 1 to 2 times per month
1208955012	663u.00	Asthma causes daytime symptoms 1 to 2 times per week
1208956013	663v.00	Asthma causes daytime symptoms most days
1208957016	663r.00	Asthma causes night symptoms 1 to 2 times per month
1208958014	663x.00	Asthma limits walking on the flat
1208959018	663w.00	Asthma limits walking up hills or stairs
1208960011	663s.00	Asthma never causes daytime symptoms
1208969012	663V100	Mild asthma
1208970013	663V200	Moderate asthma
1208971012	663V000	Occasional asthma
1208972017	663V300	Severe asthma
1212342016	663q.00	Asthma daytime symptoms
1248506100000611 2		Emergency admission, asthma
1261834100000611 6		Severe asthma with fungal sensitisation
1262360100000611 1		Excepted from asthma quality indicators - service unavailable
137491000000115	9hA2.00	Excepted from asthma quality indicators - informed dissent
139051000000111	9hA..00	Exception reporting - asthma quality indicators
145961000006117	H33z011	Acute severe exacerbation of asthma
1483199016	H330.11	Allergic asthma
1488668018	1J70.00	Suspected asthma
1488722011	66YP.00	Asthma night-time symptoms
1495417010	G581.11	Cardiac asthma
149741000006116	H35y600	Sequoiosis (red-cedar asthma)
151338014	H330100	Life threatening acute exacerbation of allergic asthma
157901000000115	9hA1.00	Excepted from asthma quality indicators - patient unsuitable
1722421000000110	66Yq.00	Asthma causes night time symptoms 1 to 2 times per week
1722501000000119	66Yr.00	Asthma causes symptoms most nights
1722581000000112	663P000	Asthma limits activities 1 to 2 times per month
1722651000000119	663P100	Asthma limits activities 1 to 2 times per week
1722731000000116	663P200	Asthma limits activities most days
1768981000000110	66Ys.00	Asthma never causes night symptoms
1780388018	1O2..00	Asthma confirmed
1807891000006111		Asthma causes daytime asthma symptoms less than weekly
1807901000006110		Asthma causes daytime symptoms more than weekly, less than daily
1807911000006113		Asthma causes daytime asthma symptoms daily
1807921000006117		Asthma causes night time symptoms less than 2 times per month
1807931000006119		Asthma causes night time symp more than 2 times a month,not wkly
1807941000006112		Asthma causes night time asthma symptoms weekly or more often

1821501000006116		Frequent night time asthma symptoms
1821511000006118		Infrequent asthma exacerbations
1821521000006114		Occasional asthma exacerbations
1821531000006112		Frequent asthma exacerbations
1859261000006117		Date of asthma diagnosis
1948051000006112	H3B..00	Asthma-chronic obstructive pulmonary disease overlap syndrome
2009981000006110		Difficult asthma
2010031000006116		Acute infective exacerbation of asthma
2010041000006114		Acute non-infective exacerbation of asthma
2011441000006114		OH respiratory questionnaire: history of asthma
21390015	H331000	Intrinsic asthma without status asthmaticus
2240591000000119	H335.00	Chronic asthma with fixed airflow obstruction
2460351000000111	38B8.00	Severe asthma exacerbation risk assessment
251716013	14B4.00	H/O: asthma
264540018	663N.00	Asthma disturbing sleep
264541019	663N000	Asthma causing night waking
264542014	663N100	Asthma disturbs sleep weekly
264543016	663N200	Asthma disturbs sleep frequently
264544010	663O.00	Asthma not disturbing sleep
264545011	663O000	Asthma never disturbs sleep
264546012	663P.00	Asthma limiting activities
264547015	663Q.00	Asthma not limiting activities
264566015	663e.00	Asthma restricts exercise
264569010	663f.00	Asthma never restricts exercise
2685251000006117		Millers' asthma
2685261000006115		Mill-workers' asthma
2685281000006113		Grain worker's asthma
2690161000006118		Feather asthma
2708621000006112		Stripper's asthma
2786201000006115		Printers' asthma
2815001000006115		Meat-wrappers' asthma
285727014	9OJ1.00	Attends asthma monitoring
2973091000006113		Miners' asthma
2988741000006118		Allergic-infective asthma
3005801000006117		EIA - Exercise-induced asthma
3005811000006119		Exercise induced asthma
301450011	H312000	Chronic asthmatic bronchitis
301480018	H33..11	Bronchial asthma
301485011	H33..00	Asthma
301499010	H330z00	Extrinsic asthma
301508013	H331z00	Intrinsic asthma NOS
301509017	H332.00	Mixed asthma
301511014	H33z.00	Asthma unspecified
3047731000006119		Bakers' asthma
3047741000006112		Flour asthma
3047751000006114		Baker's asthma
3176931000006111		Asthmatic pulmonary alveolitis
338238011	H334.00	Brittle asthma

3402341000006114		Asthma without status asthmaticus
3425341000006113		Wood asthma
3425351000006110		Wood-workers' asthma
3425371000006117		Wood dust asthma
3425381000006119		Saw dust asthma
3435971000006119		Industrial asthma
3494911000006116		Silo-workers' asthma
350147016	H330.12	Childhood asthma
350148014	H331.11	Late onset asthma
350149018	H33z200	Late-onset asthma
350151019	H330111	Acute exacerbation of allergic asthma
350152014	H33zz12	Allergic asthma NEC
350153016	H330011	Hay fever with asthma
350154010	H330.14	Pollen asthma
350156012	H331111	Acute exacerbation of intrinsic asthma
3511374015		Moderate acute exacerbation of asthma
3514315010		Asthma in pregnancy
3514925011		Acute severe exacerbation of asthma co-occurrent and due to allergic asthma
3514926012		Allergic asthma with status asthmaticus
3514928013		Uncomplicated allergic asthma
3514930010		Uncomplicated non-allergic asthma
3637387011		Exacerbation of allergic asthma
3669421000006118		Asthma - cardiac
3894091000006115		Cotton-dust asthma
396114013	H331.00	Intrinsic asthma
396118011	H33z000	Life threatening acute exacerbation of asthma
396119015	H33z100	Asthma attack
396120014	H33zz00	Asthma NOS
4009591000006110		Chemical-induced asthma
4020511000006119		Drug-induced asthma
405054019	9OJA.11	Asthma monitored
409865018	H33z111	Asthma attack NOS
419211018	H333.00	Acute exacerbation of asthma
4540771000006118		History of asthma
456163018	663j.00	Asthma - currently active
456164012	663h.00	Asthma - currently dormant
4781531000006113		Asthmatic
5054341000006110		Late-onset asthma
5054371000006119		Colophony asthma
5054381000006116		Sulphite-induced asthma
5054391000006118		Sulfite-induced asthma
5054431000006112		Asthmatic pulmonary eosinophilia
5492441000006114		Non-allergic asthma
5492451000006111		Asthma due to internal immunological process
5649191000006110		Exacerbation of asthma
6298421000006116		Eosinophilic asthma
6512381000006116		Atopic asthma
6514741000006118		Asthma finding

6550131000006111		Nocturnal asthma
655601000006113	173A.00	Exercise-induced asthma
655611000006111	H33zz11	Exercise induced asthma
660351000006118	H330.00	Allergic atopic asthma
6703821000006115		Platinum asthma
6703841000006110		Cheese-makers' asthma
6703881000006116		Isocyanate induced asthma
6720721000006111		Asthmatic bronchitis
6782071000006115		Cough variant asthma
6920631000006113		Tea-makers' asthma
69311016	H47y000	Detergent asthma
7015991000006110		Non-IgE mediated allergic asthma
7016001000006111		Non IgE mediated allergic asthma
7016011000006114		Non-immunoglobulin E mediated allergic asthma
7021801000006116		Substance induced asthma
7030311000006117		IgE-mediated allergic asthma
7030321000006113		IgE mediated asthma
7030341000006118		IgE mediated allergic asthma
7030351000006116		Immunoglobulin E-mediated allergic asthma
7052221000006118		Exacerbation of intermittent asthma
7062421000006112		Severe persistent asthma
7067211000006115		Mild persistent asthma
7071891000006112		Moderate persistent asthma
7076331000006112		Intermittent asthma
7077481000006119		Mild intermittent asthma
7100601000006111		History of aspirin-sensitive asthma with nasal polyp
7100611000006114		History of aspirin sensitive asthma with nasal polyp
7258581000006114		Acute exacerbation of chronic asthmatic bronchitis
7307941000006113		Seasonal asthma
7584511000006110		Allergic asthma due to Dermatophagoides pteronyssinus
7584521000006119		Dermatophagoides pteronyssinus allergic asthma
7584531000006116		Allergic asthma caused by Dermatophagoides pteronyssinus
7584541000006114		Allergic asthma due to Dermatophagoides farinae
7584551000006111		Dermatophagoides farinae allergic asthma
7584561000006113		Allergic asthma caused by Dermatophagoides farinae
7617601000006114		Uncomplicated asthma
7617611000006112		Exacerbation of mild persistent asthma
7617621000006116		Exacerbation of moderate persistent asthma
7617631000006118		Exacerbation of severe persistent asthma
7618791000006110		Uncomplicated mild persistent asthma
7618801000006111		Uncomplicated moderate persistent asthma
7618811000006114		Uncomplicated severe persistent asthma
7626521000006110		Acute severe exacerbation of severe persistent asthma
7626531000006113		Acute severe exacerbation of moderate persistent asthma
7626541000006115		Acute severe exacerbation of mild persistent asthma
7627241000006119		Acute exacerbation of asthma
7628191000006118		Acute severe exacerbation of asthma
7628271000006114		Acute exacerbation of extrinsic asthma

7628281000006112		Acute exacerbation of immunoglobulin E-mediated allergic asthma
7628301000006111		Acute severe exacerbation of allergic asthma
7628311000006114		Acute severe exacerbation of extrinsic asthma
7628321000006118		Acute severe exacerbation of immunoglobin E-mediated allergic asthma
7628331000006115		Acute severe exacerbation of intrinsic asthma
7961271000006111		Acute severe refractory exacerbation of asthma
7961281000006114		Acute severe asthma
7961291000006112		Status asthmaticus
7965491000006114		Asthma with irreversible airway obstruction
7966291000006118		Intermittent asthma well controlled
7970131000006117		Intermittent asthma uncontrolled
7970171000006119		Acute exacerbation of chronic obstructive airways disease with asthma
8031201000006110		Acute exacerbation of asthma co-occurrent with allergic rhinitis
8038931000006119		Severe persistent asthma co-occurrent with allergic rhinitis
8038941000006112		Moderate persistent asthma co-occurrent with allergic rhinitis
8038961000006111		Mild persistent asthma co-occurrent with allergic rhinitis
8038971000006116		Intermittent asthma co-occurrent with allergic rhinitis
8042571000006111		Acute exacerbation of moderate persistent asthma
8042581000006114		Acute exacerbation of mild persistent asthma
817361000006114	H330.13	Hay fever with asthma
8437441000006111		Moderate acute exacerbation of asthma
8465371000006113		Life threatening acute exacerbation of allergic asthma
8465381000006111		Life threatening acute exacerbation of extrinsic asthma
8465391000006114		Life threatening acute exacerbation of intrinsic asthma
8465401000006111		Life threatening acute exacerbation of non-allergic asthma
8555681000006115		Steroid dependent asthma
856031000006110		Asthma stable < 3 months
856041000006117		Asthma stable > 3 months
885291000006115	H330.99	Extrinsic asthma - atopy
905331000006118		[RFC] Asthma
908841000006111		[RFC] Asthma
929091000006111	1780.00	Aspirin-induced asthma
9314961000006112		Acute severe exacerbation of asthma co-occurrent with allergic rhinitis
9315071000006117		Severe controlled persistent asthma
9315091000006116		Severe persistent allergic asthma
9315101000006110		Acute severe exacerbation of severe persistent allergic asthma
9315111000006113		Acute severe exacerbation of severe persistent asthma co-occurrent with allergic rhinitis
9315121000006117		Severe persistent allergic asthma controlled
9315141000006112		Severe persistent asthma controlled co-occurrent with allergic rhinitis
9315151000006114		Severe persistent allergic asthma uncontrolled
9315171000006116		Severe persistent asthma uncontrolled co-occurrent with allergic rhinitis
9315181000006118		Severe uncontrolled persistent asthma
9315201000006117		Mild persistent allergic asthma
9315221000006110		Acute severe exacerbation of mild persistent allergic asthma
9315231000006113		Acute severe exacerbation of mild persistent allergic asthma co-occurrent with allergic rhinitis
9315251000006118		Mild persistent allergic asthma controlled
9315261000006116		Mild persistent asthma controlled
9315281000006114		Mild persistent asthma controlled co-occurrent with allergic rhinitis

9315291000006112		Mild persistent allergic asthma uncontrolled
9315301000006113		Mild persistent asthma uncontrolled
9315321000006115		Mild persistent asthma uncontrolled co-occurrent with allergic rhinitis
9315341000006110		Moderate persistent asthma controlled
9315351000006112		Moderate persistent allergic asthma
9315371000006119		Acute severe exacerbation of moderate persistent allergic asthma
9315391000006118		Acute severe exacerbation of moderate persistent asthma co-occurrent with allergic rhinitis
9315401000006116		Moderate persistent allergic asthma controlled
9315411000006118		Moderate persistent controlled asthma co-occurrent with allergic rhinitis
9315431000006112		Moderate persistent allergic asthma uncontrolled
9315441000006119		Moderate persistent asthma uncontrolled co-occurrent with allergic rhinitis
9315461000006115		Moderate persistent asthma uncontrolled
9317311000006119		Chronic obstructive asthma co-occurrent with acute exacerbation of asthma
9317331000006113		Asthma-COPD overlap syndrome (ACOS)
9317341000006115		ACOS - asthma-chronic obstructive pulmonary disease overlap syndrome
9322561000006117		Asthma in mother complicating childbirth
9322571000006112		Asthma in childbirth
94731013	H35y700	Wood asthma
955631000006116		Occupational asthma
95786019	173c.00	Occupational asthma
983771000006118	173d.00	Work aggravated asthma
98546013	H331100	Life threatening acute exacerbation of intrinsic asthma
9934311000006111		Oral steroid-dependent asthma

Cancer

medcode	readcode	desc
109371000006115	B471100	Malignant teratoma of descended testis
109381000006117	B47z.12	Malignant teratoma of testis
109391000006119	B470300	Teratoma of undescended testis
11777221000006116		[X]Malignant neoplasm of respiratory AND/OR intrathoracic organs
11777871000006119		[X]Malignant neoplasms of lymphoid, hematopoietic and related tissue
11777921000006112		[X]Neoplasms of uncertain and unknown behavior
11782261000006116		[M]Hemangiopericytic neoplasm NOS
11782311000006111		[M]Malignant lymphoma, follicular center cell, non-cleaved NOS

1179473 1000006 110		E-cadherin-associated hereditary gastric cancer
1179810 1000006 111		Leydig cell neoplasm of testis
1181219 1000006 116		[M]Malignant lymphoma, follicular center cell, cleaved, follicular
1181222 1000006 111		[M]No microscopic confirmation of tumor, clinically malignant
1181402 1000006 112		[M]Carcinoid tumor, nonargentaffin, malignant
1181449 1000006 119		[M]Malignant lymphoma, follicular center cell NOS
1181450 1000006 110		[M]Malignant lymphoma, follicular center cell, cleaved NOS
1181452 1000006 117		[M]Mixed tumor, malignant, NOS
1181457 1000006 116		[M]Specialized gonadal neoplasms
1181472 1000006 114		Pericarditis co-occurrent and due to malignant primary tumour
1181473 1000006 112		Pericarditis co-occurrent and due to malignant primary tumor
1182434 1000006 112		MPNST (malignant peripheral nerve sheath tumour) with perineurial differentiation
1182435 1000006 114		Malignant peripheral nerve sheath neoplasm with perineurial differentiation
1182436 1000006 111		MPNST (malignant peripheral nerve sheath tumor) with perineurial differentiation
1184499 1000006 116		Human epidermal growth factor 2 positive carcinoma of breast
1185269 1000006 116		Pericardial effusion co-occurrent and due to malignant neoplasm of pericardium
1187332 1000006 115		[M]Carcinoid tumor, malignant
1187352 1000006 113		[V]Personal history of malignant neoplasm of esophagus
1187353 1000006 111		[V]Personal history of malignant neoplasm of male genital organ (context-dependent category)

1187850 1000006 110		Carcinoma in situ of skin of head and neck
1188112 1000006 112		[M]Malignant lymphoma, follicular center cell, non-cleaved, follicular
1190394 1000006 110		Secondary malignant neoplasm of other digestive organ
1190395 1000006 112		Secondary malignant neoplasm of other respiratory organs
1190431 1000006 110		Secondary and unspec malignant neoplasm mastoid lymph nodes
1190432 1000006 119		Secondary malignant neoplasm of lymph nodes of head
1190453 1000006 110		Rectal carcinoma
1190594 1000006 114		[M]Acinar cell neoplasms
1190602 1000006 114		[M]Adenoma or adenocarcinoma NOS
1190605 1000006 117		[M]Adenomatous and adenocarcinomatous polyps
1190606 1000006 115		[M]Adenomatous and adenocarcinomatous polyps of colon
1190607 1000006 110		[M]Adenomatous or adenocarcinomatous polyp NOS
1190608 1000006 113		[M]Adenomatous or adenocarcinomatous polyps of the colon NOS
1190609 1000006 111		[M]Adnexal and skin appendage neoplasm NOS
1190610 1000006 117		[M]Adnexal and skin appendage neoplasm
1190617 1000006 111		[M]Angiolipomatous neoplasm NOS
1190618 1000006 114		[M]Angiolipomatous neoplasms
1190620 1000006 110		[M]Angiomyomatous neoplasm NOS
1190621 1000006 113		[M]Angiomyomatous neoplasms

1190622 1000006 117		[M]Apocrine adenoma and adenocarcinomas
1190623 1000006 119		[M]Apocrine adenoma or adenocarcinoma NOS
1190625 1000006 114		[M]Basal cell neoplasms
1190638 1000006 111		[M]Carcinoid tumour, malignant
1190639 1000006 114		[M]Carcinoid tumour, nonargentaffin, malignant
1190642 1000006 118		[M]Carcinoma NOS
1190644 1000006 113		[M]Ceruminous adenoma and adenocarcinoma
1190645 1000006 110		[M]Ceruminous adenoma or adenocarcinoma NOS
1190646 1000006 112		[M]Chondromatous neoplasm NOS
1190647 1000006 117		[M]Chondromatous neoplasms
1190649 1000006 116		[M]Choroid plexus papilloma, malignant
1190652 1000006 119		[M]Clear cell adenoma or adenocarcinoma NOS
1190653 1000006 116		[M]Clear cell adenomas and adenocarcinomas
1190655 1000006 111		[M]Comedocarcinoma NOS
1190656 1000006 113		[M]Complex epithelial neoplasms
1190657 1000006 118		[M]Complex mixed and stromal neoplasms
1190658 1000006 115		[M]Complex mixed or stromal neoplasm NOS
1190662 1000006 115		[M]Cystadenoma and carcinoma
1190663 1000006 117		[M]Cystadenoma or carcinoma NOS

1190664 1000006 110		[M]Cystic, mucinous and serous neoplasms
1190665 1000006 112		[M]Cystic, mucinous or serous neoplasm NOS
1190667 1000006 119		[M]Ductal, lobular and medullary neoplasms
1190668 1000006 116		[M]Ductal, lobular or medullary neoplasm NOS
1190669 1000006 118		[M]Endometrioid adenoma or carcinoma NOS
1190670 1000006 118		[M]Endometrioid adenomas and carcinomas
1190675 1000006 119		[M]Epithelial neoplasms NOS
1190681 1000006 112		[M]Fibroepithelial neoplasms
1190685 1000006 113		[M]Fibromatous neoplasms
1190689 1000006 119		[M]Ganglioneuromatous neoplasm NOS
1190690 1000006 115		[M]Ganglioneuromatous neoplasms
1190691 1000006 117		[M]Gastrinoma and carcinomas
1190692 1000006 113		[M]Gastrinoma or carcinoma NOS
1190693 1000006 111		[M]Germ cell neoplasms
1190711 1000006 117		[M]Haemangiopericytic neoplasm NOS
1190712 1000006 113		[M]Hepatobiliary adenoma or carcinoma NOS
1190713 1000006 111		[M]Hepatobiliary tract adenomas and carcinomas
1190726 1000006 114		[M]Infiltrating ductular carcinoma
1190729 1000006 118		[M]Intraductal carcinoma and lobular carcinoma in situ

1190730 1000006 117		[M]Intraductal carcinoma, non-infiltrating NOS
1190733 1000006 113		[M]Leiomyomatous neoplasm NOS
1190734 1000006 115		[M]Leiomyomatous neoplasms
1190749 1000006 119		[M]Lymphogranuloma, malignant
1190757 1000006 116		[M]Malignant lymphoma, follicular centre cell, cleaved, follicular
1190758 1000006 118		[M]Malignant lymphoma, lymphocytic, intermediate differentiation, nodular
1190759 1000006 115		[M]Malignant lymphoma, lymphocytic, poorly differentiated, nodular
1190761 1000006 114		[M]Malignant lymphoma, follicular centre cell, non-cleaved NOS
1190762 1000006 118		[M]Malignant lymphoma, lymphocytic, intermediate differentiation NOS
1190763 1000006 115		[M]Malignant lymphoma, lymphocytic, well differentiated, nodular
1190764 1000006 113		[M]Malignant lymphoma, centroblastic type NOS
1190765 1000006 110		[M]Malignant lymphoma, centroblastic-centrocytic, diffuse
1190766 1000006 112		[M]Malignant lymphoma, centroblastic-centrocytic, follicular
1190767 1000006 117		[M]Malignant lymphoma, centrocytic
1190768 1000006 119		[M]Malignant lymphoma, convoluted cell type NOS
1190769 1000006 116		[M]Malignant lymphoma, diffuse NOS
1190770 1000006 116		[M]Malignant lymphoma, follicular centre cell NOS
1190771 1000006 118		[M]Malignant lymphoma, follicular centre cell, cleaved NOS
1190772 1000006 114		[M]Malignant lymphoma, large cell, cleaved, diffuse

1190774 1000006 119		[M]Malignant lymphoma, lymphocytic, poorly differentiated NOS
1190775 1000006 117		[M]Malignant lymphoma, lymphocytic, well differentiated NOS
1190776 1000006 115		[M]Malignant lymphoma, mixed lymphocytic-histiocytic NOS
1190777 1000006 110		[M]Malignant lymphoma, nodular NOS
1190779 1000006 111		[M]Malignant lymphoma, small cleaved cell, diffuse
1190780 1000006 112		[M]Malignant lymphoma, undifferentiated cell type NOS
1190781 1000006 110		[M]Malignant lymphomatous polyposis
1190786 1000006 113		[M]Mesothelial neoplasms
1190787 1000006 118		Mesothelioma (malignant, clinical disorder)
1190798 1000006 110		[M]Mixed and stromal renal neoplasms
1190799 1000006 113		[M]Mixed cell adenoma and adenocarcinoma
1190800 1000006 115		[M]Mixed cell adenoma or adenocarcinoma NOS
1190802 1000006 113		[M]Mixed or stromal renal neoplasm NOS
1190803 1000006 111		[M]Mixed tumour, malignant, NOS
1190806 1000006 119		[M]Mucinous adenoma and adenocarcinoma
1190807 1000006 114		[M]Mucinous adenoma or adenocarcinoma NOS
1190808 1000006 112		[M]Mucoepidermoid neoplasms
1190813 1000006 112		[M]Myomatous neoplasms
1190814 1000006 119		[M]Myxomatous neoplasms

1190818 1000006 113		[M]Neoplasms NOS
1190821 1000006 112		[M]Neuroepitheliomatous neoplasms
1190827 1000006 115		[M]No microscopic confirmation of tumour, clinically malignant
1190840 1000006 110		[M]Ovarian cystic, mucinous and serous neoplasms
1190841 1000006 113		[M]Ovarian cystic, mucinous or serous neoplasm NOS
1190846 1000006 111		[M]Oxyphilic adenoma or adenocarcinoma NOS
1190847 1000006 116		[M]Oxyphilic adenomas and adenocarcinomas
1190848 1000006 118		[M]Pancreatic adenoma or carcinoma NOS
1190849 1000006 115		[M]Pancreatic adenomas and carcinomas
1190850 1000006 111		[M]Papillary adenoma or adenocarcinoma NOS
1190851 1000006 114		[M]Papillary adenomas and adenocarcinomas
1190852 1000006 118		[M]Papillary and squamous cell neoplasms
1190854 1000006 113		[M]Papillary neoplasms
1190855 1000006 110		[M]Papillary or squamous cell neoplasm NOS
1190860 1000006 112		[M]Parathyroid adenoma or adenocarcinoma NOS
1190861 1000006 110		[M]Parathyroid adenomas and adenocarcinomas
1190865 1000006 111		[M]Pituitary adenoma or carcinoma NOS
1190866 1000006 113		[M]Pituitary adenomas and carcinomas
1190873 1000006 113		[M]Renal adenoma and carcinoma

1190874 1000006 115		[M]Renal adenoma or carcinoma NOS
1190875 1000006 118		[M]Respiratory tract adenoma or adenocarcinoma NOS
1190876 1000006 116		[M]Respiratory tract adenomas and adenocarcinomas
1190881 1000006 111		[M]Rhabdomyomatous neoplasm NOS
1190882 1000006 115		[M]Rhabdomyomatous neoplasms
1190884 1000006 110		[M]Sebaceous adenoma and adenocarcinoma
1190885 1000006 112		[M]Sebaceous adenoma or adenocarcinoma NOS
1190892 1000006 112		[M]Skin appendage adenoma and carcinoma
1190893 1000006 110		[M]Skin appendage adenoma or carcinoma NOS
1190897 1000006 113		[M]Solid carcinoma NOS
1190900 1000006 114		[M]Specialised gonadal neoplasms
1190901 1000006 112		[M]Spheroidal cell carcinoma
1190904 1000006 111		[M]Squamous cell carcinoma NOS
1190905 1000006 113		[M]Squamous cell carcinoma, metastatic NOS
1190906 1000006 110		[M]Strumal neoplasm NOS
1190907 1000006 115		[M]Strumal neoplasms
1190911 1000006 111		[M]Sweat gland adenoma and adenocarcinomas
1190912 1000006 115		[M]Sweat gland adenoma or adenocarcinoma NOS
1190914 1000006 110		[M]Synovial neoplasms

1190915 1000006 112		[M]Synovial neoplasm NOS
1190921 1000006 117		[M]Theca cell carcinoma
1190922 1000006 113		[M]Thecal cell neoplasms
1190923 1000006 111		[M]Thecal cell neoplasm NOS
1190925 1000006 116		[M]Thyroid adenoma and adenocarcinoma
1190926 1000006 119		[M]Thyroid adenoma or adenocarcinoma NOS
1190927 1000006 114		[M]Transitional cell papilloma or carcinoma NOS
1190928 1000006 112		[M]Trophoblastic neoplasms
1190930 1000006 111		[M]Tubular adenoma or adenocarcinoma NOS
1190931 1000006 114		[M]Tubular adenomas and adenocarcinomas
1190934 1000006 113		[M]Urothelial carcinoma
1190935 1000006 110		[M]Villous adenoma or adenocarcinoma NOS
1190936 1000006 112		[M]Villous adenomas and adenocarcinomas
1191166 1000006 118		[V]Personal history of malignant neoplasm of male genital organ
1191177 1000006 115		[V]Personal history of malignant neoplasm of gastrointestinal tract
1191179 1000006 119		[V]Personal history of malignant neoplasm of trachea, bronchus or lung
1191180 1000006 118		[V]Personal history of malignant neoplasm of anus
1191181 1000006 115		[V]Personal history of malignant neoplasm of bladder
1191182 1000006 111		[V]Personal history of malignant neoplasm of breast

1191183 1000006 114		[V]Personal history of malignant neoplasm of bronchus
1191184 1000006 116		[V]Personal history of malignant neoplasm of cervix uteri
1191185 1000006 119		[V]Personal history of malignant neoplasm of genital organ
1191187 1000006 112		[V]Personal history of malignant neoplasm of intestine
1191189 1000006 113		[V]Personal history of malignant neoplasm of kidney
1191190 1000006 112		[V]Personal history of malignant neoplasm of large intestine
1191191 1000006 110		[V]Personal history of malignant neoplasm of liver
1191192 1000006 119		[V]Personal history of malignant neoplasm of lung
1191193 1000006 116		[V]Personal history of malignant neoplasm of oesophagus
1191194 1000006 114		[V]Personal history of malignant neoplasm of ovary
1191195 1000006 111		[V]Personal history of malignant neoplasm of prostate
1191196 1000006 113		[V]Personal history of malignant neoplasm of rectum
1191197 1000006 118		[V]Personal history of malignant neoplasm of stomach
1191198 1000006 115		[V]Personal history of malignant neoplasm of testis
1191199 1000006 117		[V]Personal history of malignant neoplasm of tongue
1191200 1000006 115		[V]Personal history of malignant neoplasm of trachea
1191201 1000006 117		[V]Personal history of malignant neoplasm of urinary organ
1191202 1000006 113		[V]Personal history of malignant neoplasm of uterine body
1191719 1000006 113		[X]In situ neoplasms

1191808 1000006 110		[X]Mal neoplasm/overlap les/periph nerv+autonomic nerv systm
1191812 1000006 112		[X]Malignant neoplasm of breast
1191813 1000006 110		[X]Malignant neoplasm of bronchus or lung, unspecified
1191814 1000006 117		[X]Malignant neoplasm of eye, brain and other parts of central nervous system
1191816 1000006 118		[X]Malignant neoplasm of female genital organs
1191817 1000006 113		[X]Malignant neoplasm of ill-defined, secondary and unspecified sites
1191819 1000006 114		Malignant tumor of male genital organ
1191820 1000006 112		Malignant tumor of mediastinum
1191821 1000006 110		[X]Malignant neoplasm of mesothelial and soft tissue
1191822 1000006 119		[X]Malignant neoplasm of respiratory and intrathoracic organs
1191823 1000006 116		[X]Malignant neoplasm of thyroid and other endocrine glands
1191824 1000006 114		[X]Malignant neoplasm of urinary tract
1191825 1000006 111		[X]Malignant neoplasm-pluriglandular involvement,unspecified
1191826 1000006 113		[X]Malignant neoplasm/bones+articular cartilage/limb,unspfd
1191827 1000006 118		[X]Malignant neoplasm/other specified female genital organs
1191828 1000006 115		[X]Malignant neoplasm/overlap lesion/bone+articulr cartilage
1191829 1000006 117		[X]Malignant neoplasm/overlap lesion/other+ill-defined sites
1191830 1000006 116		[X]Malignant neoplasm/overlapping lesion/feml genital organs
1191831 1000006 118		[X]Malignant neoplasm/overlapping lesion/male genital organs

1191832<br>1000006<br>114		Malignant tumour of peripheral nerve
1191834<br>1000006<br>119		[X]Malignant neoplasms of lymphoid, haematopoietic and related tissue
1191837<br>1000006<br>110		[X]Melanoma and other malignant neoplasms of skin
1191872<br>1000006<br>113		[X]Neoplasms of uncertain and unknown behaviour
1191960<br>1000006<br>115		[X]Other malignant immunoproliferative diseases
1191985<br>1000006<br>115		Liver cell carcinoma
1192343<br>1000006<br>116		Carcinoma in situ of lip, oral cavity and pharynx
1192346<br>1000006<br>113		Carcinoma in situ of skin of lower limb and hip
1192348<br>1000006<br>115		Carcinoma of rectum
1192553<br>1000006<br>118		Malignant neoplasm of ventral tongue surface NOS
1192555<br>1000006<br>113		Malignant tumor of vulva
1192556<br>1000006<br>110		Malignant neoplasm, overlap lesion of resp & intrathor orgs
1192557<br>1000006<br>115		Malignant neoplasm of trunk NOS
1192558<br>1000006<br>117		Malignant tumor of small intestine
1192559<br>1000006<br>119		Malignant neoplasm of stomach NOS
1192560<br>1000006<br>110		Malignant neoplasm of supraclavicular fossa NOS
1192561<br>1000006<br>113		Malignant neoplasm of prepylorus of stomach
1192562<br>1000006<br>117		Malignant neoplasm of placenta
1192563<br>1000006<br>119		Malignant neoplasm of posterior wall of stomach NEC

1192568 1000006 118		Malignant neoplasm of nose NOS
1192569 1000006 115		Malignant neoplasm of lip unspecified, inner aspect
1192570 1000006 115		Malignant neoplasm of lip, unspecified, lipstick area
1192572 1000006 113		Malignant neoplasm of long bones of leg NOS
1192573 1000006 111		Malignant tumor of head and neck
1192574 1000006 118		Malignant neoplasm of inguinal region NOS
1192576 1000006 119		Malignant neoplasm of jaw NOS
1192578 1000006 112		Malignant neoplasm of flank NOS
1192581 1000006 114		Malignant neoplasm of cheek NOS
1192582 1000006 118		Malignant neoplasm of chest wall NOS
1192583 1000006 115		Malignant neoplasm of anus unspecified
1192584 1000006 113		Malignant neoplasm of aortic body and other paraganglia
1192586 1000006 112		Malignant neoplasm of back NOS
1192587 1000006 117		Malignant neoplasm of bone and articular cartilage NOS
1192588 1000006 119		Malignant tumor of lung
1192589 1000006 116		Malignant neoplasm of accessory sinus NOS
1192590 1000006 117		Malignant neoplasm of anterior wall of stomach NEC
1192591 1000006 119		Malignant melanoma of other and unspecified parts of face
1192592 1000006 110		Malignant lymphoma NOS of intra-abdominal lymph nodes

1192593 1000006 113		Malignant lymphoma NOS of intrapelvic lymph nodes
1192594 1000006 115		Malignant lymphoma NOS of intrathoracic lymph nodes
1192595 1000006 118		Malignant lymphoma NOS of lymph node inguinal region and leg
1192596 1000006 116		Malignant lymphoma NOS of lymph nodes of axilla and arm
1192597 1000006 111		Malignant lymphoma NOS of lymph nodes of head, face and neck
1192598 1000006 114		Malignant lymphoma NOS of lymph nodes of multiple sites
1192600 1000006 113		Malignant lymphoma NOS of spleen
1192859 1000006 113		Primary malignant neoplasm of descended testis
1192866 1000006 116		[M]Squamous cell carcinoma of skin NOS
1198970 1000006 113		Human immunodeficiency virus with secondary cancers
1199922 1000006 110		Adenoid basal carcinoma of cervix uteri
1199923 1000006 113		Cervical adenoid basal carcinoma
1199924 1000006 115		Adenoid cystic carcinoma of cervix uteri
1199925 1000006 118		Cervical adenoid cystic carcinoma
1200027 1000006 114		Clear cell adenocarcinoma of ovary
1200028 1000006 112		Ovarian clear cell adenocarcinoma
1200554 1000006 114		Metaplastic carcinoma of breast
1202026 1000006 111		MiT family translocation renal cell carcinoma
1202027 1000006 116		Translocation renal cell carcinoma

1202087 1000006 115		Squamous cell carcinoma of small intestine
1202088 1000006 117		Squamous cell carcinoma of small bowel
1202090 1000006 115		Squamous cell carcinoma of corpus uteri
1202224 1000006 111		Adenocarcinoma of anal canal
1202225 1000006 113		Adenocarcinoma of penis
1202226 1000006 110		Penile adenocarcinoma
1202241 1000006 113		Acquired cystic disease associated renal cell carcinoma
1202365 1000006 119		Lymphoepithelial carcinoma
1202366 1000006 117		Lymphoepithelial-like carcinoma
1202406 1000006 114		Hereditary clear cell renal cell carcinoma
1202407 1000006 119		Hereditary clear cell renal cell adenocarcinoma
1202455 1000006 118		Mucinous tubular and spindle cell renal carcinoma
1202456 1000006 116		Renal mucinous tubular and spindle cell carcinoma
1202457 1000006 111		Renal mucinous tubular and spindle cell carcinoma
1202618 1000006 112		Renal medullary carcinoma
1202619 1000006 110		Renal medullary carcinoma
1203564 1000006 118		Adenocarcinoma of gallbladder and extrahepatic biliary tract
1204780 1000006 112		Undifferentiated carcinoma of stomach
1204781 1000006 110		Undifferentiated gastric carcinoma

1204782 1000006 119		Undifferentiated carcinoma of corpus uteri
1204975 1000006 111		Glassy cell carcinoma of cervix uteri
1205053 1000006 115		Squamous cell carcinoma of gallbladder and extrahepatic biliary tract
1205054 1000006 113		Squamous cell carcinoma of rectum
1205055 1000006 110		Rectal squamous cell carcinoma
1205056 1000006 112		Squamous cell carcinoma of stomach
1205057 1000006 117		Gastric squamous cell carcinoma
1205058 1000006 119		Squamous cell carcinoma of colon
1205629 1000006 117		Germline BRCA-mutated, HER2-negative metastatic breast cancer
1205630 1000006 116		gBRCA-mutated, HER2(-) metastatic breast carcinoma
1205631 1000006 118		Germline BRCA-mutated human epidermal growth factor receptor 2 negative metastatic carcinoma of breast
1205764 1000006 110		Intraepithelial squamous cell carcinoma of anogenital region
1208876 015	B50 ..00	Malignant neoplasm of eye
1210553 016	B54 000 0	Malignant neoplasm of adrenal cortex
1210554 010	B54 010 0	Malignant neoplasm of adrenal medulla
1210561 014	B43 ..00	Malignant neoplasm of body of uterus
1210566 016	B45 3.00	Malignant neoplasm of clitoris
1210642 019	B34 ..00	Malignant neoplasm of female breast
1210775 1000006 115		Merkel cell carcinoma of right lower limb
1210782 1000006 117		Merkel cell carcinoma of left lower limb
1210785 1000006 114		Malignant melanoma of skin of left upper limb

1210786 1000006 111		Primary malignant neoplasm of soft tissue of left upper extremity
1210787 1000006 116		Primary malignant neoplasm of soft tissue of right upper extremity
1210788 1000006 118		Primary malignant neoplasm of soft tissue of right lower extremity
1210789 1000006 115		Primary malignant neoplasm of soft tissue of left lower limb
1210790 1000006 116		Overlapping primary malignant neoplasm of bone and articular cartilage of right upper limb
1210792 1000006 114		Overlapping primary malignant neoplasm of bone and articular cartilage of left upper limb
1210793 1000006 112		Primary malignant neoplasm of bone of left upper limb
1210794 1000006 119		Primary malignant neoplasm of bone of left lower limb
1210795 1000006 117		Primary malignant neoplasm of bone of right upper limb
1210796 1000006 115		Primary malignant neoplasm of bone of right lower limb
1211679 013	B60 0.00	Reticulosarcoma
1212217 1000006 115		History of malignant neoplasm of digestive organ
1215809 015	B57 600 0	Secondary malignant neoplasm of retroperitoneum
1215862 010	B10 6.00	Malignant neoplasm, overlapping lesion of oesophagus
1216064 018	BB2 C.00	[M]Squamous cell carcinoma, keratinising type NOS
1216356 017	BB5 R60 0	[M]Mucocarcinoid tumour, malignant
1216359 012	ZV1 001 6	[V]Personal history of malignant neoplasm of oesophagus
1216363 017	ZV1 071 3	[V]Personal history of other haematopoietic neoplasm
1216442 018	BBe 9.00	[M]Triton tumour, malignant
1216464 010	B13 4.00	Malignant neoplasm of caecum
1216486 011	B10 z.11	Oesophageal cancer
1216488 012	B10 ..00	Malignant neoplasm of oesophagus

1216670013	BB5R300	[M]Carcinoid tumour, argentaffin, malignant
1216731014	BBTK.00	[M]Epithelioid haemangioendothelioma, malignant
1216845011	BBEV.00	[M]Blue naevus, malignant
1217274016	B801200	Carcinoma in situ of lower 1/3 oesophagus
1217275015	B801100	Carcinoma in situ of middle 1/3 oesophagus
1217276019	B801000	Carcinoma in situ of upper 1/3 oesophagus
1217360013	B084.00	Malignant neoplasm, overlapping lesion of hypopharynx
1217367011	B508.00	Malignant neoplasm, overlapping lesion of eye and adnexa
1219467016	B074.00	Malignant neoplasm, overlapping lesion of nasopharynx
1219468014	B214.00	Malignant neoplasm, overlapping lesion of larynx
1219531013	B017.00	Malignant overlapping lesion of tongue
1219533011	B042.00	Malignant neoplasm, overlapping lesion of floor of mouth
1219534017	B117.00	Malignant neoplasm, overlapping lesion of stomach
1219535016	B124.00	Malignant neoplasm, overlapping lesion of small intestine
1219537012	B163.00	Malignant neoplasm, overlapping lesion of biliary tract
1219538019	B175.00	Malignant neoplasm, overlapping lesion of pancreas
1219540012	B487.00	Malignant neoplasm, overlapping lesion of penis
1219545019	B627600	Diffuse non-Hodgkin's immunoblastic (diffuse) lymphoma
1219789016	BB5S200	[M]Bronchiolo-alveolar adenocarcinoma
12201281000006111		Malignant mixed mesodermal tumor
12201291000006114		Malignant mixed mesodermal tumour
1220414014	B614.00	Hodgkin's disease, nodular sclerosis
12204961000006112		Metastatic colorectal cancer
1221171011	F122.00	Malignant neuroleptic syndrome

1221209015	BB5D800	[M] Hepatocellular carcinoma, fibrolamellar
1221446018	BBEG000	[M]Acral lentiginous melanoma, malignant
1221504012	BBLG.00	[M]Carcinoma in pleomorphic adenoma
1222216017	BB5f111	[M]Follicular carcinoma
12222311000006112		Malignant pleural mesothelioma
1222289015	BBg7.00	[M]Malignant lymphoma, lymphoplasmacytoid type
12222981000006116		History of cancer
1222303015	BBQ7200	[M]Teratoma, malignant, NOS
1222626017	BB1N.00	[M]Small cell-large cell carcinoma
1224870013	BB5D500	[M]Hepatocellular carcinoma NOS
1227552010	ZV10013	[V]Personal history of malignant neoplasm of intestine
1227553017	ZV10018	[V]Personal history of malignant neoplasm of stomach
1227554011	ZV10017	[V]Personal history of malignant neoplasm of rectum
1227555012	ZV10011	[V]Personal history of malignant neoplasm of anus
1227556013	ZV10019	[V]Personal history of malignant neoplasm of tongue
1227557016	ZV10014	[V]Personal history of malignant neoplasm of large intestine
1227558014	ZV10015	[V]Personal history of malignant neoplasm of liver
1227559018	ZV10111	[V]Personal history of malignant neoplasm of bronchus
1227560011	ZV10112	[V]Personal history of malignant neoplasm of lung
1227561010	ZV10113	[V]Personal history of malignant neoplasm of trachea

1227562015	ZV10213	[V]Personal history of malignant neoplasm of middle ear
1227563013	ZV10211	[V]Personal history of malignant neoplasm - accessory sinus
1227564019	ZV10212	[V]Personal history of malignant neoplasm of larynx
1227565018	ZV10214	[V]Personal history of malignant neoplasm of nose
1227566017	ZV10416	[V]Personal history of malignant neoplasm of testis
1227568016	ZV10417	[V]Personal history of malignant neoplasm of uterine body
1227569012	ZV10415	[V]Personal history of malignant neoplasm of prostate
1227571012	ZV10414	[V]Personal history of malignant neoplasm of ovary
1227572017	ZV10411	[V]Personal history of malignant neoplasm of cervix uteri
1227573010	ZV10511	[V]Personal history of malignant neoplasm of bladder
1227574016	ZV10513	[V]Personal history of malignant neoplasm of kidney
1227579014	ZV10y14	[V]Personal history of malignant neoplasm of skin
1227580012	ZV10y11	[V]Personal history of malignant neoplasm of bone
1227581011	ZV10y12	[V]Personal history of malignant neoplasm of brain
1227582016	ZV10y15	[V]Personal history of malignant neoplasm of thyroid
1227583014	ZV10y13	[V]Personal history of malignant neoplasm of eye
1228188010	BB5y000	Basal cell adenocarcinoma
1228259019	BB91100	[M]Infiltrating duct and lobular carcinoma
1228493018	B41..00	Malignant neoplasm of cervix uteri
1228504019	B000.00	Malignant neoplasm of upper lip, vermilion border

1228506017	B001100	Malignant neoplasm of lower lip, lipstick area
1228509012	B010.11	Malignant neoplasm of posterior third of tongue
1228510019	B02..00	Malignant neoplasm of major salivary glands
1228513017	B021.00	Malignant neoplasm of submandibular gland
1228523014	B055100	Malignant neoplasm of roof of mouth
1228529013	B060100	Malignant neoplasm of palatine tonsil
1228530015	B060000	Malignant neoplasm of faucial tonsil
1228532011	B070.00	Malignant neoplasm of roof of nasopharynx
1228549016	B180.00	Malignant neoplasm of retroperitoneum
1228552012	B200.00	Malignant neoplasm of nasal cavities
1228553019	B203.00	Malignant neoplasm of ethmoid sinus
1228565015	B310000	Malignant neoplasm of soft tissue of head
1228566019	B310100	Malignant neoplasm of soft tissue of face
1228567011	B310200	Malignant neoplasm of soft tissue of neck
1228570010	B440.11	Cancer of ovary
1228575017	B480.00	Malignant neoplasm of prepuce (foreskin)
1229424011	BB5y500	[M]Lipid-rich carcinoma
1229572014	BBE1000	[M]Malignant melanoma, regressing
1229648010	BBk7.00	[M]Malignant lymphoma, centroblastic type, follicular
1230100013	BBEG.00	[M]Malignant melanoma in Hutchinson's melanotic freckle
1230128019	BBR2.00	[M]Choriocarcinoma
1230392016	BBgR.00	[M]Malignant lymphoma, large cell, diffuse NOS
1230440012	BB5L300	[M]Adenocarcinoma in multiple adenomatous polyps

1230709016	BB51000	[M]Adenocarcinoma in situ in villous adenoma
1230828016	BBgP.00	[M]Malignant lymphoma, mixed small and large cell, diffuse
1230838014	BBp2.00	[M]Malignant mastocytosis
1230882017	BB5RA00	[M] Merkel cell carcinoma
1231004013	BB51100	[M]Adenocarcinoma in situ in tubulovillous adenoma
1231028010	BBE1100	[M]Desmoplastic melanoma, malignant
1231089017	BB5D700	[M]Combined hepatocellular carcinoma and cholangiocarcinoma
1231363012	BB9K000	[M]Paget's disease and intraductal carcinoma of breast
1231370012	BB69100	[M]Sebaceous adenocarcinoma
1231544010	BB5R900	[M] Neuroendocrine carcinoma
1231622010	BB52000	[M]Adenocarcinoma in tubulovillous adenoma
1231967018	BB29.00	[M]Squamous cell carcinoma in situ NOS
1231976013	BB1M.00	[M]Small cell carcinoma, intermediate cell
1232066010	BB5L200	[M]Adenocarcinoma in situ in adenomatous polyp
1232564019	BB91000	[M]Intraductal papillary adenocarcinoma with invasion
1232576019	BBgL.00	[M]Malignant lymphoma, small lymphocytic NOS
1233222010	BB14.00	[M]Carcinomatosis
1233722011	BB5y600	[M]Glycogen-rich carcinoma
1234144017	F373.00	Polyneuropathy in malignant disease
1234197011	BBg8.00	[M]Malignant lymphoma, immunoblastic type
1235115011	BB01.00	[M]Neoplasm, uncertain whether benign or malignant
1235762013	B823000	Carcinoma in situ of skin of forehead skin

1235785014	B323000	Malignant melanoma of external surface of cheek
1235786010	B323200	Malignant melanoma of eyebrow
1235798017	B323400	Malignant melanoma of external surface of nose
1235814012	B585.00	Secondary malignant neoplasm of bone and bone marrow
1235875018	B576100	Secondary malignant neoplasm of peritoneum
1235961012	BBB7.00	[M]Epithelial-myoepithelial carcinoma
123978016	B62F100	Mantle cell lymphoma
12451621000006115		Secondary malignant neoplasm of large intestine and rectum
12455781000006112		[M]Basal cell neoplasms
12455841000006113		[M]Papillary neoplasms
12457061000006111		[V]Chemotherapy session for neoplasm
12458141000006110		[V]Follow-up examination after combined treatment for malignant neoplasm
12458191000006118		[V]Follow-up examination after radiotherapy for malignant neoplasm
12458211000006117		[V]Follow-up examination after chemotherapy for malignant neoplasm
12458231000006111		[V]Follow-up examination after surgery for malignant neoplasm
12458261000006119		[V]Follow-up examination after other treatment for malignant neoplasm
12458271000006114		[V]Follow-up examination after treatment for conditions other than malignant neoplasms
12458281000006112		[V]Follow-up examination after unspecified treatment for malignant neoplasm
12459651000006111		[V]Personal history of malignant neoplasm of other respiratory or intrathoracic organ
12459661000006113		[V]Personal history of malignant neoplasm

1245967 1000006 118		[V]Personal history of malignant neoplasm - accessory sinus
1245968 1000006 115		[V]Personal history of malignant neoplasm of breast
1245969 1000006 117		[V]Personal history of malignant neoplasm of larynx
1245970 1000006 117		[V]Personal history of malignant neoplasm of middle ear
1245971 1000006 119		[V]Personal history of malignant neoplasm of nose
1246918 1000006 119		[X]Follow-up examination after other treatment for malignant neoplasm
1246920 1000006 118		[X]Follow-up examination after unspecified treatment for malignant neoplasm
1247309 1000006 110		Malignant tumor of female genital organ
1247883 1000006 110		[X]Personal history of malignant neoplasm of other respiratory and intrathoracic organs
1248902 1000006 116		History of malignant neoplasm of skin
1261683 1000006 118		BPDCN (blastic plasmacytoid dendritic cell neoplasm) tumour and germline WGS (whole genome sequencing)
1261760 1000006 111		Paediatric FLHCC (fibrolamellar hepatocellular carcinoma) tumour and germline WGS (whole genome sequencing)
1261769 1000006 116		Paediatric midline carcinoma tumour and germline WGS (whole genome sequencing)
1261774 1000006 119		Paediatric thyroid papillary carcinoma tumour and germline WGS (whole genome sequencing)
1261785 1000006 111		Paediatric t(6;11) translocation-associated RCC (renal cell carcinoma) tumour and germline WGS (whole genome sequencing)
1261787 1000006 118		Paediatric Xp11.2 translocation-associated renal cell carcinoma tumour and germline WGS (whole genome sequencing)
1270355 1000006 113		Carcinoma in situ of urinary tract proper
1270377 1000006 114		Primary malignant neoplasm of skin
1270458 1000006 119		Malignant tumour of posterior margin of nasal septum and choanae

1270459 1000006 116		Malignant neoplasm skin of other and unspecified parts face
1270460 1000006 112		Malignant tumour of upper labial mucosa
1271628 1000006 114		Primary malignant neoplasm of intrahepatic bile ducts
1271693 1000006 116		Malignant neoplasm of long bones of leg
1271819 1000006 116		[M]Carcinoma, metastatic, NOS
1271882 1000006 112		Malignant neoplasm of skin of trunk, excluding scrotum
1271897 1000006 119		Malignant neoplasm of ovary and other uterine adnexa
1271898 1000006 116		Secondary and unspecified malignant neoplasm of lymph nodes of head, face and neck
1271908 1000006 110		Malignant neoplasm of lip, oral cavity and pharynx NOS
1271909 1000006 113		Malignant neoplasm of lesser curve of stomach unspecified
1271910 1000006 119		Malignant neoplasm of greater curve of stomach unspecified
1272065 1000006 110		Secondary and unspecified malignant neoplasm of intrathoracic lymph nodes
1272109 1000006 119		Secondary and unspecified malignant neoplasm of axilla and upper limb lymph nodes
1272110 1000006 113		Secondary and unspecified malignant neoplasm of inguinal and lower limb lymph nodes
1272121 1000006 117		Other malignant neoplasm of lymphoid and histiocytic tissue
1272124 1000006 118		Malignant neoplasm of skin NOS
1272127 1000006 114		Secondary and unspecified malignant neoplasm of intra-abdominal lymph nodes
1272136 1000006 112		Secondary and unspecified malignant neoplasm of lymph nodes in multiple sites
1272137 1000006 117		Secondary and unspecified malignant neoplasm of lymph nodes NOS

1272138<br>1000006<br>119		Malignant neoplasm of other and unspecified site NOS
1272152<br>1000006<br>111		Immunoproliferative neoplasm or myeloma NOS
1272295<br>1000006<br>118		Malignant neoplasm of other and ill-defined sites
1272326<br>1000006<br>118		Secondary malignant neoplasm of other digestive organ
1272328<br>1000006<br>111		Malignant melanoma of skin NOS
1272333<br>1000006<br>119		Secondary and unspecified malignant neoplasm of intrapelvic lymph nodes
1272347<br>1000006<br>112		Malignant neoplasm of female breast NOS
1272352<br>1000006<br>110		Malignant neoplasm of larynx NOS
1272442<br>1000006<br>114		Malignant neoplasm lymphatic or haematopoietic tissue NOS
1272454<br>1000006<br>118		Malignant neoplasm of anus unspecified
1272543<br>1000006<br>113		Malignant neoplasm of rectum, rectosigmoid junction and anus NOS
1272662<br>1000006<br>116		Secondary malignant neoplasm of other specified site NOS
1272663<br>1000006<br>118		Malignant neoplasm of unspecified site
1272664<br>1000006<br>111		Other malignant neoplasm NOS
1273014<br>1000006<br>116		Malignant neoplasm of liver and intrahepatic bile ducts NOS
1273174<br>1000006<br>112		Malignant neoplasm of bone and articular cartilage NOS
1273204<br>1000006<br>119		Malignant neoplasm of body of uterus NOS
1273222<br>1000006<br>117		Other malignant neoplasm of skin
1273223<br>1000006<br>119		Malignant neoplasm of uterine adnexa NOS

1273273 1000006 114		Malignant neoplasm of kidney or urinary organs NOS
1273279 1000006 113		Malignant neoplasm of penis, part unspecified
1273280 1000006 114		Malignant neoplasm of genitourinary organ NOS
1275925 1000006 116		Squamous cell carcinoma of skin NOS
1276188 1000006 119		[X]Neoplasms of uncertain and unknown behaviour
1276211 1000006 111		Malignant tumor of ureter
1276212 1000006 115		Malignant tumor of thyroid gland
1276213 1000006 117		Malignant tumor of tonsil
1276214 1000006 110		Malignant tumor of stomach
1276215 1000006 112		Malignant tumor of rectum
1276216 1000006 114		Malignant tumor of head of pancreas
1276217 1000006 119		Malignant tumor of hepatic flexure
1276219 1000006 118		Malignant tumor of gallbladder
1276220 1000006 115		Malignant tumor of descending colon
1276221 1000006 117		Malignant tumor of duodenum
1276222 1000006 113		Malignant tumor of base of tongue
1276223 1000006 111		Malignant neoplasm of adrenal gland NOS
1303144 1000006 113		Malignant tumor of ureter
1303145 1000006 110		Malignant tumor of thyroid gland

1303146 1000006 112		Malignant tumor of tonsil
1303147 1000006 117		Malignant tumor of stomach
1303148 1000006 119		Malignant tumor of rectum
1303149 1000006 116		Malignant tumor of head of pancreas
1303150 1000006 112		Malignant tumor of hepatic flexure
1303151 1000006 110		Malignant tumor of gallbladder
1303152 1000006 119		Malignant tumor of descending colon
1303153 1000006 116		Malignant tumor of duodenum
1303154 1000006 114		Malignant tumor of base of tongue
1310460 10	G20 0.00	Malignant essential hypertension
1336780 11	B67 4.00	Acute panmyelosis
1381040 14	H51 y70 0	Malignant pleural effusion
1395710 0000611 7	B63 010 0	Solitary myeloma
1408960 18	A22 0.11	Malignant pustule
1422710 18	M1 444 00	Malignant pemphigus
1448490 16	B54 6.00	Neuroblastoma
1466510 0000611 8	B62 2.00	Sezary's disease
1488663 010	44a 4.00	Squamous cell carcinoma antigen level
1488780 018	8CL 0.00	Cancer diagnosis discussed
1488798 019	1O0 ..00	Cancer confirmed
1489328 015	8CL 1.00	Cancer diagnosis discussed with significant other
1489329 011	8CL 2.00	Cancer diagnosis discussed with patient

15110100000611 6	B575z0 0	Secondary malig neop of large intestine or rectum NOS
15111100000611 8	B57 ..00	Secondary malignant neoplasm of respiratory and digestive systems
15112100000611 4	B57 z.00	Secondary malig neop of respiratory or digestive system NOS
15113100000611 2	B57 6.00	Secondary malignant neoplasm of retroperitoneum and peritoneum
15114100000611 9	B576z0 0	Secondary malig neop of retroperitoneum or peritoneum NOS
15115100000611 7	B574z0 0	Secondary malig neop of small intestine or duodenum NOS
15116100000611 5	G24 0.00	Malignant secondary hypertension
15132100000611 1	B57 5.00	Secondary malignant neoplasm of large intestine
15133100000611 4	B15 3.00	Secondary malignant neoplasm of liver
15140100000611 0	B58yz0 0	Secondary malignant neoplasm of other specified site NOS
15142100000611 7	B58 ..00	Secondary malignant neoplastic disease
15162100000611 9	B57 4.00	Secondary malignant neoplasm of small intestine
15261100000611 6	B560z0 0	Secondary unspec malig neop lymph nodes head/face/neck NOS
153085017	B8yy10 0	Carcinoma in situ of adrenal gland
153088015	B808600	Carcinoma in situ of ampulla of Vater
153089011	B80 5.00	Carcinoma in situ of anal canal
153099018	B803500	Carcinoma in situ of appendix
153102018	B803600	Carcinoma in situ of ascending colon
153107012	B83 7.00	Carcinoma in situ of bladder
153111018	B802200	Carcinoma in situ of body of stomach

153112013	B832.11	Carcinoma in situ of body of uterus
153124018	B802000	Carcinoma in situ of cardia of stomach
153129011	B831.00	CIS - Carcinoma in situ of cervix
153133016	B803.00	Carcinoma in situ of colon
153136012	B808500	Carcinoma in situ of common bile duct
153140015	B808400	Carcinoma in situ of cystic duct
153141016	B803200	Carcinoma in situ of descending colon
153143018	B807000	Carcinoma in situ of duodenum
153146014	B831000	Carcinoma in situ of endocervix
153149019	B832000	Carcinoma in situ of endometrium
153150019	B836100	Carcinoma in situ of epididymis
153151015	B810200	Carcinoma in situ of epiglottis
153155012	B81y700	Carcinoma in situ of ethmoidal sinus
153156013	B81y400	Carcinoma in situ of eustachian tube
153157016	B831100	Carcinoma in situ of exocervix
153160011	B8y0.00	Carcinoma in situ of eye
153162015	B833100	Carcinoma in situ of fallopian tube
153169012	B800400	Carcinoma in situ of floor of mouth
153173010	B81y800	Carcinoma in situ of frontal sinus
153174016	B802100	Carcinoma in situ of fundus of stomach
153180012	B810811	Carcinoma in situ of glottis

153187010	B803000	Carcinoma in situ of hepatic flexure of colon
153191017	B800900	Carcinoma in situ of hypopharynx
153193019	B807200	Carcinoma in situ of ileum
153200017	B808100	Carcinoma in situ of intrahepatic bile ducts
153203015	B807100	Carcinoma in situ of jejunum
153218019	B810.00	Carcinoma in situ of larynx
153229015	B800000	Carcinoma in situ of lip
153231012	B808000	Carcinoma in situ of liver
153239014	B812100	Carcinoma in situ of main bronchus
153246017	B81y500	Carcinoma in situ of mastoid air cells
153247014	B81y600	Carcinoma in situ of maxillary sinus
153248016	B807300	Carcinoma in situ of Meckel's diverticulum
153256018	B81y100	Carcinoma in situ of nasal cavity
153258017	B800700	Carcinoma in situ of nasopharynx
153265013	B800800	Carcinoma in situ of oropharynx
153267017	B833000	Carcinoma in situ of ovary
153268010	B800600	Carcinoma in situ of palate
153272014	B80z000	Carcinoma in situ of pancreas
153276012	B8yy200	Carcinoma in situ of parathyroid gland
153280019	B835.00	Carcinoma in situ of penis

1532820 10	B82 580 0	Carcinoma in situ of perianal skin
1532830 17	B80 0.12	Carcinoma in situ of pharynx
1532860 13	B8y y30 0	Carcinoma in situ of pituitary gland
1532880 14	B81 y00 0	Carcinoma in situ of pleura
1532950 17	B83 4.00	Carcinoma in situ of prostate
1532960 16	B80 230 0	Carcinoma in situ of pyloric antrum
1532990 11	B80 400 0	Carcinoma in situ of rectosigmoid junction
1533000 15	B80 410 0	Carcinoma in situ of rectum
1533110 11	B83 630 0	Carcinoma in situ of scrotum
1533130 14	B80 330 0	Carcinoma in situ of sigmoid colon
1533170 10	B82 520 0	Carcinoma in situ of skin of axilla
1533180 17	B82 530 0	Carcinoma in situ of skin of back
1533190 13	B82 500 0	Carcinoma in situ of skin of breast
1533200 19	B82 570 0	Carcinoma in situ of skin of buttock
1533210 15	B82 330 0	Carcinoma in situ of skin of cheek
1533270 16	B82 310 0	Carcinoma in situ of skin of eyebrow
1533320 15	B82 740 0	Carcinoma in situ of skin of foot
1533350 18	B82 550 0	Carcinoma in situ of skin of groin
1533360 17	B82 630 0	Carcinoma in situ of skin of hand
1533370 14	B82 700 0	Carcinoma in situ of skin of hip

153338016	B827200	Carcinoma in situ of skin of knee
153340014	B820.00	Carcinoma in situ of skin of lip
153343011	B824100	Carcinoma in situ of skin of neck
153344017	B823400	Carcinoma in situ of skin of nose
153345016	B825600	Carcinoma in situ of skin of perineum
153348019	B826000	Carcinoma in situ of skin of shoulder
153350010	B827100	Carcinoma in situ of skin of thigh
153358015	B82..00	Carcinoma in situ of skin
153363016	B836200	Carcinoma in situ of spermatic cord
153364010	B81y900	Carcinoma in situ of sphenoidal sinus
153365011	B80z100	Carcinoma in situ of spleen
153367015	B802.00	Carcinoma in situ of stomach
153376010	B836000	Carcinoma in situ of testis
153380017	B8yy000	Carcinoma in situ of thyroid
153382013	B800100	Carcinoma in situ of tongue
153386011	B811.00	Carcinoma in situ of trachea
153388012	B803100	Carcinoma in situ of transverse colon
153410011	B833200	Carcinoma in situ of vagina
153422014	B833300	Carcinoma in situ of vulva
154082012	B625200	Letterer-Siwe disease of intrathoracic lymph nodes
154089015	B625800	Letterer-Siwe disease of lymph nodes of multiple sites

154183017	B623300	Malignant histiocytosis of intra-abdominal lymph nodes
154184011	B623600	Malignant histiocytosis of intrapelvic lymph nodes
154185012	B623200	Malignant histiocytosis of intrathoracic lymph nodes
154192019	B623800	Malignant histiocytosis of lymph nodes of multiple sites
154193012	B623700	Malignant histiocytosis of spleen
154231019	B325400	Malignant melanoma of perianal skin
155060012	B32..00	Malignant melanoma of skin
155068017	B304100	Malignant neoplasm of acromion
155072018	B540.00	Malignant neoplasm of adrenal gland
155076015	B142.00	Malignant tumour of anal canal
155078019	B242.00	Malignant neoplasm of anterior mediastinum
155085015	B545100	Malignant neoplasm of aortic body
155089014	B340100	Malignant neoplasm of areola of female breast
155090017	B350100	Malignant neoplasm of areola of male breast
155155010	B442.00	Malignant neoplasm of broad ligament
155168019	B544.00	Malignant neoplasm of carotid body
155175018	B516.00	Malignant neoplasm of cerebellum
155191015	B303200	Malignant neoplasm of clavicle
155193017	B545200	Malignant neoplasm of coccygeal body
155206014	B308600	Malignant neoplasm of cuboid
155207017	B161000	Malignant neoplasm of cystic duct
155209019	B313100	Malignant neoplasm of diaphragm

1552110<br>11	B01<br>1.00	Malignant neoplasm of dorsal surface of tongue
1552180<br>17	B41<br>0.00	Malignant neoplasm of endocervix
1552220<br>10	B24<br>110<br>0	Malignant neoplasm of epicardium
1552280<br>14	B30<br>000<br>0	Malignant neoplasm of ethmoid bone
1552310<br>10	B41<br>1.00	Malignant neoplasm of exocervix
1552460<br>15	B30<br>700<br>0	Malignant neoplasm of femur
1552470<br>12	B30<br>710<br>0	Malignant neoplasm of fibula
1552590<br>11	B30<br>010<br>0	Malignant neoplasm of frontal bone
1552690<br>17	B54<br>500<br>0	Malignant neoplasm of glomus jugulare
1552720<br>12	B31<br>320<br>0	Malignant neoplasm of great vessels
1552870<br>19	B22<br>110<br>0	Malignant neoplasm of hilus of lung
1552940<br>16	B30<br>600<br>0	Malignant neoplasm of ilium
1553050<br>11	B15<br>1.00	Malignant neoplasm of intrahepatic bile ducts
1553080<br>13	B30<br>610<br>0	Malignant neoplasm of ischium
1553320<br>18	B06<br>6.00	Malignant neoplasm of lateral wall of oropharynx
1553610<br>17	B22<br>1.00	Malignant neoplasm of main bronchus
1553640<br>13	B35<br>..00	Malignant neoplasm of male breast
1553680<br>11	B30<br>1.00	Malignant neoplasm of mandible
1553700<br>19	B30<br>0A0<br>0	Malignant neoplasm of maxilla
1554020<br>13	B30<br>030<br>0	Malignant neoplasm of nasal bone
1554170<br>12	B34<br>000<br>0	Malignant neoplasm of nipple of female breast
1554180<br>19	B35<br>000<br>0	Malignant neoplasm of nipple of male breast

155421017	B300400	Malignant neoplasm of occipital bone
155425014	B520100	Malignant neoplasm of optic nerve
155441010	B443.00	Malignant neoplasm of parametrium
155444019	B300600	Malignant neoplasm of parietal bone
155446017	B18y400	Malignant neoplasm of parietal peritoneum
155447014	B230.00	Malignant neoplasm of parietal pleura
155449012	B308000	Malignant neoplasm of patella
155452016	B18y500	Malignant neoplasm of pelvic peritoneum
155457010	B180000	Malignant neoplasm of periadrenal tissue
155458017	B335900	Malignant neoplasm of perianal skin
155459013	B241300	Malignant neoplasm of pericardium
155468010	B42..00	Primary malignant neoplasm of placenta
155473016	B243.00	Malignant neoplasm of posterior mediastinum
155477015	B553100	Malignant neoplasm of presacral region
155479017	B306200	Malignant neoplasm of pubis
155483017	B304300	Malignant neoplasm of radius
155488014	B141.00	Malignant tumour of rectum
155495017	B303000	Malignant neoplasm of rib
155501015	B553200	Malignant neoplasm of sacrococcygeal region
155503017	B304000	Malignant neoplasm of scapula
155517014	B337500	Malignant neoplasm of skin of ankle

1555200<br>18	B33<br>570<br>0	Malignant neoplasm of skin of back
1555210<br>19	B33<br>520<br>0	Malignant neoplasm of skin of breast
1555220<br>14	B33<br>580<br>0	Malignant neoplasm of skin of buttock
1555250<br>11	B33<br>310<br>0	Malignant neoplasm of skin of chin
1555290<br>17	B33<br>320<br>0	Malignant neoplasm of skin of eyebrow
1555330<br>12	B33<br>640<br>0	Malignant neoplasm of skin of finger
1555340<br>18	B33<br>770<br>0	Malignant neoplasm of skin of foot
1555360<br>16	B33<br>330<br>0	Malignant neoplasm of skin of forehead
1555370<br>13	B33<br>550<br>0	Malignant neoplasm of skin of groin
1555380<br>15	B33<br>630<br>0	Malignant neoplasm of skin of hand
1555390<br>11	B33<br>700<br>0	Malignant neoplasm of skin of hip
1555400<br>13	B33<br>720<br>0	Malignant neoplasm of skin of knee
1555420<br>17	B33<br>0.00	Malignant neoplasm of skin of lip
1555450<br>15	B33<br>410<br>0	Malignant neoplasm of skin of neck
1555470<br>11	B33<br>560<br>0	Malignant neoplasm of skin of perineum
1555500<br>14	B33<br>600<br>0	Malignant neoplasm of skin of shoulder
1555520<br>18	B33<br>710<br>0	Malignant neoplasm of skin of thigh
1555530<br>11	B33<br>780<br>0	Malignant neoplasm of skin of toe
1555560<br>15	B33<br>540<br>0	Malignant neoplasm of skin of umbilicus
1555820<br>12	B30<br>070<br>0	Malignant neoplasm of sphenoid bone

1555920 16	B30 310 0	Malignant neoplasm of sternum
1556040 19	B30 810 0	Malignant neoplasm of talus
1556070 14	B30 080 0	Malignant neoplasm of temporal bone
1556240 15	B30 720 0	Malignant neoplasm of tibia
1556410 18	B30 440 0	Malignant neoplasm of ulna
1556690 14	B01 3.00	Malignant neoplasm of ventral surface of tongue
1556750 17	B23 1.00	Malignant neoplasm of visceral pleura
1556770 13	B30 0C0 0	Malignant neoplasm of vomer
1556810 13	B30 090 0	Malignant neoplasm of zygomatic bone
1557110 12	B58 7.00	Secondary malignant neoplasm of adrenal gland
1557730 15	B58 110 0	Secondary malignant neoplasm of bladder
1558790 14	B58 300 0	Secondary malignant neoplasm of brain
1559670 13	B57 500 0	Secondary malignant neoplasm of colon
1560070 19	B57 400 0	Secondary malignant neoplasm of duodenum
1561690 17	B57 420 0	Secondary malignant neoplasm of ileum
1562151 0000061 11	9Nh 1.00	Under the care of cancer primary healthcare multidisciplinary team
1562310 17	B57 410 0	Secondary malignant neoplasm of jejunum
1562370 18	B58 0.00	Secondary malignant neoplasm of kidney
1562910 15	B57 7.00	Secondary malignant neoplasm of liver
1563190 16	B57 0.00	Secondary malignant neoplasm of lung
1563750 15	B57 1.00	Secondary malignant neoplasm of mediastinum
1564930 15	B58 6.00	Secondary malignant neoplasm of ovary

1565570<br>15	B58<br>y70<br>0	Secondary malignant neoplasm of penis
1565850<br>18	B57<br>2.00	Secondary malignant neoplasm of pleura
1566070<br>18	B58<br>y50<br>0	Secondary malignant neoplasm of prostate
1566270<br>17	B57<br>510<br>0	Secondary malignant neoplasm of rectum
1566990<br>10	B58<br>260<br>0	Secondary malignant neoplasm of skin of breast
1567190<br>18	B58<br>210<br>0	Secondary malignant neoplasm of skin of face
1567470<br>19	B58<br>220<br>0	Secondary malignant neoplasm of skin of neck
1567650<br>15	B58<br>230<br>0	Secondary malignant neoplasm of skin of trunk
1567770<br>13	B58<br>2.00	Secondary malignant neoplasm of skin
1568250<br>10	B58<br>310<br>0	Metastasis to spinal cord
1568830<br>18	B58<br>y60<br>0	Secondary malignant neoplasm of testis
1569190<br>12	B58<br>y90<br>0	Secondary malignant neoplasm of tongue
1569730<br>18	B58<br>100<br>0	Secondary malignant neoplasm of ureter
1569770<br>17	B58<br>120<br>0	Secondary malignant neoplasm of urethra
1569890<br>15	B58<br>y10<br>0	Secondary malignant neoplasm of uterus
1569970<br>10	B58<br>y30<br>0	Secondary malignant neoplasm of vagina
1570250<br>19	B58<br>y40<br>0	Secondary malignant neoplasm of vulva
1570710<br>14	B62<br>130<br>0	Mycosis fungoides of intra-abdominal lymph nodes
1570750<br>17	B62<br>140<br>0	Mycosis fungoides of lymph nodes of axilla and upper limb
1570910<br>19	B65<br>3.00	Myeloid sarcoma

1574810 0000611 1	B56 130 0	Secondary malignant neoplasm of mediastinal lymph nodes
1574910 0000611 4	B56 080 0	Secondary malignant neoplasm of anterior cervical lymph nodes
1575010 0000611 8	B56 3.00	Secondary malignant neoplasm of lymph nodes of upper limb
1575110 0000611 5	B56 3z0 0	Secondary and unspec malig neop axilla and upper limb LN NOS
1575210 0000611 1	B56 300 0	Secondary malignant neoplasm of axillary lymph nodes
1575310 0000611 4	B56 180 0	Secondary and unspec malig neop bronchopulmonary lymph nodes
1575410 0000611 6	B56 520 0	Secondary malignant neoplasm of circumflex iliac lymph nodes
1575510 0000611 9	B56 200 0	Secondary malignant neoplasm of coeliac lymph nodes
1575610 0000611 7	B56 230 0	Secondary malignant neoplasm of iliac lymph nodes
1575710 0000611 2	B56 090 0	Secondary malignant neoplasm of deep cervical lymph nodes
1575810 0000611 0	B56 410 0	Secondary malignant neoplasm of deep inguinal lymph nodes
1575910 0000611 3	B56 040 0	Secondary malignant neoplasm of parotid lymph nodes
1576010 0000611 7	B56 120 0	Secondary malignant neoplasm of diaphragmatic lymph nodes
1576110 0000611 9	B56 240 0	Secondary and unspec malig neop external iliac lymph nodes
1576201 0000061 11		Cause of Death- Malignant Neoplasms
1576210 0000611 0	B56 510 0	Secondary malignant neoplasm of inferior epigastric lymph nodes
1576251 0000061 10		Cause of Death- carcinoma of oesophagus
1576310 0000611 3	B56 220 0	Secondary malignant neoplasm of mesenteric lymph nodes
1576410 0000611 5	B56 170 0	Secondary malignant neoplasm of tracheobronchial lymph nodes

15765100000611 8	B56 320 0	Secondary malignant neoplasm of infraclavicular lymph nodes
15766100000611 6	B56 4.00	Secondary and unspec malig neop inguinal and lower limb LN
15767100000611 1	B56 110 0	Secondary malignant neoplasm of intercostal lymph nodes
15768100000611 4	B56 500 0	Secondary malignant neoplasm of internal iliac lymph nodes
1576860 16	B62 030 0	Nodular lymphoma of intra-abdominal lymph nodes
15769100000611 2	B56 100 0	Secondary malignant neoplasm of internal mammary lymph nodes
1576920 10	B62 080 0	Nodular lymphoma of lymph nodes of multiple sites
15770100000611 2	B56 2z0 0	Secondary and unspec malig neop intra-abdominal LN NOS
15771100000611 0	B56 2.00	Secondary and unspec malig neop intra-abdominal lymph nodes
15772100000611 9	B56 5z0 0	Secondary and unspec malig neop intrapelvic LN NOS
15773100000611 6	B56 5.00	Secondary and unspec malig neop intrapelvic lymph nodes
1577320 17	B15 0.00	Primary malignant neoplasm of liver
15774100000611 4	B56 1z0 0	Secondary and unspec malig neop intrathoracic LN NOS
1577450 18	B60 030 0	Reticulosarcoma of intra-abdominal lymph nodes
15775100000611 1	B56 1.00	Secondary and unspec malig neop intrathoracic lymph nodes
1577520 16	B60 070 0	Reticulosarcoma of spleen
15776100000611 3	B56 0.00	Metastasis to head and neck lymph node
15777100000611 8	B56 y.00	Secondary and unspec malig neop lymph nodes multiple sites
15778100000611 5	B56 z.00	Secondary and unspec malig neop lymph nodes NOS
15779100000611 7	B56 540 0	Secondary malignant neoplasm of obturator lymph nodes

15780100000611 6	B56 060 0	Secondary malignant neoplasm of lymph nodes of face
15781100000611 8	B56 4z0 0	Secondary and unspec malig neop of inguinal and leg LN NOS
15782100000611 4	B56 000 0	Secondary malignant neoplasm of parotid lymph nodes
15783100000611 2	B56 150 0	Secondary and unspec malig neop paratracheal lymph nodes
15784100000611 9	B56 330 0	Secondary malignant neoplasm of pectoral axillary lymph nodes
15785100000611 7	B56 420 0	Secondary malignant neoplasm of popliteal lymph nodes
15786100000611 5	B56 140 0	Secondary and unspec malig neop post mediastinal lymph nodes
15787100000611 0	B56 190 0	Secondary malignant neoplasm of bronchopulmonary lymph nodes
15788100000611 3	B56 530 0	Secondary malignant neoplasm of sacral lymph nodes
15789100000611 1	B56 050 0	Secondary malignant neoplasm of submandibular lymph nodes
15790100000611 0	B56 070 0	Secondary malignant neoplasm of submental lymph nodes
15791100000611 3	B56 160 0	Secondary malignant neoplasm of tracheobronchial lymph nodes
15792100000611 7	B56 020 0	Secondary malignant neoplasm of superficial cervical lymph nodes
15793100000611 9	B56 400 0	Secondary malignant neoplasm of superficial inguinal lymph nodes
15794100000611 2	B56 210 0	Secondary malignant neoplasm of mesenteric lymph nodes
15795100000611 4	B56 310 0	Secondary malignant neoplasm of supratrochlear lymph nodes
15796100000611 1	B56 010 0	Secondary malignant neoplasm of mastoid lymph nodes
15797100000611 6	B56 030 0	Secondary malignant neoplasm of occipital lymph nodes
1591211000006111	B62 4.12	Hairy cell leukaemia

166810100000001 12	B627A0 0	Diffuse non-Hodgkin's large cell lymphoma
169477100000061 18		Malignant melanoma of the cilary body
169478100000061 15		Malignant melanoma of choroid
169789100000061 16		Malignant melanoma of iris
171746100000001 12	142700 0	H/O: prostate cancer
172816100000061 16		Hereditary nonpolyposis colon cancer
173816012	B32y00 0	Overlapping malignant melanoma of skin
173941016	B24140 0	Mesothelioma of pericardium
173946014	B05z00 0	Kaposi's sarcoma of palate
173950019	B6z0.00	Kaposi's sarcoma of lymph nodes
174518012	B14200 0	Malignant neoplasm of cloacogenic zone
174532014	B181.00	Mesothelioma of peritoneum
174562015	B471.00	Malignant neoplasm of descended testis
174576019	B83000 0	Lobular carcinoma in situ of breast
174577011	B83010 0	Intraductal carcinoma in situ of breast
175580100000061 18	BB4B.0 0	[M]Grade 1 (Stage pTa) papillary urothelial/transitional cell carcinoma
175581100000061 15	BB4C.00	[M]Grade 2 (Stage pTa) papillary urothelial/transitional cell carcinoma
175582100000061 11	BB4D.0 0	[M]Grade 3 (Stage pTa) papillary urothelial/transitional cell carcinoma
177048100000061 18	BB3C.00	Multifocal superficial basal cell carcinoma
177049100000061 15	BB3D.0 0	Basal cell carcinoma, nodular

1770501 0000061 11	BB3 E.00	Basal cell carcinoma - micronodular
1770510 0000611 1	B4A ..11	Malignant tumour of kidney
1770511 0000061 14	BB3 F.00	Infiltrating basal cell carcinoma
1770521 0000061 18	BB3 G.0 0	Pigmented basal cell carcinoma
1773111 0000061 11		Primary malignant neoplasm of lung
1773121 0000061 15		Small cell lung cancer
1773131 0000061 17		Non-small cell lung cancer
1773141 0000061 10		Squamous cell carcinoma of lung
1773151 0000061 12		Adenocarcinoma of lung
1773161 0000061 14		Large cell carcinoma of lung
1776481 0000061 16		Reason for referral: Cancer
1785811 0000061 14	BBP 3.11	Fibrous mesothelioma, malignant
1786811 017	B49 ..00	Malignant neoplasm of urinary bladder
1803771 0000061 12		Local recurrence of malignant tumour of urinary bladder
1803781 0000061 10		Local recurrence of malignant tumour of breast
1806421 0000061 10		Malignant neoplasm of skin
1808791 0000061 16		Oral carcinoma risk assessment
1815011 0000061 16	B57 700 0	Secondary malignant neoplasm of liver and intrahepatic bile duct
1815031 0000061 10	B61 ..11	Hodgkin lymphoma
1815091 0000061 14	B61 C.00	Other classical Hodgkin lymphoma

1815151000006116	B628.00	Follicular lymphoma
1815461000006110	B62F000	Low grade B-cell lymphoma
1815691000006116	B677.00	Myelodysplastic/myeloproliferative disease
18484012	B630400	Plasmacytoma - disorder
1859381000006117		Wells DVT score - active cancer
1870381000006113	BB1P.00	Non-small cell carcinoma
1877231000006117		Cancer and/or significant invasive tumors
1884191000006113	4K2M.00	Cervical smear - high grade dyskaryosis with features of invasive squamous carcinoma
1884911000006118	68W2500	Bowel scope (flexible sigmoidoscopy) screen: suspected cancer detected
1887410000006119	6864.12	Rectal neoplasm screen
1937251000006115		History of malignant tumour of pelvis
1937971000006115		Delayed diagnosis of cancer
1959991000006111		[M]Metaplastic carcinoma
1960021000006111		[M]Porocarcinoma
1962621000006116		First definitive treatment for a new primary cancer
1962641000006111		Second or subsequent treatment for a new primary cancer
1963411000006119		Treatment for a local recurrence of a primary cancer
1963431000006113		Treatment for a regional recurrence of cancer
1963441000006115		Treatment for a distant recurrence of cancer (metastatic disease)

1963941 0000061 10		Treatment for relapse of primary cancer (second or subsequent)
1963961 0000061 14		Treatment for progression of primary cancer (second or subsequent)
1963971 0000061 19		Treatment for multiple recurrence of cancer (local and/or regional and/or distant)
1980060 10	B62 7E0 0	Diffuse large B-cell lymphoma
1985161 0000061 13		History of malignant neoplasm of vulva
1985171 0000061 18		History of malignant neoplasm of cervix
1985181 0000061 15		History of malignant neoplasm of uterine body
1985191 0000061 17		History of malignant neoplasm of ovary
1992241 0000061 11		VTE risk assessment - active cancer or cancer treatment
1998881 0000061 19		High risk basal cell carcinoma
2016120 10	B65 0.00	Acute myeloid leukaemia
2016380 10	B80 340 0	Carcinoma in situ of caecum
2016400 17	B80 1.00	Carcinoma in situ of oesophagus
2016520 12	B64 1.00	Chronic lymphoid leukaemia
2016560 10	B65 1.00	Chronic myeloid leukaemia
2016580 11	B67 0.11	Di Guglielmo's disease
2017430 12	B63 1.00	Plasma cell leukaemia
2028010 0000611 0	B64 y10 0	Prolymphocytic leukaemia
2076110 12		[M]Neuroendocrine neoplasm
2157510 0000011 6	B33 ..11	Basal cell carcinoma of skin
2157610 0000011 8	BB2 ..12	Squamous cell neoplasm
2158920 12	BBH z.00	Myxomatous neoplasm

215897018	BBL..00	[M]Complex mixed and stromal neoplasms
215912012	BBgM.00	[M]Malignant lymphoma, small cleaved cell, diffuse
215915014	BBm1.00	Malignant histiocytosis
215916010	BBm1.11	[M]Malignant reticulosis
216227011	B585000	Pathological fracture due to metastatic bone disease
2164653019	B30z000	Osteosarcoma
2168691000000110	B62F200	Lymphoblastic (diffuse) lymphoma
2191810000006119	4D56.00	Pleural fluid: malignant cells present
2250510000006119	B630200	Plasmacytoma
2280371000000117	68W2400	Bowel scope (flexible sigmoidoscopy) screen: cancer detected
2438511000000112	B328.00	Malignant melanoma stage IA
2438551000000111	B329.00	Malignant melanoma stage IB
2438591000000115	B32A.00	Malignant melanoma stage IIA
2438631000000115	B32B.00	Malignant melanoma stage IIB
2438671000000118	B32C.00	Malignant melanoma stage IIC
2438711000000117	B32D.00	Malignant melanoma stage IIIA
2438751000000118	B32E.00	Malignant melanoma stage IIIB
2438791000000114	B32F.00	Malignant melanoma stage IIIC
2438871000000117	B32G.00	Malignant melanoma stage IV M1a
2438911000000115	B32H.00	Malignant melanoma stage IV M1b

2438951000000116	B32J.00	Malignant melanoma stage IV M1c
2460981000000118	8AD0.00	Active surveillance of prostate cancer
24709100000006118	B182.00	Overlapping malignant lesion of retroperitoneum and peritoneum
2475562018	B151100	Malignant neoplasm of interlobular biliary canals
2475564017	B300B00	Malignant neoplasm of turbinate
2475984012	B062200	Malignant neoplasm of palatoglossal arch
2504131000006112		Malignant peripheral nerve sheath tumour with rhabdomyoblastic differentiation
2504141000006119		Triton tumor, malignant
2504151000006117		Triton tumour, malignant
2504161000006115		Malignant schwannoma with rhabdomyoblastic differentiation
2504181000006113		Malignant peripheral nerve sheath tumor with rhabdomyoblastic differentiation
2512951000006110		Mucin-producing adenocarcinoma
2512961000006112		Mucin-producing carcinoma
2512971000006117		Mucin-secreting adenocarcinoma
2512981000006119		Mucin-secreting carcinoma
2513181000006113		Malignant melanoma in junctional naevus
2513191000006111		Malignant melanoma in junctional nevus
2514281000006119		Mixed islet cell and exocrine adenocarcinoma
2514291000006116		Mixed ductal-endocrine carcinoma

2514301000006115		Mixed acinar-endocrine carcinoma
251569011	1425000	H/O Malignant melanoma
251747019	14CB.00	H/O upper GIT neoplasm
251748012	14CC.00	H/O lower GIT neoplasm
2519751000006116		Pigmented basal cell carcinoma
2519781000006112		BCC - Basal cell carcinoma
2521381000006119		Chromophobe adenocarcinoma
2529491000006110		Malignant lymphoma, non-Hodgkin
2529501000006119		Malignant lymphoma, non-Hodgkin's
2529561000006118		Malignant lymphoma, undifferentiated cell, non-Burkitt [obs]
2529571000006113		Malignant lymphoma, undifferentiated cell type [obs]
2529581000006111		Malignant lymphoma, small cleaved cell, diffuse [obs]
2529591000006114		Malignant lymphoma, small cleaved cell [obs]
2529601000006118		Malignant lymphoma, small cell, noncleaved, diffuse [obs]
2529611000006115		Malignant lymphoma, lymphocytic, poorly differentiated, diffuse [obs]
2529621000006111		Malignant lymphoma, lymphocytic, intermediate differentiation, nodular [obs]
2529631000006114		Malignant lymphoma, diffuse
2529641000006116		Malignant lymphoma, cleaved cell [obs]
2532031000006112		MM - Malignant melanoma
2532041000006119		Malignant melanoma, no ICD-O subtype

2532051000006117		Malignant melanoma, no International Classification of Diseases for Oncology subtype
2534102018	9Ok5.00	Cancer pain and symptom management
2534331000006115		Mesonephroma, malignant
2534341000006113		Mesonephric adenocarcinoma
2534351000006110		Wolffian duct carcinoma
2534415018	B660.00	Acute monocytic leukaemia
2534461000006118		Adrenal cortical tumor, malignant
2534471000006113		Adrenal cortical adenocarcinoma
2534481000006111		Adrenal cortical tumour, malignant
2537051000006119		Malignant pyoderma
2537601000006110		Soft tissue tumor, malignant
2537611000006113		Mesenchymal tumor, malignant
2537631000006119		Soft tissue tumour, malignant
2537641000006112		Mesenchymal tumour, malignant
2537651000006114		Malignant mesenchymal tumour
2537661000006111		Malignant mesenchymal tumor
2542411000006113		Papillocystic adenocarcinoma
2546741000006119		Paget's carcinoma of the nipple
2549374014	B306500	Malignant sacral teratoma
2560241000006117		Lipid-rich carcinoma

2564551000006111		Parenteral chemotherapy for malignant neoplasm
2568751000006113		Malignant mixed tumour, osteosarcomatous type
2568761000006110		Malignant mixed tumor, osteosarcomatous type
2572011000006116		Scirrhous adenocarcinoma
2572021000006112		Scirrhous carcinoma
2572031000006110		Carcinoma with productive fibrosis
2572131000006111		Metastatic adenocarcinoma
2572791000006111		Tubular carcinoma
2578161000006117		Adenocarcinoma in situ in villous adenoma
2580121000006117		Merkel cell carcinoma
2580141000006112		Primary cutaneous neuroendocrine carcinoma
2583521000006115		Follicular carcinoma
2588811000006118		Schneiderian carcinoma
2588821000006114		Cylindrical cell carcinoma
2589231000006118		Excision of malignant lesion of skin of extremities
2589241000006111		Excision of malignant neoplastic lesion of skin of extremities
2589791000006112		Adenocarcinoma in tubulovillous adenoma
2589801000006113		Tubulopapillary adenocarcinoma
2589811000006111		Papillotubular adenocarcinoma

2590251000006117		Squamous cell carcinoma in situ with questionable stromal invasion
2590261000006115		Epidermoid carcinoma in situ with questionable stromal invasion
2594581000006117		Small cell carcinoma, intermediate cell
2599121000006113		Unclassified tumor, malignant, uncertain whether primary or metastatic
2599131000006111		Unclassified tumour, malignant, uncertain whether primary or metastatic
2599151000006116		Malignant tumor - uncertain whether primary or metastatic
2606061000006118		Metatypical carcinoma
2609581000006113		Perfusion chemotherapy for malignant neoplasm
260962019	4E33.00	Sputum: malignant cells
260992011	4F32.00	Ascitic fluid: malignant cells
2613951000000118		Lymphoedema following cancer
2614621000000117		Lymphoedema following breast cancer
2616201000006111		Lymphoepithelial carcinoma
2616241000006113		Lymphoepithelioma-like carcinoma
2619456011	B151200	Malignant neoplasm of intrahepatic biliary passages
2629941000006115		Mixed tumour, malignant
2629951000006118		Mixed tumor, malignant
2629961000006116		Mixed tumor, salivary gland type, malignant
2629971000006111		Mixed tumour, salivary gland type, malignant
2629981000006114		Malignant chondroid syringoma

2629991 0000061 12		Malignant mixed tumour
2630001 0000061 11		Malignant mixed salivary gland tumour
2630011 0000061 14		Malignant mixed salivary gland tumor
2630021 0000061 18		Malignant mixed tumor
2639401 0000061 10		Choriocarcinoma combined with other germ cell elements
2639411 0000061 13		Choriocarcinoma combined with teratoma
2639421 0000061 17		Choriocarcinoma combined with embryonal carcinoma
2639431 0000061 19		Choriocarcinoma combined with germ cell elements
2653191 0000061 12		Epithelial-myoepithelial carcinoma
2657681 0000061 14		Paraganglioma, malignant
2659756 011	B62 x20 0	Peripheral T-cell lymphoma
2659784 018	B8.. .00	Carcinoma in situ
2660421 0000061 17		Giant cell tumour of bone, malignant
2660431 0000061 19		Giant cell tumor of bone, malignant
2660441 0000061 12		Osteoclastoma, malignant
2660471 0000061 16		Malignant giant cell tumor of bone
2660481 0000061 18		Malignant osteoclastoma
2661001 0000061 10		Malignant glaucoma
2661631 0000061 14		Chief cell adenocarcinoma
2661641 0000061 16		Chief cell carcinoma

2663187010	B616.00	Hodgkin's disease, lymphocytic depletion
2663190016	B602.00	Burkitt's lymphoma
2663191017	B621.00	Mycosis fungoides
2663376010	B612.00	Hodgkin's sarcoma
2663461018	B615.00	Hodgkin's disease, mixed cellularity
2663473018	B61..00	Hodgkin's disease
2663476014	B611.00	Hodgkin's granuloma
266363100000613		Squamous cell carcinoma, spindle cell
266364100000615		Epidermoid carcinoma, spindle cell
266365100000618		Squamous cell carcinoma, sarcomatoid
266366100000616		Epidermoid carcinoma - spindle cell
266367100000611		Squamous cell carcinoma - spindle cell
268578100000616		Adenocarcinoma, cylindroid
268579100000618		Adenocystic carcinoma
2692069010	B51..00	Malignant neoplasm of brain
269614100000615		Androblastoma, malignant
269615100000618		Arrhenoblastoma, malignant
269616100000616		Malignant androblastoma
269617100000611		Malignant arrhenoblastoma
269740100000614		Papillary urothelial carcinoma
269861100000610		Squamous cell carcinoma, microinvasive
270082100000613		Melanoma, malignant, of soft parts

2710081000006118		Granular cell tumour, malignant
2710091000006115		Granular cell tumor, malignant
2710101000006114		Granular cell myoblastoma, malignant
2710111000006112		Malignant granular cell tumour
2710121000006116		Malignant granular cell tumor
2710131000006118		Malignant granular cell myoblastoma
2715231000006115		Malignant mast cell tumor
2715241000006113		Malignant mastocytoma
2715251000006110		Malignant mast cell tumour
2730251000006111		Malignant lymphoma, Hodgkin's
2731021000000110		Secondary malignant neoplasm of paratracheal lymph nodes
2731321000000112		Malignant neoplasm of respiratory tract
2731421000000116		Malignant neoplasm of genital labia
2732801000000115		Malignant neoplasm of soft tissues of lower limb
2732861000000116		Malignant neoplasm of soft tissues of lower leg
2736711000006115		International Federation of Gynecology and Obstetrics vulvar carcinoma (FIGO VC) stage II
2736721000006111		International Federation of Gynaecology and Obstetrics vulvar carcinoma (FIGO VC) stage II
2736731000006114		International Federation of Gynecology and Obstetrics vulvar carcinoma stage II
2741201000000113		Malignant neoplasm of soft tissues of thigh

2746531000006115		International Federation of Gynecology and Obstetrics cervical cancer (FIGO CC) stage I
2746541000006113		International Federation of Gynecology and Obstetrics cervical cancer stage I
2746551000006110		International Federation of Gynaecology and Obstetrics cervical cancer (FIGO CC) stage I
2747671000006116		Hepatocellular carcinoma, fibrolamellar
2748631000006115		Primary serous papillary carcinoma of peritoneum
2753081000006116		Thymoma, malignant
2767031000006117		Malignant carotid body tumour
2767041000006110		Malignant carotid body tumor
2769471000006119		Acral lentiginous melanoma, malignant
2773871000006111		Carcinoma ex pleomorphic adenoma
2773881000006114		Carcinoma in pleomorphic adenoma
2786351000006110		Squamous cell carcinoma, keratinising
2786361000006112		Squamous cell carcinoma, keratinizing
2786371000006117		Squamous cell carcinoma, large cell, keratinizing
2786381000006119		Epidermoid carcinoma, keratinizing
2786391000006116		Epidermoid carcinoma, keratinising
2786401000006119		Squamous cell carcinoma, large cell, keratinising
2786421000006112		Keratinising squamous cell carcinoma - large cell
2786431000006110		Keratinising epidermoid carcinoma

2786441 0000061 17		Keratinizing squamous cell carcinoma - large cell
2786451 0000061 15		Keratinizing epidermoid carcinoma
2787491 0000061 11		Endometrioid adenofibroma, malignant
2787501 0000061 15		Endometrioid cystadenofibroma, malignant
2787511 0000061 17		Malignant endometrioid cystadenofibroma
2787521 0000061 13		Malignant endometrioid adenofibroma
2792481 0000061 14		Laparotomy for staging of ovarian cancer
2792491 0000061 12		Staging celiotomy for ovarian cancer
2792501 0000061 16		Staging coeliotomy for ovarian cancer
2793071 0000061 11		Malignant melanoma in precancerous melanosis
2793081 0000061 14		Malignant melanoma in melanosis
2796431 0000061 14		Intraductal carcinoma and lobular carcinoma in situ
2798331 0000061 15		Malignant hydatidiform mole
2799261 0000061 10		Struma ovarii, malignant
2799271 0000061 15		Malignant struma ovarii
2799391 0000061 13		Granulosa cell tumour, malignant
2799401 0000061 10		Granulosa cell tumor, malignant
2799411 0000061 13		Granulosa cell carcinoma
2799451 0000061 14		Malignant granulosa cell tumor

2806901000006114		Malignant lymphoma, lymphoplasmacytic
2806911000006112		Malignant lymphoma, lymphoplasmacytoid
2806931000006118		Malignant lymphoma, plasmacytoid
2808981000006118		Teratoma, malignant
2809001000006115		Teratoblastoma, malignant
2809021000006113		Immature teratoma, malignant
2809041000006118		Malignant teratoblastoma
2809051000006116		Teratoma, malignant, no ICD-O subtype
2809061000006119		Teratoma, malignant, no International Classification of Diseases for Oncology subtype
2813741000006119		Gastrinoma, malignant
2813751000006117		G cell tumor, malignant
2813761000006115		Gastrin cell tumor, malignant
2813771000006110		G cell tumour, malignant
2813781000006113		Gastrin cell tumour, malignant
2813791000006111		Malignant G cell tumour
2813801000006112		Malignant G cell tumor
2815771000006111		Malignant peripheral nerve sheath tumour
2815851000006112		Malignant peripheral nerve sheath tumor
2815861000006114		MPNST - Malignant peripheral nerve sheath tumour

2815871 0000061 19		MPNST - Malignant peripheral nerve sheath tumor
2833391 0000061 17		Insulinoma, malignant
2833401 0000061 15		Beta cell tumor, malignant
2833411 0000061 17		Beta cell tumour, malignant
2833421 0000061 13		Malignant beta cell tumour
2833431 0000061 11		Malignant beta cell tumor
2839071 0000061 18		Combined small cell carcinoma
2839081 0000061 15		Small cell-large cell carcinoma
2839091 0000061 17		Mixed small cell carcinoma
2839101 0000061 11		Combined small cell-squamous cell carcinoma
2839111 0000061 14		Combined small cell-adenocarcinoma
2839871 014	B62 7.11	Non-Hodgkin lymphoma
2843491 0000061 14		Malignant
2848541 0000061 10		Malignant teratoma, intermediate
2848551 0000061 12		Malignant teratoma - intermediate
2849401 0000061 16		Malignant lymphoma, no ICD-O subtype
2849411 0000061 18		Malignant lymphoma, no International Classification of Diseases for Oncology subtype
2849461 0000061 15		Papillary carcinoma, follicular variant
2849471 0000061 10		Papillary and follicular adenocarcinoma
2849481 0000061 13		Papillary and follicular carcinoma

2849491 0000061 11		Papillary adenocarcinoma, follicular variant
2849501 0000061 15		Papillary adenocarcinoma - follicular variant
2849511 0000061 17		Papillary carcinoma - follicular variant
2856431 0000061 19		International Federation of Gynaecology and Obstetrics vulvar carcinoma (FIGO VC) stage III
2856441 0000061 12		International Federation of Gynecology and Obstetrics vulvar carcinoma (FIGO VC) stage III
2856451 0000061 14		International Federation of Gynecology and Obstetrics vulvar carcinoma stage III
2861541 0000061 10		Adenocarcinoma with apocrine metaplasia
2861551 0000061 12		Carcinoma with apocrine metaplasia
2868371 0000061 18		Pseudosarcomatous carcinoma
2868381 0000061 15		Sarcomatoid carcinoma
2870140 15	A78 860 0	Malignant neoplastic disease co-occurrent with human immunodeficiency virus infection
2870310 10	A78 980 0	HIV disease resulting in multiple malignant neoplasms
2874101 0000061 10		Mixed acidophil-basophil carcinoma
2881850 10	Ayu C80 0	Malignant neoplastic disease co-occurrent with human immunodeficiency virus infection
2881860 11	Ayu C90 0	[X]HIV disease resulting in unspecified malignant neoplasm
2883500 19	B00 010 0	Malignant neoplasm of upper lip, lipstick area
2883510 15	B00 0z0 0	Malignant tumour of vermilion border of upper lip
2883540 11	B00 100 0	Malignant neoplasm of lower lip, external
2883550 12	B00 1z0 0	Malignant tumour of vermilion border of lower lip

2883581 0000061 15		Malignant catarrhal fever
2883591 0000061 17		Malignant head catarrh
2883620 15	B00 230 0	Malignant neoplasm of upper lip, oral aspect
2883630 13	B00 2.00	Malignant neoplasm of upper lip, inner aspect
2883640 19	B00 220 0	Malignant neoplasm of upper lip, mucosa
2883720 17	B00 210 0	Malignant neoplasm of upper lip, frenulum
2883770 11	B00 2z0 0	Malignant neoplasm of upper lip, inner aspect NOS
2883800 12	B00 300 0	Malignant neoplasm of lower lip, buccal aspect
2883820 16	B00 310 0	Malignant neoplasm of lower lip, frenulum
2883870 10	B00 3z0 0	Malignant neoplasm of lower lip, inner aspect NOS
2883880 17	B00 4.00	Malignant tumour of labial mucosa
2883900 16	B00 410 0	Malignant neoplasm of lip unspecified, frenulum
2883910 17	B00 420 0	Malignant neoplasm of lip unspecified, mucosa
2883930 19	B00 430 0	Malignant neoplasm of lip, oral aspect
2884030 12	B00 4z0 0	Malignant neoplasm of lip, inner aspect NOS
2884040 18	B00 6.00	Malignant neoplasm of overlapping lesion of lip
2884060 16	B00 y.00	Malignant neoplasm of other sites of lip
2884070 13	B00 z.00	Malignant neoplasm of vermilion border of lip unspecified
2884080 15	B00 z00 0	Malignant neoplasm of lip, unspecified, external
2884090 11	B00 z10 0	Malignant tumour of lipstick area of lip
2884100 18	B00 zz00	Malignant neoplasm of lip, vermilion border NOS

288411019	B010000	Malignant neoplasm of base of tongue dorsal surface
288412014	B010z00	Malignant neoplasm of fixed part of tongue NOS
288414010	B011000	Malignant neoplasm of anterior 2/3 of tongue dorsal surface
288417015	B011100	Malignant neoplasm of midline of tongue
288418013	B011z00	Malignant neoplasm of dorsum of tongue NOS
288419017	B012.00	Malignant neoplasm of tongue, tip and lateral border
288423013	B013000	Malignant neoplasm of anterior 2/3 of tongue ventral surface
288427014	B013100	Malignant neoplasm of frenulum linguae
288429012	B013z00	Malignant neoplasm of ventral surface of tongue
288430019	B014.00	Malignant neoplasm of anterior 2/3 of tongue unspecified
288433017	B015.00	Malignant neoplasm of tongue, junctional zone
288434011	B01y.00	Malignant neoplasm of other sites of tongue
288435012	B01z.00	Malignant neoplasm of tongue NOS
288437016	B02y.00	Malignant neoplasm of other major salivary glands
288438014	B02z.00	Malignant tumour of major salivary gland
288439018	B03y.00	Malignant neoplasm of other sites of gum
288440016	B03z.00	Malignant neoplasm of gum NOS
288443019	B040.00	Malignant neoplasm of anterior portion of floor of mouth
288444013	B041.00	Malignant neoplasm of lateral portion of floor of mouth
288448011	B04y.00	Malignant neoplasm of other sites of floor of mouth
288449015	B04z.00	Malignant tumour of floor of mouth
288450015	B05..00	Malignant neoplasm of other and unspecified parts of mouth
288453018	B051.00	Malignant neoplasm of vestibule of mouth
288454012	B051000	Malignant neoplasm of upper buccal sulcus

2884590<br>19	B05<br>110<br>0	Malignant neoplasm of lower buccal sulcus
2884600<br>12	B05<br>120<br>0	Malignant neoplasm of upper labial sulcus
2884650<br>19	B05<br>130<br>0	Malignant neoplasm of lower labial sulcus
2884680<br>17	B05<br>1z0<br>0	Malignant tumour of vestibule of mouth
2884690<br>13	B05<br>5.00	Malignant tumour of palate
2884700<br>14	B05<br>500<br>0	Malignant neoplasm of junction of hard and soft palate
2884710<br>13	B05<br>5z0<br>0	Malignant neoplasm of palate NOS
2884720<br>18	B05<br>7.00	Overlapping malignant neoplasm of mouth
2884730<br>11	B05<br>y.00	Malignant neoplasm of other specified mouth parts
2884740<br>17	B05<br>z.00	Malignant neoplasm of mouth NOS
2884780<br>19	B06<br>0z0<br>0	Malignant neoplasm tonsil NOS
2884850<br>15	B06<br>200<br>0	Malignant neoplasm of faucial pillar
2884860<br>19	B06<br>2.00	Malignant neoplasm of tonsillar pillar
2885050<br>15	B06<br>2z0<br>0	Malignant tumour of tonsillar fossa
2885060<br>19	B06<br>4.00	Malignant neoplasm of anterior epiglottis
2885070<br>11	B06<br>400<br>0	Malignant neoplasm of epiglottis, free border
2885080<br>18	B06<br>410<br>0	Malignant neoplasm of glossoepiglottic fold
2885090<br>14	B06<br>4z0<br>0	Malignant neoplasm of anterior epiglottis NOS
2885100<br>16	B06<br>5.00	Malignant neoplasm of junctional region of epiglottis
2885150<br>14	B06<br>7.00	Malignant neoplasm of posterior wall of oropharynx
2885160<br>10	B06<br>y.00	Malignant neoplasm of oropharynx, other specified sites
2885170<br>18	B06<br>yz0<br>0	Malignant neoplasm of other specified site of oropharynx NOS
2885180<br>11	B06<br>z.00	Malignant neoplasm of oropharynx NOS

288522018	B07..00	Malignant neoplasm of nasopharynx
288529010	B071.00	Malignant neoplasm of posterior wall of nasopharynx
288532013	B071100	Malignant neoplasm of pharyngeal tonsil
288533015	B071000	Malignant neoplasm of adenoid
288541015	B071z00	Malignant tumour of posterior wall of nasopharynx
288546013	B072000	Malignant neoplasm of pharyngeal recess
288548014	B072100	Malignant neoplasm of opening of auditory tube
288550018	B072z00	Malignant tumour of lateral wall of nasopharynx
288552014	B073.00	Malignant neoplasm of anterior wall of nasopharynx
288554010	B073000	Malignant neoplasm of floor of nasopharynx
288556012	B073100	Malignant neoplasm of nasopharyngeal soft palate surface
288561014	B073z00	Malignant tumour of anterior wall of nasopharynx
288563012	B07y.00	Malignant neoplasm of other specified site of nasopharynx
288564018	B07z.00	Malignant tumour of nasopharynx
288568015	B083.00	Malignant neoplasm of posterior pharynx
288570012	B08y.00	Malignant neoplasm of other specified hypopharyngeal site
288571011	B08z.00	Malignant tumour of hypopharynx
288573014	B0z0.00	Malignant neoplasm of pharynx unspecified
288578017	B0z1.00	Malignant neoplasm of Waldeyer's ring
288582015	B0zz.00	Malignant neoplasm of lip, oral cavity and pharynx NOS
288589012	B100.00	Malignant neoplasm of cervical oesophagus
288594012	B101.00	Malignant neoplasm of thoracic oesophagus
288597017	B102.00	Malignant neoplasm of abdominal oesophagus
288602012	B103.00	Malignant neoplasm of upper third of oesophagus
288607018	B104.00	Malignant neoplasm of middle third of oesophagus

2886080 11	B10 5.00	Malignant neoplasm of lower third of oesophagus
2886140 16	B10 y.00	Malignant neoplasm of other specified part of oesophagus
2886250 13	B11 0.00	Malignant neoplasm of cardia of stomach
2886280 10	B11 000 0	Malignant neoplasm of cardiac orifice of stomach
2886310 11	B11 011 1	Malignant neoplasm of gastro-oesophageal junction
2886320 16	B11 010 0	Malignant neoplasm of cardio-oesophageal junction of stomach
2886330 14	B11 0z0 0	Malignant neoplasm of cardia of stomach NOS
2886350 19	B11 1.00	Malignant neoplasm of pylorus of stomach
2886380 17	B11 110 0	Malignant neoplasm of pyloric canal of stomach
2886390 13	B11 1z0 0	Malignant neoplasm of pylorus of stomach NOS
2886410 14	B11 2.00	Malignant neoplasm of pyloric antrum of stomach
2886440 18	B11 3.00	Malignant neoplasm of fundus of stomach
2886470 13	B11 4.00	Malignant neoplasm of body of stomach
2886490 11	B11 5.00	Malignant tumour of lesser curve of stomach
2886500 11	B11 6.00	Malignant neoplasm of greater curve of stomach unspecified
2886520 15	B11 y.00	Malignant neoplasm of other specified site of stomach
2886530 13	B11 y00 0	Malignant neoplasm of anterior wall of stomach
2886540 19	B11 y10 0	Malignant neoplasm of posterior wall of stomach
2886550 18	B11 yz0 0	Malignant neoplasm of other specified site of stomach NOS
2886560 17	B11 z.00	Malignant tumour of stomach
2886590 12	B12 3.00	Malignant neoplasm of Meckel's diverticulum
2886630 17	B12 z.00	Malignant neoplasm of small intestine NOS
2886700 17	B13 8.00	Malignant neoplasm, overlapping lesion of colon
2886710 18	B13 y.00	Malignant neoplasm of other specified sites of colon
2886750 10	B14 ..00	Malignant neoplasm of rectum, rectosigmoid junction and anus

288685011	B143.00	Malignant tumour of anus
288688013	B15..00	Malignant neoplasm of liver and intrahepatic bile ducts
288690014	B150000	Primary carcinoma of liver
288692018	B150z00	Primary malignant neoplasm of liver NOS
288694017	B151000	Malignant neoplasm of interlobular bile ducts
288697012	B151300	Malignant neoplasm of intrahepatic canaliculi
288698019	B151400	Malignant neoplasm of intrahepatic gall duct
288699010	B151z00	Malignant neoplasm of intrahepatic bile ducts NOS
288700011	B152.00	Malignant neoplasm of liver
288702015	B15z.00	Malignant neoplasm of liver and intrahepatic bile ducts NOS
288707014	B161100	Malignant neoplasm of hepatic duct
288711015	B161300	Malignant neoplasm of sphincter of Oddi
288712010	B161z00	Malignant neoplasm of extrahepatic bile ducts NOS
288717016	B171.00	Malignant neoplasm of body of pancreas
288720012	B172.00	Malignant neoplasm of tail of pancreas
288723014	B173.00	Malignant neoplasm of pancreatic duct
288727010	B174.00	Malignant neoplasm of Islets of Langerhans
288731016	B17y.00	Malignant neoplasm of other specified sites of pancreas
288734012	B17y000	Malignant neoplasm of ectopic pancreatic tissue
288735013	B17yz00	Malignant neoplasm of specified site of pancreas NOS
288736014	B17z.00	Malignant neoplasm of pancreas NOS
288739019	B18..00	Malignant neoplasm of retroperitoneum and peritoneum
288741018	B180100	Malignant neoplasm of perinephric tissue

288742013	B180200	Malignant neoplasm of retrocaecal tissue
288744014	B180z00	Malignant neoplasm of retroperitoneum NOS
288747019	B18y.00	Malignant neoplasm of specified parts of peritoneum
288748012	B18y000	Malignant neoplasm of mesocolon
288749016	B18y100	Malignant neoplasm of mesocaecum
288751017	B18y200	Malignant neoplasm of mesorectum
288754013	B18y600	Malignant neoplasm of the pouch of Douglas
288756010	B18yz00	Malignant neoplasm of specified parts of peritoneum NOS
288757018	B18z.00	Malignant tumour of peritoneum and retroperitoneum
288759015	B1z0.00	Malignant tumour of intestine
288760013	B1z1.00	Malignant neoplasm of spleen NEC
288762017	B1z1100	Fibrosarcoma of spleen
288763010	B1z1z00	Malignant tumour of spleen
288764016	B1z2.00	Malignant neoplasm, overlapping lesion of digestive system
288766019	B1zz.00	Malignant neoplasm of digestive tract and peritoneum NOS
288771014	B200000	Malignant neoplasm of cartilage of nose
288772019	B200100	Malignant neoplasm of nasal conchae
288775017	B200300	Malignant neoplasm of vestibule of nose
288776016	B200z00	Malignant neoplasm of nasal cavities NOS
288779011	B201000	Malignant neoplasm of auditory (Eustachian) tube
288784017	B201100	Malignant neoplasm of tympanic cavity

288787012	B201200	Malignant neoplasm of tympanic antrum
288790018	B206.00	Malignant neoplasm, overlapping lesion of accessory sinuses
288792014	B20z.00	Malignant tumour of nasal sinuses
288794010	B210.00	Malignant neoplasm of glottis
288797015	B211.00	Malignant neoplasm of supraglottis
288799017	B213000	Malignant neoplasm of arytenoid cartilage
288800018	B213100	Malignant neoplasm of cricoid cartilage
288801019	B213200	Malignant neoplasm of cuneiform cartilage
288802014	B213300	Malignant neoplasm of thyroid cartilage
288803016	B213z00	Malignant tumour of laryngeal cartilage
288806012	B21y.00	Malignant neoplasm of larynx, other specified site
288807015	B21z.00	Malignant neoplasm of larynx NOS
288808013	B22..00	Malignant neoplasm of lower respiratory tract
288809017	B220000	Malignant neoplasm of cartilage of trachea
288810010	B220100	Malignant neoplasm of mucosa of trachea
288811014	B220z00	Malignant neoplasm of trachea NOS
288813012	B221000	Malignant neoplasm of carina of bronchus
288815017	B221z00	Malignant neoplasm of main bronchus NOS
288819011	B222000	Malignant neoplasm of upper lobe bronchus
288820017	B222100	Malignant neoplasm of upper lobe of lung
288821018	B222z00	Malignant neoplasm of upper lobe, bronchus or lung NOS
288822013	B223.00	Malignant neoplasm of middle lobe, bronchus or lung

2888230 15	B22 300 0	Malignant neoplasm of middle lobe bronchus
2888240 14	B22 310 0	Malignant neoplasm of middle lobe of lung
2888250 10	B22 3z0 0	Malignant neoplasm of middle lobe, bronchus or lung NOS
2888260 11	B22 4.00	Malignant neoplasm of lower lobe, bronchus or lung
2888270 19	B22 400 0	Malignant neoplasm of lower lobe bronchus
2888280 12	B22 410 0	Malignant neoplasm of lower lobe of lung
2888290 16	B22 4z0 0	Malignant neoplasm of lower lobe, bronchus or lung NOS
2888320 18	B22 y.00	Malignant neoplasm of other sites of bronchus or lung
2888390 10	B23 y.00	Malignant neoplasm of other specified pleura
2888400 12	B23 z.00	Malignant tumour of pleura
2888410 11	B24 ..00	Malignant neoplasm of thymus, heart and mediastinum
2888470 10	B24 1z0 0	Malignant tumour of heart
2888520 17	B24 z.00	Malignant neoplasm of heart, thymus and mediastinum NOS
2888570 11	B2z y.00	Malignant neoplasm of other site of respiratory tract
2888580 18	B2z z.00	Malignant neoplasm of respiratory system
2888690 15	B30 0.00	Malignant neoplasm of bones of skull and face
2888720 10	B30 020 0	Malignant neoplasm of malar bone
2888750 12	B30 050 0	Malignant neoplasm of orbital bone
2888830 18	B30 0z0 0	Malignant neoplasm of bones of skull and face NOS
2888850 13	B30 200 0	Malignant neoplasm of cervical vertebra
2888860 14	B30 210 0	Malignant neoplasm of thoracic vertebra
2888870 17	B30 220 0	Malignant neoplasm of lumbar vertebra

288888010	B302z00	Malignant neoplasm of vertebral column NOS
288889019	B303.00	Malignant neoplasm of ribs, sternum and clavicle
288894019	B303300	Malignant neoplasm of costal cartilage
288896017	B303500	Malignant neoplasm of xiphoid process
288897014	B303z00	Malignant neoplasm of rib, sternum and clavicle NOS
288898016	B304.00	Malignant neoplasm of scapula and long bones of upper arm
288901015	B304200	Malignant neoplasm of humerus
288909018	B305000	Malignant neoplasm of carpal bone - scaphoid
288910011	B305100	Malignant neoplasm of carpal bone - lunate
288911010	B305200	Malignant neoplasm of carpal bone - triquetrum
288912015	B305300	Malignant neoplasm of carpal bone - pisiform
288913013	B305400	Malignant neoplasm of carpal bone - trapezium
288914019	B305500	Malignant neoplasm of carpal bone - trapezoid
288915018	B305600	Malignant neoplasm of carpal bone - capitate
288916017	B305700	Malignant neoplasm of carpal bone - hamate
288917014	B305800	Malignant neoplasm of first metacarpal bone
288918016	B305900	Malignant neoplasm of second metacarpal bone
288919012	B305A00	Malignant neoplasm of third metacarpal bone
288920018	B305B00	Malignant neoplasm of fourth metacarpal bone
288921019	B305C00	Malignant neoplasm of fifth metacarpal bone

288922014	B305D00	Malignant neoplasm of phalanges of hand
288923016	B305z00	Malignant neoplasm of hand bones NOS
288924010	B306.00	Malignant neoplasm of pelvic bones, sacrum and coccyx
288928013	B306300	Malignant neoplasm of sacral vertebra
288929017	B306400	Malignant neoplasm of coccygeal vertebra
288930010	B306z00	Malignant neoplasm of pelvis, sacrum or coccyx NOS
288931014	B307.00	Malignant neoplasm of long bones of leg
288935017	B307z00	Malignant neoplasm of long bone of lower limb
288941012	B308200	Malignant neoplasm of calcaneum
288942017	B308300	Malignant neoplasm of medial cuneiform
288943010	B308400	Malignant neoplasm of intermediate cuneiform
288944016	B308500	Malignant neoplasm of lateral cuneiform
288946019	B308700	Malignant neoplasm of navicular
288947011	B308800	Malignant neoplasm of first metatarsal bone
288948018	B308900	Malignant neoplasm of second metatarsal bone
288949014	B308A00	Malignant neoplasm of third metatarsal bone
288950014	B308B00	Malignant neoplasm of fourth metatarsal bone
288951013	B308C00	Malignant neoplasm of fifth metatarsal bone
288952018	B308D00	Malignant neoplasm of phalanges of foot
288953011	B308z00	Malignant neoplasm of short bones of leg NOS

288959010	B30z.00	Malignant neoplasm of bone and articular cartilage
288960017	B31..00	Malignant neoplasm of connective and other soft tissue
288962013	B310300	Malignant neoplasm of cartilage of ear
288963015	B310400	Malignant neoplasm of tarsus of eyelid
288964014	B310500	Malignant neoplasm soft tissues of cervical spine
288967019	B311000	Malignant neoplasm of connective and soft tissue of shoulder
288968012	B311100	Malignant neoplasm of connective and soft tissue, upper arm
288969016	B311200	Malignant neoplasm of connective and soft tissue of fore-arm
288970015	B311300	Malignant neoplasm of connective and soft tissue of hand
288971016	B311400	Malignant neoplasm of connective and soft tissue of finger
288972011	B311500	Malignant neoplasm of connective and soft tissue of thumb
288975013	B312000	Malignant neoplasm of connective and soft tissue of hip
288979019	B312400	Malignant neoplasm of connective and soft tissue of foot
288980016	B312500	Malignant neoplasm of connective and soft tissue of toe
288985014	B313.00	Malignant neoplasm of connective and soft tissue of thorax
288986010	B313000	Malignant neoplasm of connective and soft tissue of axilla
288991011	B314.00	Malignant neoplasm of connective and soft tissue of abdomen
288995019	B315.00	Malignant neoplasm of connective and soft tissue of pelvis
288999013	B315000	Malignant neoplasm of connective and soft tissue of buttock
289001014	B315200	Malignant neoplasm of connective and soft tissue of perineum
289007013	B31z.00	Malignant neoplasm of connective and soft tissue, site NOS

289008015	B31z000	Kaposi's sarcoma of soft tissue
289009011	B320.00	Malignant melanoma of lip
289011019	B322.00	Malignant melanoma of ear and external auricular canal
289012014	B322000	Malignant melanoma of auricle (ear)
289013016	B322100	Malignant melanoma of external auditory meatus
289014010	B322z00	Malignant melanoma of ear and external auricular canal NOS
289015011	B323.00	Malignant melanoma of skin of face
289017015	B323100	Malignant melanoma of chin
289019017	B323300	Malignant melanoma of forehead
289021010	B323500	Malignant melanoma of temple
289022015	B323z00	Malignant melanoma of skin of face
289023013	B324.00	Malignant melanoma of scalp and neck
289024019	B324000	Malignant melanoma of scalp
289025018	B324100	Malignant melanoma of neck
289026017	B324z00	Malignant melanoma of scalp and/or neck
289027014	B325.00	Malignant melanoma of skin of trunk
289028016	B325000	Malignant melanoma of axilla
289029012	B325100	Malignant melanoma of breast
289030019	B325200	Malignant melanoma of buttock
289031015	B325300	Malignant melanoma of groin
289033017	B325500	Malignant melanoma of perineum

2890340 11	B32 560 0	Malignant melanoma of umbilicus
2890370 16	B32 5z0 0	Malignant melanoma of trunk
2890390 18	B32 600 0	Malignant melanoma of shoulder
2890400 16	B32 610 0	Malignant melanoma of upper arm
2890410 17	B32 620 0	Malignant melanoma of fore-arm
2890420 12	B32 630 0	Malignant melanoma of hand
2890430 19	B32 640 0	Malignant melanoma of finger
2890440 13	B32 650 0	Malignant melanoma of thumb
2890450 14	B32 6z0 0	Malignant melanoma of upper limb or shoulder NOS
2890460 10	B32 7.00	Malignant melanoma of lower limb and hip
2890470 18	B32 700 0	Malignant melanoma of hip
2890480 11	B32 710 0	Malignant melanoma of thigh
2890490 15	B32 720 0	Malignant melanoma of knee
2890500 15	B32 730 0	Malignant melanoma of popliteal fossa area
2890510 16	B32 740 0	Malignant melanoma of lower leg
2890520 11	B32 750 0	Malignant melanoma of ankle
2890530 18	B32 760 0	Malignant melanoma of heel
2890540 12	B32 770 0	Malignant melanoma of foot
2890550 13	B32 780 0	Malignant melanoma of toe
2890560 14	B32 790 0	Malignant melanoma of great toe

289057017	B327z00	Malignant melanoma of lower limb or hip NOS
289060012	B32y.00	Malignant melanoma of other specified skin site
289063014	B32z.00	Malignant melanoma of skin NOS
289066018	B33..14	Malignant neoplasm of sebaceous gland
289079010	B332000	Malignant neoplasm of skin of auricle (ear)
289080013	B332100	Malignant neoplasm of skin of external auditory meatus
289084016	B333000	Malignant neoplasm of skin of cheek, external
289088018	B334000	Malignant neoplasm of skin of nose (external)
289089014	B335000	Malignant neoplasm of skin of temple
289091018	B334.00	Malignant neoplasm of scalp and skin of neck
289092013	B334000	Malignant neoplasm of scalp
289094014	B334z00	Malignant neoplasm of scalp and/or skin of neck
289095010	B335.00	Malignant neoplasm of skin of trunk
289096011	B335000	Malignant neoplasm of skin of axillary fold
289097019	B335100	Malignant neoplasm of skin of chest, excluding breast
289099016	B335300	Malignant neoplasm of skin of abdominal wall
2891011000006116		Carcinoma, diffuse type
2891021000006112		Adenocarcinoma, diffuse type
2891031000006110		Adenocarcinoma - diffuse type
2891041000006117		Carcinoma - diffuse type
289107010	B335z00	Malignant neoplasm of skin of trunk, excluding scrotum, NOS

2891080 17	B33 6.00	Malignant neoplasm of skin of upper limb and shoulder
2891100 15	B33 610 0	Malignant neoplasm of skin of upper arm
2891110 16	B33 620 0	Malignant neoplasm of skin of fore-arm
2891140 12	B33 650 0	Malignant neoplasm of skin of thumb
2891150 13	B33 6z0 0	Malignant neoplasm of skin of upper limb or shoulder NOS
2891160 14	B33 7.00	Malignant neoplasm of skin of lower limb and hip
2891210 12	B33 730 0	Malignant neoplasm of skin of popliteal fossa area
2891220 17	B33 740 0	Malignant neoplasm of skin of lower leg
2891240 16	B33 760 0	Malignant neoplasm of skin of heel
2891270 11	B33 790 0	Malignant neoplasm of skin of great toe
2891280 18	B33 7z0 0	Malignant neoplasm of skin of lower limb or hip NOS
2891310 17	B33 y.00	Malignant neoplasm of other specified skin sites
2891320 12	B33 z.00	Malignant neoplasm of skin NOS
2891370 18	B34 0.00	Malignant neoplasm of nipple and areola of female breast
2891400 18	B34 0z0 0	Malignant neoplasm of nipple or areola of female breast NOS
2891410 19	B34 1.00	Malignant neoplasm of central part of female breast
2891420 14	B34 2.00	Malignant neoplasm of upper-inner quadrant of female breast
2891430 16	B34 3.00	Malignant neoplasm of lower-inner quadrant of female breast
2891440 10	B34 4.00	Malignant neoplasm of upper-outer quadrant of female breast
2891450 11	B34 5.00	Malignant neoplasm of lower-outer quadrant of female breast
2891460 12	B34 6.00	Malignant neoplasm of axillary tail of female breast
2891470 15	B34 7.00	Malignant neoplasm, overlapping lesion of breast
2891480 13	B34 y.00	Malignant neoplasm of other site of female breast
2891490 17	B34 y00 0	Malignant neoplasm of ectopic site of female breast

289150017	B34yz00	Malignant neoplasm of other site of female breast NOS
289151018	B34z.00	Malignant neoplasm of female breast NOS
289153015	B350.00	Malignant neoplasm of nipple and areola of male breast
289156011	B350z00	Malignant neoplasm of nipple or areola of male breast NOS
289157019	B35z.00	Malignant neoplasm of other site of male breast
289158012	B35z000	Malignant neoplasm of ectopic site of male breast
289159016	B35zz00	Malignant neoplasm of male breast NOS
289166015	B40..00	Malignant neoplasm of uterus
289171010	B410000	Malignant neoplasm of endocervical canal
289172015	B410100	Malignant neoplasm of endocervical gland
289173013	B410z00	Malignant neoplasm of endocervix NOS
289175018	B412.00	Malignant neoplasm, overlapping lesion of cervix uteri
289178016	B41y.00	Malignant neoplasm of other site of cervix
289179012	B41y000	Malignant neoplasm of cervical stump
289180010	B41y100	Malignant neoplasm of squamocolumnar junction of cervix
289181014	B41yz00	Malignant neoplasm of other site of cervix NOS
289182019	B41z.00	Malignant neoplasm of cervix uteri NOS
289184018	B911013	Choriocarcinoma
289187013	B430.00	Malignant neoplasm of corpus uteri, excluding isthmus
289188015	B430000	Malignant neoplasm of cornu of corpus uteri
289189011	B430100	Malignant neoplasm of fundus of corpus uteri
289190019	B430200	Malignant neoplasm of endometrium of corpus uteri

289191015	B430300	Malignant neoplasm of myometrium of corpus uteri
289192010	B430z00	Malignant neoplasm of corpus uteri NOS
289193017	B431.00	Malignant neoplasm of isthmus of uterine body
289194011	B431000	Malignant neoplasm of lower uterine segment
289195012	B431z00	Malignant neoplasm of isthmus of uterine body NOS
289196013	B432.00	Malignant neoplasm of overlapping lesion of corpus uteri
289197016	B43y.00	Malignant neoplasm of other site of uterine body
289198014	B43z.00	Malignant neoplasm of body of uterus NOS
289199018	B44..00	Malignant neoplasm of uterine adnexa
289202011	B444.00	Malignant neoplasm of round ligament
289203018	B44y.00	Malignant neoplasm of other site of uterine adnexa
289204012	B44z.00	Malignant neoplasm of uterine adnexa NOS
289206014	B450000	Malignant neoplasm of Gartner's duct
289207017	B450100	Malignant neoplasm of vaginal vault
289208010	B450z00	Malignant neoplasm of vagina NOS
289209019	B451000	Malignant neoplasm of greater vestibular (Bartholin's) gland
289210012	B451z00	Malignant neoplasm of labia majora NOS
289217010	B45y.00	Malignant neoplasm of other specified female genital organ
289219013	B45z.00	Malignant tumour of female genital organ
289223017	B470.00	Malignant neoplasm of undescended testis
289224011	B470000	Malignant neoplasm of ectopic testis
289234019	B470z00	Malignant neoplasm of undescended testis NOS
289242018	B48..00	Malignant neoplasm of penis and other male genital organs
289246015	B482.00	Malignant neoplasm of body of penis

2892490 10	B48 3.00	Malignant tumour of penis
2892510 14	B48 y.00	Malignant neoplasm of other male genital organ
2892520 19	B48 y00 0	Malignant neoplasm of seminal vesicle
2892570 13	B48 y10 0	Malignant neoplasm of tunica vaginalis
2892590 11	B48 yz0 0	Malignant neoplasm of other male genital organ NOS
2892600 18	B48 z.00	Malignant neoplasm of penis and other male genital organ NOS
2892620 14	B49 0.00	Malignant neoplasm of trigone of urinary bladder
2892670 15	B49 1.00	Malignant neoplasm of dome of urinary bladder
2892700 16	B49 2.00	Malignant neoplasm of lateral wall of urinary bladder
2892710 17	B49 3.00	Malignant neoplasm of anterior wall of urinary bladder
2892720 12	B49 4.00	Malignant neoplasm of posterior wall of urinary bladder
2892750 14	B49 5.00	Malignant neoplasm of bladder neck
2892760 10	B49 6.00	Malignant neoplasm of ureteric orifice
2892790 15	B49 y.00	Malignant neoplasm of other site of urinary bladder
2892800 17	B49 y00 0	Malignant neoplasm, overlapping lesion of bladder
2892810 18	B49 z.00	Malignant neoplasm of urinary bladder NOS
2892850 10	B4A 0.00	Malignant neoplasm of kidney parenchyma
2892880 12	B4A 000 0	Hypernephroma
2892940 16	B4A 110 0	Malignant neoplasm of ureteropelvic junction
2892960 19	B4A 1z0 0	Malignant neoplasm of renal pelvis NOS
2892970 11	B4A y.00	Malignant neoplasm of other urinary organs
2892980 18	B4A y00 0	Malignant neoplasm of overlapping lesion of urinary organs
2892990 14	B4A z.00	Malignant tumour of urinary system
2893010 19	B4z. .00	Malignant neoplasm of genitourinary organ NOS
2893080 13	B50 000 0	Malignant neoplasm of ciliary body

2893110 14	B50 010 0	Malignant neoplasm of iris
2893140 18	B50 020 0	Malignant neoplasm of crystalline lens
2893150 17	B50 030 0	Malignant neoplasm of sclera
2893160 16	B50 0z0 0	Malignant neoplasm of eyeball NOS
2893170 13	B50 100 0	Malignant neoplasm of connective tissue of orbit
2893180 15	B50 110 0	Malignant neoplasm of extraocular muscle of orbit
2893190 11	B50 1z0 0	Malignant neoplasm of orbit NOS
2893220 13	B50 2.00	Malignant neoplasm of lacrimal gland
2893250 10	B50 700 0	Malignant neoplasm of lacrimal sac
2893260 11	B50 710 0	Malignant neoplasm of nasolacrimal duct
2893270 19	B50 7z0 0	Malignant neoplasm of lacrimal duct NOS
2893290 16	B50 y.00	Malignant neoplasm of other specified site of eye
2893300 14	B50 z.00	Malignant neoplasm of eye NOS
2893370 12	B51 000 0	Malignant neoplasm of basal ganglia
2893380 19	B51 010 0	Malignant neoplasm of cerebral cortex
2893390 10	B51 020 0	Malignant neoplasm of corpus striatum
2893410 11	B51 030 0	Malignant neoplasm of globus pallidus
2893430 14	B51 040 0	Malignant neoplasm of hypothalamus
2893450 19	B51 050 0	Malignant neoplasm of thalamus
2893460 18	B51 0z0 0	Malignant neoplasm of cerebrum NOS

289347010	B512000	Malignant neoplasm of hippocampus
289348017	B512100	Malignant neoplasm of uncus
289349013	B512z00	Malignant neoplasm of temporal lobe NOS
289352017	B515000	Malignant neoplasm of choroid plexus
289353010	B515100	Malignant neoplasm of floor of cerebral ventricle
289354016	B515z00	Malignant neoplasm of cerebral ventricle NOS
289355015	B517000	Malignant neoplasm of cerebral peduncle
289356019	B517100	Malignant neoplasm of medulla oblongata
289357011	B517200	Malignant neoplasm of midbrain
289358018	B517300	Malignant neoplasm of pons
289360016	B51y.00	Malignant neoplasm of other parts of brain
289361017	B51y000	Malignant neoplasm of corpus callosum
289362012	B51y100	Malignant neoplasm of tapetum
289364013	B51yz00	Malignant neoplasm of other part of brain NOS
289365014	B51z.00	Malignant neoplasm of brain NOS
289369015	B520000	Malignant neoplasm of olfactory bulb
289374011	B520z00	Malignant neoplasm of cranial nerves NOS
289375012	B521000	Malignant neoplasm of cerebral dura mater
289376013	B521100	Malignant neoplasm of cerebral arachnoid mater
289378014	B521200	Malignant neoplasm of cerebral pia mater

289379018	B521z00	Malignant neoplasm of cerebral meninges NOS
289380015	B523000	Malignant neoplasm of spinal dura mater
289381016	B523100	Malignant neoplasm of spinal arachnoid mater
289382011	B523200	Malignant neoplasm of spinal pia mater
289383018	B523z00	Malignant neoplasm of spinal meninges NOS
289388010	B524300	Malignant neoplasm of peripheral nerve of thorax
289389019	B524400	Malignant neoplasm of peripheral nerve of abdomen
289390011	B524500	Malignant neoplasm of peripheral nerve of pelvis
289396017	B52y.00	Malignant neoplasm of other specified part of nervous system
289397014	B52z.00	Malignant neoplasm of nervous system
289406011	B540z00	Malignant tumour of adrenal gland
289409016	B542100	Malignant neoplasm of craniopharyngeal duct
289413011	B545.00	Primary malignant neoplasm of paraganglion
289419010	B545z00	Malignant neoplasm of aortic body or paraganglia NOS
289421017	B54y.00	Malignant neoplasm of other specified endocrine gland
289423019	B55..00	Malignant neoplasm of other and ill-defined sites
289424013	B550.00	Malignant neoplasm of head, neck and face
289425014	B550000	Malignant tumour of head and neck
289426010	B550100	Carcinoma of cheek
289427018	B550200	Malignant neoplasm of nose
289428011	B550300	Malignant neoplasm of jaw

289429015	B550400	Malignant tumour of neck
289430013	B550500	Malignant neoplasm of supraclavicular fossa
289431012	B550z00	Malignant neoplasm of head, neck and face NOS
289432017	B551.00	Malignant neoplasm of thorax
289435015	B551000	Malignant tumour of axilla
289436019	B551100	Malignant neoplasm of chest wall
289437011	B551200	Malignant neoplasm of intrathoracic site NOS
289438018	B551z00	Malignant neoplasm of thorax NOS
289441010	B552.00	Malignant neoplasm of abdomen
289442015	B553000	Malignant neoplasm of inguinal region
289445018	B553z00	Malignant neoplasm of pelvis NOS
289446017	B554.00	Malignant neoplasm of upper limb NOS
289447014	B555.00	Malignant tumour of lower limb
289448016	B55y.00	Malignant neoplasm of other specified sites
289449012	B55y000	Malignant neoplasm of back
289450012	B55y100	Malignant neoplasm of trunk
289451011	B55y200	Malignant neoplasm of flank
289452016	B55yz00	Malignant neoplasm of specified site NOS
289515018	B573.00	Secondary malignant neoplasm of respiratory tract
2895271000006116		Pilomatrix carcinoma
2895281000006118		Pilomatrixoma, malignant

2895291 0000061 15		Pilomatricoma, malignant
2895300 10	B57 y.00	Secondary malignant neoplasm of gastrointestinal tract
2895301 0000061 19		Matrical carcinoma
2895311 0000061 16		Malignant pilomatrixoma
2895350 17	B58 1.00	Secondary malignant neoplasm of other urinary organs
2895360 16	B58 1z0 0	Secondary malignant neoplasm of other urinary organ NOS
2895380 15	B58 200 0	Secondary malignant neoplasm of skin of head
2895420 17	B58 240 0	Secondary malignant neoplasm of skin of shoulder and arm
2895430 10	B58 250 0	Secondary malignant neoplasm of skin of hip and leg
2895450 15	B58 2z0 0	Secondary malignant neoplasm of skin NOS
2895460 19	B58 3.00	Secondary malignant neoplasm of brain and spinal cord
2895490 14	B58 3z0 0	Secondary malignant neoplasm of brain or spinal cord NOS
2895500 14	B58 4.00	Secondary malignant neoplasm of other part of nervous system
2895530 11	B58 y20 0	Secondary malignant neoplasm of cervix uteri
2895540 17	B58 y21 1	Secondary cancer of the cervix
2895570 12	B58 y80 0	Secondary malignant neoplasm of epididymis and vas deferens
2895590 10	B58 z.00	Secondary malignant neoplasm of other specified site NOS
2895610 18	B59 ..00	Malignant neoplasm of unspecified site
2895650 10	B59 1.00	Other malignant neoplasm NOS
2895660 11	B59 2.00	Malignant neoplasms of independent (primary) multiple sites
2895710 16	B59 z.00	Malignant neoplasm of unspecified site NOS
2895740 12	B5z. .00	Malignant neoplasm of other and unspecified site NOS
2895800 16	B60 ..00	Lymphosarcoma and reticulosarcoma

289581017	B600000	Reticulosarcoma of unspecified site
289582012	B600100	Reticulosarcoma of lymph nodes of head, face and neck
289590012	B600z00	Reticulosarcoma NOS
289591011	B601.00	Lymphosarcoma
289592016	B601000	Lymphosarcoma of unspecified site
289593014	B601100	Lymphosarcoma of lymph nodes of head, face and neck
289594015	B601200	Lymphosarcoma of intrathoracic lymph nodes
289595019	B601300	Lymphosarcoma of intra-abdominal lymph nodes
289599013	B601700	Lymphosarcoma of spleen
289601010	B601z00	Lymphosarcoma NOS
289603013	B602100	Burkitt's lymphoma of lymph nodes of head, face and neck
289604019	B602200	Burkitt's lymphoma of intrathoracic lymph nodes
289605018	B602300	Burkitt's lymphoma of intra-abdominal lymph nodes
289612010	B602z00	Burkitt's lymphoma NOS
289614011	B60z.00	Reticulosarcoma or lymphosarcoma NOS
289621011	B610300	Hodgkin's paragranuloma of intra-abdominal lymph nodes
289629013	B611100	Hodgkin's granuloma of lymph nodes of head, face and neck
289643015	B612400	Hodgkin's sarcoma of lymph nodes of axilla and upper limb
289659015	B613z00	Hodgkin's, lymphocytic-histiocytic predominance NOS
289660013	B614000	Hodgkin's disease, nodular sclerosis of unspecified site

2896690 14	B61 4z0 0	Hodgkin's disease, nodular sclerosis NOS
2896700 10	B61 500 0	Hodgkin's disease, mixed cellularity of unspecified site
2896790 11	B61 5z0 0	Hodgkin's disease, mixed cellularity NOS
2896800 14	B61 600 0	Hodgkin's lymphocytic depletion of unspecified site
2896990 17	B61 670 0	Hodgkin's disease, lymphocytic depletion of spleen
2897010 17	B61 6z0 0	Hodgkin's disease, lymphocytic depletion NOS
2897020 12	B61 zz00	Hodgkin's disease NOS
2897030 19	B61 z00 0	Hodgkin's disease NOS, unspecified site
2897040 13	B61 z10 0	Hodgkin's disease of lymph nodes of head, face AND/OR neck
2897050 14	B61 z20 0	Hodgkin's disease of intrathoracic lymph nodes
2897100 13	B61 z70 0	Hodgkin's disease of spleen
2897110 12	B61 z80 0	Hodgkin's disease of lymph nodes of multiple sites
2897130 10	B62 ..00	Other malignant neoplasm of lymphoid and histiocytic tissue
2897170 11	B62 000 0	Nodular lymphoma of unspecified site
2897180 18	B62 010 0	Nodular lymphoma of lymph nodes of head, face and neck
2897260 14	B62 0z0 0	Nodular lymphoma NOS
2897270 17	B62 100 0	Mycosis fungoides of unspecified site
2897380 17	B62 180 0	Mycosis fungoides of lymph nodes of multiple sites
2897390 13	B62 1z0 0	Mycosis fungoides NOS
2897490 11	B62 2z0 0	Sezary's disease NOS

289750011	B623000	Malignant histiocytosis of unspecified site
289754019	B623z00	Malignant histiocytosis NOS
289756017	B624000	LRE - Leukaemic reticuloendotheliosis
289773015	B624z00	Leukaemic reticuloendotheliosis NOS
289776011	B625000	Letterer-Siwe disease of unspecified sites
289780018	B625z00	Letterer-Siwe disease NOS
289781019	B626.00	Malignant mast cell tumours
289783016	B626000	Mast cell malignancy
289791013	B626800	Mast cell malignancy of lymph nodes of multiple sites
289793011	B626z00	Malignant mast cell tumour NOS
289799010	B627300	Diffuse non-Hodgkin's small cell (diffuse) lymphoma
289812013	B627800	Diffuse non-Hodgkin's lymphoma undifferentiated (diffuse)
289813015	B627B00	Other types of follicular non-Hodgkin's lymphoma
289820010	B62x.00	Malignant lymphoma (clinical)
289826016	B62x500	Malignant immunoproliferative small intestinal disease
289829011	B62yz00	Malignant lymphoma NOS
289830018	B62y000	Malignant lymphoma NOS of unspecified site
289831019	B62y100	Malignant lymphoma of lymph nodes of head, face AND/OR neck
289832014	B62y200	Malignant lymphoma of intrathoracic lymph nodes
289842011	B62y300	Malignant lymphoma of intra-abdominal lymph nodes

289845013	B62y600	Malignant lymphoma of intrapelvic lymph nodes
289846014	B62y700	Malignant lymphoma of spleen
289847017	B62y800	Malignant lymphoma of lymph nodes of multiple sites
289849019	B62z.00	Malignant neoplasms of lymphoid and histiocytic tissue NOS
289867014	B630000	Malignant plasma cell neoplasm, extramedullary plasmacytoma
289874016	B63z.00	Immunoproliferative neoplasm or myeloma NOS
289875015	B64..11	Lymphatic leukaemia
289876019	B64..00	Lymphoid leukaemia
289880012	B642.00	Subacute lymphoid leukaemia
289881011	B64y.00	Other lymphoid leukaemia
289885019	B64y200	Adult T-cell leukaemia
289888017	B64yz00	Other lymphoid leukaemia NOS
289889013	B64z.00	Lymphoid leukaemia NOS
289892012	B65..00	Myeloid leukaemia
289895014	B651000	Chronic eosinophilic leukaemia
289898011	B651200	Chronic neutrophilic leukaemia
289900013	B651z00	Chronic myeloid leukaemia NOS
289901012	B652.00	Subacute myeloid leukaemia
289901100000006115		Topical chemotherapy for malignant neoplasm
289904016	B653100	Granulocytic sarcoma
289911017	B65yz00	Other myeloid leukaemia NOS
289912012	B65z.00	Myeloid leukaemia NOS
289917018	B66..00	Monocytic leukaemia

2899190 15	B66 ..12	Monoblastic leukaemia
2899230 11	B66 1.00	Chronic monocytic leukaemia
2899240 17	B66 2.00	Subacute monocytic leukaemia
2899260 15	B66 y.00	Other monocytic leukaemia
2899300 17	B66 yz0 0	Other monocytic leukaemia NOS
2899330 15	B66 z.00	Monocytic leukaemia NOS
2899340 14	B67 ..00	Other specified leukaemia
2899480 14	B67 2.11	Thrombocytic leukaemia
2899490 18	B67 2.00	Megakaryocytic leukaemia
2899550 11	B67 y.00	Other and unspecified leukaemia
2899590 17	B67 yz0 0	Other and unspecified leukaemia NOS
2899600 10	B67 z.00	Other specified leukaemia NOS
2899630 12	B68 ..00	Leukaemia of unspecified cell type
2899640 18	B68 0.00	Acute leukaemia
2899670 13	B68 1.00	Chronic leukaemia
2899680 15	B68 2.00	Subacute leukaemia
2899710 11	B68 y.00	Other leukaemia of unspecified cell type
2899720 16	B68 z.00	Leukaemia
2899740 15	B69 ..00	Myelomonocytic leukaemia
2899800 11	B69 2.00	Subacute myelomonocytic leukaemia
2899870 14	B6z. .00	Malignant neoplasm lymphatic or haematopoietic tissue NOS
2902531 0000061 19		Adenocarcinoma, intestinal type
2902541 0000061 12		Carcinoma, intestinal type
2902551 0000061 14		Carcinoma - intestinal type
2905761 0000061 12		Hepatocellular carcinoma
2905771 0000061 17		Liver cell carcinoma

2905781 0000061 19		Hepatocarcinoma
2905791 0000061 16		Hepatoma, malignant
2907180 13	B80 0z0 0	Carcinoma in situ of lip, oral cavity and pharynx NOS
2907320 10	B80 1z0 0	Carcinoma in situ of oesophagus NOS
2907410 17	B80 2z0 0	Carcinoma in situ of stomach NOS
2907490 15	B80 370 0	Carcinoma in situ of splenic flexure of colon
2907500 15	B80 3z0 0	Carcinoma in situ of colon NOS
2907510 16	B80 4.00	Carcinoma in situ of rectum and rectosigmoid junction
2907520 11	B80 4z0 0	Carcinoma in situ of rectum or rectosigmoid junction NOS
2907530 18	B80 6.00	Carcinoma in situ of anus
2907580 10	B80 7.00	Carcinoma in situ of other and unspecified small intestine
2907680 17	B80 820 0	Carcinoma in situ of hepatic duct
2907710 13	B80 870 0	Carcinoma in situ of sphincter of Oddi
2907720 18	B80 8z0 0	Carcinoma in situ of liver and/or biliary system
2907730 11	B80 z.00	Carcinoma in situ of other and unspecified digestive organs
2907750 16	B80 zz00	Carcinoma in situ of digestive organs NOS
2907760 15	B81 ..00	Carcinoma in situ of respiratory system
2907770 12	B81 000 0	Carcinoma in situ of thyroid cartilage
2907780 19	B81 010 0	Carcinoma in situ of cricoid cartilage
2907800 13	B81 030 0	Carcinoma in situ of arytenoid cartilage
2907810 12	B81 040 0	Carcinoma in situ of corniculate cartilage

290782017	B810500	Carcinoma in situ of cuneiform cartilage
290783010	B810600	Carcinoma in situ of aryepiglottic fold
290784016	B810700	Carcinoma in situ of vestibular fold
290787011	B810z00	Carcinoma in situ of larynx NOS
290788018	B812.00	Carcinoma in situ of bronchus and lung
290789014	B812000	Carcinoma in situ of carina of bronchus
290791018	B812200	Carcinoma in situ of upper lobe bronchus and lung
290792013	B812300	Carcinoma in situ of middle lobe bronchus and lung
290793015	B812400	Carcinoma in situ of lower lobe bronchus and lung
290794014	B812z00	Carcinoma in situ of bronchus or lung NOS
290800017	B81y200	Carcinoma in situ of tympanic cavity
290801018	B81y300	Carcinoma in situ of tympanic antrum
290804014	B81z.00	Carcinoma in situ of respiratory organ NOS
290806011	B821.00	Carcinoma in situ of skin of eyelid including canthus
290808012	B822000	Carcinoma in situ of skin of auricle
290809016	B822100	Carcinoma in situ of skin of external auricular canal
290818019	B823500	Carcinoma in situ of skin of temple
290819010	B823600	Carcinoma in situ of skin of jaw
290821017	B824.00	Carcinoma in situ of scalp and skin of neck
290822012	B824000	Carcinoma in situ of scalp
290824013	B824z00	Carcinoma in situ of scalp or skin of neck NOS

290825014	B825.00	Carcinoma in situ of skin of trunk, excluding scrotum
290827018	B825100	Carcinoma in situ of skin of chest wall NOS
290830013	B825400	Carcinoma in situ of skin of abdominal wall
290835015	B825z00	Carcinoma in situ of skin of trunk NOS
290836019	B826.00	Carcinoma in situ of skin of upper limb and shoulder
290838018	B826100	Carcinoma in situ of skin of upper arm
290839014	B826200	Carcinoma in situ of skin of lower arm
290841010	B826z00	Carcinoma in situ of skin of upper limb or shoulder NOS
290843013	B827.00	Carcinoma in situ of skin of lower limb
290849012	B827300	Carcinoma in situ of skin of lower leg
290851011	B827z00	Carcinoma in situ of skin of lower limb or hip NOS
290865013	B82y.00	Carcinoma in situ of other specified sites of skin
290866014	B82z.00	Carcinoma in situ of skin NOS
290867017	B83..00	Carcinoma in situ of breast and genitourinary system
290868010	B830.00	Carcinoma in situ of breast
290889018	B833z00	Carcinoma in situ of female genital organs NOS
290893012	B836z00	Carcinoma in situ of male genital organs NOS
290894018	B83z.00	Carcinoma in situ of urinary organs NOS
290895017	B8y..00	Carcinoma in situ of other and unspecified sites
290896016	B8yy.00	Carcinoma in situ of other specified site
290900018	B8yyz00	Carcinoma in situ of other specified site NOS
290901019	B8z..00	Carcinoma in situ NOS
291043100000611 7		International Federation of Gynaecology and Obstetrics cervical cancer stage Ib

29104410000061 10		International Federation of Gynecology and Obstetrics cervical cancer (FIGO CC) stage Ib
29104510000061 12		International Federation of Gynecology and Obstetrics cervical cancer stage Ib
29104610000061 14		International Federation of Gynaecology and Obstetrics cervical cancer (FIGO CC) stage Ib
291307016	BB0..00	[M]Neoplasms NOS
291322014	BB1..00	[M]Epithelial neoplasms NOS
291326012	BB12.00	Malignant Neoplasm (Morphology)
291327015	BB13.00	Carcinoma, metastatic
291328013	BB17.00	Large cell carcinoma
291329017	BB18.00	Carcinoma, undifferentiated
291330010	BB19.00	Carcinoma, anaplastic
291331014	BB1G.00	[M]Spheroidal cell carcinoma
291332019	BB1J.00	Small cell carcinoma
291337013	BB1z.00	[M]Unspecified epithelial neoplasm
291343010	BB22.00	Papillary carcinoma
291344016	BB24.00	Verrucous carcinoma
291347011	BB2A.00	Squamous cell carcinoma - category
291348018	BB2B.00	Squamous cell carcinoma, metastatic
291358019	BB2z.00	[M]Papillary or squamous cell neoplasm NOS
291360017	BB31.00	Basal cell carcinoma
291361018	BB3z.00	[M]Basal cell neoplasm NOS
291363015	BB43.00	Transitional cell carcinoma
291364014	BB4z.00	[M]Transitional cell papilloma or carcinoma NOS
291372011	BB52.00	Adenocarcinoma
291374012	BB53.00	Adenocarcinoma, metastatic
291375013	BB5B.00	[M]Pancreatic adenomas and carcinomas

291377017	BB5Bz00	[M]Pancreatic adenoma or carcinoma NOS
291378010	BB5C.00	[M]Gastrinoma and carcinomas
291379019	BB5Cz00	[M]Gastrinoma or carcinoma NOS
291380016	BB5D.00	[M]Hepatobiliary tract adenomas and carcinomas
291391011	BB5L.00	[M]Adenomatous and adenocarcinomatous polyps
291395019	BB5Lz00	[M]Adenomatous or adenocarcinomatous polyp NOS
291396018	BB5M.00	[M]Tubular adenomas and adenocarcinomas
291398017	BB5Mz00	[M]Tubular adenoma or adenocarcinoma NOS
291402014	BB5Nz00	[M]Adenomatous or adenocarcinomatous polyps of the colon NOS
291403016	BB5P.00	[M]Solid carcinoma NOS
291414018	BB5R100	[M]Carcinoid tumour, malignant
291419011	BB5R500	[M]Carcinoid tumour, nonargentaffin, malignant
291426011	BB5S.00	[M]Respiratory tract adenomas and adenocarcinomas
291428012	BB5Sz00	[M]Respiratory tract adenoma or adenocarcinoma NOS
291429016	BB5T.00	[M]Papillary adenomas and adenocarcinomas
291431013	BB5T100	Papillary adenocarcinoma
291433011	BB5Tz00	[M]Papillary adenoma or adenocarcinoma NOS
291434017	BB5U.00	[M]Villous adenomas and adenocarcinomas
291436015	BB5U100	Adenocarcinoma in villous adenoma
291437012	BB5Uz00	[M]Villous adenoma or adenocarcinoma NOS
291438019	BB5V.00	[M]Pituitary adenomas and carcinomas

2914390 10	BB5 Vz0 0	[M]Pituitary adenoma or carcinoma NOS
2914400 12	BB5 W.0 0	[M]Oxyphilic adenomas and adenocarcinomas
2914410 11	BB5 Wz0 0	[M]Oxyphilic adenoma or adenocarcinoma NOS
2914420 16	BB5 X.00	[M]Clear cell adenomas and adenocarcinomas
2914430 14	BB5 X10 0	Clear cell adenocarcinoma
2914440 15	BB5 Xz0 0	[M]Clear cell adenoma or adenocarcinoma NOS
2914450 19	BB5 a.00	[M]Renal adenoma and carcinoma
2914460 18	BB5 az0 0	[M]Renal adenoma or carcinoma NOS
2914470 10	BB5 c.00	[M]Parathyroid adenomas and adenocarcinomas
2914480 17	BB5 cz0 0	[M]Parathyroid adenoma or adenocarcinoma NOS
2914490 13	BB5 d.00	[M]Mixed cell adenoma and adenocarcinoma
2914500 13	BB5 dz0 0	[M]Mixed cell adenoma or adenocarcinoma NOS
2914510 12	BB5 f.00	[M]Thyroid adenoma and adenocarcinoma
2914520 17	BB5 f10 0	Follicular adenocarcinoma
2914540 16	BB5 fz00	[M]Thyroid adenoma or adenocarcinoma NOS
2914620 12	BB5 j.00	[M]Endometrioid adenomas and carcinomas
2914650 14	BB5 jz00	[M]Endometrioid adenoma or carcinoma NOS
2914700 19	BB5 z.00	[M]Adenoma or adenocarcinoma NOS
2914720 10	BB6 0.00	[M]Skin appendage adenoma and carcinoma
2914740 11	BB6 010 0	Skin appendage carcinoma
2914750 12	BB6 0z0 0	[M]Skin appendage adenoma or carcinoma NOS
2914760 13	BB6 1.00	[M]Sweat gland adenoma and adenocarcinomas
2914800 15	BB6 120 0	Sweat gland adenocarcinoma

2914810 16	BB6 1z0 0	[M]Sweat gland adenoma or adenocarcinoma NOS
2914820 11	BB6 2.00	[M]Apocrine adenoma and adenocarcinomas
2914830 18	BB6 2z0 0	[M]Apocrine adenoma or adenocarcinoma NOS
2914840 12	BB6 9.00	[M]Sebaceous adenoma and adenocarcinoma
2914850 13	BB6 9z0 0	[M]Sebaceous adenoma or adenocarcinoma NOS
2914860 14	BB6 A.0 0	[M]Ceruminous adenoma and adenocarcinoma
2914870 17	BB6 Az0 0	[M]Ceruminous adenoma or adenocarcinoma NOS
2914890 19	BB6 z.00	[M]Adnexal and skin appendage neoplasm NOS
2914900 11	BB7 ..00	[M]Mucoepidermoid neoplasms
2914910 10	BB7 z.00	[M]Mucoepidermoid neoplasm NOS
2914920 15	BB8 ..00	[M]Cystic, mucinous and serous neoplasms
2914930 13	BB8 0.00	[M]Cystadenoma and carcinoma
2914950 18	BB8 010 0	Cystadenocarcinoma
2914960 17	BB8 0z0 0	[M]Cystadenoma or carcinoma NOS
2915080 10	BB8 120 0	Serous cystadenocarcinoma
2915100 12	BB8 150 0	Papillary cystadenocarcinoma
2915150 19	BB8 1E0 0	Mucinous cystadenocarcinoma
2915240 11	BB8 1z0 0	[M]Ovarian cystic, mucinous or serous neoplasm NOS
2915250 12	BB8 2.00	[M]Mucinous adenoma and adenocarcinoma
2915260 13	BB8 2z0 0	[M]Mucinous adenoma or adenocarcinoma NOS
2915280 14	BB8 5z0 0	[M]Signet ring carcinoma NOS
2915290 18	BB8 z.00	[M]Cystic, mucinous or serous neoplasm NOS
2915300 11	BB9 ..00	[M]Ductal, lobular and medullary neoplasms

2915360 17	BB9 3.00	[M]Comedocarcinoma NOS
2915380 16	BB9 B.0 0	Medullary carcinoma
2915390 12	BB9 E00 0	[M]Intraductal carcinoma and lobular carcinoma in situ
2915400 14	BB9 F.00	Lobular carcinoma
2915430 11	BB9 z.00	[M]Ductal, lobular or medullary neoplasm NOS
2915440 17	BBA ..00	[M]Acinar cell neoplasms
2915470 12	BBA z.00	Acinar cell neoplasm
2915480 19	BBB ..00	[M]Complex epithelial neoplasms
2915530 12	BBC ..00	[M]Specialised gonadal neoplasms
2915531 0000061 11		Malignant down
2915580 15	BBC 1.00	[M]Thecal cell neoplasms
2915600 18	BBC 110 0	[M]Theca cell carcinoma
2915630 16	BBC 1z0 0	[M]Thecal cell neoplasm NOS
2916260 19	BBG ..00	[M]Fibromatous neoplasms
2916360 10	BBH ..00	[M]Myxomatous neoplasms
2916400 18	BBJ B.0 0	[M]Angiolipomatous neoplasms
2916440 10	BBJ Bz0 0	[M]Angiolipomatous neoplasm NOS
2916480 13	BBJz .00	[M]Lipomatous neoplasms NOS
2916490 17	BBK ..00	[M]Myomatous neoplasms
2916500 17	BBK 0.00	[M]Leiomyomatous neoplasms
2916580 12	BBK 0z0 0	[M]Leiomyomatous neoplasm NOS
2916590 16	BBK 1.00	[M]Angiomyomatous neoplasms
2916600 14	BBK 1z0 0	[M]Angiomyomatous neoplasm NOS
2916630 11	BBK 3.00	[M]Rhabdomyomatous neoplasms

291667012	BBK3z00	[M]Rhabdomyomatous neoplasm NOS
291669010	BBL4.00	[M]Mixed tumour, malignant, NOS
291671010	BBL7.00	[M]Mixed and stromal renal neoplasms
291675018	BBL7z00	[M]Mixed or stromal renal neoplasm NOS
291684018	BBLz.00	[M]Complex mixed or stromal neoplasm NOS
291685017	BBM..00	[M]Fibroepithelial neoplasms
291699018	BBN..00	[M]Synovial neoplasms
291701018	BBNz.00	[M]Synovial neoplasm NOS
291702013	BBP..00	[M]Mesothelial neoplasms
291713011	BBQ..00	[M]Germ cell neoplasms
291718019	BBQ3.00	Embryonal carcinoma
291728011	BBQA.00	[M]Strumal neoplasms
291730013	BBQAz00	[M]Strumal neoplasm NOS
291734016	BBR..00	[M]Trophoblastic neoplasms
291737011	BBRz.00	Trophoblastic neoplasm
291752016	BBTDz00	[M]Haemangiopericytic neoplasm NOS
291780019	BBW..00	[M]Chondromatous neoplasms
291786013	BBWz.00	[M]Chondromatous neoplasm NOS
291824019	BBb6.00	[M]Choroid plexus papilloma, malignant
291843019	BBc..00	[M]Neuroepitheliomatous neoplasms
291844013	BBc0.00	[M]Ganglioneuromatous neoplasms
291846010	BBc0z00	[M]Ganglioneuromatous neoplasm NOS
291891018	BBg1000	[M]Malignant lymphoma, diffuse NOS
291892013	BBg3.00	[M]Malignant lymphoma, undifferentiated cell type NOS

2918940 14	BBg 5.00	[M]Malignant lymphoma, convoluted cell type NOS
2919000 14	BBg A.0 0	[M]Malignant lymphoma, centroblastic-centrocytic, diffuse
2919020 18	BBg B.0 0	[M]Malignant lymphoma, follicular centre cell NOS
2919030 11	BBg C.00	[M]Malignant lymphoma, lymphocytic, well differentiated NOS
2919050 16	BBg E.00	[M]Malignant lymphoma, centrocytic
2919070 12	BBg F.00	[M]Malignant lymphoma, follicular centre cell, cleaved NOS
2919100 17	BBg J.00	[M]Malignant lymphoma, centroblastic type NOS
2919130 15	BBg Q.0 0	[M]Malignant lymphomatous polyposis
2919140 14	BBg S.00	[M]Malignant lymphoma, large cell, cleaved, diffuse
2919430 18	BBk 2.00	[M]Malignant lymphoma, centroblastic-centrocytic, follicular
2920990 15	Byu 1.00	Malignant tumour of digestive organ
2921010 10	Byu 110 0	Liver carcinoma
2921020 15	Byu 120 0	[X]Malignant neoplasm of intestinal tract, part unspecified
2921050 18	Byu 200 0	Malignant neoplasm of bronchus or lung
2921100 19	Byu 250 0	Malignant tumour of mediastinum
2921110 15	Byu 3.00	Malignant neoplasm of skeletal system
2921160 13	Byu 4.00	[X]Melanoma and other malignant neoplasms of skin
2921180 14	Byu 410 0	[X]Malignant melanoma of skin, unspecified
2921200 12	Byu 430 0	[X]Malignant neoplasm of skin, unspecified
2921210 11	Byu 5.00	[X]Malignant neoplasm of mesothelial and soft tissue
2921260 18	Byu 510 0	Cancer, mesothelioma
2921280 17	Byu 530 0	[X]Kaposi's sarcoma, unspecified
2921320 11	Byu 570 0	[X]Malignant neoplasm of peritoneum, unspecified

2921350 13	Byu 5A0 0	Overlapping malignant neoplasm of skin
2921360 14	Byu 5B0 0	Kaposi's sarcoma (clinical)
2921370 17	Byu 6.00	Malignant tumour of breast
2921380 10	Byu 7.00	[X]Malignant neoplasm of female genital organs
2921390 19	Byu 700 0	[X]Malignant neoplasm of uterine adnexa, unspecified
2921420 13	Byu 730 0	[X]Malignant neoplasm of female genital organ, unspecified
2921430 15	Byu 8.00	Malignant tumour of male genital organ
2921460 11	Byu 820 0	[X]Malignant neoplasm of male genital organ, unspecified
2921470 19	Byu 9.00	[X]Malignant neoplasm of urinary tract
2921480 12	Byu 900 0	Malignant neoplasm of urinary organ
2921520 12	Byu A20 0	Malignant tumour of meninges
2921540 13	Byu B.0 0	Malignant neoplasm of endocrine gland
2921560 10	Byu B10 0	Malignant neoplasm of endocrine gland
2921580 11	Byu C00 0	[X]Malignant neoplasm of other specified sites
2921650 15	Byu C70 0	[X]Secondary malignant neoplasm of other specified sites
2921660 19	Byu C80 0	[X]Malignant neoplasm without specification of site
2921690 14	Byu D00 0	[X]Other Hodgkin's disease
2921700 10	Byu D10 0	[X]Other types of follicular non-Hodgkin's lymphoma
2921730 12	Byu D20 0	[X]Other types of diffuse non-Hodgkin's lymphoma
2921740 18	Byu D30 0	[X]Other specified types of non-Hodgkin's lymphoma
2921750 17	Byu D40 0	Malignant immunoproliferative disease (clinical)

292177013	ByuD500	[X]Other lymphoid leukaemia
292178015	ByuD600	[X]Other myeloid leukaemia
292181013	ByuD700	[X]Other monocytic leukaemia
292182018	ByuD800	[X]Other specified leukaemias
292185016	ByuD900	[X]Other leukaemia of unspecified cell type
292191019	ByuDC00	Diffuse non-Hodgkin's lymphoma
292193016	ByuDE00	[X]Unspecified B-cell non-Hodgkin's lymphoma
292197015	ByuF.00	[X]In situ neoplasms
292199017	ByuF100	[X]Carcinoma in situ of other specified digestive organs
292200019	ByuF200	[X]Carcinoma in situ of digestive organ, unspecified
292201015	ByuF300	[X]Carcinoma in situ of other parts of respiratory system
292202010	ByuF400	[X]Carcinoma in situ of respiratory system, unspecified
292206013	ByuF800	[X]Carcinoma in situ of skin of other sites
292207016	ByuF900	[X]Carcinoma in situ of skin, unspecified
292208014	ByuFA00	[X]Carcinoma in situ of other parts of cervix
292212015	ByuFE00	[X]Carcinoma in situ of other specified sites
292214019	ByuFG00	[X]Other carcinoma in situ of breast
2926991000006116		Excision of carcinoma of urethra
2930481000006112		Odontogenic tumour, malignant
2930491000006110		Odontogenic tumor, malignant

2930501000006119		Odontogenic carcinoma
2930521000006112		Intraosseous carcinoma
2930531000006110		Carcinoma arising in an odontogenic cyst
2930541000006117		Primary intraosseous carcinoma
2930561000006118		Malignant odontogenic tumor
2933711000006115		Solid and papillary epithelial neoplasm
2933951000006116		Transitional carcinoma
2933961000006119		Urothelial carcinoma
2933971000006114		TCC - Transitional cell carcinoma
2943491000006117		Fulguration of carcinoma of urethra
2950221000006113		Embryonal adenocarcinoma
2952051000006114		Follicular adenocarcinoma, well differentiated
2952061000006111		Follicular carcinoma, well differentiated
2952071000006116		Follicular adenocarcinoma - well differentiated
2952081000006118		Follicular carcinoma - well differentiated
2954751000006115		Embryonal carcinoma, polyembryonal type
2954761000006118		Embryonal carcinoma - polyembryonal type
2958641000006118		Villous adenocarcinoma
2964691000006114		Squamous cell carcinoma

2964701 0000061 14		Squamous carcinoma
2964731 0000061 18		SCC - Squamous cell carcinoma
2964741 0000061 11		Squamous cell carcinoma, no ICD-O subtype
2964751 0000061 13		Squamous cell carcinoma, no International Classification of Diseases for Oncology subtype
2972241 0000061 17		Phaeochromocytoma, malignant
2972251 0000061 15		Pheochromocytoma, malignant
2972271 0000061 13		Adrenal medullary paraganglioma, malignant
2972301 0000061 10		Malignant pheochromocytoma
2976700 18	F40 210 0	Malignant myopia
2979601 0000061 13		Trabecular adenocarcinoma
2979611 0000061 11		Trabecular carcinoma
2985501 0000061 18		Cribriform carcinoma
2985511 0000061 15		Ductal carcinoma, cribriform type
2987620 15	F4H 600 0	Visual pathway disorder due to neoplasm
2987690 12	F4H 700 0	Visual cortex disorder due to neoplasm
2987751 0000061 15		Endometrioid adenocarcinoma
2987761 0000061 18		Endometrioid cystadenocarcinoma
2989291 0000061 13		International Federation of Gynecology and Obstetrics endometrial cancer (FIGO EC) stage II
2989301 0000061 14		International Federation of Gynaecology and Obstetrics endometrial cancer (FIGO EC) stage II

2989311000006112		International Federation of Gynaecology and Obstetrics endometrial cancer stage II
2989321000006116		International Federation of Gynecology and Obstetrics endometrial cancer stage II
2989331000006118		Mesothelioma, biphasic, malignant
299173011	F562100	Malignant positional vertigo
2991911000006111		Clear cell adenocarcinoma, mesonephroid
2991921000006115		Clear cell carcinoma
2991931000006117		Mesonephroid clear cell adenocarcinoma
2992261000006111		Noninfiltrating intraductal papillary adenocarcinoma
2992271000006116		Noninfiltrating intraductal papillary carcinoma
2992281000006118		Intraductal papillary adenocarcinoma
2992291000006115		Intraductal papillary carcinoma
2992301000006119		Ductal carcinoma in situ, papillary
2992321000006112		Non-infiltrating intraductal papillary adenocarcinoma
2992331000006110		Non-infiltrating intraductal papillary carcinoma
299676019	G240000	Secondary malignant renovascular hypertension
299677011	G240z00	Secondary malignant hypertension NOS
3001921000006115		Vipoma, malignant
3006031000006111		Mucocarcinoid tumor, malignant
3006041000006118		Mucocarcinoid tumour, malignant

3009761000006112		Intracavitary chemotherapy for malignant neoplasm
3019621000006112		Sweat gland carcinoma
3019631000006110		Sweat gland tumor, malignant
3019641000006117		Sweat gland tumour, malignant
3023731000006114		Extra-adrenal paraganglioma, malignant
3023741000006116		Nonchromaffin paraganglioma, malignant
3023751000006119		Malignant non-chromaffin paraganglioma
3023761000006117		Malignant extra-adrenal paraganglioma
3029921000006111		Medullary adenocarcinoma
3030841000006117		Inflammatory carcinoma
3030851000006115		Inflammatory adenocarcinoma
3033861000006112		Haemangioendothelioma, malignant
3033871000006117		Hemangioendothelioma, malignant
3033901000006117		Malignant haemangioendothelioma
3033911000006119		Malignant hemangioendothelioma
3039111000006119		Excision of malignant tumour of rectum by transanal approach
3039121000006110		Excision of malignant tumor of rectum by transanal approach
30425100000006114	BB5V300	[M]Acidophil carcinoma
30427100000006116	BBA2.00	Acinar cell carcinoma

304481000006119	BBZG.11	[M]Adamantinoma, malignant
304521000006119	BB5N100	[M]Adenocarcinoma in adenomatous polposis coli
304531000006116	BB5L100	Adenocarcinoma in situ in a polyp
304551000006111	BB51.00	Adenocarcinoma in situ
304621000006115	BBB5.00	[M]Adenocarcinoma with apocrine metaplasia
304631000006117	BBB3.00	[M]Adenocarcinoma with cartilaginous and osseous metaplasia
304641000006110	BBB4.00	[M]Adenocarcinoma with spindle cell metaplasia
304651000006112	BBB2.00	Adenocarcinoma with squamous metaplasia
304661000006114	BB57.00	Adenocarcinoma - intestinal type
304681000006116	BB5..11	[M]Adenocarcinomas
304701000006118	BB5J.00	Adenoid cystic carcinoma
304711000006115	BB2G.00	[M]Adenoid squamous cell carcinoma
304771000006112	BB5N.11	[M]Adenoma or or adenocarcinoma in polyposis coli
304791000006113	BB5..00	Adenoma AND/OR adenocarcinoma
304921000006113	BBB0.00	Adenosquamous carcinoma
304941000006118	BB6..00	[M]Adnexal and skin appendage neoplasm
305011000006117	BB5h100	Adrenal cortical carcinoma
305171000006110	BB5B511	[M]Alpha-cell tumour,malignant
305181000006113	BB5S400	[M]Alveolar adenocarcinoma

30520100000611 4	BB5S21 1	[M]Alveolar cell carcinoma
30529100000611 9	BBZG.0 0	Malignant ameloblastoma
3053291000006116		Fibrous histiocytoma, malignant
3053301000006115		Fibroxanthoma, malignant
30533100000611 4	BBC610 0	[M]Androblastoma, malignant
30557100000611 7	BB6210 0	[M]Apocrine adenocarcinoma
3056071000006119		International Federation of Gynaecology and Obstetrics endometrial cancer (FIGO EC) stage IV
3056081000006116		International Federation of Gynecology and Obstetrics endometrial cancer (FIGO EC) stage IV
3056091000006118		International Federation of Gynecology and Obstetrics endometrial cancer stage IV
30565100000611 1	BBC611 1	[M]Arrhenoblastoma, malignant
30579100000611 2	BB34.00	Fibroepithelial basal cell carcinoma
30580100000611 3	BB33.00	Basal cell carcinoma - morphoeic
30584100000611 0	BB48.00	Basaloid carcinoma
30586100000611 4	BB5V70 0	Basophil carcinoma
30590100000611 9	BB35.00	Basosquamous carcinoma
30592100000611 2	BB5B31 1	[M]Beta-cell tumour, malignant
30594100000611 7	BB5D11 1	Bile duct carcinoma
30595100000611 5	BB5D30 0	[M]Bile duct cystadenocarcinoma
30597100000611 3	BB5D.1 1	[M]Biliary tract adenomas and adenocarcinomas

3060910 0000611 5	BB M0 100	[M]Brenner tumour, malignant
3061310 0000611 8	BB5 S21 2	[M]Bronchiolar carcinoma
3062110 0000611 8	BB9 B.1 1	[M]C cell carcinoma
3063610 0000611 3	BB1 1.00	[M]Carcinoma in situ NOS
3063810 0000611 5	BB5 Q.0 0	[M]Carcinoma simplex
3064010 0000611 5	BB5 8.00	[M]Carcinoma, diffuse type
3065710 0000611 3	BB6 A10 0	[M]Ceruminous adenocarcinoma
3066410 0000611 6	BB5 D10 0	Cholangiocarcinoma
3066810 0000611 0	BB W8. 00	[M]Chondroblastoma, malignant
3067781 0000061 11		Infiltrating duct and lobular carcinoma
3067791 0000061 14		Infiltrating duct and lobular carcinoma in situ
3067801 0000061 10		Lobular and ductal carcinoma
3067811 0000061 13		Intraductal and lobular carcinoma
3067821 0000061 17		Infiltrating lobular carcinoma and ductal carcinoma in situ
3068210 0000611 0	BBR 3.00	[M]Choriocarcinoma combined with teratoma
3069010 0000611 1	BB5 V10 0	Chromophobe carcinoma
3070710 0000611 8	BB4 9.00	[M]Cloacogenic carcinoma
3070910 0000611 7	BB8 211 1	[M]Colloid adenocarcinoma
3071310 0000611 5	BB9 2.00	[M]Comedocarcinoma, noninfiltrating

30727100000006112	BB5K.00	[M]Cribriform carcinoma
30728100000006110	BB5J.11	[M]Cylindroid adenocarcinoma
30744100000006115	BBM9.00	Malignant cystosarcoma phyllodes
307510100000006117		Squamous cell carcinoma, small cell, nonkeratinising
307511100000006119		Squamous cell carcinoma, small cell, nonkeratinizing
307512100000006110		Epidermoid carcinoma, small cell, nonkeratinizing
307513100000006113		Epidermoid carcinoma, small cell, nonkeratinising
307514100000006115		Non-keratinising epidermoid carcinoma - small cell
307515100000006118		Non-keratinising squamous cell carcinoma - small cell
307516100000006116		Non-keratinizing squamous cell carcinoma - small cell
307517100000006111		Non-keratinizing epidermoid carcinoma - small cell
30753100000006112	BBQ9.00	[M]Dermoid cyst with malignant transformation
30763100000006111	BB91.11	Duct carcinoma
307842100000006110		Adenocarcinoma, no subtype
30792100000006115	BB5j500	[M]Endometrioid adenofibroma, malignant
307941100000006111		Excision of malignant lesion of face and ears
30797100000006119	BB5j200	Endometrioid carcinoma
30803100000006115	BB5V311	[M]Eosinophil carcinoma
30810100000006110	BB29.11	[M]Epidermoid carcinoma in situ

308111000006113	BB2A.11	Epidermoid carcinoma
308121000006117	BB2C.11	[M]Epidermoid carcinoma, keratinising type
308261000006116	BBP5.00	Malignant epithelioid mesothelioma
308291000006112	BB16.00	[M]Epithelioma, malignant
308371000006119	BBD8.00	[M]Extra-adrenal paraganglioma, malignant
308520100000006117		Alveolar adenocarcinoma
308521100000006119		Alveolar carcinoma
308671000006113	BBGF.00	Malignant fibrous histiocytoma
308701000006114	BBP3.00	[M]Fibrous mesothelioma, malignant
308731000006118	BBGJ.00	Malignant fibroxanthoma
308761000006110	BB5f300	Follicular adenocarcinoma - trabecular
308771000006115	BB5f200	[M]Follicular adenocarcinoma, well differentiated type
308821000006111	BB5C111	[M]G cell tumour, malignant
308941000006114	BB5C100	Malignant gastrinoma
308951000006111	BB82112	[M]Gelatinous adenocarcinoma
309061000006116	BB1B.00	[M]Giant cell and spindle cell carcinoma
309081000006114	BB1C.00	[M]Giant cell carcinoma
309131000006114	BBX1.00	Malignant giant cell tumour of bone
309281000006119	BBb0.00	Malignant glioma

30940100000611 2	BB5 B50 0	Malignant glucagonoma
30947100000611 8	BB5 b.00	[M]Granular cell carcinoma
30950100000611 3	BBf 1.00	[M]Granular cell tumour, malignant
30955100000611 2	BBC 4.00	Malignant granulosa cell tumour
30964100000611 1	BBT 710 0	[M]Haemangioendothelioma, malignant
30973100000611 0	BBT D20 0	[M]Haemangiopericytoma, malignant
30985100000611 4	BB5 D71 1	[M]Hepatocholangiocarcinoma
30989100000611 5	BB5 D51 2	[M]Hepatoma, malignant
3101101000006116		Mixed basal-squamous cell carcinoma
3101111000006118		Mixed basal - squamous cell carcinoma
3101121000006114		Basisquamous cell carcinoma
31016100000611 0	BB5 W1 11	Hurthle cell adenocarcinoma
31027100000611 0	BBQ 4.11	[M]Infantile embryonal carcinoma
31031100000611 0	BB9 1.00	Infiltrating duct carcinoma
31032100000611 9	BB9 G.0 0	Infiltrating ductular carcinoma
31034100000611 4	BB9 H.0 0	[M]Inflammatory carcinoma
31036100000611 3	BB5 B30 0	Malignant insulinoma
31039100000611 7	BBC C11 1	[M]Interstitial cell tumour, malignant
31041100000611 7	BB9 M.0 0	[M]Intracystic carcinoma NOS

31045100000611 6	BB90.00	[M]Intraductal carcinoma, non-infiltrating NOS
31049100000611 0	BB29.12	Intraepidermal carcinoma
31053100000611 0	BB11.11	[M]Intraepithelial carcinoma NOS
31054100000611 7	BB29.13	Intraepithelial squamous cell carcinoma
31057100000611 3	BBZ2.11	[M]Intraosseous carcinoma
31066100000611 7	BB5B100	[M]Islet cell carcinoma
31074100000611 0	BB94.00	Juvenile carcinoma of the breast
31109100000611 0	BBCC100	Malignant Leydig cell tumour
3111611000006112		Progressive malignant polyserositis
31121100000611 2	BB5D513	[M]Liver cell carcinoma
31122100000611 6	BB9E.00	Lobular carcinoma in situ
31139100000611 3	BB2M.00	[M]Lymphoepithelial carcinoma
31146100000611 1	BBk..00	Malignant lymphoma, follicular AND/OR nodular
31156100000611 2	BBk5.00	[M]Malignant lymphoma, follicular centre cell, cleaved, follicular
31157100000611 7	BBk4.00	[M]Malignant lymphoma, lymphocytic, intermediate differentiation, nodular
31158100000611 9	BBk6.00	[M]Malignant lymphoma, lymphocytic, poorly differentiated, nodular
31160100000611 2	BBgK.00	[M]Malignant lymphoma, follicular centre cell, non-cleaved NOS
31161100000611 0	BBgD.00	[M]Malignant lymphoma, lymphocytic, intermediate differentiation NOS
31162100000611 9	BBk3.00	[M]Malignant lymphoma, lymphocytic, well differentiated, nodular

31163100000061 16	BBk1.00	[M]Malignant lymphoma, mixed lymphocytic-histiocytic, nodular
31164100000061 14	BBgN.00	[M]Malignant lymphoma, lymphocytic, intermediate differentiation, diffuse
31165100000061 11	BBX3.00	[M]Malignant giant cell tumour of soft parts
31167100000061 18	BBg1.00	Malignant lymphoma
31180100000061 13	BBgT.00	[M]Malignant lymphoma, large cell, noncleaved, diffuse
31181100000061 11	BBgG.00	[M]Malignant lymphoma, lymphocytic, poorly differentiated NOS
31187100000061 19	BBg2.00	[M]Malignant lymphoma, non-Hodgkin's type
31188100000061 16	BBgV.00	[M]Malignant lymphoma, small cell, noncleaved, diffuse
31191100000061 16	BBg4.00	[M]Malignant lymphoma, stem cell type
31195100000061 15	BBEM.00	[M]Malignant melanoma in giant pigmented naevus
31197100000061 13	BBEC.00	[M]Malignant melanoma in junctional naevus
31198100000061 11	BBEE.00	[M]Malignant melanoma in precancerous melanosis
31202100000061 18	BBQ7500	[M]Malignant teratoma, intermediate type
31203100000061 15	BBR4.00	[M]Malignant teratoma, trophoblastic
31204100000061 13	BBQ7400	Malignant teratoma - undifferentiated
31205100000061 10	BB0A.00	[M]Malignant tumour, fusiform cell type
31206100000061 12	BB09.00	[M]Malignant tumour, giant cell type
3120681000006117		Undifferentiated carcinoma
31207100000061 17	BB08.00	Malignant tumour - small cell type

31216100000611 1	BB9C.00	Medullary carcinoma with amyloid stroma
31217100000611 6	BB9D.00	[M]Medullary carcinoma with lymphoid stroma
31225100000611 8	BBE1.11	[M]Melanocarcinoma
31236100000611 4	BBd2.00	[M]Meningioma, malignant
31246100000611 5	BBLC100	[M]Mesenchymoma, malignant
31253100000611 1	BBS2.00	Malignant mesonephroma
31259100000611 0	BBP7.00	Malignant biphasic mesothelioma
31260100000611 9	BBP1.00	Mesothelioma, malignant
31262100000611 2	BB85100	Metastatic signet ring cell carcinoma
31263100000611 0	BB36.00	[M]Metatypical carcinoma
3126931000006110		Mixed cell adenocarcinoma
31273100000611 8	BBmz.00	[M]Miscellaneous reticuloendothelial neoplasm NOS
31274100000611 1	BBm..00	[M]Miscellaneous reticuloendothelial neoplasms
31278100000611 7	BB5V500	[M]Mixed acidophil-basophil carcinoma
31280100000611 8	BB5d100	[M]Mixed cell adenocarcinoma
3128681000006119		Papillary epidermoid carcinoma
31288100000611 0	BB5B600	[M]Mixed islet cell and exocrine adenocarcinoma
31302100000611 4	BB...00	[M]Morphology of neoplasms
31303100000611 2	BB84.00	[M]Mucin-producing adenocarcinoma

3130410 0000611 9	BB8 210 0	Mucinous adenocarcinoma
3131310 0000611 3	BB7 1.00	Mucoepidermoid carcinoma
3131910 0000611 2	BB5 V71 1	[M]Mucoid cell carcinoma
3132010 0000611 0	BB8 211 4	[M]Mucous adenocarcinoma
3132210 0000611 7	BB3 2.00	[M]Multicentric basal cell carcinoma
3136341 0000061 19		Excision of malignant lesion of scalp and neck
3136510 0000611 2	BBE 1.14	[M]Naevocarcinoma
3136621 0000061 17		Tumor cells, uncertain whether benign or malignant
3136910 0000611 8	BB0 4.00	Malignant tumour - uncertain whether primary or metastatic
3137010 0000611 8	BB0 2.00	Malignant neoplasm
3138010 0000611 4	BBe 7.00	[M]Neurilemmoma, malignant
3140010 0000611 8	BBy 1.00	[M]No microscopic confirmation of tumour, clinically malignant
3140710 0000611 2	BB5 f70 0	[M]Nonencapsulated sclerosing carcinoma
3140810 0000611 0	BB9 8.00	[M]Noninfiltrating intracystic carcinoma
3140910 0000611 3	BB9 6.00	[M]Noninfiltrating intraductal papillary adenocarcinoma
3141110 0000611 6	BB1 K.00	Oat cell carcinoma
3142010 0000611 2	BBZ 2.00	Malignant odontogenic tumour
3142071 0000061 12		Malignant melanoma, regressing
3143110 0000611 8	BB5 W1 12	[M]Oncytic adenocarcinoma

3144210 0000611 5	BBX 1.12	[M]Osteoclastoma, malignant
3145510 0000611 3	BB8 1.11	[M]Ovarian cystadenoma or carcinoma
3146210 0000611 7	BB5 W1 00	Oxyphilic adenocarcinoma
3146710 0000611 6	BB9 K.00	Paget's disease and infiltrating duct carcinoma of breast
3147910 0000611 0	BB5 f60 0	Papillary and follicular adenocarcinoma
3148110 0000611 4	BB2 1.00	Papillary carcinoma in situ
3148910 0000611 6	BB2 6.11	[M]Papillary epidermoid carcinoma
3149210 0000611 0	BB8 1H0 0	[M]Papillary mucinous cystadenocarcinoma
3149710 0000611 1	BB8 180 0	[M]Papillary serous cystadenocarcinoma
3150110 0000611 9	BB2 6.00	Papillary squamous cell carcinoma
3150310 0000611 3	BB4 A.0 0	Papillary transitional cell carcinoma
3150391 0000061 14		Malignant lymphoma, large cell, follicular
3150401 0000061 11		Malignant lymphoma, large cell, noncleaved, follicular
3150411 0000061 14		Malignant lymphoma, histiocytic, nodular
3150421 0000061 18		Malignant lymphoma, large cleaved cell, follicular
3150431 0000061 15		Malignant lymphoma, noncleaved, follicular
3150441 0000061 13		Malignant lymphoma, lymphocytic, well differentiated, nodular [obs]
3150451 0000061 10		Malignant lymphoma, centroblastic, follicular
3150710 0000611 1	BB9 B.1 2	[M]Parafollicular cell carcinoma

3151010 0000611 8	BBD 1.00	Malignant paraganglioma
3151251 0000061 18		Malignant mixed tumour, carcinomatous type
3151261 0000061 16		Malignant mixed tumor, carcinomatous type
3152510 0000611 0	BBD A.0 0	Malignant phaeochromocytoma
3154410 0000611 4	BBn 3.00	[M]Plasma cell tumour, malignant
3155010 0000611 3	BB1 A.0 0	Pleomorphic carcinoma
3156210 0000611 6	BBQ 5.11	[M]Polyembryonal embryonal carcinoma
3156310 0000611 8	BB1 F.00	[M]Polygonal cell carcinoma
3157410 0000611 7	BB8 1E1 1	[M]Pseudomucinous adenocarcinoma
3157710 0000611 3	BB1 E.00	[M]Pseudosarcomatous carcinoma
3158610 0000611 1	BB5 a00 0	Renal cell carcinoma - morphology
3158810 0000611 8	BB1 J.11	[M]Reserve cell carcinoma
3161110 0000611 4	BB1 J.12	[M]Round cell carcinoma
3161810 0000611 9	BB4 6.00	[M]Schneiderian carcinoma
3162110 0000611 5	BBe 7.11	[M]Schwannoma, malignant
3162210 0000611 1	BB5 4.00	[M]Scirrhous adenocarcinoma
3162910 0000611 3	BB1 3.11	[M]Secondary carcinoma
3163010 0000611 4	BB0 3.11	[M]Secondary neoplasm
3163110 0000611 2	BB9 4.11	[M]Secretory breast carcinoma

31640100000611 7	BB81B0 0	Serous surface papillary carcinoma
31644100000611 5	BBCA.0 0	[M]Sertoli cell carcinoma
31651100000611 8	BB85.00	Signet ring carcinoma
31653100000611 2	BB8500 0	Signet ring cell carcinoma
31659100000611 1	BB1L.00	[M]Small cell carcinoma, fusiform cell type
31669100000611 0	BB9C.11	[M]Solid carcinoma with amyloid stroma
31676100000611 1	BB1D.0 0	Spindle cell carcinoma
31683100000611 0	BB2A.1 2	[M]Spinous cell carcinoma
31693100000611 7	BB2D.0 0	[M]Squamous cell carcinoma, large cell, non-keratinising
31695100000611 2	BB2J.00	Microinvasive squamous cell carcinoma
31696100000611 4	BB2E.00	[M]Squamous cell carcinoma, small cell, non-keratinising
31697100000611 9	BB2F.00	[M]Squamous cell carcinoma, spindle cell type
31704100000611 1	BBQA10 0	[M]Struma ovarii, malignant
3170631000006119		Renal cell carcinoma
3170641000006112		Renal cell adenocarcinoma
31719100000611 8	BB56.00	[M]Superficial spreading adenocarcinoma
31742100000611 2	BBQ721 3	[M]Teratoblastoma, malignant
31743100000611 0	BBQ730 0	[M]Teratocarcinoma
3175581000006112		Secretory carcinoma of the breast

31761100000611 9	BBB 610 0	Malignant thymoma
31765100000611 8	BB5 F.00	[M]Trabecular adenocarcinoma
31767100000611 1	BB4 2.00	Transitional cell carcinoma in situ
31769100000611 2	BB4 7.00	[M]Transitional cell carcinoma, spindle cell type
31773100000611 6	BB4 ..00	Transitional cell papilloma AND/OR carcinoma
31782100000611 4	BB5 M1 00	Tubular adenocarcinoma
31790100000611 0	BB0 7.00	Malignant tumour cells
31800100000611 3	BB4 3.11	Urothelial carcinoma
3180111000006111		Brenner tumour, malignant
3180121000006115		Brenner tumor, malignant
3180131000006117		Malignant Brenner tumour
3180141000006110		Malignant Brenner tumor
31805100000611 2	BB2 4.11	[M]Verrucous epidermoid carcinoma
31808100000611 6	BB2 4.12	[M]Verrucous squamous cell carcinoma
31810100000611 2	BB5 U20 0	[M]Villous adenocarcinoma
31819100000611 7	BB5 c20 0	[M]Water-clear cell adenocarcinoma
31823100000611 0	BBS 2.11	[M]Wolffian duct carcinoma
3186671000006113		Giant cell carcinoma
3196591000006114		Adenocarcinoma in adenomatous polyp

3196601000006118		Carcinoma in adenomatous polyp
3196611000006115		Adenocarcinoma in tubular adenoma
3196621000006111		Adenocarcinoma in polypoid adenoma
3196631000006114		Adenocarcinoma in a polyp
3196641000006116		Carcinoma in a polyp
3198781000006117		Basal cell carcinoma, fibroepithelial
3198821000006111		Fibroepithelial basal cell carcinoma, Pinkus type
3209361000006119		Malignant endocarditis
3215371000006114		Tis: Carcinoma in situ
3216591000006111		Malignant melanoma in Hutchinson's melanotic freckle
3216611000006117		Malignant melanoma in Hutchinson melanotic freckle
3221981000006119		Choriocarcinoma
3222041000006117		Choriocarcinoma, no ICD-O subtype
3222051000006115		Choriocarcinoma, no International Classification of Diseases for Oncology subtype
3232761000006118		Acinic cell adenocarcinoma
3232771000006113		Acinar adenocarcinoma
3232781000006111		Acinar carcinoma
3234021000006117		Squamous cell carcinoma, large cell, nonkeratinising
3234031000006119		Squamous cell carcinoma, large cell, nonkeratinizing

3234041 0000061 12		Epidermoid carcinoma, large cell, nonkeratinizing
3234051 0000061 14		Squamous cell carcinoma, nonkeratinizing
3234061 0000061 11		Epidermoid carcinoma, large cell, nonkeratinising
3234071 0000061 16		Squamous cell carcinoma, nonkeratinising
3234081 0000061 18		Non-keratinising squamous cell carcinoma
3234091 0000061 15		Non-keratinising epidermoid carcinoma - large cell
3234101 0000061 14		Non-keratinising squamous cell carcinoma - large cell
3234111 0000061 12		Non-keratinizing squamous cell carcinoma - large cell
3234121 0000061 16		Non-keratinizing squamous cell carcinoma
3234131 0000061 18		Non-keratinizing epidermoid carcinoma - large cell
3240901 0000061 16		Carcinoma simplex
3244801 0000061 16		Excision of malignant tumour of breast
3244811 0000061 18		Excision of malignant tumor of breast
3246841 0000061 10		Prescription for alteration of hormonal balance for cancer
3247840 19	SN5 y00 0	Malignant hyperpyrexia due to anaesthetic
3254991 0000061 17		Malignant lymphoma, large B-cell, diffuse
3255001 0000061 12		Malignant lymphoma, histiocytic, diffuse
3255011 0000061 10		Malignant lymphoma, large cell, cleaved and noncleaved
3255021 0000061 19		Malignant lymphoma, large cell

3255031000006116		Malignant lymphoma, histiocytic
3255041000006114		Malignant lymphoma, noncleaved, diffuse
3255051000006111		Malignant lymphoma, noncleaved
3255061000006113		Malignant lymphoma, large cleaved cell
3255071000006118		Malignant lymphoma, large cell, noncleaved, diffuse
3255081000006115		Malignant lymphoma, large cell, noncleaved
3255091000006117		Malignant lymphoma, large cell, diffuse [obs]
3255101000006111		Malignant lymphoma, large cell, cleaved, diffuse
3255111000006114		Malignant lymphoma, large cell, cleaved
3255121000006118		Malignant lymphoma, large B-cell, diffuse, centroblastic
3255131000006115		Malignant lymphoma, large B-cell
3255141000006113		Malignant lymphoma, centroblastic, diffuse
3255151000006110		Malignant lymphoma, centroblastic
3255231000006114		Malignant lymphoma, large B-cell, diffuse, no ICD-O subtype
3255241000006116		Malignant lymphoma, large B-cell, diffuse, no International Classification of Diseases for Oncology subtype
3255431000006113		Malignant lymphoma, small cleaved cell, follicular
3255441000006115		Malignant lymphoma, lymphocytic, poorly differentiated, nodular [obs]
3255611000006117		International Federation of Gynaecology and Obstetrics endometrial cancer (FIGO EC) stage I A
3255621000006113		International Federation of Gynecology and Obstetrics endometrial cancer (FIGO EC) stage I A

3255631 0000061 11		International Federation of Gynecology and Obstetrics endometrial cancer stage I A
3261731 0000061 12		Basophil adenocarcinoma
3261741 0000061 19		Mucoid cell adenocarcinoma
3264681 0000061 17		Adenocarcinoma in multiple adenomatous polyps
3271601 0000061 19		Malignant hypercalcaemia
3271621 0000061 12		Malignant hypercalcemia
3284101 0000061 16		Malignant giant cell tumour of soft parts
3284111 0000061 18		Malignant giant cell tumor of soft parts
3284121 0000061 14		Malignant giant cell tumour of soft tissue
3284131 0000061 12		Malignant giant cell tumor of soft tissue
3285731 0000061 12		Carcinoid tumor, argentaffin, malignant
3285741 0000061 19		Argentaffinoma, malignant
3285771 0000061 10		Carcinoid tumour, argentaffin, malignant
3285791 0000061 11		Malignant carcinoid tumour - argentaffin
3285801 0000061 12		Malignant carcinoid tumor - argentaffin
3285811 0000061 10		Malignant argentaffinoma
3296311 0000061 13		International Federation of Gynaecology and Obstetrics cervical cancer (FIGO CC) stage Ia
3296321 0000061 17		International Federation of Gynecology and Obstetrics cervical cancer (FIGO CC) stage Ia
3296341 0000061 12		International Federation of Gynecology and Obstetrics cervical cancer stage Ia

3311411000006118		Malignant lymphoma, mixed small and large cell, diffuse
3311421000006114		Malignant lymphoma, mixed lymphocytic-histiocytic, diffuse
3311431000006112		Malignant lymphoma, mixed cell type, diffuse
3311441000006119		Malignant lymphoma, centroblastic-centrocytic, diffuse [obs]
3311451000006117		Malignant lymphoma, centroblastic-centrocytic [obs]
3312281000006117		Malignant mastocytosis
3313751000006112		International Federation of Gynecology and Obstetrics endometrial cancer (FIGO EC) stage I
3313761000006114		International Federation of Gynaecology and Obstetrics endometrial cancer (FIGO EC) stage I
3313771000006119		International Federation of Gynecology and Obstetrics endometrial cancer stage I
3316471000006115		Bile duct cystadenocarcinoma
3319581000006113		Malignant catarrh virus of cattle
3319591000006111		Malignant catarrhal fever virus
3330021000006115		Acidophil carcinoma
3330031000006117		Eosinophil adenocarcinoma
3330041000006110		Acidophil adenocarcinoma
3330051000006112		Eosinophil carcinoma
3333861000006113		Mixed adenoneuroendocrine carcinoma
3333881000006115		Combined carcinoid and adenocarcinoma
3333891000006117		Mixed carcinoid-adenocarcinoma

3335011 0000061 17		Oral chemotherapy for malignant neoplasm
3336471 0000061 14		Adenocarcinoma in situ in tubulovillous adenoma
3336911 0000061 14		AIS - Adenocarcinoma in situ
3338911 0000061 10		Desmoplastic melanoma, malignant
3338921 0000061 19		Neurotropic melanoma, malignant
3338941 0000061 14		Malignant neurotropic melanoma
3345541 0000061 14		Pleurodesis with cancer chemotherapy substance
3345711 0000061 15		Combined hepatocellular carcinoma and cholangiocarcinoma
3345721 0000061 11		Mixed hepatocellular and bile duct carcinoma
3345731 0000061 14		Hepatocholangiocarcinoma
3346441 0000061 19		Malignant megakaryocytosis
3354601 0000061 12		International Federation of Gynecology and Obstetrics cervical cancer (FIGO CC) stage III
3354611 0000061 10		International Federation of Gynaecology and Obstetrics cervical cancer (FIGO CC) stage III
3354621 0000061 19		International Federation of Gynecology and Obstetrics cervical cancer stage III
3368051 0000061 15		Urothelial carcinoma in situ
3368971 0000061 12		Ovine pulmonary adenocarcinoma virus
3377951 0000061 14		Epithelioid haemangioendothelioma, malignant
3377961 0000061 11		Epithelioid hemangioendothelioma, malignant
3387071 0000061 14		Paget's disease and intraductal carcinoma of breast

3387081000006112		Paget disease and intraductal carcinoma of breast
3388321000006112		Sebaceous adenocarcinoma
3388331000006110		Sebaceous carcinoma
3393061000006110		Malignant lymphoma, mixed small cleaved and large cell, follicular
3393071000006115		Malignant lymphoma, mixed lymphocytic-histiocytic, nodular
3393081000006117		Malignant lymphoma, mixed cell type, follicular
3393091000006119		Malignant lymphoma, mixed cell type, nodular
3395261000006118		Malignant lymphoma, nodular
3395271000006113		Malignant lymphoma, lymphocytic, nodular
3395281000006111		Malignant lymphoma, follicle center, follicular
3395291000006114		Malignant lymphoma, follicle center
3395301000006110		Malignant lymphoma, centroblastic-centrocytic, follicular
3395311000006113		Malignant lymphoma, follicular
3395321000006117		Malignant lymphoma, follicle centre
3395331000006119		Malignant lymphoma, follicle centre, follicular
3398781000006111		Malignant astrocytoma
340421000006119	ZV67900	[V]Follow-up examination after combined treatment for malignant neoplasm
340471000006118	ZV67700	[V]Follow-up examination after radiotherapy for malignant neoplasm
340491000006117	ZV67800	[V]Follow-up examination after chemotherapy for malignant neoplasm

34054100000611 0	ZV67A0 0	[V]Follow-up examination after other treatment for malignant neoplasm
34055100000611 2	ZV6B.0 0	[V]Follow-up examination after treatment for conditions other than malignant neoplasms
34056100000611 4	ZV67B0 0	[V]Follow-up examination after unspecified treatment for malignant neoplasm
3407731000006115		Pericarditis secondary to malignant primary tumour
3407741000006113		Pericarditis secondary to malignant primary tumor
3408821000006112		Neuroendocrine carcinoma
3417691000006113		Adenocarcinoma with cartilaginous and osseous metaplasia
3417701000006113		Adenocarcinoma with cartilaginous metaplasia
3417711000006111		Adenocarcinoma with osseous metaplasia
3419261000006110		International Federation of Gynecology and Obstetrics vulvar carcinoma (FIGO VC) stage I
3419271000006115		International Federation of Gynaecology and Obstetrics vulvar carcinoma (FIGO VC) stage I
3419281000006117		International Federation of Gynecology and Obstetrics vulvar carcinoma stage I
3420501000006112		Infiltrating basal cell carcinoma, sclerosing
3420511000006110		Infiltrating basal cell carcinoma, non-sclerosing
3420521000006119		Basal cell carcinoma, morpheic
3420531000006116		Basal cell carcinoma, desmoplastic type
342807011	B150300	Hepatocellular carcinoma
342834014	B150100	Hepatoblastoma of liver
342851019	B150200	Primary angiosarcoma of liver

3428621 0000061 17		Apocrine adenocarcinoma
3434561 0000061 19		Adenocarcinoma in adenomatous polyposis coli
3435751 0000061 14		Oncocytic carcinoma
3435761 0000061 11		Oncocytic adenocarcinoma
3435771 0000061 16		Hurthle cell carcinoma
3435791 0000061 15		Follicular carcinoma, oxyphilic cell
3436161 0000061 17		Thecoma, malignant
3436171 0000061 12		Malignant thecoma
3443161 0000061 12		Ceruminous adenocarcinoma
3443171 0000061 17		Ceruminous carcinoma
3444510 0000611 5	ZV1 041 3	[V]Personal history of malignant neoplasm of male genital organ
3446351 0000061 15		Anaplastic carcinoma
3446570 18	B62 x00 0	T-zone lymphoma
3446700 10	B62 x10 0	Lymphoepithelioid lymphoma
3446711 0000061 15		Malignant malnutrition
3447610 0000611 3	ZV1 000 0	[V]Personal history of malignant neoplasm of gastrointestinal tract
3447810 0000611 5	ZV1 010 0	[V]Personal history of malignant neoplasm of trachea, bronchus or lung
3447910 0000611 7	ZV1 020 0	[V]Personal history of malignant neoplasm of other respiratory or intrathoracic organ
3448060 15	B62 x60 0	True histiocytic lymphoma
3448180 12	B63 0.11	Kahler's disease

344819016	B630.12	Myelomatosis
344820010	B630.00	Multiple myeloma
344846014	B675.00	Acute myelofibrosis
34490100000611 0	ZV10400	[V]Personal history of malignant neoplasm of genital organ
34493100000611 9	ZV10512	[V]Personal history of malignant neoplasm of kidney
34510100000611 4	ZV10y16	[V]Personal history of malignant neoplasm of tongue
34578100000611 6	ZV10700	[V]Personal history other lymphatic/haematopoietic neoplasm
3457851000006113		Malignant granuloma of face
3464151000006115		Mixed adenocarcinoma and squamous cell carcinoma
3464161000006118		Mixed adenocarcinoma and epidermoid carcinoma
3466821000006112		Squamous cell carcinoma in situ
3466841000006117		Epidermoid carcinoma in situ
3466871000006113		IEC - Intraepidermal carcinoma
3466881000006111		Squamous cell carcinoma in situ, no ICD-O subtype
3466891000006114		Squamous cell carcinoma in situ, no International Classification of Diseases for Oncology subtype
3478931000006114		International Federation of Gynaecology and Obstetrics endometrial cancer (FIGO EC) stage I B
3478941000006116		International Federation of Gynecology and Obstetrics endometrial cancer (FIGO EC) stage I B
3478951000006119		International Federation of Gynecology and Obstetrics endometrial cancer stage I B
3479091000006118		Adenocarcinoma in situ in adenomatous polyp
3479101000006112		Adenocarcinoma in situ in tubular adenoma

3479111 0000061 10		Carcinoma in situ in adenomatous polyp
3479121 0000061 19		Adenocarcinoma in situ in polypoid adenoma
3479131 0000061 16		Carcinoma in situ in a polyp
3479981 0000061 17		Islet cell carcinoma
3479991 0000061 19		Islet cell adenocarcinoma
3480001 0000061 13		Pancreatic endocrine tumour, malignant
3480011 0000061 11		Pancreatic endocrine tumor, malignant
3492821 0000061 13		Multicentric basal cell carcinoma
3496291 0000061 10		Cancer-related substance
3508581 0000061 16		Mesothelioma, malignant
3508601 0000061 14		Malignant mesothelioma
3512019 011		Malignant pigmented skin lesion
3513951 019		History of carcinoma in situ of vulva
3514363 013		Microsatellite instability-high solid malignant tumor
3514364 019		Microsatellite instability-high solid malignant tumour
3514365 018		dMMR cancer
3514366 017		dMMR solid malignant tumor
3514368 016		MSI-H cancer
3514369 012		MSI-H solid malignant tumor
3514565 017		Primary malignant neuroepitheliomatous neoplasm of nasal cavity
3515311 012		Primary malignant neuroendocrine neoplasm of pancreas
3515711 010		Primary malignant neuroepithelial neoplasm of retina
3515715 018		Primary malignant neoplasm of lacrimal caruncle
3515717 014		Primary malignant neuroepithelial neoplasm of iris

3515719012		Primary malignant neuroepithelial neoplasm of ciliary body
3515721019		Primary malignant neuroepithelial neoplasm of orbit
3515749014		Chronic pain due to malignant neoplastic disease
3515750014		Chronic cancer related pain
3515907014		Mismatch repair deficient solid malignant tumor
3515908016		Mismatch repair deficient solid malignant tumour
3516251016		Primary malignant neoplasm of ocular adnexa
3516361000006118		Malignant aortic body tumour
3516371000006113		Malignant aortic body tumor
3517999018		Excision of basal cell carcinoma from right postauricular region with closure
3519021000006116		Malignant tertian malaria
3519071000006115		MT - Malignant tertian malaria
3519101000006113		MTM - Malignant tertian malaria
3519261000006119		Nonencapsulated sclerosing carcinoma
3519271000006114		Nonencapsulated sclerosing adenocarcinoma
3519291000006110		Papillary carcinoma, diffuse sclerosing
3525998014		Adenocarcinoma of left lung
3526034015		Malignant melanoma of skin of right lower limb
3526066013		Transitional cell carcinoma of left ureter
3526496010		Mismatch repair deficient colorectal cancer
3526497018		Microsatellite instability-high colorectal cancer
3526498011		dMMR colorectal cancer
3526499015		MSI-H colorectal cancer
3527074017		Ductal comedocarcinoma in situ of breast
3527388010		Primary adenocarcinoma of sublingual gland

3527391 010		Primary adenocarcinoma of submandibular gland
3527392 015		Primary squamous cell carcinoma of submandibular gland
3527394 019		Primary squamous cell carcinoma of sublingual gland
3527396 017		Primary malignant neuroendocrine neoplasm of jejunum
3527399 012		Primary malignant neuroendocrine neoplasm of ileum
3527901 0000061 13		Synovioma, malignant
3530741 0000061 10		Lymphangioendothelioma, malignant
3530751 0000061 12		Malignant lymphangioendothelioma
3531622 018		Ductal carcinoma in situ of breast comedo subtype
3532634 012		Myeloid/lymphoid neoplasm associated with PDGFRA rearrangement
3532636 014		Myeloid and/or lymphoid neoplasm associated with platelet derived growth factor receptor alpha rearrangement
3537720 13	B57 620 0	Malignant ascites
3540651 0000061 15		Adnexal carcinoma
3544191 0000061 10		Squamous cell carcinoma, metastatic
3544201 0000061 13		Metastatic squamous cell carcinoma
3545210 13	K0J 1.00	Renal involvement in malignant disease
3549251 0000061 13		Intraductal papillary adenocarcinoma with invasion
3549261 0000061 10		Infiltrating and papillary adenocarcinoma
3549271 0000061 15		Infiltrating papillary adenocarcinoma
3550221 0000061 13		Malignant lymphoma, small lymphocytic
3550231 0000061 11		Malignant lymphoma, lymphocytic, diffuse
3550241 0000061 18		Malignant lymphoma, small cell

3550251 0000061 16		Malignant lymphoma, lymphocytic, well differentiated, diffuse
3550261 0000061 19		Malignant lymphoma, small lymphocytic, diffuse
3550271 0000061 14		Malignant lymphoma, lymphocytic
3550281 0000061 12		Malignant lymphoma, small cell diffuse
3550291 0000061 10		Malignant lymphoma, small B lymphocytic
3551461 0000061 10		Cancer immunotherapy
3561311 0000061 14		Epithelioid mesothelioma, malignant
3567381 0000061 10		Malignant teratoma, trophoblastic
3567391 0000061 13		Trophoblastic malignant teratoma
3568251 0000061 14		Polypoid squamous cell carcinoma
3581621 0000061 12		Glucagonoma, malignant
3581631 0000061 10		Alpha cell tumor, malignant
3581641 0000061 17		Alpha cell tumour, malignant
3581651 0000061 15		Malignant alpha cell tumour
3581661 0000061 18		Malignant alpha cell tumor
3591861 0000061 18		Blue naevus, malignant
3591871 0000061 13		Blue nevus, malignant
3591891 0000061 14		Malignant blue naevus
3591901 0000061 13		Malignant blue nevus

3592710<br>0000611<br>0	Byu<br>C50<br>0	Secondary malignant neoplasm of urinary system
3592810<br>0000611<br>3	Byu<br>C60<br>0	Secondary malignant neoplasm of nervous system
3592910<br>0000611<br>1	Byu<br>C20<br>0	Secondary malignant neoplasm of lymph nodes of multiple sites
3603191<br>0000061<br>13		Teratocarcinoma
3603201<br>0000061<br>11		Mixed embryonal carcinoma and teratoma
3610221<br>0000061<br>12		International Federation of Gynaecology and Obstetrics cervical cancer (FIGO CC) stage II
3610231<br>0000061<br>10		International Federation of Gynecology and Obstetrics cervical cancer (FIGO CC) stage II
3610241<br>0000061<br>17		International Federation of Gynecology and Obstetrics cervical cancer stage II
3612301<br>0000061<br>11		Adenocarcinoma with spindle cell metaplasia
3613891<br>0000061<br>17		Carcinoma
3613901<br>0000061<br>18		Epithelial tumor, malignant
3613911<br>0000061<br>15		Epithelial tumour, malignant
3613921<br>0000061<br>11		Malignant epithelial tumour
3613931<br>0000061<br>14		Malignant epithelial tumor
3613941<br>0000061<br>16		Carcinoma, no subtype
3619421<br>0000061<br>19		Spitzoid malignant melanoma
3620201<br>0000061<br>12		Papillary mucinous cystadenocarcinoma
3620211<br>0000061<br>10		Papillary pseudomucinous cystadenocarcinoma
3620221<br>0000061<br>19		Papillary pseudomucinous adenocarcinoma

3621421 0000061 11		Carcinoma in situ
3621431 0000061 14		Intraepithelial carcinoma
3621441 0000061 16		Carcinoma in situ morphology
3621451 0000061 19		Carcinoma in situ, no ICD-O subtype
3621461 0000061 17		Carcinoma in situ, no International Classification of Diseases for Oncology subtype
3622621 0000061 16		Granular cell carcinoma
3622631 0000061 18		Granular cell adenocarcinoma
3628711 0000061 19		Nevoid basal cell carcinoma syndrome
3628721 0000061 10		Basal cell carcinoma syndrome
3628781 0000061 14		NBCCS - Naevoid basal cell carcinoma syndrome
3628791 0000061 12		NBCCS - Nevoid basal cell carcinoma syndrome
3634392 015		Malignant peripheral nerve sheath tumour with perineurial differentiation
3634393 013		Malignant peripheral nerve sheath neoplasm with perineurial differentiation
3634394 019		Malignant peripheral nerve sheath tumor with perineurial differentiation
3634396 017		Malignant perineurioma
3636264 012		Pleomorphic lobular carcinoma in situ of breast
3636679 013		Endometrial endometrioid adenocarcinoma
3636681 010		Primary endometrioid carcinoma of endometrium of body of uterus
3636899 017		Chronic post cancer surgery pain
3636901 014		Chronic pain following surgical procedure for cancer
3637028 012		Primary malignant neuroendocrine neoplasm of perihilar bile duct
3641061 0000061 11		Bile duct adenocarcinoma
3641071 0000061 16		Cholangiocellular carcinoma

3644991 0000061 18		Polygonal cell carcinoma
3659761 0000061 12		Epithelioma, malignant
3659781 0000061 19		Malignant epithelioma
3671071 0000061 15		Myopathy associated with malignant disease
3674171 0000061 16		Follicular adenocarcinoma, trabecular
3674181 0000061 18		Follicular carcinoma, trabecular
3674191 0000061 15		Follicular adenocarcinoma, moderately differentiated
3674201 0000061 17		Follicular carcinoma, moderately differentiated
3674221 0000061 10		Follicular carcinoma - moderately differentiated
3674231 0000061 13		Follicular carcinoma - trabecular
3674241 0000061 15		Follicular adenocarcinoma - moderately differentiated
3675581 0000061 13		Non malignant mast cell disease
3678791 0000061 13		Mucous carcinoma
3678801 0000061 14		Mucinous carcinoma
3678811 0000061 12		Colloid adenocarcinoma
3678821 0000061 16		Colloid carcinoma
3678831 0000061 18		Gelatinous adenocarcinoma
3678851 0000061 13		Mucoid adenocarcinoma
3678861 0000061 10		Mucoid carcinoma

36788710000006115		Mucous adenocarcinoma
36834210000006118		International Federation of Gynecology and Obstetrics vulvar carcinoma (FIGO VC) stage IV
36834310000006115		International Federation of Gynaecology and Obstetrics vulvar carcinoma (FIGO VC) stage IV
36834410000006113		International Federation of Gynecology and Obstetrics vulvar carcinoma stage IV
36871010000006113		Giant cell and spindle cell carcinoma
370111000006119	ByuFC00	[X]Carcinoma in situ of oth+unspecified male genital organs
370161000006116	ByuFB00	[X]Carcinoma in situ of other+unspcfd female genital organs
370171000006111	ByuFD00	Carcinoma in situ of urinary tract proper
370201000006110	ByuF700	[X]Carcinoma in situ of skin of other+unspecified parts/face
370221000006117	ByuF000	Carcinoma in situ of intestinal tract
37037710000006117		Small cell carcinoma, fusiform cell
37037810000006119		Small cell carcinoma - fusiform cell
37075010000006111		Chondroblastoma, malignant
37075110000006114		Malignant chondroblastoma
37075210000006118		Glycogen-rich carcinoma
37075310000006115		Glycogen-rich clear cell carcinoma
37087910000006113		Reserve cell carcinoma
37088010000006114		Round cell carcinoma
37088110000006112		SCC - Small cell carcinoma

3709581000006111		Embryonal carcinoma, infantile
3709631000006114		Infantile embryonal carcinoma
3711491000006112		Glioma, malignant, no ICD-O subtype
3713311000006111		Malignant lymphomatous polyposis
3713321000006115		Malignant lymphoma, lymphocytic, intermediate differentiation, diffuse [obs]
3713331000006117		Malignant lymphoma, centrocytic [obs]
3734751000006115		Malignant melanoma in giant pigmented naevus
3734761000006118		Malignant melanoma in giant pigmented nevus
3734771000006113		Malignant melanoma in congenital melanocytic nevus
3734781000006111		Malignant melanoma in congenital melanocytic naevus
3756761000006114		Lobular carcinoma, noninfiltrating
3756781000006116		Non-infiltrating lobular carcinoma
3756791000006118		LCIS - Lobular carcinoma in situ
3758741000006111		Malignant lymphoma, small noncleaved, Burkitt's, diffuse
3758751000006113		Malignant lymphoma, undifferentiated, Burkitt type [obs]
3759331000006111		Neurilemoma, malignant
3759341000006118		Neurilemmoma, malignant
3759361000006119		Malignant Schwannoma
3764771000006119		Local chemotherapy for malignant neoplasm

3766851 0000061 13		Leydig cell tumour, malignant
3766861 0000061 10		Leydig cell tumor, malignant
3766871 0000061 15		Interstitial cell tumor, malignant
3766881 0000061 17		Interstitial cell tumour, malignant
3766901 0000061 15		Malignant interstitial cell tumour
3766911 0000061 17		Malignant interstitial cell tumor
3766921 0000061 13		Malignant Leydig cell tumor
3767690 11	BB2 ..11	Papillary neoplasm
3771831 0000061 18		Comedocarcinoma, noninfiltrating
3771841 0000061 11		Ductal carcinoma in situ, comedo type
3771861 0000061 10		Non-infiltrating comedocarcinoma
3773431 0000061 16		Wide excision of malignant lesion of cervical oesophagus
3773441 0000061 14		Wide excision of malignant lesion of cervical esophagus
3773701 0000061 19		Meningioma, malignant
3787041 0000061 19		Pseudomucinous adenocarcinoma
3787051 0000061 17		Pseudomucinous cystadenocarcinoma
3789351 0000061 12		Secondary carcinoma
3789361 0000061 14		Secondary neoplasm
3790880 10	B14 1.11	Carcinoma of rectum
3790890 19	B14 1.12	Rectal carcinoma
3792230 11	B33 8.00	Squamous cell carcinoma of skin

3800590 13	B59 5.00	Malignant tumour of unknown origin
3800920 19	B4.. .11	Carcinoma of genitourinary organ
3800930 12	B3.. .12	Sarcoma of bone and connective tissue
3800940 18	B3.. .11	Carcinoma of bone, connective tissue, skin and breast
3800950 17	B0.. .11	Carcinoma of lip, oral cavity and pharynx
3801290 18	B13 4.11	Carcinoma of caecum
3801370 14	B14 2.11	Anal carcinoma
3801390 12	B16 121 1	Carcinoma common bile duct
3801450 16	B30 5.11	Malignant neoplasm of carpal bones
3801460 15	B30 5.12	Malignant neoplasm of metacarpal bones
3801800 17	B47 z.11	Seminoma of testis
3801930 10	B57 ..12	Secondary carcinoma of respiratory and/or digestive systems
3802060 18	B58 ..11	Secondary carcinoma of other specified sites
3802110 16	B5.. .11	Carcinoma of other and unspecified sites
3802420 14	B82 2.11	Carcinoma in situ of ear
3802491 0000061 15		Sertoli cell carcinoma
3806271 0000061 19		Thyroid imaging for metastatic carcinoma, whole body
3808731 0000061 11		Malignant edema
3808781 0000061 12		Malignant oedema
3810371 0000061 11		Malignant myelosclerosis [obs]
3812911 0000061 19		Water-clear cell adenocarcinoma
3812921 0000061 10		Water-clear cell carcinoma
3817071 0000061 14		International Federation of Gynaecology and Obstetrics endometrial cancer (FIGO EC) stage III
3817081 0000061 12		International Federation of Gynecology and Obstetrics endometrial cancer (FIGO EC) stage III

3817091000006110		International Federation of Gynecology and Obstetrics endometrial cancer stage III
3823121000006114		Malignant arteriolar nephrosclerosis
3824461000006113		Superficial spreading adenocarcinoma
3830481000006113		International Federation of Gynecology and Obstetrics cervical cancer (FIGO CC) stage IV
3830491000006111		International Federation of Gynaecology and Obstetrics cervical cancer (FIGO CC) stage IV
3830501000006115		International Federation of Gynecology and Obstetrics cervical cancer stage IV
3832091000006117		Solid carcinoma
3832101000006111		Solid carcinoma with mucin formation
3832111000006114		Solid adenocarcinoma with mucin formation
3838051000006113		Malignant tumour, small cell type
3838061000006110		Malignant tumor, small cell type
3838081000006117		Malignant tumor - small cell type
3843581000006117		Paget disease and infiltrating duct carcinoma of breast
3845541000006112		Infiltrating duct adenocarcinoma
3845551000006114		Duct cell carcinoma
3845561000006111		Ductal carcinoma
3845571000006116		Duct adenocarcinoma
3845591000006115		Invasive breast carcinoma of no special type
3848541000006117		Neck imaging for metastatic carcinoma of thyroid

3848811000006119		Jejunal neoplasm syndrome
3850371000006118		Malignant mixed tumour, chondrosarcomatous type
3850381000006115		Malignant mixed tumor, chondrosarcomatous type
3852661000006118		Malignant rhabdoid tumour
3852691000006114		Malignant rhabdoid tumor
3855061000006116		Malignant effusion
3855431000006116		Malignant teratoma, undifferentiated
3855441000006114		Malignant teratoma, anaplastic
3855461000006113		Malignant teratoma - anaplastic
3865781000006119		Malignant tumour, giant cell type
3865791000006116		Malignant tumor, giant cell type
3865801000006115		Malignant tumour - giant cell type
3865811000006117		Malignant tumor - giant cell type
3871510000006114	ZVu0900	[X]Follow-up examination after other treatment for malignant neoplasm
3871710000006116	ZVu0A00	[X]Follow-up examination after unspecified treatment for malignant neoplasm
3873491000006119		Malignant mixed Mullerian tumor
3873501000006110		Malignant mixed Mullerian tumour
3875711000006119		Cloacogenic carcinoma
3877151000006110		Haemangiopericytoma, malignant

3877161000006112		Hemangiopericytoma, malignant
3877171000006117		Malignant haemangiopericytoma
3877181000006119		Malignant hemangiopericytoma
38787100000117	7L1dy00	Other specified delivery of chemotherapy for neoplasm
38789100000118	7L1ey00	Other specified delivery of oral chemotherapy for neoplasm
3881141000006113		pTis: Carcinoma in situ
3882431000006115		Intraepidermal squamous cell carcinoma, Bowen's type
3882451000006110		SCC - Intraepidermal squamous cell carcinoma - Bowen's type
3884121000006115		Excision of malignant lesion of trunk
3892541000006110		Medullary carcinoma with lymphoid stroma
3897081000006111		Adenoid squamous cell carcinoma
3897091000006114		Pseudoglandular squamous cell carcinoma
3897101000006115		Squamous cell carcinoma, acantholytic
3897111000006117		Acantholytic squamous cell carcinoma
3898781000006114		Cancer
3898791000006112		Unclassified tumor, malignant
3898801000006113		Tumour, malignant
3898811000006111		Tumor, malignant
3898821000006115		Unclassified tumour, malignant

3898831 0000061 17		Malignant neoplasm
3898841 0000061 10		Malignant tumour morphology
3898851 0000061 12		Malignant tumor morphology
3898871 0000061 19		Malignant neoplasm, primary
3906651 0000061 18		Malignant smallpox
3908461 0000061 19		Intraductal carcinoma, noninfiltrating
3908471 0000061 14		Intraductal adenocarcinoma, noninfiltrating
3908481 0000061 12		Intraductal carcinoma
3908501 0000061 19		Ductal carcinoma in situ
3908531 0000061 10		Non-infiltrating intraductal carcinoma
3908541 0000061 17		Non-infiltrating intraductal adenocarcinoma
3908551 0000061 15		Intraductal carcinoma, noninfiltrating, no ICD-O subtype
3908561 0000061 18		Intraductal carcinoma, noninfiltrating, no International Classification of Diseases for Oncology subtype
3926021 0000061 10		Signet ring cell adenocarcinoma
3928741 0000061 16		Phyllodes tumour, malignant
3928751 0000061 19		Phyllodes tumor, malignant
3928761 0000061 17		Cystosarcoma phyllodes, malignant
3928771 0000061 12		Malignant phyllodes tumour
3928781 0000061 10		Malignant phyllodes tumor

3929541 0000061 17		International Federation of Gynecology and Obstetrics cervical cancer stage Ib occ
3929551 0000061 15		International Federation of Gynecology and Obstetrics cervical cancer (FIGO CC) stage Ib occ
3929561 0000061 18		International Federation of Gynaecology and Obstetrics cervical cancer stage Ib occ
3929571 0000061 13		International Federation of Gynaecology and Obstetrics cervical cancer (FIGO CC) stage Ib occ
3934021 0000061 16		Choroid plexus carcinoma
3934031 0000061 18		Choroid plexus papilloma, malignant
3934051 0000061 13		Ameloblastoma, malignant
3934061 0000061 10		Adamantinoma, malignant (except of Tibia and Long bones, M-92613)
3934071 0000061 15		Malignant adamantinoma
3935411 0000061 17		Teratoma with malignant transformation
3935421 0000061 13		Dermoid cyst with malignant transformation
3936561 0000061 12		Tumour cells, malignant
3936571 0000061 17		Tumor cells, malignant
3936591 0000061 16		Malignant tumor cells
3944811 0000061 17		Malignant tumour, fusiform cell type
3944821 0000061 13		Malignant tumor, fusiform cell type
3944831 0000061 11		Malignant tumor, spindle cell type
3944841 0000061 18		Malignant tumour, spindle cell type
3944851 0000061 16		Malignant tumour - fusiform cell type

3944861000006119		Malignant tumour - spindle cell type
3944871000006114		Malignant tumor - spindle cell type
3944881000006112		Malignant tumor - fusiform cell type
3956771000006112		Mesenchymoma, malignant
3956791000006113		Malignant mesenchymoma
3958781000006114		Lobular adenocarcinoma
3958791000006112		Infiltrating lobular carcinoma
396091000006118	Byu5800	[X]Mal neoplasm/connective+soft tissue of trunk,unspecified
396101000006112	ByuDB00	Malignant tumour of lymphoid haemopoietic and related tissue
396111000006110	Byu5500	Overlapping malignant neoplasm of peripheral nerves and autonomic nervous system
396121000006119	Byu3000	[X]Mal neoplasm/overlap lesion/bone+articular cartilage/limb
396131000006116	Byu5600	Malignant neoplasm of peripheral nerves and autonomic nervous system
3961571000006115		Verrucous squamous cell carcinoma
3961581000006117		Verrucous epidermoid carcinoma
3961591000006119		Warty carcinoma
3961601000006110		Condylomatous carcinoma
396161000006113	ByuA300	Overlapping malignant neoplasm of brain and other parts of the central nervous system
3961611000006113		SCC - Verrucous squamous cell carcinoma
396171000006118	Byu4000	[X]Malignant melanoma of other+unspecified parts of face

39619100000611 7	Byu 230 0	[X]Malignant neopl/overlapping les/resp+intrathoracic organs
39625100000611 5	Byu A.0 0	Malignant neoplasm of central nervous system
39628100000611 1	Byu C.00	[X]Malignant neoplasm of ill-defined, secondary and unspecified sites
39630100000611 0	Byu 0.00	[X]Malignant neoplasm of lip, oral cavity and pharynx
39638100000611 8	Byu 2.00	[X]Malignant neoplasm of respiratory and intrathoracic organs
39646100000611 7	Byu B00 0	Malignant neoplasm of multiple endocrine glands
39647100000611 2	Byu 330 0	[X]Malignant neoplasm/bone+articular cartilage, unspecified
39648100000611 0	Byu 310 0	Malignant neoplasm of bone and articular cartilage of limb
39649100000611 3	Byu A10 0	Malignant neoplasm of central nervous system
39650100000611 7	Byu 590 0	[X]Malignant neoplasm/connective + soft tissue,unspecified
39651100000611 9	Byu 240 0	[X]Malignant neoplasm/ill-defined sites within resp system
39652100000611 0	Byu A00 0	Malignant tumour of cranial nerve
39653100000611 3	Byu 710 0	Malignant tumor of female genital organ
39654100000611 5	Byu 800 0	[X]Malignant neoplasm/other specified male genital organs
39655100000611 8	Byu 320 0	Overlapping malignant neoplasm of bone and articular cartilage
39656100000611 6	Byu 210 0	[X]Malignant neoplasm/overlap lesion/heart,mediastinm+pleura
39657100000611 1	Byu C10 0	Overlapping malignant neoplasm of ill-defined site
39658100000611 4	Byu 720 0	Overlapping malignant neoplasm of female genital organs
39659100000611 2	Byu 810 0	Overlapping malignant neoplasm of male genital organs

3966010 0000611 6	Byu 540 0	Malignant neoplasm of peripheral nerves of trunk
3966110 0000611 8	Byu 220 0	[X]Malignant neoplasm/upper resp tract, part unspecified
3966210 0000611 4	Byu D.0 0	[X]Malignant neoplasms of lymphoid, haematopoietic and related tissue
3966310 0000611 2	Byu E.00	[X]Malignant neoplasms/independent (primary) multiple sites
3966410 0000611 9	Byu E00 0	[X]Malignant neoplasms/independent(primary)multiple sites
3966510 0000611 7	Byu 130 0	Malignant neoplasm of gastrointestinal tract
3967100 0006114	B62 xX0 0	Cutaneous/peripheral T-cell lymphoma
3967601 0000061 15		Papillary serous cystadenocarcinoma
3967611 0000061 17		Papillary serous adenocarcinoma
3967621 0000061 13		Micropapillary serous carcinoma
3974511 0000061 18		Serous adenocarcinoma
3974521 0000061 14		Serous carcinoma
3978180 15	142. .00	H/O: malignant neoplasm (*)
3978210 18	142. .11	H/O: cancer
3982910 0000611 0	Byu 501 1	[X]Mesothelioma of lung
3996810 0000611 5	Byu HB0 0	[X]Neoplasm of uncert/unkn behav female genit organs, unspec
3997210 0000611 0	Byu H90 0	[X]Neoplasm/uncertain+unknown behavior/other specified sites
3997610 0000611 6	Byu H60 0	[X]Neoplasm/uncertn+unknwn behavior/oth myelodysplastic synd
4000110 0000611 9	Byu DF1 1	[X]Non-Hodgkin's lymphoma NOS
4005611 0000061 18		Carcinoma in situ of accessory sinus

4005621 0000061 14		Carcinoma in situ of paranasal sinus
4005631 0000061 12		Carcinoma in situ of adenoid
4005641 0000061 19		Carcinoma in situ of adnexa of skin
4005651 0000061 17		Carcinoma in situ of adrenal cortex
4005671 0000061 10		Carcinoma in situ of adrenal medulla
4005681 0000061 13		Carcinoma in situ of alveolar ridge mucosa
4005691 0000061 11		Carcinoma in situ of alveolar mucosa
4005711 0000061 14		Ampullary carcinoma in situ
4005731 0000061 15		Cancer in situ of anal canal
4005741 0000061 13		Carcinoma in situ of anterior aspect of epiglottis
4005751 0000061 10		Carcinoma in situ of anterior two-thirds of tongue
4005761 0000061 12		Carcinoma in situ of mobile part of tongue
4005771 0000061 17		Carcinoma in situ of anterior wall of nasopharynx
4005781 0000061 19		Carcinoma in situ of anterior wall of urinary bladder
4005811 0000061 17		Carcinoma in situ of apex of urinary bladder
4005831 0000061 11		Carcinoma in situ of areola of female breast
4005841 0000061 18		Carcinoma in situ of areola of male breast
4005861 0000061 19		Carcinoma in situ of axillary tail of female breast
4005871 0000061 14		Carcinoma in situ of base of tongue

4005881000006112		Carcinoma in situ of posterior third of tongue
4005891000006110		Carcinoma in situ of fixed part of tongue
4005901000006114		Carcinoma in situ of biliary tract
4005911000006112		Cancer in situ of urinary bladder
4005931000006118		Carcinoma in situ of body of pancreas
4005941000006111		Cancer in situ of pancreas body
4005951000006113		Carcinoma in situ of body of penis
4005971000006115		Cancer in situ of body of stomach
4005981000006117		Carcinoma in situ of gastric body
4006001000006118		Carcinoma in situ of corpus uteri
4006011000006115		Carcinoma in situ of broad ligament
4006021000006111		Carcinoma in situ of bronchus of left lower lobe
4006031000006114		Carcinoma in situ of bronchus of left upper lobe
4006041000006116		Carcinoma in situ of bronchus of right lower lobe
4006051000006119		Carcinoma in situ of bronchus of right middle lobe
4006061000006117		Carcinoma in situ of bronchus of right upper lobe
4006071000006112		Carcinoma in situ of bronchus
4006091000006113		Carcinoma in situ of buccal mucosa
4006111000006116		Carcinoma in situ of cheek mucosa

4006121000006112		Carcinoma in situ of internal cheek
4006141000006117		Carcinoma in situ of cecum
4006161000006118		Carcinoma in situ of cardia
4006171000006113		Carcinoma in situ of gastric cardia
4006181000006111		Cancer in situ of cardia of stomach
4006191000006114		Carcinoma in situ of central portion of female breast
4006201000006112		Carcinoma in situ of cervical oesophagus
4006211000006110		Carcinoma in situ of cervical esophagus
4006221000006119		Carcinoma in situ of cervical part of oesophagus
4006231000006116		Carcinoma in situ of cervical part of esophagus
4006251000006111		Carcinoma in situ of cervix uteri
4006281000006115		Carcinoma in situ of choroid
4006291000006117		Carcinoma in situ of ciliary body
4006301000006116		Carcinoma in situ of clitoris
4006321000006114		Cancer in situ of colon
4006331000006112		Carcinoma in situ of commissure of lip
4006341000006119		Carcinoma in situ of labial commissure
4006361000006115		Carcinoma in situ of distal extra hepatic duct
4006371000006110		Cancer in situ of common bile duct

4006381000006113		Carcinoma in situ of conjunctiva
4006401000006113		Carcinoma in situ of cornea
4006411000006111		Carcinoma in situ of craniopharyngeal duct
4006431000006117		Cancer in situ of cystic duct
4006451000006112		Carcinoma in situ of dorsal surface of tongue
4006471000006119		Cancer in situ of duodenum
4006481000006116		Carcinoma in situ of ectopic female breast tissue
4006491000006118		Carcinoma in situ of ectopic male breast tissue
4006511000006112		Carcinoma in situ of endocrine gland
4006521000006116		Cancer in situ of endocrine gland
4006571000006115		Carcinoma in situ of esophagus
4006581000006117		Cancer in situ of esophagus
4006591000006119		Cancer in situ of oesophagus
4006611000006113		Carcinoma in situ of ethmoid sinus
4006641000006112		Carcinoma in situ of extrahepatic bile ducts
4006651000006114		Carcinoma in situ of extrahepatic bile duct
4006671000006116		Cancer in situ of eye
4006691000006115		Carcinoma in situ of false vocal cord
4006701000006115		Carcinoma in situ of ventricular bands of larynx

4006711 0000061 17		Carcinoma in situ of female breast
4006721 0000061 13		Carcinoma in situ of female genital organ
4006741 0000061 18		FOM - Carcinoma in situ of floor of mouth
4006751 0000061 16		Carcinoma in situ of prepuce
4006761 0000061 19		Carcinoma in situ of foreskin
4006791 0000061 10		Cancer in situ fundus of stomach
4006821 0000061 18		Cancer in situ of gastrointestinal tract
4006831 0000061 15		Carcinoma in situ of gingival mucosa
4006841 0000061 13		Carcinoma in situ of glans penis
4006871 0000061 17		Carcinoma in situ of greater curvature of stomach
4006881 0000061 19		Carcinoma in situ of greater curve of stomach
4006891 0000061 16		Carcinoma in situ of gum
4006901 0000061 17		Carcinoma in situ of gingiva
4006911 0000061 19		Cancer in situ of gum
4006921 0000061 10		Carcinoma in situ of hard palate
4006931 0000061 13		Carcinoma in situ of head of pancreas
4006951 0000061 18		Carcinoma in situ of hepatic flexure
4006961 0000061 16		Carcinoma in situ of hilus of lung
4006971 0000061 11		Carcinoma in situ of hypopharyngeal aspect of aryepiglottic fold

4006981 0000061 14		Carcinoma in situ of hypopharyngeal aspect of interarytenoid fold
4007001 0000061 16		Carcinoma in situ of laryngopharynx
4007011 0000061 18		Cancer in situ of hypopharynx
4007031 0000061 12		Cancer in situ of ileum
4007041 0000061 19		Carcinoma in situ of inner aspect of lip
4007051 0000061 17		Carcinoma in situ of labial mucosa
4007061 0000061 15		Carcinoma in situ of buccal aspect of lip
4007071 0000061 10		Carcinoma in situ of oral aspect of lip
4007081 0000061 13		Carcinoma in situ of mucosa of lip
4007101 0000061 17		Carcinoma in situ of intestine
4007121 0000061 10		Carcinoma in situ of islets of Langerhans
4007131 0000061 13		Carcinoma in situ of isthmus of uterus
4007151 0000061 18		Cancer in situ of jejunum
4007161 0000061 16		Carcinoma in situ of junctional region of epiglottis
4007171 0000061 11		Carcinoma in situ of junctional zone of tongue
4007181 0000061 14		Carcinoma in situ of kidney
4007191 0000061 12		Carcinoma in situ of labia majora
4007201 0000061 10		Carcinoma in situ of labia minora
4007211 0000061 13		Carcinoma in situ of lacrimal gland

4007221000006117		Carcinoma in situ of large intestine
4007231000006119		Carcinoma in situ of laryngeal aspect of aryepiglottic fold
4007241000006112		Carcinoma in situ of laryngeal aspect of interarytenoid fold
4007251000006114		Carcinoma in situ of laryngeal commissure
4007261000006111		Carcinoma in situ of laryngeal surface of epiglottis
4007281000006118		Cancer in situ of larynx
4007291000006115		Carcinoma in situ of lateral wall of nasopharynx
4007301000006119		Carcinoma in situ of lateral wall of oropharynx
4007311000006116		Carcinoma in situ of lateral wall of urinary bladder
4007321000006112		Carcinoma in situ of left lower lobe of lung
4007331000006110		Carcinoma in situ of left upper lobe of lung
4007341000006117		Carcinoma in situ of lingual tonsil
4007371000006113		Carcinoma in situ of lower gum
4007381000006111		Carcinoma in situ of lower gingiva
4007391000006114		Carcinoma in situ of lower inner quadrant of female breast
4007401000006111		Carcinoma in situ of lower outer quadrant of female breast
4007411000006114		Carcinoma in situ of lower third of oesophagus
4007421000006118		Carcinoma in situ of lower third of esophagus
4007441000006113		Carcinoma in situ of lower 1/3 esophagus

4007451 0000061 10		Carcinoma in situ of lung
4007461 0000061 12		Cancer in situ of lung
4007481 0000061 19		Carcinoma in situ of major salivary gland
4007491 0000061 16		Carcinoma in situ of male breast
4007501 0000061 12		Carcinoma in situ of male genital organ
4007531 0000061 16		Carcinoma in situ of maxillary antrum
4007551 0000061 11		Carcinoma in situ of Meckel diverticulum
4007561 0000061 13		Carcinoma in situ of middle ear
4007571 0000061 18		Carcinoma in situ of middle third of oesophagus
4007581 0000061 15		Carcinoma in situ of middle third of esophagus
4007601 0000061 13		Carcinoma in situ of middle 1/3 esophagus
4007611 0000061 11		Carcinoma in situ of minor salivary gland
4007621 0000061 15		Carcinoma in situ of mouth
4007641 0000061 10		Cancer in situ of mouth
4007651 0000061 12		Carcinoma in situ of multiple endocrine glands
4007661 0000061 14		Carcinoma in situ of myometrium
4007691 0000061 18		Carcinoma in situ of nasopharyngeal wall
4007701 0000061 18		PNS - carcinoma in situ of postnasal space
4007711 0000061 15		Carcinoma in situ of epipharynx

4007721 0000061 11		Carcinoma in situ of postnasal space
4007731 0000061 14		Carcinoma in situ of nipple of female breast
4007741 0000061 16		Carcinoma in situ of nipple of male breast
4007761 0000061 17		Carcinoma situ of oropharynx
4007771 0000061 12		Carcinoma in situ of mesopharynx
4007781 0000061 10		Cancer in situ of oropharynx
4007811 0000061 12		Carcinoma in situ of roof of mouth
4007821 0000061 16		Carcinoma in situ of tonsil
4007831 0000061 18		Carcinoma in situ of palatine tonsil
4007851 0000061 13		Cancer in situ of pancreas
4007861 0000061 10		Carcinoma in situ of pancreatic duct
4007871 0000061 15		Carcinoma in situ of parametrium
4007891 0000061 19		Carcinoma in situ of paraurethral glands
4007901 0000061 15		Carcinoma in situ of parietal pleura
4007911 0000061 17		Carcinoma in situ of parotid gland
4007931 0000061 11		Cancer in situ of penis
4007961 0000061 19		Cancer in situ of pharynx
4007971 0000061 14		Carcinoma in situ of pineal gland
4007991 0000061 10		Carcinoma in situ of placenta

4008011 0000061 19		Carcinoma in situ of postcricoid region
4008021 0000061 10		Carcinoma in situ of posterior hypopharyngeal wall
4008031 0000061 13		Carcinoma in situ of posterior wall of nasopharynx
4008041 0000061 15		Carcinoma in situ of posterior wall of oropharynx
4008051 0000061 18		Carcinoma in situ of posterior wall of urinary bladder
4008071 0000061 11		Cancer in situ of prostate
4008091 0000061 12		Carcinoma in situ of pylorus
4008111 0000061 15		Cancer in situ of pylorus
4008121 0000061 11		Carcinoma in situ of pyriform sinus
4008151 0000061 19		Cancer in situ of rectum
4008161 0000061 17		Carcinoma in situ of renal pelvis
4008171 0000061 12		Carcinoma in situ of respiratory tract
4008181 0000061 10		Carcinoma in situ of retina
4008191 0000061 13		Carcinoma in situ of retromolar area
4008201 0000061 11		Carcinoma in situ of right lower lobe of lung
4008211 0000061 14		Carcinoma in situ of right middle lobe of lung
4008221 0000061 18		Carcinoma in situ of right upper lobe of lung
4008231 0000061 15		Carcinoma in situ of round ligament of uterus
4008241 0000061 13		Carcinoma in situ of sclera

4008261 0000061 12		Carcinoma in situ of sebaceous gland
4008281 0000061 19		Carcinoma in situ of skin of abdomen
4008291 0000061 16		Carcinoma in situ of skin of ankle
4008351 0000061 16		Carcinoma in situ of skin of chest
4008361 0000061 19		Carcinoma in situ of skin of chin
4008371 0000061 14		Carcinoma in situ of skin of ear
4008381 0000061 12		Cancer in situ of skin of ear
4008391 0000061 10		Carcinoma in situ of skin of elbow
4008411 0000061 10		Carcinoma in situ of skin of eyelid
4008421 0000061 19		Cancer in situ of skin of eyelid
4008431 0000061 16		Carcinoma in situ of skin of face
4008441 0000061 14		Cancer in situ of skin of face
4008451 0000061 11		Carcinoma in situ of skin of finger
4008471 0000061 18		Carcinoma in situ of skin of forearm
4008481 0000061 15		Carcinoma in situ of skin of forehead
4008551 0000061 12		Cancer in situ skin of lip
4008561 0000061 14		Carcinoma in situ of skin of lower limb
4008581 0000061 16		Cancer in situ skin of neck
4008611 0000061 12		Carcinoma in situ of skin of popliteal area

4008621 0000061 16		Carcinoma in situ of skin of scalp
4008631 0000061 18		Cancer in situ of skin of scalp
4008651 0000061 13		Carcinoma in situ of skin of temporal region
4008671 0000061 15		Carcinoma in situ of skin of toe
4008681 0000061 17		Carcinoma in situ of skin of trunk
4008691 0000061 19		Cancer in situ skin of trunk
4008701 0000061 19		Carcinoma in situ of skin of umbilicus
4008711 0000061 16		Carcinoma in situ of skin of upper limb
4008721 0000061 12		Cancer in situ of skin of upper limb
4008731 0000061 10		Carcinoma in situ of skin of wrist
4008751 0000061 15		Cancer in situ of skin
4008761 0000061 18		Carcinoma in situ of small intestine
4008771 0000061 13		Cancer in situ of small intestine
4008781 0000061 11		Carcinoma in situ of soft palate
4008811 0000061 13		Carcinoma in situ of sphenoid sinus
4008831 0000061 19		Cancer in situ of spleen
4008851 0000061 14		Carcinoma in situ of subglottis
4008861 0000061 11		Carcinoma in situ of sublingual gland
4008871 0000061 16		Carcinoma in situ of submaxillary gland

4008881 0000061 18		Carcinoma in situ of superior wall of nasopharynx
4008891 0000061 15		Carcinoma in situ of supraglottis
4008901 0000061 16		Carcinoma in situ of sweat gland
4008911 0000061 18		Carcinoma in situ of tail of pancreas
4008931 0000061 12		Carcinoma in situ of thyroglossal duct
4008941 0000061 19		Carcinoma in situ of thyroid gland
4008961 0000061 15		Cancer in situ of thyroid
4008971 0000061 10		Carcinoma in situ of border of tongue
4008981 0000061 13		Carcinoma in situ of tip AND/OR lateral border of tongue
4009001 0000061 11		Cancer in situ of tongue
4009011 0000061 14		Carcinoma in situ of tonsillar fossa
4009021 0000061 18		Carcinoma in situ of tonsillar pillar
4009061 0000061 12		Carcinoma in situ of trigone of urinary bladder
4009071 0000061 17		Carcinoma in situ of undescended testis
4009081 0000061 19		Carcinoma in situ of upper gum
4009091 0000061 16		Carcinoma in situ of upper gingiva
4009101 0000061 10		Carcinoma in situ of upper inner quadrant of female breast
4009111 0000061 13		Carcinoma in situ of upper outer quadrant of female breast
4009121 0000061 17		Carcinoma in situ of upper respiratory tract

4009131 0000061 19		Carcinoma in situ of upper third of oesophagus
4009141 0000061 12		Carcinoma in situ of upper third esophagus
4009151 0000061 14		Carcinoma in situ of upper third oesophagus
4009161 0000061 11		Carcinoma in situ of upper third of esophagus
4009181 0000061 18		Carcinoma in situ of upper 1/3 esophagus
4009191 0000061 15		Carcinoma in situ of urachus
4009201 0000061 17		Carcinoma in situ of ureter
4009211 0000061 19		Carcinoma in situ of ureteric orifice of urinary bladder
4009221 0000061 10		Carcinoma in situ of urethra
4009231 0000061 13		Carcinoma in situ of urinary bladder neck
4009241 0000061 15		Carcinoma in situ of urinary system
4009251 0000061 18		Cancer in situ of urinary system
4009261 0000061 16		Carcinoma in situ of uterine adnexa
4009271 0000061 11		Carcinoma in situ of uterus
4009281 0000061 14		Cancer in situ of uterus
4009291 0000061 12		Carcinoma in situ of uveal tract
4009301 0000061 13		Carcinoma in situ of uvula
4009351 0000061 12		Cancer in situ of vagina
4009361 0000061 14		Carcinoma in situ of vallecula

4009371 0000061 19		Carcinoma in situ of vas deferens
4009381 0000061 16		Carcinoma in situ of ventral surface of tongue
4009391 0000061 18		Carcinoma in situ of vermilion border of lip
4009401 0000061 16		Carcinoma in situ of external lip
4009411 0000061 18		Carcinoma in situ of lipstick area of lip
4009421 0000061 14		Carcinoma in situ of vermilion border of lower lip
4009431 0000061 12		Carcinoma in situ of external lower lip
4009441 0000061 19		Carcinoma in situ of lipstick area of lower lip
4009451 0000061 17		Carcinoma in situ of vermilion border of upper lip
4009461 0000061 15		Carcinoma in situ of lipstick area of upper lip
4009471 0000061 10		Carcinoma in situ of external upper lip
4009481 0000061 13		Carcinoma in situ of vestibule of mouth
4009491 0000061 11		Carcinoma in situ of vestibule of nose
4009501 0000061 15		Carcinoma in situ of visceral pleura
4009511 0000061 17		Carcinoma in situ of vocal cord
4009521 0000061 13		Carcinoma in situ of vocal fold
4009541 0000061 18		Cancer in situ of vulva
4009551 0000061 16		Carcinoma in situ of Waldeyer's ring
4009561 0000061 19		Carcinoma in situ of Waldeyer ring

4016251000006114		Malignant histiocytosis of lymph nodes of axilla AND/OR upper limb
4016261000006111		Malignant histiocytosis of lymph nodes of head, face AND/OR neck
4016271000006116		Malignant histiocytosis of lymph nodes of inguinal region AND/OR lower limb
4016301000006119		Malignant histiocytosis of extranodal AND/OR solid organ site
4016311000006116		Malignant lymphoma of intra-abdominal lymph nodes
4016321000006112		Malignant lymphoma of intrapelvic lymph nodes
4016331000006110		Malignant lymphoma of intrathoracic lymph nodes
4016341000006117		Malignant lymphoma of lymph nodes of axilla AND/OR upper limb
4016351000006115		Malignant lymphoma of lymph nodes of head, face AND/OR neck
4016361000006118		Malignant lymphoma of lymph nodes of inguinal region AND/OR lower limb
4016371000006113		Malignant lymphoma of lymph nodes of multiple sites
4016381000006111		Malignant lymphoma of spleen
4016391000006114		Malignant lymphoma of extranodal AND/OR solid organ site
4016401000006111		Malignant mast cell tumour of intra-abdominal lymph nodes
4016411000006114		Malignant mast cell tumor of intra-abdominal lymph nodes
4016421000006118		Malignant mast cell tumour of intrapelvic lymph nodes
4016431000006115		Malignant mast cell tumor of intrapelvic lymph nodes
4016441000006113		Malignant mast cell tumour of intrathoracic lymph nodes
4016451000006110		Malignant mast cell tumor of intrathoracic lymph nodes

4016461000006112		Malignant mast cell tumour of lymph nodes of axilla AND/OR upper limb
4016471000006117		Malignant mast cell tumor of lymph nodes of axilla AND/OR upper limb
4016481000006119		Malignant mast cell tumour of lymph nodes of head, face AND/OR neck
4016491000006116		Malignant mast cell tumor of lymph nodes of head, face AND/OR neck
4016501000006112		Malignant mast cell tumour of lymph nodes of inguinal region AND/OR lower limb
4016511000006110		Malignant mast cell tumor of lymph nodes of inguinal region AND/OR lower limb
4016521000006119		Malignant mast cell tumour of lymph nodes of multiple sites
4016531000006116		Malignant mast cell tumor of lymph nodes of multiple sites
4016541000006114		Malignant mast cell tumour of spleen
4016551000006111		Malignant mast cell tumor of spleen
4016561000006113		Malignant mast cell tumour of extranodal AND/OR solid organ site
4016571000006118		Malignant mast cell tumor of extranodal AND/OR solid organ site
4016591000006117		Malignant melanoma of skin of abdomen
4016601000006113		Malignant melanoma of skin of ankle
4016611000006111		Malignant melanoma of skin of axilla
4016621000006115		Malignant melanoma of skin of back
4016631000006117		Malignant melanoma of skin of breast
4016641000006110		Malignant melanoma of skin of buttock
4016651000006112		Malignant melanoma of skin of cheek

4016671000006119		Malignant melanoma of skin of chest
4016681000006116		Malignant melanoma of skin of chin
4016691000006118		Malignant melanoma of skin of ear
4016701000006118		Malignant melanoma of skin of elbow
4016711000006115		Malignant melanoma of skin of external auditory canal
4016721000006111		Malignant melanoma of skin of eyebrow
4016741000006116		Malignant melanoma of skin of eyelid
4016761000006117		Malignant melanoma of skin of finger
4016771000006112		Malignant melanoma of skin of foot
4016781000006110		Malignant melanoma of skin of forearm
4016791000006113		Malignant melanoma of skin of forehead
4016801000006114		Malignant melanoma of skin of groin
4024231000006119		Malignant melanoma of skin of hand
4024241000006112		Malignant melanoma of skin of hip
4024251000006114		Malignant melanoma of skin of knee
4024261000006111		Malignant melanoma of skin of lip
4024271000006116		Malignant melanoma of skin of lower limb
4024281000006118		Malignant melanoma of skin of neck
4024291000006115		Malignant melanoma of skin of nose

4024311000006116		Malignant melanoma of skin of perineum
4024321000006112		Malignant melanoma of skin of popliteal area
4024331000006110		Malignant melanoma of skin of scalp
4024341000006117		Malignant melanoma of skin of shoulder
4024351000006115		Malignant melanoma of skin of temporal region
4024361000006118		Malignant melanoma of skin of thigh
4024371000006113		Malignant melanoma of skin of toe
4024391000006114		Malignant melanoma of skin of umbilicus
4024401000006111		Malignant melanoma of skin of upper limb
4024411000006114		Malignant melanoma of skin of wrist
4024431000006115		MM - Malignant melanoma of skin
4024441000006113		Cutaneous malignant melanoma
4024461000006112		Primary malignant neoplasm of abducens nerve
4024471000006117		Primary malignant neoplasm of accessory nerve
4024481000006119		Primary malignant neoplasm of accessory sinus
4024491000006116		Primary malignant neoplasm of acoustic nerve
4024501000006112		Primary malignant neoplasm of acromion
4024521000006119		Primary malignant neoplasm of adenoid
4024531000006116		Primary malignant neoplasm of adnexa of skin

4024541 0000061 14		Primary malignant neoplasm of skin with adnexal differentiation
4024551 0000061 11		Primary malignant neoplasm of adrenal gland
4024561 0000061 13		Primary malignant neoplasm of alveolar ridge mucosa
4024571 0000061 18		Primary malignant neoplasm of anal canal
4024581 0000061 15		Primary malignant neoplasm of anterior aspect of epiglottis
4024591 0000061 17		Primary malignant neoplasm of anterior mediastinum
4024601 0000061 13		Primary malignant neoplasm of anterior portion of floor of mouth
4024611 0000061 11		Primary malignant neoplasm of anterior wall of nasopharynx
4024621 0000061 15		Primary malignant neoplasm of anus
4024631 0000061 17		Primary malignant neoplasm of aortic body
4024651 0000061 12		Primary malignant neoplasm of appendix
4024661 0000061 14		Appendiceal cancer
4024671 0000061 19		Primary malignant neoplasm of areola of female breast
4024691 0000061 18		Primary malignant neoplasm of areola of male breast
4024711 0000061 15		Primary malignant neoplasm of upper arm
4024721 0000061 11		Primary malignant neoplasm of ascending colon
4024731 0000061 14		Primary malignant neoplasm of axilla
4024741 0000061 16		Primary malignant neoplasm of back
4024751 0000061 19		Primary malignant neoplasm of base of tongue

4024761 0000061 17		Primary malignant neoplasm of bladder
4024771 0000061 12		Primary malignant neoplasm of blood vessel of abdomen
4024781 0000061 10		Primary malignant neoplasm of blood vessel of axilla
4024791 0000061 13		Primary malignant neoplasm of blood vessel of buttock
4024801 0000061 14		Primary malignant neoplasm of blood vessel of face
4024811 0000061 12		Primary malignant neoplasm of blood vessel of finger
4024821 0000061 16		Primary malignant neoplasm of blood vessel of foot
4024831 0000061 18		Primary malignant neoplasm of blood vessel of forearm
4024841 0000061 11		Primary malignant neoplasm of blood vessel of hand
4024851 0000061 13		Primary malignant neoplasm of blood vessel of head
4024861 0000061 10		Primary malignant neoplasm of blood vessel of hip
4024871 0000061 15		Primary malignant neoplasm of blood vessel of inguinal region
4024881 0000061 17		Primary malignant neoplasm of blood vessel of lower limb
4024891 0000061 19		Primary malignant neoplasm of blood vessel of neck
4024901 0000061 15		Primary malignant neoplasm of blood vessel of pelvis
4024911 0000061 17		Primary malignant neoplasm of blood vessel of perineum
4024921 0000061 13		Primary malignant neoplasm of blood vessel of popliteal space
4024931 0000061 11		Primary malignant neoplasm of blood vessel of shoulder
4024941 0000061 18		Primary malignant neoplasm of blood vessel of thigh

4024951 0000061 16		Primary malignant neoplasm of blood vessel of thorax
4024961 0000061 19		Primary malignant neoplasm of blood vessel of toe
4024971 0000061 14		Primary malignant neoplasm of blood vessel of trunk
4024981 0000061 12		Primary malignant neoplasm of blood vessel of upper limb
4024991 0000061 10		Primary malignant neoplasm of blood vessel
4025001 0000061 15		Primary malignant neoplasm of body of pancreas
4025011 0000061 17		Primary malignant neoplasm of body of penis
4025021 0000061 13		Primary malignant neoplasm of body of stomach
4025031 0000061 11		Primary malignant neoplasm of bone marrow
4025041 0000061 18		Primary malignant neoplasm of bone of face
4025051 0000061 16		Primary malignant neoplasm of bone of lower limb
4025061 0000061 19		Primary malignant neoplasm of bone of skull
4025071 0000061 14		Primary malignant neoplasm of bone of upper limb
4025081 0000061 12		Primary malignant neoplasm of bone
4025091 0000061 10		Primary malignant neoplasm of brain stem
4025101 0000061 16		Primary malignant neoplasm of brain
4025111 0000061 18		Primary malignant neoplasm of broad ligament
4025131 0000061 12		Primary malignant neoplasm of bronchus of left lower lobe
4025141 0000061 19		Primary malignant neoplasm of bronchus of left upper lobe

4025151000006117		Primary malignant neoplasm of bronchus of right lower lobe
4025161000006115		Primary malignant neoplasm of bronchus of right middle lobe
4025171000006110		Primary malignant neoplasm of bronchus of right upper lobe
4025181000006113		Primary malignant neoplasm of bronchus
4025191000006111		Primary bronchial cancer
4025201000006114		Primary malignant neoplasm of calcaneus
4025211000006112		Primary malignant neoplasm of cardia of stomach
4025221000006116		Primary malignant neoplasm of carina
4025231000006118		Primary malignant neoplasm of carotid body
4025241000006111		Primary malignant neoplasm of carpal bone
4025251000006113		Primary malignant neoplasm of cartilage of nose
4025261000006110		Primary malignant neoplasm of cauda equina
4025271000006115		Primary malignant neoplasm of central nervous system
4025281000006117		Primary malignant neoplasm of central portion of female breast
4025291000006119		Primary malignant neoplasm of cerebellum
4025311000006115		Primary malignant neoplasm of cerebral meninges
4025321000006111		Primary malignant neoplasm of cerebral ventricle
4025331000006114		Primary malignant neoplasm of cerebrum
4025341000006116		Primary malignant neoplasm of cervical vertebral column

4025351 0000061 19		Primary malignant neoplasm of cheek
4025361 0000061 17		Primary malignant neoplasm of chest wall
4025371 0000061 12		Primary malignant neoplasm of choroid (primary)
4025381 0000061 10		Primary malignant neoplasm of choroid, primary
4025391 0000061 13		Primary malignant neoplasm of ciliary body (primary)
4025401 0000061 10		Primary malignant neoplasm of ciliary body, primary
4025411 0000061 13		Primary malignant neoplasm of clavicle
4025431 0000061 19		Primary malignant neoplasm of coccygeal body
4025451 0000061 14		Primary malignant neoplasm of coccyx
4025461 0000061 11		Primary malignant neoplasm of colon
4025471 0000061 16		Primary malignant neoplasm of common bile duct
4025491 0000061 15		Primary malignant neoplasm of conjunctiva (primary)
4025501 0000061 11		Primary malignant neoplasm of conjunctiva, primary
4025511 0000061 14		Primary malignant neoplasm of cornea (primary)
4025521 0000061 18		Primary malignant neoplasm of cornea, primary
4025531 0000061 15		Primary malignant neoplasm of cranial nerve
4025541 0000061 13		Primary malignant neoplasm of craniopharyngeal duct
4025551 0000061 10		Primary malignant neoplasm of cuboid
4025571 0000061 17		Primary malignant neoplasm of cystic duct

4025591000006116		Primary malignant neoplasm of descending colon
4025601000006112		Primary malignant neoplasm of diaphragm
4025621000006119		Primary malignant neoplasm of dorsal surface of tongue
4025631000006116		Primary malignant neoplasm of duodenum
4025641000006114		Primary malignant neoplasm of ectopic female breast tissue
4025651000006111		Primary malignant neoplasm of ectopic male breast tissue
4025661000006113		Primary malignant neoplasm of endocardium
4025671000006118		Primary malignant neoplasm of endocervix
4025681000006115		Cancer of endocervix
4025691000006117		Primary malignant neoplasm of endometrium
4025701000006117		Cancer of endometrium
4025711000006119		Primary malignant neoplasm of epicardium
4025731000006113		Primary malignant neoplasm of epididymis
4025741000006115		Primary malignant neoplasm of epiglottis
4025751000006118		Primary malignant neoplasm of ethmoid bone
4025771000006111		Primary malignant neoplasm of ethmoidal sinus
4025781000006114		Primary malignant neoplasm of eustachian tube
4025791000006112		Primary malignant neoplasm of exocervix
4025801000006113		Primary malignant neoplasm of face

4025811 0000061 11		Primary malignant neoplasm of facial nerve
4025821 0000061 15		Primary malignant neoplasm of female breast
4025831 0000061 17		Primary malignant neoplasm of female genital organ
4025841 0000061 10		Primary malignant neoplasm of femur
4025861 0000061 14		Primary malignant neoplasm of fibula
4025881 0000061 16		Primary malignant neoplasm of first cuneiform bone of foot
4025891 0000061 18		Primary malignant neoplasm of flank
4025901 0000061 19		Primary malignant neoplasm of floor of mouth
4025911 0000061 16		Primary malignant neoplasm of foot
4025921 0000061 12		Primary malignant neoplasm of forearm
4025931 0000061 10		Primary malignant neoplasm of frontal bone
4025951 0000061 15		Primary malignant neoplasm of frontal lobe
4025961 0000061 18		Primary malignant neoplasm of frontal sinus
4025971 0000061 13		Primary malignant neoplasm of fundus of stomach
4025981 0000061 11		Primary malignant neoplasm of gingival mucosa
4025991 0000061 14		Primary malignant neoplasm of glomus jugulare
4026011 0000061 13		Primary malignant neoplasm of glossopharyngeal nerve
4026021 0000061 17		Primary malignant neoplasm of glottis
4026031 0000061 19		Primary malignant neoplasm of great vessels

4026051 0000061 14		Primary malignant neoplasm of greater curvature of stomach
4026061 0000061 11		Primary malignant neoplasm of hamate bone
4026071 0000061 16		Primary malignant neoplasm of hand
4026081 0000061 18		Primary malignant neoplasm of head
4026091 0000061 15		Primary malignant neoplasm of heart
4026101 0000061 14		Primary malignant neoplasm of hepatic flexure of colon
4026111 0000061 12		Primary malignant neoplasm of hilus of lung
4026131 0000061 18		Primary malignant neoplasm of hypoglossal nerve
4026141 0000061 11		Primary malignant neoplasm of hypopharyngeal aspect of aryepiglottic fold
4026151 0000061 13		Primary malignant neoplasm of hypopharyngeal aspect of interarytenoid fold
4026161 0000061 10		Primary malignant neoplasm of hypopharynx
4026171 0000061 15		Primary malignant neoplasm of ileum
4026181 0000061 17		Primary malignant neoplasm of ilium
4026201 0000061 16		Primary malignant neoplasm of inguinal region
4026211 0000061 18		Primary malignant neoplasm of inner aspect of lip
4026221 0000061 14		Primary malignant neoplasm of inner aspect of lower lip
4026231 0000061 12		Primary malignant neoplasm of inner aspect of upper lip
4026241 0000061 19		Primary malignant neoplasm of intra-abdominal organs
4026251 0000061 17		Primary malignant neoplasm of intrathoracic organs

4026261 0000061 15		Primary malignant neoplasm of ischium
4026281 0000061 13		Primary malignant neoplasm of islets of Langerhans
4026291 0000061 11		Primary malignant neoplasm of isthmus of uterus
4026301 0000061 12		Primary malignant neoplasm of jaw
4026311 0000061 10		Primary malignant neoplasm of jejunum
4026321 0000061 19		Primary malignant neoplasm of junctional zone of tongue
4026331 0000061 16		Primary malignant neoplasm of kidney
4026341 0000061 14		Primary malignant neoplasm of labia majora
4026351 0000061 11		Primary malignant neoplasm of labia minora
4026361 0000061 13		Primary malignant neoplasm of large intestine
4026371 0000061 18		Primary malignant neoplasm of laryngeal commissure
4026381 0000061 15		Primary malignant neoplasm of laryngeal surface of epiglottis
4026391 0000061 17		Primary malignant neoplasm of lateral portion of floor of mouth
4026401 0000061 15		Primary malignant neoplasm of lateral wall of nasopharynx
4026411 0000061 17		Primary malignant neoplasm of lateral wall of oropharynx
4026421 0000061 13		Primary malignant neoplasm of lower lobe of left lung
4026431 0000061 11		Primary malignant neoplasm of left lower lobe of lung
4026441 0000061 18		Primary malignant neoplasm of left upper lobe of lung
4026451 0000061 16		Primary malignant neoplasm of upper lobe of left lung

4026461000006119		Primary malignant neoplasm of lesser curvature of stomach
4026471000006114		Primary malignant neoplasm of lingual tonsil
4026491000006110		CA - Liver cancer
4026501000006119		Liver cancer
4026511000006116		Malignant tumor of liver
4026521000006112		Malignant tumour of liver
4026531000006110		Primary malignant neoplasm of long bone of lower limb
4026541000006117		Primary malignant neoplasm of long bone of upper limb
4026551000006115		Primary malignant neoplasm of lower inner quadrant of female breast
4026561000006118		Primary malignant neoplasm of lower limb
4026571000006113		Primary malignant neoplasm of lower outer quadrant of female breast
4026581000006111		Primary malignant neoplasm of lumbar vertebral column
4026591000006114		Primary malignant neoplasm of lunate bone
4026621000006111		Primary malignant neoplasm of main bronchus
4026631000006114		Primary malignant neoplasm of major salivary gland
4026641000006116		Primary malignant neoplasm of male breast
4026651000006119		Primary malignant neoplasm of male genital organ
4026661000006117		Primary malignant neoplasm of mandible
4026671000006112		Primary malignant neoplasm of mastoid air cells

4026681 0000061 10		Primary malignant neoplasm of maxilla
4026701 0000061 13		Primary malignant neoplasm of maxillary sinus
4026711 0000061 11		Primary malignant neoplasm of Meckel's diverticulum
4026721 0000061 15		Primary malignant neoplasm of Meckel diverticulum
4026731 0000061 17		Primary malignant neoplasm of mediastinum
4026741 0000061 10		Primary malignant neoplasm of metacarpal bone
4026751 0000061 12		Primary malignant neoplasm of metatarsal bone
4026761 0000061 14		Primary malignant neoplasm of middle ear
4026771 0000061 19		Primary malignant neoplasm of multiple endocrine glands
4026781 0000061 16		Primary malignant neoplasm of muscle of abdomen
4026791 0000061 18		Primary malignant neoplasm of muscle of buttock
4026801 0000061 17		Primary malignant neoplasm of muscle of face
4026811 0000061 19		Primary malignant neoplasm of muscle of head
4026821 0000061 10		Primary malignant neoplasm of muscle of hip
4026831 0000061 13		Primary malignant neoplasm of muscle of inguinal region
4026841 0000061 15		Primary malignant neoplasm of muscle of lower limb
4026851 0000061 18		Primary malignant neoplasm of muscle of neck
4026861 0000061 16		Primary malignant neoplasm of muscle of pelvis
4026871 0000061 11		Primary malignant neoplasm of muscle of perineum

4026881000006114		Primary malignant neoplasm of muscle of shoulder
4026891000006112		Primary malignant neoplasm of muscle of thorax
4026901000006111		Primary malignant neoplasm of muscle of trunk
4026911000006114		Primary malignant neoplasm of muscle of upper limb
4026921000006118		Primary malignant neoplasm of muscle
4026931000006115		Primary malignant neoplasm of myocardium
4026941000006113		Primary malignant neoplasm of myometrium
4026951000006110		Primary malignant neoplasm of nasal bone
4026971000006117		Primary malignant neoplasm of nasal cavity
4026981000006119		Primary malignant neoplasm of nasal concha
4026991000006116		Primary malignant neoplasm of navicular bone of foot
4027001000006112		Primary malignant neoplasm of neck
4027011000006110		Primary malignant neoplasm of nervous system
4027021000006119		Primary malignant neoplasm of nipple of female breast
4027041000006114		Primary malignant neoplasm of nipple of male breast
4027061000006113		Primary malignant neoplasm of nose
4027071000006118		Primary malignant neoplasm of occipital bone
4027091000006117		Primary malignant neoplasm of occipital lobe
4027101000006111		Primary malignant neoplasm of oculomotor nerve

4027111000006114		Primary malignant neoplasm of olfactory nerve
4027121000006118		Primary malignant neoplasm of optic nerve
4027131000006115		Primary malignant neoplasm of orbit
4027141000006113		Primary malignant neoplasm of oropharynx
4027151000006110		Primary malignant neoplasm of ovary
4027161000006112		Primary malignant neoplasm of palatine bone
4027171000006117		Primary malignant neoplasm of pancreatic duct
4027181000006119		Primary malignant neoplasm of para-aortic body
4027191000006116		Primary malignant neoplasm of paraganglion
4027201000006118		Primary malignant neoplasm of parametrium
4027211000006115		Primary malignant neoplasm of parathyroid gland
4027221000006111		Primary malignant neoplasm of paraurethral glands
4027231000006114		Primary malignant neoplasm of parietal bone
4027251000006119		Primary malignant neoplasm of parietal lobe
4027261000006117		Primary malignant neoplasm of parietal peritoneum
4027281000006110		Primary malignant neoplasm of parietal pleura
4027291000006113		Primary malignant neoplasm of patella
4027311000006112		Primary malignant neoplasm of pelvic bone
4027321000006116		Primary malignant neoplasm of pelvic peritoneum

4027331 0000061 18		Primary malignant neoplasm of pelvis
4027341 0000061 11		Primary malignant neoplasm of periadrenal tissue
4027361 0000061 10		Primary malignant neoplasm of perianal skin
4027381 0000061 17		Primary malignant neoplasm of pericardium
4027401 0000061 17		Primary malignant neoplasm of perirenal tissue
4027411 0000061 19		Primary malignant neoplasm of phalanx of foot
4027421 0000061 10		Primary malignant neoplasm of phalanx of hand
4027431 0000061 13		Primary malignant neoplasm of pharynx
4027441 0000061 15		Primary malignant neoplasm of pineal gland
4027451 0000061 18		Primary malignant neoplasm of pisiform bone of hand
4027461 0000061 16		Primary malignant neoplasm of pituitary gland
4027471 0000061 11		Primary malignant neoplasm of pleura
4027481 0000061 14		Primary malignant neoplasm of postcricoid region
4027491 0000061 12		Primary malignant neoplasm of posterior hypopharyngeal wall
4027501 0000061 16		Primary malignant neoplasm of posterior mediastinum
4027511 0000061 18		Primary malignant neoplasm of posterior wall of nasopharynx
4027521 0000061 14		Primary malignant neoplasm of posterior wall of oropharynx
4027531 0000061 12		Primary malignant neoplasm of presacral region
4027551 0000061 17		Primary malignant neoplasm of prostate

4027561 0000061 15		Primary malignant neoplasm of pubis
4027581 0000061 13		Primary malignant neoplasm of pyloric antrum
4027591 0000061 11		Primary malignant neoplasm of pylorus
4027601 0000061 15		Primary malignant neoplasm of pyriform sinus
4027611 0000061 17		Primary malignant neoplasm of radius
4027631 0000061 11		Primary malignant neoplasm of rectosigmoid junction
4027641 0000061 18		Primary malignant neoplasm of rectouterine pouch
4027651 0000061 16		Primary malignant neoplasm of rectovaginal septum
4027661 0000061 19		Primary malignant neoplasm of rectovesical septum
4027671 0000061 14		Primary malignant neoplasm of rectum
4027681 0000061 12		Primary malignant neoplasm of renal pelvis
4027691 0000061 10		Primary malignant neoplasm of respiratory tract
4027701 0000061 10		Primary malignant neoplasm of retina (primary)
4027711 0000061 13		Primary malignant neoplasm of retina, primary
4027721 0000061 17		Primary malignant neoplasm of retromolar area
4027731 0000061 19		Primary malignant neoplasm of rib
4027751 0000061 14		Primary malignant neoplasm of right lower lobe of lung
4027761 0000061 11		Primary malignant neoplasm of right middle lobe of lung
4027771 0000061 16		Primary malignant neoplasm of upper lobe of right lung

4027781 0000061 18		Primary malignant neoplasm of right upper lobe of lung
4027791 0000061 15		Primary malignant neoplasm of round ligament of uterus
4027801 0000061 19		Primary malignant neoplasm of sacrococcygeal region
4027821 0000061 12		Primary malignant neoplasm of sacrum
4027831 0000061 10		Primary malignant neoplasm of scapula
4027851 0000061 15		Primary malignant neoplasm of sclera (primary)
4027861 0000061 18		Primary malignant neoplasm of sclera, primary
4027871 0000061 13		Primary malignant neoplasm of sebaceous gland
4027891 0000061 14		Primary malignant neoplasm of second cuneiform bone of foot
4027901 0000061 13		Primary malignant neoplasm of septum of nose
4027911 0000061 11		Primary malignant neoplasm of short bone of lower limb
4027921 0000061 15		Primary malignant neoplasm of short bone of upper limb
4027931 0000061 17		Primary malignant neoplasm of shoulder
4027941 0000061 10		Primary malignant neoplasm of sigmoid colon
4027951 0000061 12		Primary malignant neoplasm of skin of abdomen
4027961 0000061 14		Primary malignant neoplasm of skin of ankle
4027981 0000061 16		Primary malignant neoplasm of skin of axilla
4027991 0000061 18		Primary malignant neoplasm of skin of back
4028011 0000061 14		Primary malignant neoplasm of skin of breast

4028031 0000061 15		Primary malignant neoplasm of skin of buttock
4028051 0000061 10		Primary malignant neoplasm of skin of cheek
4028061 0000061 12		Primary malignant neoplasm of skin of chest
4028071 0000061 17		Primary malignant neoplasm of skin of chin
4028091 0000061 16		Primary malignant neoplasm of skin of ear
4028101 0000061 10		Primary malignant neoplasm of skin of elbow
4028111 0000061 13		Primary malignant neoplasm of skin of external auditory canal
4028121 0000061 17		Primary malignant neoplasm of skin of eyebrow
4028141 0000061 12		Primary malignant neoplasm of skin of eyelid
4028151 0000061 14		Primary malignant neoplasm of skin of face
4028161 0000061 11		Primary malignant neoplasm of skin of finger
4028181 0000061 18		Primary malignant neoplasm of skin of foot
4028201 0000061 17		Primary malignant neoplasm of skin of forearm
4028211 0000061 19		Primary malignant neoplasm of skin of forehead
4028231 0000061 13		Primary malignant neoplasm of skin of groin
4028251 0000061 18		Primary malignant neoplasm of skin of hand
4028271 0000061 11		Primary malignant neoplasm of skin of hip
4028291 0000061 12		Primary malignant neoplasm of skin of knee
4028311 0000061 11		Primary malignant neoplasm of skin of lip

4028331 0000061 17		Primary malignant neoplasm of skin of lower limb
4028341 0000061 10		Primary malignant neoplasm of skin of neck
4028361 0000061 14		Primary malignant neoplasm of skin of nose
4028371 0000061 19		Primary malignant neoplasm of skin of perineum
4028391 0000061 18		Primary malignant neoplasm of skin of popliteal area
4028401 0000061 16		Primary malignant neoplasm of skin of scalp
4028411 0000061 18		Primary malignant neoplasm of skin of shoulder
4028431 0000061 12		Primary malignant neoplasm of skin of temporal region
4028441 0000061 19		Primary malignant neoplasm of skin of thigh
4028461 0000061 15		Primary malignant neoplasm of skin of toe
4028481 0000061 13		Primary malignant neoplasm of skin of trunk
4028491 0000061 11		Primary malignant neoplasm of skin of umbilicus
4028511 0000061 17		Primary malignant neoplasm of skin of upper limb
4028521 0000061 13		Primary malignant neoplasm of skin of arm
4028531 0000061 11		Primary malignant neoplasm of skin of wrist
4028551 0000061 16		Primary malignant neoplasm of small intestine
4028561 0000061 19		Primary malignant neoplasm of soft palate
4028571 0000061 14		Primary malignant neoplasm of soft tissues of abdomen
4028581 0000061 12		Primary malignant neoplasm of soft tissues of axilla

4028591 0000061 10		Primary malignant neoplasm of soft tissues of buttock
4028601 0000061 19		Primary malignant neoplasm of soft tissues of face
4028611 0000061 16		Primary malignant neoplasm of soft tissues of head
4028621 0000061 12		Primary malignant neoplasm of soft tissues of hip
4028631 0000061 10		Primary malignant neoplasm of soft tissues of inguinal region
4028641 0000061 17		Primary malignant neoplasm of soft tissues of lower limb
4028651 0000061 15		Primary malignant neoplasm of soft tissues of neck
4028661 0000061 18		Primary malignant neoplasm of soft tissues of pelvis
4028671 0000061 13		Primary malignant neoplasm of soft tissues of perineum
4028681 0000061 11		Primary malignant neoplasm of soft tissues of shoulder
4028691 0000061 14		Primary malignant neoplasm of soft tissues of thorax
4028701 0000061 14		Primary malignant neoplasm of soft tissues of trunk
4028711 0000061 12		Primary malignant neoplasm of sphenoid bone
4028731 0000061 18		Primary malignant neoplasm of sphenoidal sinus
4028741 0000061 11		Primary malignant neoplasm of spinal cord
4028751 0000061 13		Primary malignant neoplasm of spinal meninges
4028761 0000061 10		Primary malignant neoplasm of spleen
4028771 0000061 15		Primary malignant neoplasm of splenic flexure of colon
4028781 0000061 17		Primary malignant neoplasm of sternum

4028801 0000061 18		Primary malignant neoplasm of subglottis
4028811 0000061 15		Primary malignant neoplasm of sublingual gland
4028821 0000061 11		Primary malignant neoplasm of submaxillary gland
4028831 0000061 14		Primary malignant neoplasm of superior wall of nasopharynx
4028841 0000061 16		Primary malignant neoplasm of supraclavicular region
4028851 0000061 19		Primary malignant neoplasm of supraglottis
4028861 0000061 17		Primary malignant neoplasm of sweat gland
4028881 0000061 10		Primary malignant neoplasm of tail of pancreas
4028891 0000061 13		Primary malignant neoplasm of talus
4028911 0000061 10		Primary malignant neoplasm of tarsal bone
4028921 0000061 19		Primary malignant neoplasm of temporal bone
4028941 0000061 14		Primary malignant neoplasm of temporal lobe
4028951 0000061 11		Primary malignant neoplasm of testis
4028961 0000061 13		Primary malignant neoplasm of the mesocolon
4028971 0000061 18		Primary malignant neoplasm of omentum
4028981 0000061 15		Primary malignant neoplasm of retroperitoneum
4028991 0000061 17		Primary malignant neoplasm of thigh
4029001 0000061 17		Primary malignant neoplasm of third cuneiform bone of foot
4029011 0000061 19		Primary malignant neoplasm of thymus

4029021 0000061 10		Primary malignant neoplasm of thyroglossal duct
4029031 0000061 13		Primary malignant neoplasm of thyroid gland
4029041 0000061 15		Primary malignant neoplasm of tibia
4029061 0000061 16		Primary malignant neoplasm of tongue
4029071 0000061 11		Primary malignant neoplasm of tonsillar fossa
4029081 0000061 14		Primary malignant neoplasm of tonsillar pillar
4029091 0000061 12		Primary malignant neoplasm of trachea
4029101 0000061 18		Primary malignant neoplasm of transverse colon
4029111 0000061 15		Primary malignant neoplasm of trapezoid bone
4029121 0000061 11		Primary malignant neoplasm of trigeminal nerve
4029131 0000061 14		Primary malignant neoplasm of trigone of urinary bladder
4029141 0000061 16		Primary malignant neoplasm of trochlear nerve
4029151 0000061 19		Primary malignant neoplasm of trunk
4029161 0000061 17		Primary malignant neoplasm of ulna
4029181 0000061 10		Primary malignant neoplasm of undescended testis
4029191 0000061 13		Primary malignant neoplasm of upper inner quadrant of female breast
4029201 0000061 11		Primary malignant neoplasm of upper limb
4029211 0000061 14		Primary malignant neoplasm of upper outer quadrant of female breast
4029221 0000061 18		Primary malignant neoplasm of upper respiratory tract

4029231 0000061 15		Primary malignant neoplasm of urachus
4029241 0000061 13		Primary malignant neoplasm of ureter
4029251 0000061 10		Primary malignant neoplasm of ureteric orifice of urinary bladder
4029261 0000061 12		Primary malignant neoplasm of urethra
4029271 0000061 17		Primary malignant neoplasm of urinary bladder neck
4029281 0000061 19		Primary malignant neoplasm of urinary system
4029291 0000061 16		Primary malignant neoplasm of uterine adnexa
4029301 0000061 15		Primary malignant neoplasm of uveal tract (primary)
4029311 0000061 17		Primary malignant neoplasm of uveal tract, primary
4029321 0000061 13		Primary malignant neoplasm of uvula
4029331 0000061 11		Primary malignant neoplasm of vagus nerve
4029341 0000061 18		Primary malignant neoplasm of vallecula
4029351 0000061 16		Primary malignant neoplasm of vas deferens
4029361 0000061 19		Primary malignant neoplasm of ventral surface of tongue
4029381 0000061 12		Primary malignant neoplasm of vermilion border of lip
4029391 0000061 10		Primary malignant neoplasm of vestibule of mouth
4029401 0000061 12		Primary malignant neoplasm of vestibule of nose
4029411 0000061 10		Primary malignant neoplasm of visceral pleura
4029421 0000061 19		Primary malignant neoplasm of vomer

4029441 0000061 14		Primary malignant neoplasm of vulva
4029451 0000061 11		Primary malignant neoplasm of Waldeyer's ring
4029461 0000061 13		Primary malignant neoplasm of Waldeyer ring
4029471 0000061 18		Primary malignant neoplasm of zygomatic bone
4029571 0000061 19		Secondary malignant neoplasm of abdominal oesophagus
4029581 0000061 16		Secondary malignant neoplasm of abdominal esophagus
4029591 0000061 18		Metastatic malignant neoplasm to abdominal esophagus
4029601 0000061 14		Metastatic malignant neoplasm to abdominal oesophagus
4029611 0000061 12		Secondary malignant neoplasm of abducens nerve
4029621 0000061 16		Metastatic malignant neoplasm to abducens nerve
4029631 0000061 18		Secondary malignant neoplasm of accessory nerve
4029641 0000061 11		Metastatic malignant neoplasm to accessory nerve
4029651 0000061 13		Secondary malignant neoplasm of accessory sinus
4029661 0000061 10		Metastatic malignant neoplasm to accessory sinus
4029671 0000061 15		Secondary malignant neoplasm of acoustic nerve
4029681 0000061 17		Metastatic malignant neoplasm to acoustic nerve
4029691 0000061 19		Secondary malignant neoplasm of acromion
4029701 0000061 19		Metastatic malignant neoplasm to acromion
4029711 0000061 16		Secondary malignant neoplasm of adenoid

4029721 0000061 12		Metastatic malignant neoplasm to adenoid
4029731 0000061 10		Secondary malignant neoplasm of adnexa of skin
4029741 0000061 17		Metastatic malignant neoplasm to adnexa of skin
4029751 0000061 15		Secondary malignant neoplasm of adrenal cortex
4029761 0000061 18		Metastatic malignant neoplasm to adrenal cortex
4029781 0000061 11		Metastatic malignant neoplasm to adrenal gland
4029811 0000061 13		Cancer metastatic to adrenal gland
4029821 0000061 17		Secondary malignant neoplasm of adrenal medulla
4029831 0000061 19		Metastatic malignant neoplasm to adrenal medulla
4029841 0000061 12		Secondary malignant neoplasm of alveolar ridge mucosa
4029851 0000061 14		Metastatic malignant neoplasm to alveolar ridge mucosa
4029861 0000061 11		Secondary malignant neoplasm of ampulla of Vater
4029871 0000061 16		Metastatic malignant neoplasm to ampulla of Vater
4029881 0000061 18		Secondary malignant neoplasm of anal canal
4029891 0000061 15		Metastatic malignant neoplasm to anal canal
4029901 0000061 16		Secondary malignant neoplasm of anterior aspect of epiglottis
4029911 0000061 18		Metastatic malignant neoplasm to anterior aspect of epiglottis
4029921 0000061 14		Secondary malignant neoplasm of anterior mediastinum
4029931 0000061 12		Metastatic malignant neoplasm to anterior mediastinum

4029941 0000061 19		Secondary malignant neoplasm of anterior portion of floor of mouth
4029951 0000061 17		Metastatic malignant neoplasm to anterior portion of floor of mouth
4029961 0000061 15		Secondary malignant neoplasm of anterior two-thirds of tongue
4029971 0000061 10		Metastatic malignant neoplasm to anterior two-thirds of tongue
4029981 0000061 13		Secondary malignant neoplasm of anterior wall of nasopharynx
4029991 0000061 11		Metastatic malignant neoplasm to anterior wall of nasopharynx
4030001 0000061 10		Secondary malignant neoplasm of anterior wall of urinary bladder
4030011 0000061 13		Metastatic malignant neoplasm to anterior wall of urinary bladder
4030021 0000061 17		Secondary malignant neoplasm of anus
4030031 0000061 19		Metastatic malignant neoplasm to anus
4030041 0000061 12		Secondary malignant neoplasm of aortic body
4030051 0000061 14		Metastatic malignant neoplasm to aortic body
4030061 0000061 11		Secondary malignant neoplasm of appendix
4030071 0000061 16		Metastatic malignant neoplasm to appendix
4030081 0000061 18		Secondary malignant neoplasm of areola of female breast
4030091 0000061 15		Metastatic malignant neoplasm to areola of female breast
4030101 0000061 14		Secondary malignant neoplasm of areola of male breast
4030111 0000061 12		Metastatic malignant neoplasm to areola of male breast
4030121 0000061 16		Secondary malignant neoplasm of ascending colon

4030131000006118		Metastatic malignant neoplasm to ascending colon
4030141000006111		Secondary malignant neoplasm of axilla
4030151000006113		Metastatic malignant neoplasm to axilla
4030171000006115		Metastatic malignant neoplasm to axillary lymph nodes
4030181000006117		Secondary malignant neoplasm of axillary tail of female breast
4030191000006119		Metastatic malignant neoplasm to axillary tail of female breast
4030201000006116		Secondary malignant neoplasm of back
4030211000006118		Metastatic malignant neoplasm to back
4030221000006114		Secondary malignant neoplasm of base of tongue
4030231000006112		Metastatic malignant neoplasm to base of tongue
4030241000006119		Secondary malignant neoplasm of biliary tract
4030251000006117		Metastatic malignant neoplasm to biliary tract
4030271000006110		Metastatic malignant neoplasm to bladder
4030311000006110		Cancer metastatic to urinary bladder
4030321000006119		Secondary malignant neoplasm of blood vessel of abdomen
4030331000006116		Metastatic malignant neoplasm to blood vessel of abdomen
4030341000006114		Secondary malignant neoplasm of blood vessel of axilla
4030351000006111		Metastatic malignant neoplasm to blood vessel of axilla
4030361000006113		Secondary malignant neoplasm of blood vessel of buttock

4030371 0000061 18		Metastatic malignant neoplasm to blood vessel of buttock
4030381 0000061 15		Secondary malignant neoplasm of blood vessel of face
4030391 0000061 17		Metastatic malignant neoplasm to blood vessel of face
4030401 0000061 15		Secondary malignant neoplasm of blood vessel of finger
4030411 0000061 17		Metastatic malignant neoplasm to blood vessel of finger
4030421 0000061 13		Secondary malignant neoplasm of blood vessel of foot
4030431 0000061 11		Metastatic malignant neoplasm to blood vessel of foot
4030441 0000061 18		Secondary malignant neoplasm of blood vessel of forearm
4030451 0000061 16		Metastatic malignant neoplasm to blood vessel of forearm
4030461 0000061 19		Secondary malignant neoplasm of blood vessel of hand
4030471 0000061 14		Metastatic malignant neoplasm to blood vessel of hand
4030481 0000061 12		Secondary malignant neoplasm of blood vessel of head
4030491 0000061 10		Metastatic malignant neoplasm to blood vessel of head
4030501 0000061 19		Secondary malignant neoplasm of blood vessel of hip
4030511 0000061 16		Metastatic malignant neoplasm to blood vessel of hip
4030521 0000061 12		Secondary malignant neoplasm of blood vessel of inguinal region
4030531 0000061 10		Metastatic malignant neoplasm to blood vessel of inguinal region
4030541 0000061 17		Secondary malignant neoplasm of blood vessel of lower limb
4030551 0000061 15		Metastatic malignant neoplasm to blood vessel of lower limb

4030561000006118		Secondary malignant neoplasm of blood vessel of neck
4030571000006113		Metastatic malignant neoplasm to blood vessel of neck
4030581000006111		Secondary malignant neoplasm of blood vessel of pelvis
4030591000006114		Metastatic malignant neoplasm to blood vessel of pelvis
4030601000006118		Secondary malignant neoplasm of blood vessel of perineum
4030611000006115		Metastatic malignant neoplasm to blood vessel of perineum
4030621000006111		Secondary malignant neoplasm of blood vessel of popliteal space
4030631000006114		Metastatic malignant neoplasm to blood vessel of popliteal space
4030641000006116		Secondary malignant neoplasm of blood vessel of shoulder
4030651000006119		Metastatic malignant neoplasm to blood vessel of shoulder
4030661000006117		Secondary malignant neoplasm of blood vessel of thigh
4030671000006112		Metastatic malignant neoplasm to blood vessel of thigh
4030681000006110		Secondary malignant neoplasm of blood vessel of thorax
4030691000006113		Metastatic malignant neoplasm to blood vessel of thorax
4030701000006113		Secondary malignant neoplasm of blood vessel of toe
4030711000006111		Metastatic malignant neoplasm to blood vessel of toe
4030721000006115		Secondary malignant neoplasm of blood vessel of trunk
4030731000006117		Metastatic malignant neoplasm to blood vessel of trunk
4030741000006110		Secondary malignant neoplasm of blood vessel of upper limb

4030751 0000061 12		Metastatic malignant neoplasm to blood vessel of upper limb
4030761 0000061 14		Secondary malignant neoplasm of blood vessel
4030771 0000061 19		Metastatic malignant neoplasm to blood vessel
4030781 0000061 16		Secondary malignant neoplasm of body of pancreas
4030791 0000061 18		Metastatic malignant neoplasm to body of pancreas
4030801 0000061 17		Secondary malignant neoplasm of body of penis
4030811 0000061 19		Metastatic malignant neoplasm to body of penis
4030821 0000061 10		Secondary malignant neoplasm of body of stomach
4030831 0000061 13		Metastatic malignant neoplasm to body of stomach
4030841 0000061 15		Secondary malignant neoplasm of body of uterus
4030851 0000061 18		Metastatic malignant neoplasm to body of uterus
4030861 0000061 16		Secondary malignant neoplasm of corpus uteri
4030871 0000061 11		Metastatic malignant neoplasm to corpus uteri
4030881 0000061 14		Secondary malignant neoplasm of bone marrow
4030891 0000061 12		Metastatic malignant neoplasm to bone marrow
4030911 0000061 14		Cancer metastatic to bone marrow
4030921 0000061 18		Secondary malignant neoplasm of bone of face
4030931 0000061 15		Metastatic malignant neoplasm to bone of face
4030941 0000061 13		Secondary malignant neoplasm of bone of lower limb

4030951 0000061 10		Metastatic malignant neoplasm to bone of lower limb
4030961 0000061 12		Secondary malignant neoplasm of bone of skull
4030971 0000061 17		Metastatic malignant neoplasm to bone of skull
4030981 0000061 19		Secondary malignant neoplasm of bone of upper limb
4030991 0000061 16		Metastatic malignant neoplasm to bone of upper limb
4031001 0000061 15		Secondary malignant neoplasm of bone
4031011 0000061 17		Metastatic malignant neoplasm to bone
4031041 0000061 18		Secondary cancer of bone
4031081 0000061 12		Cancer metastatic to bone
4031111 0000061 18		Secondary malignant neoplasm of brain stem
4031121 0000061 14		Metastatic malignant neoplasm to brain stem
4031141 0000061 19		Metastatic malignant neoplasm to brain
4031151 0000061 17		Secondary cancer of brain
4031171 0000061 10		Cancer metastatic to brain
4031181 0000061 13		Secondary malignant neoplasm of broad ligament
4031191 0000061 11		Metastatic malignant neoplasm to broad ligament
4031201 0000061 14		Secondary malignant neoplasm of bronchopulmonary lymph nodes
4031211 0000061 12		Metastatic malignant neoplasm to bronchopulmonary lymph nodes
4031221 0000061 16		Metastatic malignant neoplasm to hilar lymph nodes

4031231 0000061 18		Secondary malignant neoplasm of hilar lymph nodes
4031241 0000061 11		Secondary malignant neoplasm of bronchus of left lower lobe
4031251 0000061 13		Metastatic malignant neoplasm to bronchus of left lower lobe
4031261 0000061 10		Secondary malignant neoplasm of bronchus of left upper lobe
4031271 0000061 15		Metastatic malignant neoplasm to bronchus of left upper lobe
4031281 0000061 17		Secondary malignant neoplasm of bronchus of right lower lobe
4031291 0000061 19		Metastatic malignant neoplasm to bronchus of right lower lobe
4031301 0000061 18		Secondary malignant neoplasm of bronchus of right middle lobe
4031311 0000061 15		Metastatic malignant neoplasm to bronchus of right middle lobe
4031321 0000061 11		Secondary malignant neoplasm of bronchus of right upper lobe
4031331 0000061 14		Metastatic malignant neoplasm to bronchus of right upper lobe
4031341 0000061 16		Secondary malignant neoplasm of bronchus
4031351 0000061 19		Metastatic malignant neoplasm to bronchus
4031361 0000061 17		Secondary malignant tumour of bronchus
4031371 0000061 12		Secondary malignant tumor of bronchus
4031391 0000061 13		Secondary malignant neoplasm of buccal mucosa
4031401 0000061 10		Metastatic malignant neoplasm to buccal mucosa
4031411 0000061 13		Secondary malignant neoplasm of caecum
4031421 0000061 17		Secondary malignant neoplasm of cecum

4031431 0000061 19		Metastatic malignant neoplasm to cecum
4031441 0000061 12		Metastatic malignant neoplasm to caecum
4031451 0000061 14		Secondary malignant neoplasm of calcaneus
4031461 0000061 11		Metastatic malignant neoplasm to calcaneus
4031471 0000061 16		Secondary malignant neoplasm of cardia of stomach
4031481 0000061 18		Metastatic malignant neoplasm to cardia of stomach
4031491 0000061 15		Secondary malignant neoplasm of carina
4031501 0000061 11		Metastatic malignant neoplasm to carina
4031511 0000061 14		Secondary malignant neoplasm of carotid body
4031521 0000061 18		Metastatic malignant neoplasm to carotid body
4031531 0000061 15		Secondary malignant neoplasm of carpal bone
4031541 0000061 13		Metastatic malignant neoplasm to carpal bone
4031551 0000061 10		Secondary malignant neoplasm of cartilage of nose
4031561 0000061 12		Metastatic malignant neoplasm to cartilage of nose
4031571 0000061 17		Secondary malignant neoplasm of cauda equina
4031581 0000061 19		Metastatic malignant neoplasm to cauda equina
4031591 0000061 16		Secondary malignant neoplasm of central nervous system
4031601 0000061 12		Metastatic malignant neoplasm to central nervous system
4031611 0000061 10		Cancer metastatic to CNS (central nervous system)

4031621 0000061 19		Secondary malignant neoplasm of central portion of female breast
4031631 0000061 16		Metastatic malignant neoplasm to central portion of female breast
4031641 0000061 14		Secondary malignant neoplasm of cerebellum
4031651 0000061 11		Metastatic malignant neoplasm to cerebellum
4031661 0000061 13		Secondary malignant neoplasm of cerebral meninges
4031671 0000061 18		Metastatic malignant neoplasm to cerebral meninges
4031681 0000061 15		Cancer metastatic to cerebral meninges
4031691 0000061 17		Secondary malignant neoplasm of cerebral ventricle
4031701 0000061 17		Metastatic malignant neoplasm to cerebral ventricle
4031711 0000061 19		Secondary malignant neoplasm of cerebrum
4031721 0000061 10		Metastatic malignant neoplasm to cerebrum
4031741 0000061 15		Secondary malignant neoplasm of cervical oesophagus
4031751 0000061 18		Secondary malignant neoplasm of cervical esophagus
4031761 0000061 16		Metastatic malignant neoplasm to cervical esophagus
4031771 0000061 11		Metastatic malignant neoplasm to cervical oesophagus
4031781 0000061 14		Secondary malignant neoplasm of cervical vertebral column
4031791 0000061 12		Metastatic malignant neoplasm to cervical vertebral column
4031801 0000061 13		Secondary malignant neoplasm of cheek
4031811 0000061 11		Metastatic malignant neoplasm to cheek

4031821000006115		Secondary malignant neoplasm of chest wall
4031831000006117		Metastatic malignant neoplasm to chest wall
4031841000006110		Cancer metastatic to chest wall
4031851000006112		Secondary malignant neoplasm of choroid
4031861000006114		Metastatic malignant neoplasm to choroid
4031901000006119		Cancer metastatic to choroid
4031911000006116		Secondary malignant neoplasm of ciliary body
4031921000006112		Metastatic malignant neoplasm to ciliary body
4031931000006110		Secondary malignant neoplasm of clavicle
4031941000006117		Metastatic malignant neoplasm to clavicle
4031951000006115		Secondary malignant neoplasm of clitoris
4031961000006118		Metastatic malignant neoplasm to clitoris
4031971000006113		Secondary malignant neoplasm of coccygeal body
4031981000006111		Metastatic malignant neoplasm to coccygeal body
4031991000006114		Secondary malignant neoplasm of coccyx
4032001000006111		Metastatic malignant neoplasm to coccyx
4032021000006118		Metastatic malignant neoplasm to colon
4032031000006115		Secondary malignant neoplasm of commissure of lip
4032041000006113		Metastatic malignant neoplasm to commissure of lip

4032051 0000061 10		Secondary malignant neoplasm of common bile duct
4032061 0000061 12		Metastatic malignant neoplasm to common bile duct
4032071 0000061 17		Secondary malignant neoplasm of conjunctiva
4032081 0000061 19		Metastatic malignant neoplasm to conjunctiva
4032091 0000061 16		Secondary malignant neoplasm of soft tissues
4032101 0000061 10		Secondary malignant neoplasm of connective and other soft tissues
4032111 0000061 13		Metastatic malignant neoplasm to connective and other soft tissues
4032131 0000061 19		Secondary malignant neoplasm of cornea
4032141 0000061 12		Metastatic malignant neoplasm to cornea
4032151 0000061 14		Secondary malignant neoplasm of cranial nerve
4032161 0000061 11		Metastatic malignant neoplasm to cranial nerve
4032171 0000061 16		Secondary malignant neoplasm of craniopharyngeal duct
4032181 0000061 18		Metastatic malignant neoplasm to craniopharyngeal duct
4032191 0000061 15		Secondary malignant neoplasm of cubital lymph nodes
4032201 0000061 17		Metastatic malignant neoplasm to cubital lymph nodes
4032211 0000061 19		Secondary malignant neoplasm of cuboid
4032221 0000061 10		Metastatic malignant neoplasm to cuboid
4032231 0000061 13		Secondary malignant neoplasm of cystic duct
4032241 0000061 15		Metastatic malignant neoplasm to cystic duct

4032251000006118		Secondary malignant neoplasm of descending colon
4032261000006116		Metastatic malignant neoplasm to descending colon
4032271000006111		Secondary malignant neoplasm of diaphragm
4032281000006114		Metastatic malignant neoplasm to diaphragm
4032291000006112		Secondary malignant neoplasm of dorsal surface of tongue
4032301000006113		Metastatic malignant neoplasm to dorsal surface of tongue
4032311000006111		Secondary malignant neoplasm of thoracic vertebral column
4032321000006115		Metastatic malignant neoplasm to thoracic vertebral column
4032331000006117		Secondary malignant neoplasm of dorsal vertebral column
4032341000006110		Metastatic malignant neoplasm to dorsal vertebral column
4032361000006114		Metastatic malignant neoplasm to duodenum
4032371000006119		Cancer metastatic to duodenum
4032381000006116		Secondary malignant neoplasm of ectopic female breast tissue
4032391000006118		Metastatic malignant neoplasm to ectopic female breast tissue
4032401000006116		Secondary malignant neoplasm of ectopic male breast tissue
4032411000006118		Metastatic malignant neoplasm to ectopic male breast tissue
4032421000006114		Secondary malignant neoplasm of endocardium
4032431000006112		Metastatic malignant neoplasm to endocardium
4032441000006119		Secondary malignant neoplasm of endocervix

4032451000006117		Metastatic malignant neoplasm to endocervix
4032461000006115		Secondary malignant neoplasm of endocrine gland
4032471000006110		Metastatic malignant neoplasm to endocrine gland
4032491000006111		Secondary malignant neoplasm of endometrium
4032501000006115		Metastatic malignant neoplasm to endometrium
4032511000006117		Secondary malignant neoplasm of epicardium
4032521000006113		Metastatic malignant neoplasm to epicardium
4032531000006111		Secondary malignant neoplasm of epididymis
4032541000006118		Metastatic malignant neoplasm to epididymis
4032581000006112		Secondary malignant neoplasm of epiglottis
4032591000006110		Metastatic malignant neoplasm to epiglottis
4032601000006119		Secondary malignant neoplasm of epitrochlear lymph nodes
4032611000006116		Metastatic malignant neoplasm to epitrochlear lymph nodes
4032621000006112		Secondary malignant neoplasm of oesophagus
4032631000006110		Secondary malignant neoplasm of esophagus
4032641000006117		Metastatic malignant neoplasm to esophagus
4032651000006115		Metastatic malignant neoplasm to oesophagus
4032661000006118		Cancer metastatic to esophagus
4032671000006113		Secondary malignant neoplasm of ethmoid bone

4032681 0000061 11		Metastatic malignant neoplasm to ethmoid bone
4032691 0000061 14		Secondary malignant neoplasm of ethmoidal sinus
4032701 0000061 14		Metastatic malignant neoplasm to ethmoidal sinus
4032711 0000061 12		Secondary malignant neoplasm of eustachian tube
4032721 0000061 16		Metastatic malignant neoplasm to eustachian tube
4032731 0000061 18		Secondary malignant neoplasm of exocervix
4032741 0000061 11		Metastatic malignant neoplasm to exocervix
4032751 0000061 13		Secondary malignant neoplasm of extrahepatic bile ducts
4032761 0000061 10		Metastatic malignant neoplasm to extrahepatic bile ducts
4032771 0000061 15		Secondary malignant neoplasm of eye
4032781 0000061 17		Metastatic malignant neoplasm to eye
4032801 0000061 18		Secondary malignant neoplasm of face
4032811 0000061 15		Metastatic malignant neoplasm to face
4032821 0000061 11		Secondary malignant neoplasm of facial nerve
4032831 0000061 14		Metastatic malignant neoplasm to facial nerve
4032841 0000061 16		Secondary malignant neoplasm of fallopian tube
4032851 0000061 19		Metastatic malignant neoplasm to fallopian tube
4032861 0000061 17		Secondary malignant neoplasm of false vocal cord
4032871 0000061 12		Metastatic malignant neoplasm to false vocal cord

4032881000006110		Secondary malignant neoplasm of ventricular bands of larynx
4032891000006113		Metastatic malignant neoplasm to ventricular bands of larynx
4032901000006112		Secondary malignant neoplasm of female breast
4032911000006110		Metastatic malignant neoplasm to female breast
4032931000006116		Secondary malignant deposit to breast
4032951000006111		Secondary malignant neoplasm of female genital organ
4032961000006113		Metastatic malignant neoplasm to female genital organ
4032981000006115		Cancer metastatic to female genitalia
4032991000006117		Cancer metastatic to female genital organ
4033001000006116		Secondary malignant neoplasm of femoral lymph nodes
4033011000006118		Metastatic malignant neoplasm to femoral lymph nodes
4033021000006114		Secondary malignant neoplasm of femur
4033031000006112		Metastatic malignant neoplasm to femur
4033041000006119		Secondary malignant neoplasm of fibula
4033051000006117		Metastatic malignant neoplasm to fibula
4033061000006115		Secondary malignant neoplasm of first cuneiform bone of foot
4033071000006110		Metastatic malignant neoplasm to first cuneiform bone of foot
4033081000006113		Secondary malignant neoplasm of flank
4033091000006111		Metastatic malignant neoplasm to flank

4033101000006117		Secondary malignant neoplasm of floor of mouth
4033111000006119		Metastatic malignant neoplasm to floor of mouth
4033121000006110		Secondary malignant neoplasm of foot
4033131000006113		Metastatic malignant neoplasm to foot
4033141000006115		Secondary malignant neoplasm of forearm
4033151000006118		Metastatic malignant neoplasm to forearm
4033161000006116		Secondary malignant neoplasm of prepuce
4033171000006111		Metastatic malignant neoplasm to prepuce
4033181000006114		Secondary malignant neoplasm of foreskin
4033191000006112		Metastatic malignant neoplasm to foreskin
4033201000006110		Secondary malignant neoplasm of frontal bone
4033211000006113		Metastatic malignant neoplasm to frontal bone
4033221000006117		Secondary malignant neoplasm of frontal lobe
4033231000006119		Metastatic malignant neoplasm to frontal lobe
4033241000006112		Secondary malignant neoplasm of frontal sinus
4033251000006114		Metastatic malignant neoplasm to frontal sinus
4033261000006111		Secondary malignant neoplasm of fundus of stomach
4033271000006116		Metastatic malignant neoplasm to fundus of stomach
4033281000006118		Secondary malignant neoplasm of gallbladder

4033291000006115		Metastatic malignant neoplasm to gallbladder
4033301000006119		Cancer metastatic to gallbladder
4033321000006112		Metastatic malignant neoplasm to gastrointestinal tract
4033351000006115		Secondary malignant neoplasm of gingival mucosa
4033361000006118		Metastatic malignant neoplasm to gingival mucosa
4033371000006113		Secondary malignant neoplasm of glans penis
4033381000006111		Metastatic malignant neoplasm to glans penis
4033391000006114		Secondary malignant neoplasm of glomus jugulare
4033401000006111		Metastatic malignant neoplasm to glomus jugulare
4033411000006114		Secondary malignant neoplasm of glossopharyngeal nerve
4033421000006118		Metastatic malignant neoplasm to glossopharyngeal nerve
4033431000006115		Secondary malignant neoplasm of glottis
4033441000006113		Metastatic malignant neoplasm to glottis
4033451000006110		Secondary malignant neoplasm of great vessels
4033461000006112		Metastatic malignant neoplasm to great vessels
4033471000006117		Secondary malignant neoplasm of greater curvature of stomach
4033481000006119		Metastatic malignant neoplasm to greater curvature of stomach
4033491000006116		Secondary malignant neoplasm of gum
4033501000006112		Metastatic malignant neoplasm to gum

4033511000006110		Secondary malignant neoplasm of hamate bone
4033521000006119		Metastatic malignant neoplasm to hamate bone
4033531000006116		Secondary malignant neoplasm of hand
4033541000006114		Metastatic malignant neoplasm to hand
4033551000006111		Secondary malignant neoplasm of hard palate
4033561000006113		Metastatic malignant neoplasm to hard palate
4033571000006118		Secondary malignant neoplasm of head of pancreas
4033581000006115		Metastatic malignant neoplasm to head of pancreas
4033591000006117		Secondary malignant neoplasm of head
4033601000006113		Metastatic malignant neoplasm to head
4033611000006111		Secondary malignant neoplasm of heart
4033621000006115		Metastatic malignant neoplasm to heart
4033631000006117		Secondary malignant tumour of heart
4033641000006110		Secondary malignant tumor of heart
4033661000006114		Cancer metastatic to heart
4033671000006119		Secondary malignant neoplasm of hepatic flexure of colon
4033681000006116		Metastatic malignant neoplasm to hepatic flexure of colon
4033691000006118		Secondary malignant neoplasm of hilus of lung
4033701000006118		Metastatic malignant neoplasm to hilus of lung

4033711000006115		Secondary malignant neoplasm of hypogastric lymph nodes
4033721000006111		Metastatic malignant neoplasm to hypogastric lymph nodes
4033731000006114		Cancer metastatic to hypogastric lymph node
4033741000006116		Secondary malignant neoplasm of hypoglossal nerve
4033751000006119		Metastatic malignant neoplasm to hypoglossal nerve
4033761000006117		Secondary malignant neoplasm of hypopharyngeal aspect of aryepiglottic fold
4033771000006112		Metastatic malignant neoplasm to hypopharyngeal aspect of aryepiglottic fold
4033781000006110		Secondary malignant neoplasm of hypopharyngeal aspect of interarytenoid fold
4033791000006113		Metastatic malignant neoplasm to hypopharyngeal aspect of interarytenoid fold
4033801000006114		Secondary malignant neoplasm of hypopharynx
4033811000006112		Metastatic malignant neoplasm to hypopharynx
4033831000006118		Metastatic malignant neoplasm to ileum
4033841000006111		Secondary malignant neoplasm of iliac lymph nodes
4033851000006113		Metastatic malignant neoplasm to iliac lymph nodes
4033861000006110		Cancer metastatic to iliac lymph nodes
4033871000006115		Secondary malignant neoplasm of ilium
4033881000006117		Metastatic malignant neoplasm to ilium
4033891000006119		Secondary malignant neoplasm of infraclavicular lymph nodes
4033901000006115		Metastatic malignant neoplasm to infraclavicular lymph nodes

4033911000006117		Secondary malignant neoplasm of inguinal lymph nodes
4033921000006113		Metastatic malignant neoplasm to inguinal lymph nodes
4033931000006111		Cancer metastatic to inguinal lymph nodes
4033941000006118		Secondary malignant neoplasm of inguinal region
4033951000006116		Metastatic malignant neoplasm to inguinal region
4033961000006119		Secondary malignant neoplasm of inner aspect of lip
4033971000006114		Metastatic malignant neoplasm to inner aspect of lip
4033981000006112		Secondary malignant neoplasm of inner aspect of lower lip
4033991000006110		Metastatic malignant neoplasm to inner aspect of lower lip
4034001000006118		Secondary malignant neoplasm of inner aspect of upper lip
4034011000006115		Metastatic malignant neoplasm to inner aspect of upper lip
4034021000006111		Secondary malignant neoplasm of intercostal lymph nodes
4034031000006114		Metastatic malignant neoplasm to intercostal lymph nodes
4034041000006116		Cancer metastatic to intercostal lymph nodes
4034051000006119		Secondary malignant neoplasm of intestinal lymph nodes
4034061000006117		Metastatic malignant neoplasm to intestinal lymph nodes
4034071000006112		Cancer metastatic to intestinal lymph nodes
4034081000006110		Secondary malignant neoplasm of intestinal tract
4034091000006113		Metastatic malignant neoplasm to intestinal tract

4034101 0000061 19		Secondary malignant neoplasm of intra-abdominal lymph nodes
4034111 0000061 16		Metastatic malignant neoplasm to intra-abdominal lymph nodes
4034121 0000061 12		Cancer metastatic to intra-abdominal lymph nodes
4034131 0000061 10		Secondary malignant neoplasm of intra-abdominal organs
4034141 0000061 17		Metastatic malignant neoplasm to intra-abdominal organs
4034151 0000061 15		Cancer metastatic to abdominal organs
4034161 0000061 18		Cancer metastatic to intra-abdominal organs
4034171 0000061 13		Secondary malignant neoplasm of intrahepatic bile ducts
4034181 0000061 11		Metastatic malignant neoplasm to intrahepatic bile ducts
4034191 0000061 14		Secondary malignant neoplasm of intrapelvic lymph nodes
4034201 0000061 12		Metastatic malignant neoplasm to intrapelvic lymph nodes
4034211 0000061 10		Cancer metastatic to intrapelvic lymph nodes
4034221 0000061 19		Secondary malignant neoplasm of intrathoracic lymph nodes
4034231 0000061 16		Metastatic malignant neoplasm to intrathoracic lymph nodes
4034241 0000061 14		Cancer metastatic to intrathoracic lymph nodes
4034251 0000061 11		Secondary malignant neoplasm of intrathoracic organs
4034261 0000061 13		Metastatic malignant neoplasm to intrathoracic organs
4034271 0000061 18		Secondary malignant neoplasm of ischium
4034281 0000061 15		Metastatic malignant neoplasm to ischium

4034291 0000061 17		Secondary malignant neoplasm of islets of Langerhans
4034301 0000061 16		Metastatic malignant neoplasm to islets of Langerhans
4034311 0000061 18		Secondary malignant neoplasm of isthmus of uterus
4034321 0000061 14		Metastatic malignant neoplasm to isthmus of uterus
4034331 0000061 12		Secondary malignant neoplasm of jaw
4034341 0000061 19		Metastatic malignant neoplasm to jaw
4034361 0000061 15		Metastatic malignant neoplasm to jejunum
4034371 0000061 10		Secondary malignant neoplasm of junctional region of epiglottis
4034381 0000061 13		Metastatic malignant neoplasm to junctional region of epiglottis
4034391 0000061 11		Secondary malignant neoplasm of junctional zone of tongue
4034401 0000061 13		Metastatic malignant neoplasm to junctional zone of tongue
4034421 0000061 15		Metastatic malignant neoplasm to kidney
4034461 0000061 14		Secondary renal cancer
4034481 0000061 16		Secondary cancer of kidney
4034491 0000061 18		Cancer metastatic to kidney
4034501 0000061 14		Secondary malignant neoplasm of labia majora
4034511 0000061 12		Metastatic malignant neoplasm to labia majora
4034521 0000061 16		Secondary malignant neoplasm of labia minora
4034531 0000061 18		Metastatic malignant neoplasm to labia minora

4034541<br>0000061<br>11		Secondary malignant neoplasm of lacrimal gland
4034551<br>0000061<br>13		Metastatic malignant neoplasm to lacrimal gland
4034561<br>0000061<br>10		Secondary malignant neoplasm of large intestine
4034571<br>0000061<br>15		Metastatic malignant neoplasm to large intestine
4034591<br>0000061<br>19		Cancer metastatic to large intestine
4034601<br>0000061<br>10		Secondary malignant neoplasm of laryngeal aspect of aryepiglottic fold
4034611<br>0000061<br>13		Metastatic malignant neoplasm to laryngeal aspect of aryepiglottic fold
4034621<br>0000061<br>17		Secondary malignant neoplasm of laryngeal aspect of interarytenoid fold
4034631<br>0000061<br>19		Metastatic malignant neoplasm to laryngeal aspect of interarytenoid fold
4034641<br>0000061<br>12		Secondary malignant neoplasm of laryngeal commissure
4034651<br>0000061<br>14		Metastatic malignant neoplasm to laryngeal commissure
4034661<br>0000061<br>11		Secondary malignant neoplasm of laryngeal surface of epiglottis
4034671<br>0000061<br>16		Metastatic malignant neoplasm to laryngeal surface of epiglottis
4034681<br>0000061<br>18		Secondary malignant neoplasm of larynx
4034691<br>0000061<br>15		Metastatic malignant neoplasm to larynx
4034701<br>0000061<br>15		Secondary malignant neoplasm of lateral portion of floor of mouth
4034711<br>0000061<br>17		Metastatic malignant neoplasm to lateral portion of floor of mouth
4034721<br>0000061<br>13		Secondary malignant neoplasm of lateral wall of nasopharynx
4034731<br>0000061<br>11		Metastatic malignant neoplasm to lateral wall of nasopharynx

4034741000006118		Secondary malignant neoplasm of lateral wall of oropharynx
4034751000006116		Metastatic malignant neoplasm to lateral wall of oropharynx
4034761000006119		Secondary malignant neoplasm of lateral wall of urinary bladder
4034771000006114		Metastatic malignant neoplasm to lateral wall of urinary bladder
4034781000006112		Secondary malignant neoplasm of left lower lobe of lung
4034791000006110		Metastatic malignant neoplasm to left lower lobe of lung
4034801000006111		Secondary malignant neoplasm of left upper lobe of lung
4034811000006114		Metastatic malignant neoplasm to left upper lobe of lung
4034821000006118		Secondary malignant neoplasm of lesser curvature of stomach
4034831000006115		Metastatic malignant neoplasm to lesser curvature of stomach
4034841000006113		Secondary malignant neoplasm of lingual tonsil
4034851000006110		Metastatic malignant neoplasm to lingual tonsil
4034861000006112		Secondary malignant neoplasm of lip
4034871000006117		Metastatic malignant neoplasm to lip
4034891000006116		Metastatic malignant neoplasm to liver
4034901000006117		Liver secondary cancer
4034951000006118		Cancer metastatic to liver
4034961000006116		Secondary malignant neoplasm of long bone of lower limb
4034971000006111		Metastatic malignant neoplasm to long bone of lower limb

4034981 0000061 14		Secondary malignant neoplasm of long bone of upper limb
4034991 0000061 12		Metastatic malignant neoplasm to long bone of upper limb
4035001 0000061 17		Secondary malignant neoplasm of lower gum
4035011 0000061 19		Metastatic malignant neoplasm to lower gum
4035021 0000061 10		Secondary malignant neoplasm of lower inner quadrant of female breast
4035031 0000061 13		Metastatic malignant neoplasm to lower inner quadrant of female breast
4035041 0000061 15		Secondary malignant neoplasm of lower limb
4035051 0000061 18		Metastatic malignant neoplasm to lower limb
4035061 0000061 16		Secondary malignant neoplasm of lower outer quadrant of female breast
4035071 0000061 11		Metastatic malignant neoplasm to lower outer quadrant of female breast
4035081 0000061 14		Secondary malignant neoplasm of lower third of oesophagus
4035091 0000061 12		Secondary malignant neoplasm of lower third of esophagus
4035101 0000061 18		Metastatic malignant neoplasm to lower third of esophagus
4035111 0000061 15		Metastatic malignant neoplasm to lower third of oesophagus
4035121 0000061 11		Secondary malignant neoplasm of lumbar vertebral column
4035131 0000061 14		Metastatic malignant neoplasm to lumbar vertebral column
4035141 0000061 16		Secondary malignant neoplasm of lunate bone
4035151 0000061 19		Metastatic malignant neoplasm to lunate bone
4035171 0000061 12		Metastatic malignant neoplasm to lung

4035181000006110		Secondary malignant tumour of lung
4035191000006113		Secondary malignant tumor of lung
4035211000006114		Secondary cancer of lung
4035221000006118		Cancer metastatic to lung
4035241000006113		Metastatic malignant neoplasm to lymph node
4035251000006110		Secondary lymph node cancer
4035261000006112		Cancer metastatic to lymph nodes
4035271000006117		Secondary malignant neoplasm of lymph nodes of face
4035281000006119		Metastatic malignant neoplasm to lymph nodes of face
4035291000006116		Cancer metastatic to lymph nodes of face
4035311000006117		Metastatic malignant neoplasm to lymph nodes of head
4035321000006113		Cancer metastatic to lymph nodes of head
4035331000006111		Secondary malignant neoplasm of lymph nodes of lower limb
4035341000006118		Metastatic malignant neoplasm to lymph nodes of lower limb
4035361000006119		Cancer metastatic to lymph nodes of lower limb
4035371000006114		Secondary malignant neoplasm of lymph nodes of multiple sites
4035381000006112		Metastatic malignant neoplasm to lymph nodes of multiple sites
4035391000006110		Cancer metastatic to lymph nodes of multiple sites
4035401000006112		Secondary malignant neoplasm of lymph nodes of neck

4035411000006110		Metastatic malignant neoplasm to lymph nodes of neck
4035431000006116		Cancer metastatic to neck lymph nodes
4035451000006111		Metastatic malignant neoplasm to lymph nodes of upper limb
4035471000006118		Cancer metastatic to lymph nodes of upper limb
4035481000006115		Secondary malignant neoplasm of main bronchus
4035491000006117		Metastatic malignant neoplasm to main bronchus
4035501000006113		Secondary malignant neoplasm of major salivary gland
4035511000006111		Metastatic malignant neoplasm to major salivary gland
4035521000006115		Secondary malignant neoplasm of male breast
4035531000006117		Metastatic malignant neoplasm to male breast
4035541000006110		Secondary malignant neoplasm of male genital organ
4035551000006112		Metastatic malignant neoplasm to male genital organ
4035571000006119		Cancer metastatic to male genital organ
4035581000006116		Cancer metastatic to male genitalia
4035591000006118		Secondary malignant neoplasm of mandible
4035601000006114		Metastatic malignant neoplasm to mandible
4035611000006112		Secondary malignant neoplasm of mastoid air cells
4035621000006116		Metastatic malignant neoplasm to mastoid air cells
4035631000006118		Secondary malignant neoplasm of maxilla

4035641000006111		Metastatic malignant neoplasm to maxilla
4035651000006113		Secondary malignant neoplasm of maxillary sinus
4035661000006110		Metastatic malignant neoplasm to maxillary sinus
4035671000006115		Secondary malignant neoplasm of Meckel's diverticulum
4035681000006117		Metastatic malignant neoplasm to Meckel's diverticulum
4035691000006119		Secondary malignant neoplasm of Meckel diverticulum
4035711000006116		Metastatic malignant neoplasm to mediastinal lymph nodes
4035731000006110		Metastatic malignant neoplasm to mediastinum
4035741000006117		Secondary malignant tumour of mediastinum
4035751000006115		Secondary malignant tumor of mediastinum
4035781000006111		Cancer metastatic to mediastinum
4035801000006110		Metastatic malignant neoplasm to mesenteric lymph nodes
4035811000006113		Cancer metastatic to mesenteric lymph nodes
4035821000006117		Secondary malignant neoplasm of metacarpal bone
4035831000006119		Metastatic malignant neoplasm to metacarpal bone
4035841000006112		Secondary malignant neoplasm of metatarsal bone
4035851000006114		Metastatic malignant neoplasm to metatarsal bone
4035861000006111		Secondary malignant neoplasm of middle ear
4035871000006116		Metastatic malignant neoplasm to middle ear

4035881 0000061 18		Secondary malignant neoplasm of middle third of oesophagus
4035891 0000061 15		Secondary malignant neoplasm of middle third of esophagus
4035901 0000061 16		Metastatic malignant neoplasm to middle third of esophagus
4035911 0000061 18		Metastatic malignant neoplasm to middle third of oesophagus
4035921 0000061 14		Secondary malignant neoplasm of minor salivary gland
4035931 0000061 12		Metastatic malignant neoplasm to minor salivary gland
4035941 0000061 19		Secondary malignant neoplasm of mouth
4035951 0000061 17		Metastatic malignant neoplasm to mouth
4035961 0000061 15		Secondary malignant neoplasm of multiple endocrine glands
4035971 0000061 10		Metastatic malignant neoplasm to multiple endocrine glands
4035981 0000061 13		Secondary malignant neoplasm of muscle of abdomen
4035991 0000061 11		Metastatic malignant neoplasm to muscle of abdomen
4036001 0000061 12		Secondary malignant neoplasm of muscle of buttock
4036011 0000061 10		Metastatic malignant neoplasm to muscle of buttock
4036021 0000061 19		Secondary malignant neoplasm of muscle of face
4036031 0000061 16		Metastatic malignant neoplasm to muscle of face
4036041 0000061 14		Secondary malignant neoplasm of muscle of head
4036051 0000061 11		Metastatic malignant neoplasm to muscle of head
4036061 0000061 13		Secondary malignant neoplasm of muscle of hip

4036071 0000061 18		Metastatic malignant neoplasm to muscle of hip
4036081 0000061 15		Secondary malignant neoplasm of muscle of inguinal region
4036091 0000061 17		Metastatic malignant neoplasm to muscle of inguinal region
4036101 0000061 11		Secondary malignant neoplasm of muscle of lower limb
4036111 0000061 14		Metastatic malignant neoplasm to muscle of lower limb
4036121 0000061 18		Secondary malignant neoplasm of muscle of neck
4036131 0000061 15		Metastatic malignant neoplasm to muscle of neck
4036141 0000061 13		Secondary malignant neoplasm of muscle of pelvis
4036151 0000061 10		Metastatic malignant neoplasm to muscle of pelvis
4036161 0000061 12		Secondary malignant neoplasm of muscle of perineum
4036171 0000061 17		Metastatic malignant neoplasm to muscle of perineum
4036181 0000061 19		Secondary malignant neoplasm of muscle of shoulder
4036191 0000061 16		Metastatic malignant neoplasm to muscle of shoulder
4036201 0000061 18		Secondary malignant neoplasm of muscle of thorax
4036211 0000061 15		Metastatic malignant neoplasm to muscle of thorax
4036221 0000061 11		Secondary malignant neoplasm of muscle of trunk
4036231 0000061 14		Metastatic malignant neoplasm to muscle of trunk
4036241 0000061 16		Secondary malignant neoplasm of muscle of upper limb
4036251 0000061 19		Metastatic malignant neoplasm to muscle of upper limb

4036261000006117		Secondary malignant neoplasm of muscle
4036271000006112		Metastatic malignant neoplasm to muscle
4036281000006110		Malignant infiltration of muscle
4036291000006113		Secondary malignant neoplasm of myocardium
4036301000006114		Metastatic malignant neoplasm to myocardium
4036311000006112		Secondary malignant neoplasm of myometrium
4036321000006116		Metastatic malignant neoplasm to myometrium
4036331000006118		Secondary malignant neoplasm of nasal bone
4036341000006111		Metastatic malignant neoplasm to nasal bone
4036351000006113		Secondary malignant neoplasm of nasal cavity
4036361000006110		Metastatic malignant neoplasm to nasal cavity
4036371000006115		Secondary malignant neoplasm of nasal concha
4036381000006117		Metastatic malignant neoplasm to nasal concha
4036391000006119		Secondary malignant neoplasm of navicular bone of foot
4036401000006117		Metastatic malignant neoplasm to navicular bone of foot
4036411000006119		Secondary malignant neoplasm of scaphoid bone
4036421000006110		Metastatic malignant neoplasm to scaphoid bone
4036431000006113		Secondary malignant neoplasm of navicular bone of hand
4036441000006115		Metastatic malignant neoplasm to navicular bone of hand

4036451 0000061 18		Secondary malignant neoplasm of neck
4036461 0000061 16		Metastatic malignant neoplasm to neck
4036471 0000061 11		Secondary malignant neoplasm of nervous system
4036481 0000061 14		Metastatic malignant neoplasm to nervous system
4036501 0000061 16		Cancer metastatic to nervous system
4036511 0000061 18		Secondary malignant neoplasm of nipple of female breast
4036521 0000061 14		Metastatic malignant neoplasm to nipple of female breast
4036531 0000061 12		Secondary malignant neoplasm of nipple of male breast
4036541 0000061 19		Metastatic malignant neoplasm to nipple of male breast
4036551 0000061 17		Secondary malignant neoplasm of nose
4036561 0000061 15		Metastatic malignant neoplasm to nose
4036571 0000061 10		Secondary malignant neoplasm of obturator lymph nodes
4036581 0000061 13		Metastatic malignant neoplasm to obturator lymph nodes
4036591 0000061 11		Cancer metastatic to obturator lymph nodes
4036601 0000061 15		Secondary malignant neoplasm of occipital bone
4036611 0000061 17		Metastatic malignant neoplasm to occipital bone
4036621 0000061 13		Secondary malignant neoplasm of occipital lobe
4036631 0000061 11		Metastatic malignant neoplasm to occipital lobe
4036641 0000061 18		Secondary malignant neoplasm of occipital lymph nodes

4036651 0000061 16		Metastatic malignant neoplasm to occipital lymph nodes
4036661 0000061 19		Secondary malignant neoplasm of oculomotor nerve
4036671 0000061 14		Metastatic malignant neoplasm to oculomotor nerve
4036681 0000061 12		Secondary malignant neoplasm of olfactory nerve
4036691 0000061 10		Metastatic malignant neoplasm to olfactory nerve
4036701 0000061 10		Secondary malignant neoplasm of optic nerve
4036711 0000061 13		Metastatic malignant neoplasm to optic nerve
4036730 17	B1.. .00	Malignant neoplasm of digestive organs and peritoneum
4036740 11	B10 z.00	Malignant neoplasm of oesophagus NOS
4036751 0000061 14		Secondary malignant neoplasm of orbit
4036761 0000061 11		Metastatic malignant neoplasm to orbit
4036801 0000061 19		Secondary malignant neoplasm of orbit proper
4036811 0000061 16		Secondary malignant neoplasm of oropharynx
4036820 11	B13 z.00	Malignant tumour of colon
4036821 0000061 12		Metastatic malignant neoplasm to oropharynx
4036841 0000061 17		Metastatic malignant neoplasm to ovary
4036880 10	B22 2.00	Malignant neoplasm of upper lobe, bronchus or lung
4036890 19	B22 z.00	Malignant tumour of lung
4036891 0000061 14		Secondary cancer of ovary
4036910 10	B30 ..00	Malignant neoplasm of bone and articular cartilage
4036921 0000061 15		Cancer metastatic to ovary

4036931 0000061 17		Secondary malignant neoplasm of palate
4036941 0000061 10		Metastatic malignant neoplasm to palate
4036951 0000061 12		Secondary malignant neoplasm of palatine bone
4036961 0000061 14		Metastatic malignant neoplasm to palatine bone
4036970 14	B30 5.00	Malignant neoplasm of hand bones
4036971 0000061 19		Secondary malignant neoplasm of tonsil
4036980 16	B30 8.00	Malignant neoplasm of short bone of lower limb
4036981 0000061 16		Metastatic malignant neoplasm to tonsil
4036991 0000061 18		Metastatic malignant neoplasm to palatine tonsil
4037000 13	B33 ..00	Malignant neoplasm of skin
4037001 0000061 14		Secondary malignant neoplasm of palatine tonsil
4037010 12	B45 4.00	Malignant tumour of vulva
4037011 0000061 12		Secondary malignant neoplasm of pancreas
4037020 17	B47 z.00	Malignant neoplasm of testis NOS
4037021 0000061 16		Metastatic malignant neoplasm to pancreas
4037040 16	B59 0.00	Disseminated malignancy
4037041 0000061 11		Secondary malignant deposit in pancreas
4037050 15	B6.. .00	Malignant neoplasm of lymphatic and haemopoietic tissue
4037061 0000061 10		Cancer metastatic to pancreas
4037071 0000061 15		Secondary malignant neoplasm of pancreatic duct
4037081 0000061 17		Metastatic malignant neoplasm to pancreatic duct
4037091 0000061 19		Secondary malignant neoplasm of para-aortic body

403710016	B62zz00	Lymphoid and histiocytic malignancy NOS
4037101000006113		Metastatic malignant neoplasm to para-aortic body
4037111000006111		Secondary malignant neoplasm of paraganglion
4037121000006115		Metastatic malignant neoplasm to paraganglion
4037131000006117		Secondary malignant neoplasm of paramammary lymph nodes
4037141000006110		Metastatic malignant neoplasm to paramammary lymph nodes
4037151000006112		Secondary malignant neoplasm of parametrial lymph nodes
4037161000006114		Metastatic malignant neoplasm to parametrial lymph nodes
4037171000006119		Secondary malignant neoplasm of parametrium
4037181000006116		Metastatic malignant neoplasm to parametrium
4037191000006118		Secondary malignant neoplasm of pararectal lymph nodes
4037201000006115		Metastatic malignant neoplasm to pararectal lymph nodes
4037211000006117		Cancer metastatic to pararectal lymph nodes
4037221000006113		Secondary malignant neoplasm of parathyroid gland
4037231000006111		Metastatic malignant neoplasm to parathyroid gland
4037241000006118		Secondary malignant neoplasm of paraurethral glands
4037251000006116		Metastatic malignant neoplasm to paraurethral glands
4037261000006119		Secondary malignant neoplasm of paravaginal lymph nodes
4037271000006114		Metastatic malignant neoplasm to paravaginal lymph nodes
4037281000006112		Secondary malignant neoplasm of parietal bone

4037291 0000061 10		Metastatic malignant neoplasm to parietal bone
4037301 0000061 11		Secondary malignant neoplasm of parietal lobe
4037311 0000061 14		Metastatic malignant neoplasm to parietal lobe
4037321 0000061 18		Secondary malignant neoplasm of parietal peritoneum
4037331 0000061 15		Metastatic malignant neoplasm to parietal peritoneum
4037341 0000061 13		Secondary malignant neoplasm of parietal pleura
4037350 10	B83 2.00	Carcinoma in situ of other and unspecified parts of uterus
4037351 0000061 10		Metastatic malignant neoplasm to parietal pleura
4037361 0000061 12		Secondary malignant neoplasm of parotid gland
4037371 0000061 17		Metastatic malignant neoplasm to parotid gland
4037381 0000061 19		Secondary malignant neoplasm of parotid lymph nodes
4037391 0000061 16		Metastatic malignant neoplasm to parotid lymph nodes
4037401 0000061 19		Secondary malignant neoplasm of patella
4037411 0000061 16		Metastatic malignant neoplasm to patella
4037421 0000061 12		Secondary malignant neoplasm of pectoral axillary lymph nodes
4037431 0000061 10		Metastatic malignant neoplasm to pectoral axillary lymph nodes
4037441 0000061 17		Secondary malignant neoplasm of pelvic bone
4037451 0000061 15		Metastatic malignant neoplasm to pelvic bone
4037461 0000061 18		Secondary malignant neoplasm of pelvic peritoneum
4037471 0000061 13		Metastatic malignant neoplasm to pelvic peritoneum

4037481 0000061 11		Secondary malignant neoplasm of pelvis
4037491 0000061 14		Metastatic malignant neoplasm to pelvis
4037511 0000061 15		Metastatic malignant neoplasm to penis
4037520 14	BB5 N.0 0	[M]Adenomatous and adenocarcinomatous polyps of colon
4037530 16	BB8 1.00	[M]Ovarian cystic, mucinous and serous neoplasms
4037540 10	BBE 1.00	Malignant melanoma
4037551 0000061 19		Secondary malignant neoplasm of periadrenal tissue
4037561 0000061 17		Metastatic malignant neoplasm to periadrenal tissue
4037571 0000061 12		Secondary malignant neoplasm of perianal skin
4037581 0000061 10		Metastatic malignant neoplasm to perianal skin
4037590 17	BBg 9.00	[M]Malignant lymphoma, mixed lymphocytic-histiocytic NOS
4037591 0000061 13		Secondary malignant neoplasm of pericardium
4037600 10	BBj 0.11	[M]Lymphogranuloma, malignant
4037601 0000061 17		Metastatic malignant neoplasm to pericardium
4037610 14	BBk 0.00	[M]Malignant lymphoma, nodular NOS
4037611 0000061 19		Secondary malignant neoplasm of perirenal tissue
4037620 19	Byu 500 0	Mesothelioma (malignant, clinical disorder)
4037621 0000061 10		Metastatic malignant neoplasm to perirenal tissue
4037631 0000061 13		Secondary malignant neoplasm of phalanx of foot
4037641 0000061 15		Metastatic malignant neoplasm to phalanx of foot
4037651 0000061 18		Secondary malignant neoplasm of phalanx of hand

4037661 0000061 16		Metastatic malignant neoplasm to phalanx of hand
4037671 0000061 11		Secondary malignant neoplasm of pharynx
4037681 0000061 14		Metastatic malignant neoplasm to pharynx
4037691 0000061 12		Secondary malignant neoplasm of pineal gland
4037701 0000061 12		Metastatic malignant neoplasm to pineal gland
4037710 11	B00 ..11	Carcinoma of lip
4037711 0000061 10		Secondary malignant neoplasm of pisiform bone of hand
4037721 0000061 19		Metastatic malignant neoplasm to pisiform bone of hand
4037731 0000061 16		Secondary malignant neoplasm of pituitary gland
4037741 0000061 14		Metastatic malignant neoplasm to pituitary gland
4037761 0000061 13		Cancer metastatic to pituitary gland
4037771 0000061 18		Secondary malignant neoplasm of placenta
4037781 0000061 15		Metastatic malignant neoplasm to placenta
4037801 0000061 16		Metastatic malignant neoplasm to pleura
4037811 0000061 18		Secondary malignant tumour of pleura
4037821 0000061 14		Secondary malignant tumor of pleura
4037851 0000061 17		Cancer metastatic to pleura
4037861 0000061 15		Secondary malignant neoplasm of popliteal lymph nodes
4037871 0000061 10		Metastatic malignant neoplasm to popliteal lymph nodes
4037881 0000061 13		Cancer metastatic to popliteal lymph nodes

4037891 0000061 11		Secondary malignant neoplasm of postcricoid region
4037901 0000061 10		Metastatic malignant neoplasm to postcricoid region
4037911 0000061 13		Secondary malignant neoplasm of posterior hypopharyngeal wall
4037921 0000061 17		Metastatic malignant neoplasm to posterior hypopharyngeal wall
4037931 0000061 19		Secondary malignant neoplasm of posterior mediastinum
4037941 0000061 12		Metastatic malignant neoplasm to posterior mediastinum
4037951 0000061 14		Secondary malignant neoplasm of posterior wall of nasopharynx
4037961 0000061 11		Metastatic malignant neoplasm to posterior wall of nasopharynx
4037971 0000061 16		Secondary malignant neoplasm of posterior wall of oropharynx
4037981 0000061 18		Metastatic malignant neoplasm to posterior wall of oropharynx
4037991 0000061 15		Secondary malignant neoplasm of posterior wall of urinary bladder
4038001 0000061 13		Metastatic malignant neoplasm to posterior wall of urinary bladder
4038011 0000061 11		Secondary malignant neoplasm of preauricular lymph nodes
4038021 0000061 15		Metastatic malignant neoplasm to preauricular lymph nodes
4038031 0000061 17		Secondary malignant neoplasm of presacral region
4038041 0000061 10		Metastatic malignant neoplasm to presacral region
4038061 0000061 14		Metastatic malignant neoplasm to prostate
4038101 0000061 12		Cancer metastatic to prostate
4038111 0000061 10		Secondary malignant neoplasm of pubis

4038121 0000061 19		Metastatic malignant neoplasm to pubis
4038131 0000061 16		Secondary malignant neoplasm of pyloric antrum
4038141 0000061 14		Metastatic malignant neoplasm to pyloric antrum
4038151 0000061 11		Secondary malignant neoplasm of pylorus
4038161 0000061 13		Metastatic malignant neoplasm to pylorus
4038171 0000061 18		Secondary malignant neoplasm of pyriform sinus
4038181 0000061 15		Metastatic malignant neoplasm to pyriform sinus
4038191 0000061 17		Secondary malignant neoplasm of radius
4038201 0000061 19		Metastatic malignant neoplasm to radius
4038211 0000061 16		Secondary malignant neoplasm of rectosigmoid junction
4038221 0000061 12		Metastatic malignant neoplasm to rectosigmoid junction
4038231 0000061 10		Cancer metastatic to rectosigmoid junction
4038241 0000061 17		Secondary malignant neoplasm of rectouterine pouch
4038251 0000061 15		Metastatic malignant neoplasm to rectouterine pouch
4038261 0000061 18		Secondary malignant neoplasm of rectovaginal septum
4038271 0000061 13		Metastatic malignant neoplasm to rectovaginal septum
4038281 0000061 11		Secondary malignant neoplasm of rectovesical septum
4038291 0000061 14		Metastatic malignant neoplasm to rectovesical septum
4038311 0000061 13		Metastatic malignant neoplasm to rectum

4038321000006117		Cancer metastatic to rectum
4038331000006119		Secondary malignant neoplasm of renal pelvis
4038341000006112		Metastatic malignant neoplasm to renal pelvis
4038371000006116		Metastatic malignant neoplasm to respiratory tract
4038381000006118		Cancer metastatic to respiratory tract
4038391000006115		Secondary malignant neoplasm of retina
4038401000006118		Metastatic malignant neoplasm to retina
4038411000006115		Secondary malignant neoplasm of retrocaecal tissue
4038421000006111		Secondary malignant neoplasm of retrocecal tissue
4038431000006114		Metastatic malignant neoplasm to retrocecal tissue
4038441000006116		Metastatic malignant neoplasm to retrocaecal tissue
4038451000006119		Secondary malignant neoplasm of retromolar area
4038461000006117		Metastatic malignant neoplasm to retromolar area
4038471000006112		Secondary malignant neoplasm of retroperitoneal lymph nodes
4038481000006110		Metastatic malignant neoplasm to retroperitoneal lymph nodes
4038491000006113		Cancer metastatic to retroperitoneal lymph nodes
4038501000006117		Secondary malignant neoplasm of retropharyngeal lymph nodes
4038511000006119		Metastatic malignant neoplasm to retropharyngeal lymph nodes
4038521000006110		Secondary malignant neoplasm of rib

4038531 0000061 13		Metastatic malignant neoplasm to rib
4038541 0000061 15		Secondary malignant neoplasm of right lower lobe of lung
4038551 0000061 18		Metastatic malignant neoplasm to right lower lobe of lung
4038561 0000061 16		Secondary malignant neoplasm of right middle lobe of lung
4038571 0000061 11		Metastatic malignant neoplasm to right middle lobe of lung
4038581 0000061 14		Secondary malignant neoplasm of right upper lobe of lung
4038591 0000061 12		Metastatic malignant neoplasm to right upper lobe of lung
4038601 0000061 16		Secondary malignant neoplasm of round ligament of uterus
4038611 0000061 18		Metastatic malignant neoplasm to round ligament of uterus
4038621 0000061 14		Secondary malignant neoplasm of sacrococcygeal region
4038631 0000061 12		Metastatic malignant neoplasm to sacrococcygeal region
4038641 0000061 19		Secondary malignant neoplasm of sacrum
4038651 0000061 17		Metastatic malignant neoplasm to sacrum
4038661 0000061 15		Secondary malignant neoplasm of scalene lymph nodes
4038671 0000061 10		Metastatic malignant neoplasm to scalene lymph nodes
4038681 0000061 13		Secondary malignant neoplasm of scapula
4038691 0000061 11		Metastatic malignant neoplasm to scapula
4038701 0000061 11		Secondary malignant neoplasm of sclera
4038711 0000061 14		Metastatic malignant neoplasm to sclera

4038721 0000061 18		Secondary malignant neoplasm of scrotum
4038731 0000061 15		Metastatic malignant neoplasm to scrotum
4038781 0000061 19		Secondary malignant neoplasm of sebaceous gland
4038791 0000061 16		Metastatic malignant neoplasm to sebaceous gland
4038801 0000061 15		Secondary malignant neoplasm of second cuneiform bone of foot
4038811 0000061 17		Metastatic malignant neoplasm to second cuneiform bone of foot
4038821 0000061 13		Secondary malignant neoplasm of septum of nose
4038831 0000061 11		Metastatic malignant neoplasm to septum of nose
4038841 0000061 18		Secondary malignant neoplasm of short bone of lower limb
4038851 0000061 16		Metastatic malignant neoplasm to short bone of lower limb
4038861 0000061 19		Secondary malignant neoplasm of short bone of upper limb
4038871 0000061 14		Metastatic malignant neoplasm to short bone of upper limb
4038881 0000061 12		Secondary malignant neoplasm of shoulder
4038891 0000061 10		Metastatic malignant neoplasm to shoulder
4038901 0000061 14		Secondary malignant neoplasm of sigmoid colon
4038911 0000061 12		Metastatic malignant neoplasm to sigmoid colon
4038921 0000061 16		Secondary malignant neoplasm of skin of abdomen
4038931 0000061 18		Metastatic malignant neoplasm to skin of abdomen
4038941 0000061 11		Secondary malignant neoplasm of skin of ankle

4038951 0000061 13		Metastatic malignant neoplasm to skin of ankle
4038961 0000061 10		Secondary malignant neoplasm of skin of axilla
4038971 0000061 15		Metastatic malignant neoplasm to skin of axilla
4038981 0000061 17		Secondary malignant neoplasm of skin of back
4038991 0000061 19		Metastatic malignant neoplasm to skin of back
4039011 0000061 16		Metastatic malignant neoplasm to skin of breast
4039021 0000061 12		Secondary malignant neoplasm of skin of buttock
4039031 0000061 10		Metastatic malignant neoplasm to skin of buttock
4039041 0000061 17		Secondary malignant neoplasm of skin of cheek
4039051 0000061 15		Metastatic malignant neoplasm to skin of cheek
4039061 0000061 18		Secondary malignant neoplasm of skin of chest
4039071 0000061 13		Metastatic malignant neoplasm to skin of chest
4039081 0000061 11		Secondary malignant neoplasm of skin of chin
4039091 0000061 14		Metastatic malignant neoplasm to skin of chin
4039101 0000061 15		Secondary malignant neoplasm of skin of ear
4039111 0000061 17		Metastatic malignant neoplasm to skin of ear
4039121 0000061 13		Secondary malignant neoplasm of skin of elbow
4039131 0000061 11		Metastatic malignant neoplasm to skin of elbow
4039141 0000061 18		Secondary malignant neoplasm of skin of external auditory canal

4039151000006116		Metastatic malignant neoplasm to skin of external auditory canal
4039161000006119		Secondary malignant neoplasm of skin of eyebrow
4039171000006114		Metastatic malignant neoplasm to skin of eyebrow
4039181000006112		Secondary malignant neoplasm of skin of eyelid
4039191000006110		Metastatic malignant neoplasm to skin of eyelid
4039211000006111		Metastatic malignant neoplasm to skin of face
4039221000006115		Secondary malignant neoplasm of skin of finger
4039231000006117		Metastatic malignant neoplasm to skin of finger
4039241000006110		Secondary malignant neoplasm of skin of foot
4039251000006112		Metastatic malignant neoplasm to skin of foot
4039261000006114		Secondary malignant neoplasm of skin of forearm
4039271000006119		Metastatic malignant neoplasm to skin of forearm
4039281000006116		Secondary malignant neoplasm of skin of forehead
4039291000006118		Metastatic malignant neoplasm to skin of forehead
4039301000006117		Secondary malignant neoplasm of skin of groin
4039311000006119		Metastatic malignant neoplasm to skin of groin
4039321000006110		Secondary malignant neoplasm of skin of hand
4039331000006113		Metastatic malignant neoplasm to skin of hand
4039341000006115		Secondary malignant neoplasm of skin of hip

4039351 0000061 18		Metastatic malignant neoplasm to skin of hip
4039361 0000061 16		Secondary malignant neoplasm of skin of knee
4039371 0000061 11		Metastatic malignant neoplasm to skin of knee
4039381 0000061 14		Secondary malignant neoplasm of skin of lip
4039391 0000061 12		Metastatic malignant neoplasm to skin of lip
4039401 0000061 14		Secondary malignant neoplasm of skin of lower limb
4039411 0000061 12		Metastatic malignant neoplasm to skin of lower limb
4039431 0000061 18		Metastatic malignant neoplasm to skin of neck
4039441 0000061 11		Secondary malignant neoplasm of skin of nose
4039451 0000061 13		Metastatic malignant neoplasm to skin of nose
4039461 0000061 10		Secondary malignant neoplasm of skin of perineum
4039471 0000061 15		Metastatic malignant neoplasm to skin of perineum
4039481 0000061 17		Secondary malignant neoplasm of skin of popliteal area
4039491 0000061 19		Metastatic malignant neoplasm to skin of popliteal area
4039501 0000061 10		Secondary malignant neoplasm of skin of scalp
4039511 0000061 13		Metastatic malignant neoplasm to skin of scalp
4039521 0000061 17		Secondary malignant neoplasm of skin of shoulder
4039531 0000061 19		Metastatic malignant neoplasm to skin of shoulder
4039541 0000061 12		Secondary malignant neoplasm of skin of temporal region

4039551000006114		Metastatic malignant neoplasm to skin of temporal region
4039561000006111		Secondary malignant neoplasm of skin of thigh
4039571000006116		Metastatic malignant neoplasm to skin of thigh
4039581000006118		Secondary malignant neoplasm of skin of toe
4039591000006115		Metastatic malignant neoplasm to skin of toe
4039611000006114		Metastatic malignant neoplasm to skin of trunk
4039621000006118		Secondary malignant neoplasm of skin of umbilicus
4039631000006115		Metastatic malignant neoplasm to skin of umbilicus
4039641000006113		Secondary malignant neoplasm of skin of upper limb
4039651000006110		Metastatic malignant neoplasm to skin of upper limb
4039661000006112		Secondary malignant neoplasm of skin of wrist
4039671000006117		Metastatic malignant neoplasm to skin of wrist
4039691000006116		Metastatic malignant neoplasm to skin
4039721000006114		Malignant infiltration of skin
4039741000006119		Secondary cancer of skin
4039761000006115		Cancer metastatic to skin
4039781000006113		Metastatic malignant neoplasm to small intestine
4039801000006112		Cancer metastatic to small intestine
4039811000006110		Secondary malignant neoplasm of soft palate

4039821000006119		Metastatic malignant neoplasm to soft palate
4039831000006116		Secondary malignant neoplasm of soft tissues of abdomen
4039841000006114		Metastatic malignant neoplasm to soft tissues of abdomen
4039851000006111		Secondary malignant neoplasm of soft tissues of axilla
4039861000006113		Metastatic malignant neoplasm to soft tissues of axilla
4039871000006118		Secondary malignant neoplasm of soft tissues of buttock
4039881000006115		Metastatic malignant neoplasm to soft tissues of buttock
4039891000006117		Secondary malignant neoplasm of soft tissues of face
4039901000006118		Metastatic malignant neoplasm to soft tissues of face
4039911000006115		Secondary malignant neoplasm of soft tissues of head
4039921000006111		Metastatic malignant neoplasm to soft tissues of head
4039931000006114		Secondary malignant neoplasm of soft tissues of hip
4039941000006116		Metastatic malignant neoplasm to soft tissues of hip
4039951000006119		Secondary malignant neoplasm of soft tissues of inguinal region
4039961000006117		Metastatic malignant neoplasm to soft tissues of inguinal region
4039971000006112		Secondary malignant neoplasm of soft tissues of lower limb
4039981000006110		Metastatic malignant neoplasm to soft tissues of lower limb
4039991000006113		Secondary malignant neoplasm of soft tissues of neck
4040001000006111		Metastatic malignant neoplasm to soft tissues of neck

4040011 0000061 14		Secondary malignant neoplasm of soft tissues of pelvis
4040021 0000061 18		Metastatic malignant neoplasm to soft tissues of pelvis
4040031 0000061 15		Cancer metastatic to soft tissues of pelvis
4040041 0000061 13		Secondary malignant neoplasm of soft tissues of perineum
4040051 0000061 10		Metastatic malignant neoplasm to soft tissues of perineum
4040061 0000061 12		Secondary malignant neoplasm of soft tissues of shoulder
4040071 0000061 17		Metastatic malignant neoplasm to soft tissues of shoulder
4040081 0000061 19		Secondary malignant neoplasm of soft tissues of thorax
4040091 0000061 16		Metastatic malignant neoplasm to soft tissues of thorax
4040101 0000061 10		Secondary malignant neoplasm of soft tissues of trunk
4040111 0000061 13		Metastatic malignant neoplasm to soft tissues of trunk
4040121 0000061 17		Secondary malignant neoplasm of soft tissues of upper limb
4040131 0000061 19		Metastatic malignant neoplasm to soft tissues of upper limb
4040141 0000061 12		Secondary malignant neoplasm of spermatic cord
4040151 0000061 14		Metastatic malignant neoplasm to spermatic cord
4040191 0000061 15		Secondary malignant neoplasm of sphenoid bone
4040201 0000061 17		Metastatic malignant neoplasm to sphenoid bone
4040211 0000061 19		Secondary malignant neoplasm of sphenoidal sinus
4040221 0000061 10		Metastatic malignant neoplasm to sphenoidal sinus

4040231 0000061 13		Secondary malignant neoplasm of spinal cord
4040241 0000061 15		Metastatic malignant neoplasm to spinal cord
4040261 0000061 16		Secondary malignant neoplasm of spinal meninges
4040271 0000061 11		Metastatic malignant neoplasm to spinal meninges
4040281 0000061 14		Cancer metastatic to spinal meninges
4040291 0000061 12		Secondary malignant neoplasm of vertebral column
4040301 0000061 13		Secondary malignant neoplasm of spine
4040311 0000061 11		Metastatic malignant neoplasm to spine
4040321 0000061 15		Metastatic malignant neoplasm to vertebral column
4040361 0000061 14		Secondary cancer of spine
4040371 0000061 19		CA - Secondary cancer of spine
4040401 0000061 16		Secondary malignant neoplasm of spleen
4040411 0000061 18		Metastatic malignant neoplasm to spleen
4040431 0000061 12		Secondary malignant deposit to spleen
4040441 0000061 19		Cancer metastatic to spleen
4040451 0000061 17		Secondary malignant neoplasm of splenic flexure of colon
4040461 0000061 15		Metastatic malignant neoplasm to splenic flexure of colon
4040471 0000061 10		Secondary malignant neoplasm of sternum
4040481 0000061 13		Metastatic malignant neoplasm to sternum

4040491000006111		Secondary malignant neoplasm of stomach
4040501000006115		Metastatic malignant neoplasm to stomach
4040511000006117		Cancer metastatic to stomach
4040521000006113		Secondary malignant neoplasm of subglottis
4040531000006111		Metastatic malignant neoplasm to subglottis
4040541000006118		Secondary malignant neoplasm of sublingual gland
4040551000006116		Metastatic malignant neoplasm to sublingual gland
4040561000006119		Secondary malignant neoplasm of submandibular lymph nodes
4040571000006114		Metastatic malignant neoplasm to submandibular lymph nodes
4040581000006112		Secondary malignant neoplasm of submaxillary gland
4040591000006110		Metastatic malignant neoplasm to submaxillary gland
4040601000006119		Secondary malignant neoplasm of submental lymph nodes
4040611000006116		Metastatic malignant neoplasm to submental lymph nodes
4040621000006112		Secondary malignant neoplasm of superficial inguinal lymph nodes
4040631000006110		Metastatic malignant neoplasm to superficial inguinal lymph nodes
4040641000006117		Secondary malignant neoplasm of superior wall of nasopharynx
4040651000006115		Metastatic malignant neoplasm to superior wall of nasopharynx
4040661000006118		Secondary malignant neoplasm of supraclavicular lymph nodes
4040671000006113		Metastatic malignant neoplasm to supraclavicular lymph nodes

4040681 0000061 11		Secondary malignant neoplasm of supraclavicular region
4040691 0000061 14		Metastatic malignant neoplasm to supraclavicular region
4040701 0000061 14		Secondary malignant neoplasm of supraglottis
4040711 0000061 12		Metastatic malignant neoplasm to supraglottis
4040721 0000061 16		Secondary malignant neoplasm of sweat gland
4040731 0000061 18		Metastatic malignant neoplasm to sweat gland
4040741 0000061 11		Secondary malignant neoplasm of tail of pancreas
4040751 0000061 13		Metastatic malignant neoplasm to tail of pancreas
4040761 0000061 10		Secondary malignant neoplasm of talus
4040771 0000061 15		Metastatic malignant neoplasm to talus
4040781 0000061 17		Secondary malignant neoplasm of tarsal bone
4040791 0000061 19		Metastatic malignant neoplasm to tarsal bone
4040801 0000061 18		Secondary malignant neoplasm of temporal bone
4040811 0000061 15		Metastatic malignant neoplasm to temporal bone
4040821 0000061 11		Secondary malignant neoplasm of temporal lobe
4040831 0000061 14		Metastatic malignant neoplasm to temporal lobe
4040851 0000061 19		Metastatic malignant neoplasm to testis
4040891 0000061 13		Secondary malignant neoplasm of the mesentery
4040901 0000061 12		Metastatic malignant neoplasm to the mesentery

4040911000006110		Secondary malignant neoplasm of the mesocolon
4040921000006119		Metastatic malignant neoplasm to the mesocolon
4040931000006116		Secondary malignant neoplasm of omentum
4040971000006118		Cancer metastatic to omentum
4040981000006115		Metastatic malignant neoplasm to omentum
4041021000006114		Secondary malignant peritoneal deposit
4041031000006112		Cancer metastatic to peritoneum
4041041000006119		Metastatic malignant neoplasm to peritoneum
4041061000006115		Secondary malignant neoplasm of the retroperitoneum
4041071000006110		Metastatic malignant neoplasm to the retroperitoneum
4041101000006117		Secondary malignant neoplasm of thigh
4041111000006119		Metastatic malignant neoplasm to thigh
4041121000006110		Secondary malignant neoplasm of third cuneiform bone of foot
4041131000006113		Metastatic malignant neoplasm to third cuneiform bone of foot
4041141000006115		Secondary malignant neoplasm of thoracic oesophagus
4041151000006118		Secondary malignant neoplasm of thoracic esophagus
4041161000006116		Metastatic malignant neoplasm to thoracic esophagus
4041171000006111		Metastatic malignant neoplasm to thoracic oesophagus
4041181000006114		Secondary malignant neoplasm of thymus

4041191<br>0000061<br>12		Metastatic malignant neoplasm to thymus
4041211<br>0000061<br>13		Secondary malignant tumour of thymus
4041221<br>0000061<br>17		Secondary malignant tumor of thymus
4041231<br>0000061<br>19		Secondary malignant neoplasm of thyroglossal duct
4041241<br>0000061<br>12		Metastatic malignant neoplasm to thyroglossal duct
4041251<br>0000061<br>14		Secondary malignant neoplasm of thyroid gland
4041261<br>0000061<br>11		Metastatic malignant neoplasm to thyroid gland
4041281<br>0000061<br>18		Cancer metastatic to thyroid
4041291<br>0000061<br>15		Secondary malignant neoplasm of tibia
4041301<br>0000061<br>19		Metastatic malignant neoplasm to tibia
4041311<br>0000061<br>16		Secondary malignant neoplasm of tibial lymph nodes
4041321<br>0000061<br>12		Metastatic malignant neoplasm to tibial lymph nodes
4041331<br>0000061<br>10		Cancer metastatic to tibial lymph nodes
4041341<br>0000061<br>17		Secondary malignant neoplasm of tip and lateral border of tongue
4041351<br>0000061<br>15		Metastatic malignant neoplasm to tip and lateral border of tongue
4041371<br>0000061<br>13		Metastatic malignant neoplasm to tongue
4041381<br>0000061<br>11		Secondary malignant neoplasm of tonsillar fossa
4041391<br>0000061<br>14		Metastatic malignant neoplasm to tonsillar fossa
4041401<br>0000061<br>11		Secondary malignant neoplasm of tonsillar pillar

4041411 0000061 14		Metastatic malignant neoplasm to tonsillar pillar
4041421 0000061 18		Secondary malignant neoplasm of trachea
4041431 0000061 15		Metastatic malignant neoplasm to trachea
4041441 0000061 13		Secondary malignant tumour of trachea
4041451 0000061 10		Secondary malignant tumor of trachea
4041471 0000061 17		Cancer metastatic to trachea
4041481 0000061 19		Secondary malignant neoplasm of tracheobronchial lymph nodes
4041491 0000061 16		Metastatic malignant neoplasm to tracheobronchial lymph nodes
4041501 0000061 12		Cancer metastatic to tracheobronchial lymph nodes
4041511 0000061 10		Secondary malignant neoplasm of transverse colon
4041521 0000061 19		Metastatic malignant neoplasm to transverse colon
4041531 0000061 16		Secondary malignant neoplasm of trapezium
4041541 0000061 14		Metastatic malignant neoplasm to trapezium
4041551 0000061 11		Secondary malignant neoplasm of trapezial bone
4041561 0000061 13		Metastatic malignant neoplasm to trapezial bone
4041571 0000061 18		Secondary malignant neoplasm of trapezoid bone
4041581 0000061 15		Metastatic malignant neoplasm to trapezoid bone
4041591 0000061 17		Secondary malignant neoplasm of trigeminal nerve
4041601 0000061 13		Metastatic malignant neoplasm to trigeminal nerve

4041611000006111		Secondary malignant neoplasm of trigone of urinary bladder
4041621000006115		Metastatic malignant neoplasm to trigone of urinary bladder
4041631000006117		Secondary malignant neoplasm of trochlear nerve
4041641000006110		Metastatic malignant neoplasm to trochlear nerve
4041651000006112		Secondary malignant neoplasm of trunk
4041661000006114		Metastatic malignant neoplasm to trunk
4041671000006119		Secondary malignant neoplasm of ulna
4041681000006116		Metastatic malignant neoplasm to ulna
4041691000006118		Secondary malignant neoplasm of undescended testis
4041701000006118		Metastatic malignant neoplasm to undescended testis
4041711000006115		Secondary malignant neoplasm of upper gum
4041721000006111		Metastatic malignant neoplasm to upper gum
4041731000006114		Secondary malignant neoplasm of upper inner quadrant of female breast
4041741000006116		Metastatic malignant neoplasm to upper inner quadrant of female breast
4041751000006119		Secondary malignant neoplasm of upper limb
4041761000006117		Metastatic malignant neoplasm to upper limb
4041771000006112		Secondary malignant neoplasm of upper outer quadrant of female breast
4041781000006110		Metastatic malignant neoplasm to upper outer quadrant of female breast
4041791000006113		Secondary malignant neoplasm of upper respiratory tract

4041801 0000061 14		Metastatic malignant neoplasm to upper respiratory tract
4041811 0000061 12		Secondary malignant neoplasm of upper third of oesophagus
4041821 0000061 16		Metastatic malignant neoplasm to upper third of esophagus
4041831 0000061 18		Secondary malignant neoplasm of upper third of esophagus
4041841 0000061 11		Metastatic malignant neoplasm to upper third of oesophagus
4041851 0000061 13		Secondary malignant neoplasm of urachus
4041861 0000061 10		Metastatic malignant neoplasm to urachus
4041881 0000061 17		Metastatic malignant neoplasm to ureter
4041921 0000061 13		Secondary malignant neoplasm of ureteric orifice of urinary bladder
4041931 0000061 11		Metastatic malignant neoplasm to ureteric orifice of urinary bladder
4041951 0000061 16		Metastatic malignant neoplasm to urethra
4041991 0000061 10		Secondary malignant neoplasm of urinary bladder neck
4042001 0000061 12		Metastatic malignant neoplasm to urinary bladder neck
4042021 0000061 19		Metastatic malignant neoplasm to urinary system
4042041 0000061 14		Cancer metastatic to urinary tract
4042051 0000061 11		Secondary malignant neoplasm of uterine adnexa
4042061 0000061 13		Metastatic malignant neoplasm to uterine adnexa
4042081 0000061 15		Metastatic malignant neoplasm to uterus
4042101 0000061 11		Cancer metastatic to uterus

4042111000006114		Secondary malignant neoplasm of uveal tract
4042121000006118		Metastatic malignant neoplasm to uveal tract
4042131000006115		Secondary malignant neoplasm of uvula
4042141000006113		Metastatic malignant neoplasm to uvula
4042161000006112		Metastatic malignant neoplasm to vagina
4042201000006118		Cancer metastatic to vagina
4042211000006115		Secondary malignant neoplasm of vagus nerve
4042221000006111		Metastatic malignant neoplasm to vagus nerve
4042231000006114		Secondary malignant neoplasm of vallecula
4042241000006116		Metastatic malignant neoplasm to vallecula
4042251000006119		Secondary malignant neoplasm of vas deferens
4042261000006117		Metastatic malignant neoplasm to vas deferens
4042271000006112		Secondary malignant neoplasm of ventral surface of tongue
4042281000006110		Metastatic malignant neoplasm to ventral surface of tongue
4042291000006113		Secondary malignant neoplasm of vermilion border of lip
4042301000006114		Metastatic malignant neoplasm to vermilion border of lip
4042311000006112		Secondary malignant neoplasm of vermilion border of lower lip
4042321000006116		Metastatic malignant neoplasm to vermilion border of lower lip
4042331000006118		Secondary malignant neoplasm of vermilion border of upper lip

4042341000006111		Metastatic malignant neoplasm to vermilion border of upper lip
4042351000006113		Secondary malignant neoplasm of vestibule of mouth
4042361000006110		Metastatic malignant neoplasm to vestibule of mouth
4042371000006115		Secondary malignant neoplasm of vestibule of nose
4042381000006117		Metastatic malignant neoplasm to vestibule of nose
4042391000006119		Secondary malignant neoplasm of visceral pleura
4042401000006117		Metastatic malignant neoplasm to visceral pleura
4042411000006119		Secondary malignant neoplasm of vocal cord
4042421000006110		Metastatic malignant neoplasm to vocal cord
4042431000006113		Secondary malignant neoplasm of vomer
4042441000006115		Metastatic malignant neoplasm to vomer
4042461000006116		Metastatic malignant neoplasm to vulva
4042481000006114		Cancer metastatic to vulva
4042491000006112		Secondary malignant neoplasm of Waldeyer's ring
4042501000006116		Metastatic malignant neoplasm to Waldeyer's ring
4042511000006118		Secondary malignant neoplasm of Waldeyer ring
4042521000006114		Secondary malignant neoplasm of zygomatic bone
4042531000006112		Metastatic malignant neoplasm to zygomatic bone
40480100000006116	Byu4200	[X]Oth malignant neoplasm/skin of oth+unspecfd parts of face
406052010	B0...00	Malignant neoplasm of lip, oral cavity and pharynx

406054011	B800.00	Carcinoma in situ of lip, oral cavity and pharynx
406154013	ByuDF00	Non-Hodgkin's lymphoma (clinical)
406263015	B4...00	Malignant neoplasm of genitourinary organ
406265010	B5...00	Malignant neoplastic disease
406266011	B58y.00	Secondary malignant neoplasm of other specified sites
406281017	BB2..00	[M]Papillary and squamous cell neoplasms
406332017	B808.00	Carcinoma in situ of liver and biliary system
406333010	B56..00	Secondary malignant neoplasm of lymph node
406399011	B003.00	Malignant neoplasm of lower lip, inner aspect
40641000006114	B30..12	Osteoma
408556100000006117		Mucin-like carcinoma-associated antigen
409430100000006111		Atypical squamous cells of uncertain significance, probably malignant
409459100000006113		Atypical mesenchymal cells of uncertain significance, probably malignant
409464100000006115		Atypical smooth muscle cells of uncertain significance, probably malignant
409469100000006112		Atypical striated muscle cells of uncertain significance, probably malignant
409551100000006116		Meibomian adenocarcinoma
409844012	B509.00	Malignant melanoma of eye
409845013	B594.00	Secondary malignant neoplasm of unknown site
411399019	B454.11	Primary vulval cancer
411872014	142..12	H/O: carcinoma
411873016	142..15	H/O: neoplasm
412853100000006117		Gleason grading system for prostatic cancer
412854100000006110		Gleason grading for prostatic cancer
412855100000006112		Gleason's microscopic prostatic carcinoma grading system

4128581 0000061 16		FIGO staging system for endometrial carcinoma
4128591 0000061 18		FIGO staging of endometrial carcinoma
4128601 0000061 14		International Federation of Gynaecology and Obstetrics (FIGO) staging system for endometrial carcinoma
4128611 0000061 12		International Federation of Gynecology and Obstetrics staging system for endometrial carcinoma
4128621 0000061 16		FIGO staging system for vulvar carcinoma
4128631 0000061 18		FIGO staging of vulvar carcinoma
4128671 0000061 15		International Federation of Gynaecology and Obstetrics (FIGO) staging system for vulvar carcinoma
4128681 0000061 17		International Federation of Gynecology and Obstetrics (FIGO) staging system for vulvar carcinoma
4128691 0000061 19		International Federation of Gynecology and Obstetrics staging system for vulvar carcinoma
4128801 0000061 10		TNM classification of malignant tumour before any treatment
4128811 0000061 13		TNM classification of malignant tumor before any treatment
4128851 0000061 14		Tumor-node-metastasis (TNM) classification of malignant tumor before any treatment
4128861 0000061 11		Tumor-node-metastasis (TNM) classification of malignant tumour before any treatment
4128871 0000061 16		TNM classification of malignant tumour after operation
4128881 0000061 18		TNM classification of malignant tumor after operation
4128921 0000061 14		Tumor-node-metastasis (TNM) classification of malignant tumour after operation
4128931 0000061 12		Tumor-node-metastasis (TNM) classification of malignant tumor after operation
4128941 0000061 19		FIGO staging system for cervical carcinoma
4128951 0000061 17		FIGO staging of cervical carcinoma

4128991 0000061 11		International Federation of Gynaecology and Obstetrics (FIGO) staging system for cervical carcinoma
4129001 0000061 11		International Federation of Gynecology and Obstetrics staging system for cervical carcinoma
4140080 12	B64 100 0	B-cell chronic lymphocytic leukaemia
4141600 18	B64 000 0	B-cell acute lymphoblastic leukaemia
4142520 13	B62 790 0	Mucosa-associated lymphoma
4142570 19	B62 700 0	Follicular non-Hodgkin's small cleaved cell lymphoma
4142780 16	B62 720 0	Follicular non-Hodgkin's large cell lymphoma
4161771 0000061 10		Overlapping malignant neoplasm of skin
4161811 0000061 10		In situ malignant melanoma of skin
4161821 0000061 19		ISMM - In situ malignant melanoma of skin
4162981 0000061 19		Overlapping malignant neoplasm of bone and articular cartilage
4162991 0000061 16		Overlapping malignant neoplasm of bone and articular cartilage of limbs
4163011 0000061 17		Overlapping malignant neoplasm of soft tissues
4163021 0000061 13		Overlapping malignant neoplasm of connective and other soft tissues
4163071 0000061 14		Cancer in situ
4163081 0000061 12		Primary malignant neoplasm of unspecified site (clinical)
4163091 0000061 10		Primary malignant neoplasm of unspecified site
4163101 0000061 16		Primary malignant neoplasm of ill-defined site
4163111 0000061 18		Overlapping malignant neoplasm of ill-defined site

4163121 0000061 14		Primary malignant neoplasm of independent multiple sites
4163191 0000061 11		Overlapping malignant neoplasm of accessory sinuses
4163201 0000061 14		Overlapping malignant neoplasm of nasopharynx
4163211 0000061 12		Overlapping malignant neoplasm of nasopharyngeal wall
4163231 0000061 18		Overlapping malignant neoplasm of hypopharynx
4163251 0000061 13		Overlapping malignant neoplasm of larynx
4163271 0000061 15		Primary malignant neoplasm of laryngeal cartilage
4163281 0000061 17		Overlapping malignant neoplasm of bronchus and lung
4163321 0000061 11		Overlapping malignant neoplasm of mediastinum and pleura
4163371 0000061 12		Malignant mesothelioma
4163431 0000061 19		Malignant mesothelioma of pericardium
4163451 0000061 14		Overlapping malignant neoplasm of heart, mediastinum and pleura
4170251 0000061 14		Overlapping malignant neoplasm of gastrointestinal tract
4170261 0000061 11		Overlapping malignant neoplasm of digestive tract
4170271 0000061 16		Overlapping malignant neoplasm of lip
4170281 0000061 18		Overlapping malignant neoplasm of tongue
4170301 0000061 19		Overlapping malignant neoplasm of major salivary gland
4170321 0000061 12		Cancer major salivary glands contiguous
4170361 0000061 18		Carcinoma in situ of salivary gland duct

4170371000006113		Carcinoma in situ of salivary duct
4170381000006111		Cancer in situ of salivary gland duct
4170391000006114		Primary malignant neoplasm of salivary gland duct
4170401000006111		Overlapping malignant neoplasm of floor of mouth
4170421000006118		Cancer of the floor of mouth, overlapping sites
4170431000006115		Overlapping malignant neoplasm of palate
4170441000006113		Cancer of the palate, overlapping sites
4170451000006110		Overlapping malignant neoplasm of oropharynx
4170461000006112		Overlapping malignant neoplasm of lip, oral cavity and pharynx
4170471000006117		Overlapping malignant neoplasm of lip, oral cavity and/or pharynx
4170481000006119		Primary malignant neoplasm of branchial cleft
4170491000006116		Overlapping malignant neoplasm of oesophagus
4170501000006112		Overlapping malignant neoplasm of esophagus
4170521000006119		Malignant neoplasm, overlapping lesion of esophagus
4170531000006116		Overlapping malignant neoplasm of stomach
4170551000006111		Cancer of the stomach, overlapping sites
4170561000006113		Overlapping malignant neoplasm of small intestine
4170581000006115		Cancer of the small intestine, overlapping sites
4170591000006117		Overlapping malignant neoplasm of colon

4170601 0000061 13		Cancer of the colon, overlapping sites
4170611 0000061 11		Overlapping malignant neoplasm of rectum, anus and anal canal
4170621 0000061 15		Primary malignant neoplasm of cloacogenic zone
4170641 0000061 10		Hepatocarcinoma
4170661 0000061 14		Malignant hepatoma
4170671 0000061 19		HCC - Hepatocellular carcinoma
4170681 0000061 16		LCC - Liver cell carcinoma
4170691 0000061 18		Liver carcinoma
4170701 0000061 18		Intrahepatic bile duct carcinoma
4170751 0000061 19		Overlapping malignant neoplasm of biliary tract
4170771 0000061 12		Overlapping malignant neoplasm of pancreas
4170791 0000061 13		Cancer of the pancreas, overlapping sites
4170811 0000061 12		Overlapping malignant neoplasm of retroperitoneum and peritoneum
4170841 0000061 11		Malignant mesothelioma of peritoneum
4170851 0000061 13		Cancer of the peritoneum, mesothelioma
4170871 0000061 15		Malignant mesothelioma of parietal peritoneum
4170891 0000061 19		Malignant mesothelioma of pelvic peritoneum
4170911 0000061 17		Malignant mesothelioma of mesentery
4170941 0000061 18		Malignant mesothelioma of omentum

4171111 0000061 13		Overlapping malignant neoplasm of urinary system
4171121 0000061 17		Overlapping malignant neoplasm of male genital organs
4171141 0000061 12		Overlapping malignant neoplasm of penis
4171161 0000061 11		Cancer of penis, overlapping sites
4171191 0000061 15		Overlapping malignant neoplasm of female genital organs
4171201 0000061 17		Overlapping malignant neoplasm of body of uterus
4171211 0000061 19		Overlapping malignant neoplasm of corpus uteri
4171221 0000061 10		Overlapping malignant neoplasm of uterine cervix
4171231 0000061 13		Overlapping malignant neoplasm of cervix uteri
4171261 0000061 16		Primary malignant neoplasm of fundus uteri
4171281 0000061 14		Overlapping malignant neoplasm of female breast
4171291 0000061 12		Overlapping malignant neoplasm of male breast
4171321 0000061 15		Carcinoma in situ of other site of breast
4171631 0000061 10		Cancer of the brain, overlapping sites
4171691 0000061 14		Primary malignant neoplasm of meninges
4171741 0000061 11		Primary malignant neoplasm of peripheral nerves and peripheral autonomic nervous system
4171751 0000061 13		Overlapping malignant neoplasm of peripheral nerves and autonomic nervous system
4171781 0000061 17		Primary malignant neoplasm of peripheral nerve of head, face AND/OR neck
4171791 0000061 19		Primary malignant neoplasm of peripheral nerve of head, face and/or neck

4171811 0000061 15		Primary malignant neoplasm of peripheral nerves of head
4171831 0000061 14		Primary malignant neoplasm of peripheral nerves of face
4171851 0000061 19		Primary malignant neoplasm of peripheral nerves of neck
4171871 0000061 12		Primary malignant neoplasm of peripheral nerves of upper limb
4171891 0000061 13		Primary malignant neoplasm of peripheral nerves of shoulder
4171911 0000061 10		Primary malignant neoplasm of peripheral nerves of lower limb
4171931 0000061 16		Primary malignant neoplasm of peripheral nerves of hip
4171951 0000061 11		Primary malignant neoplasm of peripheral nerves of thorax
4171971 0000061 18		Primary malignant neoplasm of peripheral nerves of abdomen
4172001 0000061 15		Primary malignant neoplasm of peripheral nerves of pelvis
4172011 0000061 17		Primary malignant neoplasm of peripheral nerves of pelvis region
4172031 0000061 11		Primary malignant neoplasm of peripheral nerves of trunk
4172041 0000061 18		Overlapping malignant neoplasm of eye and adnexa (primary)
4172061 0000061 19		Overlapping malignant neoplasm of eye and adnexa, primary
4172071 0000061 14		Cancer of the eye and adnexa, overlapping sites
4172091 0000061 10		Overlapping malignant neoplasm of multiple endocrine glands
4172261 0000061 10		Malignant lymphoma - lymphoblastic
4172321 0000061 11		Malignant lymphoma - immunoblastic
4172341 0000061 16		Immunoblastic malignant lymphoma - large cell

4172451000006114		Diffuse malignant lymphoma - histiocytic
4172461000006111		Diffuse malignant lymphoma - large cell
4172471000006116		Malignant lymphoma - large cell cleaved and non-cleaved
4172751000006118		Malignant immunoproliferative disease (clinical)
4172761000006116		Malignant immunoproliferative disease
4173811000006119		Overlapping malignant neoplasm of palatine tonsil
4179961000006114		Malignant odontogenic ghost cell tumour
4179971000006119		Malignant odontogenic ghost cell tumor
4179981000006116		Malignant lymphoma, metastatic
4189831000006118		Diffuse palmoplantar keratoderma with esophageal cancer
4189851000006113		Keratoderma with carcinoma of oesophagus
4189861000006110		Diffuse palmoplantar keratoderma with oesophageal cancer
4189871000006115		Keratoderma with carcinoma of esophagus
420391000006117	ZVu6H00	[X]Personal history/malignant neoplasms/other organs+systems
420471000006114	ZVu6F00	[X]Personal history of malignant neoplasm of other respiratory and intrathoracic organs
420481000006112	ZVu6G00	History of malignant haematologic neoplasm
4212921000006117		Pseudocarcinomatous hyperplasia
4213071000006113		Transitional cell carcinoma, spindle cell
4213081000006111		Transitional cell carcinoma, sarcomatoid

4213091 0000061 14		Transitional cell carcinoma - spindle cell
4213101 0000061 15		Bronchiolo-alveolar adenocarcinoma
4213111 0000061 17		Bronchiolo-alveolar carcinoma
4213121 0000061 13		Alveolar cell carcinoma
4213131 0000061 11		Bronchiolar carcinoma
4213141 0000061 18		Bronchiolar adenocarcinoma
4213381 0000061 14		Malignant mixed mesodermal tumor
4213391 0000061 12		Malignant mixed mesodermal tumour
4251100 0033112		Oestrogens In Malignant Diseases
4255441 0000061 18		Adnexal AND/OR skin appendage neoplasm
4255631 0000061 14		Adipocytic neoplasm
4255691 0000061 13		Complex mixed AND/OR stromal neoplasm
4255791 0000061 18		Osseous AND/OR chondromatous neoplasm
4255921 0000061 18		Malignant lymphoma - category
4257710 0000611 3	Byu C30 0	Secondary malignant neoplasm of respiratory tract
4257810 0000611 1	Byu C40 0	Secondary malignant neoplasm of gastrointestinal tract
4267431 0000061 12		Solid pseudopapillary carcinoma
4309281 0000061 10		Malignant Hodgkin's lymphoma
4309371 0000061 15		Malignant lymphoma, non-Hodgkin's type
4309701 0000061 10		Malignant histiocytosis (clinical)

430989100000006114		Malignant mast cell tumour (clinical)
430990100000006113		Malignant mast cell tumor (clinical)
430992100000006115		Malignant mast cell tumour
430993100000006117		Malignant mast cell tumor
431001100000006116		Burkitt's type malignant lymphoma - undifferentiated
431003100000006110		Burkitt's type malignant lymphoma - small non-cleaved
435660100000006115		Endocervical adenocarcinoma
435661100000006117		Adenocarcinoma of endocervix
435668100000006112		Adenocarcinoma of endometrium
435669100000006110		Endometrial adenocarcinoma
439326100000006117		Chronic cold agglutinin disease associated with B-cell neoplasm
439356100000006115		Malignant histiocytic disorder
439512100000006117		Malignant lymphoma of lymph nodes
439676100000006117		Foetal neoplasm
440024100000006116		Neuroendocrine carcinoma, grade 1
440025100000006119		Well-differentiated neuroendocrine carcinoma
440026100000006117		Grade 1 neuroendocrine carcinoma
440027100000006112		Neuroendocrine carcinoma, grade 2
440028100000006110		Moderately differentiated neuroendocrine carcinoma

4400291 0000061 13		Grade 2 neuroendocrine carcinoma
4400301 0000061 14		Neuroendocrine carcinoma, grade 3
4400311 0000061 12		Malignant neuroendocrine neoplasm, epithelial
4400321 0000061 16		Malignant neuroendocrine neoplasm, neural
4409441 0000061 13		Metastatic neoplasm (disease)
4409451 0000061 10		CA - Secondary cancer
4409471 0000061 17		Metastatic neoplasm
4409491 0000061 16		Secondary cancer
4409501 0000061 12		Metastatic malignant disease
4409531 0000061 16		Secondary malignant deposit
4409561 0000061 13		Metastatic cancer
4409591 0000061 17		Secondary malignant neoplasm of articular cartilage
4409601 0000061 13		Primary malignant neoplasm of articular cartilage
4412011 0000061 13		Trichilemmocarcinoma
4412021 0000061 17		Trichilemmal carcinoma
4412031 0000061 19		Papillary transitional cell neoplasm of low malignant potential
4412041 0000061 12		Papillary urothelial neoplasm of low malignant potential
4412071 0000061 16		Malignant tumour, clear cell type
4412081 0000061 18		Malignant tumor, clear cell type

4412091000006115		Large cell neuroendocrine carcinoma
4412101000006114		Large cell carcinoma with rhabdoid phenotype
4412111000006112		Glassy cell carcinoma
4412121000006116		Carcinoma with osteoclast-like giant cells
4412141000006111		Squamous cell carcinoma with horn formation
4412151000006113		Basaloid squamous cell carcinoma
4412161000006110		Squamous cell carcinoma, clear cell type
4412181000006117		Adenoid basal carcinoma
4412251000006117		Transitional cell carcinoma, micropapillary
4412331000006116		Somatostatinoma, malignant
4412341000006114		Somatostatin cell tumor, malignant
4412351000006111		Somatostatin cell tumour, malignant
4412371000006118		Hepatocellular carcinoma, scirrhous
4412381000006115		Sclerosing hepatic carcinoma
4412391000006117		Papillary squamous cell carcinoma, non-invasive
4412401000006115		Papillary squamous cell carcinoma in situ
4412411000006117		Hepatocellular carcinoma, spindle cell variant
4412421000006113		Hepatocellular carcinoma, sarcomatoid
4412431000006111		Hepatocellular carcinoma, clear cell type

4412441000006118		Hepatocellular carcinoma, pleomorphic type
4412491000006110		Parietal cell carcinoma
4412501000006119		Parietal cell adenocarcinoma
4412511000006116		Adenocarcinoma of anal glands
4412521000006112		Adenocarcinoma of anal ducts
4412551000006115		Enterochromaffin-like cell tumour, malignant
4412561000006118		ECL cell carcinoid, malignant
4412571000006113		Enterochromaffin-like cell tumor, malignant
4412601000006118		Bronchiolo-alveolar carcinoma, non-mucinous
4412611000006115		Bronchiolo-alveolar carcinoma, type II pneumocyte
4412621000006111		Bronchiolo-alveolar carcinoma, Clara cell
4412631000006114		Bronchiolo-alveolar carcinoma, mucinous
4412641000006116		Bronchiolo-alveolar carcinoma, goblet cell type
4412651000006119		Bronchiolo-alveolar carcinoma, mixed mucinous and non-mucinous
4412661000006117		Bronchiolo-alveolar carcinoma, type II pneumocyte and goblet cell type
4412671000006112		Bronchiolo-alveolar carcinoma, indeterminate type
4412681000006110		Bronchiolo-alveolar carcinoma, Clara cell and goblet cell type
4412691000006113		Adenocarcinoma with mixed subtypes
4412701000006113		Adenocarcinoma combined with other types of carcinoma

4412761000006114		Pituitary carcinoma
4412771000006119		Cyst-associated renal cell carcinoma
4412781000006116		Renal cell carcinoma, chromophobe cell
4412791000006118		Chromophobe cell renal carcinoma
4412801000006117		Renal cell carcinoma, sarcomatoid
4412811000006119		Renal cell carcinoma, spindle cell
4412821000006110		Collecting duct carcinoma
4412831000006113		Renal carcinoma, collecting duct type
4412841000006115		Bellini duct carcinoma
4412861000006116		Follicular carcinoma, minimally invasive
4412871000006111		Follicular carcinoma, encapsulated
4412901000006111		Insular carcinoma
4412911000006114		Papillary microcarcinoma
4412921000006118		Papillary carcinoma, oxyphilic cell
4412931000006115		Papillary carcinoma, encapsulated
4412941000006113		Papillary carcinoma, columnar cell
4412951000006110		Papillary carcinoma, tall cell
4412961000006112		Mixed medullary-follicular carcinoma
4412971000006117		Mixed medullary-papillary carcinoma

4412981000006119		Endometrioid adenocarcinoma, secretory variant
4412991000006116		Endometrioid adenocarcinoma, ciliated cell variant
4413001000006115		Adenocarcinoma, endocervical type
4413061000006119		Eccrine poroma, malignant
4413071000006114		Porocarcinoma
4413081000006112		Eccrine adenocarcinoma
4413151000006117		Intraductal papillary-mucinous carcinoma, non-invasive
4413161000006115		Intraductal papillary-mucinous carcinoma, invasive
4413211000006112		Mucinous adenocarcinoma, endocervical type
4413221000006116		Intraductal micropapillary carcinoma
4413231000006118		Ductal carcinoma in situ, micropapillary
4413241000006111		Intraductal carcinoma, clinging, high grade
4413251000006113		Cystic hypersecretory carcinoma
4413261000006110		Atypical medullary carcinoma
4413271000006115		Duct carcinoma, desmoplastic type
4413281000006117		Infiltrating duct mixed with other types of carcinoma
4413291000006119		Infiltrating duct and tubular carcinoma
4413301000006118		Infiltrating duct and mucinous carcinoma
4413311000006115		Infiltrating duct and cribriform carcinoma

4413321000006111		Infiltrating duct and colloid carcinoma
4413331000006114		Infiltrating lobular mixed with other types of carcinoma
4413341000006116		Polymorphous low grade adenocarcinoma
4413351000006119		Terminal duct adenocarcinoma
4413361000006117		Acinar cell cystadenocarcinoma
4413371000006112		Adenocarcinoma with neuroendocrine differentiation
4413381000006110		Carcinoma with neuroendocrine differentiation
4413391000006113		Metaplastic carcinoma
4413401000006110		Hepatoid adenocarcinoma
4413411000006113		Hepatoid carcinoma
4413451000006114		Thymoma, type A, malignant
4413461000006111		Thymoma, spindle cell, malignant
4413471000006116		Thymoma, medullary, malignant
4413501000006111		Thymoma, type AB, malignant
4413511000006114		Thymoma, mixed type, malignant
4413571000006117		Thymoma, type B1, malignant
4413581000006119		Thymoma, predominantly cortical, malignant
4413591000006116		Thymoma, organoid, malignant
4413601000006112		Thymoma, lymphocytic, malignant

4413611 0000061 10		Thymoma, lymphocyte-rich, malignant
4413641 0000061 14		Thymoma, type B2, malignant
4413651 0000061 11		Thymoma, cortical, malignant
4413691 0000061 17		Thymoma, type B3, malignant
4413701 0000061 17		Well differentiated thymic carcinoma
4413711 0000061 19		Thymoma, epithelial, malignant
4413721 0000061 10		Thymoma, atypical, malignant
4413741 0000061 15		Thymic carcinoma
4413811 0000061 11		Carcinoma showing thymus-like element
4413821 0000061 15		Carcinoma showing thymus-like differentiation
4414211 0000061 14		Solitary fibrous tumour, malignant
4414221 0000061 18		Solitary fibrous tumor, malignant
4414681 0000061 17		Gastrointestinal stromal tumor, malignant
4414691 0000061 19		GIST, malignant
4414701 0000061 19		Gastrointestinal stromal tumour, malignant
4414731 0000061 10		Malignant cystic nephroma
4414741 0000061 17		Malignant multilocular cystic nephroma
4415141 0000061 15		Malignant tenosynovial giant cell tumour
4415151 0000061 18		Giant cell tumor of tendon sheath, malignant

4415161 0000061 16		Malignant tenosynovial giant cell tumor
4415171 0000061 11		Giant cell tumour of tendon sheath, malignant
4415471 0000061 17		Perineurioma, malignant
4415791 0000061 13		Malignant reticulosis [obs]
4415801 0000061 14		Malignant midline reticulosis [obs]
4415871 0000061 15		Malignant lymphoma, lymphoblastic
4415881 0000061 17		Malignant lymphoma, convoluted cell [obs]
4418241 0000061 16		Papillary serous tumor of low malignant potential
4418281 0000061 10		Papillary serous tumour of low malignant potential
4418321 0000061 16		Mucinous tumor of low malignant potential
4418371 0000061 15		Mucinous tumour of low malignant potential
4418441 0000061 15		Papillary mucinous tumor of low malignant potential
4418451 0000061 18		Papillary mucinous tumour of low malignant potential
4419051 0000061 19		Papillary transitional cell carcinoma, non-invasive
4419061 0000061 17		Papillary urothelial carcinoma, non-invasive
4419111 0000061 16		Cribriform carcinoma in situ
4419121 0000061 12		Ductal carcinoma in situ, cribriform type
4419131 0000061 10		Ductal carcinoma in situ, solid type
4419141 0000061 17		Intraductal carcinoma, solid type

4419191000006114		Malignant myoepithelioma
4419201000006112		Myoepithelial carcinoma
4419261000006113		Malignant serous cystadenofibroma
4419271000006118		Malignant serous adenofibroma
4419321000006114		Malignant mucinous cystadenofibroma
4419331000006112		Malignant mucinous adenofibroma
4419441000006110		Fetal adenocarcinoma
4419451000006112		Foetal adenocarcinoma
4419461000006114		Nodular hidradenoma, malignant
4419471000006119		Hidradenocarcinoma
4419481000006116		Malignant eccrine spiradenoma
4419491000006118		Sclerosing sweat duct carcinoma
4419501000006114		Syringomatous carcinoma
4419511000006112		Microcystic adnexal carcinoma
4419531000006118		Eccrine papillary adenocarcinoma
4419541000006111		Digital papillary adenocarcinoma
4419551000006113		Mucinous cystadenocarcinoma, non-invasive
4419581000006117		Intratubular malignant germ cells
4419731000006111		Steroid cell tumour, malignant

4419741 0000061 18		Steroid cell tumor, malignant
4419751 0000061 16		Glomus tumour, malignant
4419761 0000061 19		Glomus tumor, malignant
4419881 0000061 19		Parafollicular cell carcinoma
4419891 0000061 16		C cell carcinoma
4447730 19	B54 0.11	Phaeochromocytoma
4447990 14	B62 7D0 0	Diffuse non-Hodgkin's centroblastic lymphoma
4455860 18	B62 3.00	Malignant histiocytosis
4455870 10	B62 x40 0	Malignant reticulosis
4505481 0000061 13		Basal cell carcinoma - morpheic
4505491 0000061 11		Morpheaform basal cell carcinoma
4506551 0000061 11		Odontogenic ghost cell carcinoma
4510770 18	D21 200 0	Anaemia in ovarian carcinoma
4514280 19	B21 5.00	Malignant neoplasm of epiglottis NOS
4514290 10	B62 7C0 0	Follicular non-Hodgkin's lymphoma
4533010 18	B00 7.00	Malignant tumour of lip
4539441 0000061 14		History of malignant melanoma
4540850 11	B32 570 0	Malignant melanoma of back
4540910 13	B59 3.00	Primary malignant neoplasm of unknown site
4540961 0000061 11		History of upper gastrointestinal tract neoplasm
4540981 0000061 18		History of lower gastrointestinal tract neoplasm

454201010	B331000	Malignant neoplasm of canthus
455404011	B332200	Malignant neoplasm of pinna NEC
455405012	B335A00	Malignant neoplasm of skin of scapular region
456641000006119	B670.00	Acute erythraemia and erythroleukaemia
457109011	B325800	Malignant melanoma of chest wall
457110018	B331100	Malignant neoplasm of upper eyelid
457111019	B331200	Malignant neoplasm of lower eyelid
457317011	B630300	Lambda light chain myeloma
457318018	B470200	Seminoma of undescended testis
457319014	B471000	Seminoma of descended testis
458201000006115	B690.00	M4 - Acute myelomonocytic leukaemia
459378019	B36..00	Local recurrence of malignant tumour of breast
459399016	B498.00	Local recurrence of malignant tumour of urinary bladder
459527013	B139.00	Hereditary nonpolyposis colon cancer
459741000006115	B65y100	APL - Acute promyelocytic leukaemia
460259014	ZV10.00	[V]Personal history of malignant neoplasm
460263019	ZV10300	History of malignant neoplasm of breast
460264013	ZV10412	[V]Personal history of malignant neoplasm of genital organ
460265014	ZV10500	[V]Personal history of malignant neoplasm of urinary organ
460270019	ZV10y00	[V]Personal history of other specified malignant neoplasm
460271015	ZV10z00	[V]Personal history of unspecified malignant neoplasm

4614980 13	ZVu 6J0 0	[X]Personal history of other neoplasms
4641121 0000061 19		Cancer of cervix screening not wanted
4739910 0000011 9	B47 1z0 0	Malignant neoplasm of descended testis
4740770 18	B69 1.00	Chronic myelomonocytic leukaemia
4745221 0000061 16		Malignant tumour of oral aspect of upper lip
4745231 0000061 18		Malignant tumour of inner aspect of upper lip
4745241 0000061 11		Malignant tumour of buccal aspect of upper lip
4745261 0000061 10		Malignant tumor of upper labial mucosa
4745311 0000061 15		Malignant tumor of buccal aspect of upper lip
4745321 0000061 11		Malignant tumor of inner aspect of upper lip
4745331 0000061 14		Malignant tumor of oral aspect of upper lip
4745341 0000061 16		Malignant tumour of fraenum of upper lip
4745351 0000061 19		Malignant tumor of frenum of upper lip
4745381 0000061 10		Malignant tumour of fraenum of lower lip
4745391 0000061 13		Malignant tumor of frenum of lower lip
4745411 0000061 13		Malignant tumour of labial mucosa
4745421 0000061 17		Malignant tumor of labial mucosa
4745441 0000061 12		Malignant tumor of buccal aspect of lip
4745451 0000061 14		Malignant tumor of mucosa of lip
4745461 0000061 11		Malignant tumor of oral aspect of lip

4745471000006116		Malignant tumor of inner aspect of lip
4745481000006118		Malignant tumour of inner aspect of lip
4745491000006115		Malignant tumour of buccal aspect of lip
4745501000006111		Malignant tumour of oral aspect of lip
4745511000006114		Malignant tumour of mucosa of lip
4745551000006110		Malignant tumour of anterior two-thirds of tongue - dorsal surface
4745571000006117		Malignant tumor of anterior two-thirds of tongue - dorsal surface
4745621000006119		Malignant tumour of anterior two-thirds of tongue - ventral surface
4745641000006114		Malignant tumor of anterior two-thirds of tongue - ventral surface
4745651000006111		Malignant tumour of fraenum linguae
4745661000006113		Malignant tumor of frenum linguae
4745681000006115		Malignant tumour of junctional zone of tongue
4745691000006117		Malignant tumor of junctional zone of tongue
4745711000006119		Malignant tumour of anterior floor of mouth
4745721000006110		Malignant tumor of anterior floor of mouth
4745741000006115		Malignant tumour of lateral floor of mouth
4745761000006116		Malignant tumor of lateral floor of mouth
4745781000006114		Malignant tumor of vestibule of mouth
4745801000006113		Malignant tumour of upper buccal sulcus

4745821000006115		Malignant tumor of upper buccal sulcus
4745831000006117		Malignant tumour of lower buccal sulcus
4745841000006110		Malignant tumor of lower buccal sulcus
4745861000006114		Malignant tumour of upper labial sulcus
4745881000006116		Malignant tumor of upper labial sulcus
4745891000006118		Malignant tumour of lower labial sulcus
4745901000006119		Malignant tumor of lower labial sulcus
4745931000006110		Malignant tumour of tonsillar pillar
4745941000006117		Malignant tumour of palatopharyngeal arch
4745951000006115		Malignant tumour of posterior tonsillar pillar
4745961000006118		Malignant tumour of pharyngopalatine arch
4745971000006113		Malignant tumour of posterior faucial pillar
4745981000006111		Malignant tumor of tonsillar pillar
4746011000006113		Malignant tumor of palatopharyngeal arch
4746021000006117		Malignant tumor of pharyngopalatine arch
4746031000006119		Malignant tumor of posterior faucial pillar
4746041000006112		Malignant tumor of posterior tonsillar pillar
4746081000006118		Malignant tumour of glossoepiglottic fold
4746091000006115		Malignant tumor of glossoepiglottic fold

4746111000006112		Malignant tumour of posterior wall of oropharynx
4746121000006116		Malignant tumor of posterior wall of oropharynx
4746151000006113		Malignant tumor of epipharynx
4746161000006110		Malignant tumor of postnasal space
4746171000006115		Cancer of nasopharynx
4746191000006119		CA - Cancer of nasopharynx
4746201000006116		Malignant tumour of epipharynx
4746211000006118		Malignant tumour of postnasal space
4746221000006114		Malignant tumor of nasopharynx
4746251000006117		Malignant tumor of posterior wall of nasopharynx
4746261000006115		Malignant tumour of adenoid
4746271000006110		Malignant tumor of adenoid
4746301000006112		Malignant tumor of nasopharyngeal tonsil
4746311000006110		Malignant tumor of pharyngeal tonsil
4746321000006119		Malignant tumour of pharyngeal tonsil
4746331000006116		Malignant tumour of nasopharyngeal tonsil
4746341000006114		Malignant tumour of pharyngeal recess
4746351000006111		Malignant tumour of fossa of Rosenmuller
4746361000006113		Malignant tumor of pharyngeal recess

4746371 0000061 18		Malignant tumor of fossa of Rosenmuller
4746391 0000061 17		Malignant tumour of opening of auditory tube
4746401 0000061 15		Malignant tumor of opening of auditory tube
4746431 0000061 11		Malignant tumor of anterior wall of nasopharynx
4746461 0000061 19		Malignant tumour of nasopharyngeal soft palate surface
4746471 0000061 14		Malignant tumor of nasopharyngeal soft palate surface
4746491 0000061 10		Malignant tumour aryepiglottic fold - hypopharyngeal aspect
4746501 0000061 19		Malignant tumor aryepiglottic fold - hypopharyngeal aspect
4746541 0000061 17		Malignant tumour of Waldeyer's ring
4746551 0000061 15		Malignant tumor of Waldeyer's ring
4746571 0000061 13		Malignant tumor of Waldeyer ring
4746581 0000061 11		Malignant tumour of cervical part of oesophagus
4746601 0000061 18		Malignant tumor of cervical part of esophagus
4746611 0000061 15		Malignant neoplasm of cervical esophagus
4746621 0000061 11		Malignant tumour of thoracic part of oesophagus
4746631 0000061 14		Malignant neoplasm of thoracic esophagus
4746641 0000061 16		Malignant tumor of thoracic part of esophagus
4746661 0000061 17		Malignant tumour of abdominal part of oesophagus
4746681 0000061 10		Malignant tumor of abdominal part of esophagus

4746691000006113		Malignant neoplasm of abdominal esophagus
4746701000006113		Malignant tumour of upper third of oesophagus
4746711000006111		Malignant neoplasm of upper third of esophagus
4746721000006115		Malignant tumor of upper third of esophagus
4746761000006114		Malignant tumour of middle third of oesophagus
4746771000006119		Malignant tumor of middle third of esophagus
4746781000006116		Malignant neoplasm of middle third of esophagus
4746821000006110		Malignant tumour of lower third of oesophagus
4746841000006115		Malignant neoplasm of lower third of esophagus
4746851000006118		Malignant tumor of lower third of esophagus
4746881000006114		Malignant tumour of cardia
4746901000006111		Malignant tumor of cardia
4746941000006113		Malignant neoplasm of cardio-esophageal junction of stomach
4746951000006110		Malignant neoplasm of gastro-esophageal junction
4746971000006117		Malignant neoplasm of cardioesophageal junction of stomach
4746981000006119		Malignant tumour of pylorus
4746991000006116		Malignant tumor of pylorus
4747031000006116		Malignant tumour of pyloric antrum
4747041000006114		Malignant tumor of pyloric antrum

4747061 0000061 13		Malignant tumour of fundus of stomach
4747081 0000061 15		Malignant tumor of fundus of stomach
4747101 0000061 11		Malignant tumour of body of stomach
4747111 0000061 14		Malignant tumor of body of stomach
4747141 0000061 13		Malignant tumour of Meckel's diverticulum
4747151 0000061 10		Malignant tumor of Meckel's diverticulum
4747171 0000061 17		Malignant tumor of Meckel diverticulum
4747281 0000061 10		Intrahepatic bile duct cancer
4747311 0000061 12		Malignant tumour of body of pancreas
4747321 0000061 16		Malignant tumor of body of pancreas
4747351 0000061 13		Malignant tumour of tail of pancreas
4747371 0000061 15		Malignant tumor of tail of pancreas
4747391 0000061 19		Malignant tumour of pancreatic duct
4747401 0000061 17		Malignant tumor of pancreatic duct
4747421 0000061 10		Malignant tumour of Islets of Langerhans
4747431 0000061 13		Malignant Islet cell tumour
4747451 0000061 18		Malignant Islet cell tumor
4747461 0000061 16		Malignant tumor of Islets of Langerhans
4747501 0000061 16		Malignant tumor of peritoneum and retroperitoneum

4747541 0000061 19		Malignant neoplasm of retrocecal tissue
4747591 0000061 11		Malignant neoplasm of mesocecum
4747681 0000061 12		Malignant neoplasm of nasal turbinate
4747701 0000061 10		Malignant tumour of nasal vestibule
4747711 0000061 13		Malignant tumor of nasal vestibule
4747741 0000061 12		Malignant tumour of Eustachian tube
4747751 0000061 14		Malignant tumor of Eustachian tube
4747771 0000061 16		Malignant tumor of auditory tube
4747781 0000061 18		Malignant tumour of auditory tube
4747791 0000061 15		Malignant tumour of tympanic cavity
4747811 0000061 16		Malignant tumor of tympanic cavity
4747821 0000061 12		Malignant tumour of tympanic antrum
4747831 0000061 10		Malignant tumor of tympanic antrum
4747861 0000061 18		Malignant tumour of glottis
4747871 0000061 13		Malignant tumor of glottis
4747901 0000061 13		Malignant tumour of supraglottis
4747921 0000061 15		Malignant tumor of supraglottis
4748061 0000061 12		Malignant neoplasm of right middle lobe of lung
4748181 0000061 18		Malignant neoplasm of ribs and/or sternum and/or clavicle

4748231 0000061 13		Malignant neoplasm of costovertebral joint
4748281 0000061 14		Malignant neoplasm of navicular bone of hand
4748331 0000061 17		Malignant neoplasm of trapezial bone
4748651 0000061 15		Malignant neoplasm of connective and soft tissue of forearm
4748911 0000061 10		Malignant neoplasm, overlapping lesion of connective and soft tissue
4748961 0000061 13		Malignant melanoma of ear and/or external auditory canal
4748981 0000061 15		Malignant melanoma of ear and/or external auricular canal
4749081 0000061 14		Malignant melanoma of trunk (excluding scrotum)
4749171 0000061 12		Malignant melanoma of forearm
4749341 0000061 18		Malignant neoplasm of skin of ear and external auditory canal
4749431 0000061 16		Malignant neoplasm of scalp AND/OR skin of neck
4749451 0000061 11		Malignant neoplasm of skin of scalp
4749501 0000061 13		Malignant neoplasm of skin of forearm
4749881 0000061 18		Malignant neoplasm of Gartner duct
4749921 0000061 14		Malignant tumor of undescended testis
4749931 0000061 12		Cancer of undescended testis
4749951 0000061 17		Malignant tumour of retained testis
4749961 0000061 15		Malignant tumor of retained testis
4749971 0000061 10		Malignant tumour of ectopic testis

4749991000006111		Cancer of ectopic testis
4750001000006111		Malignant tumor of ectopic testis
4750011000006114		Malignant tumour of body of penis
4750021000006118		Malignant tumour of shaft of penis
4750031000006115		Malignant tumor of body of penis
4750051000006110		Cancer of body of penis
4750061000006112		Malignant tumor of shaft of penis
4750071000006117		Malignant tumour of seminal vesicle
4750091000006116		Malignant tumor of seminal vesicle
4750101000006110		Malignant tumour of tunica vaginalis
4750111000006113		Malignant tumor of tunica vaginalis
4750131000006119		Malignant tumour of trigone of urinary bladder
4750141000006112		Malignant tumor of trigone of urinary bladder
4750161000006111		Malignant tumour of trigone of bladder
4750171000006116		Malignant tumor of trigone of bladder
4750181000006118		Malignant tumour of vault of bladder
4750191000006115		Malignant tumour of bladder dome
4750201000006117		Malignant neoplasm of vault of bladder
4750221000006110		Malignant tumor of bladder dome

4750231000006113		Malignant tumor of vault of bladder
4750271000006111		Malignant tumour of bladder neck
4750281000006114		Malignant tumor of bladder neck
4750301000006113		Malignant tumour of ureteric orifice
4750321000006115		Malignant tumor of ureteric orifice
4750341000006110		Overlapping malignant neoplasm of urinary bladder
4750351000006112		Overlapping malignant neoplasm of bladder
4750361000006114		Malignant tumour of kidney parenchyma
4750381000006116		Malignant tumor of kidney parenchyma
4750401000006116		Malignant tumour of renal calyx
4750411000006118		Malignant neoplasm of renal calyx
4750421000006114		Malignant tumor of renal calyx
4750431000006112		Malignant tumour of pelviureteric junction
4750441000006119		Malignant tumor of pelviureteric junction
4750461000006115		Malignant neoplasm of pelviureteric junction
4750501000006115		Malignant tumour of ciliary body
4750521000006113		Malignant tumor of ciliary body
4750531000006111		Malignant tumour of iris
4750551000006116		Malignant tumor of iris

4750601 0000061 19		Malignant tumour of lacrimal gland
4750621 0000061 12		Malignant tumor of lacrimal gland
4750651 0000061 15		Malignant neoplasm of lacrimal duct
4750711 0000061 12		Malignant tumour of hypothalamus
4750721 0000061 16		Malignant tumor of hypothalamus
4750771 0000061 15		Malignant tumour of choroid plexus
4750781 0000061 17		Malignant tumor of choroid plexus
4750881 0000061 10		Malignant tumor of cranial nerve
4751011 0000061 18		Peripheral nerve cancer, thorax
4751071 0000061 10		Malignant tumour of craniopharyngeal duct
4751081 0000061 13		Malignant tumor of craniopharyngeal duct
4751131 0000061 13		Malignant tumour of thorax
4751141 0000061 15		Malignant tumor of thorax
4751161 0000061 16		Malignant tumor of abdomen
4751171 0000061 11		Malignant tumour of abdomen
4751301 0000061 19		Metastatic malignant neoplasm to uterine cervix
4751311 0000061 16		Metastatic malignant neoplasm to cervix uteri
4751321 0000061 12		Secondary malignant neoplasm of uterine cervix
4751331 0000061 10		Cancer metastatic to cervix

4751341 0000061 17		Secondary malignant neoplasm of epididymis and vas deferens
4752881 0000061 11		Malignant mast cell tumors
4752991 0000061 18		Diffuse malignant lymphoma - small non-cleaved cell
4753001 0000061 17		Non-Burkitt's malignant lymphoma - undifferentiated cell
4753021 0000061 10		Malignant lymphoma - undifferentiated cell type
4753031 0000061 13		Malignant lymphoma - small cleaved cell
4753061 0000061 16		Malignant lymphoma, lymphocytic - poorly differentiated
4753071 0000061 11		Malignant lymphoma - mixed small and large cell
4753111 0000061 15		Malignant lymphoma - mixed lymphocytic-histiocytic
4755801 0000061 11		Carcinoma in situ of splenic flexure
4755991 0000061 12		Carcinoma in situ of carina
4756071 0000061 19		Carcinoma in situ of skin of external auditory canal
4756241 0000061 17		Carcinoma in situ of breast and genitourinary system
4756261 0000061 18		Non-invasive carcinoma of breast
4756971 0000061 15		Squamous cell carcinoma in situ
4757241 0000061 19		Dermoid cyst with malignant transformation
4757261 0000061 15		Choriocarcinoma combined with teratoma
4757371 0000061 18		Malignant lymphoma, stem cell type
4775791 0000061 13		Malignant secondary renovascular hypertension
4812660 13		Carcinoid tumour of uncertain malignant potential

482537012	B18y700	Malignant neoplasm of mesentery
482641017	B18y300	Malignant neoplasm of omentum
482690017	B451.00	Malignant neoplasm of labia majora
482691018	B452.00	Malignant neoplasm of labia minora
482730011	B511.00	Malignant neoplasm of frontal lobe
482731010	B512.00	Malignant neoplasm of temporal lobe
482732015	B513.00	Malignant neoplasm of parietal lobe
482733013	B514.00	Malignant neoplasm of occipital lobe
482734019	B515.00	Malignant neoplasm of cerebral ventricles
482737014	B521.00	Malignant neoplasm of cerebral meninges
482740014	B523.00	Malignant neoplasm of spinal meninges
482741013	B525.00	Malignant neoplasm of cauda equina
4870831000006114		Malignant hyperpyrexia due to anesthetic
4870841000006116		Malignant hyperpyrexia due to anaesthesia
4870851000006119		Malignant hyperpyrexia due to anesthesia
4870901000006112		Malignant hyperpyrexia caused by anaesthesia
4870911000006110		Malignant hyperpyrexia caused by anesthesia
4870921000006119		Malignant hyperpyrexia caused by anaesthetic
4870931000006116		Malignant hyperpyrexia caused by anesthetic
5004171000006111		Malignant meningitis
5004271000006116		Malignant ventriculitis
5004281000006118		Malignant ventriculitis, brain
501272016	B33z100	Naevoid basal cell carcinoma syndrome

5028061 0000061 16		Malignant tumor of eyelid
5028071 0000061 11		Malignant neoplasm of eyelid
5028101 0000061 18		Squamous cell carcinoma of eyelid
5028111 0000061 15		Basal cell carcinoma of eyelid
5028131 0000061 14		Sebaceous adenocarcinoma of eyelid
5028141 0000061 16		Meibomian gland carcinoma
5053901 0000061 14		Malignant chylothorax
5060881 0000061 13		Malignant vasovagal syndrome
5083021 0000061 11		Malignant oesophageal stricture
5083031 0000061 14		Malignant esophageal stricture
5087911 0000061 10		Malignant cystic tumour of exocrine pancreas
5087921 0000061 19		Malignant cystic tumor of exocrine pancreas
5087931 0000061 16		Cystadenocarcinoma of pancreas
5088651 0000061 19		Malignant retroperitoneal fibrosis
5097410 17	B64 0.00	Acute lymphoid leukaemia
5100290 18	B80 050 0	Carcinoma in situ of cheek
5100450 16	B81 080 0	Carcinoma in situ of vocal fold - glottis
5100610 19	B80 0.11	Carcinoma in situ of oral cavity
5100740 13	B80 240 0	Carcinoma in situ of pyloric canal
5112500 10	B58 y00 0	Secondary malignant neoplasm of breast

511385013	B58y411	Secondary cancer of the vulva
5124411000006114		Malignant acanthosis nigricans
5141001000006116		Polymyositis with malignant disease
5141031000006112		Dermatomyositis with malignant disease
5144281000006116		Malignant neoplasm of nasopharyngeal wall
5168481000006119		Metastatic malignant neoplasm to nasopharynx
5168491000006116		Secondary malignant neoplasm of nasopharynx
51742100000115	9Ow1.00	Bowel cancer detected by national screening programme
5179631000006117		Secondary malignant neoplasm of nasopharyngeal wall
5179641000006110		Metastatic malignant neoplasm to nasopharyngeal wall
5257341000006114		Malignant tertian fever
527801000006112	B602500	Burkitt's lymphoma of lymph nodes of inguinal region and lower limb
531851000006119	B34..11	Ca female breast
531861000006117	B80..11	Carcinoma in situ of gastrointestinal tract
5324131000006119		Malignant squamous tumour
5324141000006112		Malignant squamous tumor
5324151000006114		Malignant squamous tumor, primary
5324161000006111		Malignant squamous tumour, primary
5324171000006116		Squamous carcinoma in situ

5324181000006118		Squamous carcinoma in situ - category
5324231000006113		Malignant basal cell tumour
5324241000006115		Malignant basal cell tumor
5324301000006113		Malignant endocrine tumour morphology
5324311000006111		Malignant endocrine tumor morphology
5324321000006115		Neuroendocrine carcinoma
5324331000006117		Merkel cell carcinoma
5324341000006110		Trabecular cell carcinoma of skin
5324641000006117		Malignant endometrioid tumour
5324651000006115		Malignant endometrioid tumor
5324731000006118		Fibrolamellar hepatocellular carcinoma
5324841000006116		Adenocarcinoma with metaplasia
5324991000006117		Myxoid malignant fibrous histiocytoma
5325011000006110		Malignant lipomatous tumour morphology
5325021000006119		Malignant lipomatous tumor morphology
5325061000006113		Malignant myomatous tumour
5325071000006118		Malignant myomatous tumor
5325101000006111		Malignant stromal tumour
5325111000006114		Malignant stromal tumor

5325141 0000061 13		Malignant haemangioma
5325151 0000061 10		Malignant hemangioma
5325201 0000061 18		Malignant lymphangioma
5325221 0000061 11		Malignant osseous and chondromatous tumour morphology
5325231 0000061 14		Malignant osseous and chondromatous tumor morphology
5325451 0000061 18		Malignant pineal germinoma
5325611 0000061 17		Malignant Schwannoma with divergent mesenchymal and epithelial differentiation
5325621 0000061 13		Malignant Schwannoma with divergent epithelial differentiation
5325631 0000061 11		Malignant Schwannoma with divergent mesenchymal differentiation
5325641 0000061 18		Epithelioid malignant nerve sheath tumour
5325651 0000061 16		Epithelioid malignant nerve sheath tumor
5325661 0000061 19		Malignant peripheral nerve sheath tumour, epithelioid
5325671 0000061 14		Malignant peripheral nerve sheath tumor, epithelioid
5325691 0000061 10		Melanotic malignant peripheral nerve sheath tumour
5325701 0000061 10		Malignant melanotic Schwannoma
5325711 0000061 13		Melanotic malignant peripheral nerve sheath tumor
5333210 0000611 9	B.... 11	Cancer
5341501 0000061 15		TNM Carcinoma of skin tumour staging
5341511 0000061 17		TNM Carcinoma of skin tumor staging

5341521000006113		Tumor-node-metastasis (TNM) carcinoma of skin tumor staging
5341531000006111		Tumor-node-metastasis (TNM) carcinoma of skin tumour staging
5341541000006118		TNM Malignant melanoma of skin staging
5341551000006116		Tumor-node-metastasis (TNM) malignant melanoma of skin staging
5342201000006114		TNM Carcinoma of eyelid staging
5342211000006112		Tumor-node-metastasis (TNM) carcinoma of eyelid staging
5342221000006116		TNM Malignant melanoma of eyelid staging
5342231000006118		Tumor-node-metastasis (TNM) malignant melanoma of eyelid staging
5342241000006111		TNM Carcinoma of conjunctiva staging
5342251000006113		Tumor-node-metastasis (TNM) carcinoma of conjunctiva staging
5342261000006110		TNM Malignant melanoma of conjunctiva staging
5342271000006115		Tumor-node-metastasis (TNM) malignant melanoma of conjunctiva staging
5342281000006117		TNM Malignant melanoma of uvea staging
5342291000006119		Tumor-node-metastasis (TNM) malignant melanoma of uvea staging
5342301000006118		TNM Malignant melanoma of iris staging
5342311000006115		Tumor-node-metastasis (TNM) malignant melanoma of iris staging
5342321000006111		TNM Malignant melanoma of ciliary body staging
5342331000006114		Tumor-node-metastasis (TNM) malignant melanoma of ciliary body staging
5342341000006116		TNM Malignant melanoma of choroid staging

5342351000006119		Tumor-node-metastasis (TNM) malignant melanoma of choroid staging
5342401000006110		TNM Lacrimal gland carcinoma staging
5342411000006113		Tumor-node-metastasis (TNM) lacrimal gland carcinoma staging
5343391000006117		Carcinoma of vermilion border of upper lip
5343401000006115		Carcinoma of lipstick area of upper lip
5343411000006117		Carcinoma of external upper lip
5343421000006113		Carcinoma of vermilion border of lower lip
5343431000006111		Carcinoma of lipstick area of lower lip
5343441000006118		Carcinoma of external lower lip
5343491000006110		Carcinoma of fraenum of lip
5343501000006119		Carcinoma of frenum of lip
5343511000006116		Carcinoma in situ of fraenum of lip
5343521000006112		Carcinoma in situ of frenum of lip
5343591000006114		Carcinoma of fraenum of upper lip
5343601000006118		Carcinoma of frenum of upper lip
5343611000006115		Carcinoma in situ of fraenum of upper lip
5343621000006111		Carcinoma in situ of frenum of upper lip
5343671000006112		Carcinoma of fraenum of lower lip
5343681000006110		Carcinoma of frenum of lower lip

5343691000006113		Carcinoma in situ of fraenum of lower lip
5343701000006113		Carcinoma in situ of frenum of lower lip
5343711000006111		Carcinoma of commissure of lip
5343741000006110		Carcinoma in situ of anterior two-thirds of tongue - dorsal surface
5343751000006112		Carcinoma in situ of anterior two-thirds of tongue - dorsum
5343781000006116		Malignant tumour of anterior two-thirds of tongue - lateral margin
5343791000006118		Malignant tumour of anterior two-thirds of tongue - border
5343801000006117		Malignant tumor of anterior two-thirds of tongue - lateral margin
5343811000006119		Malignant tumor of anterior two-thirds of tongue - border
5343821000006110		Carcinoma in situ of anterior two-thirds of tongue - lateral margin
5343831000006113		Carcinoma in situ of anterior two-thirds of tongue - border
5343881000006114		Malignant tumour of tip of tongue
5343891000006112		Malignant tumor of tip of tongue
5343901000006111		Carcinoma in situ of tip of tongue
5343971000006117		Carcinoma of fraenum linguae
5343981000006119		Carcinoma of frenum linguae
5343991000006116		Carcinoma in situ of fraenum linguae
5344001000006113		Carcinoma in situ of frenum linguae
5344051000006112		Carcinoma of lingual tonsil

5344061 0000061 14		Carcinoma of upper gum
5344071 0000061 19		Carcinoma of lower gum
5344111 0000061 10		Carcinoma of anterior part of floor of mouth
5344121 0000061 19		Carcinoma in situ anterior floor of mouth
5344131 0000061 16		Carcinoma in situ anterior part of floor of mouth
5344141 0000061 14		Carcinoma in situ of anterior portion of floor of mouth
5344231 0000061 10		Carcinoma of lateral part of floor of mouth
5344241 0000061 17		Carcinoma in situ lateral floor of mouth
5344251 0000061 15		Carcinoma in situ lateral part of floor of mouth
5344261 0000061 18		Carcinoma in situ of lateral portion of floor of mouth
5344271 0000061 13		Carcinoma of hard palate
5344281 0000061 11		Carcinoma of soft palate
5344291 0000061 14		Carcinoma of uvula
5344301 0000061 10		Squamous cell carcinoma of buccal mucosa
5344311 0000061 13		Carcinoma of cheek mucosa
5344321 0000061 17		SCC - Squamous cell carcinoma of buccal mucosa
5344331 0000061 19		Carcinoma of buccal mucosa
5344401 0000061 18		Carcinoma of upper buccal sulcus
5344411 0000061 15		Carcinoma in situ of upper buccal sulcus

5344461 0000061 17		Carcinoma of lower buccal sulcus
5344471 0000061 12		Carcinoma in situ of lower buccal sulcus
5344541 0000061 15		Carcinoma of upper labial sulcus
5344551 0000061 18		Carcinoma in situ upper labial sulcus
5344601 0000061 16		Carcinoma of lower labial sulcus
5344611 0000061 18		Carcinoma in situ of lower labial sulcus
5344641 0000061 19		Carcinoma of retromolar area
5344681 0000061 13		Malignant tumor of anterior pillar of fauces
5344691 0000061 11		Malignant tumour of anterior faucial pillar
5344701 0000061 11		Malignant tumour of anterior tonsillar pillar
5344711 0000061 14		Malignant tumour of palatoglossal arch
5344721 0000061 18		Malignant tumor of palatoglossal arch
5344731 0000061 15		Malignant tumor of anterior tonsillar pillar
5344741 0000061 13		Malignant tumor of anterior faucial pillar
5344761 0000061 12		Carcinoma in situ of anterior pillar of fauces
5344771 0000061 17		Carcinoma of parotid gland
5344821 0000061 13		Carcinoma of submandibular gland
5344831 0000061 11		Carcinoma of sublingual gland
5344991 0000061 19		Malignant tumour of nasal skeleton

5345001 0000061 14		Malignant tumor of nasal skeleton
5345071 0000061 15		Malignant tumour of inferior turbinate
5345081 0000061 17		Malignant tumor of inferior turbinate
5345171 0000061 19		Malignant tumour of middle turbinate
5345181 0000061 16		Malignant tumor of middle turbinate
5345241 0000061 18		Malignant tumor of posterior margin of nasal septum and choanae
5345441 0000061 17		Malignant tumour of inferior surface of soft palate
5345451 0000061 15		Malignant tumor of inferior surface of soft palate
5345571 0000061 12		Malignant tumour of anterior commissure
5345581 0000061 10		Malignant tumor of anterior commissure
5345591 0000061 13		Carcinoma in situ of anterior commissure
5345641 0000061 15		Malignant tumour of posterior commissure
5345651 0000061 18		Malignant tumor of posterior commissure
5345661 0000061 16		Carcinoma in situ of posterior commissure
5345711 0000061 10		Malignant tumour of suprahyoid epiglottis
5345721 0000061 19		Malignant tumor of suprahyoid epiglottis
5345771 0000061 18		Malignant tumour of infrahyoid epiglottis
5345781 0000061 15		Malignant tumor of infrahyoid epiglottis
5345851 0000061 17		Malignant tumour of laryngeal ventricle

5345861000006115		Malignant tumor of laryngeal ventricle
5345871000006110		Malignant tumor of laryngeal ventricular cavity
5345881000006113		Malignant tumor of laryngeal ventricular sinus
5345891000006111		Malignant tumour of laryngeal ventricular sinus
5345901000006110		Malignant tumour of laryngeal ventricular cavity
5346011000006116		Malignant tumour of parapharyngeal space
5346021000006112		Malignant tumor of parapharyngeal space
5346111000006117		Carcinoma of cervical part of oesophagus
5346121000006113		Carcinoma of cervical part of esophagus
5346131000006111		Carcinoma of thoracic part of oesophagus
5346141000006118		Carcinoma of thoracic part of esophagus
5346151000006116		Carcinoma in situ of thoracic part of oesophagus
5346161000006119		Carcinoma in situ of thoracic part of esophagus
5346171000006114		Carcinoma in situ of thoracic oesophagus
5346181000006112		Carcinoma in situ of thoracic esophagus
5346191000006110		Carcinoma of abdominal part of oesophagus
5346201000006113		Carcinoma of abdominal part of esophagus
5346211000006111		Carcinoma in situ of abdominal part of oesophagus
5346221000006115		Carcinoma in situ of abdominal part of esophagus

5346231000006117		Carcinoma of upper third of oesophagus
5346241000006110		Carcinoma of upper third of esophagus
5346251000006112		Carcinoma of middle third of oesophagus
5346261000006114		Carcinoma of middle third of esophagus
5346271000006119		Carcinoma of lower third of oesophagus
5346281000006116		Carcinoma of lower third of esophagus
5346291000006118		Carcinoma of cardia
5346301000006117		Carcinoma of fundus of stomach
5346311000006119		Carcinoma of body of stomach
5346321000006110		Carcinoma of pyloric antrum
5346331000006113		Carcinoma of pylorus
5346341000006115		Carcinoma of lesser curve of stomach
5346351000006118		Carcinoma in situ of lesser curve of stomach
5346361000006116		Carcinoma in situ of lesser curvature of stomach
5346371000006111		Carcinoma of greater curve of stomach
5346381000006114		Carcinoma of duodenum
5346461000006110		Adenocarcinoma of rectum
5346521000006117		Malignant tumour of anorectal junction
5346531000006119		Malignant tumor of anorectal junction

5346761000006115		Carcinoma of ampulla of Vater
5346771000006110		Ampullary carcinoma
5346791000006111		Malignant tumour of endocrine pancreas
5346801000006112		Malignant tumor of endocrine pancreas
5346811000006110		Carcinoma of endocrine pancreas
5346821000006119		Endocrine pancreatic carcinoma
5346931000006114		Adenoid cystic carcinoma of trachea
5346951000006119		Squamous cell carcinoma of trachea
5346981000006110		Squamous cell carcinoma of bronchus
5346991000006113		SCC - Squamous cell carcinoma of bronchus
5347001000006117		Carcinoma of bronchus
5347011000006119		CA - Carcinoma of bronchus
5347021000006110		BC - Bronchogenic carcinoma
5347031000006113		Bronchial carcinoma
5347041000006115		Bronchogenic carcinoma
5347081000006114		Malignant tumour of lung parenchyma
5347091000006112		Malignant tumor of lung parenchyma
5347131000006114		Carcinoma of lung parenchyma
5347151000006119		Giant cell carcinoma of lung

5347161 0000061 17		Small cell carcinoma of lung
5347171 0000061 12		SCLC - Small cell lung cancer
5347191 0000061 13		Oat cell carcinoma of lung
5347211 0000061 14		Epidermoid carcinoma of lung
5347221 0000061 18		SCC - Squamous cell carcinoma of lung
5347301 0000061 15		NSCLC - Non-small cell lung cancer
5347351 0000061 16		Carcinoma in situ of lung parenchyma
5347561 0000061 14		Malignant epithelial neoplasm of skin
5347571 0000061 19		Malignant tumour of surface epithelium
5347581 0000061 16		Malignant tumor of surface epithelium
5347601 0000061 14		SCC - Cutaneous squamous cell carcinoma
5347611 0000061 12		Cutaneous squamous cell carcinoma
5347621 0000061 16		SCC - Squamous cell carcinoma of skin
5347631 0000061 18		Spinous cell carcinoma
5347641 0000061 11		Clear cell squamous cell carcinoma of skin
5347651 0000061 13		SCC - Clear cell squamous cell carcinoma of skin
5347661 0000061 10		Spindle cell squamous carcinoma of skin
5347681 0000061 17		Acantholytic squamous cell carcinoma of skin
5347691 0000061 19		SCC - Acantholytic squamous cell carcinoma of skin

5347701000006119		Plantar verrucous carcinoma
5347721000006112		Carcinoma cuniculatum
5347731000006110		Squamous cell carcinoma in situ of skin
5347751000006115		IEC - Intraepidermal carcinoma of skin
5347761000006118		SCC - Squamous cell carcinoma in situ of skin
5347771000006113		Intraepidermal carcinoma of skin
5347781000006111		Cancer in situ skin, squamous cell
5348531000006118		BCC - Basal cell carcinoma of skin
5348551000006113		Cancer of skin, basal cell
5348561000006110		Basosquamous carcinoma of skin
5348571000006115		BCC - Metatypical basal cell carcinoma of skin
5348581000006117		Metatypical basal cell carcinoma of skin
5348661000006111		Malignant skin tumour with eccrine differentiation
5348671000006116		Malignant sweat gland tumour
5348681000006118		Sweat gland carcinoma
5348691000006115		Malignant sweat gland tumor
5348701000006115		Malignant skin tumor with eccrine differentiation
5348711000006117		Eccrine porocarcinoma
5348721000006113		Eccrine porocarcinoma of skin

5348731 0000061 11		Digital papillary eccrine carcinoma of skin
5348741 0000061 18		Sweat gland adenocarcinoma
5348751 0000061 16		Hidradenocarcinoma
5348761 0000061 19		Papillary digital eccrine carcinoma
5348771 0000061 14		Digital papillary adenocarcinoma
5348781 0000061 12		Basal cell carcinoma with eccrine differentiation
5348791 0000061 10		Adenoid cystic eccrine carcinoma
5348801 0000061 11		Primary cutaneous adenocystic carcinoma
5348811 0000061 14		Adenoid cystic eccrine carcinoma of skin
5348821 0000061 18		Microcystic adnexal carcinoma
5348831 0000061 15		Malignant syringoma
5348841 0000061 13		Syringoid eccrine carcinoma
5348851 0000061 10		Syringomatous sweat duct carcinoma
5348861 0000061 12		Sclerosing sweat duct carcinoma
5348871 0000061 17		Microcystic adnexal carcinoma of skin
5348881 0000061 19		Mucoepidermoid carcinoma of skin
5348891 0000061 16		Mucoepidermal eccrine carcinoma
5348901 0000061 17		Primary mucoepidermoid carcinoma of skin
5348911 0000061 19		Mucinous eccrine carcinoma

5348921 0000061 10		Mucinous eccrine carcinoma of skin
5348931 0000061 13		Primary mucinous carcinoma of skin
5349221 0000061 11		Malignant skin tumour with apocrine differentiation
5349231 0000061 14		Malignant skin tumor with apocrine differentiation
5349281 0000061 10		Superficial spreading malignant melanoma of skin
5349301 0000061 14		SSMM - Superficial spreading malignant melanoma of skin
5349321 0000061 16		Nodular malignant melanoma of skin
5349351 0000061 13		Acral lentiginous malignant melanoma of skin
5349381 0000061 17		ALMM - Acral lentiginous malignant melanoma of skin
5349391 0000061 19		Malignant melanoma arising in intradermal naevus
5349401 0000061 17		Malignant melanoma arising in intradermal nevus
5349411 0000061 19		Malignant melanoma arising in congenital naevus
5349421 0000061 10		Malignant melanoma arising in congenital nevus
5350551 0000061 13		Malignant haemangiopericytoma of skin
5350561 0000061 10		Malignant hemangiopericytoma of skin
5351261 0000061 18		Malignant tumour of mesothelial tissue
5351271 0000061 13		Malignant tumor of mesothelial tissue
5351341 0000061 12		Malignant lipomatous tumour
5351351 0000061 14		Malignant lipomatous tumor

5351491 0000061 13		Malignant tumour of breast
5351501 0000061 17		Malignant tumor of breast
5351511 0000061 19		Breast cancer
5351521 0000061 10		CA - Breast cancer
5351531 0000061 13		Malignant neoplasm of breast
5351541 0000061 15		Carcinoma of breast
5351551 0000061 18		CA - Carcinoma of breast
5351561 0000061 16		Scirrhous carcinoma of breast
5351571 0000061 11		Inflammatory carcinoma of breast
5351611 0000061 18		Malignant phyllodes tumour of breast
5351621 0000061 14		Malignant phyllodes tumor of breast
5351631 0000061 12		Malignant cystosarcoma phyllodes of breast
5351711 0000061 14		Malignant epithelial tumour of ovary
5351721 0000061 18		Malignant epithelial tumor of ovary
5351731 0000061 15		Carcinoma of ovary
5351741 0000061 13		Serous papillary cystadenocarcinoma ovary
5351751 0000061 10		Mucinous cystadenocarcinoma of ovary
5351761 0000061 12		Endometrioid carcinoma ovary
5351811 0000061 17		Undifferentiated carcinoma of ovary

5351821 0000061 13		Undifferentiated ovarian cancer
5351881 0000061 12		Malignant sex cord tumour of ovary
5351891 0000061 10		Malignant sex cord tumor of ovary
5351901 0000061 14		Malignant granulosa cell tumour of ovary
5351911 0000061 12		Malignant granulosa cell tumor of ovary
5352061 0000061 16		Malignant germ cell tumour of ovary
5352071 0000061 11		Malignant germ cell tumor of ovary
5352081 0000061 14		Choriocarcinoma of ovary
5352101 0000061 18		Embryonal carcinoma of ovary
5352210 0000611 3	B16 0.11	Carcinoma of gallbladder
5352211 0000061 14		Endometrial carcinoma
5352301 0000061 15		Squamous cell carcinoma of cervix
5352311 0000061 17		Adenocarcinoma of cervix
5352321 0000061 13		Adenocarcinoma cervix uteri
5352331 0000061 11		Adenosquamous carcinoma of cervix
5352341 0000061 18		Carcinoma of cervix stage 0
5352351 0000061 16		Adenocarcinoma in situ of cervix
5352361 0000061 19		AIS - Adenocarcinoma in situ of cervix
5352371 0000061 14		Cervical ACIS (adenocarcinoma in situ)

5352401 0000061 12		Carcinoma of vagina
5352431 0000061 16		Squamous cell carcinoma of vulva
5352451 0000061 11		SCC - Squamous cell carcinoma of vulva
5352461 0000061 13		CA - Squamous cell carcinoma of vulva
5352471 0000061 18		Malignant melanoma of vulva
5352521 0000061 15		Paget disease vulvar cancer
5352541 0000061 10		Carcinoma of prostate
5352551 0000061 12		CA - Carcinoma of prostate
5352561 0000061 14		Prostate carcinoma
5352571 0000061 19		Prostate cancer
5352611 0000061 12		Carcinoma of glans penis
5352651 0000061 13		Malignant tumour of skin of penis
5352661 0000061 10		Malignant tumor of penile skin
5352671 0000061 15		Malignant tumor of skin of penis
5352681 0000061 17		Cancer of penile skin
5352691 0000061 19		Malignant tumour of penile skin
5352701 0000061 19		Carcinoma of foreskin
5352751 0000061 15		Regressed malignant testicular tumour
5352761 0000061 18		Regressed malignant testicular tumor

5352831 0000061 19		Clear cell carcinoma of kidney
5352841 0000061 12		Clear cell renal cell carcinoma
5352851 0000061 14		Cystadenocarcinoma of kidney
5352861 0000061 11		Papillary cystadenocarcinoma of kidney
5353061 0000061 13		Malignant tumour of urethral stump
5353071 0000061 18		Malignant tumor of urethral stump
5353161 0000061 12		Intracranial neoplasm
5353310 0000611 5	B80 8.11	Carcinoma in situ of biliary system
5353491 0000061 12		Pituitary carcinoma
5353721 0000061 17		Malignant tumour of olfactory tract
5353731 0000061 19		Malignant tumor of olfactory tract
5353761 0000061 11		Malignant tumour of optic nerve and sheath
5353771 0000061 16		Malignant tumor of optic nerve and sheath
5353781 0000061 18		Malignant astrocytoma of optic nerve
5353791 0000061 15		Malignant tumour of optic nerve sheath
5353801 0000061 19		Malignant tumor of optic nerve sheath
5353811 0000061 16		Malignant meningioma of optic nerve sheath
5353931 0000061 17		Malignant tumour of acoustic vestibular nerve
5353941 0000061 10		Malignant tumor of acoustic vestibular nerve

5354001 0000061 10		Malignant tumour of spinal nerve and sheath
5354011 0000061 13		Malignant tumor of spinal nerve and sheath
5354051 0000061 14		Malignant tumor of peripheral nerve
5354061 0000061 11		Peripheral nerve cancer
5354071 0000061 16		Cancer of peripheral nerve
5354081 0000061 18		Adenoid cystic carcinoma of lacrimal gland
5354091 0000061 15		Adenocarcinoma of lacrimal gland
5354101 0000061 14		Carcinoma ex pleomorphic adenoma of lacrimal gland
5354111 0000061 12		Malignant mixed tumor of lacrimal gland
5354121 0000061 16		Carcinoma in pleomorphic adenoma of lacrimal gland
5354131 0000061 18		Malignant mixed tumour of lacrimal gland
5354201 0000061 16		Malignant haemangiopericytoma of orbit
5354211 0000061 18		Malignant hemangiopericytoma of orbit
5354221 0000061 14		Malignant fibrous histiocytoma of orbit
5354361 0000061 13		Squamous cell carcinoma of conjunctiva
5354371 0000061 18		Malignant melanoma of conjunctiva
5354431 0000061 11		Squamous cell carcinoma of cornea
5354501 0000061 19		Malignant melanoma of ciliary body
5354521 0000061 12		Adenocarcinoma of pigmented epithelium of ciliary body

5354531000006110		Adenocarcinoma of non-pigmented epithelium of ciliary body
5354731000006117		Follicular thyroid carcinoma
5354741000006110		FTC - Follicular thyroid carcinoma
5354751000006112		Papillary thyroid carcinoma
5354761000006114		PTC - Papillary thyroid carcinoma
5354771000006119		Mixed follicular and papillary thyroid carcinoma
5354781000006116		Papillary thyroid carcinoma, follicular variant
5354791000006118		Follicular variant of papillary thyroid carcinoma
5354801000006117		Anaplastic thyroid carcinoma
5354811000006119		Medullary thyroid carcinoma
5354821000006110		Medullary carcinoma of thyroid
5354831000006113		MTC - Medullary thyroid carcinoma
5354861000006116		Adrenal carcinoma
5354871000006111		Adrenal cortical adenocarcinoma
5354891000006112		Parathyroid carcinoma
5355071000006117		Malignant pinealoma
5355201000006117		Malignant tumor of unknown origin
5355211000006119		Malignant tumor - unknown primary
5355221000006110		UKP - Malignant tumor - unknown primary

5355231 0000061 13		Malignant tumour - unknown primary
5355241 0000061 15		UKP - Malignant tumour - unknown primary
5355251 0000061 18		CA - Cancer of unknown origin
5355271 0000061 11		Cancer - unknown origin
5355361 0000061 14		Malignant tumour of head and neck
5355381 0000061 16		Malignant tumour of head and/or neck
5355391 0000061 18		Malignant tumor of head and/or neck
5355410 0000611 6	B80 ..00	Carcinoma in situ of digestive organ
5355601 0000061 19		Carcinoma of lip, oral cavity and/or pharynx
5355611 0000061 16		Squamous cell carcinoma of lip
5355621 0000061 12		SCC - Squamous cell carcinoma of lip
5355631 0000061 10		Malignant tumour of salivary gland
5355641 0000061 17		Cancer of salivary gland
5355651 0000061 15		CA - Cancer of salivary gland
5355661 0000061 18		Malignant tumor of salivary gland
5355671 0000061 13		Salivary gland cancer
5355681 0000061 11		Malignant tumour of ear, nose and throat
5355691 0000061 14		Malignant tumor of ear, nose and throat
5355701 0000061 14		Malignant tumour of nasal cavity and nasopharynx

5355711 0000061 12		Malignant tumor of nasal cavity and nasopharynx
5355721 0000061 16		Malignant tumour of lateral nasal wall
5355731 0000061 18		Malignant tumor of lateral nasal wall
5355751 0000061 13		Malignant tumor of digestive organ
5355761 0000061 10		Malignant tumour of oesophagus, stomach and duodenum
5355771 0000061 15		Malignant tumor of esophagus, stomach and duodenum
5355791 0000061 19		Carcinoma of cecum
5355801 0000061 18		Malignant tumour of anus and anal canal
5355811 0000061 15		Malignant tumor of anus and anal canal
5355821 0000061 11		Squamous cell carcinoma of anal margin
5355831 0000061 14		Carcinoma of anal margin
5355841 0000061 16		SCC - Squamous cell carcinoma of anal margin
5355851 0000061 19		Anal squamous cell carcinoma
5355861 0000061 17		Anal carcinoma
5355881 0000061 10		Malignant polyp of biliary tract
5355891 0000061 13		Malignant tumour of exocrine pancreas
5355901 0000061 12		Malignant tumor of exocrine pancreas
5355911 0000061 10		Pancreatic exocrine cancer
5355941 0000061 14		Malignant tumour of epidermal appendage

5355951000006111		Malignant tumor of epidermal appendage
5355961000006113		Malignant skin tumor with adnexal differentiation
5355971000006118		Malignant skin tumour with adnexal differentiation
5355981000006115		Malignant tumour of dermis
5355991000006117		Malignant tumor of dermis
5356051000006117		Malignant angioendotheliomatosis
5356061000006115		Malignant tumour of mesothelial and soft tissue
5356071000006110		Malignant tumor of connective tissue
5356081000006113		Malignant tumor of mesothelial and soft tissue
5356091000006111		Malignant tumour of connective tissue
5356111000006119		Carcinoma of bladder
5356121000006110		Transitional cell carcinoma of bladder
5356131000006113		TCC - Transitional cell carcinoma of bladder
5356141000006115		Adenocarcinoma of bladder
5356151000006118		Squamous cell carcinoma of bladder
5356161000006116		Malignant tumour of pituitary and hypothalamus
5356171000006111		Malignant tumor of pituitary and hypothalamus
5356221000006117		Secondary lymphangitic carcinoma
5356231000006119		Lymphangitis carcinomatosa

5356261000006111		Peritoneal carcinomatosis
5356291000006115		Cancer metastatic to peripheral nerve
5356331000006110		Malignant infiltration of peripheral nerve
5356341000006117		Malignant infiltration of peripheral nerve plexus
5356351000006115		Carcinoma in situ of oral cavity, lips, salivary glands
5356361000006118		Carcinoma in situ of anterior two-thirds of tongue - ventral surface
5356371000006113		Carcinoma in situ anterior 2/3 tongue ventrum
5356381000006111		Carcinoma in situ of upper labial mucosa
5356391000006114		Carcinoma in situ of mucosa of upper lip
5356401000006111		Carcinoma in situ of inner aspect of upper lip
5356411000006114		Carcinoma in situ of oral aspect of upper lip
5356421000006118		Carcinoma in situ of buccal aspect of upper lip
5356431000006115		Carcinoma in situ of lower labial mucosa
5356441000006113		Carcinoma in situ of buccal aspect of lower lip
5356451000006110		Carcinoma in situ of mucosa of lower lip
5356461000006112		Carcinoma in situ of oral aspect of lower lip
5356471000006117		Carcinoma in situ of inner aspect of lower lip
5356481000006119		Carcinoma in situ of ear, nose and throat
5356491000006116		Carcinoma in situ of middle ear and mastoid

5356501 0000061 12		Carcinoma in situ of nasal cavity and nasopharynx
5356511 0000061 10		Carcinoma in situ of respiratory and intrathoracic organ
5356561 0000061 13		Carcinoma in situ of surface epithelium
5356571 0000061 18		Carcinoma in situ of epidermal appendage
5356601 0000061 13		Carcinoma in situ of prostatic ducts
5356621 0000061 15		Carcinoma in situ of urinary tract
5357110 0000611 0	B80 830 0	Carcinoma in situ of gallbladder
5357251 0000061 12		Localised malignant reticulohistiocytoma
5357261 0000061 14		Localized malignant reticulohistiocytoma
5357310 0000611 6	B80 030 0	Carcinoma in situ of gums
5359810 0000611 8	B81 y.11	Carcinoma in situ of nasal sinuses
5360810 0000611 1	B81 y.00	Carcinoma in situ of other specified part respiratory system
5363010 0000611 7	B80 020 0	Carcinoma in situ of salivary gland
5365610 0000611 1	B82 7.11	Carcinoma in situ of skin of leg
5366410 0000611 3	B82 3.00	Carcinoma in situ of skin of other parts of face
5366510 0000611 0	B82 3z0 0	Carcinoma in situ of skin of other parts of face NOS
5367510 0000611 7	B81 yz0 0	Carcinoma in situ of specified parts respiratory system NOS
5369810 0000611 0	B83 3.00	Carcinoma in situ other and unspecified female genital organ
5369910 0000611 3	B83 6.00	Carcinoma in situ other and unspecified male genital organs

53700100000611 7	B80 7z0 0	Carcinoma in situ other and unspecified small intestine NOS
53701100000611 9	B82 2.00	Carcinoma in situ of skin of ear and external auricular canal
53702100000611 0	B82 2z0 0	Carcinoma in situ skin of ear/external auricular canal NOS
53705100000611 8	B1.. .11	Carcinoma of digestive organs and peritoneum
538094100000061 14		Hyperaldosteronism due to neoplasm of the adrenal cortex
538095100000061 11		Aldosteronism due to neoplasm of the adrenal cortex
539268100000061 11		Cancer staging
541683100000061 19		Squamous cell carcinoma antigen
541684100000061 12		SCCA - Squamous cell carcinoma antigen
54304100000611 1	B58 320 0	Cerebral metastases
54311100000611 1	B51 ..11	Cerebral tumour - malignant
54348100000611 1	B41 ..11	Malignant tumour of cervix
54377100000611 8	B83 1.13	Carcinoma in situ of uterine cervix
544132100000061 13		Occult carcinoma
544432100000061 11		Metastatic islet cell carcinoma
547735100000061 13		Malignant ACTH/MSH-secreting tumour
547736100000061 10		Malignant ACTH/MSH-secreting tumor
547737100000061 15		Malignant adrenocorticotropic hormone/melanocyte stimulating hormone-secreting tumor
547738100000061 17		Malignant adrenocorticotropic hormone/melanocyte stimulating hormone-secreting tumour

5477391000006119		Malignant pleomorphic adenocarcinoma
5477401000006117		Malignant pleomorphic carcinoma
5477411000006119		Microinvasive carcinoma
5496831000006118		History of malignant neoplasm
5511561000006114		Malignant neoplasm of digestive organs and peritoneum
5511581000006116		Malignant tumor of lesser curve of stomach
5511601000006114		Malignant tumour of greater curve of stomach
5511611000006112		Malignant tumor of greater curve of stomach
5511631000006118		Malignant tumour of middle ear and mastoid
5511641000006111		Malignant tumor of middle ear and mastoid
5511671000006115		Malignant neoplasm of soft tissue
5511681000006117		Malignant tumour of soft tissue
5511691000006119		Malignant tumor of soft tissue
5511731000006110		Malignant neoplasm of lymphatic and hemopoietic tissue
5511741000006117		Malignant tumor of lymphoid hemopoietic and related tissue
5511771000006113		Malignant tumor of lymphoid, hemopoietic AND/OR related tissue
5511991000006111		Tongue carcinoma
5512021000006115		Carcinoma of colon
5512031000006117		Carcinoma of the rectosigmoid junction

5512041<br>0000061<br>10		Cancer of the rectosigmoid junction
5512051<br>0000061<br>12		Rectosigmoid junction cancer
5512081<br>0000061<br>16		Malignant melanoma of head and neck
5512111<br>0000061<br>10		Malignant melanoma of lower limb
5523391<br>0000061<br>18		Malignant neoplasm of lip, oral cavity and/or pharynx
5523431<br>0000061<br>12		Carcinoma in situ of lip, oral cavity and/or pharynx
5526171<br>0000061<br>10		Malignant tumour of mucosa of lower lip
5526181<br>0000061<br>13		Malignant tumour of oral aspect of lower lip
5526191<br>0000061<br>11		Malignant tumour of inner aspect of lower lip
5526201<br>0000061<br>14		Malignant tumour of buccal aspect of lower lip
5526211<br>0000061<br>12		Malignant tumor of lower labial mucosa
5526221<br>0000061<br>16		Malignant tumor of buccal aspect of lower lip
5526231<br>0000061<br>18		Malignant tumor of inner aspect of lower lip
5526241<br>0000061<br>11		Malignant tumor of oral aspect of lower lip
5526251<br>0000061<br>13		Malignant tumor of mucosa of lower lip
5526610<br>0000611<br>4	B42<br>0.00	Choriocarcinoma
5531741<br>0000061<br>10		Carcinoma of base of tongue
5531761<br>0000061<br>14		Malignant melanoma of soft tissue
5541510<br>0000611<br>2	B67<br>1.00	Polycythaemia vera (clinical)

5546410 0000611 5	B65 1.11	Chronic granulocytic leukaemia
5551310 0000611 8	B64 1.11	Chronic lymphoid leukaemia, disease
5559981 0000061 16		Palate carcinoma
5559991 0000061 18		Tonsil carcinoma
5560011 0000061 14		MM - Malignant melanoma of eye
5565010 0000011 6	B33 z.11	Squamous cell carcinoma of skin NOS
5565110 0000011 9	BB2 A.1 3	[M]Squamous cell carcinoma of skin NOS
5567241 0000061 19		Borst-Jadassohn intraepidermal carcinoma
5567251 0000061 17		Clonal intraepidermal carcinoma
5567401 0000061 15		Combined hepatocellular carcinoma and cholangiocarcinoma
5567411 0000061 17		Mixed hepatocellular and bile duct carcinoma
5567421 0000061 13		Hepatocholangiocarcinoma
5567551 0000061 15		Malignant lymphoma - lymphocytic, intermediate differentiation
5571431 0000061 14		Cancer metastatic to digestive organs
5572831 0000061 19		Carcinoma ventral surface of tongue
5572841 0000061 12		Carcinoma anterior 2/3 tongue ventrum
5572851 0000061 14		Carcinoma of anterior two-thirds of tongue - dorsal surface
5572861 0000061 11		Carcinoma of midline of tongue
5572871 0000061 16		Malignant tumour of lipstick area of lip

5572881 0000061 18		Malignant tumor of lipstick area of lip
5573751 0000061 14		Carcinoma of tongue base - dorsal surface
5573781 0000061 18		Carcinoma in situ tongue base - dorsal surface
5574010 0000611 3	B83 1.11	CIN III - carcinoma in situ of cervix
5578381 0000061 10		History of carcinoma
5579691 0000061 12		Rectal neoplasm screening
5584951 0000061 17		Malignant tumour of corpus spongiosum
5584961 0000061 15		Malignant tumor of corpus spongiosum
5584971 0000061 10		Malignant tumour of corpus cavernosum
5584981 0000061 13		Malignant tumor of corpus cavernosum
5589861 0000061 12		Primary signet ring carcinoma of skin
5589991 0000061 12		Malignant tumour of skin with pilar differentiation
5590001 0000061 13		Malignant tumor of skin with pilar differentiation
5590011 0000061 11		Amelanotic malignant melanoma of skin
5590621 0000061 14		Malignant tumour of fibrous tissue
5590631 0000061 12		Malignant tumor of fibrous tissue
5590751 0000061 10		Adenocarcinoma of oesophagus
5590761 0000061 12		Adenocarcinoma of esophagus
5590771 0000061 17		Squamous cell carcinoma of oesophagus

5590781 0000061 19		Squamous cell carcinoma of esophagus
5590791 0000061 16		SCC - Squamous cell carcinoma of oesophagus
5590801 0000061 15		SCC - Squamous cell carcinoma of esophagus
5590871 0000061 14		Early gastric cancer
5590881 0000061 12		EGC - Early gastric cancer
5590891 0000061 10		Late gastric cancer
5590901 0000061 14		LGC - Late gastric cancer
5591061 0000061 17		Malignant melanoma of anus
5591071 0000061 12		Cancer anal melanoma
5591081 0000061 10		Malignant melanoma of rectum
5591091 0000061 13		Cancer rectum melanoma
5591131 0000061 10		Malignant glioma of brain
5591141 0000061 17		Malignant glioma of spinal cord
5591551 0000061 13		Squamous cell carcinoma of scrotum
5591711 0000061 17		Carcinoma of fallopian tube
5591891 0000061 16		Carcinoma of Bartholin's gland
5591901 0000061 17		Carcinoma of Bartholin gland
5592891 0000061 11		Squamous cell carcinoma of tongue
5592901 0000061 10		SCC - Squamous cell carcinoma of tongue

5592911 0000061 13		Squamous cell carcinoma of gum
5592921 0000061 17		SCC - Squamous cell carcinoma of gum
5592931 0000061 19		Squamous cell carcinoma of gingiva
5592941 0000061 12		Squamous cell carcinoma of floor of mouth
5592951 0000061 14		SCC - Squamous cell carcinoma of floor of mouth
5593071 0000061 13		Squamous cell carcinoma of palate
5593081 0000061 11		SCC - Squamous cell carcinoma of palate
5593241 0000061 10		Carcinoma of larynx
5593251 0000061 12		Laryngeal carcinoma
5595371 0000061 12		Malignant tumour of external ear
5595381 0000061 10		Malignant tumor of external ear
5600621 0000061 18		Malignant melanoma of meninges
5600821 0000061 19		Malignant white blood cell disorder
5602261 0000061 13		Nodular malignant lymphoma
5602351 0000061 17		Follicular malignant lymphoma - mixed cell type
5602361 0000061 15		Follicular malignant lymphoma - mixed small cleaved and large cell
5602371 0000061 10		Follicular malignant lymphoma - small cleaved cell
5602411 0000061 11		Diffuse malignant lymphoma - large cleaved cell
5602421 0000061 15		Diffuse malignant lymphoma - large non-cleaved cell

5602461 0000061 14		Diffuse malignant lymphoma - centroblastic polymorphic
5602571 0000061 15		Follicular malignant lymphoma - large cell
5602581 0000061 17		Malignant lymphoma - histiocytic, nodular
5602591 0000061 19		Malignant lymphoma - non-cleaved, follicular
5602911 0000061 19		Malignant lymphoma of testis
5604701 0000061 14		Malignant peritoneal local recurrence
5607311 0000061 15		Olfactory neuroendocrine carcinoma
5607891 0000061 18		Malignant teratoma of mediastinum
5607901 0000061 19		Malignant seminoma of mediastinum
5607911 0000061 16		Malignant neuroma of mediastinum
5608041 0000061 19		Malignant lymphoma of thyroid gland
5608051 0000061 17		Malignant lymphoma of breast
5608061 0000061 15		Intraductal carcinoma of breast
5608071 0000061 10		Infiltrating lobular carcinoma of breast
5608081 0000061 13		Lobular carcinoma of breast
5608091 0000061 11		Malignant Leydig cell tumour of testis
5608101 0000061 17		Malignant Leydig cell tumor of testis
5608211 0000061 13		Endometrioid carcinoma of prostate
5612941 0000061 18		Primary intra-osseous carcinoma

5613211 0000061 19		Malignant infiltration of soft tissue
5645721 0000061 12		Malignant tumour of lacrimal drainage structure
5645731 0000061 10		Malignant tumor of lacrimal drainage structure
5668581 0000061 19		Malignant bone pain
5699691 0000061 10		Squamous cell carcinoma of skin of upper lip
5699701 0000061 10		Squamous cell carcinoma of skin of lower lip
5699711 0000061 13		Squamous cell carcinoma of skin of cheek
5699721 0000061 17		Carcinoma of anal canal
5699751 0000061 14		Carcinoma of sigmoid colon
5701321 0000061 12		Carcinoma of cervix
5720811 0000061 14		Carcinoma liver and/or biliary system
5720821 0000061 18		Carcinoma liver/biliary system
5720831 0000061 15		Carcinoma of upper limb bones/scapula
5720841 0000061 13		Carcinoma of lower limb bones
5720851 0000061 10		Carcinoma of skin of head/neck
5720861 0000061 12		Carcinoma of breast - upper, inner quadrant
5720871 0000061 17		Carcinoma of breast - lower, inner quadrant
5720881 0000061 19		Carcinoma of breast - upper, outer quadrant
5720891 0000061 16		Carcinoma breast - lower, outer quadrant

5720901 0000061 17		Carcinoma of breast - axillary tail
5720911 0000061 19		Carcinoma of genital organ
5720921 0000061 10		Carcinoma genital organs
5720931 0000061 13		Carcinoma of epididymis/spermatic cord
5720941 0000061 15		Secondary carcinoma of gastrointestinal tract
5735010 0000611 7	686 4.11	Colon neoplasm screening
5736110 0000611 8	B13 z.11	Colonic cancer
5869801 0000061 12		Malignant frozen pelvis
5872010 0000611 5	B62 7.00	Non-Hodgkin's lymphoma
5873861 0000061 17		Excision of choroid neoplasm
5873951 0000061 11		Excision of basal cell carcinoma
5873961 0000061 13		Cryotherapy of basal cell carcinoma
5874081 0000061 17		Excision of skin carcinoma
5887531 0000061 12		Disseminated squamous cell carcinoma
5887541 0000061 19		Transitional cell carcinoma of ureter
5888221 0000061 10		Disseminated adenocarcinoma
5898351 0000061 11		Adenocarcinoma of sigmoid colon
5900061 0000061 17		Excision of malignant skin tumor
5911401 0000061 16		Malignant tumour of fraenum of lip

5911411 0000061 18		Malignant tumor of frenum of lip
5911421 0000061 14		Malignant tumour of soft tissue of head, face and neck
5911431 0000061 12		Malignant tumor of soft tissue of head, face and neck
5911441 0000061 19		Malignant tumour of unknown origin or ill-defined site
5911451 0000061 17		Malignant tumor of unknown origin or ill-defined site
5911531 0000061 11		Basal cell carcinoma - sclerosing type
5911541 0000061 18		Basal cell carcinoma, sclerosing type
5911951 0000061 11		Malignant lymphoma - small lymphocytic
5911961 0000061 13		Malignant lymphoma, lymphocytic, well differentiated
5911991 0000061 17		Malignant lymphoma - small cell
5912011 0000061 17		Diffuse malignant lymphoma - centroblastic
5912041 0000061 18		Malignant lymphoma, centroblastic type
5912081 0000061 12		Nodular malignant lymphoma, lymphocytic - well differentiated
5912121 0000061 14		Nodular malignant lymphoma, lymphocytic - intermediate differentiation
5912201 0000061 14		Malignant synovioma
5914081 0000061 16		Malignant tumour of posterior wall of hypopharynx
5914091 0000061 18		Malignant tumor of posterior wall of hypopharynx
5914101 0000061 12		Malignant lymphoma, convoluted cell type
5914251 0000061 15		Malignant lymphoma, follicular centre cell

5914261 0000061 18		Malignant lymphoma, follicular center cell
5914271 0000061 13		Malignant lymphoma, follicular centre cell, cleaved
5914281 0000061 11		Malignant lymphoma, follicular center cell, cleaved
5914291 0000061 14		Malignant lymphoma, follicular centre cell, non-cleaved
5914301 0000061 10		Malignant lymphoma, follicular center cell, non-cleaved
5930621 0000061 14		Adenocarcinoma of ileum
5965731 0000061 17		Perforated carcinoma of oesophagus
5965741 0000061 10		Perforated carcinoma of esophagus
5965851 0000061 18		Metastatic adenocarcinoma of unknown origin
5969251 0000061 15		Squamous cell carcinoma of mouth
5970321 0000061 19		Sebaceous adenocarcinoma
5970331 0000061 16		Sebaceous carcinoma
5970341 0000061 14		Carcinoma of sebaceous gland
5970371 0000061 18		Malignant blue naevus of skin
5970381 0000061 15		Malignant blue nevus of skin
5970391 0000061 17		Malignant melanoma in blue naevus
5970401 0000061 15		Malignant melanoma in blue nevus
5970481 0000061 12		Pilomatrix carcinoma of skin
5970661 0000061 17		Malignant lymphoma - lymphoplasmacytic

5970671000006112		Malignant lymphoma, lymphoplasmacytoid type
5970701000006113		Diffuse malignant lymphoma - centroblastic-centrocytic
5970721000006115		Malignant lymphoma - centrocytic
5970861000006116		Malignant lymphoma, mixed lymphocytic-histiocytic, nodular
5970891000006112		Malignant lymphoma, centroblastic-centrocytic, follicular
5970991000006116		Malignant lymphoma, lymphocytic, poorly differentiated, nodular
5971001000006115		Malignant lymphoma, centroblastic type, follicular
5972081000006119		Anemia in ovarian carcinoma
5986491000006117		Adenocarcinoma of uterus
5991921000006118		Occult carcinoma - stage
6001051000006117		Malignant tumour of soft tissue of back
6001061000006115		Malignant tumor of soft tissue of back
6012841000006119		Cholangiocarcinoma
6012851000006117		Cholangiocarcinoma of biliary tract
6012931000006119		Carcinoma of ascending colon
6012941000006112		Carcinoma of transverse colon
6012951000006114		Carcinoma of descending colon
6012961000006111		Carcinoma of hepatic flexure
6012971000006116		Carcinoma of splenic flexure

6023381000006117		Carcinoma-associated retinopathy
6023531000006112		Retinal pigment epithelial adenocarcinoma
6029141000006110		Squamous cell carcinoma of bronchus in left lower lobe
6029151000006112		Squamous cell carcinoma of bronchus in left upper lobe
6029161000006114		Squamous cell carcinoma of bronchus in right lower lobe
6029171000006119		Squamous cell carcinoma of bronchus in right middle lobe
6029181000006116		Squamous cell carcinoma of bronchus in right upper lobe
6043581000006110		Cystadenocarcinoma of ovary
6051531000006112		Malignant skin appendage tumour
6051541000006119		Malignant skin appendage tumor
6051841000006117		Local recurrence of malignant tumour of tongue
6051851000006115		Local recurrence of malignant tumor of tongue
6051861000006118		Local recurrence of malignant tumour of buccal cavity
6051871000006113		Local recurrence of malignant tumor of buccal cavity
6051881000006111		Local recurrence of malignant tumour of thyroid gland
6051891000006114		Local recurrence of malignant tumor of thyroid gland
6051901000006113		Local recurrence of malignant tumour of lung
6051911000006111		Local recurrence of malignant tumor of lung
6051931000006117		Local recurrence of malignant tumor of breast

6051991 0000061 18		Local recurrence of malignant tumour of oesophagus
6052001 0000061 16		Local recurrence of malignant tumor of esophagus
6052011 0000061 18		Local recurrence of malignant tumour of stomach
6052021 0000061 14		Local recurrence of malignant tumor of stomach
6052031 0000061 12		Local recurrence of malignant tumour of gallbladder
6052041 0000061 19		Local recurrence of malignant tumor of gallbladder
6052051 0000061 17		Local recurrence of malignant tumour of liver
6052061 0000061 15		Local recurrence of malignant tumor of liver
6052071 0000061 10		Local recurrence of malignant tumour of pancreas
6052081 0000061 13		Local recurrence of malignant tumor of pancreas
6052091 0000061 11		Local recurrence of malignant tumour of colon
6052101 0000061 17		Local recurrence of malignant tumor of colon
6052111 0000061 19		Local recurrence of malignant tumour of rectum
6052121 0000061 10		Local recurrence of malignant tumor of rectum
6052131 0000061 13		Local recurrence of malignant tumour of kidney
6052141 0000061 15		Local recurrence of malignant tumor of kidney
6052161 0000061 16		Local recurrence of malignant tumor of urinary bladder
6052171 0000061 11		Local recurrence of malignant tumour of prostate
6052181 0000061 14		Local recurrence of malignant tumor of prostate

6052191 0000061 12		Local recurrence of malignant tumour of cervix
6052201 0000061 10		Local recurrence of malignant tumor of cervix
6052231 0000061 19		Local recurrence of malignant tumour of bone
6052241 0000061 12		Local recurrence of malignant tumor of bone
6052251 0000061 14		Local recurrence of malignant tumour of soft tissue
6052261 0000061 11		Local recurrence of malignant tumor of soft tissue
6052271 0000061 16		Local recurrence of malignant tumour of skin
6052281 0000061 18		Local recurrence of malignant tumor of skin
6052291 0000061 15		Local recurrence of malignant melanoma of skin
6052381 0000061 11		Metastasis from malignant melanoma of skin
6052391 0000061 14		Metastasis from malignant tumour of skin
6052401 0000061 11		Metastasis from malignant tumor of skin
6052411 0000061 14		Metastasis from malignant tumour of soft tissues
6052421 0000061 18		Metastasis from malignant tumor of soft tissues
6052431 0000061 15		Metastasis from malignant tumour of bone
6052441 0000061 13		Metastasis from malignant tumor of bone
6052451 0000061 10		Metastasis from malignant tumour of adrenal gland
6052461 0000061 12		Metastasis from malignant tumor of adrenal gland
6052471 0000061 17		Metastasis from malignant tumour of cervix

6052481 0000061 19		Metastasis from malignant tumor of cervix
6052491 0000061 16		Metastasis from malignant tumour of uterus
6052501 0000061 12		Metastasis from malignant tumor of uterus
6052511 0000061 10		Metastasis from malignant tumour of prostate
6052521 0000061 19		Metastasis from malignant tumor of prostate
6052531 0000061 16		Cancer of the prostate with metastasis
6052541 0000061 14		Metastatic prostate cancer
6052551 0000061 11		Metastasis from malignant tumour of bladder
6052561 0000061 13		Metastasis from malignant tumor of bladder
6052571 0000061 18		Metastasis from malignant tumour of kidney
6052581 0000061 15		Metastasis from malignant tumor of kidney
6052591 0000061 17		Metastasis from malignant tumour of rectum
6052601 0000061 13		Metastasis from malignant tumor of rectum
6052611 0000061 11		Metastasis from malignant tumour of colon
6052621 0000061 15		Metastasis from malignant tumor of colon
6052631 0000061 17		Metastasis from malignant tumour of pancreas
6052641 0000061 10		Metastasis from malignant tumor of pancreas
6052651 0000061 12		Metastasis from malignant tumour of liver
6052661 0000061 14		Metastasis from malignant tumor of liver

6052671 0000061 19		Metastasis from malignant tumour of gallbladder
6052681 0000061 16		Metastasis from malignant tumor of gallbladder
6052691 0000061 18		Metastasis from malignant tumour of stomach
6052701 0000061 18		Metastasis from malignant tumor of stomach
6052711 0000061 15		Metastasis from malignant tumour of oesophagus
6052721 0000061 11		Metastasis from malignant tumor of esophagus
6052731 0000061 14		Metastasis from malignant tumour of breast
6052741 0000061 16		Metastasis from malignant tumor of breast
6052751 0000061 19		Metastasis from malignant tumour of bronchus
6052761 0000061 17		Metastasis from malignant tumor of bronchus
6052771 0000061 12		Metastasis from malignant tumour of lung
6052781 0000061 10		Metastasis from malignant tumor of lung
6052791 0000061 13		Metastasis from malignant tumour of thyroid
6052801 0000061 14		Metastasis from malignant tumor of thyroid
6052811 0000061 12		Metastasis from malignant tumour of buccal cavity
6052821 0000061 16		Metastasis from malignant tumor of buccal cavity
6052831 0000061 18		Metastasis from malignant tumour of tongue
6052841 0000061 11		Metastasis from malignant tumor of tongue
6053471 0000061 10		HNPCC - hereditary nonpolyposis colon cancer

6053491 0000061 11		HNPCC - hereditary nonpolyposis colorectal cancer
6131001 0000061 18		Carcinoma of head of pancreas
6206231 0000061 16		Metastatic malignant neoplasm to lateral axillary lymph nodes
6206241 0000061 14		Secondary malignant neoplasm of lateral axillary lymph nodes
6206251 0000061 11		Secondary malignant neoplasm of brachial lymph nodes
6206261 0000061 13		Metastatic malignant neoplasm to brachial lymph nodes
6206281 0000061 15		Metastatic malignant neoplasm to apex of urinary bladder
6206291 0000061 17		Secondary malignant neoplasm of apex of urinary bladder
6206311 0000061 18		Metastatic malignant neoplasm to dome of urinary bladder
6206321 0000061 14		Secondary malignant neoplasm of dome of urinary bladder
6210410 0000611 6	B62 750 0	Diffuse non-Hodgkin's mixed small and large cell (diffuse) lymphoma
6210710 0000611 2	B62 770 0	Diffuse non-Hodgkin's lymphoma, lymphoblastic (clinical)
6210910 0000611 3	B62 7X0 0	Diffuse non-Hodgkin's lymphoma, unspecified
6212051 0000061 18		Malignant soft tissue tumour of skeletal muscle differentiation
6212061 0000061 16		Malignant soft tissue tumor of skeletal muscle differentiation
6242751 0000061 14		Malignant tumour
6242761 0000061 11		Malignant tumor
6242781 0000061 18		CA - Cancer
6242811 0000061 16		Malignant tumor of lip

6242821000006112		CA - Cancer of lip
6242831000006110		Cancer of lip
6242841000006117		Malignant tumour of stomach
6242861000006118		Cancer of stomach
6242871000006113		Gastric cancer
6242881000006111		CA - Cancer of stomach
6242891000006114		Malignant tumour of caecum
6242901000006113		Malignant tumor of cecum
6242911000006111		CA - Cancer of caecum
6242921000006115		Cancer of caecum
6242941000006110		Malignant neoplasm of cecum
6242951000006112		CA - Cancer of cecum
6242961000006114		Cancer of cecum
6242971000006119		Cecal cancer
6242981000006116		Malignant tumour of rectum
6243001000006117		Rectal cancer
6243011000006119		CA - Cancer of rectum
6243031000006113		Malignant tumor of anal canal
6243041000006115		Cancer of anal canal

6243051000006118		Malignant tumour of gallbladder
6243091000006112		Malignant tumor of cervix
6243101000006118		Cancer of cervix
6243111000006115		Malignant neoplasm of cervix
6243131000006114		Cancer of the uterine cervix
6243141000006116		Cervical cancer
6243151000006119		Malignant tumour of adrenal gland
6243161000006117		Malignant tumor of adrenal gland
6243171000006112		CA - Cancer of adrenal gland
6243181000006110		Cancer of adrenal gland
6243201000006111		Malignant tumour of ill-defined site
6243211000006114		Malignant tumor of ill-defined site
6243221000006118		Cancer of ill-defined site
6243231000006115		CA - Cancer of ill-defined site
6243241000006113		Malignant tumour of lung
6243261000006112		CA - Lung cancer
6243271000006117		Malignant tumour of middle ear
6243281000006119		Malignant tumor of middle ear
6243291000006116		Malignant tumour of anterior two-thirds of tongue

6243301 0000061 15		Malignant tumor of anterior two-thirds of tongue
6243311 0000061 17		Malignant tumour of soft tissue of shoulder
6243321 0000061 13		Malignant tumor of soft tissue of shoulder
6243331 0000061 11		Malignant neoplasm of soft tissue of shoulder
6243341 0000061 18		Malignant tumour of soft tissue of hip
6243351 0000061 16		Malignant tumor of soft tissue of hip
6243361 0000061 19		Malignant neoplasm of soft tissue of hip
6243371 0000061 14		Malignant tumour of soft tissue of thorax
6243381 0000061 12		Malignant tumor of soft tissue of thorax
6243391 0000061 10		Malignant neoplasm of soft tissue of thorax
6243401 0000061 12		Malignant tumour of soft tissue of pelvis
6243411 0000061 10		Malignant tumor of soft tissue of pelvis
6243421 0000061 19		Malignant tumour of vulva
6243441 0000061 14		Cancer of vulva
6243451 0000061 11		Carcinoma of body of pancreas
6243461 0000061 13		Carcinoma of tail of pancreas
6243491 0000061 17		Malignant tumor of vermilion border of upper lip
6243501 0000061 13		Malignant tumour of lipstick area of upper lip
6243511 0000061 11		Malignant tumor of lipstick area of upper lip

6243541 0000061 10		Malignant tumor of vermilion border of lower lip
6243551 0000061 12		Malignant tumour of lipstick area of lower lip
6243561 0000061 14		Malignant tumor of lipstick area of lower lip
6243591 0000061 18		Malignant tumour of commissure of lip
6243601 0000061 14		Malignant tumor of commissure of lip
6243611 0000061 12		Malignant tumour of labial commissure
6243621 0000061 16		Malignant tumor of labial commissure
6243641 0000061 11		Malignant tumor of tongue
6243651 0000061 13		CA - Cancer of tongue
6243661 0000061 10		Cancer of tongue
6243671 0000061 15		Malignant tumour of base of tongue
6243691 0000061 19		Malignant tumour of posterior third of tongue
6243701 0000061 19		Malignant tumour of fixed part of tongue
6243711 0000061 16		Malignant tumour of tongue posterior to vallate papillae
6243721 0000061 12		Malignant tumor of fixed part of tongue
6243731 0000061 10		Malignant tumor of tongue posterior to vallate papillae
6243741 0000061 17		Malignant tumor of posterior third of tongue
6243761 0000061 18		Malignant tumour of lingual tonsil
6243771 0000061 13		Malignant tumor of lingual tonsil

6243791 0000061 14		Malignant tumor of major salivary gland
6243821 0000061 17		Malignant tumor of parotid gland
6243831 0000061 19		Cancer of parotid gland
6243841 0000061 12		CA - Cancer of parotid gland
6243851 0000061 14		Malignant tumour of submandibular gland
6243861 0000061 11		Malignant tumor of submandibular gland
6243881 0000061 18		Malignant tumour of sublingual gland
6243891 0000061 15		Malignant tumor of sublingual gland
6243901 0000061 16		Malignant tumour of gum
6243911 0000061 18		Malignant tumor of gum
6243921 0000061 14		Malignant tumour of alveolar mucosa
6243931 0000061 12		Malignant tumour of gingiva
6243941 0000061 19		Malignant tumor of alveolar mucosa
6243951 0000061 17		Malignant tumor of gingiva
6243961 0000061 15		Cancer of gum
6243971 0000061 10		CA - Cancer of gum
6243981 0000061 13		Malignant tumour of upper gingiva
6243991 0000061 11		Malignant tumor of upper gingiva
6244001 0000061 19		Malignant tumour of upper gum

6244011 0000061 16		Malignant tumor of upper gum
6244021 0000061 12		Malignant tumour of lower gingiva
6244031 0000061 10		Malignant tumor of lower gingiva
6244041 0000061 17		Malignant tumour of lower gum
6244051 0000061 15		Malignant tumor of lower gum
6244071 0000061 13		Malignant tumor of floor of mouth
6244081 0000061 11		FOM - Malignant tumour of floor of mouth
6244091 0000061 14		FOM - Malignant tumor of floor of mouth
6244101 0000061 15		FOM - Cancer of floor of mouth
6244111 0000061 17		Cancer of floor of mouth
6244121 0000061 13		CA - Cancer of floor of mouth
6244141 0000061 18		Malignant tumour of buccal mucosa
6244151 0000061 16		Malignant tumor of buccal mucosa
6244161 0000061 19		Malignant tumour of internal cheek
6244171 0000061 14		Malignant tumour of cheek mucosa
6244181 0000061 12		Malignant tumor of internal cheek
6244191 0000061 10		Malignant tumor of cheek mucosa
6244201 0000061 13		CA - Cancer of cheek mucosa
6244211 0000061 11		CA - Cancer of buccal mucosa

6244221 0000061 15		Cancer of cheek mucosa
6244231 0000061 17		Cancer of buccal mucosa
6244241 0000061 10		Malignant tumour of hard palate
6244251 0000061 12		Malignant tumor of hard palate
6244261 0000061 14		Malignant tumour of soft palate
6244271 0000061 19		Malignant tumor of soft palate
6244281 0000061 16		Malignant tumour of uvula
6244291 0000061 18		Malignant tumor of uvula
6244311 0000061 19		Malignant tumor of palate
6244321 0000061 10		Malignant tumour of roof of mouth
6244331 0000061 13		Malignant tumor of roof of mouth
6244351 0000061 18		Cancer of palate
6244361 0000061 16		CA - Cancer of palate
6244371 0000061 11		Malignant tumour of retromolar area
6244381 0000061 14		Malignant tumor of retromolar area
6244401 0000061 14		Malignant tumor of oropharynx
6244411 0000061 12		Cancer of oropharynx
6244421 0000061 16		CA - Cancer of oropharynx
6244431 0000061 18		Malignant tumour of mesopharynx

6244441 0000061 11		Malignant tumor of mesopharynx
6244451 0000061 13		Malignant tumour of tonsil
6244471 0000061 15		Malignant tumour of palatine tonsil
6244481 0000061 17		Malignant tumour of faucial tonsil
6244491 0000061 19		Malignant tumor of faucial tonsil
6244501 0000061 10		Malignant tumor of palatine tonsil
6244511 0000061 13		Cancer of tonsil
6244541 0000061 12		CA - Cancer of tonsil
6244561 0000061 11		Malignant tumor of tonsillar fossa
6244571 0000061 16		Malignant tumour of vallecula
6244581 0000061 18		Malignant tumor of vallecula
6244591 0000061 15		Malignant tumour of branchial cleft
6244601 0000061 11		Malignant tumor of branchial cleft
6244611 0000061 14		Malignant tumour of roof of nasopharynx
6244621 0000061 18		Malignant tumor of roof of nasopharynx
6244631 0000061 15		Malignant tumour of superior wall of nasopharynx
6244641 0000061 13		Malignant tumor of superior wall of nasopharynx
6244671 0000061 17		Malignant tumor of lateral wall of nasopharynx
6244691 0000061 16		Malignant tumor of hypopharynx

6244701 0000061 16		Malignant tumour of laryngopharynx
6244711 0000061 18		Malignant tumor of laryngopharynx
6244731 0000061 12		Malignant tumour of postcricoid region
6244741 0000061 19		Malignant tumor of postcricoid region
6244751 0000061 17		Malignant tumour of pharyngo-oesophageal junction
6244761 0000061 15		Malignant tumor of pharyngoesophageal junction
6244771 0000061 10		Malignant tumour of pyriform fossa
6244781 0000061 13		Malignant tumor of pyriform fossa
6244791 0000061 11		Malignant tumour of pyriform sinus
6244801 0000061 12		Malignant tumor of pyriform sinus
6244811 0000061 10		Malignant tumour of oesophagus
6244821 0000061 19		Malignant tumor of esophagus
6244831 0000061 16		Cancer of oesophagus
6244851 0000061 11		CA - Cancer of oesophagus
6244871 0000061 18		Cancer of esophagus
6244881 0000061 15		CA - Cancer of esophagus
6244891 0000061 17		Esophageal cancer
6244901 0000061 18		Malignant neoplasm of esophagus
6244911 0000061 15		Malignant tumour of duodenum

6244931<br>0000061<br>14		Duodenal cancer
6244941<br>0000061<br>16		Malignant tumour of jejunum
6244951<br>0000061<br>19		Malignant tumor of jejunum
6244971<br>0000061<br>12		Malignant tumor of ileum
6244991<br>0000061<br>13		Malignant tumor of colon
6245001<br>0000061<br>18		CA - Cancer of colon
6245011<br>0000061<br>15		Cancer of colon
6245031<br>0000061<br>14		Malignant tumour of hepatic flexure
6245061<br>0000061<br>17		Hepatic flexure colon cancer
6245081<br>0000061<br>10		Malignant tumor of transverse colon
6245101<br>0000061<br>19		Transverse colon cancer
6245111<br>0000061<br>16		Malignant tumour of descending colon
6245131<br>0000061<br>10		Descending colon cancer
6245151<br>0000061<br>15		Malignant tumor of sigmoid colon
6245161<br>0000061<br>18		Sigmoid colon cancer
6245171<br>0000061<br>13		Malignant tumour of appendix
6245181<br>0000061<br>11		Malignant tumor of appendix
6245201<br>0000061<br>12		Malignant tumor of ascending colon
6245211<br>0000061<br>10		Ascending colon cancer

6245221 0000061 19		Malignant tumour of splenic flexure
6245231 0000061 16		Malignant tumor of splenic flexure
6245251 0000061 11		Splenic flexure colon cancer
6245271 0000061 18		Malignant tumor of rectosigmoid junction
6245281 0000061 15		Rectosigmoid colon cancer
6245291 0000061 17		Malignant tumour of biliary tract
6245301 0000061 16		Malignant tumor of biliary tract
6245321 0000061 14		Malignant tumor of extrahepatic bile duct
6245341 0000061 19		Extrahepatic bile duct cancer
6245351 0000061 17		Malignant tumour of ampulla of Vater
6245361 0000061 15		Malignant tumor of ampulla of Vater
6245381 0000061 13		Malignant tumor of pancreas
6245391 0000061 11		CA - Cancer of pancreas
6245401 0000061 13		Pancreatic cancer
6245411 0000061 11		CA - Pancreatic cancer
6245421 0000061 15		Malignant tumour of head of pancreas
6245451 0000061 12		Malignant retroperitoneal tumour
6245461 0000061 14		Malignant retroperitoneal tumor
6245491 0000061 18		Cancer of omentum

6245501 0000061 14		CA - Cancer of omentum
6245511 0000061 12		Malignant tumour of nasal cavity
6245521 0000061 16		Malignant tumor of nasal cavity
6245541 0000061 11		Malignant tumour of nasal septum
6245551 0000061 13		Malignant tumor of nasal septum
6245561 0000061 10		Malignant tumour of mastoid air cells
6245571 0000061 15		Malignant tumor of mastoid air cells
6245581 0000061 17		Malignant tumour of maxillary sinus
6245591 0000061 19		Malignant tumor of maxillary sinus
6245601 0000061 10		Malignant tumour of maxillary antrum
6245611 0000061 13		Malignant tumor of maxillary antrum
6245621 0000061 17		Malignant tumour of ethmoid sinus
6245631 0000061 19		Malignant tumor of ethmoid sinus
6245651 0000061 14		Malignant tumour of frontal sinus
6245661 0000061 11		Malignant tumor of frontal sinus
6245671 0000061 16		Malignant tumour of sphenoid sinus
6245681 0000061 18		Malignant tumor of sphenoid sinus
6245701 0000061 15		Malignant tumor of larynx
6245711 0000061 17		CA - Cancer of larynx

6245721000006113		Laryngeal cancer
6245731000006111		Cancer of larynx
6245741000006118		Malignant tumour of subglottis
6245751000006116		Malignant tumor of subglottis
6245781000006112		Malignant tumor of laryngeal cartilage
6245791000006110		Malignant tumour of trachea
6245801000006111		Malignant tumor of trachea
6245821000006118		CA - Cancer of trachea
6245831000006115		Malignant tracheal tumor
6245841000006113		Malignant tracheal tumour
6245861000006112		Malignant tumor of pleura
6245871000006117		Cancer of pleura
6245881000006119		CA - Cancer of pleura
6245891000006116		Malignant tumour of thymus
6245901000006117		Malignant tumor of thymus
6245921000006110		Malignant tumor of heart
6245931000006113		Malignant tumour of endocardium
6245941000006115		Malignant tumor of endocardium
6245951000006118		Malignant tumour of myocardium

6245961 0000061 16		Malignant tumor of myocardium
6245971 0000061 11		Malignant tumour of vertebral column
6245981 0000061 14		Malignant tumor of vertebral column
6245991 0000061 12		CA - Cancer of spine
6246001 0000061 13		Cancer of spine
6246011 0000061 11		Malignant neoplasm of spine
6246021 0000061 15		Malignant tumour of soft tissue of head
6246031 0000061 17		Malignant tumor of soft tissue of head
6246051 0000061 12		Malignant tumour of soft tissue of face
6246061 0000061 14		Malignant tumor of soft tissue of face
6246081 0000061 16		Malignant tumour of soft tissue of neck
6246091 0000061 18		Malignant tumor of soft tissue of neck
6246121 0000061 19		Malignant tumor of ovary
6246131 0000061 16		CA - Cancer of ovary
6246151 0000061 11		Ovarian cancer
6246161 0000061 13		Malignant tumour of fallopian tube
6246171 0000061 18		Malignant tumor of fallopian tube
6246191 0000061 17		Malignant tumor of vagina
6246201 0000061 19		Cancer of vagina

6246241 0000061 17		Malignant tumor of testis
6246251 0000061 15		Malignant tumour of foreskin
6246261 0000061 18		Malignant tumor of foreskin
6246271 0000061 13		Malignant tumour of prepuce
6246281 0000061 11		Malignant tumor of prepuce
6246301 0000061 10		Cancer of foreskin
6246311 0000061 13		Malignant neoplasm of foreskin
6246321 0000061 17		Malignant tumour of glans penis
6246331 0000061 19		Malignant tumor of glans penis
6246341 0000061 12		Cancer of glans penis
6246351 0000061 14		Malignant tumour of epididymis
6246361 0000061 11		Malignant tumor of epididymis
6246371 0000061 16		Malignant tumour of spermatic cord
6246381 0000061 18		Malignant tumor of spermatic cord
6246401 0000061 18		Malignant tumour of scrotum
6246411 0000061 15		Malignant tumor of scrotum
6246421 0000061 11		Malignant scrotal tumour
6246431 0000061 14		Malignant scrotal tumor
6246441 0000061 16		CA - Cancer of scrotum

6246451 0000061 19		Cancer of scrotum
6246471 0000061 12		Malignant tumour of urachus
6246481 0000061 10		Malignant tumor of urachus
6246501 0000061 17		Malignant tumor of renal pelvis
6246511 0000061 19		Malignant tumour of ureter
6246531 0000061 13		Cancer of ureter
6246551 0000061 18		Malignant tumour of urethra
6246561 0000061 16		Malignant tumor of urethra
6246571 0000061 11		Malignant urethral tumour
6246581 0000061 14		Malignant urethral tumor
6246601 0000061 16		CA - Cancer of urethra
6246611 0000061 18		Malignant tumour of paraurethral gland
6246621 0000061 14		Malignant tumor of paraurethral gland
6246631 0000061 12		Malignant neoplasm of paraurethral gland
6246651 0000061 17		Malignant tumour of eye
6246661 0000061 15		Malignant tumor of eye
6246671 0000061 10		Malignant tumour of orbit
6246681 0000061 13		Malignant tumor of orbit
6246691 0000061 11		Malignant orbital tumour

6246701 0000061 11		Malignant orbital tumor
6246711 0000061 14		Malignant tumour of conjunctiva
6246721 0000061 18		Malignant tumor of conjunctiva
6246731 0000061 15		Malignant tumour of cornea
6246741 0000061 13		Malignant tumor of cornea
6246751 0000061 10		Malignant tumour of retina
6246761 0000061 12		Malignant tumor of retina
6246771 0000061 17		Malignant retinal tumour
6246781 0000061 19		Malignant retinal tumor
6246791 0000061 16		Malignant tumour of choroid
6246801 0000061 15		Malignant tumor of choroid
6246881 0000061 12		Cancer of the cerebral meninges
6246891 0000061 10		Malignant tumour of spinal cord
6246901 0000061 14		Malignant tumor of spinal cord
6246931 0000061 18		Malignant tumour of thyroid gland
6246951 0000061 13		Thyroid cancer
6246971 0000061 15		Malignant tumour of parathyroid gland
6246981 0000061 17		Malignant tumor of parathyroid gland
6246991 0000061 19		Parathyroid cancer

6247001 0000061 10		Malignant tumour of pituitary gland
6247011 0000061 13		Malignant tumor of pituitary gland
6247021 0000061 17		Cancer of pituitary gland
6247031 0000061 19		CA - Cancer of pituitary gland
6247041 0000061 12		Pituitary cancer
6247051 0000061 14		Malignant tumour of pineal gland
6247061 0000061 11		Malignant tumor of pineal gland
6247071 0000061 16		Pineal cancer
6247081 0000061 18		Cancer of pineal gland
6247091 0000061 15		Malignant tumour of pelvis
6247101 0000061 14		Malignant tumor of pelvis
6247111 0000061 12		Malignant tumour of minor salivary gland
6247121 0000061 16		Malignant tumor of minor salivary gland
6247131 0000061 18		Malignant tumour of vocal cord
6247141 0000061 11		Malignant tumor of vocal cord
6247151 0000061 13		Malignant tumour of vocal fold
6247161 0000061 10		Malignant tumor of vocal fold
6247171 0000061 15		CA - Cancer of vocal cord
6247181 0000061 17		Cancer of vocal cord

6247191000006119		Malignant tumour of aryepiglottic fold - laryngeal aspect
6247201000006116		Malignant tumor of aryepiglottic fold - laryngeal aspect
6247211000006118		Malignant neoplasm of laryngeal aspect of interarytenoid fold
6247221000006114		Malignant tumour of false cord
6247231000006112		Malignant tumor of false cord
6247241000006119		Malignant tumour of ventricular band
6247251000006117		Malignant tumour of vestibular fold
6247261000006115		Malignant tumor of ventricular band
6247271000006110		Malignant tumor of vestibular fold
6247291000006111		Malignant tumor of neck
6247301000006112		Malignant tumour of anus
6247311000006110		Malignant tumor of anus
6247321000006119		Malignant tumour of cloacogenic zone
6247331000006116		Malignant tumor of cloacogenic zone
6247341000006114		Malignant tumour of peritoneum
6247351000006111		Malignant tumor of peritoneum
6247361000006113		Peritoneal cancer
6247371000006118		CA - Cancer of peritoneum
6247381000006115		Cancer of peritoneum

6247391 0000061 17		Malignant tumour of bronchus
6247401 0000061 15		Malignant tumor of bronchus
6247411 0000061 17		CA - Cancer of bronchus
6247421 0000061 13		Cancer of bronchus
6247431 0000061 11		Malignant tumour of mediastinum
6247451 0000061 16		Malignant neoplasm of mediastinum
6247461 0000061 19		Malignant mediastinal tumor
6247471 0000061 14		Malignant mediastinal tumour
6247481 0000061 12		Malignant tumour of muscle
6247491 0000061 10		Malignant tumor of muscle
6247501 0000061 19		Cancer of muscle
6247511 0000061 16		CA - Cancer of muscle
6247521 0000061 12		Malignant tumour of soft tissue of abdomen
6247531 0000061 10		Malignant tumor of soft tissue of abdomen
6247551 0000061 15		Malignant tumor of meninges
6247561 0000061 18		Malignant tumour of optic nerve
6247571 0000061 13		Malignant tumor of optic nerve
6247591 0000061 14		Malignant tumor of spleen
6247611 0000061 15		Multiple cancer

6247621<br>0000061<br>11		CA - Multiple cancer
6247631<br>0000061<br>14		Malignant tumour of face
6247641<br>0000061<br>16		Malignant tumor of face
6247661<br>0000061<br>17		Malignant tumor of axilla
6247671<br>0000061<br>12		Malignant tumour of upper limb
6247681<br>0000061<br>10		Malignant tumor of upper limb
6247701<br>0000061<br>13		Malignant tumor of lower limb
6247711<br>0000061<br>11		Malignant tumour of oral cavity
6247721<br>0000061<br>15		Malignant tumor of oral cavity
6247731<br>0000061<br>17		Malignant tumour of mouth
6247741<br>0000061<br>10		Malignant tumor of mouth
6247751<br>0000061<br>12		Cancer of oral cavity
6247761<br>0000061<br>14		CA - Mouth cancer
6247771<br>0000061<br>19		Mouth cancer
6247781<br>0000061<br>16		Malignant neoplasm of mouth
6247791<br>0000061<br>18		Malignant tumour of nasal sinuses
6247801<br>0000061<br>17		Malignant tumor of nasal sinuses
6247811<br>0000061<br>19		Malignant tumour of pharynx
6247821<br>0000061<br>10		Malignant tumor of pharynx

6247831 0000061 13		Cancer of pharynx
6247841 0000061 15		CA - Cancer of pharynx
6247851 0000061 18		Malignant neoplasm of pharynx
6247871 0000061 11		Malignant tumor of intestine
6247881 0000061 14		Cancer of intestine
6247891 0000061 12		Malignant neoplasm of intestine
6247901 0000061 11		CA - Cancer of intestine
6247911 0000061 14		Intestinal cancer
6247931 0000061 15		CA - Cancer of bowel
6247941 0000061 13		Malignant tumour of small intestine
6247961 0000061 12		Malignant tumour of small bowel
6247971 0000061 17		Malignant tumor of small bowel
6247981 0000061 19		Malignant tumour of large intestine
6247991 0000061 16		Malignant tumor of large intestine
6248001 0000061 14		Cancer of large intestine
6248011 0000061 12		Cancer of large bowel
6248021 0000061 16		CA - Cancer of large bowel
6248031 0000061 18		Colorectal cancer
6248051 0000061 13		Malignant tumor of female genital organ

6248061 0000061 10		Malignant tumour of male genital organ
6248091 0000061 19		Malignant tumor of penis
6248101 0000061 13		Cancer of penis
6248131 0000061 17		CA - Cancer of penis
6248141 0000061 10		Malignant tumour of urinary tract proper
6248151 0000061 12		Malignant tumor of urinary tract
6248161 0000061 14		Malignant tumour of urinary tract
6248171 0000061 19		Malignant tumor of urinary tract proper
6248191 0000061 18		Malignant tumor of kidney
6248201 0000061 15		Renal malignant tumour
6248211 0000061 17		Renal malignant tumor
6248221 0000061 13		CA - Renal cancer
6248231 0000061 11		CA - Cancer of kidney
6248241 0000061 18		Renal cancer
6249091 0000061 10		Hurthle cell neoplasm
6249101 0000061 16		Follicular neoplasm
6250441 0000061 17		Primary malignant neoplasm of gastrointestinal tract
6298041 0000061 17		Infantile malignant osteopetrosis
6300131 0000061 18		Malignant Neoplasm (Morphology)

6300141 0000061 11		Malignant neoplasm of primary, secondary, or uncertain origin
6324541 0000061 16		Malignant tumour involving rectum by direct extension from endometrium
6324551 0000061 19		Malignant tumor involving rectum by direct extension from endometrium
6324561 0000061 17		Malignant tumour involving rectum by direct extension from fallopian tube
6324571 0000061 12		Malignant tumor involving rectum by direct extension from fallopian tube
6324581 0000061 10		Malignant tumour involving rectum by direct extension from ovary
6324591 0000061 13		Malignant tumor involving rectum by direct extension from ovary
6324601 0000061 17		Malignant tumour involving rectum by direct extension from prostate
6324611 0000061 19		Malignant tumor involving rectum by direct extension from prostate
6324621 0000061 10		Malignant tumour involving rectum by direct extension from uterine cervix
6324631 0000061 13		Malignant tumor involving rectum by direct extension from uterine cervix
6324641 0000061 15		Malignant tumour involving rectum by direct extension from uterus
6324651 0000061 18		Malignant tumor involving rectum by direct extension from uterus
6324661 0000061 16		Malignant tumour involving rectum by direct extension from vagina
6324671 0000061 11		Malignant tumor involving rectum by direct extension from vagina
6324681 0000061 14		Malignant tumour involving rectum by separate metastasis from endometrium
6324691 0000061 12		Malignant tumor involving rectum by separate metastasis from endometrium
6324701 0000061 12		Malignant tumour involving rectum by separate metastasis from fallopian tube
6324711 0000061 10		Malignant tumor involving rectum by separate metastasis from fallopian tube

6324721000006119		Malignant tumour involving rectum by separate metastasis from ovary
6324731000006116		Malignant tumor involving rectum by separate metastasis from ovary
6324741000006114		Malignant tumour involving rectum by separate metastasis from prostate
6324751000006111		Malignant tumor involving rectum by separate metastasis from prostate
6324761000006113		Malignant tumour involving rectum by separate metastasis from uterine cervix
6324771000006118		Malignant tumor involving rectum by separate metastasis from uterine cervix
6324781000006115		Malignant tumour involving rectum by separate metastasis from uterus
6324791000006117		Malignant tumor involving rectum by separate metastasis from uterus
6324801000006116		Malignant tumour involving rectum by separate metastasis from vagina
6324811000006118		Malignant tumor involving rectum by separate metastasis from vagina
6324831000006112		Malignant tumour involving ureter by direct extension from bladder
6324841000006119		Malignant tumor involving ureter by direct extension from bladder
6324851000006117		Malignant tumour involving ureter by separate metastasis from bladder
6324861000006115		Malignant tumor involving ureter by separate metastasis from bladder
6324871000006110		Malignant tumour involving urethra by direct extension from bladder
6324881000006113		Malignant tumor involving urethra by direct extension from bladder
6324891000006111		Malignant tumour involving urethra by direct extension from prostate
6324901000006110		Malignant tumor involving urethra by direct extension from prostate
6324911000006113		Malignant tumour involving urethra by separate metastasis from bladder

6324921 0000061 17		Malignant tumor involving urethra by separate metastasis from bladder
6324931 0000061 19		Malignant tumour involving urethra by separate metastasis from prostate
6324941 0000061 12		Malignant tumor involving urethra by separate metastasis from prostate
6324951 0000061 14		Malignant tumour involving bladder by direct extension from endometrium
6324961 0000061 11		Malignant tumor involving bladder by direct extension from endometrium
6324971 0000061 16		Malignant tumour involving bladder by direct extension from fallopian tube
6324981 0000061 18		Malignant tumor involving bladder by direct extension from fallopian tube
6324991 0000061 15		Malignant tumour involving bladder by direct extension from ovary
6325001 0000061 10		Malignant tumor involving bladder by direct extension from ovary
6325011 0000061 13		Malignant tumour involving bladder by direct extension from prostate
6325021 0000061 17		Malignant tumor involving bladder by direct extension from prostate
6325031 0000061 19		Malignant tumour involving bladder by direct extension from uterine cervix
6325041 0000061 12		Malignant tumor involving bladder by direct extension from uterine cervix
6325051 0000061 14		Malignant tumour involving bladder by direct extension from uterus
6325061 0000061 11		Malignant tumor involving bladder by direct extension from uterus
6325071 0000061 16		Malignant tumour involving bladder by direct extension from vagina
6325081 0000061 18		Malignant tumor involving bladder by direct extension from vagina
6325091 0000061 15		Malignant tumour involving bladder by separate metastasis from endometrium
6325101 0000061 14		Malignant tumor involving bladder by separate metastasis from endometrium

6325111 0000061 12		Malignant tumour involving bladder by separate metastasis from fallopian tube
6325121 0000061 16		Malignant tumor involving bladder by separate metastasis from fallopian tube
6325131 0000061 18		Malignant tumour involving bladder by separate metastasis from ovary
6325141 0000061 11		Malignant tumor involving bladder by separate metastasis from ovary
6325151 0000061 13		Malignant tumour involving bladder by separate metastasis from prostate
6325161 0000061 10		Malignant tumor involving bladder by separate metastasis from prostate
6325171 0000061 15		Malignant tumour involving bladder by separate metastasis from uterine cervix
6325181 0000061 17		Malignant tumor involving bladder by separate metastasis from uterine cervix
6325191 0000061 19		Malignant tumour involving bladder by separate metastasis from uterus
6325201 0000061 16		Malignant tumor involving bladder by separate metastasis from uterus
6325211 0000061 18		Malignant tumour involving bladder by separate metastasis from vagina
6325221 0000061 14		Malignant tumor involving bladder by separate metastasis from vagina
6325231 0000061 12		Malignant tumour involving vasa deferentia by direct extension from prostate
6325241 0000061 19		Malignant tumor involving vasa deferentia by direct extension from prostate
6325251 0000061 17		Malignant tumour involving vasa deferentia by separate metastasis from prostate
6325261 0000061 15		Malignant tumor involving vasa deferentia by separate metastasis from prostate
6325271 0000061 10		Malignant tumour involving prostate by direct extension from bladder
6325281 0000061 13		Malignant tumor involving prostate by direct extension from bladder
6325291 0000061 11		Malignant tumour involving prostate by separate metastasis from bladder

6325301000006112		Malignant tumor involving prostate by separate metastasis from bladder
6325311000006110		Primary malignant neoplasm of seminal vesicle
6325321000006119		Secondary malignant neoplasm of seminal vesicle
6325331000006116		Malignant tumour involving seminal vesicle by direct extension from bladder
6325341000006114		Malignant tumor involving seminal vesicle by direct extension from bladder
6325351000006111		Malignant tumour involving seminal vesicle by direct extension from prostate
6325361000006113		Malignant tumor involving seminal vesicle by direct extension from prostate
6325371000006118		Malignant tumour involving seminal vesicle by separate metastasis from bladder
6325381000006115		Malignant tumor involving seminal vesicle by separate metastasis from bladder
6325391000006117		Malignant tumour involving seminal vesicle by separate metastasis from prostate
6325401000006115		Malignant tumor involving seminal vesicle by separate metastasis from prostate
6325411000006117		Malignant tumour involving uterine corpus by direct extension from bladder
6325421000006113		Malignant tumor involving uterine corpus by direct extension from bladder
6325431000006111		Malignant tumour involving uterine corpus by direct extension from ovary
6325441000006118		Malignant tumor involving uterine corpus by direct extension from ovary
6325451000006116		Malignant tumour involving uterine corpus by direct extension from uterine cervix
6325461000006119		Malignant tumor involving uterine corpus by direct extension from uterine cervix
6325471000006114		Malignant tumour involving uterine corpus by direct extension from vagina
6325481000006112		Malignant tumor involving uterine corpus by direct extension from vagina

6325491 0000061 10		Malignant tumour involving uterine cervix by direct extension from fallopian tube
6325501 0000061 19		Malignant tumor involving uterine cervix by direct extension from fallopian tube
6325511 0000061 16		Malignant tumour involving uterine cervix by direct extension from ovary
6325521 0000061 12		Malignant tumor involving uterine cervix by direct extension from ovary
6325531 0000061 10		Malignant tumour involving uterine cervix by direct extension from vagina
6325541 0000061 17		Malignant tumor involving uterine cervix by direct extension from vagina
6325551 0000061 15		Malignant tumour involving uterine cervix by separate metastasis from fallopian tube
6325561 0000061 18		Malignant tumor involving uterine cervix by separate metastasis from fallopian tube
6325571 0000061 13		Malignant tumour involving uterine cervix by separate metastasis from ovary
6325581 0000061 11		Malignant tumor involving uterine cervix by separate metastasis from ovary
6325591 0000061 14		Malignant tumour involving uterine corpus by separate metastasis from bladder
6325601 0000061 18		Malignant tumor involving uterine corpus by separate metastasis from bladder
6325611 0000061 15		Malignant tumour involving vagina by direct extension from uterus
6325621 0000061 11		Malignant tumor involving vagina by direct extension from uterus
6325631 0000061 14		Malignant tumour involving vagina by direct extension from bladder
6325641 0000061 16		Malignant tumor involving vagina by direct extension from bladder
6325651 0000061 19		Malignant tumour involving vagina by direct extension from endometrium
6325661 0000061 17		Malignant tumor involving vagina by direct extension from endometrium
6325671 0000061 12		Malignant tumour involving vagina by direct extension from fallopian tube

6325681000006110		Malignant tumor involving vagina by direct extension from fallopian tube
6325691000006113		Malignant tumour involving vulva by direct extension from endometrium
6325701000006113		Malignant tumor involving vulva by direct extension from endometrium
6325711000006111		Malignant tumour involving vulva by direct extension from fallopian tube
6325721000006115		Malignant tumor involving vulva by direct extension from fallopian tube
6325731000006117		Malignant tumour involving vulva by direct extension from ovary
6325741000006110		Malignant tumor involving vulva by direct extension from ovary
6325751000006112		Malignant tumour involving vulva by direct extension from uterine cervix
6325761000006114		Malignant tumor involving vulva by direct extension from uterine cervix
6325771000006119		Malignant tumour involving vulva by direct extension from uterus
6325781000006116		Malignant tumor involving vulva by direct extension from uterus
6325791000006118		Malignant tumour involving vagina by direct extension from ovary
6325801000006117		Malignant tumor involving vagina by direct extension from ovary
6325811000006119		Primary malignant neoplasm of left fallopian tube
6325821000006110		Secondary malignant neoplasm of left fallopian tube
6325831000006113		Malignant tumour involving left fallopian tube by direct extension from endometrium
6325841000006115		Malignant tumor involving left fallopian tube by direct extension from endometrium
6325851000006118		Malignant tumour involving left fallopian tube by direct extension from ovary
6325861000006116		Malignant tumor involving left fallopian tube by direct extension from ovary

6325871 0000061 11		Malignant tumour involving left fallopian tube by direct extension from right fallopian tube
6325881 0000061 14		Malignant tumor involving left fallopian tube by direct extension from right fallopian tube
6325891 0000061 12		Malignant tumour involving left fallopian tube by direct extension from uterine cervix
6325901 0000061 11		Malignant tumor involving left fallopian tube by direct extension from uterine cervix
6325911 0000061 14		Malignant tumour involving left fallopian tube by direct extension from uterus
6325921 0000061 18		Malignant tumor involving left fallopian tube by direct extension from uterus
6325931 0000061 15		Primary malignant neoplasm of right fallopian tube
6325941 0000061 13		Secondary malignant neoplasm of right fallopian tube
6325951 0000061 10		Primary malignant neoplasm of left ovary
6325961 0000061 12		Secondary malignant neoplasm of left ovary
6325971 0000061 17		Cancer metastatic to left ovary
6325981 0000061 19		Malignant tumour involving left ovary by direct extension from endometrium
6325991 0000061 16		Malignant tumor involving left ovary by direct extension from endometrium
6326001 0000061 15		Malignant tumour involving left ovary by direct extension from fallopian tube
6326011 0000061 17		Malignant tumor involving left ovary by direct extension from fallopian tube
6326021 0000061 13		Malignant tumour involving left ovary by direct extension from right ovary
6326031 0000061 11		Malignant tumor involving left ovary by direct extension from right ovary
6326041 0000061 18		Malignant tumour involving left ovary by direct extension from uterine cervix
6326051 0000061 16		Malignant tumor involving left ovary by direct extension from uterine cervix

6326061 0000061 19		Malignant tumour involving left ovary by direct extension from uterus
6326071 0000061 14		Malignant tumor involving left ovary by direct extension from uterus
6326081 0000061 12		Primary malignant neoplasm of right ovary
6326091 0000061 10		Secondary malignant neoplasm of right ovary
6326101 0000061 16		Cancer metastatic to right ovary
6326111 0000061 18		Malignant tumour involving right ovary by direct extension from endometrium
6326121 0000061 14		Malignant tumor involving right ovary by direct extension from endometrium
6326131 0000061 12		Malignant tumour involving right ovary by direct extension from fallopian tube
6326141 0000061 19		Malignant tumor involving right ovary by direct extension from fallopian tube
6326151 0000061 17		Malignant tumour involving right ovary by direct extension from left ovary
6326161 0000061 15		Malignant tumor involving right ovary by direct extension from left ovary
6326171 0000061 10		Malignant tumour involving right ovary by direct extension from uterine cervix
6326181 0000061 13		Malignant tumor involving right ovary by direct extension from uterine cervix
6326191 0000061 11		Secondary neoplasm of left broad ligament
6326201 0000061 14		Secondary neoplasm of right broad ligament
6326211 0000061 12		Malignant tumour involving left broad ligament by direct extension from ovary
6326221 0000061 16		Malignant tumor involving left broad ligament by direct extension from ovary
6326231 0000061 18		Malignant tumour involving left broad ligament by metastasis from ovary
6326241 0000061 11		Malignant tumor involving left broad ligament by metastasis from ovary

6326251 0000061 13		Malignant tumour involving right broad ligament by direct extension from ovary
6326261 0000061 10		Malignant tumor involving right broad ligament by direct extension from ovary
6326271 0000061 15		Malignant tumour involving right broad ligament by metastasis from ovary
6326281 0000061 17		Malignant tumor involving right broad ligament by metastasis from ovary
6326291 0000061 19		Malignant tumour involving left fallopian tube by direct extension from vagina
6326301 0000061 18		Malignant tumor involving left fallopian tube by direct extension from vagina
6326311 0000061 15		Malignant tumour involving left fallopian tube by separate metastasis from endometrium
6326321 0000061 11		Malignant tumor involving left fallopian tube by separate metastasis from endometrium
6326331 0000061 14		Malignant tumour involving left fallopian tube by separate metastasis from ovary
6326341 0000061 16		Malignant tumor involving left fallopian tube by separate metastasis from ovary
6326351 0000061 19		Malignant tumour involving left fallopian tube by separate metastasis from right fallopian tube
6326361 0000061 17		Malignant tumor involving left fallopian tube by separate metastasis from right fallopian tube
6326371 0000061 12		Malignant tumour involving left fallopian tube by separate metastasis from uterus
6326381 0000061 10		Malignant tumor involving left fallopian tube by separate metastasis from uterus
6326391 0000061 13		Malignant tumour involving left fallopian tube by separate metastasis from vagina
6326401 0000061 10		Malignant tumor involving left fallopian tube by separate metastasis from vagina
6326411 0000061 13		Malignant tumour involving right fallopian tube by direct extension from endometrium
6326421 0000061 17		Malignant tumor involving right fallopian tube by direct extension from endometrium
6326431 0000061 19		Malignant tumour involving right fallopian tube by direct extension from left fallopian tube

6326441000006112		Malignant tumor involving right fallopian tube by direct extension from left fallopian tube
6326451000006114		Malignant tumour involving right fallopian tube by direct extension from ovary
6326461000006111		Malignant tumor involving right fallopian tube by direct extension from ovary
6326471000006116		Malignant tumour involving right fallopian tube by direct extension from uterine cervix
6326481000006118		Malignant tumor involving right fallopian tube by direct extension from uterine cervix
6326491000006115		Malignant tumour involving right fallopian tube by direct extension from uterus
6326501000006111		Malignant tumor involving right fallopian tube by direct extension from uterus
6326511000006114		Malignant tumour involving right fallopian tube by direct extension from vagina
6326521000006118		Malignant tumor involving right fallopian tube by direct extension from vagina
6326531000006115		Malignant tumour involving right fallopian tube by separate metastasis from endometrium
6326541000006113		Malignant tumor involving right fallopian tube by separate metastasis from endometrium
6326551000006110		Malignant tumour involving right fallopian tube by separate metastasis from left fallopian tube
6326561000006112		Malignant tumor involving right fallopian tube by separate metastasis from left fallopian tube
6326571000006117		Malignant tumour involving right fallopian tube by separate metastasis from ovary
6326581000006119		Malignant tumor involving right fallopian tube by separate metastasis from ovary
6326591000006116		Malignant tumour involving right fallopian tube by separate metastasis from uterine cervix
6326601000006112		Malignant tumor involving right fallopian tube by separate metastasis from uterine cervix
6326611000006110		Malignant tumour involving right fallopian tube by separate metastasis from uterus
6326621000006119		Malignant tumor involving right fallopian tube by separate metastasis from uterus

6326631<br>0000061<br>16		Malignant tumour involving right fallopian tube by separate metastasis from vagina
6326641<br>0000061<br>14		Malignant tumor involving right fallopian tube by separate metastasis from vagina
6326651<br>0000061<br>11		Malignant tumour involving left ovary by direct extension from vagina
6326661<br>0000061<br>13		Malignant tumor involving left ovary by direct extension from vagina
6326671<br>0000061<br>18		Malignant tumour involving left ovary by separate metastasis from endometrium
6326681<br>0000061<br>15		Malignant tumor involving left ovary by separate metastasis from endometrium
6326691<br>0000061<br>17		Malignant tumour involving left ovary by separate metastasis from fallopian tube
6326701<br>0000061<br>17		Malignant tumor involving left ovary by separate metastasis from fallopian tube
6326711<br>0000061<br>19		Malignant tumour involving left ovary by separate metastasis from right ovary
6326721<br>0000061<br>10		Malignant tumor involving left ovary by separate metastasis from right ovary
6326731<br>0000061<br>13		Malignant tumour involving left ovary by separate metastasis from uterine cervix
6326741<br>0000061<br>15		Malignant tumor involving left ovary by separate metastasis from uterine cervix
6326751<br>0000061<br>18		Malignant tumour involving left ovary by separate metastasis from vagina
6326761<br>0000061<br>16		Malignant tumor involving left ovary by separate metastasis from vagina
6326771<br>0000061<br>11		Malignant tumour involving left ovary by separate metastasis uterus
6326781<br>0000061<br>14		Malignant tumor involving left ovary by separate metastasis uterus
6326791<br>0000061<br>12		Malignant tumour involving right ovary by direct extension from uterus
6326801<br>0000061<br>13		Malignant tumor involving right ovary by direct extension from uterus
6326811<br>0000061<br>11		Malignant tumour involving right ovary by direct extension from vagina

6326821000006115		Malignant tumor involving right ovary by direct extension from vagina
6326831000006117		Malignant tumour involving right ovary by separate metastasis from endometrium
6326841000006110		Malignant tumor involving right ovary by separate metastasis from endometrium
6326851000006112		Malignant tumour involving right ovary by separate metastasis from fallopian tube
6326861000006114		Malignant tumor involving right ovary by separate metastasis from fallopian tube
6326871000006119		Malignant tumour involving right ovary by separate metastasis from left ovary
6326881000006116		Malignant tumor involving right ovary by separate metastasis from left ovary
6326891000006118		Malignant tumour involving right ovary by separate metastasis from uterine cervix
6326901000006119		Malignant tumor involving right ovary by separate metastasis from uterine cervix
6326911000006116		Malignant tumour involving right ovary by separate metastasis from uterus
6326921000006112		Malignant tumor involving right ovary by separate metastasis from uterus
6326931000006110		Malignant tumour involving right ovary by separate metastasis from vagina
6326941000006117		Malignant tumor involving right ovary by separate metastasis from vagina
6326951000006115		Malignant tumour involving uterine cervix by separate metastasis from vagina
6326961000006118		Malignant tumor involving uterine cervix by separate metastasis from vagina
6326971000006113		Malignant tumour involving uterine corpus by separate metastasis from fallopian tube
6326981000006111		Malignant tumor involving uterine corpus by separate metastasis from fallopian tube
6326991000006114		Malignant tumour involving uterine corpus by separate metastasis from ovary
6327001000006118		Malignant tumor involving uterine corpus by separate metastasis from ovary

6327011 0000061 15		Malignant tumour involving uterine corpus by separate metastasis from uterine cervix
6327021 0000061 11		Malignant tumor involving uterine corpus by separate metastasis from uterine cervix
6327031 0000061 14		Malignant tumour involving uterine corpus by separate metastasis from vagina
6327041 0000061 16		Malignant tumor involving uterine corpus by separate metastasis from vagina
6327051 0000061 19		Malignant tumour involving uterine corpus by direct extension from fallopian tube
6327061 0000061 17		Malignant tumor involving uterine corpus by direct extension from fallopian tube
6327071 0000061 12		Malignant tumour involving vagina by direct extension from uterine cervix
6327081 0000061 10		Malignant tumor involving vagina by direct extension from uterine cervix
6327091 0000061 13		Malignant tumour involving vagina by separate metastasis from bladder
6327101 0000061 19		Malignant tumor involving vagina by separate metastasis from bladder
6327111 0000061 16		Malignant tumour involving vagina by separate metastasis from endometrium
6327121 0000061 12		Malignant tumor involving vagina by separate metastasis from endometrium
6327131 0000061 10		Malignant tumour involving vagina by separate metastasis from fallopian tube
6327141 0000061 17		Malignant tumor involving vagina by separate metastasis from fallopian tube
6327151 0000061 15		Malignant tumour involving vagina by separate metastasis from ovary
6327161 0000061 18		Malignant tumor involving vagina by separate metastasis from ovary
6327171 0000061 13		Malignant tumour involving vagina by separate metastasis from uterine cervix
6327181 0000061 11		Malignant tumor involving vagina by separate metastasis from uterine cervix
6327191 0000061 14		Malignant tumour involving vagina by separate metastasis from uterus

6327201000006112		Malignant tumor involving vagina by separate metastasis from uterus
6327211000006110		Malignant tumour involving vulva by direct extension from vagina
6327221000006119		Malignant tumor involving vulva by direct extension from vagina
6327231000006116		Malignant tumour involving vulva by separate metastasis from endometrium
6327241000006114		Malignant tumor involving vulva by separate metastasis from endometrium
6327251000006111		Malignant tumour involving vulva by separate metastasis from fallopian tube
6327261000006113		Malignant tumor involving vulva by separate metastasis from fallopian tube
6327271000006118		Malignant tumour involving vulva by separate metastasis from ovary
6327281000006115		Malignant tumor involving vulva by separate metastasis from ovary
6327291000006117		Malignant tumour involving vulva by separate metastasis from uterine cervix
6327301000006116		Malignant tumor involving vulva by separate metastasis from uterine cervix
6327311000006118		Malignant tumour involving vulva by separate metastasis from uterus
6327321000006114		Malignant tumor involving vulva by separate metastasis from uterus
6327331000006112		Malignant tumour involving vulva by separate metastasis from vagina
6327341000006119		Malignant tumor involving vulva by separate metastasis from vagina
6327351000006117		Malignant tumour involving an organ by direct extension from bladder
6327361000006115		Malignant tumor involving an organ by direct extension from bladder
6327371000006110		Malignant tumour involving an organ by direct extension from endometrium
6327381000006113		Malignant tumor involving an organ by direct extension from endometrium

6327391000006111		Malignant tumour involving an organ by direct extension from fallopian tube
6327401000006113		Malignant tumor involving an organ by direct extension from fallopian tube
6327411000006111		Malignant tumour involving an organ by direct extension from ovary
6327421000006115		Malignant tumor involving an organ by direct extension from ovary
6327431000006117		Malignant tumour involving an organ by direct extension from prostate
6327441000006110		Malignant tumor involving an organ by direct extension from prostate
6327451000006112		Malignant tumour involving an organ by direct extension from uterine cervix
6327461000006114		Malignant tumor involving an organ by direct extension from uterine cervix
6327471000006119		Malignant tumour involving an organ by direct extension from uterus
6327481000006116		Malignant tumor involving an organ by direct extension from uterus
6327491000006118		Malignant tumour involving an organ by direct extension from vagina
6327501000006114		Malignant tumor involving an organ by direct extension from vagina
6327511000006112		Malignant tumour involving an organ by separate metastasis from bladder
6327521000006116		Malignant tumor involving an organ by separate metastasis from bladder
6327531000006118		Malignant tumour involving an organ by separate metastasis from endometrium
6327541000006111		Malignant tumor involving an organ by separate metastasis from endometrium
6327551000006113		Malignant tumour involving an organ by separate metastasis from fallopian tube
6327561000006110		Malignant tumor involving an organ by separate metastasis from fallopian tube
6327571000006115		Malignant tumour involving an organ by separate metastasis from ovary

6327581 0000061 17		Malignant tumor involving an organ by separate metastasis from ovary
6327591 0000061 19		Malignant tumour involving an organ by separate metastasis from prostate
6327601 0000061 10		Malignant tumor involving an organ by separate metastasis from prostate
6327611 0000061 13		Malignant tumour involving an organ by separate metastasis from uterine cervix
6327621 0000061 17		Malignant tumor involving an organ by separate metastasis from uterine cervix
6327631 0000061 19		Malignant tumour involving an organ by separate metastasis from uterus
6327641 0000061 12		Malignant tumor involving an organ by separate metastasis from uterus
6327651 0000061 14		Malignant tumour involving an organ by separate metastasis from vagina
6327661 0000061 11		Malignant tumor involving an organ by separate metastasis from vagina
6327671 0000061 16		Malignant tumour involving left fallopian tube by separate metastasis from uterine cervix
6327681 0000061 18		Malignant tumor involving left fallopian tube by separate metastasis from uterine cervix
6331211 0000061 18		Ductal carcinoma in situ nuclear pleomorphism, grade 1: monotonous nuclei, 1.5 - 2.0 red blood cells diameters, with finely dispersed chromatin and only occasional nucleoli
6331231 0000061 12		Ductal carcinoma in situ nuclear pleomorphism, grade 2: neither nuclear grade 1 nor nuclear grade 3
6331251 0000061 17		Ductal carcinoma in situ nuclear pleomorphism, grade 3: Markedly pleomorphic nuclei, usually >2.5 red blood cells diameters, with coarse chromatin and multiple nucleoli
6331461 0000061 19		T1 (I): Cervical carcinoma confined to uterus
6331471 0000061 14		T1a (IA): Invasive carcinoma of uterine cervix diagnosed by microscopy only
6331841 0000061 15		T3 (III): Endometrial tumour with local and/or regional spread as specified in T3a, b, N1 and FIGO IIIA, B, and C below or adnexa (direct extension or metastasis) and/or cancer cells in ascites or peritoneal washings
6331851 0000061 18		T3 (III): Endometrial tumor with local and/or regional spread as specified in T3a, b, N1 and FIGO IIIA, B, and C below or adnexa (direct extension or metastasis) and/or cancer cells in ascites or peritoneal washings
6332021 0000061 12		T1c (IC): Tumour limited to one or both tube(s) with extension into or through the tubal serosa; or with malignant cells in ascites or peritoneal washings: FALLOPIAN TUBE: Salpingectomy

6332031 0000061 10		T1c (IC): Tumor limited to one or both tube(s) with extension into or through the tubal serosa; or with malignant cells in ascites or peritoneal washings: FALLOPIAN TUBE: Salpingectomy
6332101 0000061 15		T2c (IIC): Fallopian tube/ovarian tumour with pelvic extension (T2a or b) with malignant cells in ascites or peritoneal washings
6332111 0000061 17		T2c (IIC): Fallopian tube/ovarian tumor with pelvic extension (T2a or b) with malignant cells in ascites or peritoneal washings
6333321 0000061 18		T4: Lung tumour of any size with a malignant pleural effusion
6333331 0000061 15		T4: Lung tumor of any size with a malignant pleural effusion
6333741 0000061 14		T4d: Inflammatory carcinoma (breast)
6333751 0000061 11		Tis: Carcinoma in situ, breast: Intraductal carcinoma, lobular carcinoma in situ, or Paget's disease of the nipple with no tumour
6333761 0000061 13		Tis: Carcinoma in situ, breast: Intraductal carcinoma, lobular carcinoma in situ, or Paget's disease of the nipple with no tumor
6333771 0000061 18		Tis: Carcinoma in situ, breast: Intraductal carcinoma, lobular carcinoma in situ, or Paget disease of the nipple with no tumour
6333781 0000061 15		Tis: Carcinoma in situ, breast: Intraductal carcinoma, lobular carcinoma in situ, or Paget disease of the nipple with no tumor
6334091 0000061 17		Tis (0): Carcinoma in situ (vagina)
6334101 0000061 11		Tis (0): Carcinoma in situ (limited to fallopian tube mucosa)
6334111 0000061 14		Tis: Epithelium only (carcinoma in situ, no invasion)
6334281 0000061 10		Tis: Carcinoma in situ ('flat tumor' of urinary bladder)
6334291 0000061 13		Tis: Carcinoma in situ ('flat tumour' of urinary bladder)
6334581 0000061 13		Ta: Noninvasive papillary carcinoma (urinary tract)
6334591 0000061 11		Tis: Intratubular germ cell neoplasia (carcinoma in situ, testis)
6335041 0000061 10		T1c (IC): Tumour limited to one or both ovaries with any of the following: capsule ruptured, tumour on ovarian surface, malignant cells in ascites or peritoneal washings: OVARY: Resection
6335051 0000061 12		T1c (IC): Tumor limited to one or both ovaries with any of the following: capsule ruptured, tumor on ovarian surface, malignant cells in ascites or peritoneal washings: OVARY: Resection

6336641000006112		Choriocarcinoma, metastatic
6343391000006115		Excision of malignant neoplasm
6349601000006118		Malignant lymphoma, large cell, polymorphous, immunoblastic
6362141000006113		Primary malignant neoplasm of abdominal oesophagus
6362151000006110		Primary malignant neoplasm of abdominal esophagus
6362161000006112		Primary malignant neoplasm of adrenal cortex
6362181000006119		Malignant tumour of adrenal cortex
6362191000006116		Malignant tumor of adrenal cortex
6362211000006115		Malignant tumour of adrenal medulla
6362221000006111		Malignant tumor of adrenal medulla
6362231000006114		Primary malignant neoplasm of adrenal medulla
6362241000006116		Primary malignant neoplasm of ampulla of Vater
6362251000006119		Primary malignant neoplasm of anterior two-thirds of tongue
6362261000006117		Primary malignant neoplasm of apex of urinary bladder
6362271000006112		Primary malignant neoplasm of biliary tract
6362281000006110		Primary malignant neoplasm of body of uterus
6362301000006114		Malignant tumour of body of uterus
6362311000006112		Malignant tumor of body of uterus
6362341000006111		Malignant tumour of uterus

6362351 0000061 13		Malignant tumor of uterus
6362361 0000061 10		CA - Cancer of uterus
6362371 0000061 15		Cancer of uterus
6362381 0000061 17		Uterine cancer
6362391 0000061 19		Malignant neoplasm of border of tongue
6362401 0000061 17		Malignant neoplasm of tip AND/OR lateral border of tongue
6362411 0000061 19		Primary malignant neoplasm of border of tongue
6362421 0000061 10		Primary malignant neoplasm of buccal mucosa
6362431 0000061 13		Primary malignant neoplasm of caecum
6362441 0000061 15		Primary malignant neoplasm of cecum
6362451 0000061 18		Primary malignant neoplasm of cervical oesophagus
6362461 0000061 16		Primary malignant neoplasm of cervical esophagus
6362491 0000061 12		Primary malignant neoplasm of clitoris
6362501 0000061 16		Cancer of clitoris
6362511 0000061 18		Primary malignant neoplasm of commissure of lip
6362531 0000061 12		Malignant tumour of endocrine gland
6362541 0000061 19		Malignant tumor of endocrine gland
6362551 0000061 17		Primary malignant neoplasm of endocrine gland
6362561 0000061 15		Primary malignant neoplasm of oesophagus

6362571 0000061 10		Primary malignant neoplasm of esophagus
6362581 0000061 13		Primary malignant neoplasm of eye
6362591 0000061 11		Primary malignant neoplasm of fallopian tube
6362601 0000061 15		Primary malignant neoplasm of false vocal cord
6362611 0000061 17		Primary malignant neoplasm of glans penis
6362621 0000061 13		Primary malignant neoplasm of gum
6362631 0000061 11		Primary malignant neoplasm of hard palate
6362641 0000061 18		Primary malignant neoplasm of intestinal tract
6362651 0000061 16		Primary malignant neoplasm of lacrimal gland
6362661 0000061 19		Primary malignant neoplasm of laryngeal aspect of aryepiglottic fold
6362671 0000061 14		Primary malignant neoplasm of larynx
6362681 0000061 12		Primary malignant neoplasm of lip
6362691 0000061 10		Primary malignant neoplasm of lower gum
6362701 0000061 10		Primary malignant neoplasm of lower third of oesophagus
6362711 0000061 13		Primary malignant neoplasm of lower third of esophagus
6362721 0000061 17		Primary malignant neoplasm of middle third of oesophagus
6362731 0000061 19		Primary malignant neoplasm of middle third of esophagus
6362741 0000061 12		Primary malignant neoplasm of minor salivary gland
6362751 0000061 14		Primary malignant neoplasm of oral cavity

6362761 0000061 11		Primary malignant neoplasm of palate
6362771 0000061 16		Primary malignant neoplasm of pancreas
6362781 0000061 18		Primary malignant neoplasm of parotid gland
6362791 0000061 15		Primary malignant neoplasm of penis
6362801 0000061 19		Primary malignant neoplasm of prepuce
6362811 0000061 16		Primary malignant neoplasm of retrocaecal tissue
6362821 0000061 12		Primary malignant neoplasm of retrocecal tissue
6362831 0000061 10		Primary malignant neoplasm of scaphoid bone
6362841 0000061 17		Primary malignant neoplasm of scrotum
6362851 0000061 15		Primary malignant neoplasm of soft tissues
6362861 0000061 18		Malignant tumour of soft tissue of upper limb
6362871 0000061 13		Malignant tumor of soft tissue of upper limb
6362881 0000061 11		Primary malignant neoplasm of soft tissues of upper limb
6362891 0000061 14		Primary malignant neoplasm of spermatic cord
6362901 0000061 13		Primary malignant neoplasm of stomach
6362911 0000061 11		Primary malignant neoplasm of the mesentery
6362921 0000061 15		Primary malignant neoplasm of the peritoneum
6362931 0000061 17		Primary malignant neoplasm of thoracic oesophagus
6362941 0000061 10		Primary malignant neoplasm of thoracic esophagus

6362951<br>0000061<br>12		Malignant neoplasm of thoracic vertebral column
6362961<br>0000061<br>14		Primary malignant neoplasm of thoracic vertebral column
6362971<br>0000061<br>19		Primary malignant neoplasm of tonsil
6362981<br>0000061<br>16		Primary malignant neoplasm of trapezium
6362991<br>0000061<br>18		Primary malignant neoplasm of upper gum
6363001<br>0000061<br>17		Primary malignant neoplasm of upper third of oesophagus
6363011<br>0000061<br>19		Primary malignant neoplasm of upper third of esophagus
6363021<br>0000061<br>10		Primary malignant neoplasm of uterine cervix
6363031<br>0000061<br>13		Primary malignant neoplasm of vagina
6363041<br>0000061<br>15		Primary malignant neoplasm of vermilion border of lower lip
6363051<br>0000061<br>18		Primary malignant neoplasm of vermilion border of upper lip
6363061<br>0000061<br>16		Primary malignant neoplasm of vertebral column
6363081<br>0000061<br>14		Primary malignant neoplasm of vocal cord
6363601<br>0000061<br>14		Malignant tumour of nervous system
6363611<br>0000061<br>12		Malignant tumor of nervous system
6363631<br>0000061<br>18		Female breast cancer
6363641<br>0000061<br>11		Cancer of female breast
6363951<br>0000061<br>17		Primary malignant neoplasm
6363981<br>0000061<br>13		Primary malignant neoplasm of axillary tail of breast

6363991 0000061 11		Secondary malignant neoplasm of axillary tail of breast
6364001 0000061 19		Malignant neoplasm of axillary tail of breast
6364041 0000061 17		Carcinoma of male breast
6364071 0000061 13		Carcinoma of endocervix
6364091 0000061 14		Cancer of exocervix
6364101 0000061 15		Carcinoma of exocervix
6364111 0000061 17		Carcinoma of extrahepatic bile duct
6364171 0000061 14		Carcinoma of glottis
6364181 0000061 12		Carcinoma of subglottis
6364191 0000061 10		Carcinoma of supraglottis
6364201 0000061 13		Carcinoma of penis
6364211 0000061 11		Primary malignant neoplasm of ribs and/or sternum and/or clavicle
6364221 0000061 15		Primary malignant neoplasm of ribs, sternum and clavicle
6364231 0000061 17		Malignant neoplasm of bone of lower limb
6364271 0000061 19		Primary malignant neoplasm of lower lobe, bronchus or lung
6364281 0000061 16		Carcinoma of lower lobe, bronchus or lung
6364291 0000061 18		Primary malignant neoplasm of middle lobe, bronchus or lung
6364301 0000061 17		Carcinoma of middle lobe, bronchus or lung
6364341 0000061 15		Primary malignant neoplasm of pelvic bones, sacrum and coccyx

6364351 0000061 18		Carcinoma of pelvic bones, sacrum and coccyx
6364361 0000061 16		Primary malignant neoplasm of head of pancreas
6364371 0000061 11		Carcinoma of main bronchus
6364381 0000061 14		Carcinoma of ribs and/or sternum and/or clavicle
6364391 0000061 12		Carcinoma of ribs, sternum and/or clavicle
6364401 0000061 14		Malignant neoplasm of skin head and neck
6364421 0000061 16		Primary malignant neoplasm of skin head and neck
6364431 0000061 18		Malignant neoplasm of skin of lower limb
6364451 0000061 13		Carcinoma of skin of lower limb
6364481 0000061 17		Carcinoma of skin of trunk
6364491 0000061 19		Malignant neoplasm of skin of upper limb
6364511 0000061 13		Carcinoma of skin of upper limb
6364531 0000061 19		Skin cancer
6364541 0000061 12		Cancer of skin
6364551 0000061 14		Malignant neoplasm of upper limb bones and scapula
6364611 0000061 14		Primary malignant neoplasm of upper limb bones and scapula
6364641 0000061 13		Primary malignant neoplasm of upper lobe, bronchus or lung
6364651 0000061 10		Carcinoma of upper lobe, bronchus or lung
6364661 0000061 12		Primary malignant neoplasm of breast

6364671000006117		Carcinoma of oesophagus
6364681000006119		Carcinoma of esophagus
6364691000006116		Primary malignant neoplasm of gallbladder
6364711000006118		Carcinoma of vocal cord
6364721000006114		Carcinoma of pancreas
6364731000006112		Carcinoma of stomach
6364741000006119		Gastric carcinoma
6364881000006115		Malignant melanoma - category
6364891000006117		Malignant melanocytic lesion
6364901000006118		Malignant melanoma, metastatic
6365751000006116		Malignant melanoma
6376181000006114		Malignant neoplasm of breast lower inner quadrant
6376191000006112		Malignant neoplasm of breast lower outer quadrant
6376201000006110		Malignant neoplasm of breast upper inner quadrant
6376211000006113		Malignant neoplasm of breast upper outer quadrant
6376261000006111		Primary malignant neoplasm of breast upper outer quadrant
6376271000006116		Primary malignant neoplasm of breast upper inner quadrant
6376281000006118		Primary malignant neoplasm of breast lower inner quadrant
6376291000006115		Primary malignant neoplasm of breast lower outer quadrant

6377211 0000061 11		pN3b: Tumor of breast with metastasis as per American Joint Committee on Cancer 6th Edition definition (breast)
6377221 0000061 15		pN3b: Tumour of breast with metastasis as per American Joint Committee on Cancer 6th Edition definition (breast)
6377421 0000061 16		pTis: Carcinoma in situ (breast)
6377431 0000061 18		pTis: Ductal carcinoma in situ (breast)
6377441 0000061 11		Ductal carcinoma in situ (breast)
6377451 0000061 13		pTis: Lobular carcinoma in situ (breast)
6377461 0000061 10		pTis: Paget disease without invasive carcinoma (breast)
6377491 0000061 19		pTis: Paget's disease without invasive carcinoma
6377821 0000061 19		pT4d: Inflammatory carcinoma (breast)
6377991 0000061 13		pTis: Carcinoma in situ, invasion of lamina propria
6380471 0000061 13		Invasive ductal carcinoma with an extensive intraductal component
6383441 0000061 12		Bronchioloalveolar carcinoma - disorder
6383451 0000061 14		Bronchiolar adenocarcinoma
6383461 0000061 11		Bronchiolar carcinoma
6383471 0000061 16		Alveolar cell carcinoma
6383481 0000061 18		Bronchioloalveolar carcinoma
6383491 0000061 15		Bronchoalveolar cancer
6385281 0000061 18		Cancer diagnosis based on death certificate
6385291 0000061 15		Cancer diagnosis based on clinical evidence

6385301000006119		Cancer diagnosis based on investigations, without a tissue diagnosis
6385311000006116		Cancer diagnosis based on specific tumour markers
6385321000006112		Cancer diagnosis based on specific tumor markers
6385331000006110		Cancer diagnosis based on cytological evidence
6385341000006117		Cancer diagnosis based on metastatic histological evidence
6385351000006115		Cancer diagnosis based on primary site histological evidence
6385701000006118		No anti-cancer treatment - advanced stage cancer
6386541000006118		Extrauterine adenocarcinoma
63872100000113	B118.00	Siewert type II adenocarcinoma
63882100000118	B119.00	Siewert type III adenocarcinoma
63884100000113	B107.00	Siewert type I adenocarcinoma
64130100000114	F205.00	Malignant multiple sclerosis
6425911000006115		Surgical proximal margin uninvolved by malignant neoplasm
6425921000006111		Surgical proximal margin involved by malignant neoplasm
6425931000006114		Surgical proximal margin uninvolved by in situ carcinoma
6425941000006116		Surgical proximal margin involved by in situ carcinoma
6425991000006113		Surgical circumferential margin involved by malignant neoplasm (tumor present 0-1 mm from CRM)
6426001000006114		Surgical circumferential margin involved by malignant neoplasm (tumor present 0-1 mm from circumferential resection margin)
6426011000006112		Surgical circumferential margin involved by malignant neoplasm (tumour present 0-1 mm from CRM)

6426021 0000061 16		Surgical circumferential margin uninvolved by malignant neoplasm
6426031 0000061 18		Surgical distal margin involved by malignant neoplasm
6426041 0000061 11		Surgical distal margin uninvolved by malignant neoplasm
6426261 0000061 19		Surgical proximal margin uninvolved by in situ carcinoma/adenoma
6426271 0000061 14		Surgical proximal margin involved by in situ carcinoma/adenoma
6426281 0000061 12		Surgical distal margin involved by in situ carcinoma/adenoma
6426291 0000061 10		Surgical distal margin uninvolved by in situ carcinoma/adenoma
6427121 0000061 17		Surgical margin uninvolved by ductal carcinoma in situ
6427141 0000061 12		Surgical margin involved by ductal carcinoma in situ
6427161 0000061 11		Surgical margin involved by malignant neoplasm
6427171 0000061 16		Surgical margin uninvolved by malignant neoplasm
6427291 0000061 12		Surgical margin involved by malignant neoplasm, unifocal
6427301 0000061 13		Surgical margin involved by malignant neoplasm, multifocal
6427311 0000061 11		Surgical margin involved by malignant neoplasm, extensive
6427341 0000061 10		Surgical margin involved by ductal carcinoma in situ, unifocal
6427371 0000061 19		Surgical margin involved by ductal carcinoma in situ, multifocal
6427391 0000061 18		Surgical margin involved by ductal carcinoma in situ, extensive
6428141 0000061 11		pT2: Tumor of lung as per American Joint Committee on Cancer 6th Edition definition (lung)
6428151 0000061 13		pT2: Tumour of lung as per American Joint Committee on Cancer 6th Edition definition (lung)

6428201000006116		pT4: Tumor of lung as per American Joint Committee on Cancer 6th Edition definition (lung)
6428211000006118		pT4: Tumour of lung as per American Joint Committee on Cancer 6th Edition definition (lung)
6429441000006113		Surgical lateral (mucosal/mural) margin involved by in situ carcinoma
6429481000006119		Surgical lateral (mucosal/mural) margin involved by malignant neoplasm
6429491000006116		Surgical lateral (mucosal/mural) margin uninvolved by in situ carcinoma
6429531000006116		Surgical lateral (mucosal/mural) margin uninvolved by malignant neoplasm
6431441000006111		Carcinoma with pleomorphic, sarcomatoid or sarcomatous elements
6431451000006113		Carcinoma of salivary gland type
6431491000006119		Surgical bronchial margin involved by malignant neoplasm
6431501000006110		Surgical vascular margin involved by malignant neoplasm
6431511000006113		Surgical parenchymal margin involved by malignant neoplasm
6431521000006117		Surgical parietal pleural margin involved by malignant neoplasm
6431531000006119		Surgical chest wall margin involved by malignant neoplasm
6431541000006112		Surgical margin site involved by malignant neoplasm
6431551000006114		Surgical attached tissue margin site involved by malignant neoplasm
6431561000006111		Site involved by direct extension of malignant neoplasm
6431571000006116		Chest wall involved by direct extension of malignant neoplasm
6431581000006118		Diaphragm involved by direct extension of malignant neoplasm
6431591000006115		Mediastinal pleura involved by direct extension of malignant neoplasm

6431601000006111		Visceral pleura involved by direct extension of malignant neoplasm
6431611000006114		Parietal pericardium involved by direct extension of malignant neoplasm
6431621000006118		Mediastinum involved by direct extension of malignant neoplasm
6431631000006115		Heart involved by direct extension of malignant neoplasm
6431641000006113		Great vessels involved by direct extension of malignant neoplasm
6431721000006114		Main bronchus (less than 2 cm distal to the carina) involved by direct extension of malignant neoplasm
6432011000006113		Surgical apical margin involved by malignant neoplasm
6432021000006117		Surgical anterior margin involved by malignant neoplasm
6432031000006119		Surgical lateral margin involved by malignant neoplasm
6432041000006112		Surgical posterior margin involved by malignant neoplasm
6432051000006114		Surgical bladder neck margin involved by malignant neoplasm
6432061000006111		Surgical posterolateral (neurovascular bundle) margin involved by malignant neoplasm
6435491000006116		Linear extent of involvement of carcinoma
6435651000006112		Surgical distal mucosal margin involved by in situ carcinoma
6435661000006114		Surgical distal mucosal margin uninvolved by in situ carcinoma
6435671000006119		Surgical proximal mucosal margin uninvolved by in situ carcinoma
6435681000006116		Surgical proximal mucosal margin involved by in situ carcinoma
6435971000006114		Surgical omental margin uninvolved by malignant neoplasm
6435981000006112		Surgical lesser omental margin involved by malignant neoplasm

6435991 0000061 10		Surgical greater omental margin involved by malignant neoplasm
6436711 0000061 12		FIGO stage for cervical carcinoma
6436721 0000061 16		International Federation of Gynaecology and Obstetrics (FIGO) stage for cervical carcinoma
6436731 0000061 18		International Federation of Gynecology and Obstetrics stage for cervical carcinoma
6436741 0000061 11		FIGO stage finding for cervical carcinoma
6436751 0000061 13		International Federation of Gynaecology and Obstetrics (FIGO) stage finding for cervical carcinoma
6436761 0000061 10		International Federation of Gynecology and Obstetrics stage finding for cervical carcinoma
6436771 0000061 15		FIGO stage for vulvar carcinoma
6436781 0000061 17		International Federation of Gynaecology and Obstetrics (FIGO) stage for vulvar carcinoma
6436791 0000061 19		International Federation of Gynecology and Obstetrics stage for vulvar carcinoma
6436801 0000061 18		FIGO stage finding for vulvar carcinoma
6436811 0000061 15		International Federation of Gynaecology and Obstetrics (FIGO) stage finding for vulvar carcinoma
6436821 0000061 11		International Federation of Gynecology and Obstetrics stage finding for vulvar carcinoma
6436971 0000061 18		FIGO stage for endometrial carcinoma
6436981 0000061 15		International Federation of Gynaecology and Obstetrics (FIGO) stage for endometrial carcinoma
6436991 0000061 17		International Federation of Gynecology and Obstetrics stage for endometrial carcinoma
6437001 0000061 13		FIGO stage finding for endometrial carcinoma
6437011 0000061 11		International Federation of Gynaecology and Obstetrics (FIGO) stage finding for endometrial carcinoma
6437021 0000061 15		International Federation of Gynecology and Obstetrics stage finding for endometrial carcinoma

6437431000006114		Surgical bronchial margin involved by squamous cell carcinoma in situ
6437481000006110		Linear extent of involvement of carcinoma in specimen obtained by needle biopsy
6501491000006118		Well differentiated adenocarcinoma, gastric foveolar type
6510041000006112		Minimal deviation adenocarcinoma of endocervical type
6510810000006111	7G03K00	Excision of malignant skin tumour
6549381000006118		Squamous cell carcinoma antigen level
6556511000006111		Surgical mucosal margin uninvolved by malignant neoplasm
6556521000006115		Mucosal surgical margin uninvolved by malignant neoplasm
6556531000006117		Surgical mucosal margin involved by malignant neoplasm
6556541000006110		Mucosal surgical margin involved by malignant neoplasm
6556611000006112		Surgical deep margin involved by malignant neoplasm
6556621000006116		Deep surgical margin involved by malignant neoplasm
6556631000006118		Surgical mucosal margin uninvolved by in situ carcinoma
6556641000006111		Mucosal surgical margin uninvolved by in situ carcinoma
6556651000006113		Surgical mucosal margin involved by in situ carcinoma
6556661000006110		Mucosal surgical margin involved by in situ carcinoma
6556671000006115		Surgical deep margin uninvolved by malignant neoplasm
6556691000006119		Surgical deep margin uninvolved by in situ carcinoma
6556701000006119		Deep surgical margin uninvolved by in situ carcinoma

6556711000006116		Surgical deep margin involved by in situ carcinoma
6556721000006112		Deep surgical margin involved by in situ carcinoma
6557191000006111		Cancer diagnosis discussed with partner in relationship
6557651000006111		pTis: Carcinoma in situ, intraepithelial (colon/rectum)
6557781000006114		Surgical deep margin involved by malignant neoplasm, focal
6557791000006112		Deep surgical margin involved by malignant neoplasm, focal
6557801000006113		Surgical deep margin involved by malignant neoplasm, multifocal
6557811000006111		Deep surgical margin involved by malignant neoplasm, multifocal
6565521000006119		Small cell carcinoma of prostate
6565571000006118		Surgical renal capsular margin involved by malignant neoplasm
6565581000006115		Renal capsular surgical margin involved by malignant neoplasm
6565591000006117		Surgical perinephric fat margin involved by malignant neoplasm
6565601000006113		Perinephric fat surgical margin involved by malignant neoplasm
6565611000006111		Surgical renal vein margin involved by malignant neoplasm
6565631000006117		Surgical Gerota's fascial margin involved by malignant neoplasm
6565641000006110		Gerota's fascia surgical margin involved by malignant neoplasm
6565651000006112		Surgical Gerota fascial margin involved by malignant neoplasm
6565721000006111		Surgical ureteral margin involved by malignant neoplasm
6565731000006114		Ureteral surgical margin involved by malignant neoplasm

6565781000006110		Surgical renal parenchymal margin involved by malignant neoplasm
6566411000006118		Surgical endocervical margin uninvolved by malignant neoplasm
6566451000006117		Surgical endocervical margin involved by malignant neoplasm
6566461000006115		Surgical endocervical margin involved by malignant neoplasm, focal
6566471000006110		Surgical endocervical margin involved by malignant neoplasm, diffuse
6566561000006119		Surgical exocervical margin involved by malignant neoplasm
6566591000006110		Surgical exocervical margin uninvolved by malignant neoplasm
6566601000006119		Surgical exocervical margin involved by malignant neoplasm, focal
6566611000006116		Surgical exocervical margin involved by malignant neoplasm, diffuse
6566901000006112		pT1 (I): Cervical carcinoma confined to uterus (extension to corpus should be disregarded) (TNM category and FIGO stage) (uterine cervix)
6566951000006111		pT1a (IA): Invasive carcinoma diagnosed only by microscopy. All macroscopically visible lesions (even with superficial invasion) are pT1b/IB (TNM category and FIGO stage) (uterine cervix)
6567021000006115		pT2 (II): Cervical carcinoma invades beyond uterus but not to pelvic wall or to lower third of vagina (TNM category and FIGO stage) (uterine cervix)
6567201000006119		Surgical distal margin involved by in situ carcinoma
6567211000006116		Surgical distal margin uninvolved by in situ carcinoma
6567471000006112		pT3a (IIIA): Tumour involves serosa, AND/OR adnexa (direct extension or metastasis) AND/OR cancer cells in ascites or peritoneal washings (TNM category and FIGO stage) (corpus uteri)
6567481000006110		pT3a (IIIA): Tumor involves serosa, AND/OR adnexa (direct extension or metastasis) AND/OR cancer cells in ascites or peritoneal washings (TNM category and FIGO stage) (corpus uteri)
6569951000006118		Surgical parenchymal margin uninvolved by malignant neoplasm
6569981000006114		Surgical bile duct margin uninvolved by malignant neoplasm
6569991000006112		Surgical bile duct margin involved by malignant neoplasm

6570011 0000061 18		Surgical bile duct margin involved by carcinoma in situ
6570021 0000061 14		Surgical bile duct margin uninvolved by carcinoma in situ
6570251 0000061 14		Carcinoma of skin (excluding eyelid, vulva, penis, and melanomas) TNM finding
6570261 0000061 11		pT1: Tumour 2 cm or less in greatest dimension (carcinoma of skin excluding eyelid, vulva, penis, and melanomas)
6570271 0000061 16		pT1: Tumor 2 cm or less in greatest dimension (carcinoma of skin excluding eyelid, vulva, penis, and melanomas)
6570281 0000061 18		pT1a: Tumour 2 cm or less in greatest dimension, limited to dermis or 2 mm or less in thickness (carcinoma of skin excluding eyelid, vulva, penis, and melanomas)
6570291 0000061 15		pT1a: Tumor 2 cm or less in greatest dimension, limited to dermis or 2 mm or less in thickness (carcinoma of skin excluding eyelid, vulva, penis, and melanomas)
6570301 0000061 19		pT1b: Tumour 2 cm or less in greatest dimension, limited to dermis and greater than 2 mm in thickness, but not more than 6 mm in thickness (carcinoma of skin excluding eyelid, vulva, penis, and melanomas)
6570311 0000061 16		pT1b: Tumor 2 cm or less in greatest dimension, limited to dermis and greater than 2 mm in thickness, but not more than 6 mm in thickness (carcinoma of skin excluding eyelid, vulva, penis, and melanomas)
6570321 0000061 12		pT1c: Tumour 2 cm or less in greatest dimension, invading the subcutis and/or greater than 6 mm in thickness (carcinoma of skin excluding eyelid, vulva, penis, and melanomas)
6570331 0000061 10		pT1c: Tumor 2 cm or less in greatest dimension, invading the subcutis and/or greater than 6 mm in thickness (carcinoma of skin excluding eyelid, vulva, penis, and melanomas)
6570341 0000061 17		pT2: Tumour more than 2 cm, but not more than 5 cm, in greatest dimension (carcinoma of skin excluding eyelid, vulva, penis, and melanomas)
6570351 0000061 15		pT2: Tumor more than 2 cm, but not more than 5 cm, in greatest dimension (carcinoma of skin excluding eyelid, vulva, penis, and melanomas)
6570361 0000061 18		pT2a: Tumour more than 2 cm, but not more than 5 cm, in greatest dimension, limited to dermis or greater than 2 mm in thickness (carcinoma of skin excluding eyelid, vulva, penis, and melanomas)
6570371 0000061 13		pT2a: Tumor more than 2 cm, but not more than 5 cm, in greatest dimension, limited to dermis or greater than 2 mm in thickness (carcinoma of skin excluding eyelid, vulva, penis, and melanomas)
6570381 0000061 11		pT2b: Tumour more than 2 cm, but not more than 5 cm, in greatest dimension, limited to dermis and greater than 2 mm in thickness, but not more than 6 mm in thickness (carcinoma of skin excluding eyelid, vulva, penis, and melanomas)
6570391 0000061 14		pT2b: Tumor more than 2 cm, but not more than 5 cm, in greatest dimension, limited to dermis and greater than 2 mm in thickness, but not more than 6 mm in thickness (carcinoma of skin excluding eyelid, vulva, penis, and melanomas)
6570401 0000061 11		pT2c: Tumour more than 2 cm, but not more than 5 cm, in greatest dimension, invading the subcutis and/or greater than 6 mm in thickness (carcinoma of skin excluding eyelid, vulva, penis, and melanomas)
6570411 0000061 14		pT2c: Tumor more than 2 cm, but not more than 5 cm, in greatest dimension, invading the subcutis and/or greater than 6 mm in thickness (carcinoma of skin excluding eyelid, vulva, penis, and melanomas)

6570421 0000061 18		pT3: Tumour more than 5 cm in greatest dimension (carcinoma of skin excluding eyelid, vulva, penis, and melanomas)
6570431 0000061 15		pT3: Tumor more than 5 cm in greatest dimension (carcinoma of skin excluding eyelid, vulva, penis, and melanomas)
6570441 0000061 13		pT3a: Tumour more than 5 cm in greatest dimension, limited to dermis or not more than 2 mm in thickness (carcinoma of skin excluding eyelid, vulva, penis, and melanomas)
6570451 0000061 10		pT3a: Tumor more than 5 cm in greatest dimension, limited to dermis or not more than 2 mm in thickness (carcinoma of skin excluding eyelid, vulva, penis, and melanomas)
6570461 0000061 12		pT3b: Tumour more than 5 cm in greatest dimension, limited to dermis and greater than 2 mm in thickness, but not more than 6 mm in thickness (carcinoma of skin excluding eyelid, vulva, penis, and melanomas)
6570471 0000061 17		pT3b: Tumor more than 5 cm in greatest dimension, limited to dermis and greater than 2 mm in thickness, but not more than 6 mm in thickness (carcinoma of skin excluding eyelid, vulva, penis, and melanomas)
6570481 0000061 19		pT3c: Tumour more than 5 cm in greatest dimension, invading the subcutis and/or greater than 6 mm in thickness (carcinoma of skin excluding eyelid, vulva, penis, and melanomas)
6570491 0000061 16		pT3c: Tumor more than 5 cm in greatest dimension, invading the subcutis and/or greater than 6 mm in thickness (carcinoma of skin excluding eyelid, vulva, penis, and melanomas)
6570511 0000061 10		pT4: Tumour invades deep extradermal structures (i.e. cartilage, skeletal muscle, or bone) (carcinoma of skin excluding eyelid, vulva, penis, and melanomas)
6570521 0000061 19		pT4: Tumor invades deep extradermal structures (i.e. cartilage, skeletal muscle, or bone) (carcinoma of skin excluding eyelid, vulva, penis, and melanomas)
6570531 0000061 16		pT4b: Tumour invades deep extradermal structures (i.e. cartilage, skeletal muscle, or bone) greater than 6mm in thickness (carcinoma of skin excluding eyelid, vulva, penis, and melanomas)
6570541 0000061 14		pT4b: Tumor invades deep extradermal structures (i.e. cartilage, skeletal muscle, or bone) greater than 6mm in thickness (carcinoma of skin excluding eyelid, vulva, penis, and melanomas)
6570551 0000061 11		pT4a: Tumour invades deep extradermal structures (i.e. cartilage, skeletal muscle, or bone) not more than 6mm in thickness (carcinoma of skin excluding eyelid, vulva, penis, and melanomas)
6570561 0000061 13		pT4a: Tumor invades deep extradermal structures (i.e. cartilage, skeletal muscle, or bone) not more than 6mm in thickness (carcinoma of skin excluding eyelid, vulva, penis, and melanomas)
6570611 0000061 11		Surgical lateral margin uninvolved by malignant melanoma
6570621 0000061 15		Surgical lateral margin involved by malignant melanoma
6570651 0000061 12		Surgical deep margin uninvolved by malignant melanoma
6570661 0000061 14		Surgical deep margin involved by malignant melanoma
6570701 0000061 18		Surgical lateral margin uninvolved by malignant neoplasm

6570711000006115		Surgical lateral margin uninvolved by in situ carcinoma
6570721000006111		Surgical lateral margin involved by in situ carcinoma
6570911000006117		Stage I: Tumour confined to gland, 5 cm or less (adrenal cortical carcinoma)
6570921000006113		Stage I: Tumor confined to gland, 5 cm or less (adrenal cortical carcinoma)
6570931000006111		Stage 1: Tumour confined to gland, 5 cm or less (adrenal cortical carcinoma)
6570941000006118		Stage 1: Tumor confined to gland, 5 cm or less (adrenal cortical carcinoma)
6570951000006116		Stage II: Tumour confined to gland, greater than 5 cm (adrenal cortical carcinoma)
6570961000006119		Stage II: Tumor confined to gland, greater than 5 cm (adrenal cortical carcinoma)
6570971000006114		Stage 2: Tumour confined to gland, greater than 5 cm (adrenal cortical carcinoma)
6570981000006112		Stage 2: Tumor confined to gland, greater than 5 cm (adrenal cortical carcinoma)
6570991000006110		Stage III: Extraglandular extension of tumour without other organ involvement (adrenal cortical carcinoma)
6571001000006111		Stage III: Extraglandular extension of tumor without other organ involvement (adrenal cortical carcinoma)
6571011000006114		Stage 3: Extraglandular extension of tumour without other organ involvement (adrenal cortical carcinoma)
6571021000006118		Stage 3: Extraglandular extension of tumor without other organ involvement (adrenal cortical carcinoma)
6571031000006115		Stage IV: Distant metastasis or extension into other organs (adrenal cortical carcinoma)
6571041000006113		Stage 4: Distant metastasis or extension into other organs (adrenal cortical carcinoma)
6571151000006114		Surgical margin involved by in situ carcinoma/adenoma
6571161000006111		Surgical margin uninvolved by in situ carcinoma/adenoma
6571211000006119		Surgical common bile duct margin involved by in situ carcinoma

6571221000006110		Surgical common bile duct margin uninvolved by in situ carcinoma
6571231000006113		Surgical pancreatic duct margin uninvolved by in situ carcinoma
6571241000006115		Surgical pancreatic duct margin involved by in situ carcinoma
6572691000006117		Surgical margin uninvolved by in situ carcinoma
6572721000006110		Surgical margin involved by in situ carcinoma
6572731000006113		Surgical margin involvement by in situ carcinoma not applicable
6573111000006112		Urothelial carcinoma WHO/ISUP histologic grade
6573121000006116		Urothelial carcinoma World Health Organization/International Society of Urological Pathology histologic grade
6573251000006117		pTa: Non-invasive papillary carcinoma (urinary bladder, ureter, renal pelvis)
6573261000006115		pTis: Flat carcinoma in situ (urinary bladder, ureter, renal pelvis)
6573741000006110		Surgical margin site involved by in situ carcinoma
6574491000006113		pTis: Intratubular germ cell neoplasia (carcinoma in situ) (testis)
6575331000006115		Tunica vaginalis involved by direct extension of malignant neoplasm
6575341000006113		Perihilar fat involved by direct extension of malignant neoplasm
6575361000006112		Rete testis involved by direct extension of malignant neoplasm
6575371000006117		Epididymis involved by direct extension of malignant neoplasm
6575381000006119		Scrotal wall involved by direct extension of malignant neoplasm
6576051000006115		Surgical pancreatic parenchymal margin involved by in situ carcinoma
6576061000006118		Surgical margin closest to malignant neoplasm

6576071000006113		Surgical distal pancreatic margin involved by malignant neoplasm
6576081000006111		Surgical common bile duct margin involved by malignant neoplasm
6576091000006114		Surgical proximal pancreatic margin involved by malignant neoplasm
6576101000006115		Surgical uncinate process margin involved by malignant neoplasm
6576111000006117		Surgical posterior retroperitoneal pancreatic margin involved by malignant neoplasm
6577881000006118		Surgical ductal margin uninvolved by in situ carcinoma
6577891000006115		Mixed acinar-endocrine carcinoma
6577901000006116		Mixed ductal-endocrine carcinoma
6579611000006117		pT4: Extraocular tumor extension which includes sites as per American Joint Committee on Cancer 6th Edition definition (retinoblastoma)
6579621000006113		pT4: Extraocular tumour extension which includes sites as per American Joint Committee on Cancer 6th Edition definition (retinoblastoma)
6580311000006112		Malignant mast cell neoplasm
6581021000006110		Surgical circumferential margin involved by malignant neoplasm
6581471000006118		Mucoepidermoid carcinoma, low grade
6581481000006115		Mucoepidermoid carcinoma, intermediate grade
6581491000006117		Mucoepidermoid carcinoma, high grade
6581501000006113		Salivary duct carcinoma
6581511000006111		Surgical pancreatic margin involved by malignant neoplasm
6581521000006115		Surgical pancreatic margin uninvolved by malignant neoplasm
6581651000006113		Surgical proximal margin involvement by in situ carcinoma/adenoma not applicable

6581791000006114		Surgical cystic duct margin uninvolved by in situ carcinoma
6581801000006110		Surgical cystic duct margin involved by malignant neoplasm
6583081000006110		Surgical lateral (mucosal/mural) margin involved by in situ carcinoma/adenoma
6583221000006119		Microcalcifications present in malignant neoplasm
6583231000006116		Microcalcifications present in ductal carcinoma in situ
6583681000006118		Perivesical fat involved by direct extension of malignant neoplasm
6583711000006117		Rectum involved by direct extension of malignant neoplasm
6583721000006113		Uterus and adnexae involved by direct extension of malignant neoplasm
6583731000006111		Pelvic sidewall involved by direct extension of malignant neoplasm
6585181000006112		Surgical proximal bile duct margin involved by malignant neoplasm
6585191000006110		Surgical distal bile duct margin involved by malignant neoplasm
6585921000006111		Surgical cystic duct margin involved by in situ carcinoma
6586301000006111		pT3: Tumor involves any of the ipsilateral pleural surfaces plus additional sites as by American Joint Committee on Cancer 6th Edition definition (thoracic mesothelioma)
6587291000006112		pT4: Tumor involves any of the ipsilateral pleural surfaces plus additional sites as by American Joint Committee on Cancer 6th Edition definition (thoracic mesothelioma)
6587301000006113		pT4: Tumour involves any of the ipsilateral pleural surfaces plus additional sites as by American Joint Committee on Cancer 6th Edition definition (thoracic mesothelioma)
6587741000006111		pT1a (IA): Tumour limited to one ovary; capsule intact, no tumour on ovarian surface. No malignant cells in ascites or peritoneal washings (TNM category and FIGO stage) (ovary)
6587751000006113		pT1a (IA): Tumor limited to one ovary; capsule intact, no tumor on ovarian surface. No malignant cells in ascites or peritoneal washings (TNM category and FIGO stage) (ovary)
6587761000006110		pT1b (IB): Tumour limited to both ovaries; capsule intact, no tumour on ovarian surface. No malignant cells in ascites or peritoneal washings (TNM category and FIGO stage) (ovary)
6587771000006115		pT1b (IB): Tumor limited to both ovaries; capsule intact, no tumor on ovarian surface. No malignant cells in ascites or peritoneal washings (TNM category and FIGO stage) (ovary)

6587781 0000061 17		pT1c (IC): Tumour limited to one or both ovaries with any of the following: capsule ruptured, tumour on ovarian surface, malignant cells in ascites or peritoneal washings (TNM category and FIGO stage) (ovary)
6587791 0000061 19		pT1c (IC): Tumor limited to one or both ovaries with any of the following: capsule ruptured, tumor on ovarian surface, malignant cells in ascites or peritoneal washings (TNM category and FIGO stage) (ovary)
6587821 0000061 11		pT2a (IIA): Extension to AND/OR implants on uterus AND/OR tube(s). No malignant cells in ascites or peritoneal washings (TNM category and FIGO stage) (ovary)
6587831 0000061 14		pT2b (IIB): Extension to AND/OR implants on other pelvic tissues. No malignant cells in ascites or peritoneal washings (TNM category and FIGO stage) (ovary)
6587841 0000061 16		pT2c (IIC): Pelvic extension AND/OR implants (T2a or T2b/IIA or IIB) with malignant cells in ascites or peritoneal washings (TNM category and FIGO stage) (ovary)
6613831 0000061 12		Pleomorphic malignant fibrous histiocytoma of skin
6616721 0000061 13		Malignant tumor of prostate
6616731 0000061 11		Malignant prostatic tumour
6616741 0000061 18		Malignant prostatic tumor
6616751 0000061 16		CA - Cancer of prostate
6616761 0000061 19		Cancer of prostate
6619851 0000061 19		Chromaffin cell neoplasm
6620961 0000061 12		Malignant tumour of urinary bladder
6620971 0000061 17		Malignant tumor of urinary bladder
6620981 0000061 19		Bladder cancer
6621001 0000061 15		CA - Bladder cancer
6621841 0000061 10		Specimen margin involved by ductal carcinoma in situ
6621881 0000061 16		Choriocarcinoma, biphasic
6621891 0000061 18		Biphasic choriocarcinoma

6621961000006118		Vagina involved by direct extension of malignant neoplasm
6622351000006112		Warty (condylomatous) carcinoma
6622361000006114		Condylomatous carcinoma
6622371000006119		Warty carcinoma
6622691000006114		Monitoring of patient with cancer
6622781000006117		Prostatic stroma involved by direct extension of malignant neoplasm
6623021000006114		Mucinous adenocarcinoma, intestinal type
6623081000006113		pT1c (IC): Tumour limited to one or both tubes with extension onto or through the tubal serosa, or with malignant cells in ascites or peritoneal washings (TNM category and FIGO stage) (fallopian tube)
6623091000006111		pT1c (IC): Tumor limited to one or both tubes with extension onto or through the tubal serosa, or with malignant cells in ascites or peritoneal washings (TNM category and FIGO stage) (fallopian tube)
6623211000006113		Transitional cell carcinoma with squamous differentiation
6623321000006112		Basal cell carcinoma with eccrine differentiation
6623331000006110		Eccrine basal cell carcinoma
6623341000006117		Seminal vesicle involved by direct extension of malignant neoplasm
6623501000006112		Malignant trophoblastic tumour, type cannot be determined
6623511000006110		Malignant trophoblastic tumor, type cannot be determined
6623621000006115		Basal cell carcinoma with adnexal differentiation
6623661000006114		Adenocarcinoma of prostate
6623731000006114		Squamous cell carcinoma in post-traumatic skin lesion
6623951000006116		pT4b: Extrathyroidal anaplastic carcinoma - surgically unresectable (anaplastic carcinoma) (thyroid)

6624281000006115		Islet cell neoplasm
6624351000006117		Bartholin's gland carcinoma
6624361000006115		Bartholin gland carcinoma
6624471000006119		Mucinous eccrine carcinoma
6624571000006115		Keratotic basal cell carcinoma
6624581000006117		Adenocarcinoma and squamous carcinoma histologic grade
6624801000006111		Transitional cell carcinoma with glandular differentiation
6624901000006117		Basal cell carcinoma in basal cell naevus syndrome
6624911000006119		Basal cell carcinoma in basal cell nevus syndrome
6625001000006117		pT4a: Intrathyroidal anaplastic carcinoma - surgically resectable (anaplastic carcinoma) (thyroid)
6625121000006111		Squamous cell carcinoma, nonkeratinising, differentiated
6625131000006114		Squamous cell carcinoma, nonkeratinizing, differentiated
6625161000006117		Pigmented basal cell carcinoma
6625251000006110		Squamous cell carcinoma of prostate
6625431000006116		Carcinoma of minor salivary gland, histologic type
6626041000006114		Neurotropic melanoma, malignant
6626051000006111		Malignant neurotropic melanoma
6626351000006113		Specimen margin closest to ductal carcinoma in situ
6626381000006117		Carcinoma resembling breast carcinoma

6626431000006113		Choriocarcinoma, monophasic
6626441000006115		Monophasic choriocarcinoma
6626901000006113		Squamous cell carcinoma, nonkeratinising, mixed differentiated and undifferentiated
6626911000006111		Squamous cell carcinoma, nonkeratinizing, mixed differentiated and undifferentiated
6626951000006112		pT2c (IIC): Tumour involves one or both fallopian tubes with pelvic extension with malignant cells in ascites or peritoneal washings (TNM category and FIGO stage) (fallopian tube)
6626961000006114		pT2c (IIC): Tumor involves one or both fallopian tubes with pelvic extension with malignant cells in ascites or peritoneal washings (TNM category and FIGO stage) (fallopian tube)
6627361000006112		Ureter involved by direct extension of malignant neoplasm
6627541000006116		Metaplastic squamous cell carcinoma
6627611000006119		Carcinoma of upper aerodigestive tract, histologic type
6627661000006116		Basal cell carcinoma with follicular differentiation
6627671000006111		Infundibulocystic basal cell carcinoma
6627681000006114		Follicular basal cell carcinoma
6630901000006113		Malignant neoplasm with pilar differentiation
6630911000006111		Malignant epithelial neoplasm - category
6630991000006118		Primary malignant neoplasm of blood vessel of upper arm
6631021000006110		Signet ring carcinoma, primary cutaneous
6631121000006111		Adenomatous neoplasm of borderline malignancy
6631211000006114		Basal cell carcinoma of postauricular skin
6631561000006114		Carcinoma in situ - category

6631571000006119		Non-invasive carcinoma
6631791000006114		Basal cell carcinoma of preauricular skin
6631801000006110		Ductal carcinoma in situ - category
6631931000006112		Malignant cylindroma of skin
6631941000006119		Malignant dermal cylindroma
6632161000006113		Basal cell carcinoma of earlobe
6632281000006111		Basal cell carcinoma with matrical differentiation
6632351000006114		Malignant adenomatous neoplasm - category
6632441000006116		Adenoid cystic eccrine carcinoma
6632451000006119		Secondary malignant neoplasm of blood vessel of upper arm
6632691000006111		In situ adenomatous neoplasm - category
6633981000006114		Basal cell carcinoma of pinnal sulcus
6633991000006112		Secondary malignant neoplasm of blood vessel of lower leg
6634081000006112		Intraepithelial squamous cell carcinoma
6634151000006117		Basal cell carcinoma, micronodular
6634181000006113		Primary malignant neoplasm of blood vessel of lower leg
6634371000006112		Basal cell carcinoma with sebaceous differentiation
6635051000006115		Malignant nerve sheath tumour - category
6635061000006118		Malignant nerve sheath tumor - category

6635121 0000061 13		Malignant chondroid syringoma
6635341 0000061 15		Vascular neoplasm of skin
6635411 0000061 12		Small cell eccrine carcinoma
6635441 0000061 11		Basal cell carcinoma - category
6635641 0000061 13		Clear cell eccrine hidradenocarcinoma
6635791 0000061 11		Squamous cell carcinoma - category
6635801 0000061 12		Malignant blood vessel neoplasm, primary
6636021 0000061 16		Malignant fibromatous neoplasm
6636121 0000061 15		Malignant atrophic papulosis
6636161 0000061 14		MAP - malignant atrophic papulosis
6636171 0000061 19		Malignant atrophic papulosis of Degos
6636181 0000061 16		Malignant neoplasm with eccrine differentiation
6636191 0000061 18		Eccrine carcinoma of skin
6636201 0000061 15		Basal cell carcinoma of antihelix of ear
6636221 0000061 13		Myxoid malignant fibrous histiocytoma of skin
6636271 0000061 14		Mucoepidermoid eccrine carcinoma
6636341 0000061 13		Malignant neoplasm with apocrine differentiation
6636671 0000061 11		Eccrine ductal carcinoma
6636751 0000061 11		Malignant fibrohistiocytic neoplasm - category

6666221 0000061 15		Basal cell carcinoma of lower eyelid
6666231 0000061 17		Basal cell carcinoma of medial canthus
6666241 0000061 10		Basal cell carcinoma of lateral canthus
6666251 0000061 12		Basal cell carcinoma of root of nose
6666261 0000061 14		Basal cell carcinoma of dorsum of nose
6666271 0000061 19		Basal cell carcinoma of lateral side wall of nose
6666281 0000061 16		Basal cell carcinoma of ala nasi
6666291 0000061 18		Basal cell carcinoma of supratip of nose
6666301 0000061 17		Basal cell carcinoma of tip of nose
6666311 0000061 19		Basal cell carcinoma of nasal columella
6666321 0000061 10		Basal cell carcinoma of nasolabial groove
6666331 0000061 13		Basal cell carcinoma of upper lip
6666341 0000061 15		Basal cell carcinoma of lower lip
6666351 0000061 18		Basal cell carcinoma of cheek
6666361 0000061 16		Basal cell carcinoma of chin
6666371 0000061 11		Cancer of chin, basal cell
6666381 0000061 14		Basal cell carcinoma of neck
6666391 0000061 12		Cancer of skin of neck, basal cell
6666401 0000061 14		Basal cell carcinoma of helix of ear

6666411000006112		Basal cell carcinoma of conchal bowl of ear
6666421000006116		Basal cell carcinoma of antitragus
6666431000006118		Basal cell carcinoma of tragus
6666441000006111		Basal cell carcinoma of obverse of pinna
6666451000006113		Basal cell carcinoma of anterior chest
6666461000006110		Basal cell carcinoma of abdomen
6666471000006115		Basal cell carcinoma of upper back
6666481000006117		Basal cell carcinoma of lower back
6666491000006119		Basal cell carcinoma of face
6666501000006110		Cancer of skin of face, basal cell
6666511000006113		Basal cell carcinoma of hand
6666521000006117		Carcinoma on hand, basal cell
6666531000006119		Cancer of hand, basal cell
6666541000006112		Basal cell carcinoma of upper extremity
6666551000006114		Basal cell carcinoma of arm
6666561000006111		Cancer of skin of upper limb, basal cell
6666571000006116		Basal cell carcinoma of lower extremity
6666581000006118		Cancer of skin of lower limb, basal cell
6666591000006115		Basal cell carcinoma of truncal skin

6666601 0000061 11		Cancer of skin of trunk, basal cell
6666611 0000061 14		Basal cell carcinoma - adamantinoid
6666621 0000061 18		Basal cell carcinoma - adenoid
6666631 0000061 15		Basal cell carcinoma - follicular
6666641 0000061 13		Basal cell carcinoma - infiltrative
6666651 0000061 10		Basal cell carcinoma - keratotic
6666671 0000061 17		Basal cell carcinoma with matrical differentiation
6666681 0000061 19		Basal cell carcinoma with granular cell change
6666691 0000061 16		Basal cell carcinoma with monster cells
6666701 0000061 16		Basal cell carcinoma with sebaceous differentiation
6666711 0000061 18		Basal cell carcinoma with signet ring change
6666721 0000061 14		Basal cell carcinoma - primary
6666731 0000061 12		Metastatic basal cell carcinoma
6666741 0000061 19		Recurrent basal cell carcinoma
6666751 0000061 17		Basal cell carcinoma recurrent following excision
6666761 0000061 15		Basal cell carcinoma recurrent following Mohs' excision
6666771 0000061 10		Basal cell carcinoma recurrent following curettage
6666781 0000061 13		Basal cell carcinoma recurrent following cryosurgery
6666791 0000061 11		Basal cell carcinoma recurrent following radiotherapy

6666801000006112		Basal cell carcinoma - first recurrence
6666811000006110		Basal cell carcinoma - second recurrence
6666821000006119		Basal cell carcinoma - third recurrence
6666831000006116		Basal cell carcinoma - multiple recurrences (more than three times)
6667051000006118		Malignant melanoma (radial growth phase)
6667061000006116		In situ superficial spreading malignant melanoma
6667071000006111		In situ acral lentiginous malignant melanoma
6667081000006114		Malignant melanoma (vertical growth phase)
6667091000006112		Congenital malignant melanoma
6667101000006118		Materno-foetal metastatic malignant melanoma
6667111000006115		Materno-fetal metastatic malignant melanoma
6667121000006111		Malignant melanoma of soft tissues
6667141000006116		Malignant melanoma animal-type
6667151000006119		Metastatic malignant melanoma with diffuse hypermelanosis
6667161000006117		Multiple primary malignant melanomata
6667991000006111		Hypomelanosis surrounding melanocytic neoplasm
6668151000006115		Malignant neoplasm of nail apparatus
6668321000006114		Malignant vascular tumour of skin
6668331000006112		Malignant vascular tumor of skin

6668481000006116		Cancer-associated vasculitis
6670291000006110		Squamous neoplasm of surface epithelium
6670311000006114		Carcinoma-in-situ of oral mucosa
6670321000006118		Intraepithelial squamous carcinoma of anogenital region
6670331000006115		Squamous cell carcinoma
6670341000006113		Squamous cell carcinoma of anogenital area
6670351000006110		Squamous cell carcinoma of nail apparatus
6670361000006112		Basal cell carcinoma of nose
6670371000006117		Cancer of nose, basal cell
6670381000006119		Basal cell carcinoma of skin of lip
6670391000006116		Cancer of skin of lip, basal cell
6670401000006119		Basal cell carcinoma of ear
6670411000006116		Cancer of skin of ear, basal cell
6671071000006113		Malignant neoplasm of subcutaneous fibrous tissue
6671091000006114		Malignant fibrohistiocytic tumour of skin
6671101000006115		Malignant fibrohistiocytic tumor of skin
6671131000006111		Malignant tumour of nerve sheath origin
6671141000006118		Malignant tumor of nerve sheath origin
6671781000006113		Anogenital verrucous carcinoma of Buschke-LÃ�Â¶wenstein

666718110000006110		Anogenital verrucous carcinoma of Buschke-Lowenstein
66718210000006119		Penile verrucous carcinoma of Buschke-LÃÂ¶wenstein
66718510000006111		Verrucous carcinoma of penis (Buschke-LÃÂ¶wenstein)
66718610000006113		Penile verrucous carcinoma of Buschke-Lowenstein
66718710000006118		Vulval verrucous carcinoma of Buschke-LÃÂ¶wenstein
66719010000006118		Verrucous carcinoma of vulva (Buschke-LÃÂ¶wenstein)
66719110000006115		Vulval verrucous carcinoma of Buschke-Lowenstein
66783710000006115		Hypermelanosis due to malignant ACTH/MSH-secreting tumour
66783810000006117		Hypermelanosis due to malignant ACTH/MSH-secreting tumor
66783910000006119		Hypermelanosis due to malignant adrenocorticotropic hormone/melanocyte stimulating hormone-secreting tumor
66784010000006117		Hypermelanosis due to malignant adrenocorticotropic hormone/melanocyte stimulating hormone-secreting tumour
66787310000006119		Hypomelanosis surrounding malignant melanoma
66813910000006115		Localised skin involvement by breast carcinoma
66814010000006118		Localized skin involvement by breast carcinoma
66815310000006113		Squamous cell carcinoma of penis
66815410000006115		Penile squamous cell carcinoma
66815510000006118		Cancer of the penis, squamous cell
66818010000006115		Perianal intraepidermal carcinoma
66856410000006117		PUVA therapy-associated basal cell carcinoma

6685651000006115		Psoralen and long-wave ultraviolet radiation (PUVA) therapy-associated basal cell carcinoma
6685661000006118		Psoralen and long-wave ultraviolet radiation therapy-associated basal cell carcinoma
6685681000006111		Psoralen and long-wave ultraviolet radiation (PUVA) therapy-associated squamous cell carcinoma
6685691000006114		Psoralen and long-wave ultraviolet radiation therapy-associated squamous cell carcinoma
6685701000006114		PUVA therapy-associated malignant melanoma
6685711000006112		Psoralen and long-wave ultraviolet radiation (PUVA) therapy-associated malignant melanoma
6685721000006116		Psoralen and long-wave ultraviolet radiation therapy-associated malignant melanoma
6688811000006111		Intraepidermal squamous carcinoma of scalp
6688831000006117		Intraepidermal squamous carcinoma of face
6688851000006112		Intraepidermal squamous carcinoma of forehead
6688871000006119		Intraepidermal squamous carcinoma of ear
6688891000006118		Intraepidermal squamous carcinoma of hand
6688911000006116		Intraepidermal squamous carcinoma of arm
6688931000006110		Intraepidermal squamous carcinoma of upper extremity
6688941000006117		Intraepidermal squamous carcinoma of leg
6688951000006115		Intraepidermal squamous carcinoma of lower extremity
6688971000006113		Intraepidermal squamous carcinoma of trunk
6689131000006117		Multiple intraepidermal squamous carcinomata
6689141000006110		Verrucous carcinoma of oral cavity

6689151000006112		Snuff dipper's cancer
6689161000006114		Squamous cell carcinoma of vulva due to lichen sclerosus
6689171000006119		Squamous cell carcinoma of scalp
6689181000006116		Cancer of the scalp, squamous cell
6689191000006118		Squamous cell carcinoma of skin of face
6689201000006115		Squamous cell cancer of skin of face
6689211000006117		Squamous cell carcinoma of forehead
6689221000006113		Cancer of the forehead, squamous cell
6689231000006111		Squamous cell carcinoma of skin of ear
6689241000006118		Squamous cell cancer of skin of ear
6689251000006116		Squamous cell carcinoma of foot
6689261000006119		Squamous cell carcinoma of hand
6689271000006114		Squamous cell carcinoma of upper extremity
6689281000006112		Squamous cell carcinoma of skin of lower extremity
6689291000006110		Squamous cell cancer of skin of lower limb
6689301000006111		Squamous cell carcinoma of skin of trunk
6689311000006114		Spindle cell squamous cell carcinoma
6689331000006115		Acantholytic squamous cell carcinoma
6689341000006113		Adenosquamous cell carcinoma

6689351 0000061 10		Adenosquamous carcinoma
6689361 0000061 12		Signet ring squamous cell carcinoma
6689371 0000061 17		Verrucous squamous cell carcinoma
6689381 0000061 19		Verrucous epidermoid carcinoma
6689401 0000061 19		Metastatic squamous cell carcinoma
6689441 0000061 17		Circumscribed solid basal cell carcinoma
6689451 0000061 15		Nodulo-ulcerative basal cell carcinoma
6689461 0000061 18		Cystic basal cell carcinoma
6689471 0000061 13		Morpheic basal cell carcinoma
6689481 0000061 11		Cicatrising basal cell carcinoma
6689491 0000061 14		Superficial basal cell carcinoma
6689501 0000061 18		Multifocal basal cell carcinoma
6689511 0000061 15		Basal cell carcinoma of scalp
6689521 0000061 11		Cancer of scalp, basal cell
6689531 0000061 14		Basal cell carcinoma of forehead
6689541 0000061 16		Cancer of forehead, basal cell
6689551 0000061 19		Basal cell carcinoma of temple
6689561 0000061 17		Cancer of temple, basal cell
6689571 0000061 12		Basal cell carcinoma of glabella

6689581 0000061 10		Basal cell carcinoma of upper eyelid
6689591 0000061 13		Cancer of upper eyelid, basal cell
6689601 0000061 17		Minimal deviation malignant melanoma
6689611 0000061 19		Borderline malignant melanoma
6689631 0000061 13		Spindle cell malignant melanoma
6689641 0000061 15		Spitzoid malignant melanoma
6689651 0000061 18		Desmoplastic malignant melanoma
6689661 0000061 16		Neurotropic malignant melanoma
6689671 0000061 11		Malignant melanoma of oral cavity
6689681 0000061 14		Malignant melanoma of nail apparatus
6689691 0000061 12		Subungual malignant melanoma
6689711 0000061 10		Trichilemmal carcinoma
6689891 0000061 11		Eccrine ductal carcinoma
6689901 0000061 10		Eccrine ductal carcinoma of skin
6689911 0000061 13		Carcinoma simplex
6689931 0000061 19		Malignant acrospiroma
6689941 0000061 12		Malignant eccrine acrospiroma
6689951 0000061 14		Clear cell eccrine hidradenocarcinoma
6689961 0000061 11		Clear cell eccrine hidradenocarcinoma of skin

6689971000006116		Malignant cylindroma
6689981000006118		Malignant cylindroma of skin
6689991000006115		Malignant dermal cylindroma
6690001000006119		Malignant eccrine spiradenoma
6690011000006116		Malignant eccrine spiradenoma of skin
6690021000006112		Malignant spiradenoma
6690031000006110		Malignant chondroid syringoma
6690041000006117		Malignant mixed tumour of the skin
6690051000006115		Eccrine mixed tumour, malignant
6690061000006118		Malignant mixed tumor of the skin
6690071000006113		Eccrine mixed tumor, malignant
6690081000006111		Malignant chondroid syringoma of skin
6690091000006114		Small cell eccrine carcinoma
6690101000006115		Small cell eccrine carcinoma of skin
6690161000006119		Apocrine adenocarcinoma of skin
6690171000006114		Moll's gland adenocarcinoma
6690181000006112		Moll gland adenocarcinoma
6690191000006110		Ceruminous gland adenocarcinoma
6690201000006113		Ceruminous gland adenocarcinoma of skin

6690231 0000061 17		Lymphoepithelioma-like carcinoma of skin
6690251 0000061 12		Mixed eccrine/pilar adnexal carcinoma of skin
6690261 0000061 14		Undifferentiated adnexal carcinoma of skin
6690271 0000061 19		Mixed adnexal neoplasm of skin, unclassifiable
6690701 0000061 16		Malignant haemangioendothelioma
6690711 0000061 18		Malignant hemangioendothelioma
6691301 0000061 11		Malignant fibrous histiocytoma of skin
6691311 0000061 14		Superficial malignant fibrous histiocytoma of skin
6691321 0000061 18		Giant cell malignant fibrous histiocytoma of skin
6691331 0000061 15		Inflammatory malignant fibrous histiocytoma of skin
6691671 0000061 11		Malignant peripheral nerve sheath tumour
6691681 0000061 14		Malignant peripheral nerve sheath tumor
6691701 0000061 12		Malignant schwannoma
6691711 0000061 10		Epithelioid malignant nerve sheath tumour
6691721 0000061 19		Epithelioid malignant nerve sheath tumor
6691731 0000061 16		Melanotic malignant nerve sheath tumour
6691741 0000061 14		Melanotic malignant nerve sheath tumor
6691751 0000061 11		Malignant melanotic schwannoma
6691761 0000061 13		Malignant Triton tumour

6691771000006118		Malignant Triton tumor
6691781000006115		Malignant granular cell tumour
6691791000006117		Malignant granular cell tumor
6692421000006115		Malignant infiltration of oral cavity by underlying tumour
6692431000006117		Malignant infiltration of oral cavity by underlying tumor
6692441000006110		Malignant infiltration of skin by underlying tumour
6692451000006112		Malignant infiltration of skin by underlying tumor
6692471000006119		Metastatic carcinoma involving skin
6693231000006110		Anaplastic large T-cell systemic malignant lymphoma
6693241000006117		Angiocentric NK/T-cell malignant lymphoma involving skin
6693261000006118		Angiocentric natural killer/T-cell malignant lymphoma involving skin
6693921000006116		Malignant histiocytosis involving skin
6702751000006112		Malignant optic glioma
6715291000006117		Malignant pericardial effusion
6718931000006119		Squamous cell carcinoma of larynx
6718941000006112		Cancer of the larynx, squamous cell
6718961000006111		Breast cancer 1, early onset gene mutation carrier detection test
6719001000006115		Breast cancer 2, early onset gene mutation carrier detection test
6719331000006114		Widespread metastatic malignant neoplastic disease

6719341 0000061 16		Generalised cancer
6719371 0000061 12		Generalized cancer
6719391 0000061 13		Disseminated cancer
6719401 0000061 10		Malignant neoplasm, disseminated
6719411 0000061 13		CA - Disseminated cancer
6720741 0000061 16		Malignant neoplasm of metatarsal bone of foot
6721441 0000061 19		Surgical mesenteric margin uninvolved by malignant neoplasm
6721451 0000061 17		Surgical mesenteric margin involved by malignant neoplasm
6723061 0000061 11		Squamous cell carcinoma in situ of uterine cervix
6723591 0000061 14		Type of polyp from which malignant neoplasm originated
6736110 0000611 3	B62 0.00	Nodular lymphoma
6736810 0000611 8	B62 050 0	Nodular lymphoma of lymph nodes of inguinal region and lower limb
6765081 0000061 14		Transitional cell carcinoma of kidney
6765091 0000061 12		Infiltrating duct carcinoma of breast
6765101 0000061 18		Invasive duct carcinoma of breast
6765111 0000061 15		Infiltrating ductal carcinoma of breast
6765121 0000061 11		Invasive ductal carcinoma of breast
6765131 0000061 14		Adenocarcinoma of duodenum
6765141 0000061 16		Adenocarcinoma of large intestine

6765151000006119		Adenocarcinoma of liver
6765161000006117		Adenocarcinoma of stomach
6765171000006112		Cancer of stomach, adenocarcinoma
6765181000006110		Squamous cell carcinoma of epiglottis
6765191000006113		Squamous cell carcinoma of pharynx
6765201000006111		Cancer of the pharynx, squamous cell
6765431000006116		Polyneuropathy in collagen malignant disease
682691000006117	F396200	Myopathy due to malignant disease
6830821000006117		Breast cancer 1, early onset gene mutation positive
6830861000006111		Breast cancer 1, early onset gene mutation negative
6830901000006116		Breast cancer 2, early onset gene mutation positive
6830921000006114		Breast cancer 2, early onset gene mutation negative
683351000006111	B6y0.11	Myeloproliferative disease
683691000006113	B621500	Mycosis fungoides of lymph nodes of inguinal region and lower limb
6841411000006118		Adenocarcinoma of appendix
6841421000006114		Adenocarcinoma of caecum
6841431000006112		Adenocarcinoma of cecum
6841441000006119		Adenocarcinoma, no subtype, high grade
6841451000006117		Adenocarcinoma, no subtype, intermediate grade

6841461000006115		Adenocarcinoma, no subtype, low grade
6861721000006113		Malignant haematopoietic neoplasm
6861731000006111		Malignant hematopoietic neoplasm
6861741000006118		Malignant histiocytic neoplasm
6861751000006116		Malignant histiocytic neoplasm - category
6861761000006119		Malignant immunoproliferative neoplasm
6861771000006114		Malignant meningeal neoplasm
6864741000006116		Myeloid neoplasm
6869511000006111		History of malignant neoplasm of bronchus
6869521000006115		H/O: malignant neoplasm of bronchus
6869531000006117		Personal history of primary malignant neoplasm of bronchus
6869551000006112		H/O: malignant neoplasm of ear, nose AND/OR throat
6869561000006114		Personal history of primary malignant neoplasm of ear, nose AND/OR throat
6869581000006116		H/O: malignant neoplasm of female genital organ
6869591000006118		Personal history of primary malignant neoplasm of female genital organ
6869611000006112		H/O: malignant neoplasm of gastrointestinal tract
6869621000006116		Personal history of primary malignant neoplasm of gastrointestinal tract
6869641000006111		H/O: malignant neoplasm of kidney
6869651000006113		Personal history of primary malignant neoplasm of kidney

6869671 0000061 15		H/O: malignant neoplasm of lung
6869681 0000061 17		Personal history of primary malignant neoplasm of lung
6869691 0000061 19		History of malignant neoplasm of male genital organ
6869701 0000061 19		H/O: malignant neoplasm of male genital organ
6869711 0000061 16		Personal history of primary malignant neoplasm of male genital organ
6869731 0000061 10		H/O: malignant neoplasm of skin
6869741 0000061 17		Personal history of primary malignant neoplasm of skin
6869751 0000061 15		H/O: primary malignant neoplasm of skin
6869761 0000061 18		History of malignant neoplasm of trachea
6869771 0000061 13		H/O: malignant neoplasm of trachea
6869781 0000061 11		Personal history of primary malignant neoplasm of trachea
6869801 0000061 10		H/O: malignant neoplasm of bladder
6869811 0000061 13		Personal history of primary malignant neoplasm of urinary bladder
6869821 0000061 17		History of malignant neoplasm of urinary system
6869831 0000061 19		H/O: malignant neoplasm of urinary system
6869841 0000061 12		Personal history of primary malignant neoplasm of urinary system
6871551 0000061 15		Primary malignant neoplasm of ear, nose AND/OR throat
6881221 0000061 17		Surgical bile duct margin is closest uninvolved margin to malignant neoplasm
6881231 0000061 19		Surgical circumferential margin is closest uninvolved margin to malignant neoplasm

6881241 0000061 12		Surgical distal margin is closest uninvolved margin to malignant neoplasm
6881261 0000061 11		Surgical mesenteric margin is closest uninvolved margin to malignant neoplasm
6881271 0000061 16		Surgical omental margin is closest uninvolved margin to malignant neoplasm
6881281 0000061 18		Surgical pancreatic margin is closest uninvolved margin to malignant neoplasm
6881291 0000061 15		Surgical proximal margin is closest uninvolved margin to malignant neoplasm
6881661 0000061 14		T-cell AND/OR NK-cell neoplasm
6886531 0000061 13		Cervicovaginal cytology: High grade squamous intraepithelial lesion or carcinoma
6886541 0000061 15		Cervicovaginal cytology: HGSIL or carcinoma
6886551 0000061 18		Cervicovaginal cytology: HSIL or carcinoma
6886751 0000061 10		Malignant teratoma - category
6889811 0000061 11		Primary malignant clear cell tumour of ovary
6889821 0000061 15		Primary malignant clear cell tumor of ovary
6889831 0000061 17		Malignant mesonephroid tumor of ovary
6889841 0000061 10		Malignant mesonephroid tumour of ovary
6893061 0000061 15		Malignant glioma - category
6896091 0000061 17		Clear cell (mesonephric) neoplasm of ovary
6899081 0000061 11		Malignant medulloepithelioma of ciliary body
6899411 0000061 12		Carcinoma in situ of nasolacrimal duct
6901441 0000061 19		Gestational choriocarcinoma

6903201000006116		Hormone receptor positive malignant neoplasm of breast
6903211000006118		Glioma, uncertain whether benign or malignant
6905481000006111		Clear cell (mesonephroid) neoplasm - category
6906531000006119		Malignant teratoma of undescended testis
6908591000006119		Malignant teratoma of descended testis
6908771000006114		Gestational choriocarcinoma
6912741000006118		Surgical deep margin is closest uninvolved margin to ductal carcinoma in situ
6914341000006110		Surgical inferior margin involved by malignant neoplasm
6915221000006111		Surgical inferior margin involved by ductal carcinoma in situ
6915431000006113		Surgical superior margin is closest uninvolved margin to malignant neoplasm
6918671000006119		Surgical lateral margin is closest uninvolved margin to ductal carcinoma in situ
6920081000006117		Surgical lateral margin is closest uninvolved margin to malignant neoplasm
6920241000006118		Squamous cell carcinoma of mucous membrane of lower lip
6922661000006117		Secondary malignant neoplasm of lacrimal drainage structure
6922671000006112		Secondary malignant neoplasm of lacrimal duct
6922681000006110		Secondary malignant neoplasm of lacrimal drainage system
6924241000006113		Surgical medial margin is closest uninvolved margin to ductal carcinoma in situ
6924871000006116		Surgical medial margin is closest uninvolved margin to malignant neoplasm
6924921000006114		Surgical superior margin involved by malignant neoplasm

6928171000006117		Surgical deep margin involved by ductal carcinoma in situ
6928561000006115		Surgical deep margin is closest uninvolved margin to malignant neoplasm
6931041000006119		Malignant tumor of urinary system
6934201000006113		Squamous cell carcinoma of mucous membrane of upper lip
6938971000006116		Surgical medial margin involved by ductal carcinoma in situ
6939541000006113		Surgical inferior margin is closest uninvolved margin to ductal carcinoma in situ
6943731000006111		Surgical lateral margin involved by ductal carcinoma in situ
6943961000006116		Squamous cell carcinoma of oral mucous membrane
6944851000006112		Surgical superior margin is closest uninvolved margin to ductal carcinoma in situ
6948991000006116		Surgical superior margin involved by ductal carcinoma in situ
6949651000006112		Surgical medial margin involved by malignant neoplasm
6949781000006110		Surgical inferior margin is closest uninvolved margin to malignant neoplasm
6951661000006113		Follicular carcinoma, widely invasive
6955610000006118	B63..00	Multiple myeloma and immunoproliferative neoplasms
6955611000006112		Malignant lymphoma of the eye region
6960371000006112		Primary intraocular non-Hodgkin malignant lymphoma
6960401000006110		Intraocular non-Hodgkin malignant lymphoma
6968521000006115		Malignant tumor of vermilion border of lip
6971461000006114		Papillary carcinoma, macrofollicular

6975201000006114		Papillary carcinoma, clear cell
6975761000006116		Papillary carcinoma, Warthin-like
6976241000006119		Follicular carcinoma, clear cell
6980401000006112		Papillary carcinoma, diffuse follicular
6981471000006111		Papillary carcinoma, solid
6981481000006114		Papillary carcinoma, radiation-induced paediatric variant
6981491000006112		Papillary carcinoma, radiation-induced pediatric variant
6985131000006118		T-cell AND/OR NK-cell neoplasm
6985141000006111		T-cell AND/OR natural killer-cell neoplasm
6985711000006111		Papillary carcinoma, tall cell
6986331000006114		Papillary carcinoma, cribriform-morular
6987121000006112		Malignant neoplasm associated with AIDS
6987131000006110		Malignant neoplasm associated with acquired immunodeficiency syndrome
6988371000006111		Follicular carcinoma, grossly encapsulated with angioinvasion
6988581000006118		Carcinoma of colon, stage III
6988591000006115		Colon cancer, stage 3
6988601000006111		Cancer of colon, stage 3
6988631000006115		Squamous cell carcinoma of bridge of nose
6988641000006113		Cancer of the bridge of nose, squamous cell

6989101 0000061 12		Infiltrating ductal carcinoma of breast, stage 1
6990601 0000061 10		Infiltrating ductal carcinoma of breast, stage 2
6991611 0000061 18		Undifferentiated carcinoma of nasopharynx
6992091 0000061 12		Squamous cell carcinoma of chin
6992231 0000061 15		Carcinoma of colon, stage II
6992241 0000061 13		Colon cancer, stage 2
6992251 0000061 10		Cancer of the colon, stage 2
6992581 0000061 16		Squamous cell carcinoma of back
6992591 0000061 18		Cancer of the back, squamous cell
6992751 0000061 15		Carcinoma in situ of lacrimal drainage system
6994031 0000061 19		Squamous cell carcinoma of ala nasi
6994261 0000061 15		Squamous cell carcinoma of nasopharynx
6994951 0000061 10		Primary malignant neoplasm of peripheral nerve
6995301 0000061 13		Carcinoma of nasal meatus
6995311 0000061 11		Carcinoma of meatus nasi
6995321 0000061 15		Cancer of nasal meatus
6995331 0000061 17		Nasal meatus cancer
6995681 0000061 11		Ovarian cancer, disseminated
6995691 0000061 14		Primary malignant neoplasm of ovary, with widespread metastatic disease

6996671 0000061 19		Adenoid cystic carcinoma of salivary gland
6997841 0000061 14		Carcinoma in situ of lacrimal gland duct
6997851 0000061 11		Carcinoma in situ of lacrimal duct
6997961 0000061 17		pTis: Carcinoma in situ, intraepithelial (appendix)
6999311 0000061 14		Non-small cell carcinoma of lung, TNM stage 3
6999371 0000061 17		Location of malignant melanoma in specimen with involved lateral margin
6999571 0000061 12		Carcinoma of colon, stage IV
6999581 0000061 10		Colon cancer stage 4
6999961 0000061 11		Primary malignant neoplasm of lacrimal gland duct
7000641 0000061 18		Polymorphous low grade adenocarcinoma of salivary gland
7000851 0000061 15		Large cell carcinoma of lung, TNM stage 2
7000941 0000061 10		pTis: Carcinoma in situ, invasion of lamina propria (appendix)
7001801 0000061 10		Adenocarcinoma of nasopharynx
7001961 0000061 15		Infiltrating ductal carcinoma of breast, stage 3
7002061 0000061 14		Non-small cell carcinoma of lung, TNM stage 4
7002661 0000061 15		Hurthle cell carcinoma of thyroid
7002671 0000061 10		Cancer of thyroid, Hurthle cell
7003181 0000061 11		Adenoid cystic carcinoma of submandibular gland
7003191 0000061 14		Cancer of submandibular gland, adenoid cystic

7003281000006115		Primary malignant neoplasm of lacrimal drainage system
7004231000006118		Malignant blood vessel neoplasm, metastatic
7004611000006110		Carcinoma of ovary, stage 4
7004621000006119		Cancer of ovary, stage 4
7004631000006116		Ovarian cancer stage 4
7004761000006116		Malignant melanoma of skin of canthus of eye
7004841000006110		Squamous cell carcinoma of skin of neck
7004851000006112		Cancer of skin of neck, squamous cell
7005031000006110		Squamous cell carcinoma of lung, TNM stage 1
7005381000006114		Adenoid cystic carcinoma of oropharynx
7005501000006110		Basal cell carcinoma of external auditory canal
7005931000006114		Carcinoma of tip of nose
7005941000006116		Cancer of tip of nose
7006541000006116		Secondary malignant neoplasm of lacrimal gland duct
7007181000006118		Mucoepidermoid carcinoma of submandibular gland
7007191000006115		Cancer of submandibular gland, mucoepidermoid
7007201000006117		Malignant neoplasm of skin of eyelid
7007571000006114		Malignant melanoma of skin of lower eyelid
7007931000006116		Basal cell carcinoma of back

7007941000006114		Cancer of back, basal cell
7007951000006111		Squamous cell carcinoma of oropharynx
7007961000006113		Cancer of oropharynx, squamous cell
7008011000006113		Squamous cell carcinoma of lung, TNM stage 2
7008241000006119		Carcinoma of ovary, stage 3
7008251000006117		Ovarian cancer stage 3
7008261000006115		Cancer of ovary, stage 3
7008531000006110		Malignant melanoma of skin of upper eyelid
7008751000006112		Squamous cell carcinoma of external auditory canal
7009331000006110		Basal cell carcinoma of chest wall
7009341000006117		Cancer of chest wall, basal cell
7010391000006116		Adenocarcinoma carcinomatosis
7010481000006111		Large cell carcinoma of lung, TNM stage 4
7010621000006110		Adenocarcinoma of anus
7010761000006113		Adenoid cystic carcinoma of parotid gland
7011781000006113		Malignant melanoma of retina
7012071000006116		Malignant neoplasm of gum and contiguous sites
7012221000006114		Squamous cell carcinoma of auricle of ear
7012231000006112		Squamous cell carcinoma of pinna

7012241000006119		Squamous cell carcinoma of auricle
7012421000006113		Mucoepidermoid carcinoma of salivary gland
7013191000006111		Adenocarcinoma of pelvis
7014071000006111		Mucoepidermoid carcinoma of parotid gland
7014081000006114		Cancer of parotid gland, mucoepidermoid
7016111000006113		Squamous cell carcinoma of tip of nose
7016121000006117		Cancer of tip of nose, squamous cell
7017461000006117		Carcinoma of uterine cervix, invasive
7017471000006112		Cancer of uterine cervix, invasive
7017481000006110		Invasive cervical cancer
7017741000006113		Cancer of vulva, disseminated
7017751000006110		Primary malignant neoplasm of vulva, with widespread metastatic disease
7018991000006118		Small cell carcinoma carcinomatosis
7019001000006118		Small cell carcinomatosis
7019471000006119		Adenocarcinoma of lung, stage I
7020421000006116		Non-small cell carcinoma of lung, TNM stage 1
7021611000006119		Malignant melanoma of unknown origin
7021621000006110		Malignant melanoma of unknown primary
7022421000006115		Infiltrating ductal carcinoma of breast, stage 4

7022951000006118		Squamous cell carcinoma of nasolabial fold
7022961000006116		Cancer of nasolabial fold, squamous cell
7023221000006112		Malignant glioma of brainstem
7023751000006110		Malignant melanoma of skin of lower lip
7024321000006115		Malignant tumour of spinal cord, intramedullary
7024331000006117		Malignant tumor of spinal cord, intramedullary
7024351000006112		Cancer of spinal cord, intramedullary
7026441000006114		Adenocarcinoma of small intestine
7026721000006112		Adenocarcinoma of lung, stage III
7027271000006115		Carcinoma of ovary, stage 2
7027281000006117		Ovarian cancer stage 2
7027291000006119		Cancer of ovary, stage 2
7027301000006118		Malignant melanoma of skin of upper lip
7028481000006119		Malignant tumour of spinal cord, extramedullary
7028491000006116		Malignant tumor of spinal cord, extramedullary
7028501000006112		Cancer of spinal cord, extramedullary
7029491000006110		Carcinoma of ovary, stage 1
7029501000006119		Cancer of ovary, stage 1
7029511000006116		Ovarian cancer stage 1

7030141 0000061 16		Adenocarcinoma of lung, stage II
7030731 0000061 10		Basal cell carcinoma of auricle of ear
7030741 0000061 17		Basal cell carcinoma of pinna
7030751 0000061 15		Cancer of auricle of ear, basal cell
7031381 0000061 17		Location of malignant melanoma in specimen with uninvolved lateral margin
7032251 0000061 14		Signet-ring cells present, comprising less that 50% of malignant cells
7034801 0000061 16		Thyroid cancer metastatic to bone
7034821 0000061 14		Primary malignant neoplasm of thyroid gland, metastatic to bone
7035671 0000061 12		Large cell carcinoma of lung, TNM stage 1
7035751 0000061 12		Mucinous adenocarcinoma cells present, comprising less than 50% of malignant cells
7035831 0000061 13		Location of malignant melanoma in specimen with uninvolved deep margin
7035941 0000061 13		Undifferentiated large cell carcinomatosis
7036201 0000061 14		Large cell carcinoma of lung, TNM stage 3
7036651 0000061 11		Adenocarcinoma of lung, stage IV
7037531 0000061 18		Non-small cell carcinoma of lung, TNM stage 2
7037801 0000061 11		Carcinoma of urinary bladder, invasive
7037811 0000061 14		Invasive bladder cancer
7039021 0000061 15		Carcinoma ex pleomorphic adenoma of parotid gland
7039131 0000061 16		Location of malignant melanoma in specimen with involved deep margin

7039421 0000061 11		Squamous cell carcinoma of temple
7039431 0000061 14		Cancer of temple, squamous cell
7039961 0000061 10		Adenocarcinoma of rectosigmoid junction
7040541 0000061 14		Carcinoma of colon, stage I
7040551 0000061 11		Colon cancer stage 1
7040721 0000061 11		Malignant mixed tumour of salivary gland
7040731 0000061 14		Malignant mixed tumor of salivary gland
7040741 0000061 16		Cancer of salivary gland, mixed tumor
7040751 0000061 19		Cancer of salivary gland, mixed tumour
7040841 0000061 11		Squamous cell carcinoma of lung, TNM stage 3
7040851 0000061 13		Carcinoma of urinary bladder, superficial
7040861 0000061 10		Superficial bladder cancer
7042111 0000061 18		Squamous cell carcinomatosis
7043361 0000061 13		Squamous cell carcinoma of lung, TNM stage 4
7046911 0000061 15		Surgical bone margin closest to malignant neoplasm
7047110 0000611 9	B57 ..11	Metastases of respiratory and/or digestive systems
7052110 0000611 1	B23 2.00	Malignant mesothelioma of pleura
7066961 0000061 12		Epidermal growth factor receptor positive non-small cell lung cancer
7066971 0000061 17		Non-small cell lung cancer, positive for epidermal growth factor receptor expression

7066981 0000061 19		EGF-R positive non-small cell lung cancer
7066991 0000061 16		EGFR positive non-small cell lung cancer
7068051 0000061 10		Epidermal growth factor receptor negative non-small cell lung cancer
7068061 0000061 12		Non-small cell lung cancer, negative for epidermal growth factor receptor expression
7068071 0000061 17		EGFR negative non-small cell lung cancer
7068081 0000061 19		EGF-R negative non-small cell lung cancer
7069571 0000061 19		Malignant lymphoma in remission
7072091 0000061 14		Surgical soft tissue margin closest to malignant neoplasm
7074671 0000061 14		Hormone refractory prostate cancer
7074681 0000061 12		Castration-resistant prostate cancer
7074691 0000061 10		Castrate-resistant prostate cancer
7077551 0000061 11		HER2-positive carcinoma of breast
7079661 0000061 18		History of malignant neoplasm of vagina
7079671 0000061 13		History of cancer of vagina
7080811 0000061 15		History of malignant neoplasm of thymus
7082731 0000061 19		History of malignant basal cell neoplasm of skin
7082741 0000061 12		History of malignant basal cell tumor of skin
7082751 0000061 14		History of malignant basal cell tumour of skin
7082911 0000061 11		History of follicular adenocarcinoma of thyroid

7082921000006115		History of follicular thyroid carcinoma
7083431000006116		Malignant neoplasm of thoracic cavity structure
7083441000006114		Intrathoracic malignant neoplasm
7083751000006115		History of malignant neoplasm of mediastinum
7085791000006110		History of malignant neoplasm of prostate
7086121000006119		Malignant neoplasm of bone
7094361000006115		History of malignant neoplasm of small bowel
7094371000006110		History of malignant neoplasm of small intestine
7095011000006119		History of malignant neoplasm of thoracic cavity structure
7095021000006110		History of intrathoracic malignant neoplasm
7095041000006115		History of malignant neoplasm of penis
7095051000006118		History of cancer of the penis
7095911000006118		History of cancer of uterine body
7095941000006119		History of malignant neoplasm of uterine adnexa
7095951000006117		History of cancer of uterine adnexa
7096481000006114		History of choriocarcinoma of placenta
7097081000006117		History of malignant lymphoma
7097101000006113		History of medullary carcinoma of thyroid
7097111000006111		History of medullary thyroid carcinoma

7097221000006113		History of squamous cell carcinoma of skin
7097371000006117		Malignant neoplasm of cerebrum
7097601000006117		History of malignant neoplasm of skin
7097611000006119		H/O: malignant neoplasm of skin
7098041000006110		History of malignant neoplasm of rectum
7098081000006116		History of malignant neoplasm of breast
7098121000006119		History of malignant neoplasm of endometrium
7098141000006114		History of cancer of ovary
7098531000006113		Malignant basal cell neoplasm of skin
7098541000006115		Malignant basal cell tumor of skin
7098551000006118		Malignant basal cell tumour of skin
7098941000006111		History of malignant mesothelioma
7100131000006111		History of non-small cell malignant neoplasm of lung
7100141000006118		History of non-small cell cancer of the lung
7100441000006111		History of malignant neoplasm of bone
7100451000006113		History of cancer of the bone
7100551000006114		History of malignant neoplasm of thyroid
7100561000006111		History of papillary adenocarcinoma of thyroid
7100571000006116		History of papillary thyroid carcinoma

7102021 0000061 11		History of malignant neoplasm of pleura
7102061 0000061 17		History of malignant neoplasm of epididymis
7102851 0000061 10		History of malignant neoplasm of tongue
7102861 0000061 12		History of cancer of the tongue
7102871 0000061 17		History of malignant neoplasm of oesophagus
7102881 0000061 19		History of malignant neoplasm of esophagus
7102891 0000061 16		History of cancer of esophagus
7102901 0000061 17		History of cancer of oesophagus
7103381 0000061 18		History of malignant neoplasm of ureter
7104051 0000061 10		History of cancer of cervix
7106341 0000061 18		History of cancer of vulva
7107371 0000061 12		History of malignant neoplasm of colon
7123191 0000061 14		Malignant neoplasm of genital structure
7136971 0000061 18		Human epidermal growth factor 2 negative carcinoma of breast
7136981 0000061 15		Human epidermal growth factor 2 (HER2) negative carcinoma of breast
7140921 0000061 18		History of malignant neoplasm of endocrine gland
7140931 0000061 15		History of cancer of an endocrine gland
7140941 0000061 13		History of malignant neoplasm of head and/or neck
7143831 0000061 18		History of malignant neoplasm of nervous system

7143841 0000061 11		History of cancer of the nervous system
7151091 0000061 13		History of malignant mesothelioma of pleura
7157751 0000061 14		History of malignant neoplasm of spinal cord
7157761 0000061 11		History of cancer of the spinal cord
7168710 0000611 2	B67 3.00	Mast cell leukaemia
7169110 0000611 0	B62 650 0	Mast cell malignancy of lymph nodes of inguinal region and lower limb
7189481 0000061 13		History of malignant neoplasm of neck
7189491 0000061 11		History of cancer of the neck
7190210 0000611 1	B20 4.00	Malignant neoplasm of frontal sinus
7199310 0000611 3	B02 3.00	Malignant neoplasm, overlapping lesion of major salivary glands
7200010 0000611 9	B52 460 0	Malignant neoplasm, overlapping lesion of peripheral nerves and autonomic nervous system
7200110 0000611 6	B54 X.00	Malignant neoplasm-pluriglandular involvement,unspecified
7200210 0000611 2	B30 X.00	Malignant neoplasm/bones+articular cartilage/limb,unspfd
7200310 0000611 0	B30 W.0 0	Malignant neoplasm/overlap lesion/bone+articulr cartilage
7200410 0000611 7	B45 X.00	Malignant neoplasm/overlapping lesion/feml genital organs
7200510 0000611 5	B52 4X0 0	Malignant neoplasm/peripheral nerves of trunk,unspecified
7201610 0000611 9	B62 x30 0	Malignant reticuloendotheliosis
7203410 0000611 5	B30 2.00	Malignant neoplasm of vertebral column
7204510 0000611 3	B16 y.00	Malignant neoplasm other gallbladder/extrahepatic bile duct

72046100000611 0	B1zy.00	Malignant neoplasm other spec digestive tract and peritoneum
72047100000611 5	B33X.00	Malignant neoplasm overlapping lesion of skin
72048100000611 7	B542.00	Malignant neoplasm of pituitary gland and craniopharyngeal duct
72049100000611 9	B073200	Malignant tumour of posterior margin of nasal septum and choanae
72050100000611 0	B14z.00	Malignant neoplasm rectum,rectosigmoid junction and anus NOS
72051100000611 3	B332.00	Malignant neoplasm of skin of ear and external auricular canal
72052100000611 7	B333.00	Malignant neoplasm of skin of face
72053100000611 9	B333z00	Malignant neoplasm skin other and unspec part of face NOS
72056100000611 1	B309.00	Malignant neoplasm, overlapping lesion of bone and articular cartilage of limbs
72057100000611 6	B317.00	Malignant neoplasm, overlap lesion connective & soft tissue
72058100000611 8	B26..00	Primary malignant neoplasm of intrathoracic organs
72059100000611 5	B48y200	Malignant neoplasm, overlapping lesion of male genital organs
72063100000611 5	B51y200	Overlapping malignant neoplasm of brain
72076100000611 5	B030.00	Malignant neoplasm of upper gum
72079100000611 1	B002000	Malignant tumour of upper labial mucosa
72080100000611 2	B000000	Malignant tumour of vermilion border of lip
72095100000611 9	B497.00	Malignant neoplasm of urachus
72096100000611 7	B4A2.00	Malignant tumour of ureter
72099100000611 3	B4A3.00	Malignant neoplasm of urethra

7210410 0000611 1	B05 4.00	Malignant neoplasm of uvula
7210510 0000611 3	B45 0.00	Malignant tumour of vagina
7210910 0000611 9	B33 ..15	Malignant neoplasm of sweat gland
7211710 0000611 9	B47 ..00	Malignant tumour of testis
7212710 0000611 4	B24 0.00	Malignant neoplasm of thymus
7212910 0000611 0	B53 ..00	Malignant tumour of thyroid gland
7213110 0000611 4	B01 ..00	Malignant tumour of tongue
7213510 0000611 0	B06 0.00	Malignant tumour of tonsil
7213610 0000611 2	B06 1.00	Malignant neoplasm of tonsillar fossa
7213910 0000611 6	B22 0.00	Malignant neoplasm of trachea
7214210 0000611 2	B13 1.00	Malignant tumour of transverse colon
7214810 0000611 1	B06 3.00	Malignant neoplasm of vallecula
7216010 0000611 7	B12 ..00	Malignant tumour of small intestine
7216210 0000611 0	B05 3.00	Malignant neoplasm of soft palate
7216351 0000061 16		Siewert type I adenocarcinoma of oesophagogastric junction
7216361 0000061 19		Siewert type I adenocarcinoma of esophagogastric junction
7217010 0000611 2	B48 5.00	Malignant neoplasm of spermatic cord
7217210 0000611 9	B20 5.00	Malignant neoplasm of sphenoidal sinus
7217510 0000611 1	B52 2.00	Malignant neoplasm of spinal cord

7218210 0000611 4	B13 7.00	Malignant neoplasm of splenic flexure of colon
7218510 0000611 7	B11 ..00	Malignant tumour of stomach
7218710 0000611 0	B21 2.00	Malignant neoplasm of subglottis
7218810 0000611 3	B02 2.00	Malignant neoplasm of sublingual gland
7219610 0000611 1	B20 020 0	Malignant neoplasm of septum of nose
7219910 0000611 5	B13 3.00	Malignant tumour of sigmoid colon
7220541 0000061 16		Excision of malignant neoplasm of mandible
7223410 0000611 9	B11 100 0	Malignant neoplasm of prepylorus of stomach
7223601 0000061 12		Siewert type III adenocarcinoma of oesophagogastric junction
7223610 0000611 5	B46 ..00	Malignant tumour of prostate
7223611 0000061 10		Siewert type III adenocarcinoma of esophagogastric junction
7224210 0000611 5	B08 1.00	Malignant neoplasm of pyriform sinus
7224410 0000611 0	B14 0.00	Malignant tumour of rectosigmoid junction
7224710 0000611 9	B4A 100 0	Malignant neoplasm of renal calyces
7224810 0000611 6	B4A 1.00	Malignant tumour of renal pelvis
7225110 0000611 2	B47 010 0	Malignant tumour of undescended testis
7225210 0000611 6	B50 5.00	Malignant neoplasm of retina
7225410 0000611 1	B05 6.00	Malignant neoplasm of retromolar area
7227610 0000611 9	B55 3.00	Malignant neoplasm of pelvis

7227641 0000061 19		Cervical Papanicolaou smear positive for malignant neoplasm
7228710 0000611 7	B52 420 0	Malignant neoplasm of peripheral nerves of lower limb, including hip
7229010 0000611 7	B52 410 0	Malignant neoplasm of peripheral nerves of upper limb, including shoulder
7229110 0000611 9	B52 400 0	Malignant neoplasm of peripheral nerves of head, face and neck
7229201 0000061 12		Anal Papanicolaou smear positive for malignant neoplasm
7229610 0000611 6	B54 3.00	Malignant neoplasm of pineal gland
7229810 0000611 4	B54 200 0	Malignant neoplasm of pituitary gland
7230010 0000611 3	B23 ..00	Malignant neoplasm of pleura
7230310 0000611 7	B08 0.00	Malignant neoplasm of postcricoid region
7231110 0000611 0	B48 6.00	Malignant neoplasm of scrotum
7232210 0000611 2	B12 y.00	Malignant neoplasm of other specified site small intestine
7232810 0000611 1	B44 0.00	Malignant tumour of ovary
7233010 0000611 0	B22 5.00	Malignant neoplasm of overlapping lesion of bronchus and lung
7233210 0000611 7	B06 020 0	Overlapping malignant neoplasm of tonsil
7233351 0000061 16		Nonsquamous nonsmall cell neoplasm of lung
7233410 0000611 2	B45 y00 0	Overlapping malignant neoplasm of vulva
7233910 0000611 5	B06 230 0	Malignant neoplasm of palatopharyngeal arch
7234010 0000611 8	B17 ..00	Malignant tumour of pancreas
7234101 0000061 15		Destruction of malignant neoplasm of rectum using cryosurgery

7234371000006111		Destruction of malignant neoplasm of breast using microwave phased array thermoprobe
72344100000611 6	B541.00	Malignant neoplasm of parathyroid gland
72345100000611 9	B4A4.00	Malignant neoplasm of paraurethral glands
72350100000611 7	B020.00	Malignant tumour of parotid gland
72354100000611 5	B501.00	Malignant neoplasm of orbit
72357100000611 1	B06..00	Malignant tumour of oropharynx
72360100000611 6	B55z.00	Malignant neoplasm of other and ill defined site NOS
72364100000611 9	B5y..00	Malignant neoplasm of other and unspecified site OS
72380100000611 5	B0zy.00	Malignant neoplasm of other sites lip, oral cavity, pharynx
7238321000006118		Siewert type II adenocarcinoma of oesophagogastric junction
7238331000006115		Siewert type II adenocarcinoma of esophagogastric junction
7238691000006112		Primary small cell neoplasm of thymus
7238711000006110		Primary squamous cell carcinoma of naris
72395100000611 3	B308.11	Malignant neoplasm of metatarsal bones of foot
72405100000611 0	B241200	Malignant neoplasm of myocardium
72431100000611 1	B031.00	Malignant neoplasm of lower gum
72440100000611 6	B003200	Malignant tumour of lower labial mucosa
72441100000611 8	B003300	Malig neoplasm of lower lip, oral aspect
72442100000611 4	B001.00	Malignant neoplasm of lower lip, vermilion border

72462100000611 2	B20 130 0	Malignant neoplasm of mastoid air cells
72464100000611 7	B20 2.00	Malignant neoplasm of maxillary sinus
72467100000611 3	B24 X.00	Malignant neoplasm of mediastinum, part unspecified
72468100000611 1	B52 X.00	Malignant neoplasm of meninges, unspecified
72472100000611 6	B50 7.00	Malignant neoplasm of lacrimal duct
72476100000611 0	B21 3.00	Malignant neoplasm of laryngeal cartilage
72478100000611 7	B0z 2.00	Malignant neoplasm of laryngopharynx
72479100000611 9	B21 ..00	Malignant tumour of larynx
72484100000611 6	B07 2.00	Malignant neoplasm of lateral wall of nasopharynx
72488100000611 0	B01 6.00	Malignant neoplasm of lingual tonsil
72489100000611 3	B00 ..00	Malignant neoplasm of lip
72490100000611 2	B00 400 0	Malignant neoplasm of lip unspecified, buccal aspect
72510100000611 1	B05 2.00	Malignant neoplasm of hard palate
72512100000611 8	B17 0.00	Malignant tumour of head of pancreas
72513510000061 19		Adenocarcinoma of head and neck
72515100000611 0	B24 1.00	Malignant neoplasm of heart
72519100000611 6	B13 0.00	Malignant tumour of hepatic flexure
72522100000611 1	B6.. .11	Malignant neoplasm of histiocytic tissue
72524100000611 6	B08 ..00	Malignant neoplasm of hypopharynx

7252710 0000611 2	B12 2.00	Malignant tumour of ileum
7254510 0000611 8	B12 1.00	Malignant neoplasm of jejunum
7255821 0000061 17		Malignant catatonia
7256110 0000611 7	B04 ..00	Malignant neoplasm of floor of mouth
7257110 0000611 3	B16 0.00	Malignant tumour of gallbladder
7257610 0000611 1	B4y. .00	Malignant neoplasm of genitourinary organ OS
7257710 0000611 6	B48 1.00	Malignant neoplasm of glans penis
7258110 0000611 6	B06 210 0	Malignant tumour of anterior pillar of fauces
7258610 0000611 8	B03 ..00	Malignant neoplasm of gum
7259331 0000061 13		Thrombophilia due to malignant neoplasm
7259510 0000611 2	B13 2.00	Malignant tumour of descending colon
7260210 0000611 0	B12 0.00	Malignant tumour of duodenum
7260710 0000611 1	B24 100 0	Malignant neoplasm of endocardium
7260711 0000061 19		Papillary neoplasm
7261410 0000611 6	B48 4.00	Malignant neoplasm of epididymis
7262010 0000611 1	B16 1.00	Malignant tumour of extrahepatic bile duct
7262610 0000611 2	B33 1.00	Malignant tumour of eyelid
7262710 0000611 7	B44 1.00	Malignant neoplasm of fallopian tube
7263310 0000611 1	B13 ..00	Malignant neoplasm of colon

7263510 0000611 6	B00 5.00	Malignant neoplasm of commissure of lip
7263610 0000611 9	B16 120 0	Malignant neoplasm of common bile duct
7263710 0000611 4	B50 3.00	Malignant neoplasm of conjunctiva
7265610 0000611 4	B50 4.00	Malignant neoplasm of cornea
7266310 0000611 8	B30 340 0	Malignant neoplasm of costo-vertebral joint
7266410 0000611 1	B52 0.00	Malignant neoplasm of cranial nerves
7271510 0000611 7	B05 0.00	Malignant neoplasm of cheek mucosa
7271810 0000611 3	B50 6.00	Malignant neoplasm of choroid
7272621 0000061 10		Malignant neoplasm of skin of face
7272810 0000611 7	B13 5.00	Malignant neoplasm of appendix
7273210 0000611 1	B13 6.00	Malignant tumour of ascending colon
7273810 0000611 0	B01 0.00	Malignant tumour of base of tongue
7274511 0000061 10		Malignant fibromatous neoplasm
7274691 0000061 18		Oxyphilic adenocarcinoma
7275110 0000611 4	B51 7.00	Malignant neoplasm of brainstem
7275210 0000611 8	B51 7z0 0	Malignant neoplasm of brain stem NOS
7275310 0000611 5	B06 y00 0	Malignant neoplasm of branchial cleft
7275610 0000611 2	B05 0.11	Malignant neoplasm of buccal mucosa
7277310 0000611 3	B32 6.00	Malignant melanoma of upper limb

7277551 0000061 17		Malignant fibrous histiocytoma
7277610 0000611 6	B08 2.00	Malignant neoplasm of aryepiglottic fold, hypopharyngeal aspect
7277710 0000611 1	B51 0.00	Malignant neoplasm of cerebrum (excluding lobes and ventricles)
7277810 0000611 4	B16 ..00	Malignant neoplasm gallbladder and extrahepatic bile ducts
7277910 0000611 2	B16 z.00	Malignant neoplasm gallbladder/extrahepatic bile ducts NOS
7278110 0000611 1	B6y. .00	Malignant neoplasm lymphatic or haematopoietic tissue OS
7278231 0000061 13		Malignant neoplasm of anorectum
7278241 0000061 15		Anorectal cancer
7278381 0000061 16		Metastatic malignant melanoma
7278410 0000611 0	B52 020 0	Malignant neoplasm of acoustic nerve
7279110 0000611 6	B16 2.00	Malignant neoplasm of ampulla of Vater
7280821 0000061 17		Malignant neoplasm of ear
7282010 0000611 0	B32 1.00	Malignant melanoma of eyelid
7282591 0000061 17		Lobular carcinoma in situ with microinvasion
7282711 0000061 15		Invasion of neoplasm to visceral peritoneum
7284510 0000611 0	B31 530 0	Malignant neoplasm of connective and soft tissue of sacrum or coccyx
7284610 0000611 2	B52 4.00	Malig neopl peripheral nerves and autonomic nervous system
7284710 0000611 7	B52 W.0 0	Malig neopl, overlap lesion brain & other part of CNS
7284810 0000611 9	B31 410 0	Malignant neoplasm of connective and soft tissues of lumbar spine

7284881 0000061 13		History of malignant neoplasm of skin excluding melanoma
7284910 0000611 6	B31 330 0	Malignant neoplasm of connective and soft tissues of thoracic spine
7285291 0000061 11		Local excision of malignant neoplasm of stomach
7285301 0000061 12		Local excision of malignant tumor of stomach
7285311 0000061 10		Local excision of malignant tumour of stomach
7285551 0000061 15		Ductal carcinoma in situ with microinvasion
7285710 0000611 8	B62 310 0	Malignant histiocytosis of lymph nodes of head, face and neck
7285810 0000611 5	B62 350 0	Malignant histiocytosis of lymph nodes of inguinal region and lower limb
7285910 0000611 7	B62 340 0	Malignant histiocytosis of lymph nodes of axilla and upper limb
7285921 0000061 18		Malignant adenomatous neoplasm
7285931 0000061 15		Adenocarcinoma
7286381 0000061 10		Cribriform neoplasm pattern
7287210 0000611 1	B62 y.00	Malignant lymphoma
7287381 0000061 13		Infiltrating carcinoma with ductal and lobular features
7287391 0000061 11		Invasive carcinoma with ductal and lobular features (Mixed type carcinoma)
7287561 0000061 10		Thoracoscopic excision of neoplasm of pericardium
7287710 0000611 2	B62 y50 0	Malignant lymphoma of lymph nodes of inguinal region AND/OR lower limb
7287810 0000611 0	B62 y40 0	Malignant lymphoma of lymph nodes of axilla AND/OR upper limb
7288581 0000061 18		Ductal carcinoma in situ with microinvasion and involving nipple skin

7288591000006115		Paget disease and ductal carcinoma in situ with microinvasion
7289101000006112		Invasion of skeletal muscle by carcinoma
7291431000006114		Synchronous primary carcinomas
72928100000006113	B31y.00	Malig neop connective and soft tissue other specified site
72929100000006111	B311.00	Malignant neoplasm of connective and soft tissue of upper limb and shoulder
72931100000006110	B500.00	Malignant neoplasm of eyeball excluding conjunctiva, cornea, retina and choroid
72932100000006119	B20..00	Malignant neoplasm of nasal cavities, middle ear and accessory sinuses
72933100000006116	B3...00	Malignant neoplasm of bone, connective tissue, skin and breast
72934100000006114	B3z..00	Malig neop of bone, connective tissue, skin and breast NOS
72935100000006111	B3y..00	Malig neop of bone, connective tissue, skin and breast OS
72936100000006113	B310.00	Malig neop of connective and soft tissue head, face and neck
72937100000006118	B314z00	Malig neop of connective and soft tissue of abdomen NOS
72938100000006115	B314000	Malignant neoplasm of connective and soft tissue of abdominal wall
72939100000006117	B312600	Malignant neoplasm of connective and soft tissue of great toe
72940100000006115	B312.00	Malignant neoplasm of connective and soft tissue of hip and lower limb
72941100000006117	B315100	Malignant neoplasm of connective and soft tissue of inguinal region
72942100000006113	B312300	Malignant neoplasm of connective and soft tissue of lower leg
72943100000006111	B315z00	Malig neop of connective and soft tissue of pelvis NOS
72944100000006118	B313z00	Malig neop of connective and soft tissue of thorax NOS

72945100000611 6	B31 210 0	Malignant neoplasm of connective and soft tissue of thigh and upper leg
72946100000611 9	B31 6.00	Malignant neoplasm of connective and soft tissues of trunk
72947100000611 4	B54 z.00	Malig neop of endocrine gland or related structure NOS
72948100000611 2	B4A ..00	Malignant tumour of urinary system
7294901000006111		Malignant neoplasm detection during interval between recommended screening examinations
72949100000611 0	B45 ..00	Malig neop of other and unspecified female genital organs
7294911000006114		Interval cancer
7294931000006115		Pleomorphic lobular carcinoma in situ
72950100000611 9	B52 ..00	Malig neop of other and unspecified parts of nervous system
7295011000006114		Malignant thymoma
72951100000611 6	B54 ..00	Malig neop of other endocrine glands and related structures
7295111000006113		Mixed ductal and lobular carcinoma of breast
7295121000006117		Carcinoma of breast with ductal and lobular features
72952100000611 2	B24 y.00	Malig neop of other site of heart, thymus and mediastinum
72953100000611 0	B2.. .00	Malignant neoplasm of thoracic cavity structure
72954100000611 7	B30 4z0 0	Malig neop of scapula and long bones of upper arm NOS
72955100000611 5	B2z 0.00	Malig neop of upper respiratory tract, part unspecified
72956100000611 8	B1z. .00	Malig neop oth/ill-defined sites digestive tract/peritoneum
72957100000611 3	B20 y.00	Malig neop other site nasal cavity, middle ear and sinuses

729581000006111	B14y.00	Malig neop other site rectum, rectosigmoid junction and anus
729591000006114	B0z..00	Malig neop other/ill-defined sites lip, oral cavity, pharynx
729601000006118	B2z..00	Malig neop other/ill-defined sites resp/intrathoracic organs
7296481000006119		Apocrine intraductal carcinoma
7296491000006116		Apocrine ductal carcinoma in situ
7296621000006115		Surgical procedure on primary malignant neoplasm of breast
7296771000006112		Mucinous carcinoma of breast
7297061000006118		Clinging intraductal carcinoma
7297071000006113		Clinging ductal carcinoma in situ
7297141000006118		Classic lobular carcinoma in situ
7300001000006111		Percentage of carcinoma in situ in neoplasm
730001000006117	B25..00	Malignant neoplasm, overlapping lesion of heart, mediastinum and pleura
730011000006119	B201.00	Malignant neoplasm of auditory tube, middle ear and mastoid air cells
730021000006110	B201z00	Malignant neoplasm of auditory tube, middle ear and mastoid air cells
730031000006113	B310z00	Malig neop connective and soft tissue head, face, neck NOS
730041000006115	B542z00	Malig neop pituitary gland or craniopharyngeal duct NOS
730241000006113	B312z00	Malig neop connective and soft tissue hip and leg NOS
730251000006110	B31220 0	Malignant neoplasm of connective and soft tissue of popliteal space
7302961000006114		T4d: Inflammatory carcinoma of breast with involvement of skin and chest wall by carcinoma

73049100000611 7	B52 4W 00	Mal neoplasm/periph nerves+autonomic nervous system,unspc
7305001 0000061 18		Malignant carcinoid tumour
7305011 0000061 15		Malignant carcinoid tumor
7316210 0000611 3	B67 y00 0	Lymphosarcoma cell leukaemia
7316910 0000611 0	B60 150 0	Lymphosarcoma of lymph nodes of inguinal region and lower limb
7317761 0000061 11		Carcinoma of uterus
7317771 0000061 16		Malignant epithelial neoplasm of uterus
7319331 0000061 11		Excision of carcinoma of nose
7319341 0000061 18		Excision of malignant epithelial neoplasm of nose
7320611 0000061 13		Primary malignant neoplasm of extrahepatic bile duct
7324410 0000611 3	B56 ..11	Lymph node metastases
7330371 0000061 13		Primary malignant neoplasm of perihilar bile duct
7333541 0000061 17		Mixed serous and mucinous cystadenocarcinoma
7333710 0000611 9	B22 z.11	Lung cancer
7334331 0000061 19		Partial excision of neoplasm of spinal cord
7334851 0000061 16		Primary malignant neoplasm of intrahepatic bile duct
7335851 0000061 17		Excision of contiguous neoplasm of extradural and intradural space of spine
7336861 0000061 13		Myelodysplastic/myeloproliferative neoplasm, unclassifiable
7339561 0000061 15		Primary malignant neoplasm of distal bile duct

7342971000006112		Myelodysplastic/myeloproliferative neoplasm, unclassifiable
7344821000006112		Malignant germ cell neoplasm of posterior mediastinum
7344851000006115		Malignant germ cell neoplasm of anterior mediastinum
7344891000006114		Follicular neoplasm of thyroid
7344901000006113		Malignant melanoma of skin of anus
7345581000006114		Endocervical adenocarcinoma in situ
7345801000006115		Carcinoma of peritoneum
7345811000006117		Malignant epithelial neoplasm of peritoneum
7345821000006113		Carcinoma of female breast
7345831000006111		Malignant epithelial neoplasm of female breast
7346021000006111		Adenocarcinoma of scrotum
7347011000006118		Carcinoma of vulva
7347021000006114		Malignant epithelial neoplasm of vulva
7347041000006119		Cancer of carotid body
7347071000006110		Anorectal adenocarcinoma
7347081000006113		Adenocarcinoma of anorectum
7347901000006115		Carcinoma of cheek
7347911000006117		Malignant epithelial neoplasm of cheek
7350801000006110		Squamous cell carcinoma arising in chronic ulcer

7351551000006117		Malignant epithelial neoplasm of oropharynx
7351561000006115		Carcinoma of renal pelvis
7351571000006110		Malignant epithelial neoplasm of renal pelvis
7351581000006113		Carcinoma of thyroid
7351591000006111		Malignant epithelial neoplasm of thyroid
7351621000006113		Malignant neoplasm of cerebellopontine angle
7351631000006111		Cancer of cerebellopontine angle
7351931000006117		Cancer of urinary organ
7352111000006119		Malignant neoplasm of axial suprasellar region of brain
7352131000006113		Malignant teratoma of pineal region
7352441000006113		Malignant melanoma of skin of scrotum
7352451000006110		Malignant neoplasm of connective tissue
7352781000006110		Malignant melanoma of skin of penis
7352791000006113		Malignant neoplasm of mastoid
7352801000006114		Malignant melanoma of skin of vulva
7352971000006114		Carcinoma of spinal cord
7352981000006112		Malignant epithelial neoplasm of spinal cord
7352991000006110		Carcinoma of anus
7353001000006111		Malignant epithelial neoplasm of anus

7354451 0000061 15		Choriocarcinoma of placenta
7354511 0000061 15		Grade III squamous intraepithelial neoplasia with microinvasive squamous cell carcinoma
7356051 0000061 16		Transglottic malignant neoplasm of larynx
7356061 0000061 19		Transglottic cancer of larynx
7356601 0000061 12		Combined large cell neuroendocrine carcinoma
7356731 0000061 13		Malignant neoplasm of maxillofacial bone
7356741 0000061 15		Malignant teratoma of retroperitoneum
7358291 0000061 18		Carcinoma of small intestine
7358301 0000061 17		Carcinoma of small bowel
7358311 0000061 19		Malignant epithelial neoplasm of small intestine
7358321 0000061 10		Malignant epithelial neoplasm of hypopharynx
7358381 0000061 14		Cancer of mandible
7358391 0000061 12		Malignant neoplasm of soft tissue of orbit
7358401 0000061 14		Cancer of soft tissue of orbit
7358421 0000061 16		Cancer of posterior mediastinum
7358481 0000061 17		Cancer of parametrium
7358491 0000061 19		Malignant neoplasm of digestive system
7358501 0000061 10		Cancer of digestive system
7361161 0000061 17		Carcinoma of pineal gland

7361171000006112		Carcinoma of pineal body
7361181000006110		Malignant epithelial neoplasm of pineal gland
7361191000006113		Carcinoma of ureter
7361201000006111		Malignant epithelial neoplasm of ureter
7361281000006119		Cancer of lateral wall of oropharynx
7361431000006116		Malignant neoplasm of intraabdominal organ
7361441000006114		Cancer of intraabdominal organ
7362061000006114		Squamous cell carcinoma of nose
7362071000006119		Squamous cell carcinoma of shoulder
7362291000006114		Infiltrating duct carcinoma of female breast
7362301000006110		Invasive ductal carcinoma of female breast
7362321000006117		Carcinoma of urethra
7362331000006119		Malignant epithelial neoplasm of urethra
7362851000006116		Carcinoma of nose
7362861000006119		Malignant epithelial neoplasm of nose
7362871000006114		Carcinoma of brain
7362881000006112		Malignant epithelial neoplasm of brain
7362891000006110		Carcinoma of nasal cavity
7362901000006114		Malignant epithelial neoplasm of nasal cavity

7362931 0000061 18		Carcinoma of appendix
7362941 0000061 11		Malignant epithelial neoplasm of appendix
7362951 0000061 13		Carcinoma of lung
7362961 0000061 10		Malignant epithelial neoplasm of lung
7362971 0000061 15		Carcinoma of upper rectum
7362981 0000061 17		Malignant epithelial neoplasm of upper rectum
7363581 0000061 17		Malignant neoplasm of anterior and lateral floor of mouth
7363591 0000061 19		Cancer of anterior and lateral floor of mouth
7363901 0000061 17		Carcinoma of fundus of uterus
7363911 0000061 19		Malignant epithelial neoplasm of fundus of uterus
7364151 0000061 17		Malignant neoplasm of upper respiratory tract
7364161 0000061 15		Cancer of upper respiratory tract
7364181 0000061 13		Cancer of parietal pleura
7364291 0000061 19		Carcinoma of corpus uteri
7364301 0000061 18		Malignant epithelial neoplasm of body of uterus
7364381 0000061 10		Carcinoma of maxilla
7364391 0000061 13		Malignant epithelial neoplasm of maxilla
7364661 0000061 13		Cancer of respiratory system
7365431 0000061 18		Adenocarcinoma of lower oesophagus

7365441000006111		Adenocarcinoma of lower esophagus
7365491000006119		Carcinoma of floor of mouth
7365501000006110		Malignant epithelial neoplasm of floor of mouth
7366481000006111		Carcinoma of face
7366491000006114		Malignant epithelial neoplasm of face
7366721000006119		Malignant neoplasm of alveolus of maxilla
7366731000006116		Cancer of maxillary alveolus
7366751000006111		Cancer of anterior mediastinum
7367041000006113		Nasopharyngeal carcinoma
7367141000006112		Carcinoma of hypothalamus
7367151000006114		Malignant epithelial neoplasm of hypothalamus
7367161000006111		Carcinoma of pharynx
7367171000006116		Malignant epithelial neoplasm of pharynx
7367261000006116		Malignant neoplasm of broad ligament of uterus
7367271000006111		Cancer of broad ligament of uterus
7367281000006114		Malignant neoplasm of alveolus dentalis
7367291000006112		Cancer of alveolus dentalis
7368081000006118		Cancer of visceral pleura
7368091000006115		Malignant neoplasm of skin of scrotum

7368101 0000061 14		Cancer of scrotal skin
7368421 0000061 13		Papillary and solid transitional cell carcinoma
7368431 0000061 11		Transitional carcinoma with mixed papillary and solid growth pattern
7369071 0000061 10		Cancer of pelvic peritoneum
7369601 0000061 13		Carcinoma of skin of anus
7369611 0000061 11		Malignant epithelial neoplasm of skin of anus
7369621 0000061 15		Carcinoma of nasal septum
7369631 0000061 17		Malignant epithelial neoplasm of nasal septum
7369701 0000061 18		Cancer of cerebellum
7370491 0000061 19		Malignant epithelial neoplasm of alveolus dentalis
7370501 0000061 10		Carcinoma of alveolus dentalis
7370781 0000061 13		Carcinoma of mandible
7370791 0000061 11		Malignant epithelial neoplasm of mandible
7372311 0000061 18		Malignant neoplasm of alveolus of mandible
7372321 0000061 14		Cancer of mandibular alveolus
7373061 0000061 14		Malignant neoplasm of long bone of lower limb
7373071 0000061 19		Malignant neoplasm of long bone of lower leg
7373101 0000061 12		Secondary malignant neoplasm of skin of lower leg
7373111 0000061 10		Secondary malignant neoplasm of skin of upper arm

7373121 0000061 19		Secondary malignant neoplasm of lower leg
7373131 0000061 16		Secondary malignant neoplasm of upper arm
7373141 0000061 14		Primary malignant neoplasm of skin of lower leg
7373151 0000061 11		Primary malignant neoplasm of lower leg
7373161 0000061 13		Malignant melanoma of skin of lower leg
7373171 0000061 18		Malignant melanoma of skin of upper arm
7374731 0000061 18		Undifferentiated nonkeratinizing squamous cell carcinoma
7375071 0000061 18		Carcinoma in situ of dome of urinary bladder
7375081 0000061 15		Primary malignant neoplasm of dome of urinary bladder
7378671 0000061 19		Excision of squamous cell carcinoma
7380901 0000061 11		Primary malignant adenomatous neoplasm
7384741 0000061 18		Non-invasive pancreatobiliary neoplasm
7384751 0000061 16		Papillary neoplasm, pancreatobiliary-type, with high grade intraepithelial neoplasia
7384761 0000061 19		Pancreatobiliary-type carcinoma
7384771 0000061 14		Adenocarcinoma, pancreatobiliary-type
7384781 0000061 12		Micropapillary carcinoma
7384801 0000061 11		Mixed acinar-ductal carcinoma
7385491 0000061 17		Myeloid or lymphoid neoplasm with alpha-type platelet-derived growth factor receptor gene rearrangement
7385501 0000061 13		Myeloid or lymphoid neoplasm with PDGFRA rearrangement

7385511 0000061 11		Myeloid and lymphoid neoplasms with PDGFRA rearrangement
7385521 0000061 15		Myeloid neoplasm with beta-type platelet-derived growth factor receptor gene rearrangement
7385531 0000061 17		Myeloid neoplasm with PDGFRB rearrangement
7385541 0000061 10		Myeloid or lymphoid neoplasm with fibroblast growth factor receptor 1 abnormality
7385551 0000061 12		Myeloid or lymphoid neoplasm with FGFR1 abnormality
7385651 0000061 13		Serrated adenocarcinoma
7385841 0000061 12		Malignant lymphoma, diffuse large B-cell, immunoblastic
7398010 0000611 5	B57 7.11	Metastasis to liver
7450010 0000611 4	B62 4.00	Leukaemic reticuloendotheliosis
7450110 0000611 2	B62 4.11	Leukaemic reticuloendotheliosis
7459710 0000611 6	B62 5.00	Letterer-Siwe disease (clinical)
7463010 0000611 7	B62 430 0	Leukaemic reticuloendotheliosis of intra-abdominal lymph nodes
7482041 0000061 18		History of malignant neoplasm of brain
7482051 0000061 16		History of malignant tumor of brain
7482061 0000061 19		History of malignant tumour of brain
7482081 0000061 12		History of malignant neoplasm of stomach
7482091 0000061 10		History of malignant tumour of stomach
7482101 0000061 16		History of malignant tumor of stomach
7482111 0000061 18		History of recurrent malignant neoplasm of breast

7482121 0000061 14		History of recurrent malignant tumor of breast
7482131 0000061 12		History of recurrent malignant tumour of breast
7482141 0000061 19		History of malignant neoplasm of oral cavity
7482151 0000061 17		History of malignant tumor of oral cavity
7482161 0000061 15		History of malignant tumour of oral cavity
7482171 0000061 10		History of cancer of oral cavity
7482181 0000061 13		History of oral cancer
7482191 0000061 11		H/O carcinoma of larynx
7482201 0000061 14		History of malignant tumour of larynx
7482211 0000061 12		History of malignant neoplasm of larynx
7482221 0000061 16		History of malignant tumor of larynx
7482241 0000061 11		History of malignant neoplasm of testis
7482251 0000061 13		History of malignant tumour of testis
7482261 0000061 10		History of malignant tumor of testis
7482271 0000061 15		History of malignant neoplasm of liver
7482281 0000061 17		History of malignant tumor of liver
7482291 0000061 19		History of malignant tumour of liver
7484901 0000061 14		Bilateral oophorectomy for malignant neoplasm of ovary
7484921 0000061 16		Bilateral oophorectomy for malignant tumor of ovary

7484931 0000061 18		Bilateral oophorectomy for malignant tumour of ovary
7487881 0000061 18		Intraductal papillary mucinous neoplasm with low grade dysplasia
7487891 0000061 15		Intraductal papillary mucinous carcinoma in situ of pancreas
7487901 0000061 16		Intraductal papillary mucinous neoplasm with high grade dysplasia
7495001 0000061 10		Magnetic resonance imaging of breast for screening for malignant neoplasm
7502931 0000061 15		Undifferentiated carcinoma of nasal sinus
7502941 0000061 13		SNUC - sinonasal undifferentiated carcinoma
7502951 0000061 10		Sinonasal undifferentiated carcinoma
7503201 0000061 14		Keratinising squamous cell carcinoma of nasopharynx
7503211 0000061 12		Keratinizing squamous cell carcinoma of nasopharynx
7503701 0000061 17		Malignant melanoma of nasal cavity
7503711 0000061 19		Malignant melanoma of vestibule of mouth
7503721 0000061 10		Malignant melanoma of tongue
7503731 0000061 13		Malignant melanoma of floor of mouth
7503741 0000061 15		Malignant melanoma of palatine arch
7503751 0000061 18		Malignant melanoma of tonsillar pillar
7503761 0000061 16		Malignant melanoma of buccal mucosa
7503791 0000061 12		Undifferentiated nonkeratinizing carcinoma of nasopharynx
7503801 0000061 13		Nasopharyngeal carcinoma type 2b

750381100000061 11		Undifferentiated nonkeratinising carcinoma of nasopharynx
750382100000061 15		Undifferentiated nonkeratinizing squamous cell carcinoma of nasopharynx
750383100000061 17		Nasopharyngeal carcinoma type 3
750661100000061 16		Noninvasive carcinoma ex pleomorphic adenoma
750662100000061 12		Intracapsular carcinoma ex pleomorphic adenoma
750710100000061 12		Malignant melanoma of ethmoid sinus
750711100000061 10		Malignant melanoma of ethmoidal sinus
750713100000061 16		Malignant melanoma of palate
750714100000061 14		Malignant melanoma of gum
750715100000061 11		Malignant melanoma of gingiva
750716100000061 13		Malignant melanoma of maxillary sinus
750733100000061 19		History of malignant neoplasm of adrenal gland
750749100000061 13		Consultation for malignant neoplasm disease
750750100000061 17		Consultation for cancer
751577100000061 14		History of malignant haematologic neoplasm
751578100000061 12		History of malignant hematologic neoplasm
751872100000061 19		Intraosseous mucoepidermoid carcinoma
752046100000061 12		Paratesticular malignant neoplasm
752047100000061 17		Paratesticular malignant tumor

7520481000006119		Paratesticular malignant tumour
7520911000006117		Hereditary cancer-predisposing syndrome
7521051000006110		Primary malignant stromal sarcoma of endometrium
7525271000006114		Glandular malignant peripheral nerve sheath tumour
7525281000006112		Glandular malignant peripheral nerve sheath tumor
7525291000006110		Glandular malignant peripheral nerve sheath tumour
7525301000006111		Glandular malignant peripheral nerve sheath tumor
7532511000006112		History of malignant neoplasm of renal pelvis
7532521000006116		History of malignant tumor of renal pelvis
7532531000006118		History of malignant tumour of renal pelvis
7534911000006110		Adenocarcinoma of pancreas
7534921000006119		Pancreatic adenocarcinoma
7535781000006118		Malignant sex cord tumour of testis
7535791000006115		Malignant sex cord tumor of testis
75563100000006110	B592X00	Kaposi's sarcoma of multiple organs
75567100000006113	B59zX00	Kaposi's sarcoma, unspecified
7559091000006116		Malignant mixed Mullerian tumor of uterus
7559101000006110		Malignant mixed Mullerian tumour of uterus
7559601000006119		Renal cell carcinoma

7559611 0000061 16		Metastatic renal cell carcinoma
7559851 0000061 19		Malignant granulosa cell tumour of testis
7559861 0000061 17		Malignant granulosa cell tumor of testis
7561421 0000061 17		Malignant neoplasm of augmented bladder
7561431 0000061 19		Malignant tumor of augmented bladder
7561441 0000061 12		Malignant tumour of augmented bladder
7565471 0000061 18		Carcinoma in situ of common hepatic duct
7565481 0000061 15		Carcinoma in situ of perihilar extra hepatic duct
7565801 0000061 10		Sampling of vagina for Papanicolaou smear after hysterectomy for malignant disease
7570221 0000061 18		Poorly differentiated carcinoma
7571051 0000061 11		Anaemia in malignant neoplastic disease
7571061 0000061 13		Cancer-related anaemia
7571071 0000061 18		Anemia in malignant neoplastic disease
7571081 0000061 15		Cancer-related anemia
7572571 0000061 18		Non-small cell lung cancer with mutation in epidermal growth factor receptor
7572591 0000061 17		Non-small cell lung cancer without mutation in epidermal growth factor receptor
7573731 0000061 18		Primary hyperaldosteronism due to aldosterone-secreting malignant neoplasm of adrenal gland
7573741 0000061 11		Aldosterone-producing carcinoma
7573751 0000061 13		Hyperaldosteronism due to carcinoma

7575541000006117		Malignant optic glioma of adulthood
7576101000006116		Excision of secondary malignant neoplasm
7577631000006119		Encapsulated papillary carcinoma
7577641000006112		Encysted papillary carcinoma
7577651000006114		Non-infiltrating intracystic carcinoma
7577661000006111		Noninfiltrating intracystic carcinoma
7577671000006116		Intracystic papillary carcinoma
7577681000006118		Intracystic papillary adenocarcinoma
7577691000006115		Solid papillary carcinoma in situ
7577701000006115		Encapsulated papillary carcinoma with invasion
7577711000006117		Intracystic papillary carcinoma with invasion
7577721000006113		Intracystic papillary adenocarcinoma with invasion
7577731000006111		Encysted papillary carcinoma with invasion
7577761000006119		Small cell carcinoma, hypercalcaemic type
7577771000006114		Small cell carcinoma, hypercalcemic type
7577791000006110		Villoglandular carcinoma
7577911000006119		Serous intraepithelial carcinoma
7577941000006115		Serous carcinoma, non-invasive, low grade
7577961000006116		Low grade serous carcinoma

7577981000006114		High grade serous carcinoma
7578061000006118		Seromucinous carcinoma
7578071000006113		Mucinous adenocarcinoma, endocervical, gastric type
7578081000006111		Adenocarcinoma of mammary gland type
7578111000006117		Cystic hypersecretory carcinoma, intraductal
7578141000006118		Intraductal papilloma with ductal carcinoma in situ
7578161000006119		Papillary carcinoma of the breast
7578171000006114		Invasive micropapillary carcinoma of breast
7578461000006110		Solid papillary carcinoma with invasion
7578471000006115		Medullary-like carcinoma
7578481000006117		Tubulolobular carcinoma
7578671000006117		Perivascular epithelioid tumour, malignant
7578681000006119		Perivascular epithelioid tumor, malignant
7578691000006116		PEComa, malignant
7578771000006110		Myxoid malignant fibrous histiocytoma
7579071000006119		Ossifying fibromyxoid tumour, malignant
7579081000006116		Ossifying fibromyxoid tumor, malignant
7579321000006117		Adenomyoepithelioma with carcinoma
7579331000006119		Malignant adenomyoepithelioma

7579481 0000061 10		Phosphaturic mesenchymal tumour, malignant
7579491 0000061 13		Phosphaturic mesenchymal tumor, malignant
7579561 0000061 16		Adenocarcinoma of rete ovarii
7610911 0000061 17		Triple-negative breast cancer
7610921 0000061 13		Triple negative malignant neoplasm of breast
7610931 0000061 11		TNBC - Triple-negative breast cancer
7615531 0000061 14		Primary adenocarcinoma of accessory sinus
7615541 0000061 16		Adenocarcinoma of accessory sinus
7615561 0000061 17		Primary adenocarcinoma of maxillary sinus
7615571 0000061 12		Adenocarcinoma of maxillary sinus
7615631 0000061 13		Primary adenocarcinoma of ethmoidal sinus
7615641 0000061 15		Adenocarcinoma of ethmoidal sinus
7615651 0000061 18		Primary adenocarcinoma of frontal sinus
7615661 0000061 16		Adenocarcinoma of frontal sinus
7615671 0000061 11		Primary adenocarcinoma of sphenoidal sinus
7615681 0000061 14		Adenocarcinoma of sphenoidal sinus
7615691 0000061 12		Primary carcinoma of accessory sinus
7615701 0000061 12		Primary carcinoma of ethmoidal sinus
7615711 0000061 10		Carcinoma of ethmoidal sinus

7615721 0000061 19		Primary carcinoma of maxillary sinus
7615731 0000061 16		Primary carcinoma of sphenoidal sinus
7615741 0000061 14		Carcinoma of sphenoidal sinus
7615751 0000061 11		Primary carcinoma of frontal sinus
7615761 0000061 13		Carcinoma of frontal sinus
7615771 0000061 18		Malignant melanoma of accessory sinus
7615851 0000061 17		Primary squamous cell carcinoma of accessory sinus
7615861 0000061 15		Squamous cell carcinoma of accessory sinus
7615871 0000061 10		Primary squamous cell carcinoma of maxillary sinus
7615881 0000061 13		Squamous cell carcinoma of maxillary sinus
7615891 0000061 11		Primary squamous cell carcinoma of sphenoidal sinus
7615901 0000061 10		Squamous cell carcinoma of sphenoidal sinus
7615911 0000061 13		Primary squamous cell carcinoma of frontal sinus
7615921 0000061 17		Squamous cell carcinoma of frontal sinus
7615931 0000061 19		Primary squamous cell carcinoma of laryngeal cartilage
7615941 0000061 12		Squamous cell carcinoma of laryngeal cartilage
7615951 0000061 14		Primary squamous cell carcinoma of larynx
7615961 0000061 11		Squamous cell carcinoma of larynx
7615971 0000061 16		Primary squamous cell carcinoma of ethmoidal sinus

7615981000006118		Squamous cell carcinoma of ethmoidal sinus
7615991000006115		Primary lymphoepithelial carcinoma of larynx
7616001000006119		Lymphoepithelial carcinoma of larynx
7616011000006116		Malignant melanoma of frontal sinus
7616031000006110		Malignant melanoma of sphenoidal sinus
7616471000006115		Primary signet ring cell carcinoma of trachea
7616481000006117		Signet ring cell carcinoma of trachea
7616491000006119		Primary myoepithelial carcinoma of trachea
7616501000006110		Myoepithelial carcinoma of trachea
7616511000006113		Primary mucoepidermoid carcinoma of trachea
7616521000006117		Mucoepidermoid carcinoma of trachea
7616531000006119		Primary salivary gland type carcinoma of trachea
7616581000006118		Primary mucinous cystadenocarcinoma of trachea
7616591000006115		Mucinous cystadenocarcinoma of trachea
7616601000006111		Primary solid carcinoma of trachea
7616611000006114		Solid carcinoma of trachea
7616621000006118		Primary undifferentiated carcinoma of trachea
7616631000006115		Undifferentiated carcinoma of trachea
7616641000006113		Primary acinar cell carcinoma of trachea

7616651000006110		Acinar cell carcinoma of trachea
7616681000006119		Primary squamous cell carcinoma of trachea
7616691000006116		Primary clear cell squamous cell carcinoma of trachea
7616701000006116		Clear cell squamous cell carcinoma of trachea
7616711000006118		Primary basaloid squamous cell carcinoma of trachea
7616721000006114		Basaloid squamous cell carcinoma of trachea
7616731000006112		Primary papillary squamous cell carcinoma of trachea
7616741000006119		Papillary squamous cell carcinoma of trachea
7616751000006117		Primary giant cell carcinoma of trachea
7616761000006115		Giant cell carcinoma of trachea
7616771000006110		Primary adenosquamous carcinoma of trachea
7616781000006113		Adenosquamous carcinoma of trachea
7616791000006111		Primary spindle cell carcinoma of trachea
7616801000006112		Spindle cell carcinoma of trachea
7616811000006110		Primary adenocarcinoma of hypopharynx
7616821000006119		Primary oxyphilic adenocarcinoma of oropharynx
7616831000006116		Oncocytic carcinoma of oropharynx
7616841000006114		Primary basal cell adenocarcinoma of oropharynx
7616851000006111		Basal cell adenocarcinoma of oropharynx

7616861 0000061 13		Primary polymorphous low grade adenocarcinoma of oropharynx
7616871 0000061 18		Polymorphous low grade adenocarcinoma of oropharynx
7616881 0000061 15		Primary papillary adenocarcinoma of oropharynx
7616891 0000061 17		Papillary adenocarcinoma of oropharynx
7616901 0000061 18		Primary mucinous adenocarcinoma of oropharynx
7616911 0000061 15		Mucinous adenocarcinoma of oropharynx
7616921 0000061 11		Primary clear cell adenocarcinoma of oropharynx
7616931 0000061 14		Clear cell adenocarcinoma of oropharynx
7616941 0000061 16		Primary adenocarcinoma of oropharynx
7616951 0000061 19		Adenocarcinoma of oropharynx
7616961 0000061 17		Primary fetal adenocarcinoma of lung
7616971 0000061 12		Primary foetal adenocarcinoma of lung
7616981 0000061 10		Fetal adenocarcinoma of lung
7616991 0000061 13		Foetal adenocarcinoma of lung
7617001 0000061 17		Primary mixed subtype adenocarcinoma of lung
7617011 0000061 19		Mixed subtype adenocarcinoma of lung
7617021 0000061 10		Primary adenosquamous carcinoma of lung
7617031 0000061 13		Adenosquamous carcinoma of lung
7617041 0000061 15		Primary mucoepidermoid carcinoma of hypopharynx

7617051 0000061 18		Mucoepidermoid carcinoma of hypopharynx
7617061 0000061 16		Primary signet ring cell carcinoma of lung
7617071 0000061 11		Signet ring cell carcinoma of lung
7617081 0000061 14		Primary small cell non-keratinising squamous cell carcinoma of lung
7617091 0000061 12		Primary small cell non-keratinizing squamous cell carcinoma of lung
7617101 0000061 18		Small cell non-keratinizing squamous cell carcinoma of lung
7617111 0000061 15		Small cell non-keratinising squamous cell carcinoma of lung
7617121 0000061 11		Primary acinar cell carcinoma of lung
7617131 0000061 14		Acinar cell carcinoma of lung
7617141 0000061 16		Primary solid carcinoma of lung
7617151 0000061 19		Solid carcinoma of lung
7617161 0000061 17		Primary papillary adenocarcinoma of lung
7617171 0000061 12		Papillary adenocarcinoma of lung
7617301 0000061 15		Primary undifferentiated carcinoma of larynx
7617311 0000061 17		Primary spindle cell squamous cell carcinoma of larynx
7617321 0000061 13		Spindle cell squamous cell carcinoma of larynx
7617331 0000061 11		Primary basaloid carcinoma of larynx
7617341 0000061 18		Basaloid carcinoma of larynx
7617351 0000061 16		Primary adenosquamous cell carcinoma of larynx

7617361 0000061 19		Adenosquamous cell carcinoma of larynx
7617371 0000061 14		Primary adenoid squamous cell carcinoma of larynx
7617381 0000061 12		Adenoid squamous cell carcinoma of larynx
7617391 0000061 10		Primary papillary squamous cell carcinoma of larynx
7617401 0000061 12		Papillary squamous cell carcinoma of larynx
7617411 0000061 10		Primary verrucous carcinoma of larynx
7617421 0000061 19		Verrucous carcinoma of larynx
7617431 0000061 16		Overlapping squamous cell carcinoma of larynx
7617441 0000061 14		Overlapping squamous cell carcinoma of laryngeal cartilage
7617681 0000061 17		Primary adenocarcinoma of lung
7617691 0000061 19		Primary mucinous adenocarcinoma of lung
7617701 0000061 19		Mucinous adenocarcinoma of lung
7617711 0000061 16		Primary clear cell squamous cell carcinoma of lung
7617721 0000061 12		Clear cell squamous cell carcinoma of lung
7617731 0000061 10		Primary basaloid squamous cell carcinoma of lung
7617741 0000061 17		Basaloid squamous cell carcinoma of lung
7617751 0000061 15		Primary papillary squamous cell carcinoma of lung
7617761 0000061 18		Papillary squamous cell carcinoma of lung
7617771 0000061 13		Primary undifferentiated carcinoma of lung

7617781 0000061 11		Undifferentiated carcinoma of lung
7617791 0000061 14		Primary spindle cell carcinoma of lung
7617801 0000061 10		Spindle cell carcinoma of lung
7617811 0000061 13		Primary pleomorphic carcinoma of lung
7617821 0000061 17		Pleomorphic carcinoma of lung
7617831 0000061 19		Primary pseudosarcomatous carcinoma of lung
7617841 0000061 12		Sarcomatoid carcinoma of lung
7617881 0000061 18		Primary myoepithelial carcinoma of lung
7617891 0000061 15		Myoepithelial carcinoma of lung
7617901 0000061 16		Primary mucoepidermoid carcinoma of lung
7617911 0000061 18		Mucoepidermoid carcinoma of lung
7617921 0000061 14		Primary adenoid cystic carcinoma of lung
7617931 0000061 12		Adenoid cystic carcinoma of lung
7617941 0000061 19		Primary salivary gland type carcinoma of lung
7617971 0000061 10		Primary mixed mucinous and non-mucinous bronchiolo-alveolar carcinoma of lung
7617981 0000061 13		Mixed mucinous and non-mucinous bronchiolo-alveolar carcinoma of lung
7617991 0000061 11		Primary non-mucinous bronchiolo-alveolar carcinoma of lung
7618001 0000061 18		Non-mucinous bronchiolo-alveolar carcinoma of lung
7618011 0000061 15		Primary mucinous bronchiolo-alveolar carcinoma of lung

7618021000006111		Mucinous bronchiolo-alveolar carcinoma of lung
7618031000006114		Primary clear cell adenocarcinoma of trachea
7618041000006116		Clear cell adenocarcinoma of trachea
7618051000006119		Primary papillary adenocarcinoma of trachea
7618061000006117		Papillary adenocarcinoma of trachea
7618071000006112		Primary mucinous adenocarcinoma of trachea
7618081000006110		Mucinous adenocarcinoma of trachea
7618101000006119		Primary adenocarcinoma of trachea
7618111000006116		Adenocarcinoma of trachea
7618181000006111		Primary adenocarcinoma of subglottis
7618211000006110		Primary basaloid squamous cell carcinoma of hypopharynx
7618221000006119		Basaloid squamous cell carcinoma of hypopharynx
7618231000006116		Primary papillary squamous cell carcinoma of hypopharynx
7618241000006114		Papillary squamous cell carcinoma of hypopharynx
7618251000006111		Primary undifferentiated carcinoma of hypopharynx
7618261000006113		Undifferentiated carcinoma of hypopharynx
7618271000006118		Primary adenoid squamous cell carcinoma of hypopharynx
7618281000006115		Adenoid squamous cell carcinoma of hypopharynx
7618291000006117		Primary adenosquamous carcinoma of hypopharynx

7618301 0000061 16		Adenosquamous carcinoma of hypopharynx
7618311 0000061 18		Primary basaloid carcinoma of hypopharynx
7618321 0000061 14		Basaloid carcinoma of hypopharynx
7618331 0000061 12		Primary giant cell carcinoma of hypopharynx
7618341 0000061 19		Giant cell carcinoma of hypopharynx
7618351 0000061 17		Primary spindle cell squamous cell carcinoma of hypopharynx
7618361 0000061 15		Spindle cell squamous cell carcinoma of hypopharynx
7618371 0000061 10		Primary verrucous carcinoma of hypopharynx
7618381 0000061 13		Verrucous carcinoma of hypopharynx
7618391 0000061 11		Primary lymphoepithelial carcinoma of hypopharynx
7618401 0000061 13		Lymphoepithelial carcinoma of hypopharynx
7618411 0000061 11		Primary squamous cell carcinoma of hypopharynx
7618421 0000061 15		Primary lymphoepithelial carcinoma of trachea
7618431 0000061 17		Lymphoepithelial carcinoma of trachea
7618441 0000061 10		Primary verrucous carcinoma of trachea
7618451 0000061 12		Verrucous carcinoma of trachea
7618461 0000061 14		Primary squamous cell adenoid carcinoma of trachea
7618471 0000061 19		Squamous cell adenoid carcinoma of trachea
7619161 0000061 12		Primary squamous cell carcinoma of nasopharynx

7619171 0000061 17		Overlapping squamous cell carcinoma of oropharynx
7619181 0000061 19		Squamous cell carcinoma of overlapping lesion of oropharynx
7619211 0000061 15		Primary squamous cell carcinoma of branchial cleft
7619221 0000061 11		Squamous cell carcinoma of branchial cleft
7619231 0000061 14		Primary squamous cell carcinoma of posterior wall of oropharynx
7619241 0000061 16		Squamous cell carcinoma of posterior wall of oropharynx
7619261 0000061 17		Primary squamous cell carcinoma of lateral wall of oropharynx
7619271 0000061 12		Squamous cell carcinoma of lateral wall of oropharynx
7619301 0000061 14		Primary squamous cell carcinoma of anterior surface of epiglottis
7619311 0000061 12		Primary squamous cell carcinoma of vallecula
7619321 0000061 16		Squamous cell carcinoma of vallecula
7619331 0000061 18		Primary adenoid cystic carcinoma of hypopharynx
7619341 0000061 11		Adenoid cystic carcinoma of hypopharynx
7620131 0000061 19		Primary squamous cell carcinoma of supraglottis
7620141 0000061 12		Primary squamous cell carcinoma of subglottis
7620211 0000061 19		Primary basaloid squamous cell carcinoma of oropharynx
7620221 0000061 10		Basaloid squamous cell carcinoma of oropharynx
7620231 0000061 13		Primary basaloid carcinoma of oropharynx
7620241 0000061 15		Basaloid carcinoma of oropharynx

7620251000006118		Primary papillary squamous cell carcinoma of oropharynx
7620261000006116		Papillary squamous cell carcinoma of oropharynx
7620271000006111		Primary spindle cell squamous cell carcinoma of oropharynx
7620281000006114		Spindle cell squamous cell carcinoma of oropharynx
7620291000006112		Primary adenosquamous carcinoma of oropharynx
7620301000006113		Adenosquamous carcinoma of oropharynx
7620311000006111		Primary lymphoepithelial carcinoma of oropharynx
7620321000006115		Lymphoepithelial carcinoma of oropharynx
7620331000006117		Primary squamous cell carcinoma of oropharynx
7620341000006110		Squamous cell carcinoma of oropharynx
7620351000006112		Primary myoepithelial carcinoma of oropharynx
7620361000006114		Myoepithelial carcinoma of oropharynx
7620371000006119		Primary carcinoma ex pleomorphic adenoma of oropharynx
7620381000006116		Carcinoma ex pleomorphic adenoma of oropharynx
7620391000006118		Primary epithelial-myoepithelial carcinoma of oropharynx
7620401000006116		Epithelial-myoepithelial carcinoma of oropharynx
7620411000006118		Primary cystadenocarcinoma of oropharynx
7620421000006114		Cystadenocarcinoma of oropharynx
7620431000006112		Primary acinar cell carcinoma of oropharynx

7620441 0000061 19		Acinar cell carcinoma of oropharynx
7620451 0000061 17		Primary mucoepidermoid carcinoma of oropharynx
7620461 0000061 15		Mucoepidermoid carcinoma of oropharynx
7620471 0000061 10		Primary infiltrating duct carcinoma of oropharynx
7620481 0000061 13		Infiltrating duct carcinoma of oropharynx
7620561 0000061 19		Primary mucinous cystadenocarcinoma of lung
7620571 0000061 14		Mucinous cystadenocarcinoma of lung
7621001 0000061 16		Primary salivary gland type carcinoma of hypopharynx
7621051 0000061 17		Overlapping squamous cell carcinoma of hypopharynx
7621061 0000061 15		Squamous cell carcinomas of overlapping lesion of hypopharynx
7621571 0000061 18		Primary giant cell carcinoma of larynx
7621581 0000061 15		Giant cell carcinoma of larynx
7621601 0000061 13		Primary basaloid squamous cell carcinoma of larynx
7621611 0000061 11		Basaloid squamous cell carcinoma of larynx
7621631 0000061 17		Primary squamous cell carcinoma of glottis
7621781 0000061 10		Primary malignant epithelial neoplasm of trachea
7621791 0000061 13		Primary carcinoma of trachea
7622011 0000061 10		Primary squamous cell carcinoma of posterior wall of hypopharynx
7622021 0000061 19		Squamous cell carcinomas of posterior wall of hypopharynx

7622231 0000061 14		Primary squamous cell carcinoma of hypopharyngeal aspect of aryepiglottic fold
7622241 0000061 16		Squamous cell carcinomas of aryepiglottic fold, hypopharyngeal aspect
7622311 0000061 12		Primary squamous cell carcinoma of postcricoid region
7622321 0000061 16		Squamous cell carcinomas of postcricoid region
7622331 0000061 18		Primary squamous cell carcinoma of pyriform sinus
7622341 0000061 11		Squamous cell carcinoma of piriform sinus
7622351 0000061 13		Nonkeratinizing carcinoma of the nasopharynx
7627431 0000061 11		Malignant neoplasm after immunosuppressive therapy
7632151 0000061 12		International Federation of Gynaecology and Obstetrics cervical cancer (FIGO CC) stage IA1
7632161 0000061 14		International Federation of Gynecology and Obstetrics cervical cancer (FIGO CC) stage IA1
7632231 0000061 11		International Federation of Gynecology and Obstetrics cervical cancer (FIGO CC) stage IA2
7632241 0000061 18		International Federation of Gynaecology and Obstetrics cervical cancer (FIGO CC) stage IA2
7632411 0000061 16		International Federation of Gynecology and Obstetrics cervical cancer (FIGO CC) stage IB1
7632421 0000061 12		International Federation of Gynaecology and Obstetrics cervical cancer (FIGO CC) stage IB1
7632711 0000061 10		International Federation of Gynecology and Obstetrics cervical cancer (FIGO CC) stage IB2
7632721 0000061 19		International Federation of Gynaecology and Obstetrics cervical cancer (FIGO CC) stage IB2
7632891 0000061 11		International Federation of Gynaecology and Obstetrics cervical cancer (FIGO CC) stage IIA1
7632901 0000061 10		International Federation of Gynecology and Obstetrics cervical cancer (FIGO CC) stage IIA1
7632941 0000061 12		International Federation of Gynecology and Obstetrics cervical cancer (FIGO CC) stage IIA2

7632951 0000061 14		International Federation of Gynaecology and Obstetrics cervical cancer (FIGO CC) stage IIA2
7633021 0000061 12		International Federation of Gynaecology and Obstetrics cervical cancer (FIGO CC) stage IIB
7633031 0000061 10		International Federation of Gynecology and Obstetrics cervical cancer (FIGO CC) stage IIB
7633121 0000061 13		International Federation of Gynecology and Obstetrics cervical cancer (FIGO CC) stage IIIA
7633131 0000061 11		International Federation of Gynaecology and Obstetrics cervical cancer (FIGO CC) stage IIIA
7633181 0000061 12		International Federation of Gynaecology and Obstetrics cervical cancer (FIGO CC) stage IIIB
7633191 0000061 10		International Federation of Gynecology and Obstetrics cervical cancer (FIGO CC) stage IIIB
7633281 0000061 16		International Federation of Gynaecology and Obstetrics cervical cancer (FIGO CC) stage IVA
7633291 0000061 18		International Federation of Gynecology and Obstetrics cervical cancer (FIGO CC) stage IVA
7633371 0000061 11		International Federation of Gynecology and Obstetrics cervical cancer (FIGO CC) stage IVB
7633381 0000061 14		International Federation of Gynaecology and Obstetrics cervical cancer (FIGO CC) stage IVB
7633571 0000061 16		International Federation of Gynaecology and Obstetrics endometrial cancer (FIGO EC) stage IIIB
7633581 0000061 18		International Federation of Gynecology and Obstetrics endometrial cancer (FIGO EC) stage IIIB
7633591 0000061 15		International Federation of Gynecology and Obstetrics endometrial cancer stage IIIB
7633601 0000061 11		International Federation of Gynaecology and Obstetrics endometrial cancer stage IIIB
7633911 0000061 15		International Federation of Gynaecology and Obstetrics endometrial cancer (FIGO EC) stage IIIC1
7633921 0000061 11		International Federation of Gynaecology and Obstetrics endometrial cancer (FIGO EC) stage IIIC1
7633931 0000061 14		International Federation of Gynecology and Obstetrics endometrial cancer stage IIIC1
7633941 0000061 16		International Federation of Gynaecology and Obstetrics endometrial cancer stage IIIC1

7633991 0000061 13		International Federation of Gynecology and Obstetrics endometrial cancer (FIGO EC) stage IIIC2
7634001 0000061 16		International Federation of Gynaecology and Obstetrics endometrial cancer (FIGO EC) stage IIIC2
7634011 0000061 18		International Federation of Gynaecology and Obstetrics endometrial cancer stage IIIC2
7634021 0000061 14		International Federation of Gynecology and Obstetrics endometrial cancer stage IIIC2
7634151 0000061 18		International Federation of Gynaecology and Obstetrics endometrial cancer (FIGO EC) stage IVA
7634161 0000061 16		International Federation of Gynecology and Obstetrics endometrial cancer (FIGO EC) stage IVA
7634210 0000611 6	B62 7C1 1	Follicular lymphoma NOS
7634211 0000061 13		International Federation of Gynecology and Obstetrics endometrial cancer (FIGO EC) stage IVB
7634221 0000061 17		International Federation of Gynaecology and Obstetrics endometrial cancer (FIGO EC) stage IVB
7634310 0000611 8	B62 710 0	Follicular non-Hodgkin's mixed small cleaved and large cell lymphoma
7634641 0000061 10		International Federation of Gynaecology and Obstetrics vulvar carcinoma (FIGO VC) stage IA
7634651 0000061 12		International Federation of Gynecology and Obstetrics vulvar carcinoma (FIGO VC) stage IA
7634711 0000061 15		International Federation of Gynaecology and Obstetrics vulvar carcinoma (FIGO VC) stage IB
7634721 0000061 11		International Federation of Gynecology and Obstetrics vulvar carcinoma (FIGO VC) stage IB
7634771 0000061 12		International Federation of Gynaecology and Obstetrics vulvar carcinoma (FIGO VC) stage IIIA
7634781 0000061 10		International Federation of Gynaecology and Obstetrics vulvar carcinoma (FIGO VC) stage IIIA
7634841 0000061 11		International Federation of Gynaecology and Obstetrics vulvar carcinoma (FIGO VC) stage IIIB
7634851 0000061 13		International Federation of Gynecology and Obstetrics vulvar carcinoma (FIGO VC) stage IIIB
7634901 0000061 15		International Federation of Gynaecology and Obstetrics vulvar carcinoma (FIGO VC) stage IVA

7634911 0000061 17		International Federation of Gynecology and Obstetrics vulvar carcinoma (FIGO VC) stage IVA
7634991 0000061 10		International Federation of Gynaecology and Obstetrics vulvar carcinoma (FIGO VC) stage IVB
7635001 0000061 15		International Federation of Gynecology and Obstetrics vulvar carcinoma (FIGO VC) stage IVB
7635691 0000061 17		International Federation of Gynaecology and Obstetrics endometrial cancer (FIGO EC) stage IIIA
7635711 0000061 19		International Federation of Gynecology and Obstetrics endometrial cancer (FIGO EC) stage IIIA
7641581 0000061 18		Carcinoma of central portion of breast
7641881 0000061 15		Papillary adenocarcinoma, metastatic
7642331 0000061 16		Diffuse sclerosing papillary thyroid carcinoma
7642361 0000061 13		Metastatic hepatocellular carcinoma
7643151 0000061 17		Malignant neoplasm of superior wall of nasopharynx
7644591 0000061 18		Neck pain due to malignant neoplastic disease
7644601 0000061 14		Neck pain due to malignant neoplasm
7645511 0000061 10		Metastatic papillary thyroid carcinoma
7645541 0000061 14		Metastatic small cell carcinoma
7645561 0000061 13		Metastatic thymic carcinoma
7647041 0000061 10		Secondary malignant neoplasm of lumbosacral plexus
7647051 0000061 12		Metastatic malignant neoplasm to lumbosacral plexus
7647061 0000061 14		Cancer metastatic to lumbosacral plexus
7650911 0000061 11		Malignant carcinoid tumour of small intestine

765092100000061 15		Malignant carcinoid tumor of small intestine
765265100000061 17		Mucin-like carcinoma-associated antigen
765266100000061 15		Mucin-like carcinoma-associated Ag
765857100000061 13		Malignant carcinoid tumour of stomach
765858100000061 11		Malignant carcinoid tumor of stomach
766547100000061 14		Malignant odontogenic neoplasm of lower jaw
766548100000061 12		Malignant odontogenic tumour of upper jaw
766549100000061 10		Malignant odontogenic tumor of upper jaw
766550100000061 19		Malignant odontogenic neoplasm of upper jaw
768805100000061 17		Primary clear cell adenocarcinoma of lung
769490100000061 13		Malignant neoplasm of chest wall
769622100000061 19		Prostate cancer metastatic to bone
769623100000061 16		Carcinoma of prostate with bony metastases
769624100000061 14		Primary malignant neoplasm of prostate metastatic to bone
769892100000061 14		Overlapping primary malignant neoplasm of bone and articular cartilage of upper limb
769893100000061 12		Overlapping primary malignant neoplasm of bone and articular cartilage of upper extremity
770074100000061 10		Malignant insulinoma
770203100000061 15		Malignant ameloblastoma of mandible
770206100000061 12		Malignant germ cell neoplasm of mediastinum

7702501 0000061 15		Malignant meningioma of meninges of brain
7706081 0000061 15		Malignant carcinoid tumour of rectum
7706091 0000061 17		Malignant carcinoid tumor of rectum
7706101 0000061 11		Malignant carcinoid tumour of kidney
7706111 0000061 14		Malignant carcinoid tumor of kidney
7706221 0000061 11		Malignant germ cell neoplasm
7706551 0000061 17		Invasive carcinoma of breast
7706611 0000061 17		Hereditary nonpolyposis colon cancer gene mutation positive
7707271 0000061 14		Malignant germ cell tumour of testis
7707281 0000061 12		Malignant germ cell tumor of testis
7734141 0000061 13		Hereditary papillary renal cell carcinoma
7745031 0000061 16		Nodular basal cell carcinoma of skin
7749001 0000061 10		Carcinoma of stomach due to Epstein-Barr virus disease
7749011 0000061 13		Gastric carcinoma due to Epstein-Barr virus disease
7749041 0000061 12		Primary non-gestational choriocarcinoma of ovary
7749051 0000061 14		Primary non-gestational ovarian choriocarcinoma
7749131 0000061 18		Carcinoma of salivary gland type of breast
7749141 0000061 11		Salivary gland type cancer of breast
7750071 0000061 10		Extraovarian primary peritoneal carcinoma

7750081000006113		EOPPC - Extraovarian primary peritoneal carcinoma
7750091000006111		Primary peritoneal serous carcinoma
7750101000006117		Serous surface papillary carcinoma
7750161000006116		Primary hepatic neuroendocrine carcinoma
7750171000006111		Neuroendocrine carcinoma of thymus
7750181000006114		Thymic neuroendocrine carcinoma
7750251000006114		Squamous cell carcinoma of head and neck
7750291000006115		Childhood neoplasm of heart
7751501000006115		Cancer-related fatigue
7751511000006117		Fatigue associated with malignant neoplastic disease
7753031000006119		Theca steroid producing cell malignant neoplasm of ovary
7753041000006112		Theca steroid producing cell malignant tumor of ovary
7753051000006114		Theca steroid producing cell malignant tumour of ovary
7753081000006118		Hereditary diffuse carcinoma of stomach
7753091000006115		Hereditary diffuse gastric cancer
7761571000006118		Basal cell carcinoma of vulva
7761601000006113		Papillary thyroid carcinoma with renal papillary neoplasia
7761611000006111		Renal cell carcinoma of kidney except renal pelvis
7764261000006114		Neuroendocrine carcinoma of appendix

7764341000006115		Poorly-differentiated neuroendocrine carcinoma of thymus
7764351000006118		Poorly-differentiated thymic neuroendocrine carcinoma
7764361000006116		Well-differentiated neuroendocrine carcinoma of thymus
7764371000006111		Well-differentiated thymic neuroendocrine carcinoma
7769431000006119		Hereditary breast and ovarian cancer syndrome
7771751000006110		Basal cell carcinoma in situ
7774561000006118		Small cell neuroendocrine carcinoma of bladder
7780991000006115		Recurrent squamous cell carcinoma
7781001000006119		Recurrent SCC (squamous cell carcinoma)
7781611000006114		Small cell neuroendocrine carcinoma
7792041000006111		Monitoring following treatment for cancer
7792051000006113		Post-cancer treatment monitoring
7815251000006110		Myeloid and lymphoid neoplasm with fibroblast growth factor receptor 1 abnormality
7815261000006112		Myeloid and lymphoid neoplasm with FGFR1 abnormality
7819281000006116		Primary adnexal carcinoma of skin
7819291000006118		Primary malignant sarcoma of skin
7819401000006114		Primary adenocarcinoma of ciliary epithelium
7819411000006112		Primary adenocarcinoma of epithelium of iris
7819421000006116		Primary adenocarcinoma of iris epithelium

7819431000006118		Primary malignant neoplasm of lacrimal apparatus
7819441000006111		Primary adenocarcinoma of lacrimal apparatus
7819521000006117		Primary adenocarcinoma of palate
7819531000006119		Primary adenocarcinoma of parotid gland
7819541000006112		Primary adenocarcinoma of cystic duct
7819551000006114		Primary adenocarcinoma of common bile duct
7819561000006111		Primary adenocarcinoma of nasal cavity
7819571000006116		Primary adenocarcinoma of middle ear
7819581000006118		Primary adenocarcinoma overlapping lesion of retroperitoneum peritoneum and omentum
7819591000006115		Primary malignant melanoma of vagina
7819601000006111		Primary malignant neoplasm of placenta
7819611000006114		Primary adenocarcinoma of parametrium
7819621000006118		Primary adenocarcinoma of uterine ligament
7820001000006115		Papillary carcinoma in situ of breast
7820011000006117		Primary intracystic papillary carcinoma of breast
7820021000006113		Solid papillary carcinoma in situ of breast
7820091000006110		Primary embryonal carcinoma of testis
7820101000006116		Primary choriocarcinoma of testis
7820111000006118		Primary squamous cell carcinoma of overlapping lesion of male genital organ

7820121000006114		Primary squamous cell carcinoma of overlapping lesion of accessory sinuses
7820131000006112		Primary adenocarcinoma of overlapping lesion of accessory sinuses
7820141000006119		Primary undifferentiated carcinoma of oropharynx
7820331000006114		Primary adenocarcinoma of lower third of oesophagus due to Barrett oesophagus
7820341000006116		Primary adenocarcinoma of lower third of esophagus due to Barrett esophagus
7820351000006119		Barrett adenocarcinoma
7820361000006117		Primary squamous cell carcinoma of upper third of oesophagus
7820371000006112		Primary squamous cell carcinoma of upper third of esophagus
7820381000006110		Primary squamous cell carcinoma of middle third of oesophagus
7820391000006113		Primary squamous cell carcinoma of middle third of esophagus
7820401000006110		Primary squamous cell carcinoma of lower third of oesophagus
7820411000006113		Primary squamous cell carcinoma of lower third of esophagus
7820421000006117		Primary squamous cell carcinoma of overlapping lesion of oesophagus
7820431000006119		Primary squamous cell carcinoma of overlapping lesion of esophagus
7820441000006112		Primary adenocarcinoma of upper third of oesophagus
7820451000006114		Primary adenocarcinoma of upper third of esophagus
7820461000006111		Primary adenocarcinoma of middle third of oesophagus
7820471000006116		Primary adenocarcinoma of middle third of esophagus
7820481000006118		Primary adenocarcinoma of overlapping lesion of oesophagus

7820491 0000061 15		Primary adenocarcinoma of overlapping lesion of esophagus
7820501 0000061 11		Primary neuroendocrine carcinoma of oesophagus
7820511 0000061 14		Primary neuroendocrine carcinoma of esophagus
7820521 0000061 18		Primary malignant neuroendocrine neoplasm of oesophagus
7820531 0000061 15		Primary malignant neuroendocrine neoplasm of esophagus
7820541 0000061 13		Malignant melanoma of oesophagus
7820551 0000061 10		Malignant melanoma of esophagus
7820561 0000061 12		Primary adenocarcinoma of oesophagogastric junction
7820571 0000061 17		Primary adenocarcinoma of esophagogastric junction
7820581 0000061 19		Primary adenocarcinoma of cardioesophageal junction
7820601 0000061 12		Primary adenocarcinoma of cardia of stomach
7820621 0000061 19		Primary adenocarcinoma of pyloric antrum of stomach
7820631 0000061 16		Primary adenocarcinoma of overlapping lesion of stomach
7820641 0000061 14		Primary malignant neuroendocrine neoplasm of stomach
7820651 0000061 11		Primary malignant neuroendocrine neoplasm of cardia of stomach
7820661 0000061 13		Primary malignant neuroendocrine neoplasm of body of stomach
7820671 0000061 18		Primary malignant neuroendocrine neoplasm of pyloric antrum of stomach
7820681 0000061 15		Primary neuroendocrine carcinoma of stomach
7820691 0000061 17		Primary neuroendocrine carcinoma of cardia of stomach

7820701000006117		Primary neuroendocrine carcinoma of body of stomach
7820711000006119		Primary neuroendocrine carcinoma of pyloric antrum of stomach
7820721000006110		Primary neuroendocrine carcinoma of overlapping lesion of stomach
7820731000006113		Primary malignant mesenchymal neoplasm of stomach
7820741000006115		Primary malignant neuroendocrine neoplasm of duodenum
7820751000006118		Primary neuroendocrine carcinoma of duodenum
7821061000006111		Primary adenocarcinoma of overlapping lesion of small intestine
7821071000006116		Primary malignant neuroendocrine neoplasm of small intestine
7821081000006118		Primary neuroendocrine carcinoma of small intestine
7821091000006115		Primary mucinous adenocarcinoma of appendix
7821101000006114		Primary malignant neuroendocrine neoplasm of appendix
7821391000006117		Primary adenocarcinoma of ascending colon and right flexure
7821401000006115		Primary adenocarcinoma of transverse colon
7821411000006117		Primary neuroendocrine carcinoma of colon
7821421000006113		Primary malignant neuroendocrine neoplasm of colon
7821431000006111		Primary adenocarcinoma of descending colon and splenic flexure
7821441000006118		Primary malignant neuroendocrine neoplasm of rectum
7821451000006116		Primary neuroendocrine carcinoma of rectum
7821621000006111		Primary cloacogenic carcinoma of anal canal

782172100000006115		Primary cholangiocarcinoma of intrahepatic biliary tract
782173100000006117		Intrahepatic cholangiocarcinoma
782175100000006112		Primary adenocarcinoma of ampulla of Vater
782183100000006113		Primary adenocarcinoma of peritoneum
782708100000006113		Obstructive nephropathy due to prostate cancer
782709100000006111		Obstructive nephropathy due to carcinoma of prostate
782716100000006116		Membranous glomerulonephritis due to malignant neoplastic disease
782718100000006114		Obstructive nephropathy due to bladder cancer
782730100000006119		Hormone sensitive prostate cancer
782731100000006116		Castrate-sensitive prostate cancer
782732100000006112		Castration-sensitive prostate cancer
782749100000006116		Primary squamous cell carcinoma of ear
782893100000006112		Comedocarcinoma, no International Classification of Diseases for Oncology subtype
782894100000006119		Comedocarcinoma, no ICD-O subtype
783140100000006118		Reactive oxygen species 1 positive non-small cell lung cancer
783141100000006115		ROS1 positive NSCLC - reactive oxygen species 1 positive non-small cell lung cancer
783274100000006117		Primary malignant nerve sheath neoplasm of peripheral nervous system structure
783275100000006115		Primary malignant nerve sheath neoplasm of autonomic nerve
783283100000006119		Mixed ductal and lobular carcinoma in situ of breast

7832841 0000061 12		Primary invasive pleomorphic lobular carcinoma of breast
7832871 0000061 16		Primary malignant neuroendocrine neoplasm of bronchus
7832881 0000061 18		Primary malignant neuroendocrine neoplasm of lung
7832891 0000061 15		Primary malignant epithelial neoplasm of nasopharynx
7832901 0000061 16		Primary squamous cell carcinoma of pharyngeal tonsil
7832911 0000061 18		Primary squamous cell carcinoma of adenoid
7832961 0000061 15		Primary malignant neoplasm of oesophagogastric junction
7832971 0000061 10		Primary malignant neoplasm of esophagogastric junction
7832981 0000061 13		Primary malignant neoplasm of cardioesophageal junction
7833091 0000061 17		Primary squamous cell carcinoma of anal canal
7833101 0000061 11		Primary malignant melanoma of anal canal
7834971 0000061 17		Carcinoma in situ of ocular adnexa
7834981 0000061 19		Primary malignant melanoma of cornea
7834991 0000061 16		Primary squamous cell carcinoma of lacrimal apparatus
7835031 0000061 15		Primary thymic carcinoma
7835041 0000061 13		Metastatic malignant neoplasm of meninges
7835051 0000061 10		Primary squamous cell carcinoma of base of tongue
7835061 0000061 12		Primary squamous cell carcinoma of root of tongue
7835071 0000061 17		Primary squamous cell carcinoma of lingual tonsil

7835081 0000061 19		Primary squamous cell carcinoma of parotid gland
7835141 0000061 12		Primary squamous cell carcinoma of middle ear
7835161 0000061 11		Primary squamous cell carcinoma of vagina
7835171 0000061 16		Primary mucinous adenocarcinoma of endometrium
7835181 0000061 18		Primary endometrial mucinous adenocarcinoma
7835191 0000061 15		Primary serous adenocarcinoma of endometrium
7835201 0000061 17		Primary endometrial serous adenocarcinoma
7835211 0000061 19		Primary mixed adenocarcinoma of endometrium
7835221 0000061 10		Primary endometrial mixed adenocarcinoma
7835231 0000061 13		Primary small cell carcinoma of endometrium
7835241 0000061 15		Primary endometrial small cell carcinoma
7835251 0000061 18		Primary neuroendocrine carcinoma of cervix uteri
7835261 0000061 16		Primary low grade serous adenocarcinoma of ovary
7835271 0000061 11		Primary high grade serous adenocarcinoma of ovary
7835281 0000061 14		Primary serous carcinoma of uterine adnexa
7835291 0000061 12		Primary mucinous carcinoma of uterine adnexa
7835301 0000061 13		Malignant epithelial neoplasm
7835311 0000061 11		Carcinoma
7835501 0000061 15		Metastatic malignant neoplasm of peripheral nervous system

7835521000006113		Occupational cancer of skin
7835531000006111		Occupational skin cancer
7835541000006118		Carcinoma in situ of anal margin
7835551000006116		Primary mucoepidermoid carcinoma of lacrimal apparatus
7835561000006119		Primary malignant meningioma
7836841000006111		Primary solid papillary carcinoma with invasion of breast
7841271000006112		Carcinoma in situ of skin of penis
7841441000006115		Primary squamous cell carcinoma of nasal cavity
7842091000006111		Primary squamous cell carcinoma of anus
7842241000006112		Primary malignant melanoma of anus
7842361000006118		Squamous non-small cell lung cancer
7842371000006113		Squamous NSCLC (non-small cell lung cancer)
7848871000006118		Primary adenocarcinoma of overlapping lesion of urinary organ
7850981000006110		Malignant neoplasm of lower lobe of right lung
7850991000006113		Malignant neoplasm of upper lobe of left lung
7851001000006114		Malignant neoplasm of lower lobe of left lung
7851011000006112		Malignant neoplasm of right upper lobe of lung
7855951000006112		Lymphoedema due to malignant infiltration
7856221000006118		Primary squamous cell carcinoma of overlapping lesion of urinary organs

7856231000006115		Primary urothelial carcinoma of overlapping lesion of urinary organ
7856241000006113		Primary transitional cell carcinoma of overlapping lesion of urinary organ
7857531000006115		Primary adenocarcinoma of ileum
7857541000006113		Primary adenocarcinoma of jejunum
7857741000006115		Primary poorly differentiated carcinoma of thyroid gland
7857751000006118		Primary undifferentiated carcinoma of thyroid gland
7857761000006116		Primary malignant epithelial neoplasm of endocrine gland
7858901000006115		Myeloid neoplasm associated with beta-type platelet-derived growth factor receptor gene rearrangement
7858911000006117		Myeloid neoplasm associated with PDGFRB rearrangement
7861531000006119		Primary malignant neuroepitheliomatous neoplasm of peripheral nerve
7861541000006112		Primary malignant neuroepitheliomatous neoplasm of autonomic nervous system
7862411000006117		Primary malignant neoplasm of skin due to and following radiotherapy caused by ionising radiation
7862421000006113		Primary malignant neoplasm of skin due to and following radiotherapy caused by ionizing radiation
7869411000006111		Irreversible electroporation ablation of neoplasm of pancreas
7869821000006118		Poorly cohesive carcinoma
7869831000006115		Thymic carcinoma with adenoid cystic carcinoma-like features
7877121000006111		Intraductal papillary mucinous neoplasm
7881221000006118		Malignant carcinoid tumour of thymus
7881231000006115		Malignant carcinoid tumor of thymus

7881241000006113		Malignant carcinoid tumour of bronchus
7881251000006110		Malignant carcinoid tumor of bronchus
7881261000006112		Malignant carcinoid tumour of colon
7881271000006117		Malignant carcinoid tumor of colon
7952181000006119		Non-intestinal type adenocarcinoma
7952191000006116		Nonintestinal type adenocarcinoma
7954271000006119		Primary squamous cell carcinoma of paraurethral gland
7954281000006116		Primary urothelial carcinoma of paraurethral gland
7954291000006118		Primary transitional cell carcinoma of paraurethral gland
7954301000006117		Primary adenocarcinoma of urethra
7954411000006112		Malignant epithelial neoplasm of bronchus
7954721000006114		Primary malignant neuroendocrine neoplasm of anus
7954731000006112		Primary malignant neuroendocrine neoplasm of anal canal
7955101000006119		Dementia due to primary malignant neoplasm of brain
7956931000006118		Primary squamous cell carcinoma of oral cavity
7956941000006111		Primary squamous cell carcinoma of lip
7956951000006113		Primary squamous cell carcinoma of pharynx
7956961000006110		Primary mucinous cystic neoplasm with associated invasive carcinoma of perihilar bile duct
7956971000006115		Primary mucinous cystic neoplasm with associated invasive carcinoma of cystic duct

7956981000006117		Primary malignant neuroendocrine neoplasm of cystic duct
7956991000006119		Primary mucinous cystic neoplasm with associated invasive carcinoma of distal bile duct
7957001000006110		Primary malignant neuroendocrine of distal bile duct
7957011000006113		Primary malignant neuroendocrine neoplasm of ampulla of Vater
7957021000006117		Primary mucinous cystic neoplasm with associated invasive carcinoma of biliary tract
7957031000006119		Primary malignant neuroendocrine neoplasm of biliary tract
7957041000006112		Primary adenocarcinoma of biliary tract
7957051000006114		Primary adenocarcinoma of digestive organ
7957061000006111		Primary mucinous carcinoma of digestive organ
7957071000006116		Primary mucinous adenocarcinoma of digestive organ
7957081000006118		Primary squamous cell carcinoma of intrathoracic organ
7957091000006115		Primary squamous cell carcinoma of respiratory system
7957101000006114		Primary squamous cell carcinoma of endometrium
7957111000006112		Endometrial squamous cell carcinoma
7957121000006116		Primary undifferentiated carcinoma of endometrium
7957131000006118		Endometrial undifferentiated carcinoma
7957141000006111		Primary mucinous adenocarcinoma of ovary
7957151000006113		Primary adenocarcinoma of paraurethral gland
7957661000006117		Primary ameloblastic carcinoma

7958341000006113		Collecting duct carcinoma of kidney
7958351000006110		Bellini carcinoma
7958361000006112		Bellini duct carcinoma
7958371000006117		Renal collecting duct carcinoma
7958381000006119		Chromophobe renal cell carcinoma
7959491000006115		Tubulocystic renal cell carcinoma
7959501000006111		Tubulocystic renal cell carcinoma
7959591000006116		Papillary renal cell carcinoma
7959601000006112		Papillary renal cell carcinoma
7960971000006114		Invasive carcinoma of uterine cervix co-occurrent with human immunodeficiency virus infection
7960981000006112		Invasive carcinoma of uterine cervix co-occurrent with HIV (human immunodeficiency virus) infection
7961031000006115		Cribriform comedo-type adenocarcinoma
7961131000006119		Neuroendocrine type combined small cell carcinoma
7961531000006111		Fibromatosis-like metaplastic carcinoma
7961541000006118		Micropapillary adenocarcinoma
7961561000006119		MiT family translocation renal cell carcinoma
7961571000006114		Mucinous adenocarcinoma in situ
7961581000006112		Non-mucinous adenocarcinoma in situ
7961591000006110		Mucinous minimally invasive adenocarcinoma

79616010000006119		Non-mucinous minimally invasive adenocarcinoma
79617810000006117		Pseudovascular squamous cell carcinoma
79619810000006115		Primary intraosseous squamous cell carcinoma derived from odontogenic cyst
79619910000006117		Primary intraosseous squamous cell carcinoma derived from keratocystic odontogenic neoplasm
79621010000006116		Nuclear protein in testis associated carcinoma
79631510000006113		Clear cell papillary renal cell carcinoma
79631610000006110		Clear cell papillary renal cell carcinoma
79634210000006113		Clear cell odontogenic carcinoma
79636110000006115		Mucinous cystic neoplasm with low-grade intraepithelial neoplasia
79636210000006111		Sebaceous lymphadenocarcinoma
79636310000006114		Mucinous cystic neoplasm with high-grade intraepithelial neoplasia
79636710000006112		Mammary analogue secretory carcinoma
79636810000006110		Mammary analog secretory carcinoma
79636910000006113		MASC Ã¢â�¬â�� mammary analogue secretory carcinoma
79637010000006113		Secondary dedifferentiated peripheral ameloblastic carcinoma
79637110000006111		Secondary dedifferentiated intraosseous ameloblastic carcinoma
79637710000006119		Malignant peripheral nerve sheath neoplasm with perineurial differentiation
79637810000006116		MPNST (malignant peripheral nerve sheath tumor) with perineurial differentiation
79637910000006118		MPNST (malignant peripheral nerve sheath tumour) with perineurial differentiation

7963801 0000061 17		Germ cell neoplasm with haematological malignancy
7963811 0000061 19		Germ cell neoplasm with hematological malignancy
7963821 0000061 10		Germ cell neoplasm with somatic-type solid malignancy
7963861 0000061 16		Mucinous cystic neoplasm with invasive carcinoma
7963871 0000061 11		Intraductal papillary neoplasm with invasive carcinoma
7963901 0000061 11		Mucinous tubular and spindle cell carcinoma
7964161 0000061 13		Intraductal papillary neoplasm with low-grade intraepithelial neoplasia
7964201 0000061 19		Primary solid type intraosseous squamous cell carcinoma
7964461 0000061 17		Intraductal papillary neoplasm with high grade intraepithelial neoplasia
7964481 0000061 10		Intracystic papillary neoplasm with low grade intraepithelial neoplasia
7965261 0000061 19		History of malignant melanoma of the skin
7968011 0000061 15		History of primary malignant neoplasm of testis
7968031 0000061 14		History of primary malignant neoplasm of oropharynx
7968051 0000061 19		History of primary malignant neoplasm of larynx
7969671 0000061 14		Primary adenocarcinoma of pancreas
7969681 0000061 12		Pancreatic adenocarcinoma
7969741 0000061 12		Metastasis to lung from adenocarcinoma
7969771 0000061 16		Metastasis to lymph node from squamous cell carcinoma
7969821 0000061 12		Metastasis to lymph node from adenocarcinoma

7969911 0000061 11		Metastasis to liver from adenocarcinoma
7970001 0000061 14		Primary adenocarcinoma of colon
7987461 0000061 10		Primary adenoid cystic carcinoma of nasopharynx
7990721 0000061 14		Primary adenocarcinoma of gallbladder
8005521 0000061 11		Primary squamous cell carcinoma of palatine tonsil
8009411 0000061 19		Malignant phaeochromocytoma
8009421 0000061 10		Malignant pheochromocytoma
8011921 0000061 17		Primary squamous cell carcinoma of upper limb
8014171 0000061 16		Adenocarcinoma in adenomatous polyp
8017201 0000061 14		Primary invasive malignant neoplasm of female breast
8017691 0000061 17		History of invasive malignant neoplasm of breast
8017711 0000061 19		History of malignant meningeal neoplasm
8017721 0000061 10		History of malignant neoplasm of meninges
8017781 0000061 14		History of hepatocellular carcinoma
8019631 0000061 14		Basal cell carcinoma of naris
8021331 0000061 13		Primary small cell malignant neoplasm of lung, TNM stage 1
8021341 0000061 15		Primary small cell malignant neoplasm of lung, TNM stage 2
8021351 0000061 18		Primary small cell malignant neoplasm of lung, TNM stage 3
8021361 0000061 16		Primary small cell malignant neoplasm of lung, TNM stage 4

8021851000006111		History of basal cell carcinoma of eyelid
8021891000006117		History of squamous cell carcinoma
8022211000006118		Recurrent primary malignant neoplasm of vulva
8023001000006115		History of cholangiocarcinoma
8023011000006117		History of malignant neoplasm of hypopharynx
8023021000006113		History of malignant neoplasm of nasopharynx
8023031000006111		History of malignant neoplasm of salivary gland
8023041000006118		History of cancer of gall bladder
8023051000006116		History of malignant neoplasm of pancreas
8023061000006119		History of cancer of ampulla of duodenum
8023071000006114		History of malignant neoplasm of appendix
8023081000006112		History of malignant neoplasm of common bile duct
8023091000006110		History of cancer of urethra
8023151000006117		History of cancer of unknown primary site
8024191000006113		Primary adenocarcinoma of skin
8024271000006117		Basal cell carcinoma of skin in situ
8024281000006119		Carcinoma in situ basal cell
8025631000006117		History of malignant neoplasm of pharynx
8025911000006117		Malignant glioma of cerebrum

8025931 0000061 11		Malignant glioma of hypothalamus
8025941 0000061 18		Malignant glioma of cerebellum
8025951 0000061 16		Malignant glioma of central nervous system
8026431 0000061 12		History of malignant neoplasm of peritoneum
8026491 0000061 11		History of malignant neoplasm of retroperitoneum
8026511 0000061 17		History of disseminated malignant neoplasm
8026531 0000061 11		History of malignant neoplasm of tonsil
8026551 0000061 16		History of ductal carcinoma in situ of breast
8026581 0000061 12		History of cancer of floor of mouth
8026601 0000061 19		History of malignant neoplasm of gum
8027721 0000061 18		Primary adenocarcinoma of vulva
8027731 0000061 15		Primary squamous cell carcinoma of chest wall
8027741 0000061 13		Primary adenocarcinoma of chest wall
8027781 0000061 19		Primary undifferentiated large cell malignant neoplasm of chest wall
8028011 0000061 10		Secondary adenocarcinoma of bone
8028021 0000061 19		Metastatic adenocarcinoma to bone
8029961 0000061 16		Malignant multiple endocrine neoplasia type 2a
8029971 0000061 11		Mucinous adenocarcinoma of gastrointestinal tract
8029981 0000061 14		Breast cancer detected by national screening programme

8030291000006112		Overlapping malignant neoplasm of colon and rectum
8030301000006113		Primary malignant inflammatory neoplasm of female breast
8030471000006110		Malignant neoplasm of rectosigmoid junction metastatic to brain
8030751000006113		History of squamous cell carcinoma in situ
8031221000006117		Primary adenocarcinoma of fallopian tube
8031231000006119		Primary adenocarcinoma of intestinal tract
8031241000006112		Primary adenocarcinoma of vagina
8031381000006111		Adenocarcinoma in situ in villous adenoma
8032421000006110		Sampling of vagina for Papanicolaou smear after hysterectomy for malignant disease done
8032821000006112		Primary squamous cell carcinoma of thymus
8032831000006110		Squamous cell carcinoma of vagina
8032841000006117		Squamous cell cancer of vagina
8033661000006110		Primary squamous cell carcinoma of urethra
8033671000006115		Primary transitional cell carcinoma of urethra
8033701000006119		Non-seminomatous germ cell neoplasm of testis
8033751000006115		Primary malignant mixed Mullerian neoplasm of endometrium
8033761000006118		Primary malignant Mullerian mixed tumour of endometrium
8033771000006113		Primary malignant Mullerian mixed tumor of endometrium
8033781000006111		Primary malignant clear cell neoplasm of endometrium

8033791000006114		Primary papillary serous cystadenocarcinoma of endometrium
8033801000006110		Primary adenosquamous carcinoma of endometrium
8035431000006117		History of malignant neoplasm of anus
8035501000006114		Cervical cancer Papanicolaou smear screening declined
8036351000006114		History of malignant melanoma of eye
8038291000006110		History of malignant cutaneous T-cell lymphoma
8038701000006112		Malignant poorly differentiated neuroendocrine carcinoma
8038821000006114		History of malignant neoplasm of fallopian tube
8040381000006117		Primary adenocarcinoma of distal third of oesophagus
8040391000006119		Primary adenocarcinoma of distal third of esophagus
8040401000006117		Primary adenocarcinoma of lower third of oesophagus
8040411000006119		Primary adenocarcinoma of lower third of esophagus
8041921000006114		Malignant neuroendocrine tumour
8041931000006112		Malignant neuroendocrine tumor
8042041000006110		Merkel cell carcinoma of upper limb
8042051000006112		Merkel cell carcinoma of lower limb
8042281000006111		History of neuroendocrine malignant neoplasm
8042711000006114		Myelopathy due to malignant neoplastic disease
8042721000006118		Myelopathy in malignant neoplastic disease

8043431000006117		History of merkel cell carcinoma
8043551000006113		History of sebaceous carcinoma
8043691000006115		History of malignant germ cell neoplasm of testis
8043701000006115		History of malignant germ cell neoplasm of mediastinum
8043841000006113		History of malignant neoplasm of duodenum
8043851000006110		History of malignant ameloblastoma of mandible
8044201000006114		History of transitional cell carcinoma of kidney
8044401000006110		History of gastrointestinal stromal neoplasm
8044861000006114		History of malignant germ cell neoplasm of ovary
8044871000006119		History of malignant ovarian germ cell tumor
8044881000006116		History of malignant ovarian germ cell tumour
8045681000006119		History of lobular carcinoma in situ
8046071000006115		History of carotid body neoplasm
8046351000006110		Primary malignant germ cell neoplasm
8046361000006112		Primary malignant germ cell tumor
8046371000006117		Primary malignant germ cell tumour
8046551000006119		Primary malignant astrocytoma of central nervous system
8047761000006110		History of malignant neoplasm of eye
8047771000006115		History of cancer of eye

8052651 0000061 15		Primary adenocarcinoma of anus
8052661 0000061 18		Primary adenocarcinoma of cervix uteri
8052671 0000061 13		Primary adenocarcinoma of uterine cervix
8052681 0000061 11		Primary adenocarcinoma of pelvis
8052691 0000061 14		Primary adenocarcinoma of rectosigmoid junction
8052701 0000061 14		Primary adenocarcinoma of small intestine
8067131 0000061 13		Primary adenocarcinoma of endocervix
8073481 0000061 17		Malignant glioma of eye
8076911 0000061 14		Primary squamous cell carcinoma of vermilion border of lip
8077261 0000061 17		Symptoms due to cancer
8078541 0000061 18		Under care of cancer primary healthcare multidisciplinary team
8081410 0000611 4	142 8.11	History of malignant neoplasm of bladder
8081510 0000611 1	142 4.00	Personal history of primary malignant neoplasm of breast
8081710 0000611 8	142 B.0 0	History of malignant neoplasm of ear, nose AND/OR throat
8081810 0000611 5	142 6.00	History of malignant neoplasm of female genital organ
8081910 0000611 7	142 1.00	History of malignant neoplasm of gastrointestinal tract
8082010 0000611 9	142 8.12	History of malignant neoplasm of kidney
8082210 0000611 2	142 3.11	History of malignant neoplasm of lung
8082410 0000611 7	142 Z.00	H/O: malignant neoplasm

80827100000611 3	142 5.00	History of primary malignant neoplasm of skin
80830100000611 0	142 8.00	History of malignant neoplasm of urinary system
8088291000006118		History of malignant carcinoid tumour of colon
8088301000006117		History of malignant carcinoid tumor of colon
8088341000006115		History of malignant carcinoid tumour of small intestine
8088351000006118		History of malignant carcinoid tumor of small intestine
8088361000006116		History of malignant carcinoid tumour of rectum
8088371000006111		History of malignant carcinoid tumor of rectum
8112161000006112		History of malignant meningioma of meninges of brain
8113461000006113		Secondary malignant neoplasm of right lung
8113471000006118		Cancer metastatic to right lung
8113511000006111		Overlapping primary malignant neoplasm of bone and articular cartilage of lower limb
8113521000006115		Overlapping primary malignant neoplasm of bone and articular cartilage of lower extremity
8113541000006110		Secondary malignant neoplasm of left lung
8113551000006112		Cancer metastatic to left lung
8113731000006110		Primary malignant neoplasm of left kidney
8113741000006117		Primary malignant neoplasm of right kidney
8126051000006115		[M]Classifications of neoplasm morphologies
8134541000006118		[M]Adenocarcinoma NOS

8135191000006116		[M]Malignant lymphoma, follicular centre cell, non-cleaved, follicular
81401000006110	B62z300	Unspec malig neop lymphoid/histiocytic intra-abdominal nodes
81421000006117	B62z100	Unspec malig neop lymphoid/histiocytic lymph node head/neck
81431000006119	B62z500	Unspec malig neop lymphoid/histiocytic nodes inguinal/leg
81451000006114	B62z200	Unspec malig neop lymphoid/histiocytic of intrathoracic node
81461000006111	B62z800	Unspec malig neop lymphoid/histiocytic of multiple sites
8160121000006116		[M] Adenoma and adenocarcinoma OS
8160231000006112		[M] Intracystic carcinoma NOS
8162851000006119		[M]Malignant melanoma NOS
8168871000006114		[M]Malignant lymphoma, small cell, non-cleaved, diffuse
8184861000006110		[M]Malignant lymphoma, large cell, non-cleaved, diffuse
8195631000006115		Lymphoedema due to malignant disease
82041000006113	B627W00	B-cell non-Hodgkin's lymphoma
8211141000006118		Primary adenocarcinoma of ascending colon
8211151000006116		Primary adenocarcinoma of body of pancreas
8211161000006119		Primary adenocarcinoma of body of stomach
8211181000006112		Primary adenocarcinoma of head of pancreas
8211451000006113		Extensive stage primary small cell carcinoma of lung
8212171000006115		Intraductal papillary mucinous neoplasm of pancreas

8212301000006112		History of malignant neoplasm of parotid gland
8223921000006116		Colorectal cancer
8232021000006114		Grade 1 (Stage pTa) papillary urothelial/transitional cell carcinoma
8232041000006119		Grade 2 (Stage pTa) papillary urothelial/transitional cell carcinoma
8232061000006115		Grade 3 (Stage pTa) papillary urothelial/transitional cell carcinoma
8242310000006111	B625.11	Histiocytosis X (acute, progressive)
8258410000006115	A788W00	HIV disease resulting in unspecified malignant neoplasm
8261210000006114	B61z.00	Hodgkin's disease NOS
8261710000006110	B61z500	Hodgkin's disease of lymph nodes of inguinal region AND/OR lower limb
8261810000006113	B61z400	Hodgkin's disease of lymph nodes of axilla AND/OR upper limb
8262610000006110	B613.00	Hodgkin's disease, lymphocytic-histiocytic predominance
8264710000006116	B616400	Hodgkin's disease, lymphocytic depletion of lymph nodes of axilla and upper limb
8265610000006112	B615200	Hodgkin's disease, mixed cellularity of intrathoracic lymph nodes
8265710000006117	B615100	Hodgkin's disease, mixed cellularity of lymph nodes of head, face and neck
8266110000006110	B614100	Hodgkin's disease, nodular sclerosis of lymph nodes of head, face and neck
8266310000006116	B614300	Hodgkin's disease, nodular sclerosis of intra-abdominal lymph nodes
8266510000006111	B614200	Hodgkin's disease, nodular sclerosis of intrathoracic lymph nodes
8266610000006113	B614400	Hodgkin's disease, nodular sclerosis of lymph nodes of axilla and upper limb
8266710000006118	B614800	Hodgkin's disease, nodular sclerosis of lymph nodes of multiple sites

8266810 0000611 5	B61 0.00	Hodgkin lymphoma, nodular lymphocyte predominance (clinical)
8267510 0000611 8	B61 010 0	Hodgkin's paragranuloma of lymph nodes of head, face, and neck
8269110 0000611 6	B61 350 0	Hodgkin's disease, lymphocytic-histiocytic predominance of lymph nodes of inguinal region and lower limb
8269210 0000611 2	B61 330 0	Hodgkin's disease, lymphocytic-histiocytic predominance of intra-abdominal lymph nodes
8269310 0000611 0	B61 360 0	Hodgkin's disease, lymphocytic-histiocytic predominance of intrapelvic lymph nodes
8269410 0000611 7	B61 320 0	Hodgkin's disease, lymphocytic-histiocytic predominance of intrathoracic lymph nodes
8269510 0000611 5	B61 310 0	Hodgkin's disease, lymphocytic-histiocytic predominance of lymph nodes of head, face and neck
8269910 0000611 4	B61 300 0	Hodgkin's disease, lymphocytic-histiocytic predominance (clinical)
8277551 0000061 11		H/O malignant hyperthermia due to anaesthesia
8282541 0000061 19		Interstitial photodynamic therapy of malignant tumour of parotid gland
8284301 0000061 17		Management of cancer
8290581 0000061 11		Re-excision of local recurrence of breast cancer
8290631 0000061 14		Wide excision of local recurrence of breast cancer
8308571 0000061 16		Malignant neoplasm of foot and ankle
8308581 0000061 18		Malignant tumour of foot and ankle
8413310 0000611 5	B1z 0.11	Cancer of bowel
8449510 0000611 1	4M 0..0 0	Gleason grade finding for prostatic cancer
8454101 0000061 10		Primary adenocarcinoma of lower lobe of left lung
8454131 0000061 19		Primary adenocarcinoma of upper lobe of left lung

845418100000061 18		Primary adenocarcinoma of lower lobe of right lung
845424100000061 15		Primary adenocarcinoma of upper lobe of right lung
845442100000061 14		Primary basal cell carcinoma of left upper limb
845444100000061 19		Primary basal cell carcinoma of left ear
845446100000061 15		Primary basal cell carcinoma of left eyelid
845449100000061 11		Primary basal cell carcinoma of left lower limb
845452100000061 13		Primary basal cell carcinoma of right upper limb
845454100000061 18		Primary basal cell carcinoma of right ear
845456100000061 19		Primary basal cell carcinoma of right eyelid
845459100000061 10		Primary basal cell carcinoma of right lower limb
845558100000061 13		Infiltrating ductal carcinoma of central portion of left female breast
845563100000061 11		Infiltrating ductal carcinoma of upper inner quadrant of left female breast
845565100000061 16		Infiltrating ductal carcinoma of upper outer quadrant of left female breast
845569100000061 10		Infiltrating ductal carcinoma of central portion of right female breast
845575100000061 14		Infiltrating ductal carcinoma of upper inner quadrant of right female breast
845577100000061 16		Infiltrating ductal carcinoma of upper outer quadrant of right female breast
845580100000061 19		Infiltrating lobular carcinoma of left female breast
845590100000061 13		Infiltrating lobular carcinoma of right female breast
845661100000061 12		Malignant melanoma of left choroid

8456661000006110		Malignant melanoma of right choroid
8456721000006112		Malignant melanoma of skin of left lower limb
8457381000006110		Recurrent primary malignant neoplasm of left female breast
8457401000006110		Recurrent primary malignant neoplasm of right female breast
8458001000006116		Primary squamous cell carcinoma of left ear
8458041000006119		Primary squamous cell carcinoma of skin of left lower limb
8458151000006118		Primary squamous cell carcinoma of right ear
8458201000006110		Primary squamous cell carcinoma of skin of right lower limb
8458271000006116		Transitional cell carcinoma of left renal pelvis
8458301000006119		Transitional cell carcinoma of right renal pelvis
8458331000006110		Transitional cell carcinoma of right ureter
8468271000006112		History of cancer metastatic to lymph nodes
8468281000006110		History of cancer metastatic to skin
8468291000006113		History of cancer metastatic to liver
8468301000006114		History of cancer metastatic to lung
8468311000006112		History of cancer metastatic to brain
8468321000006116		Recurrent malignant neoplasm of prostate
8468411000006119		History of cancer metastatic to bone
8468501000006116		Hereditary non-polyposis colon cancer gene mutation positive

8468511000006118		HNPCC (hereditary non-polyposis colon cancer) gene mutation positive
85315100000006113		Basal cell carcinoma
87968100000006114	B00..99	Lip carcinoma
87969100000006112	B01..99	Tongue carcinoma
88026100000006119	B34..99	Carcinoma breast
88035100000006110	B4...99	Carcinoma genital organs
88038100000006119	B43..99	Carcinoma body of uterus
88046100000006118	B49..99	Carcinoma bladder
88055100000006119	B55..99	Other primary carcinomas
88078100000006115	B6...99	Lymphatic tissue carcinoma
88118100000006112	B830.99	Carcinoma in situ breast
88119100000006110	B831.99	Carcinoma in situ cervix uteri
88122100000006115	B911099	Hydatidiform mole - malignant
88128100000006116	BB02.98	Malignant neoplasm NOS
88129100000006118	BB02.99	Malignant neoplasms
88130100000006117	BB13.99	Metastatic Carcinoma
90697100000006110		[RFC] Cancer of the prostate
90700100000006115		[RFC] Cancer of the rectum
90702100000006113		[RFC] Cancer of the testes

9070410 0000611 8		[RFC] Cancer of the ovary
9070610 0000611 9		[RFC] Cancer of the uterus
9070910 0000611 0		[RFC] Cancer of the cervix
9071010 0000611 6		[RFC] Cancer of the vagina & vulva
9071110 0000611 8		[RFC] Lung cancer
9071210 0000611 4		[RFC] Cancer of the larynx
9072610 0000611 0		[RFC] Neoplasm
9073010 0000611 8		[RFC] Following surgery for malignant or benign neoplasm
9073210 0000611 1		[RFC] With neoplasm but not receiving active treatment
9073310 0000611 4		[RFC] Bowel cancer
9073410 0000611 6		[RFC] Breast cancer
9073510 0000611 9		[RFC] Cancer of the thyroid
9073610 0000611 7		[RFC] Cancer of the eye
9073710 0000611 2		[RFC] Cancer of the stomach
9073810 0000611 0		[RFC] Liver cancer
9073910 0000611 3		[RFC] Cancer of the oesophagus
9074010 0000611 0		[RFC] Cancer of the mouth
9074310 0000611 9		[RFC] Neoplasm
9087810 0000611 0		[RFC] Cancer

91019100000611 3		[RFC] Cancer prevention
9318441000006112		Primary malignant neoplasm of uterus
9323141000006118		Malignant neoplastic disease in mother complicating childbirth
9323151000006116		Cancer in childbirth
9323721000006114		Malignant neoplastic disease in pregnancy
93325100000611 3	B14 1.99	Adenocarcinoma of rectum
9344051000006115		History of malignant neoplasm of lip
9349001000006115		History of malignant carcinoid tumour of thymus
9349011000006117		History of malignant carcinoid tumor of thymus
9349021000006113		History of malignant neoplasm of middle ear
9349051000006116		History of malignant neoplasm of nasal cavity
9349681000006115		History of malignant carcinoid tumour of kidney
9349691000006117		History of malignant carcinoid tumor of kidney
9349701000006117		History of rectosigmoid junction cancer
9349711000006119		History of malignant neoplasm of rectosigmoid junction
9349721000006110		History of malignant carcinoid tumour of stomach
9349731000006113		History of malignant carcinoid tumor of stomach
9350571000006112		History of malignant carcinoid tumour of bronchus
9350581000006110		History of malignant carcinoid tumor of bronchus

9350611 0000061 19		History of malignant neoplasm of external ear
9351691 0000061 16		History of carcinoma in situ of breast
9352851 0000061 18		Small cell carcinoma
9478691 0000061 18		Squamous cell carcinoma of left lung
9478701 0000061 18		Squamous cell carcinoma of right lung
9479531 0000061 10		Secondary malignant neoplasm of bilateral adrenal glands
9479541 0000061 17		Metastatic malignant neoplasm to bilateral adrenal glands
9616461 0000061 16		Secondary malignant neoplasm of lymph nodes of neck from thyroid
9616471 0000061 11		Metastatic malignant neoplasm of lymph nodes of neck from thyroid
9638541 0000061 13		Excision of hepatic neoplasm
9800210 0000611 3	B43 021 1	Malignant neoplasm of endometrium
9828010 0000611 2		Recurrence of cancer confirmed
9835031 0000061 11		Primary malignant neoplasm of both ovaries
9835041 0000061 18		Primary malignant neoplasm of bilateral ovaries
9835051 0000061 16		Primary malignant neoplasm of bilateral female breasts
9868051 0000061 15		Secondary adenocarcinoma of bilateral lungs
9868061 0000061 18		Metastatic adenocarcinoma to bilateral lungs
9868091 0000061 14		Adenocarcinoma of right lung
9887710 0000611 3	B62 ..99	Lymphatic tissue carcinoma

98880100000611 0	B59 1.99	Malignant neoplasm NOS
98940910000061 17		Cerebellar neoplasm sample

Chronic Pain

12122351000006111		Bilateral chronic pain following total hip arthroplasty
12122361000006113		Chronic pain following bilateral total hip arthroplasty
12122371000006118		Chronic pain following total arthroplasty of both hips
12126301000006113		Bilateral chronic pain of upper limbs
12126311000006111		Chronic pain of bilateral upper limbs
12126321000006115		Chronic pain of both upper limbs
12221131000006113		Chronic painful polyneuropathy co-occurrent and due to diabetes mellitus
136713011	1M52.00	Chronic pain
1841501000006118		Chronic pain review
2225421000000111	66n..00	Chronic pain review
2724141000006113		Alteration in comfort: chronic pain
297551015	F372100	Chronic painful diabetic neuropathy
318040019	Ryu7000	[X]Other chronic pain
3515749014		Chronic pain due to malignant neoplastic disease
3527381016		Primary chronic pain
3527382011		Chronic pain secondary to viscus structure
3561159012		Referral to chronic pain management service
3636662019		Chronic pain following trauma
3636666016		Chronic pain secondary to musculoskeletal structure
3636901014		Chronic pain following surgical procedure for cancer
3636902019		Chronic painful chemotherapy-induced polyneuropathy
3636903012		Chronic painful polyneuropathy following chemotherapy
3636906016		Chronic pain following radiotherapy
5009561000006114		Diabetic chronic painful polyneuropathy
5103021000006116		Chronic pain in female pelvis
6383361000006117		Chronic pain syndrome
6770101000006118		Chronic pain control
6770111000006115		Chronic pain control assessment
6770121000006111		Assess chronic pain control
6770131000006114		Chronic pain control education
6770141000006116		Teach chronic pain control
6770151000006119		Chronic pain control management
6770161000006117		Manage chronic pain control
6954401000006113		Consultation for chronic pain
6954411000006111		Chronic pain consultation
7062031000006118		Chronic pain in vagina
7138771000006116		Chronic pain due to injury
7163611000006117		Chronic pain in face
7254011000006115		Chronic pain, psychogenic
7567261000006119		Chronic pain clinic

7716811000006118		Post-mastectomy chronic pain syndrome
7991631000006113		Chronic pain due to malignancy
8036281000006111		Psychosocial dysfunction due to chronic pain
8040761000006111		Chronic pain in coccyx for more than three months
8042001000006113		Chronic pain in male pelvis
8253141000006113		Referral to chronic pain management service
8435291000006112		Chronic Pain TOM (Therapy Outcome Measure) activity score
8435301000006113		Chronic Pain TOM (Therapy Outcome Measure) carer wellbeing score
8435311000006111		Chronic Pain TOM (Therapy Outcome Measure) impairment score
8435321000006115		Chronic Pain TOM (Therapy Outcome Measure) participation score
8435331000006117		Chronic Pain TOM (Therapy Outcome Measure) wellbeing score
9493181000006113		Bilateral total knee chronic pain following arthroplasty
9493191000006111		Chronic pain following bilateral total knee arthroplasty
9493211000006112		Chronic pain following left total hip arthroplasty
9493221000006116		Chronic pain following left total knee arthroplasty
9493231000006118		Chronic pain following right total hip arthroplasty
9493251000006113		Chronic pain following right total knee arthroplasty
9847831000006111		Chronic pain of right upper limb
9847841000006118		Chronic pain of left upper limb
9872891000006113		Chronic pain of right foot
9872911000006110		Chronic pain of left foot

Chronic Kidney Disease

12716071000006117		Predicted stage chronic kidney disease
1573151000006111		No evidence of chronic kidney disease
1770901000006119		Chronic kidney disease diagnosis reviewed
1823941000006114		Chronic kidney disease confirmed
1825111000006112		Chronic kidney disease diagnosis discussed with patient
1854991000006119		EGFR 3 month repeat test for CKD confirmatory
1932031000006119		3D study - problems with chronic kidney disease management
1940501000006119	1Z1a.00	CKD G4A1 - chronic kidney disease with glomerular filtration rate category G4 and albuminuria category A1
1940511000006116	1Z1b.00	CKD G4A2 - chronic kidney disease with glomerular filtration rate category G4 and albuminuria category A2
1940521000006112	1Z1c.00	CKD G4A3 - chronic kidney disease with glomerular filtration rate category G4 and albuminuria category A3
1940531000006110	1Z1d.00	CKD G5A1 - chronic kidney disease with glomerular filtration rate category G5 and albuminuria category A1
1940541000006117	1Z1e.00	CKD G5A2 - chronic kidney disease with glomerular filtration rate category G5 and albuminuria category A2
1940551000006115	1Z1f.00	CKD G5A3 - chronic kidney disease with glomerular filtration rate category G5 and albuminuria category A3
1940561000006118	1Z1M.00	CKD G1A1 - chronic kidney disease with glomerular filtration rate category G1 and albuminuria category A1
1940571000006113	1Z1N.00	CKD G1A2 - chronic kidney disease with glomerular filtration rate category G1 and albuminuria category A2
1940581000006111	1Z1P.00	CKD G1A3 - chronic kidney disease with glomerular filtration rate category G1 and albuminuria category A3
1940591000006114	1Z1Q.00	CKD G2A1 - chronic kidney disease with glomerular filtration rate category G2 and albuminuria category A1

1940601000006118	1Z1R.00	CKD G2A2 - chronic kidney disease with glomerular filtration rate category G2 and albuminuria category A2
1940611000006115	1Z1S.00	CKD G2A3 - chronic kidney disease with glomerular filtration rate category G2 and albuminuria category A3
1940621000006111	1Z1T.00	CKD G3aA1 - chronic kidney disease with glomerular filtration rate category G3a and albuminuria category A1
1940631000006114	1Z1V.00	CKD G3aA2 - chronic kidney disease with glomerular filtration rate category G3a and albuminuria category A2
1940641000006116	1Z1W.00	CKD G3aA3 - chronic kidney disease with glomerular filtration rate category G3a and albuminuria category A3
1940651000006119	1Z1X.00	CKD G3bA1 - chronic kidney disease with glomerular filtration rate category G3b and albuminuria category A1
1940661000006117	1Z1Y.00	CKD G3bA2 - chronic kidney disease with glomerular filtration rate category G3b and albuminuria category A2
1940671000006112	1Z1Z.00	CKD G3bA3 - chronic kidney disease with glomerular filtration rate category G3b and albuminuria category A3
2002951000006111		Chronic kidney disease 2 monthly review
2002961000006113		Chronic kidney disease 3 monthly review
2002971000006118		Chronic kidney disease 4 monthly review
2323001000000115	4Z1..00	Chronic kidney disease laboratory study
2767154014	K055.00	Chronic kidney disease stage 5
2767383018	K051.00	Chronic kidney disease stage 1
2767384012	K052.00	Chronic kidney disease stage 2
2767385013	K054.00	Chronic kidney disease stage 4
2771041011	K05..13	Chronic kidney disease
2773184015	K053.00	Chronic kidney disease stage 3
304031000000117	1Z10.00	Chronic kidney disease stage 1
304051000000112	1Z11.00	Chronic kidney disease stage 2
304071000000115	1Z12.00	Chronic kidney disease stage 3
304091000000116	1Z13.00	Chronic kidney disease stage 4
304111000000114	1Z14.00	Chronic kidney disease stage 5
408381000000119	6AA..00	Chronic kidney disease annual review
408661000000117	9Ot5.00	Predicted stage chronic kidney disease
557811000000119	1Z15.00	Chronic kidney disease stage 3A
557831000000110	1Z16.00	Chronic kidney disease stage 3B
595541000000119	1Z17.00	Chronic kidney disease stage 1 with proteinuria
595601000000118	1Z18.00	Chronic kidney disease stage 1 without proteinuria
595661000000119	1Z19.00	Chronic kidney disease stage 2 with proteinuria
595721000000116	1Z1A.00	Chronic kidney disease stage 2 without proteinuria
595811000000117	1Z1B.00	Chronic kidney disease stage 3 with proteinuria
595871000000110	1Z1C.00	Chronic kidney disease stage 3 without proteinuria
595931000000116	1Z1D.00	Chronic kidney disease stage 3A with proteinuria
595991000000115	1Z1E.00	Chronic kidney disease stage 3A without proteinuria
596051000000115	1Z1F.00	Chronic kidney disease stage 3B with proteinuria
596131000000114	1Z1G.00	Chronic kidney disease stage 3B without proteinuria
596191000000110	1Z1H.00	Chronic kidney disease stage 4 with proteinuria
596261000000111	1Z1J.00	Chronic kidney disease stage 4 without proteinuria
596321000000110	1Z1K.00	Chronic kidney disease stage 5 with proteinuria
596401000000111	1Z1L.00	Chronic kidney disease stage 5 without proteinuria
618391000000118	1Z17.11	CKD stage 1 with proteinuria
618401000000115	1Z18.11	CKD stage 1 without proteinuria

618411000000118	1Z19.11	CKD stage 2 with proteinuria
618421000000112	1Z1A.11	CKD stage 2 without proteinuria
618431000000114	1Z1B.11	CKD stage 3 with proteinuria
618441000000117	1Z1C.11	CKD stage 3 without proteinuria
618451000000119	1Z1D.11	CKD stage 3A with proteinuria
618461000000116	1Z1E.11	CKD stage 3A without proteinuria
618471000000111	1Z1F.11	CKD stage 3B with proteinuria
618481000000113	1Z1G.11	CKD stage 3B without proteinuria
618491000000110	1Z1H.11	CKD stage 4 with proteinuria
618501000000116	1Z1J.11	CKD stage 4 without proteinuria
618511000000119	1Z1K.11	CKD stage 5 with proteinuria
618521000000113	1Z1L.11	CKD stage 5 without proteinuria
7147741000006114		CKD stage 1
7147761000006113		CKD stage 2
7147781000006115		CKD stage 4
7174791000006111		CKD stage 3
7174851000006111		CKD stage 5
7290571000006115		Erythropoietin resistance in anaemia of chronic kidney disease
7290581000006117		Erythropoietin resistance in anemia of chronic kidney disease
7480371000006113		H/O chronic kidney disease
7480381000006111		History of chronic kidney disease
7615331000006115		Anaemia in chronic kidney disease
7615341000006113		Anemia co-occurrent and due to chronic kidney disease
7615351000006110		Anaemia co-occurrent and due to chronic kidney disease
7615361000006112		Anemia in chronic kidney disease
7643311000006115		CKD - chronic kidney disease
7702321000006115		Chronic kidney disease mineral and bone disorder
7702331000006117		CKD-MBD - chronic kidney disease mineral and bone disorder
7714891000006118		Chronic kidney disease stage 5 on dialysis
7714901000006119		Chronic kidney disease 5d
7714911000006116		CKD (chronic kidney disease) stage 5d
7714921000006112		Chronic kidney disease stage 5 with transplant
7714931000006110		Chronic kidney disease 5t
7714941000006117		CKD (chronic kidney disease) stage 5t
7813391000006114		Low alkaline phosphatase due to chronic kidney disease
7813401000006111		Low alkaline phosphatase in chronic kidney disease
7827261000006111		Chronic kidney disease following donor nephrectomy
7827951000006116		Chronic kidney disease following excision of renal neoplasm
7827961000006119		Chronic kidney disease due to tumor nephrectomy
7827971000006114		Chronic kidney disease due to tumour nephrectomy
7827981000006112		Chronic kidney disease due to systemic infection
7832091000006112		Chronic kidney disease due to traumatic loss of kidney
7966611000006114		Chronic kidney disease stage 5 due to type 2 diabetes mellitus
7966631000006115		Chronic kidney disease stage 5 due to type II diabetes mellitus
7966641000006113		Chronic kidney disease stage 4 due to type 2 diabetes mellitus
7966661000006112		Chronic kidney disease stage 4 due to type II diabetes mellitus
7966691000006116		Chronic kidney disease stage 3 due to type 2 diabetes mellitus
7966711000006118		Chronic kidney disease stage 3 due to type II diabetes mellitus

7966761000006115		Chronic kidney disease stage 2 due to type 2 diabetes mellitus
7966781000006113		Chronic kidney disease stage 2 due to type II diabetes mellitus
7966791000006111		Chronic kidney disease stage 1 due to type 2 diabetes mellitus
7966811000006110		Chronic kidney disease stage 1 due to type II diabetes mellitus
7966871000006118		Chronic kidney disease due to type 2 diabetes mellitus
7990031000006110		Hypertensive heart and chronic kidney disease
8001631000006110		Hypertensive heart AND chronic kidney disease with congestive heart failure
8022361000006113		Hypertension in chronic kidney disease due to type 2 diabetes mellitus
8022371000006118		Hypertension in chronic kidney disease due to type II diabetes mellitus
8022401000006115		Hypertension in chronic kidney disease due to type 1 diabetes mellitus
8022411000006117		Hypertension in chronic kidney disease due to type I diabetes mellitus
8027391000006115		Chronic kidney disease stage 1 due to type 1 diabetes mellitus
8027401000006118		Chronic kidney disease stage 1 due to type I diabetes mellitus
8027411000006115		Chronic kidney disease stage 2 due to type 1 diabetes mellitus
8027421000006111		Chronic kidney disease stage 2 due to type I diabetes mellitus
8027451000006119		Chronic kidney disease stage 3 due to type 1 diabetes mellitus
8027461000006117		Chronic kidney disease stage 3 due to type I diabetes mellitus
8027471000006112		Chronic kidney disease stage 4 due to type 1 diabetes mellitus
8027481000006110		Chronic kidney disease stage 4 due to type I diabetes mellitus
8027491000006113		Chronic kidney disease stage 5 due to type 1 diabetes mellitus
8027501000006117		Chronic kidney disease stage 5 due to type I diabetes mellitus
8030331000006117		Chronic kidney disease due to type 1 diabetes mellitus
8030341000006110		Chronic kidney disease due to type I diabetes mellitus
8030391000006118		Hypertensive heart AND chronic kidney disease on dialysis
8030411000006118		Hypertensive heart AND chronic kidney disease stage 5
8030421000006114		Hypertensive heart AND chronic kidney disease stage 4
8030431000006112		Hypertensive heart AND chronic kidney disease stage 3
8030441000006119		Hypertensive heart AND chronic kidney disease stage 2
8030451000006117		Hypertensive heart AND chronic kidney disease stage 1
8036941000006111		Chronic kidney disease stage 1 due to hypertension
8037161000006110		Pre-existing hypertensive chronic kidney disease in mother complicating pregnancy
8037171000006115		Preexisting hypertensive chronic kidney disease in pregnancy
8040661000006119		Chronic kidney disease stage 4 due to hypertension
8040671000006114		Chronic kidney disease stage 5 due to hypertension
8040681000006112		Chronic kidney disease stage 3 due to hypertension
8040691000006110		Chronic kidney disease stage 2 due to hypertension
8044011000006117		Hypertension in chronic kidney disease stage 5 due to type 2 diabetes mellitus
8044021000006113		Hypertension in chronic kidney disease stage 5 due to type II diabetes mellitus
8044041000006118		Hypertension in chronic kidney disease stage 4 due to type 2 diabetes mellitus
8044051000006116		Hypertension in chronic kidney disease stage 4 due to type II diabetes mellitus
8044061000006119		Hypertension in chronic kidney disease stage 3 due to type 2 diabetes mellitus
8044071000006114		Hypertension in chronic kidney disease stage 3 due to type II diabetes mellitus
8044081000006112		Hypertension in chronic kidney disease stage 2 due to type 2 diabetes mellitus

8044091000006110		Hypertension in chronic kidney disease stage 2 due to type II diabetes mellitus
8047971000006118		Malignant hypertensive chronic kidney disease stage 5
8087041000006116		Chronic kidney disease due to benign hypertension
8087051000006119		Chronic kidney disease stage 1 due to benign hypertension
8087061000006117		Chronic kidney disease stage 2 due to benign hypertension
8087071000006112		Chronic kidney disease stage 3 due to benign hypertension
8087081000006110		Chronic kidney disease stage 4 due to benign hypertension
8087091000006113		Chronic kidney disease stage 5 due to benign hypertension
8087641000006112		Malignant hypertensive chronic kidney disease
8087661000006111		Malignant hypertensive chronic kidney disease stage 1
8087681000006118		Malignant hypertensive chronic kidney disease stage 2
8087691000006115		Malignant hypertensive chronic kidney disease stage 3
8087701000006115		Malignant hypertensive chronic kidney disease stage 4
8104101000006118		CKD (chronic kidney disease stage) 1 with proteinuria
8104131000006114		CKD (chronic kidney disease) stage 1 without proteinuria
8104161000006117		CKD (chronic kidney disease) stage 2 with proteinuria
8104191000006113		CKD (chronic kidney disease) stage 2 without proteinuria
8104231000006115		CKD (chronic kidney disease) stage 3 with proteinuria
8104261000006112		CKD (chronic kidney disease) stage 3 without proteinuria
8104301000006115		CKD (chronic kidney disease) stage 3A with proteinuria
8104351000006116		CKD (chronic kidney disease) stage 3A without proteinuria
8104381000006112		CKD (chronic kidney disease) stage 3B with proteinuria
8104411000006110		CKD (chronic kidney disease) stage 3B without proteinuria
8104451000006111		CKD (chronic kidney disease) stage 4 with proteinuria
8104481000006115		CKD (chronic kidney disease) stage 4 without proteinuria
8104511000006111		CKD (chronic kidney disease) stage 5 with proteinuria
8104551000006112		CKD (chronic kidney disease) stage 5 without proteinuria
8212311000006110		Anaemia in chronic kidney disease stage 4
8212321000006119		Anemia co-occurrent and due to chronic kidney disease stage 4
8212331000006116		Anemia in chronic kidney disease stage 4
8212341000006114		Anaemia co-occurrent and due to chronic kidney disease stage 4
8212361000006113		Anaemia in chronic kidney disease stage 5
8212371000006118		Anemia co-occurrent and due to chronic kidney disease stage 5
8212381000006115		Anaemia co-occurrent and due to chronic kidney disease stage 5
8212391000006117		Anemia in chronic kidney disease stage 5
8212401000006115		Anaemia co-occurrent and due to chronic kidney disease stage 3
8212411000006117		Anemia co-occurrent and due to chronic kidney disease stage 3
8345541000006113		Chronic kidney disease with glomerular filtration rate category G1 and albuminuria category A1
8345571000006117		Chronic kidney disease with glomerular filtration rate category G1 and albuminuria category A2
8345611000006110		Chronic kidney disease with glomerular filtration rate category G1 and albuminuria category A3
8345651000006111		Chronic kidney disease with glomerular filtration rate category G2 and albuminuria category A1
8345681000006115		Chronic kidney disease with glomerular filtration rate category G2 and albuminuria category A2
8345731000006113		Chronic kidney disease with glomerular filtration rate category G2 and albuminuria category A3

8345881000006116		Chronic kidney disease with glomerular filtration rate category G3a and albuminuria category A1
8345901000006119		Chronic kidney disease with glomerular filtration rate category G3a and albuminuria category A2
8345931000006110		Chronic kidney disease with glomerular filtration rate category G3a and albuminuria category A3
8346001000006110		Chronic kidney disease with glomerular filtration rate category G3b and albuminuria category A1
8346021000006117		Chronic kidney disease with glomerular filtration rate category G3b and albuminuria category A2
8346041000006112		Chronic kidney disease with glomerular filtration rate category G3b and albuminuria category A3
8346101000006114		Chronic kidney disease with glomerular filtration rate category G4 and albuminuria category A1
8346121000006116		Chronic kidney disease with glomerular filtration rate category G4 and albuminuria category A2
8346151000006113		Chronic kidney disease with glomerular filtration rate category G4 and albuminuria category A3
8346171000006115		Chronic kidney disease with glomerular filtration rate category G5 and albuminuria category A1
8346201000006116		Chronic kidney disease with glomerular filtration rate category G5 and albuminuria category A2
8346221000006114		Chronic kidney disease with glomerular filtration rate category G5 and albuminuria category A3
9325291000006119		Pre-existing hypertensive heart and chronic kidney disease in mother complicating childbirth
9325301000006118		Preexisting hypertensive heart and chronic kidney disease in childbirth
9325341000006116		Pre-existing hypertensive heart and chronic kidney disease in mother complicating pregnancy
9325351000006119		Preexisting hypertensive heart and chronic kidney disease in pregnancy
994391000006115		Chronic kidney disease stage
994401000006118		Chronic kidney disease stage 1
994411000006115		Chronic kidney disease stage 2
994421000006111		Chronic kidney disease stage 3
994431000006114		Chronic kidney disease stage 4
994441000006116		Chronic kidney disease stage 5
8032721000006117		Chronic kidney disease due to hypertension

Constipation

medcode	readcode	desc
1726521000006115		Chronic constipation
25076018	19C..00	Constipation
2733891000006114		CN - Constipation
2735751000000118		Constipation care assessment
2791650019	J521100	Irritable bowel syndrome characterised by constipation
295382018	E264500	Psychogenic constipation
3007481000006116		Overflow incontinence due to constipation
303161017	J520.00	Constipation - functional
303162012	J520000	Acute constipation
303163019	J520200	Chronic constipation without overflow
303165014	J520y00	Other specified constipation
303166010	19CZ.00	Constipation NOS

3068881000006119		Slow transit constipation
3068891000006116		Colonic constipation
3068901000006117		Constipation by delayed colonic transit
3446051000006117		Alteration in bowel elimination: constipation
3446061000006115		Intermittent constipation pattern
353853017	J520400	Chronic constipation
3689851000006111		Encopresis with constipation AND overflow incontinence
3849221000006117		Chronic idiopathic constipation
3896451000006110		Constipation by outlet obstruction
4194671000006117		Intractable constipation
4786191000006112		Functional constipation
4786201000006110		Constipation-functional
484894013	J520100	Chronic constipation with overflow
5086181000006116		Irritable bowel syndrome variant of childhood with constipation
5089151000006118		Simple constipation
5272771000006119		Constipation alternates with diarrhoea
5272781000006116		Constipation alternates with diarrhea
590101000006113	J520z00	Constipation NOS
590111000006111	19C..11	Constipation symptom
630451000006116	J520300	Drug-induced constipation
6511391000006113		Constipation care
6511401000006110		Constipation management
7114441000006117		Constipation due to spasm of colon
7114451000006115		Spastic constipation
7159921000006114		Atonic constipation
7159931000006112		Constipation due to atony of colon
7171751000006119		Constipation due to neurogenic bowel
7171761000006117		Neurogenic constipation
7240101000006111		Constipation predominant irritable bowel syndrome
7240111000006114		Irritable bowel syndrome characterized by constipation
8042951000006113		Therapeutic opioid induced constipation
8062121000006113		Constipation due to pelvic floor outlet obstruction
906171000006112		[RFC] Constipation
909031000006119		[RFC] Constipation
982721000006114		Constipation

COPD

medcode	readcode	desc
105519017	H31..00	Chronic bronchitis
113497011	H322.00	Centrilobular emphysema
11932331000006115		COPD GOLD group A
11932341000006113		COPD GOLD group B
11932351000006110		COPD GOLD group C
11932361000006112		COPD GOLD group D

1222334016	H3y..11	Other specified chronic obstructive pulmonary disease
1222335015	H3z..11	Chronic obstructive pulmonary disease NOS
1230190015	H32y200	MacLeod's unilateral emphysema
123588010	H311.00	Mucopurulent chronic bronchitis
12489801000006116		Admit COPD emergency
12704691000006117		Interstitial emphysema
139979010	H311100	Fetid chronic bronchitis
1699821000006113	14OX.00	At risk of chronic obstructive pulmonary disease exacerbation
1765681000000110	14B3.12	History of chronic obstructive pulmonary disease
1813881000006119	9NgP.11	On COPD (chronic obstructive pulmonary disease) supportive care pathway
1882421000006113		1 COPD exacerbation in past year
1882431000006111		3+ COPD exacerbations in past year
1882441000006118		2 COPD exacerbations in past year
1882451000006116		No COPD exacerbations in past year
19421011	H581.00	Interstitial pulmonary emphysema
216596014	H3A..00	End stage chronic obstructive airways disease
2578881000006118		Panacinar emphysema
2578911000006118		Alveolar emphysema of lung
2678601000006119		Interstitial emphysema
27096010	H320200	Giant bullous emphysema
2716321000006116		COPD - Chronic obstructive pulmonary disease
2767261000006118		Mediastinal emphysema
2767421000006111		Obstructive emphysema
285100019	H312100	Emphysematous bronchitis
285104011	H312.00	Obstructive chronic bronchitis
301444018	H310z00	Simple chronic bronchitis NOS
301448015	H311z00	Mucopurulent chronic bronchitis NOS
301453013	H312200	Acute exacerbation of chronic obstructive airways disease
301455018	H312z00	Obstructive chronic bronchitis NOS
301456017	H313.00	Mixed simple and mucopurulent chronic bronchitis
301457014	H31y.00	Other chronic bronchitis
301458016	H31yz00	Other chronic bronchitis NOS
301459012	H31z.00	Chronic bronchitis NOS
301460019	H320.00	Chronic bullous emphysema
301463017	H320000	Segmental bullous emphysema
301464011	H320100	Zonal bullous emphysema
301468014	H320z00	Chronic bullous emphysema NOS
301469018	H32y.00	Other emphysema
301470017	H32y000	Acute vesicular emphysema
301477019	H32z.00	Emphysema NOS
301539010	H3y..00	Other specified chronic obstructive airways disease
301545019	H3z..00	Chronic obstructive airway disease

301572010	H46400 0	Chronic emphysema due to chemical fumes
301835010	Hyu300 0	[X]Other emphysema
3228301000006115		Unilateral emphysema
3295361000006115		Emphysema
3304321000006119		Occupational chronic bronchitis
3575061000006118		Chronic diffuse emphysema due to inhalation of chemical fumes AND/OR vapours
3575071000006113		Chronic diffuse emphysema due to inhalation of chemical fumes AND/OR vapors
3575081000006111		Chronic diffuse emphysema caused by inhalation of chemical fumes AND/OR vapors
3575091000006114		Chronic diffuse emphysema caused by inhalation of chemical fumes AND/OR vapours
3611791000006110		Centriacinar emphysema
3764021000006117		Interstitial emphysema of lung
3764031000006119		Interstitial pulmonary emphysema
3873191000006110		Fetid chronic bronchitis
3921361000006112		Emphysema of lung
396109014	H32y100	Atrophic (senile) emphysema
396110016	H32yz00	Other emphysema NOS
4374671000006119		Acute emphysema
4374681000006116		Chronic emphysema
457168017	H36..00	Mild chronic obstructive pulmonary disease
457169013	H37..00	Moderate chronic obstructive pulmonary disease
457171013	H38..00	Severe chronic obstructive pulmonary disease
457581000006111	H32y111	Acute interstitial emphysema
4732991000006119		Chronic bronchitis with emphysema
4733021000006111		Obstructive chronic bronchitis
475431013	H3...00	Chronic obstructive pulmonary disease
4781421000006111		Acute exacerbation of COPD
4781461000006117		Emphysematous bulla
4781471000006112		Bullous emphysema
4781821000006113		Chronic emphysema caused by chemical fumes
5054271000006111		Pulmonary emphysema in alpha-1 PI deficiency
5054281000006114		Pulmonary emphysema in alpha-1 primary immunodeficiency deficiency
506053014	H31100 0	Purulent chronic bronchitis
508561017	H310.00	Simple chronic bronchitis
508562012	H31000 0	Chronic catarrhal bronchitis
516801000000112	H39..00	Very severe chronic obstructive pulmonary disease
53589018	J650200	Acute emphysematous cholecystitis
5468391000006110		Emphysematous
553211000006119	H3y1.00	Chron obstruct pulmonary dis wth acute exacerbation, unspec
555461000006119	H3y0.00	Chronic obstructive pulmonary disease with acute lower respiratory infection
555471000006114	H3...11	COAD - Chronic obstructive airways disease
640491000006111	H32..00	Pulmonary emphysema
7049011000006110		Acute exacerbation of chronic bronchitis
7462014	SK07.00	Subcutaneous emphysema
7627141000006111		Pulmonary emphysema co-occurrent with fibrosis of lung
7627161000006110		Combined pulmonary fibrosis and emphysema syndrome
8058301000006110		COPD (chronic obstructive pulmonary disease) disturbs sleep

8058321000006117		COPD (chronic obstructive pulmonary disease) does not disturb sleep
8125721000006119		At risk of COPD (chronic obstructive pulmonary disease) exacerbation
8287171000006115		Acute non-infective exacerbation of COPD (chronic obstructive pulmonary disease)
8437811000006110		Number of hospital admissions due to COPD (chronic obstructive pulmonary disease) in past year
851261000006116		Chronic bronchitis, acute exac
90243017	SK07.11	Traumatic subcutaneous emphysema
909711000006111		[RFC] Chronic obstructive pulmonary disease (COPD)
909721000006115		[RFC] Emphysema
9317331000006113		Asthma-COPD overlap syndrome (ACOS)
9337016	H321.00	Panlobular emphysema

Diabetes Mellitus

medcode	readcode	desc
65526011	F381311	Diabetic amyotrophy
10928019		Diabetes mellitus associated with genetic syndrome
11004741000006116		Roche Diabetes Care Ltd
11633211000006112		Atypical diabetes mellitus
11633221000006116		ADM - atypical diabetes mellitus
1177721000000112	66As.00	Diabetic on subcutaneous treatment
11903141000006113		Diabetic oculopathy associated with type I diabetes mellitus
11927551000006115		Insulin dependent diabetes mellitus with ulcer
11927561000006118		Hypoglycemic coma co-occurrent and due to diabetes mellitus type II
11931861000006112		Pre-existing type 2 diabetes mellitus in pregnancy
12078901000006118		Cataract of right eye due to diabetes mellitus
12078911000006115		Cataract of right eye co-occurrent and due to diabetes mellitus
12078931000006114		Cataract of left eye due to diabetes mellitus
12078951000006119		Cataract of left eye co-occurrent and due to diabetes mellitus
12078981000006110		Cataract of bilateral eyes co-occurrent and due to diabetes mellitus
12078991000006113		Bilateral cataracts due to diabetes mellitus
12079071000006119		Iritis of right eye co-occurrent and due to diabetes mellitus
12079101000006112		Iritis of left eye co-occurrent and due to diabetes mellitus
12079111000006110		Iritis of left eye due to diabetes mellitus
12079121000006119		Bilateral iritis due to diabetes mellitus

120791410000 06114		Iritis of bilateral eyes co-occurrent and due to diabetes mellitus
120850310000 06111		Preproliferative retinopathy of right eye co-occurrent and due to diabetes mellitus
120850410000 06118		Preproliferative retinopathy of right eye due to diabetes mellitus
120850610000 06119		Preproliferative retinopathy of left eye co-occurrent and due to diabetes mellitus
120851110000 06118		Mild nonproliferative retinopathy of right eye co-occurrent and due to diabetes mellitus
120851510000 06117		Mild nonproliferative retinopathy of left eye co-occurrent and due to diabetes mellitus
120851910000 06111		Moderate nonproliferative retinopathy of right eye co-occurrent and due to diabetes mellitus
120852010000 06114		Moderate nonproliferative retinopathy of right eye due to diabetes mellitus
120852210000 06116		Moderate nonproliferative retinopathy of left eye co-occurrent and due to diabetes mellitus
120852410000 06111		Moderate nonproliferative retinopathy of left eye due to diabetes mellitus
120852610000 06110		Severe nonproliferative retinopathy of right eye co-occurrent and due to diabetes mellitus
120852710000 06115		Severe nonproliferative retinopathy of right eye due to diabetes mellitus
120853010000 06118		Severe nonproliferative retinopathy of left eye co-occurrent and due to diabetes mellitus
120853110000 06115		Severe nonproliferative retinopathy of left eye due to diabetes mellitus
120853610000 06117		Very severe nonproliferative retinopathy of right eye co-occurrent and due to diabetes mellitus
120853710000 06112		Very severe nonproliferative retinopathy of right eye due to diabetes mellitus
120854010000 06110		Very severe nonproliferative retinopathy of left eye co-occurrent and due to diabetes mellitus
120857410000 06115		Macular edema of right eye co-occurrent and due to diabetes mellitus
120857510000 06118		Macular oedema of right eye co-occurrent and due to diabetes mellitus
120857710000 06111		Macular oedema of left eye co-occurrent and due to diabetes mellitus
120857810000 06114		Macular edema of left eye co-occurrent and due to diabetes mellitus
120858010000 06113		Macular oedema due to type 1 diabetes mellitus
120858110000 06111		Macular edema due to type 1 diabetes mellitus
120858210000 06115		Macular edema co-occurrent and due to type 1 diabetes mellitus
120858310000 06117		Macular oedema co-occurrent and due to type 1 diabetes mellitus
120858410000 06110		Macular oedema due to type 2 diabetes mellitus
120858510000 06112		Macular edema co-occurrent and due to type 2 diabetes mellitus
120858610000 06114		Macular edema due to type 2 diabetes mellitus
120858710000 06119		Macular oedema co-occurrent and due to type 2 diabetes mellitus

120858910000 06118		Clinically significant macular oedema of right eye co-occurrent and due to diabetes mellitus
120859010000 06119		Clinically significant macular edema of right eye due to diabetes mellitus
120859110000 06116		Clinically significant macular edema of right eye co-occurrent and due to diabetes mellitus
120859210000 06112		Clinically significant macular oedema of left eye due to diabetes mellitus
120859310000 06110		Clinically significant macular oedema of left eye co-occurrent and due to diabetes mellitus
120859510000 06115		Clinically significant macular edema of left eye due to diabetes mellitus
120862910000 06111		Maculopathy of right eye due to diabetes mellitus
120863010000 06112		Disorder of right macula co-occurrent and due to diabetes mellitus
120863210000 06119		Maculopathy of left eye due to diabetes mellitus
120863410000 06114		Disorder of left macula co-occurrent and due to diabetes mellitus
121024910000 06115		Cervical radiculoplexus neuropathy co-occurrent and due to diabetes mellitus
121025010000 06111		Cervical radiculoplexus neuropathy with diabetes mellitus
121025210000 06118		Cranial nerve palsy with diabetes mellitus
121025310000 06115		Cranial nerve palsy due to diabetes mellitus
121025410000 06113		Cranial nerve palsy co-occurrent and due to diabetes mellitus
121025510000 06110		Erectile dysfunction with diabetes mellitus
121025610000 06112		Erectile dysfunction co-occurrent and due to diabetes mellitus
121025710000 06117		Clinically significant macular oedema with diabetes mellitus
121025810000 06119		Clinically significant macular oedema co-occurrent and due to diabetes mellitus
121025910000 06116		Clinically significant macular edema due to diabetes mellitus
121026010000 06112		Clinically significant macular edema co-occurrent and due to diabetes mellitus
121026110000 06110		Clinically significant macular oedema due to diabetes mellitus
121026210000 06119		Clinically significant macular edema with diabetes mellitus
121026310000 06116		Cranial nerve palsy with type 1 diabetes mellitus
121026410000 06114		Cranial nerve palsy due to type 1 diabetes mellitus
121026510000 06111		Cranial nerve palsy with type I diabetes mellitus
121026610000 06113		Cranial nerve palsy co-occurrent and due to type 1 diabetes mellitus
121061610000 06119		Retinal oedema with diabetes mellitus
121061710000 06114		Retinal edema due to diabetes mellitus

121061810000 06112		Retinal oedema due to diabetes mellitus
121061910000 06110		Retinal oedema co-occurrent and due to diabetes mellitus
121062010000 06113		Retinal edema with diabetes mellitus
121062110000 06111		Retinal edema co-occurrent and due to diabetes mellitus
121062210000 06115		Retinal ischaemia with diabetes mellitus
121062310000 06117		Retinal ischemia due to diabetes mellitus
121062410000 06110		Retinal ischaemia co-occurrent and due to diabetes mellitus
121062510000 06112		Retinal ischaemia due to diabetes mellitus
121062610000 06114		Retinal ischemia with diabetes mellitus
121062710000 06119		Retinal ischemia co-occurrent and due to diabetes mellitus
121196810000 06110		Ketosis-prone diabetes mellitus
121196910000 06113		KPDM - ketosis-prone diabetes mellitus
121197010000 06113		KPD - ketosis-prone diabetes mellitus
121589010	C10..0 0	Diabetes mellitus
121661010000 06119		Retinopathy co-occurrent and due to diabetes mellitus
121661110000 06116		Retinopathy due to diabetes mellitus
121722810000 06112		Pseudotabes co-occurrent and due to diabetes mellitus
121748010000 06114		Eye disorder due to diabetes mellitus
121748110000 06112		Disorder of eye co-occurrent and due to diabetes mellitus
121803910000 06110		Diarrhea co-occurrent and due to diabetes mellitus
121804010000 06112		Diarrhoea with diabetes mellitus
121804110000 06110		Diarrhea with diabetes mellitus
121804210000 06119		Diarrhoea co-occurrent and due to diabetes mellitus
121807710000 06113		Lumbosacral radiculoplexus neuropathy with diabetes mellitus
121807810000 06111		Lumbosacral radiculoplexus neuropathy co-occurrent and due to diabetes mellitus
121808410000 06112		Symmetric proximal motor neuropathy with diabetes mellitus
121808510000 06114		Symmetric proximal motor neuropathy co-occurrent and due to diabetes mellitus
121809110000 06118		Radiculoplexus neuropathy co-occurrent and due to diabetes mellitus
121809210000 06114		Radiculoplexus neuropathy with diabetes mellitus

121832210000 06110		Cataract co-occurrent and due to diabetes mellitus
121832310000 06113		Cataract due to diabetes mellitus
121854210000 06113		Polyneuropathy due to diabetes mellitus
121854310000 06111		Polyneuropathy co-occurrent and due to diabetes mellitus
121860110000 06117		Autonomic neuropathy with diabetes mellitus
121860210000 06113		Autonomic neuropathy co-occurrent and due to diabetes mellitus
121898810000 06112		Proliferative retinopathy with diabetes mellitus
121899010000 06114		Proliferative retinopathy co-occurrent and due to diabetes mellitus
121992810000 06112		Asymmetric proximal motor neuropathy co-occurrent and due to diabetes mellitus
121992910000 06110		Asymmetric proximal motor neuropathy with diabetes mellitus
122002410000 06114		Mononeuropathy simplex co-occurrent and due to diabetes mellitus
122002510000 06111		Mononeuropathy simplex with diabetes mellitus
1220321013	L1808 00	Diabetes mellitus arising in pregnancy
122184710000 06119		Mixed sensorimotor polyneuropathy co-occurrent and due to diabetes mellitus
122184810000 06116		Motor polyneuropathy co-occurrent and due to diabetes mellitus
122184910000 06118		Sensory neuropathy co-occurrent and due to diabetes mellitus
122185010000 06114		Disorder of kidney co-occurrent and due to diabetes mellitus
122185110000 06112		Kidney disorder due to diabetes mellitus
122206410000 06117		Mononeuritis multiplex co-occurrent and due to diabetes mellitus
122206510000 06115		Mononeuritis multiplex with diabetes mellitus
122206610000 06118		Asymptomatic neuropathy co-occurrent and due to diabetes mellitus
122206710000 06113		Asymptomatic neuropathy with diabetes mellitus
122206910000 06114		Advanced maculopathy co-occurrent and due to diabetes mellitus
122207010000 06114		Advanced maculopathy with diabetes mellitus
122207110000 06112		Iritis co-occurrent and due to diabetes mellitus
122207210000 06116		Iritis due to diabetes mellitus
122211110000 06119		Neuropathy co-occurrent and due to diabetes mellitus
122211210000 06110		Acute painful polyneuropathy co-occurrent and due to diabetes mellitus
122211310000 06113		Chronic painful polyneuropathy co-occurrent and due to diabetes mellitus

122211410000 06115		Asymmetric polyneuropathy co-occurrent and due to diabetes mellitus
122211510000 06118		Mononeuropathy co-occurrent and due to diabetes mellitus
122211610000 06116		Thoracic radiculopathy co-occurrent and due to diabetes mellitus
122211710000 06111		Thoracic radiculopathy with diabetes mellitus
122212410000 06112		Disorder of macula co-occurrent and due to diabetes mellitus
122212510000 06114		Maculopathy due to diabetes mellitus
122212610000 06111		Maculopathy with diabetes mellitus
122212710000 06116		Traction retinal detachment with diabetes mellitus
122212810000 06118		Traction retinal detachment co-occurrent and due to diabetes mellitus
122214210000 06118		Soft tissue disorder due to diabetes mellitus
122214310000 06115		Disorder of soft tissue co-occurrent and due to diabetes mellitus
122245810000 06118		Advanced retinal disease co-occurrent and due to diabetes mellitus
122245910000 06115		Advanced retinal disease with diabetes mellitus
122247210000 06114		Mild nonproliferative retinopathy co-occurrent and due to diabetes mellitus
122247410000 06119		Moderate nonproliferative retinopathy co-occurrent and due to diabetes mellitus
122247510000 06117		Severe nonproliferative retinopathy co-occurrent and due to diabetes mellitus
122247710000 06110		Vitreous haemorrhage with diabetes mellitus
122247810000 06113		Vitreous hemorrhage with diabetes mellitus
122247910000 06111		Vitreous hemorrhage co-occurrent and due to diabetes mellitus
122248010000 06112		Vitreous haemorrhage co-occurrent and due to diabetes mellitus
122248110000 06110		Macular oedema co-occurrent and due to diabetes mellitus
122248210000 06119		Macular edema co-occurrent and due to diabetes mellitus
122248310000 06116		Macular edema due to diabetes mellitus
122248410000 06114		Macular oedema due to diabetes mallitus
122249010000 06118		Focal exudative maculopathy co-occurrent and due to diabetes mellitus
122249210000 06111		Ischemic maculopathy co-occurrent and due to diabetes mellitus
122249310000 06114		Ischaemic maculopathy co-occurrent and due to diabetes mellitus
122249410000 06116		Ischemic maculopathy with diabetes mellitus
122249510000 06119		Ischaemic maculopathy with diabetes mellitus

122249610000 06117		Mixed maculopathy co-occurrent and due to diabetes mellitus
122249710000 06112		Mixed maculopathy due to diabetes mellitus
122249810000 06110		Mixed maculopathy with diabetes mellitus
1223147012	C10FJ1 1	Insulin treated Type II diabetes mellitus
1223148019	C109J1 1	Insulin treated non-insulin dependent diabetes mellitus
122730510000 06117		Femoral mononeuropathy co-occurrent and due to diabetes mellitus
122730610000 06115		Femoral mononeuropathy with diabetes mellitus
122954410000 06116		Nonproliferative retinopathy co-occurrent and due to diabetes mellitus
123003410000 06112		Very severe nonproliferative retinopathy without macular edema co-occurrent and due to diabetes mellitus
123003510000 06114		Very severe nonproliferative retinopathy without macular oedema co-occurrent and due to diabetes mellitus
123003810000 06118		Macular edema not clinically significant with diabetes mellitus
123003910000 06115		Macular oedema not clinically significant with diabetes mellitus
123004010000 06118		Macular edema not clinically significant co-occurrent and due to diabetes mellitus
123004110000 06115		Macular oedema not clinically significant co-occurrent and due to diabetes mellitus
123004210000 06111		Severe nonproliferative retinopathy with clinically significant macular edema co-occurrent and due to diabetes mellitus
123004310000 06114		Severe nonproliferative retinopathy with clinically significant macular oedema co-occurrent and due to diabetes mellitus
123004610000 06117		Severe nonproliferative retinopathy without macular edema co-occurrent and due to diabetes mellitus
123004710000 06112		Severe nonproliferative retinopathy without macular oedema co-occurrent and due to diabetes mellitus
123005010000 06117		Very severe nonproliferative retinopathy co-occurrent and due to diabetes mellitus
123005210000 06110		Very severe nonproliferative retinopathy with clinically significant macular edema co-occurrent and due to diabetes mellitus
123005310000 06113		Very severe nonproliferative retinopathy with clinically significant macular oedema co-occurrent and due to diabetes mellitus
1230890017	F1711 00	Autonomic neuropathy due to diabetes
1230929011	C10G. 00	Secondary pancreatic diabetes mellitus
1231774017		Malnutrition-related diabetes mellitus - protein-deficient
123310510000 06112		Mononeuropathy with type 2 diabetes mellitus
123310610000 06114		Mononeuropathy co-occurrent and due to type 2 diabetes mellitus
123312210000 06112		Exudative maculopathy with type 1 diabetes mellitus
123312310000 06110		Exudative maculopathy co-occurrent and due to type 1 diabetes mellitus
123321010000 06118		Cataract due to diabetes mellitus type 2

123321110000 06115		Cataract co-occurrent and due to diabetes mellitus type 2
123322710000 06117		Retinopathy with type 1 diabetes mellitus
123322810000 06119		Retinopathy co-occurrent and due to type 1 diabetes mellitus
123327510000 06115		Mononeuropathy with type 1 diabetes mellitus
123327610000 06118		Mononeuropathy co-occurrent and due to type 1 diabetes mellitus
123341310000 06118		Neurological disorder co-occurrent and due to type 2 diabetes mellitus
123341410000 06111		Neurological disorder with diabetes type 2
123346110000 06115		Neurological disorder with type 1 diabetes mellitus
123346210000 06111		Neurological disorder co-occurrent and due to type 1 diabetes mellitus
123356710000 06113		Exudative maculopathy with type 2 diabetes mellitus
123356810000 06111		Exudative maculopathy co-occurrent and due to type 2 diabetes mellitus
123359810000 06115		Peripheral vascular disorder co-occurrent and due to diabetes mellitus
123359910000 06117		Peripheral vascular disorder due to diabetes mellitus
123361110000 06119		Cataract due to diabetes mellitus type 1
123361210000 06110		Cataract co-occurrent and due to diabetes mellitus type 1
123365310000 06116		Retinopathy with type 2 diabetes mellitus
123365410000 06114		Retinopathy co-occurrent and due to type 2 diabetes mellitus
123368510000 06113		Nervous system disorder due to diabetes mellitus
123368610000 06110		Disorder of nervous system co-occurrent and due to diabetes mellitus
123368710000 06115		Disorder of eye with type 2 diabetes mellitus
123368810000 06117		Disorder of eye co-occurrent and due to type 2 diabetes mellitus
123420410000 06119		Peripheral neuropathy co-occurrent and due to diabetes mellitus
123448010000 06116		Lumbosacral radiculoplexus neuropathy co-occurrent and due to type 2 diabetes mellitus
123448110000 06118		Lumbosacral radiculoplexus neuropathy with type 2 diabetes mellitus
123448210000 06114		Diabetes type II with amyotrophy
123454610000 06119		Lumbosacral radiculoplexus neuropathy with type 1 diabetes mellitus
123454710000 06114		Lumbosacral radiculoplexus neuropathy co-occurrent and due to type 1 diabetes mellitus
123454810000 06112		Lumbosacral radiculoplexus neuropathy with type I diabetes mellitus
123459610000 06112		Ophthalmoplegia with diabetes mellitus

12345971000006117		Ophthalmoplegia co-occurrent and due to diabetes mellitus
12346061000006119		Erectile dysfunction with type 2 diabetes mellitus
12346071000006114		Erectile dysfunction co-occurrent and due to type 2 diabetes mellitus
12363201000006114		Autonomic neuropathy with type 1 diabetes mellitus
12363211000006112		Autonomic neuropathy co-occurrent and due to type 1 diabetes mellitus
12363221000006116		Autonomic neuropathy with type I diabetes mellitus
12363231000006118		Autonomic neuropathy with type 2 diabetes mellitus
12363241000006111		Autonomic neuropathy co-occurrent and due to type 2 diabetes mellitus
12363251000006113		Autonomic neuropathy with type II diabetes mellitus
12363301000006118		Neovascular glaucoma with diabetes mellitus
12363311000006115		Neovascular glaucoma co-occurrent and due to diabetes mellitus
12363661000006113		Gastroparesis with type 1 diabetes mellitus
12363671000006118		Gastroparesis co-occurrent and due to type 1 diabetes mellitus
12363681000006115		Gastroparesis with type 2 diabetes mellitus
12363691000006117		Gastroparesis co-occurrent and due to type 2 diabetes mellitus
12363701000006117		Gastroparesis with diabetes mellitus
12363711000006119		Gastroparesis co-occurrent and due to diabetes mellitus
12363721000006110		Polyneuropathy co-occurrent and due to type 1 diabetes mellitus
12363731000006113		Polyneuropathy co-occurrent and due to type 2 diabetes mellitus
12370271000006116		Disorder of eye with type 1 diabetes mellitus
12370281000006118		Disorder of eye co-occurrent and due to type 1 diabetes mellitus
12370361000006118		Erectile dysfunction with type 1 diabetes mellitus
12370371000006113		Erectile dysfunction co-occurrent and due to type 1 diabetes mellitus
12370381000006111		Peripheral neuropathy with type 2 diabetes
12370391000006114		Peripheral neuropathy co-occurrent and due to type 2 diabetes mellitus
12370401000006111		Nonproliferative retinopathy co-occurrent and due to type 2 diabetes mellitus
12370541000006114		Retinal oedema with type 2 diabetes mellitus
12370551000006111		Retinal oedema co-occurrent and due to type 2 diabetes mellitus
12370561000006113		Retinal edema with type 2 diabetes mellitus

123705710000 06118		Retinal edema co-occurrent and due to type 2 diabetes mellitus
123706010000 06113		Blindness co-occurrent and due to type 2 diabetes mellitus
123706110000 06111		Nonproliferative retinopathy co-occurrent and due to type 1 diabetes mellitus
123706210000 06115		Blindness co-occurrent and due to type 1 diabetes mellitus
123706310000 06117		Peripheral neuropathy with type 1 diabetes mellitus
123706710000 06119		Traction retinal detachment with type 2 diabetes mellitus
123706810000 06116		Traction retinal detachment co-occurrent and due to type 2 diabetes mellitus
123706910000 06118		Rubeosis iridis with type 2 diabetes mellitus
123707010000 06118		Rubeosis iridis co-occurrent and due to type 2 diabetes mellitus
123707110000 06115		Traction retinal detachment with type 1 diabetes mellitus
123707210000 06111		Traction retinal detachment co-occurrent and due to type 1 diabetes mellitus
123707310000 06114		Rubeosis iridis with type 1 diabetes mellitus
123707410000 06116		Rubeosis iridis co-occurrent and due to type 1 diabetes mellitus
123707510000 06119		Cranial nerve palsy with type 2 diabetes mellitus
123707610000 06117		Cranial nerve palsy co-occurrent and due to type 2 diabetes mellitus
123707710000 06112		Cranial nerve palsy with type II diabetes mellitus
123707910000 06113		Retinal ischaemia with type 1 diabetes mellitus
123708010000 06114		Retinal ischemia with type 1 diabetes mellitus
123708110000 06112		Retinal ischaemia co-occurrent and due to type 1 diabetes mellitus
123708210000 06116		Retinal ischemia co-occurrent and due to type 1 diabetes mellitus
123708310000 06118		Retinal ischaemia with type 2 diabetes mellitus
123708410000 06111		Retinal ischaemia co-occurrent and due to type 2 diabetes mellitus
123708510000 06113		Retinal ischemia with type 2 diabetes mellitus
123708610000 06110		Retinal ischemia co-occurrent and due to type 2 diabetes mellitus
123708710000 06115		Retinal oedema with type 1 diabetes mellitus
123708810000 06117		Retinal edema with type 1 diabetes mellitus
123708910000 06119		Retinal oedema co-occurrent and due to type 1 diabetes mellitus
123709010000 06115		Retinal edema co-occurrent and due to type 1 diabetes mellitus
123709310000 06111		Mild nonproliferative retinopathy co-occurrent and due to type 1 diabetes mellitus

123709410000 06118		Moderate nonproliferative retinopathy co-occurrent and due to type 1 diabetes mellitus
123709510000 06116		Mild nonproliferative retinopathy co-occurrent and due to type 2 diabetes mellitus
123709610000 06119		Moderate nonproliferative retinopathy co-occurrent and due to type 2 diabetes mellitus
123711310000 06119		Mild nonproliferative retinopathy co-occurrent and due to secondary diabetes mellitus
123711410000 06112		Nonproliferative retinopathy co-occurrent and due to secondary diabetes mellitus
123711510000 06114		Moderate nonproliferative retinopathy due to secondary diabetes mellitus
123711610000 06111		Moderate nonproliferative retinopathy co-occurrent and due to secondary diabetes mellitus
124507510000 06113		Type 2 diabetes mellitus with gangrene
124507610000 06110		Type I diabetes mellitus with gangrene
124507710000 06115		Type II diabetes mellitus with gangrene
124541410000 06118		Non-insulin-dependent diabetes mellitus with gangrene
124895810000 06110		Type 1 diabetes mellitus with gangrene
124898510000 06117		Type II diabetes mellitus with exudative maculopathy
124915510000 06119		Radiculoplexoneuropathy due to diabetes mellitus
124915710000 06112		Lumbosacral plexopathy co-occurrent and due to diabetes mellitus
124915810000 06110		Lumbosacral plexopathy with diabetes mellitus
125705011		Diabetes mellitus due to insulin receptor antibodies
126169510000 06116		Germline WGS (whole genome sequencing) targeting neonatal diabetes panel
127023610000 06111		Diabetic cataract associated with type 1 diabetes mellitus
127023710000 06116		Mononeuropathy associated with type 1 diabetes mellitus
127023810000 06118		Diabetic oculopathy due to type I diabetes mellitus
127023910000 06115		Diabetic cataract associated with type 2 diabetes mellitus
127024010000 06118		Mononeuropathy associated with type 2 diabetes mellitus
127024110000 06115		Type II diabetes mellitus with neurological complications
127024210000 06111		Type II diabetes mellitus with ophthalmic complications
127039910000 06112		[X]Unspecified diabetes mellitus with renal complications
127041000006 110	C11y0 00	Steroid-induced diabetes
127043910000 06113		Neurologic disorder associated with diabetes mellitus
127044110000 06113		Peripheral circulatory disorder associated with diabetes mellitus

127049510000 06115		Diabetic retinopathy associated with type 1 diabetes mellitus
127049610000 06118		Diabetic oculopathy associated with type 2 diabetes mellitus
127049710000 06113		Neurologic disorder associated with type 2 diabetes mellitus
127049810000 06111		Diabetic retinopathy associated with type 2 diabetes mellitus
127049910000 06114		Ketoacidotic coma in type 2 diabetes mellitus
127050010000 06119		Exudative maculopathy associated with type 1 diabetes mellitus
127050110000 06116		Exudative maculopathy associated with type 2 diabetes mellitus
127050210000 06112		Clinically significant macular oedema of right eye due to diabetes mellitus
127050710000 06113		Gastroparesis due to type 1 diabetes mellitus
127050810000 06111		Gastroparesis due to type 2 diabetes mellitus
127051000006 112	C10B0 00	Steroid-induced diabetes mellitus without complication
127188610000 06118		Diabetes mellitus, adult onset, with no mention of complication
127251910000 06116		Other specified diabetes mellitus with coma
127309710000 06110		Diabetes mellitus with other specified manifestation
127309810000 06113		Diabetes mellitus NOS with other specified manifestation
127623010000 06111		Diabetic oculopathy associated with type 2 diabetes mellitus
137510000061 17	C108y 00	Other specified diabetes mellitus with multiple comps
137610000061 15	C106y 00	Other specified diabetes mellitus with neurological comps
137710000061 10	C105y 00	Other specified diabetes mellitus with ophthalmic complicatn
137810000061 13	C10yy 00	Other specified diabetes mellitus with other spec comps
137910000061 11	C107y 00	Other specified diabetes mellitus with periph circ comps
138110000061 10	C10zy0 0	Other specified diabetes mellitus with unspecified comps
1484867016	F4206 00	Non proliferative diabetic retinopathy
1488898011	C10FK 00	Hyperosmolar non-ketotic state in type 2 diabetes mellitus
155057100000 0114	C10A.1 1	Jamaica type diabetes
15518018	C10N. 00	Secondary diabetes mellitus
166789100000 0113	C10FK 11	Hyperosmolar non-ketotic state in type II diabetes mellitus
166792100000 0117	C10FR 11	Type II diabetes mellitus with gastroparesis
166794100000 0112	C10EQ 11	Type I diabetes mellitus with gastroparesis

1694761000006113		Newly diagnosed diabetes
1697310000006118	2BBF.00	Retinal abnormality - diabetes-related
1703761000000114	8CE0000	Gestational diabetes information leaflet given
1775531012		Hypogonadism, diabetes mellitus, alopecia, mental retardation and electrocardiographic abnormalities
1780311019	C10EL00	Type 1 diabetes mellitus with persistent microalbuminuria
1786715019	C350011	Bronzed diabetes
1786841000006118		Gestational diabetes mellitus annual review
1823921000006119		Diabetes mellitus confirmed
1824641000006115		Advised about diabetes and driving
1850931000006111		Reason for influenza vaccine - diabetes mellitus
189711000000119	C10M000	Lipoatrophic diabetes mellitus without complication
189721000000113	C10N000	Secondary diabetes mellitus without complication
1957911000006111		H/O: hypoglycaemic event in diabetes
1968641000006114	C10Q.00	Maturity-onset diabetes of the young, type 5
197761014	C10F.00	Type 2 diabetes mellitus
197984010	C10E.00	Type 1 diabetes mellitus
1984081000006115		Manchester triage - Diabetes
198461000000116	C10G000	Diabetes mellitus associated with pancreatic disease
1988741000006117		Pregnancy and non-insulin-dependent diabetes mellitus
19931010	66AJ100	Brittle diabetes
20191016	L180900	Gestational diabetes mellitus
205225016	C104.11	Diabetic nephropathy
2128081000000111	66Ay.00	Gestational diabetes mellitus annual review
213141000006111	L180X00	Pre-existing diabetes mellitus, unspecified
214921000006116	F372.00	Polyneuropathy in diabetes
2156000010		Lipodystrophy, partial, with Rieger anomaly, short stature, and insulinopenic diabetes mellitus
2159979014	2BBW.00	Maculopathy of right eye with diabetes mellitus
2159980012	2BBX.00	Maculopathy of left eye with diabetes mellitus
2160090014	C10H.00	Diabetes mellitus induced by non-steroid drugs

216195015	66AU.00	Diabetes care by hospital only
223291000000111	C100111	Maturity onset diabetes
2243331000000115	K27y700	Erectile dysfunction due to diabetes mellitus
2279291000000114	679L211	Advice about diabetes and driving
2288011000000114	C10P000	Type I diabetes mellitus in remission
2288041000000110	C10P011	Type 1 diabetes mellitus in remission
2288061000000111	C10P100	Type II diabetes mellitus in remission
2288071000000116	C10P111	Type 2 diabetes mellitus in remission
2474726011	7276.00	Pan retinal photocoagulation for diabetes
2476117016	C10C.12	Maturity onset diabetes in youth type 1
251591016	1434.00	H/O: diabetes mellitus
2532967014	2BBm.00	Clinically significant macular oedema of right eye due to diabetes mellitus
2567511000006118		Pregestational diabetes mellitus AND/OR impaired glucose tolerance, modified White class F
2575261000006118		Maternal diabetes mellitus with hypoglycaemia affecting foetus OR newborn
2575271000006113		Maternal diabetes mellitus with hypoglycemia affecting fetus OR newborn
2575281000006111		Maternal diabetes mellitus with hypoglycaemia affecting fetus OR newborn
259365016	44V3.00	Glucose tolerance test indicates diabetes mellitus
2622193012	C101.00	Diabetes mellitus with ketoacidosis
264681018	66A4.00	Diabetic on oral treatment
264682013	66A5.00	Diabetic on insulin
264707013	66AI.00	Diabetic - good control
264716012	66AJz00	Diabetic - poor control NOS
264717015	66AK.00	Diabetic - cooperative patient
264718013	66AL.00	Diabetic-uncooperative patient
2649991000006110		Latent diabetes
2650001000006110		Chemical diabetes
2656981000006116		Type 2 diabetes mellitus with acanthosis nigricans
2656991000006118		Insulin-resistant diabetes mellitus AND acanthosis nigricans
2674067015	C10ER00	Latent autoimmune diabetes mellitus in adult

2683561000006119		Brittle diabetes mellitus
2683581000006112		Labile diabetes
2683591000006110		Unstable diabetes mellitus
2686071000006114		Gestational diabetes
2686081000006112		Maternal gestational diabetes mellitus
2686101000006116		GDM - Gestational diabetes mellitus
2722711000006118		Impaired glucose tolerance in and individual with a heritable form of maturity onset diabetes in the young
280511000006113	C109D00	Non-insulin dependent diabetes mellitus with hypoglyca coma
280521000006117	C109A00	Non-insulin dependent diabetes mellitus with mononeuropathy
280531000006119	C109C00	Non-insulin dependent diabetes mellitus with nephropathy
280541000006112	C109B00	Non-insulin dependent diabetes mellitus with polyneuropathy
280551000006114	C109400	Non-insulin-dependent diabetes mellitus with ulcer
280561000006111	C109F00	Non-insulin-dependent diabetes mellitus with peripheral angiopathy
280571000006116	C109.00	Non-insulin dependent diabetes mellitus
280581000006118	C109300	Non-insulin-dependent diabetes mellitus with multiple complications
280591000006115	C109200	Non-insulin-dependent diabetes mellitus with neuro comps
281161000006114	C109E00	Non-insulin depend diabetes mellitus with diabetic cataract
281171000006119	C109700	Type II diabetes mellitus uncontrolled
281181000006116	C109H00	Non-insulin dependent diabetes mellitus with neuropathic arthropathy
281211000006117	C109500	Gangrene associated with type 2 diabetes mellitus
2817479019		Hyperglycaemic crisis in diabetes mellitus
2843291000006110		Maternal diabetes syndrome
2867421000006114		Insulin dependent diabetes mellitus type 1A
2867431000006112		Insulin dependent diabetes mellitus type IA
2900941000006117		Ophthalmic manifestations of diabetes
292466013	C100.00	Diabetes mellitus without complication
292475010	C100z00	Diabetes mellitus NOS with no mention of complication
292478012	C101y00	Other specified diabetes mellitus with ketoacidosis
292479016	C101z00	Diabetes mellitus NOS with ketoacidosis

292480018	C102.00	Diabetes mellitus with hyperosmolar coma
292482014	C102000	Diabetes mellitus, juvenile type, with hyperosmolar coma
292483016	C102100	Diabetes mellitus, adult onset, with hyperosmolar coma
292484010	C102z00	Hyperosmolar coma associated with diabetes mellitus
292488013	C103y00	Coma associated with diabetes mellitus
292489017	C103z00	Diabetes mellitus NOS with ketoacidotic coma
292495016	C104y00	Other specified diabetes mellitus with renal complications
292496015	C104z00	Diabetes mellitus with nephropathy NOS
292503016	C105z00	Diabetes mellitus NOS with ophthalmic manifestation
292512019	C106z00	Diabetes mellitus NOS with neurological manifestation
292523015	C107z00	Diabetes mellitus NOS with peripheral circulatory disorder
292538019	C10E511	Type I diabetes mellitus with ulcer
292540012	C10E500	Type 1 diabetes mellitus with ulcer
292541011	C10E600	Type 1 diabetes mellitus with gangrene
292543014	C10E611	Type I diabetes mellitus with gangrene
292548017	C10E800	Type 1 diabetes mellitus - poor control
292550013	C10E811	Type I diabetes mellitus - poor control
292551012	C10E911	Type I diabetes mellitus maturity onset
292553010	C10E900	Type 1 diabetes mellitus maturity onset
292565014	C108z00	Diabetes mellitus with multiple complications
292576013	C10F311	Type II diabetes mellitus with multiple complications
292577016	C10F300	Type 2 diabetes mellitus with multiple complications
292579018	C10F400	Type 2 diabetes mellitus with ulcer
292581016	C10F411	Type II diabetes mellitus with ulcer
292582011	C10F511	Type II diabetes mellitus with gangrene
292583018	C10F500	Type 2 diabetes mellitus with gangrene
292589019	C10F711	Type II diabetes mellitus - poor control
292590011	C10F700	Type II diabetes mellitus poorly controlled
292606016	C10A100	Malnutrition-related diabetes mellitus with ketoacidosis

292612014	C10A7 00	Malnutrition-related diabetes mellitus without complications
292617015	C10y.0 0	Diabetes mellitus with other specified manifestation
292621010	C10yz0 0	Diabetes mellitus NOS with other specified manifestation
292622015	C10z.0 0	Diabetes mellitus with unspecified complication
292626017	C10zz0 0	Diabetic complication
293756010	Cyu20 00	[X]Other specified diabetes mellitus
293759015	Cyu23 00	Diabetic renal disease
294987100000 6119		Insulin dependent diabetes mellitus type 1B
294988100000 6116		Insulin dependent diabetes mellitus type IB
294989100000 6118		Primary autoimmune diabetes mellitus
2967831017		Pre-existing type 1 diabetes mellitus in pregnancy
297754014	F4202 00	Preproliferative diabetic retinopathy
297755010	F4203 00	Advanced diabetic maculopathy
297758012	F420z0 0	Diabetic retinopathy NOS
299601000000 114	C10EQ 00	Gastroparesis with type 1 diabetes mellitus
299621000000 117	C10FR 00	Gastroparesis with type 2 diabetes mellitus
303846016	K01x1 00	Nephrotic syndrome in diabetes mellitus
303847013	K01x1 11	Kimmelstiel - Wilson disease
303994100000 6118		Pineal hyperplasia AND diabetes mellitus syndrome
303997100000 6114		Pineal hyperplasia, insulin-resistant diabetes mellitus and somatic abnormalities
304180100000 6111		Pregestational diabetes mellitus AND/OR impaired glucose tolerance, modified White class D
306074100000 6116		Pregestational diabetes mellitus AND/OR impaired glucose tolerance, modified White class B
306108017	L1801 00	Diabetes mellitus during pregnancy - baby delivered
306110015	L1803 00	Diabetes mellitus during pregnancy - baby not yet delivered
306112011	L1805 00	Pre-existing diabetes mellitus, insulin-dependent
306113018	L1806 00	Pre-existing diabetes mellitus, non-insulin-dependent
306114012	L1807 00	Pre-existing malnutrition-related diabetes mellitus
308110013	Lyu29 00	Pre-existing diabetes mellitus
310188100000 6116		Prader-Willi syndrome AND diabetes

3112811000006117		Pregestational diabetes mellitus AND/OR impaired glucose tolerance, modified White class C
3115111000006117		Diarrhoea in diabetes
3115121000006113		Diarrhea in diabetes
3192531000006114		Diabetes mellitus associated with receptor abnormality
3209431000006116		Diabetes mellitus type II
3209441000006114		Diabetes mellitus type 2
3220341000006116		Glucoaminophosphate diabetes
3232031000006113		Pituitary diabetes insipidus
3232091000006112		Central diabetes insipidus
3253231000006110		Diabetes mellitus type I
3253241000006117		Diabetes mellitus type 1
3258001000006116		Gestational diabetes mellitus, class A>2<
3291658019		Diabetes mellitus caused by insulin receptor antibodies
3378931000006118		Diabetes-nephrosis syndrome
345486016	F372.12	Diabetic neuropathy
345487013	C106.12	Diabetes mellitus with neuropathy
345492010	F3y0.00	Diabetic mononeuropathy
3459801000006118		Diabetes mellitus associated with hormonal aetiology
3459811000006115		Diabetes mellitus associated with hormonal etiology
347657010	F420400	Diabetic maculopathy
3511581000006114		Pretibial pigmental patches in diabetes
3511973017		Diabetes mellitus education service
3513589015		History of lower extremity amputation as a complication of diabetes mellitus
3513595019		History of diabetes related lower limb amputation
3513596018		History of diabetes related lower extremity amputation
3513751010		Absence of lower extremity due to diabetes mellitus
3513752015		Absence of lower limb due to diabetes mellitus
3514794010		Hyperosmolar hyperglycemic coma due to diabetes mellitus without ketoacidosis
3514795011		Hyperosmolar hyperglycaemic coma due to diabetes mellitus without ketoacidosis
3514797015		Lactic acidosis with diabetes mellitus
3514799017		Lactic acidosis co-occurrent and due to diabetes mellitus
3514801019		Metabolic acidosis with diabetes mellitus
3514803016		Metabolic acidosis co-occurrent and due to diabetes mellitus
3522045015		Hyperosmolarity due to type 1 diabetes mellitus
3526507010		Disorder of nerve co-occurrent and due to type 1 diabetes mellitus

3527174019		Diabetes mellitus caused by chemical
3527175018		Chemical-induced diabetes mellitus
3537388019		Diabetic oculopathy due to type 1 diabetes mellitus
354316011	C314.11	Kidney disorder due to diabetes mellitus
356077018		Malnutrition-related diabetes mellitus - fibrocalculous
356078011		Secondary endocrine diabetes mellitus
356085010	C10D.11	Maturity onset diabetes in youth type 2
356099014		Photomyoclonus, diabetes mellitus, deafness, nephropathy and cerebral dysfunction
356110010		Megaloblastic anaemia, thiamine-responsive, with diabetes mellitus and sensorineural deafness
356111014		Insulin-dependent diabetes mellitus secretory diarrhoea syndrome
356115017		Congenital insulin-dependent diabetes mellitus with fatal secretory diarrhoea
356118015		Diabetes-deafness syndrome maternally transmitted
356119011		Abnormal metabolic state in diabetes mellitus
3636751012		Acute complication with diabetes mellitus
3636752017		Acute complication co-occurrent and due to diabetes mellitus
3649791000006119		Diabetes mellitus AND insipidus with optic atrophy AND deafness
3690681000006114		DM - Diabetes mellitus
3719631000006112		Gestational diabetes mellitus, class A>1<
3730121000006115		Lipodystrophic diabetes with partial lipoatrophy
3748251000006115		Diabetes mellitus in mother complicating pregnancy, childbirth AND/OR puerperium
3770010000006117	Cyu2.00	[X]Diabetes mellitus
3812041000006116		Dwarfism-hepatomegaly-obesity-juvenile diabetes syndrome
3825721000006111		Type 2 diabetes mellitus in obese
3825731000006114		Diabetes mellitus type 2 in obese
3835761000006119		Pregestational diabetes mellitus AND/OR impaired glucose tolerance, modified White class A
3837921000006118		Pregestational diabetes mellitus AND/OR impaired glucose tolerance, modified White class R
3845411000006112		Pregestational diabetes mellitus AND/OR impaired glucose tolerance, modified White class FR
3850111000006112		Anaemia of diabetes
3850121000006116		Anemia of diabetes
3862341000006115		Diabetes mellitus, Addison's disease and myxoedema
3862421000006116		Diabetes mellitus, Addison's disease and myxedema
3966610000006115	Cyu2100	Malnutrition-related diabetes mellitus with multiple complications
3984161000006119		Diabetes mellitus due to structurally abnormal insulin
399419012	F381300	Myasthenic syndrome due to diabetic amyotrophy

401531012	66AJ.00	Diabetic - poor control
411891014	66AJ.11	Unstable diabetes
429970018	C10E411	Unstable type I diabetes mellitus
429971019	C10E400	Unstable type 1 diabetes mellitus
429972014	C10E412	Unstable insulin dependent diabetes mellitus
4313891000006110		Drug-induced nephrogenic diabetes insipidus
4392141000006116		Lipoatrophic diabetes
4392151000006119		Lipodystrophic diabetes
4392181000006110		Renal disorder associated with diabetes mellitus
4392211000006114		Diabetes with peripheral circulatory disorder
4392221000006118		Peripheral angiopathy due to diabetes mellitus
453100011	14F4.00	H/O: Admission in last year for diabetes foot problem
4539551000006112		Pre-existing diabetes mellitus
4539561000006114		History of diabetes mellitus
457325013	C10EA11	Type I diabetes mellitus without complication
457326014	C10EA00	Type 1 diabetes mellitus without complication
457327017	C10EA12	Insulin-dependent diabetes without complication
457328010	C109900	Non-insulin-dependent diabetes mellitus without complication
457329019	C10F900	Type 2 diabetes mellitus without complication
457330012	C10F911	Type II diabetes mellitus without complication
458512016	66AV.00	Diabetic on insulin and oral treatment
459161015	C10EE00	Type 1 diabetes mellitus with hypoglycaemic coma
459162010	C10EE12	Insulin dependent diabetes mellitus with hypoglycaemic coma
459163017	C10EE11	Type I diabetes mellitus with hypoglycaemic coma
459167016	C10FD00	Type 2 diabetes mellitus with hypoglycaemic coma
459169018	C10FD11	Hypoglycaemic coma co-occurrent and due to diabetes mellitus type II
459292011	C10EH00	Type 1 diabetes mellitus with arthropathy
459293018	C10EH12	Insulin dependent diabetes mellitus with arthropathy
459294012	C10EH11	Type I diabetes mellitus with arthropathy

459295013	C10EJ11	Type I diabetes mellitus with neuropathic arthropathy
459296014	C10EJ00	Type 1 diabetes mellitus with neuropathic arthropathy
459306016	C10FF11	Type II diabetes mellitus with peripheral angiopathy
459308015	C10FF00	Type 2 diabetes mellitus with peripheral angiopathy
459309011	C109G00	Non-insulin dependent diabetes mellitus with arthropathy
459310018	C10FG00	Type 2 diabetes mellitus with arthropathy
459311019	C10FG11	Type II diabetes mellitus with arthropathy
459312014	C10FH11	Type II diabetes mellitus with neuropathic arthropathy
459313016	C10FH00	Type 2 diabetes mellitus with neuropathic arthropathy
4600881000006113		Glucose tolerance test indicates diabetes mellitus
4757991000006116		Type 1 diabetes mellitus with hyperosmolar coma
4758011000006112		Type 2 diabetes mellitus with hyperosmolar coma
4758061000006110		Insulin-dependent diabetes mellitus with ulcer
4758081000006117		Insulin-dependent diabetes mellitus with gangrene
4758121000006115		Insulin-dependent diabetes maturity onset
4788421000006117		Nephrotic syndrome due to diabetes mellitus
4796311000006117		Pre-existing type 1 diabetes mellitus
4796321000006113		Pre-existing type 2 diabetes mellitus
483882011	C10C.00	Maturity-onset diabetes of the young
483886014	C10C.11	Maturity onset diabetes in youth
493773010	C109.11	NIDDM - Non-insulin dependent diabetes mellitus
493774016	C10F.11	Type II diabetes mellitus
494564012	C10E.11	Type I diabetes mellitus
494831000000119	C10N100	Diabetes mellitus associated with cystic fibrosis
498512013		Genetic syndromes of diabetes mellitus
5109461000006115		Diabetes type 2 on insulin
5109551000006116		Transitory neonatal diabetes mellitus
5109561000006119		Maturity onset diabetes of the young, type 2
5109581000006112		Maturity onset diabetes in youth type II

510959100000 6110		MODY - Maturity onset diabetes in youth type II
510961100000 6116		MODY - Maturity onset diabetes in youth type 2
510962100000 6112		MODY - Maturity onset diabetes glucokinase-related
510967100000 6113		Insulin receptor defect with insulin-resistant diabetes mellitus and acanthosis nigricans
510970100000 6114		Muscular atrophy, ataxia, retinitis pigmentosa, and diabetes mellitus
510983100000 6114		Congenital insulin-dependent diabetes mellitus with fatal secretory diarrhea
510985100000 6119		Insulin-dependent diabetes mellitus secretory diarrhea syndrome
511000100000 6118		Pregnancy and type 2 diabetes mellitus
511007100000 6112		Hypoglycaemic event in diabetes
511008100000 6110		Hypoglycemic event in diabetes
511009100000 6113		Hypoglycaemic state in diabetes
511010100000 6119		Hypoglycemic state in diabetes
511045100000 6112		Insulin resistance in diabetes
512873100000 6118		Soft tissue complication of diabetes mellitus
512925100000 6110		Hypohidrosis-diabetes insipidus syndrome
533750100000 6114		Epiphyseal dysplasia, multiple, with early onset diabetes mellitus
550565100000 6113		Diabetes mellitus uncontrolled
558707100000 6119		Diabetes mellitus in neonate small for gestational age
568775100000 6119		Lipoatrophic diabetes mellitus
575458100000 6110		Brittle type 1 diabetes mellitus
575461100000 6119		Brittle type I diabetes mellitus
575462100000 6110		Labile type I diabetes mellitus
587111000006 111	C1091 00	Non-insulin-dependent diabetes mellitus with ophthalm comps
587521000006 111	C1090 00	Non-insulin-dependent diabetes mellitus with renal comps
591434100000 6112		Postpancreatectomy diabetes mellitus
599099100000 6114		History of admission in last year for diabetes foot problem
603000100000 6116		Insulin-dependent diabetes without complication
605004100000 6115		Insulin dependent diabetes mellitus with hypoglycaemic coma
605006100000 6116		Type I diabetes mellitus with hypoglycemic coma

605007100000 6111		Insulin dependent diabetes mellitus with hypoglycemic coma
605008100000 6114		Type 1 diabetes mellitus with hypoglycemic coma
605117100000 6117		Insulin dependent diabetes mellitus with arthropathy
616081000006 113	889A.0 0	Diabetes mellitus insulin-glucose infusion in acute myocardial infarction
616151000006 118	L1800 00	Diabetes mellitus - unspec whether in pregnancy/puerperium
616191000006 112	L180.0 0	Diabetes mellitus during pregnancy, childbirth and the puerperium
616211000006 113	L180z0 0	Diabetes mellitus in pregnancy/childbirth/puerperium NOS
616221000006 117	L1804 00	Diabetes mellitus in the puerperium - baby delivered during previous episode of care
616231000006 119	L1802 00	Diabetes mellitus in the puerperium - baby delivered during current episode of care
616241000006 112	C10B.0 0	Diabetes mellitus induced by steroids
616351000006 115	C107.1 1	Diabetes mellitus with gangrene
616381000006 111	C103.0 0	Diabetes mellitus with ketoacidotic coma
616391000006 114	C106.0 0	Nervous system disorder due to diabetes mellitus
616421000006 118	C105.0 0	Eye disorder due to diabetes mellitus
616441000006 113	C107.0 0	Peripheral vascular disorder due to diabetes mellitus
616451000006 110	C106.1 3	Diabetes mellitus with polyneuropathy
616461000006 112	C104.0 0	Diabetes mellitus with renal manifestation
616481000006 119	C1061 00	Diabetes mellitus, adult onset, + neurological manifestation
616491000006 116	C1051 00	Diabetes mellitus, adult onset, + ophthalmic manifestation
616501000006 112	C10z1 00	Disorder due to type 2 diabetes mellitus
616511000006 110	C1001 00	Diabetes mellitus, adult onset, no mention of complication
616531000006 116	C1011 00	Diabetes mellitus, adult onset, with ketoacidosis
616541000006 114	C1031 00	Diabetes mellitus, adult onset, with ketoacidotic coma
616551000006 111	C1041 00	Diabetes mellitus, adult onset, with renal manifestation
616561000006 113	C1072 00	Diabetes mellitus, adult with gangrene
616571000006 118	C10y1 00	Diabetes mellitus, adult, + other specified manifestation
616581000006 115	C1071 00	Diabetes mellitus, adult, + peripheral circulatory disorder
616591000006 117	C1070 00	Diabetes mellitus, juvenile +peripheral circulatory disorder
616601000006 113	C1050 00	Diabetes mellitus, juvenile type, + ophthalmic manifestation

616611000006111	C10z000	Disorder due to type 1 diabetes mellitus
616621000006115	C100000	Type 1 diabetes mellitus without complication
616641000006110	C101000	Diabetes mellitus, juvenile type, with ketoacidosis
616651000006112	C103000	Diabetes mellitus, juvenile type, with ketoacidotic coma
616661000006114	C104000	Diabetes mellitus, juvenile type, with renal manifestation
616671000006119	C106000	Diabetes mellitus, juvenile, + neurological manifestation
616681000006116	C10y000	Diabetes mellitus, juvenile, + other specified manifestation
616831000006118	C107.12	Diabetes with gangrene
616921000006113	C106.11	Diabetic amyotrophy
6204741000006112		Type 2 diabetes mellitus in nonobese
6204751000006114		Diabetes mellitus type 2 in nonobese
622051000000118	ZV13F00	History of gestational diabetes mellitus
622221000000118	C10FS00	Maternally inherited diabetes mellitus
641581000006115	C109600	Non-insulin-dependent diabetes mellitus with retinopathy
6618061000006116		Bronze diabetes
674961000006118	C107400	Peripheral circulatory disorder associated with type 2 diabetes mellitus
6763751000006119		Diabetes mellitus caused by non-steroid drugs
6837961000006116		Panretinal photocoagulation for diabetes
6837991000006112		Diabetes mellitus caused by non-steroid drugs without complication
6931861000006110		Infection of foot associated with diabetes
6951081000006112		Ketoacidosis in type I diabetes mellitus
6951091000006110		Diabetes type 1 with ketoacidosis
6951281000006117		Kidney disorder associated with type 2 diabetes mellitus
6953871000006115		Ketoacidosis in diabetes mellitus
6954121000006112		Mononeuropathy associated with type II diabetes mellitus
6954991000006112		Exudative maculopathy associated with type I diabetes mellitus
6955501000006113		Persistent proteinuria associated with type I diabetes mellitus
6958521000006110		Neurological disorder associated with malnutrition-related diabetes mellitus
6959091000006114		Persistent microalbuminuria associated with type II diabetes mellitus

695980100000 6112		Diabetic cataract associated with type II diabetes mellitus
695981100000 6110		Diabetes type 2 with cataract
696043100000 6119		Diabetic retinopathy associated with type I diabetes mellitus
696044100000 6112		Diabetes type 1 with retinopathy
696108100000 6118		Gangrene associated with type 1 diabetes mellitus
696109100000 6115		Gangrene associated with type I diabetes mellitus
696110100000 6114		Diabetes type 1 with gangrene
696190100000 6111		Disorder associated with type I diabetes mellitus
696275100000 6117		Mononeuropathy associated with type I diabetes mellitus
696425100000 6119		Coma associated with malnutrition-related diabetes mellitus
696556100000 6116		Ketoacidotic coma in type I diabetes mellitus
696557100000 6111		Diabetes mellitus type 1 with ketoacidotic coma
696709100000 6111		Diabetic oculopathy associated with type 1 diabetes mellitus
696864100000 6111		Ophthalmic complication of malnutrition-related diabetes mellitus
696952100000 6113		Persistent microalbuminuria associated with type 1 diabetes mellitus
696953100000 6111		Persistent microalbuminuria associated with type I diabetes mellitus
696992100000 6119		Neurologic disorder associated with type II diabetes mellitus
696993100000 6116		Diabetes type 2 with neurological disorder
697062100000 6114		Peripheral circulatory disorder associated with type 1 diabetes mellitus
697063100000 6112		Peripheral circulatory disorder associated with type I diabetes mellitus
697187100000 6117		Hypoglycaemic coma in type 1 diabetes mellitus
697188100000 6119		Hypoglycemic coma in type I diabetes mellitus
697189100000 6116		Hypoglycaemic coma in type I diabetes mellitus
697190100000 6117		Hypoglycemic coma in type 1 diabetes mellitus
697191100000 6119		Diabetes type 1 with hypoglycemic coma
697192100000 6110		Diabetes type 1 with hypoglycaemic coma
697244100000 6117		Neurological disorder associated with type I diabetes mellitus
697245100000 6115		Diabetes type 1 with neurological disorder
697521100000 6112		Gangrene associated with type 2 diabetes mellitus

6975221000006116		Gangrene associated with type II diabetes mellitus
6976921000006118		Hypoglycaemic coma in diabetes mellitus
6976931000006115		Hypoglycemic coma in diabetes mellitus
6977421000006110		Ketoacidosis in type II diabetes mellitus
6977431000006113		Diabetes type 2 with ketoacidosis
6977971000006119		Exudative maculopathy associated with type II diabetes mellitus
6979251000006110		Diabetes mellitus type 2 with ketoacidotic coma
6979991000006111		Renal disorder associated with type I diabetes mellitus
6980001000006117		Kidney disorder associated with type 1 diabetes mellitus
6980431000006116		Diabetic cataract associated with type I diabetes mellitus
6980441000006114		Diabetes type 1 with cataract
6981271000006112		Non-ketotic non-hyperosmolar coma associated with diabetes mellitus
6981621000006113		Persistent proteinuria associated with type II diabetes mellitus
6982151000006118		Disorder due to type II diabetes mellitus
6982511000006110		Diabetic retinopathy associated with type II diabetes mellitus
6982521000006119		Diabetes type 2 with retinopathy
6983511000006117		Neurologic complication of diabetes mellitus
6983691000006114		Diabetic oculopathy associated with type II diabetes mellitus
6985001000006110		Peripheral circulatory disorder associated with type 2 diabetes mellitus
6985011000006113		Peripheral circulatory disorder associated with type II diabetes mellitus
6985421000006113		Skin ulcer associated with diabetes mellitus
6986151000006117		Multiple complications of type I diabetes mellitus
6987011000006115		Gangrene associated with diabetes mellitus
7063141000006118		Diabetes mellitus co-occurrent and due to cystic fibrosis
7065531000006115		Latent autoimmune diabetes mellitus in adult (LADA)
7066041000006112		Small vessel disease due to type 1 diabetes mellitus
7066051000006114		Diabetes type 1 with small vessel disease
7067891000006114		Amyotrophy due to type 2 diabetes mellitus
7067901000006113		Diabetes type 2 with amyotrophy

706883100000 6114		Diabetes mellitus due to cystic fibrosis
706948100000 6115		Small vessel disease due to type 2 diabetes mellitus
707589100000 6110		Amyotrophy due to type 1 diabetes mellitus
708202100000 6119		Erectile dysfunction associated with type 2 diabetes mellitus
709525100000 6110		Hyperosmolality due to uncontrolled type 1 diabetes mellitus
719471000006 119	C10A W00	Malnutrit-related diabetes mellitus with unspec complics
719481000006 116	C10A3 00	Malnutrit-related diabetes mellitus wth ophthalmic complicat
719531000006 118	C10A.0 0	Malnutrition related diabetes mellitus
719541000006 111	C10A0 00	Malnutrition-related diabetes mellitus with coma
719561000006 110	C10A6 00	Malnutrition-related diabetes mellitus with multiple comps
719571000006 115	C10A2 00	Malnutrition-related diabetes mellitus with renal complications
719591000006 119	C10A4 00	Malnutrition-related diabetes mellitus wth neuro complicatns
719601000006 110	C10A5 00	Malnutrition-related diabetes mellitus with peripheral circulatory complications
720311000006 119	C10AX 00	Malnutrit-relat diabetes mellitus with other spec comps
725281100000 6115		Multiple complications due to diabetes mellitus
725322100000 6117		Hyperglycemic crisis in diabetes mellitus
726510000061 14	C1084 00	Unstable insulin dependent diabetes mellitus
727110000061 17	C1084 12	Unstable type 1 diabetes mellitus
727210000061 13	C1084 11	Unstable type I diabetes mellitus
728151100000 6118		Type 2 diabetes mellitus uncontrolled
728761100000 6113		Type 1 diabetes mellitus uncontrolled
728762100000 6117		Type I diabetes mellitus uncontrolled
728764100000 6112		Type 1 diabetes mellitus well controlled
728765100000 6114		Type I diabetes mellitus well controlled
728819100000 6110		Type 2 diabetes mellitus well controlled
728820100000 6113		Type II diabetes mellitus well controlled
730532100000 6114		Posttransplant diabetes mellitus
730675100000 6110		Brittle type 2 diabetes mellitus
730676100000 6112		Brittle type II diabetes mellitus

7306771000006117		Labile type II diabetes mellitus
7306781000006119		Unstable type II diabetes mellitus
73466011	C100112	Non-insulin dependent diabetes mellitus
7467910000006111	C108A00	Insulin-dependent diabetes without complication
7476191000006118		Gestational diabetes mellitus uncontrolled
7480661000006114		H/O diabetes mellitus type 2
7480671000006119		History of diabetes mellitus type 2
7480681000006116		History of diabetes mellitus type II
7480691000006118		H/O diabetes mellitus type 1
7480701000006118		History of diabetes mellitus type I
7480711000006115		History of diabetes mellitus type 1
7480721000006111		H/O gestational diabetes mellitus
7480741000006116		History of maturity onset diabetes mellitus in young
7480751000006119		History of autosomal dominant diabetes mellitus
7484151000006111		On subcutaneous insulin for diabetes mellitus
7500191000006118		Maturity onset diabetes of the young, type 1
7500211000006117		Pre-existing diabetes mellitus in pregnancy
7500221000006113		Pre-existing type 1 diabetes mellitus in pregnancy
7500231000006111		Permanent neonatal diabetes mellitus
7500241000006118		Permanent diabetes mellitus of infancy
7500251000006116		Pregnancy and type 1 diabetes mellitus
7500261000006119		Pre-existing type 2 diabetes mellitus in pregnancy
7500271000006114		Diabetes mellitus due to genetic defect in beta cell function
7500281000006112		Diabetes mellitus due to genetic defect in insulin action
7500291000006110		Maturity-onset diabetes of the young, type 3
7500311000006114		Maturity-onset diabetes of the young, type 4
7500341000006113		MODY5 - maturity-onset diabetes of the young type 5
7500351000006110		Renal cysts and diabetes syndrome
7500361000006112		RCAD (renal cysts and diabetes) syndrome

750037100000 6117		Maturity-onset diabetes of the young, type 6
750039100000 6116		Maturity-onset diabetes of the young, type 7
750041100000 6116		Maturity-onset diabetes of the young, type 8
750043100000 6110		Diabetes-pancreatic exocrine dysfunction syndrome
750044100000 6117		Maturity-onset diabetes of the young, type 9
750046100000 6118		Maturity-onset diabetes of the young, type 10
750048100000 6111		Maturity-onset diabetes of the young, type 11
756585100000 6114		Supervision of high risk pregnancy with history of gestational diabetes mellitus
758820100000 6117		Fetal hypertrophic cardiomyopathy due to maternal diabetes mellitus
758821100000 6119		Foetal hypertrophic cardiomyopathy due to maternal diabetes mellitus
760984100000 6119		Neuropathy due to unstable diabetes mellitus type 1
760985100000 6117		Neuropathy due to brittle type I diabetes mellitus
760988100000 6113		Retinopathy due to unstable diabetes mellitus type 1
760989100000 6111		Retinopathy due to brittle type I diabetes mellitus
764191000006 112	2G510 00	Foot abnormality - diabetes related
764201000006 110	2G5C. 00	Foot abnormality - diabetes related
764468100000 6117		Gingivitis co-occurrent with diabetes mellitus
769679100000 6110		Diabetic autonomic neuropathy due to type 1 diabetes mellitus
769680100000 6111		Diabetic autonomic neuropathy due to type 2 diabetes mellitus
770435100000 6117		Neovascular glaucoma due to diabetes mellitus
770805100000 6112		Gastroparesis due to diabetes mellitus type I
770807100000 6119		Gastroparesis due to diabetes mellitus type II
770808100000 6116		Gastroparesis due to diabetes mellitus
770809100000 6118		Polyneuropathy due to type 1 diabetes mellitus
770812100000 6119		Polyneuropathy due to diabetes mellitus type II
771331000006 116	C108H 00	Insulin dependent diabetes mellitus with arthropathy
771341000006 114	C108F 00	Insulin dependent diabetes mellitus with diabetic cataract
771351000006 111	C1086 00	Insulin dependent diabetes mellitus with gangrene
771361000006 113	C108E 00	Insulin dependent diabetes mellitus with hypoglycaemic coma

771371000006118	C108B00	Insulin dependent diabetes mellitus with mononeuropathy
771381000006115	C108300	Insulin dependent diabetes mellitus with multiple complicatn
771391000006117	C108D00	Insulin dependent diabetes mellitus with nephropathy
771401000006115	C108C00	Insulin dependent diabetes mellitus with polyneuropathy
771411000006117	C108700	Insulin dependent diabetes mellitus with retinopathy
771421000006113	C108500	Insulin dependent diabetes mellitus with ulcer
771481000006112	C108200	Insulin-dependent diabetes mellitus with neurological comps
771491000006110	C108100	Insulin-dependent diabetes mellitus with ophthalmic comps
771501000006119	C108000	Insulin-dependent diabetes mellitus with renal complications
772151000006116	C108900	Insulin dependent diabetes maturity onset
772161000006119	C100011	Insulin dependent diabetes mellitus
772171000006114	C108.00	Insulin dependent diabetes mellitus
772181000006112	C108800	Insulin dependent diabetes mellitus - poor control
7746161000006117		Gingival disease co-occurrent with diabetes mellitus
77727018	C10E.12	Insulin dependent diabetes mellitus
7783451000006114		Hypoglycaemic coma co-occurrent and due to diabetes mellitus type II
7814941000006115		Acidosis due to type 1 diabetes mellitus
7814951000006118		Acidosis due to type 2 diabetes mellitus
7828581000006116		Pancreatic hypoplasia, diabetes mellitus, congenital heart disease syndrome
7831891000006110		Intellectual disability, craniofacial dysmorphism, hypogonadism, diabetes mellitus syndrome
7839901000006110		Renal papillary necrosis due to diabetes mellitus
7851031000006118		Proximal tubulopathy, diabetes mellitus, cerebellar ataxia syndrome
7851131000006117		Permanent neonatal diabetes mellitus with cerebellar agenesis syndrome
7851551000006119		Neonatal diabetes, congenital hypothyroidism, congenital glaucoma, hepatic fibrosis, polycystic kidney syndrome
7861571000006116		Radiculoplexoneuropathy due to diabetes mellitus
7862571000006113		Lesion of skin co-occurrent and due to diabetes mellitus
7863801000006113		Lumbosacral plexopathy co-occurrent and due to diabetes mellitus
7863811000006111		Lumbosacral plexopathy with diabetes mellitus
787101000006114	C107300	IDDM with peripheral circulatory disorder

787111000006112	C108.11	IDDM-Insulin dependent diabetes mellitus
7965021000006116		Disorder associated with well controlled type 2 diabetes mellitus
7966541000006112		Diabetic erectile dysfunction associated with type 1 diabetes mellitus
7966551000006114		Erectile dysfunction associated with type I diabetes mellitus
7966561000006111		Mixed hyperlipidaemia due to type 2 diabetes mellitus
7966571000006116		Mixed hyperlipidaemia associated with type II diabetes mellitus
7966581000006118		Mixed hyperlipidemia associated with type II diabetes mellitus
7966591000006115		Mixed hyperlipidemia due to type 2 diabetes mellitus
7966601000006111		Mixed hyperlipidemia due to type II diabetes mellitus
7966611000006114		Chronic kidney disease stage 5 due to type 2 diabetes mellitus
7966621000006118		Diabetic stage 5 chronic renal impairment due to type 2 diabetes mellitus
7966631000006115		Chronic kidney disease stage 5 due to type II diabetes mellitus
7966641000006113		Chronic kidney disease stage 4 due to type 2 diabetes mellitus
7966651000006110		Diabetic stage 4 chronic renal impairment due to type 2 diabetes mellitus
7966661000006112		Chronic kidney disease stage 4 due to type II diabetes mellitus
7966691000006116		Chronic kidney disease stage 3 due to type 2 diabetes mellitus
7966701000006116		Diabetic stage 3 chronic renal impairment due to type 2 diabetes mellitus
7966711000006118		Chronic kidney disease stage 3 due to type II diabetes mellitus
7966761000006115		Chronic kidney disease stage 2 due to type 2 diabetes mellitus
7966771000006110		Diabetic stage 2 chronic renal impairment due to type 2 diabetes mellitus
7966781000006113		Chronic kidney disease stage 2 due to type II diabetes mellitus
7966791000006111		Chronic kidney disease stage 1 due to type 2 diabetes mellitus
7966801000006112		Diabetic stage 1 chronic renal impairment due to type 2 diabetes mellitus
7966811000006110		Chronic kidney disease stage 1 due to type II diabetes mellitus
7966831000006116		Diabetic dyslipidemia associated with type 2 diabetes mellitus
7966841000006114		Dyslipidemia associated with type II diabetes mellitus
7966871000006118		Chronic kidney disease due to type 2 diabetes mellitus
7966881000006115		Chronic renal impairment due to type II diabetes mellitus
7966891000006117		Diabetic chronic renal impairment due to type 2 diabetes mellitus

7966901000006118		Chronic renal impairment due to type 2 diabetes mellitus
7966911000006115		Diabetic neuropathic arthropathy associated with type 2 diabetes mellitus
7966921000006111		Diabetic Charcot's arthropathy associated with type 2 diabetes mellitus
7966931000006114		Angina associated with type 2 diabetes mellitus
7966941000006116		Angina associated with type II diabetes mellitus
7966951000006119		Diabetic angina pectoris associated with type 2 diabetes mellitus
7968751000006116		Diabetes mellitus type 2 without retinopathy
7968811000006114		Diabetic vitreous haemorrhage associated with type 2 diabetes mellitus
7968821000006118		Diabetic vitreous haemorrhage associated with type II diabetes mellitus
7968831000006115		Diabetic vitreous hemorrhage associated with type II diabetes mellitus
7968841000006113		Diabetic vitreous hemorrhage associated with type 2 diabetes mellitus
7968881000006119		Proliferative diabetic retinopathy due to type 2 diabetes mellitus
7968891000006116		Proliferative diabetic retinopathy due to type II diabetes mellitus
7968941000006115		Diabetic peripheral neuropathy associated with type 2 diabetes mellitus
7968951000006118		Diabetic peripheral neuropathy associated with type II diabetes mellitus
7969001000006112		Foot ulcer due to type 2 diabetes mellitus
7969011000006110		Diabetic foot ulcer associated with type II diabetes mellitus
7969021000006119		Foot ulcer due to type II diabetes mellitus
7969051000006111		Diabetic dermopathy associated with diabetes mellitus type 2
7969151000006110		Nonproliferative diabetic retinopathy due to type 2 diabetes mellitus
7969161000006112		Nonproliferative diabetic retinopathy due to type II diabetes mellitus
7969211000006115		Mixed hyperlipidaemia due to type 1 diabetes mellitus
7969221000006111		Mixed hyperlipidemia due to type 1 diabetes mellitus
7997131000006112		Complication due to diabetes mellitus type 2
8006221000006115		Microalbuminuria due to type 1 diabetes mellitus
8012071000006112		Type II diabetes on insulin
8012091000006113		Type II diabetes on diet only
8014011000006114		Retinal oedema due to type 2 diabetes mellitus
8014021000006118		Retinal edema due to type 2 diabetes mellitus

8014491000006111		Peripheral vascular disease due to type I diabetes
8014501000006115		Diabetes mellitus type 1 without retinopathy
8016601000006114		Postpartum gestational diabetes mellitus
8016611000006112		Gestational diabetes, delivered
8016621000006116		Gestational diabetes mellitus complicating pregnancy
8016791000006114		Glaucoma due to type 2 diabetes mellitus
8019010000006119	L180811	Gestational diabetes mellitus
8019321000006119		History of small vessel disease due to diabetes mellitus
8019511000006116		Blindness due to type 2 diabetes mellitus
8019521000006112		Nonproliferative diabetic retinopathy due to type 1 diabetes mellitus
8019531000006110		Proliferative retinopathy due to type 1 diabetes mellitus
8019541000006117		Blindness due to type 1 diabetes mellitus
8022361000006113		Hypertension in chronic kidney disease due to type 2 diabetes mellitus
8022371000006118		Hypertension in chronic kidney disease due to type II diabetes mellitus
8022381000006115		Nephrotic syndrome due to type 2 diabetes mellitus
8022401000006115		Hypertension in chronic kidney disease due to type 1 diabetes mellitus
8022411000006117		Hypertension in chronic kidney disease due to type I diabetes mellitus
8022431000006111		Nephrotic syndrome due to type 1 diabetes mellitus
8022441000006118		Neuropathic arthropathy due to type 1 diabetes mellitus
8022461000006119		Diabetic neuropathic arthropathy due to type 1 diabetes mellitus
8022471000006114		Diabetes mellitus type 1 with diabetic Charcot's arthropathy
8022481000006112		Peripheral neuropathy due to type 1 diabetes mellitus
8022491000006110		Peripheral neuropathy co-occurrent and due to type 1 diabetes mellitus
8022541000006117		Diabetic dermopathy due to type 1 diabetes mellitus
8022551000006115		Severe malnutrition due to type 1 diabetes mellitus
8022561000006118		Osteomyelitis due to type 1 diabetes mellitus
8022571000006113		Severe malnutrition due to type 2 diabetes mellitus
8022581000006111		Osteomyelitis due to type 2 diabetes mellitus
8022601000006118		Chronic ulcer of skin due to type 1 diabetes mellitus

8024741000006117		Traction retinal detachment due to type 2 diabetes mellitus
8024751000006115		Rubeosis iridis due to type 2 diabetes mellitus
8024761000006118		Traction retinal detachment due to type 1 diabetes mellitus
8024771000006113		Rubeosis iridis due to type 1 diabetes mellitus
8025151000006118		Insulin reactive hypoglycaemia in type 2 diabetes mellitus
8025161000006116		Insulin reactive hypoglycemia in type 2 diabetes mellitus
8025171000006111		Hypoglycaemia due to type 1 diabetes mellitus
8025181000006114		Hypoglycemia due to type 1 diabetes mellitus
8025481000006119		Supervision of high risk pregnancy with history of gestational diabetes mellitus done
8026021000006118		Ankle ulcer due to type 2 diabetes mellitus
8026031000006115		Heel AND/OR midfoot ulcer due to type 2 diabetes mellitus
8026041000006113		Forefoot ulcer due to type 2 diabetes mellitus
8026051000006110		Ankle ulcer due to type 1 diabetes mellitus
8026061000006112		Heel AND/OR midfoot ulcer due to type 1 diabetes mellitus
8026071000006117		Forefoot ulcer due to type 1 diabetes mellitus
8026181000006118		Cranial nerve palsy due to type 2 diabetes mellitus
8027391000006115		Chronic kidney disease stage 1 due to type 1 diabetes mellitus
8027401000006118		Chronic kidney disease stage 1 due to type I diabetes mellitus
8027411000006115		Chronic kidney disease stage 2 due to type 1 diabetes mellitus
8027421000006111		Chronic kidney disease stage 2 due to type I diabetes mellitus
8027451000006119		Chronic kidney disease stage 3 due to type 1 diabetes mellitus
8027461000006117		Chronic kidney disease stage 3 due to type I diabetes mellitus
8027471000006112		Chronic kidney disease stage 4 due to type 1 diabetes mellitus
8027481000006110		Chronic kidney disease stage 4 due to type I diabetes mellitus
8027491000006113		Chronic kidney disease stage 5 due to type 1 diabetes mellitus
8027501000006117		Chronic kidney disease stage 5 due to type I diabetes mellitus
8027511000006119		End stage renal disease on dialysis due to type 1 diabetes mellitus
8027521000006110		Microalbuminuria due to type 2 diabetes mellitus
8027531000006113		End stage renal disease on dialysis due to type 2 diabetes mellitus

8030331000006117		Chronic kidney disease due to type 1 diabetes mellitus
8030341000006110		Chronic kidney disease due to type I diabetes mellitus
8030601000006119		Macular oedema and retinopathy due to type 2 diabetes mellitus
8030611000006116		Macular edema and retinopathy due to type 2 diabetes mellitus
8030621000006112		Proliferative retinopathy with retinal oedema due to type 2 diabetes mellitus
8030631000006110		Proliferative retinopathy with retinal edema due to type 2 diabetes mellitus
8032031000006116		Skin ulcer due to type 2 diabetes mellitus
8032041000006114		Ulcer of skin co-occurrent and due to type II diabetes mellitus
8032051000006111		Ulcer of skin co-occurrent and due to type 2 diabetes mellitus
8032081000006115		Sensory neuropathy due to type 1 diabetes mellitus
8032731000006119		Retinal ischaemia due to type 1 diabetes mellitus
8032741000006112		Retinal ischemia due to type 1 diabetes mellitus
8032751000006114		Diabetic vitreous haemorrhage due to type 1 diabetes mellitus
8032761000006111		Vitreous haemorrhage due to type 1 diabetes mellitus
8032771000006116		Vitreous hemorrhage due to type 1 diabetes mellitus
8032781000006118		Diabetic vitreous hemorrhage due to type 1 diabetes mellitus
8032791000006115		Retinal ischaemia due to type 2 diabetes mellitus
8032801000006119		Retinal ischemia due to type 2 diabetes mellitus
8032891000006114		Diabetes mellitus due to pancreatic injury
8033291000006116		Pre-existing diabetes mellitus in mother complicating childbirth
8033301000006115		Pre-existing diabetes mellitus in childbirth
8034251000006112		Neuropathic ulcer of midfoot AND/OR heel due to type 2 diabetes mellitus
8034261000006114		Suspected glaucoma due to type 2 diabetes mellitus
8034431000006118		Retinal oedema due to type 1 diabetes mellitus
8034441000006111		Retinal edema due to type 1 diabetes mellitus
8034841000006114		Ulcer of lower limb due to type 1 diabetes mellitus
8034851000006111		Ulcer of lower extremity due to type 2 diabetes mellitus
8034861000006113		Leg ulcer due to type 2 diabetes mellitus
8034871000006118		Ulcer of lower extremity due to diabetes mellitus type 2

803488100000 6115		Peripheral sensory neuropathy due to type 2 diabetes mellitus
803521100000 6110		Dyslipidemia with high density lipoprotein below reference range and triglyceride above reference range due to type 2 diabetes mellitus
803754100000 6117		Hypoglycaemia unawareness in type 2 diabetes mellitus
803755100000 6115		Hypoglycemia unawareness in type 2 diabetes mellitus
803782100000 6110		Hypoglycaemic unawareness in type 1 diabetes mellitus
803783100000 6113		Hypoglycemic unawareness in type 1 diabetes mellitus
803784100000 6115		Hypoglycaemia due to type 2 diabetes mellitus
803785100000 6118		Hypoglycemia due to type 2 diabetes mellitus
804033100000 6118		Hypertension concurrent and due to end stage renal disease on dialysis due to type 2 diabetes mellitus
804034100000 6111		Hypertension concurrent and due to end stage renal disease on dialysis due to type 1 diabetes mellitus
804330100000 6116		Hyperlipidaemia due to type 2 diabetes mellitus
804331100000 6118		Hyperlipidemia due to type 2 diabetes mellitus
804332100000 6114		Hyperlipidaemia due to type 1 diabetes mellitus
804333100000 6112		Hyperlipidemia due to type 1 diabetes mellitus
804359100000 6119		Mild nonproliferative retinopathy due to type 1 diabetes mellitus
804360100000 6110		Moderate nonproliferative retinopathy due to type 1 diabetes mellitus
804362100000 6117		Mild nonproliferative retinopathy due to type 2 diabetes mellitus
804363100000 6119		Moderate nonproliferative retinopathy due to type 2 diabetes mellitus
804401100000 6117		Hypertension in chronic kidney disease stage 5 due to type 2 diabetes mellitus
804402100000 6113		Hypertension in chronic kidney disease stage 5 due to type II diabetes mellitus
804404100000 6118		Hypertension in chronic kidney disease stage 4 due to type 2 diabetes mellitus
804405100000 6116		Hypertension in chronic kidney disease stage 4 due to type II diabetes mellitus
804406100000 6119		Hypertension in chronic kidney disease stage 3 due to type 2 diabetes mellitus
804407100000 6114		Hypertension in chronic kidney disease stage 3 due to type II diabetes mellitus
804408100000 6112		Hypertension in chronic kidney disease stage 2 due to type 2 diabetes mellitus
804409100000 6110		Hypertension in chronic kidney disease stage 2 due to type II diabetes mellitus
804417100000 6110		Neuropathic toe ulcer due to type 2 diabetes mellitus
804418100000 6113		Ulcer of toe due to type 2 diabetes mellitus
804422100000 6116		Ischaemic foot ulcer due to type 2 diabetes mellitus

8044231000006118		Ischemic foot ulcer due to type 2 diabetes mellitus
8044241000006111		Neuropathic foot ulcer due to type 2 diabetes mellitus
8048421000006113		Proteinuria due to type 2 diabetes mellitus
8050091000006115		Foot ulcer due to type 1 diabetes mellitus
8050101000006114		Type 2 diabetes mellitus controlled by diet
8077141000006113		Proteinuria due to type 1 diabetes mellitus
8118151000006112		Hyperosmolarity co-occurrent and due to drug induced diabetes mellitus
8119331000006114		Hyperglycaemia due to type 1 diabetes mellitus
8119341000006116		Hyperglycemia due to type 1 diabetes mellitus
8119451000006114		Hyperglycaemia due to type 2 diabetes mellitus
8119461000006111		Hyperglycemia due to type 2 diabetes mellitus
8119711000006119		Dermatitis due to drug induced diabetes mellitus
8120371000006113		Dyslipidemia due to type 1 diabetes mellitus
8120381000006111		Neuropathy due to type 2 diabetes mellitus
8120391000006114		Cheiropathy due to type 2 diabetes mellitus
8120401000006111		Hyperosmolar coma due to secondary diabetes mellitus
8120431000006115		Mild nonproliferative retinopathy due to secondary diabetes mellitus
8120441000006113		Non-proliferative retinopathy due to secondary diabetes mellitus
8120451000006110		Moderate non-proliferative retinopathy due to secondary diabetes mellitus
82373015	F372.11	Diabetic polyneuropathy
8240511000006110		H/O: secondary diabetes mellitus
8264221000006117		Management of gestational diabetes mellitus
82979011	Q441.00	Neonatal diabetes mellitus
840971000006112	C10D.00	Diabetes mellitus autosomal dominant type II
841011000006112	F420800	Severe nonproliferative diabetic retinopathy
841351000006110	C109J12	Insulin treated Type II diabetes mellitus
842810000006115	C108.12	Type 1 diabetes mellitus
842910000006117	C108812	Type 1 diabetes mellitus - poor control
843010000006116	C108912	Type 1 diabetes mellitus maturity onset

84311000006118	C108H12	Type 1 diabetes mellitus with arthropathy
84321000006114	C108F12	Type 1 diabetes mellitus with diabetic cataract
84331000006112	C108612	Type 1 diabetes mellitus with gangrene
84341000006119	C108E12	Type 1 diabetes mellitus with hypoglycaemic coma
84351000006117	C108B12	Type 1 diabetes mellitus with mononeuropathy
84361000006115	C108312	Type 1 diabetes mellitus with multiple complications
84371000006110	C108D12	Type 1 diabetes mellitus with nephropathy
8437231000006117		Type 2 diabetes mellitus risk assessment declined
84381000006113	C108212	Type 1 diabetes mellitus with neurological complications
8438161000006115		Type 2 diabetes mellitus risk assessment invitation
8438181000006113		Type 2 diabetes mellitus risk assessment invitation first letter
8438201000006114		Type 2 diabetes mellitus risk assessment invitation second letter
8438221000006116		Type 2 diabetes mellitus risk assessment invitation third letter
8438241000006111		Type 2 diabetes mellitus risk assessment verbal invitation
8438261000006110		Type 2 diabetes mellitus risk assessment telephone invitation
8438281000006117		Type 2 diabetes mellitus risk assessment invitation SMS (short message service) text message
84391000006111	C108J12	Type 1 diabetes mellitus with neuropathic arthropathy
84401000006113	C108112	Type 1 diabetes mellitus with ophthalmic complications
8440231000006115		Eating disorder co-occurrent with diabetes mellitus type 1
8440241000006113		ED-DMT1 - Eating disorder-diabetes mellitus type 1
84411000006111	C108G12	Type 1 diabetes mellitus with peripheral angiopathy
84421000006115	C108C12	Type 1 diabetes mellitus with polyneuropathy
84431000006117	C108012	Type 1 diabetes mellitus with renal complications
84441000006110	C108712	Type 1 diabetes mellitus with retinopathy
84451000006112	C108512	Type 1 diabetes mellitus with ulcer
84461000006114	C108A12	Type 1 diabetes mellitus without complication
84471000006119	C109.12	Type 2 diabetes mellitus
84481000006116	C109712	Type 2 diabetes mellitus - poor control
84491000006118	C109G12	Type 2 diabetes mellitus with arthropathy

845010000061 14	C109E 12	Type 2 diabetes mellitus with diabetic cataract
845110000061 12	C1095 12	Type 2 diabetes mellitus with gangrene
845210000061 16	C109D 12	Type 2 diabetes mellitus with hypoglycaemic coma
845310000061 18	C109A 12	Type 2 diabetes mellitus with mononeuropathy
845410000061 11	C1093 12	Type 2 diabetes mellitus with multiple complications
845510000061 13	C109C 12	Type 2 diabetes mellitus with nephropathy
845610000061 10	C1092 12	Type 2 diabetes mellitus with neurological complications
845710000061 15	C109H 12	Type 2 diabetes mellitus with neuropathic arthropathy
845810000061 17	C1091 12	Type 2 diabetes mellitus with ophthalmic complications
845910000061 19	C109F 12	Type 2 diabetes mellitus with peripheral angiopathy
846010000061 10	C109B 12	Type 2 diabetes mellitus with polyneuropathy
846110000061 13	C1090 12	Type 2 diabetes mellitus with renal complications
846210000061 17	C1096 12	Type 2 diabetes mellitus with retinopathy
846310000061 19	C1094 12	Type 2 diabetes mellitus with ulcer
846410000061 12	C1099 12	Type 2 diabetes mellitus without complication
846510000061 14	C108.1 3	Type I diabetes mellitus
846610000061 11	C1088 11	Type I diabetes mellitus poorly controlled
846710000061 16	C1089 11	Type I diabetes mellitus maturity onset
846810000061 18	C108H 11	Type I diabetes mellitus with arthropathy
846910000061 15	C108F 11	Cataract due to diabetes mellitus type 1
847010000061 15	C1086 11	Type I diabetes mellitus with gangrene
847110000061 17	C108E 11	Type I diabetes mellitus with hypoglycaemic coma
847210000061 13	C108B 11	Mononeuropathy with type 1 diabetes mellitus
847310000061 11	C1083 11	Multiple complications of type 1 diabetes mellitus
847410000061 18	C108D 11	Type I diabetes mellitus with nephropathy
847510000061 16	C1082 11	Type I diabetes mellitus with neurological complications
847610000061 19	C108J1 1	Type I diabetes mellitus with neuropathic arthropathy
847710000061 14	C1081 11	Disorder of eye with type 1 diabetes mellitus
847810000061 12	C108G 11	Type I diabetes mellitus with peripheral angiopathy

84791000006110	C108C11	Polyneuropathy due to diabetes mellitus type I
84801000006111	C108011	Type I diabetes mellitus with renal complications
84811000006114	C108711	Type I diabetes mellitus with retinopathy
84821000006118	C108511	Type I diabetes mellitus with ulcer
84831000006115	C108A11	Type I diabetes mellitus without complication
84841000006113	C109.13	Type II diabetes mellitus
84851000006110	C109711	Type II diabetes mellitus - poor control
84861000006112	C109G11	Type II diabetes mellitus with arthropathy
84871000006117	C109E11	Cataract due to diabetes mellitus type 2
84881000006119	C109511	Type II diabetes mellitus with gangrene
84891000006116	C109D11	Type II diabetes mellitus with hypoglycaemic coma
84901000006117	C109A11	Mononeuropathy with type 2 diabetes mellitus
84911000006119	C109311	Type II diabetes mellitus with multiple complications
84921000006110	C109C11	Renal disorder due to type 2 diabetes mellitus
84931000006113	C109211	Neurological disorder with diabetes type 2
84941000006115	C109H11	Type II diabetes mellitus with neuropathic arthropathy
84951000006118	C109111	Disorder of eye with type 2 diabetes mellitus
84961000006116	C109F11	Type II diabetes mellitus with peripheral angiopathy
84971000006111	C109B11	Type II diabetes mellitus with polyneuropathy
84981000006114	C109011	Type II diabetes mellitus with renal complications
84991000006112	C109611	Type II diabetes mellitus with retinopathy
85001000006117	C109411	Type II diabetes mellitus with ulcer
85011000006119	C109911	Type II diabetes mellitus without complication
850691000006118	C109K00	Hyperosmolar non-ketotic state in type 2 diabetes mellitus
856771000006110		Patient has been told has diabetes
881441000006111	C100099	Diabetes mellitus - juvenile
881451000006113	C100199	Diabetes mellitus -adult onset
881461000006110	C101.99	Diabetes+ketoacidosis -no coma
881471000006115	C103.99	Diabetes with coma

881481000006117	C104.99	Diabetes + nephropathy
881491000006119	C105.99	Diabetes + eye manifestation
881501000006110	C106.99	Diabetes + neuropathy
881511000006113	C107.99	Diabetes + periph.circulat.dis
881521000006117	C10y.99	Diabetes + other complications
887971000006115	L180.99	Preg.+ diabetes mellitus
905621000006113		[RFC] Diabetes
908831000006118		[RFC] Diabetes mellitus
9093013	F420.00	Diabetic retinopathy
913441000006119	C10E012	Insulin-dependent diabetes mellitus with renal complications
913451000006117	C10E000	Type 1 diabetes mellitus with renal complications
913461000006115	C10E011	Type I diabetes mellitus with renal complications
913471000006110	C10E112	Insulin-dependent diabetes mellitus with ophthalmic comps
913481000006113	C10E100	Type 1 diabetes mellitus with ophthalmic complications
913491000006111	C10E111	Type I diabetes mellitus with ophthalmic complications
913501000006115	C10E212	Insulin-dependent diabetes mellitus with neurological comps
913511000006117	C10E200	Type 1 diabetes mellitus with neurological complications
913521000006113	C10E211	Neurological disorder with type 1 diabetes mellitus
913531000006111	C10E312	Insulin dependent diabetes mellitus with multiple complicat
913541000006118	C10E300	Type 1 diabetes mellitus with multiple complications
913551000006116	C10E311	Type I diabetes mellitus with multiple complications
913591000006110	C10E512	Skin ulcer associated with diabetes mellitus
913621000006112	C10E612	Insulin dependent diabetes mellitus with gangrene
913651000006115	C10E712	Insulin dependent diabetes mellitus with retinopathy
913661000006118	C10E700	Retinopathy with type 1 diabetes mellitus
913671000006113	C10E711	Type I diabetes mellitus with retinopathy
913681000006111	C10E812	Insulin dependent diabetes mellitus - poor control
913711000006112	C10E912	Insulin dependent diabetes maturity onset
913771000006115	C10EB12	Insulin dependent diabetes mellitus with mononeuropathy

913781000006117	C10EB00	Type 1 diabetes mellitus with mononeuropathy
913791000006119	C10EB11	Type I diabetes mellitus with mononeuropathy
913801000006118	C10EC12	Insulin dependent diabetes mellitus with polyneuropathy
913811000006115	C10EC00	Type 1 diabetes mellitus with polyneuropathy
913821000006111	C10EC11	Type I diabetes mellitus with polyneuropathy
913831000006114	C10ED12	Insulin dependent diabetes mellitus with nephropathy
913841000006116	C10ED00	Renal disorder associated with type 1 diabetes mellitus
913851000006119	C10ED11	Type I diabetes mellitus with nephropathy
913891000006113	C10EF12	Insulin dependent diabetes mellitus with diabetic cataract
913901000006112	C10EF00	Type 1 diabetes mellitus with diabetic cataract
913911000006110	C10EF11	Type I diabetes mellitus with diabetic cataract
913931000006116	C10EG00	Type 1 diabetes mellitus with peripheral angiopathy
913941000006114	C10EG11	Peripheral angiopathy due to type 1 diabetes mellitus
914031000006118	C10F000	Renal disorder associated with type II diabetes mellitus
914041000006111	C10F011	Type II diabetes mellitus with renal complications
914051000006113	C10F100	Disorder of eye with type 2 diabetes mellitus
914061000006110	C10F111	Type II diabetes mellitus with ophthalmic complications
914071000006115	C10F200	Neurologic disorder associated with type 2 diabetes mellitus
914081000006117	C10F211	Type II diabetes mellitus with neurological complications
914151000006112	C10F600	Retinopathy with type 2 diabetes mellitus
914161000006114	C10F611	Type II diabetes mellitus with retinopathy
914221000006113	C10FA00	Type 2 diabetes mellitus with mononeuropathy
914231000006111	C10FA11	Type II diabetes mellitus with mononeuropathy
914241000006118	C10FB00	Polyneuropathy due to type 2 diabetes mellitus
914251000006116	C10FB11	Type II diabetes mellitus with polyneuropathy
914261000006119	C10FC00	Diabetes type 2 with nephropathy
914271000006114	C10FC11	Type II diabetes mellitus with nephropathy
914301000006111	C10FE00	Type 2 diabetes mellitus with diabetic cataract
914311000006114	C10FE11	Type II diabetes mellitus with diabetic cataract

914391000006116	C10FJ00	Insulin treated Type 2 diabetes mellitus
928461000006119	C10EK00	Persistent proteinuria associated with type 1 diabetes mellitus
928471000006114	C10EK11	Type I diabetes mellitus with persistent proteinuria
928491000006110	C10EL11	Type I diabetes mellitus with persistent microalbuminuria
928501000006119	C10EM00	Ketoacidosis in type 1 diabetes mellitus
928511000006116	C10EM11	Type I diabetes mellitus with ketoacidosis
928521000006112	C10EN00	Ketoacidotic coma in type 1 diabetes mellitus
928531000006110	C10EN11	Type I diabetes mellitus with ketoacidotic coma
928541000006117	C10FL00	Persistent proteinuria associated with type 2 diabetes mellitus
928551000006115	C10FL11	Type II diabetes mellitus with persistent proteinuria
928561000006118	C10FM00	Persistent microalbuminuria associated with type 2 diabetes mellitus
928571000006113	C10FM11	Type II diabetes mellitus with persistent microalbuminuria
928581000006111	C10FN00	Ketoacidosis in type 2 diabetes mellitus
928591000006114	C10FN11	Type II diabetes mellitus with ketoacidosis
928601000006118	C10FP00	Ketoacidotic coma in type 2 diabetes mellitus
928611000006115	C10FP11	Ketoacidotic coma in type II diabetes mellitus
9312981000006113		Skin ulcer of toe due to diabetes mellitus type 1
9312991000006111		Diabetes type 1 with diabetic ulcer of toe, skin breakdown
9313001000006112		Skin ulcer of toe due to diabetes mellitus type 2
9313011000006110		Diabetes type 2 with diabetic ulcer of toe, skin breakdown
9313421000006110		Diabetic ulcer of left foot due to diabetes mellitus type 2
9313431000006113		Ulcer of left foot co-occurrent and due to diabetes mellitus type 2
9313441000006115		Diabetes type 2 with diabetic ulcer of left foot
9313571000006110		Diabetic ulcer of right foot due to diabetes mellitus type 2
9313581000006113		Diabetes type 2 with diabetic ulcer of right foot
9313591000006111		Ulcer of right foot co-occurrent and due to diabetes mellitus type 2
9324601000006113		Gestational diabetes mellitus in childbirth
9324871000006115		Diabetes mellitus in mother complicating childbirth
9324881000006117		Diabetes mellitus in childbirth

932641000006116	C103y99	Diabetes with coma
933231000006118	C10yz97	Hypo states in diabetes
938301000006114	C10EP00	Exudative maculopathy with type 1 diabetes mellitus
938311000006112	C10EP11	Type I diabetes mellitus with exudative maculopathy
938321000006116	C10FQ00	Exudative maculopathy associated with type 2 diabetes mellitus
938331000006118	C10FQ11	Exudative maculopathy with type 2 diabetes mellitus
958071000006117		DNA - Diabetes, Heart disease & Stroke prevention pilot project
96244016		Protein-deficient diabetes mellitus
967631000006115	C10H000	Diabetes mellitus induced by non-steroid drugs without complication
967681000006119	C10L.00	Fibrocalculous pancreatic diabetes
967701000006116	C10M.00	Lipoatrophic diabetes mellitus
98476015	F420100	Proliferative diabetic retinopathy
9986011		Drug-induced diabetes mellitus

Depression

medcode	readcode	desc
11776661000006110		[X]Single episode major depression w'out psychotic symptoms
11779381000006115		[X]Single episode agitated depressn w'out psychotic symptoms
11903751000006114		Single major depressive episode
11903771000006116		Single major depressive episode, in full remission
11903781000006118		Single major depressive episode, mild
11903791000006115		Single major depressive episode, moderate
11903801000006119		Single major depressive episode, severe
11914901000006114		[X]Depressive personality disorder
11918531000006118		[X]Mild depressive episode
11918561000006110		[X]Moderate depressive episode
11921141000006115		[X]Recurrent severe episodes/reactive depressive psychosis
11921301000006119		[X]Severe depressive episode without psychotic symptoms
11921311000006116		[X] Single episode agitated depression without psychotic symptoms

11921321000006112		[X] Single episode major depression without psychotic symptoms
12127531000006113		Recurrent mild major depressive disorder co-occurrent with anxiety
12127541000006115		Recurrent severe major depressive disorder co-occurrent with anxiety
12127551000006118		Recurrent moderate major depressive disorder co-occurrent with anxiety
12127561000006116		Recurrent major depressive disorder co-occurrent with anxiety in full remission
12127571000006111		Recurrent major depressive disorder in partial remission co-occurrent with anxiety
1222477019	R007z13	[D]Postoperative depression
1231868010	62T1.00	Puerperal depression
12451451000006110		Single major depressive episode, in full remission
12451461000006112		Single major depressive episode, moderate
12452121000006118		Recurrent major depressive episodes, in full remission
12727931000006110		Depressive disorder NEC
138421012	E135.00	Agitated depression
142521000006110	E112500	Major depression single episode, in partial remission
142541000006115	E112300	Severe major depression, single episode
1488626018	1B1U.00	Symptoms of depression
1494612017	1B1U.11	Depressive symptoms
1650771000000113	1JJ..00	Suspected depression
1680571000006118	9kQ..00	On full dose long term treatment depression - enh serv admin
1715181000006114	Eu32600	Moderate major depression
1715191000006112	Eu32800	Severe major depression with psychotic features
1715771000006112	Eu32500	Mild major depression
1715781000006110	Eu32700	Severe major depression without psychotic features
1755901000006112	Eu32900	[X]Single major depressive episode, severe, with psychosis, psychosis in remission
1755911000006110	Eu32A00	[X]Recurrent major depressive episodes, severe, with psychosis, psychosis in remission
1771531000006110		Antenatal depression
1785881000006119	Eu32B00	Antenatal depression

18064310000061 13		Adjustment disorder with depressed mood
18238810000061 10		Depression confirmed
18272100000611 1	E113.00	Recurrent major depressive episodes
18277100000611 2	E11330 0	Recurrent major depressive episodes, severe
18280100000611 4	E11350 0	Recurrent major depressive episodes,partial/unspec remission
18395510000061 16		PHQ9 total score 5-9 (mild depression)
18395610000061 19		PHQ9 total score 10-14 (moderate depression)
18395710000061 14		PHQ9 total score 15-19 (moderately severe depression)
18395810000061 12		PHQ9 total score 20-27 (severe depression)
19720710000061 18		Unspecified dementia, other symptoms, predominantly depressive
19721110000061 14		Organic depressive disorder
19721310000061 15		Dementia in Alzheimer's disease with early onset, other symptoms, predominantly depressive
19722010000061 18		Dementia in Alzheimer's disease with late onset, other symptoms, predominantly depressive
19723110000061 12		Dementia in Alzheimer's dis, atypical or mixed type, other symptoms, predominantly depressive
19724510000061 18		Dementia in Alzheimer's disease, unspecified, other symptoms, predominantly depressive
19725410000061 19		Vascular dementia of acute onset, other symptoms, predominantly depressive
19726610000061 19		Multi-infarct dementia, other symptoms, predominantly depressive
19729110000061 11		Mixed cortical and subcortical vascular dementia, other symptoms, predominantly depressive
19735510000061 12		Vascular dementia, unspecified, other symptoms, predominantly depressive
19751910000061 14		Post-schizophrenic depression, continuous
19752110000061 10		Post-schizophrenic depression, episodic with progressive deficit
19752310000061 16		Post-schizophrenic depression, episodic with stable deficit
19752610000061 13		Post-schizophrenic depression, episodic remittent
19752810000061 15		Post-schizophrenic depression, incomplete remission
19753010000061 16		Post-schizophrenic depression, complete remission
19753210000061 14		Post-schizophrenic depression, course uncertain, period of observation too short

1975981000006114		Mild depressive episode, without somatic syndrome
1975991000006112		Mild depressive episode, with somatic syndrome
1976021000006115		Moderate depressive episode, without somatic syndrome
1976051000006112		Moderate depressive episode, with somatic syndrome
1976211000006116		Recurrent depressive disorder, current episode mild, without somatic syndrome
1976231000006110		Recurrent depressive disorder, current episode mild, with somatic syndrome
1976251000006115		Recurrent depressive disorder, current episode moderate, without somatic syndrome
1976271000006113		Recurrent depressive disorder, current episode moderate, with somatic syndrome
1976491000006113		Mixed anxiety and depressive reaction
1976921000006116		Post-schizophrenic depression, other
202561000006114	E291.00	Prolonged depressive adjustment reaction
213641000000111	Eu32400	[X]Mild depression
2164005017	1BT..00	Depressed mood
2164006016	1B17.00	Depressed
223651000000118	E204.11	Postpartum depression
223741000000112	Eu32212	Severe depression
2474715017	9HA0.00	On depression register
251629019	1465.00	H/O: depression
253619019	2257.00	O/E - depressed
2540711000006115		Chronic recurrent major depressive disorder
2620391000000111		Maternal postnatal depression
2667431000006115		Primary degenerative dementia of the Alzheimer type, presenile onset, with depression
2667441000006113		Dementia of the Alzheimer's type, with early onset, with depressive mood
2667451000006110		Alzheimers dementia, early onset, with depressed mood
2722931000006114		Multi-infarct dementia with depression
2722941000006116		Vascular dementia, with depressive mood
2724681000006112		Chronic major depressive disorder, single episode
2729751000000118		Recurrent depression with current severe episode and psychotic features

2729991000000112		Recurrent depression with current severe episode without psychotic features
2730011000000110		Recurrent depression with current moderate episode
2740851000006114		Severe recurrent major depression with psychotic features, mood-incongruent
2748031000006110		Moderate major depression, single episode
2798691000006112		Moderate recurrent major depression
2809811000006111		Single episode of major depression in full remission
2809821000006115		Major depression, single episode, in complete remission
2809831000006117		Major depression, single episode, in full remission
2821731000006115		Severe major depression, single episode, with psychotic features, mood-incongruent
2914841000006115		Major depressive disorder, single episode with postpartum onset
2929931000006112		Primary degenerative dementia of the Alzheimer type, senile onset, with depression
2929941000006119		Dementia of the Alzheimer's type, with late onset, with depressive mood
2929951000006117		Alzheimers dementia, late onset, with depressive mood
294642013	E001300	Presenile dementia with depression
294644014	E002.00	Senile dementia with depressive or paranoid features
294646011	E002100	Senile dementia with depression
294647019	E002z00	Senile dementia with depressive or paranoid features NOS
294655014	E004300	Arteriosclerotic dementia with depression
294824018	E112000	Single major depressive episode, unspecified
294825017	E112100	Mild major depression, single episode
294826016	E112200	Moderate major depression, single episode
294828015	E112400	Single major depressive episode, severe, with psychosis
294831019	E112600	Single episode of major depression in full remission
294832014	E112z00	Single major depressive episode NOS
294836012	E113000	Recurrent major depressive episodes, unspecified
294837015	E113100	Recurrent major depressive episodes, mild
294838013	E113200	Recurrent major depressive episodes, moderate

294840015	E113400	Recurrent major depressive episodes, severe, with psychosis
294843018	E113600	Recurrent major depression in full remission
294844012	E113700	Recurrent depression
294845013	E113z00	Recurrent major depressive episode NOS
294894013	E11y200	Atypical depressive disorder
294917018	E130.00	Reactive depressive psychosis
294918011	E130.11	Psychotic reactive depression
295494011	E290z00	Brief depressive reaction NOS
295535012	E2B..00	Depressive disorder
295536013	E2B0.00	Postviral depression
295537016	E2B1.00	Chronic depression
2957311000006113		Severe recurrent major depression with psychotic features
296137015	Eu32000	Mild depression
296138013	Eu32100	[X]Moderate depressive episode
296180012	Eu33000	[X]Recurrent depressive disorder, current episode mild
296181011	Eu33100	[X]Recurrent depressive disorder, current episode moderate
296198011	Eu33400	[X]Recurrent depressive disorder, currently in remission
296199015	Eu33y00	[X]Other recurrent depressive disorders
2993091000006116		Major depression in partial remission
3032461000006117		Severe recurrent major depression with psychotic features, mood-congruent
3033261000006113		Recurrent major depression in partial remission
3042901000006110		Severe major depression with psychotic features, mood-congruent
3071791000006111		Depressive illness
3071801000006112		Depressed
3071811000006110		Mood disorder of depressed type
3087971000006112		Severe recurrent major depression without psychotic features
3094941000006114		Major depression, single episode
3094951000006111		Major depressive disorder, single episode
3122721000006111		Recurrent major depressive disorder with atypical features

3140411000006112		Recurrent major depressive disorder with catatonic features
3149661000006117		Mild recurrent major depression
3153071000006116		Recurrent brief depressive disorder
3186661000006118		Organic mood disorder of depressed type
3190221000006112		Major depression in remission
3190231000006110		Major depression, in remission
3192061000006110		Major depressive disorder, single episode with atypical features
3247071000006112		Recurrent major depression in full remission
3247081000006110		Recurrent major depression in complete remission
3286331000006115		Minor depressive disorder
346972018	E112.13	Endogenous depression first episode
346973011	E11z200	Masked depression
3475971000006117		Severe major depression with psychotic features, mood-incongruent
3517985017		Postpartum major depression in remission
3527827013		Major depressive disorder clinical management plan
3531371000006112		Major depression in complete remission
3531381000006110		Major depression in full remission
3537261000006119		Major depressive disorder, single episode with melancholic features
3578851000006118		Recurrent major depression
3578861000006116		Recurrent major depressive disorder
3591210000061116	Eu32z14	Reactive depression
3606411000006115		Recurrent major depression in remission
3628471000006116		Major depressive disorder, single episode with catatonic features
3650771000006113		Major depression, single episode, in partial remission
3660551000006117		Recurrent major depressive disorder with postpartum onset
3665610000061119	Eu32y11	[X]Atypical depression
3670610000061114	Eu31500	Psychosis and severe depression co-occurrent and due to bipolar affective disorder
369982012	E118.00	Seasonal affective disorder

37009110000061 15		Psychotic depression
37332010000061 14		Mood disorder with depressive features due to general medical condition
37332110000061 12		Mood disorder, condition with depressive features
37434510000061 10		Severe major depression, single episode, without psychotic features
37669100000611 6	Eu32z1 1	Depression
37670100000611 6	Eu9200 0	[X]Depressive conduct disorder
37671100000611 8	Eu32z1 2	[X]Depressive disorder NOS
37672100000611 4	Eu32.00	Depressive episode
37674100000611 9	Eu3411 1	[X]Depressive neurosis
37675100000611 7	Eu3411 2	Depressive personality disorder
37675310000061 19		Severe major depression, single episode, with psychotic features, mood-congruent
37895810000061 12		Mild major depression, single episode
37943100000611 3	Eu3410 0	Dysthymia
37977100000611 6	Eu3331 1	[X]Endogenous depression with psychotic symptoms
37978100000611 8	Eu3321 1	[X]Endogenous depression without psychotic symptoms
38371210000061 16		Postoperative depression
38790310000061 13		Postmenopausal depression
39608100000611 6	Eu3321 2	[X]Major depression, recurrent without psychotic symptoms
39835100000611 0	Eu4121 1	[X]Mild anxiety depression
39856100000611 7	Eu4120 0	[X]Mixed anxiety and depressive disorder
39884100000611 9	Eu33z1 1	[X]Monopolar depression NOS
39996100000611 8	Eu3411 3	Depressive neurosis
401766011	E112.00	Major depression, single episode
401866015	Eu3220 0	[X]Severe depressive episode without psychotic symptoms
401869010	Eu3230 0	[X]Severe depressive episode with psychotic symptoms
401871010	Eu32y0 0	[X]Other depressive episodes
401872015	Eu32z0 0	[X]Depressive episode, unspecified

401873013	Eu33.00	[X]Recurrent depressive disorder
401876017	Eu33z00	[X]Recurrent depressive disorder, unspecified
407062014	1B17.11	C/O - feeling depressed
410861011	E113.11	Endogenous depression - recurrent
419841000006116	Eu34114	[X]Persistant anxiety depression
423041000006119	Eu20400	[X]Post-schizophrenic depression
423131000006113	Eu53011	[X]Postnatal depression NOS
423141000006115	Eu53012	[X]Postpartum depression NOS
423611000006111	Eu32z13	[X]Prolonged single episode of reactive depression
424531000006118	Eu33200	[X]Recurr depress disorder cur epi severe without psyc sympt
424541000006111	Eu33313	[X]Recurr severe episodes/major depression+psychotic symptom
424551000006113	Eu33314	[X]Recurr severe episodes/psychogenic depressive psychosis
424561000006110	Eu3y111	[X]Recurrent brief depressive episodes
424571000006115	Eu33300	[X]Recurrent depress disorder cur epi severe with psyc symp
424631000006119	Eu33.11	[X]Recurrent episodes of depressive reaction
424641000006112	Eu33.12	[X]Recurrent episodes of psychogenic depression
424651000006114	Eu33.13	[X]Recurrent episodes of reactive depression
424671000006116	Eu33315	[X]Recurrent severe episodes of psychotic depression
424681000006118	Eu33316	Recurrent reactive depressive episodes, severe, with psychosis
425411000006110	Eu33.15	SAD - Seasonal affective disorder
425501000006113	Eu25100	Schizoaffective disorder, depressive type
425561000006114	Eu25111	[X]Schizoaffective psychosis, depressive type
425691000006119	Eu25112	[X]Schizophreniform psychosis, depressive type
425751000006115	Eu33.14	[X]Seasonal depressive disorder
426891000006114	Eu32211	[X] Single episode agitated depression without psychotic symptoms
426911000006111	Eu32.11	[X]Single episode of depressive reaction
426921000006115	Eu32311	[X]Single episode of major depression and psychotic symptoms

426931000006117	Eu32y12	[X]Single episode of masked depression NOS
426941000006110	Eu32.12	[X]Single episode of psychogenic depression
426951000006112	Eu32312	[X]Single episode of psychogenic depressive psychosis
426961000006114	Eu32313	[X]Single episode of psychotic depression
426971000006119	Eu32.13	[X]Single episode of reactive depression
426981000006116	Eu32314	[X]Single episode of reactive depressive psychosis
426991000006118	Eu32213	[X]Single episode vital depression w'out psychotic symptoms
432511000006119	Eu33214	[X]Vital depression, recurrent without psychotic symptoms
441826016	E112.14	Endogenous depression
4539951000006117		History of depressive disorder
4539961000006115		Has had depression
4539971000006110		History of depression
4550951000006112		On examination - depressed
474171000006112	E112.11	Agitated depression
488211000006112	E200300	Mixed anxiety and depressive disorder
5023901000006110		Post-schizophrenic depression
5024071000006118		Anxiety depression
5024511000006118		Depressive conduct disorder
5106051000006119		Mild postnatal depression
5106061000006117		Severe postnatal depression
5247381000006117		Depressive delusion of catastrophe
5247391000006119		Depressive delusion of poverty
5247411000006119		Depressive hypochondriacal delusion
5248531000006111		Depressive preoccupation
5248911000006110		Depression worse in morning
5248931000006116		Depression worse later in day
525921000006119	E290.00	Brief depressive adjustment reaction

5291491000006114		Single stimulus depression - finding
5291501000006118		Single stimulus depression
5359011000006112		Depression - motion
5359021000006116		Depression
5532721000006114		Complaining of feeling depressed
5934641000006110		Depressive position relationship
6000691000006114		Mild depression
6000711000006112		Moderate depression
6000721000006116		Severe depression
6081011000006117		Recurrent major depressive disorder with melancholic features
6088151000006111		Major depression, melancholic type
6095581000006115		Involutional depression
613781000006118	E211200	Depressive personality disorder
613791000006115	E11..12	Depressive psychoses
6337491000006116		Major depressive disorder
6337501000006112		Major depression
642461000006116	E112.12	Endogenous depression first episode
675861000006113	E204.00	Reactive depression (situational)
681801000000113	9kQ..11	On full dose long term treatment for depression
7127651000006115		Severe major depression, single episode, with psychotic features
7127661000006118		Major depressive disorder, single episode, severe with psychotic features
7259021000006112		Chronic depressive personality disorder
7382291000006112		Severe major depression
7483151000006113		Suspected depressive disorder
7511521000006119		Adjustment disorder with depressed mood in remission
7515151000006115		Depressive disorder in remission

7593831000006115		Depressed mood with postpartum onset
7593841000006113		Depressed mood in postpartum period
7695931000006118		Acute depression
7775181000006118		Minimal depression
7788651000006116		Moderately severe major depression
7788661000006119		Moderately severe depression
7803691000006116		Minimal recurrent major depression
7803701000006116		Moderately severe recurrent major depression
7803711000006118		Moderately severe major depression single episode
7803721000006114		Moderately severe major depression one episode
7803731000006112		Minimal major depression single episode
7803741000006119		Minimal major depression one episode
7803751000006117		Minimal major depression
7806991000006116		Major depressive disorder clinical management plan
7859461000006119		Anxiolytic-induced mood disorder with depressive symptoms
7859581000006111		Anxiolytic-induced mood disorder with mixed depressive and manic symptoms
7860801000006116		Depressive symptoms co-occurrent and due to primary psychotic disorder
7860811000006118		Depressive symptoms with primary psychotic disorder
7882721000006118		Major depression with psychotic features
7965131000006117		Severe major depression, single episode
7965191000006118		Severe recurrent major depression
7991681000006114		Perinatal depression
8030051000006110		Depressive disorder in mother complicating pregnancy
8044941000006117		Depressed mood in Alzheimer's disease
8044951000006115		Alzheimers dementia with depressed mood
8044961000006118		Depressed mood in Alzheimer disease

8089301000006110		Reactive depressive psychosis, single episode
8100481000006111		History of postnatal depression
8135001000006112		[X]Severe depressive episode with psychotic symptoms
8232571000006118		Single major depressive episode, severe, with psychosis, psychosis in remission
8232591000006117		Recurrent major depressive episodes, severe, with psychosis, psychosis in remission
8237831000006115		Recurrent major depressive episodes, in partial remission
8237841000006113		Recurrent major depressive episodes, in remission
8237851000006110		Single major depressive episode, in remission
8460861000006115		Depressive personality disorder
8464961000006115		Recurrent reactive depressive episodes, severe, with psychosis
8465321000006112		Reactive depression, prolonged single episode
8465331000006110		Prolonged single episode of reactive depression
8465341000006117		Reactive depression, single episode
8465351000006115		Reactive depression, recurrent
8465361000006118		Reactive depression, first episode
853871000006111		Post natal depression
882401000006115	E204.99	Reactive (neurotic) depression
882421000006113	E211299	Depressive personality
882671000006112	E2B..98	Depression
882681000006110	E2B..99	Depression NOS
882811000006119	Eu32099	Mild depression
882821000006110	Eu32199	Moderate depression
882831000006113	Eu32299	Severe depression
908731000006114		[RFC] Postnatal depression
909681000006110		[RFC] Depression
9331521000006118		Major depressive disorder in mother complicating childbirth

93315310000061<br>15		Major depressive disorder in childbirth
93315410000061<br>13		Major depressive disorder in mother complicating pregnancy
93315510000061<br>10		Major depressive disorder in pregnancy
93341610000061<br>18		Depressive disorder in mother complicating childbirth
93341710000061<br>13		Depression in childbirth
98161100000611<br>5		Abnormal depressed feelings
99011610000061<br>19		Mild major depressive disorder co-occurrent with anxiety single episode
99012610000061<br>14		Moderate major depressive disorder co-occurrent with anxiety single episode
99012810000061<br>16		Severe major depressive disorder co-occurrent with anxiety single episode

Epilepsy

medcode	readc<br>ode	desc
10353100000<br>6110	F2510<br>11	Tonic-clonic epilepsy
10791000006<br>116	F250y<br>00	Other specified generalised nonconvulsive epilepsy
11300012	F25A.<br>00	Juvenile myoclonic epilepsy
11831371000<br>006117		Amish infantile epilepsy syndrome
11831381000<br>006119		Infantile-onset symptomatic epilepsy syndrome
12003041000<br>006118		Progressive myoclonic epilepsy with dystonia
12003051000<br>006116		Progressive myoclonus epilepsy with dystonia
12006401000<br>006116		Hot water reflex epilepsy
12007971000<br>006115		Thinking epilepsy
12010351000<br>006112		Micturition induced epilepsy
12010621000<br>006112		Orgasm induced epilepsy
12011241000<br>006112		Pachygyria, intellectual disability, epilepsy syndrome
12016641000<br>006119		Progressive myoclonic epilepsy type 4
12017661000<br>006117		FFEVF - familial focal epilepsy with variable foci
12026111000<br>006117		Focal epilepsy, intellectual disability, cerebro-cerebellar malformation syndrome

12026121000006113		Focal epilepsy, intellectual disability, dysarthria, ataxia syndrome
12026171000006114		Rolandic epilepsy, speech dyspraxia syndrome
12027451000006111		SCN8A-related epilepsy with encephalopathy
12027471000006118		SCN8A (sodium voltage-gated channel alpha subunit 8) related epilepsy with encephalopathy
12027481000006115		Early infantile epileptic encephalopathy 13
12027491000006117		Sodium voltage-gated channel alpha subunit 8-related epilepsy with encephalopathy
12028401000006113		Audiogenic epilepsy
12073681000006111		PURA-related severe neonatal hypotonia, seizure, encephalopathy syndrome
12076991000006114		STXBP1 encephalopathy with epilepsy
12077011000006115		STXBP1 (syntaxin binding protein 1) epileptic encephalopathy
12077021000006111		Syntaxin binding protein 1 encephalopathy with epilepsy
12077031000006114		Early infantile epileptic encephalopathy 4
12077041000006116		STXBP1-related epileptic encephalopathy
12221091000006111		Aphasia co-occurrent with epilepsy
12359581000006111		Progressive epilepsy-intellectual disability syndrome Finnish type
12368901000006113		Intellectual disability, epileptic seizures, hypogonadism and hypogenitalism, microcephaly, obesity syndrome
12368911000006111		X-linked intellectual disability, epileptic seizures, hypogonadism and hypogenitalism, microcephaly, obesity syndrome
12485141000006112		Epilepsy drug side effects
125721000006111	F25X.00	Status epilepticus, unspecified
12618161000006115		Early onset or syndromic epilepsy germline WGS (whole genome sequencing) targeting early onset or syndromic epilepsy panel
137801000000112	667K.00	Epilepsy limits activities
141981000000118	667G.00	Epilepsy restricts employment
141991000000116	667H.00	Epilepsy prevents employment
142041000000115	667L.00	Epilepsy does not limit activities
148427014	F25y100	Gelastic epilepsy
148591000006114	F255100	Sensory induced epilepsy

1489345013	1B1W.00	Transient epileptic amnesia
154851000000115	667J.00	Epilepsy impairs education
1776671000006116		Reason for referral: Epilepsy
178739011	F25..00	Epilepsy
2159216019	667N.00	Epilepsy severity
2159218018	667Q.00	1 to 12 seizures a year
2159219014	667R.00	2 to 4 seizures a month
2159220015	667S.00	1 to 7 seizures a week
2159221016	667T.00	Daily seizures
2159227017	1O30.00	Epilepsy confirmed
2159274014	F254500	Complex partial epileptic seizure
223811000000118	F251000	Generalised epilepsy
230321000006117	F250000	Petit mal (minor) epilepsy
23820010	F253.11	Status epilepticus
245341000006117	F255.00	Localisation-related epilepsy
245351000006115	F255z00	Partial epilepsy
245361000006118	F255y00	Partial epilepsy without impairment of consciousness OS
2474650018	F25E.00	Stress-induced epilepsy
2478828012	SC20000	Traumatic epilepsy
2502351000006114		Epilepsia partialis continua
2502381000006118		Kojewnikov's epilepsy
2502401000006118		Kojevnikov epilepsy
251269010	13ZD.00	Witnessed epileptic seizure
251644018	1473.00	H/O: epilepsy
2533404015	8CE7.00	Epilepsy leaflet given
2534041000006111		Visceral epilepsy

2550890017	F250.00	Generalised non-convulsive epilepsy
2587911000000119		Neurological Disorders Depression Inventory in Epilepsy
2589101000000111		SUDEP - sudden unexpected death in epilepsy
2598861000006119		Juvenile myoclonic epilepsy of Janz
2598871000006114		JME - Juvenile myoclonic epilepsy
2598891000006110		Impulsive petit-mal epilepsy
2598901000006114		Myoclonic epilepsy of adolescence
2612111000006116		Epileptic absence status
2612121000006112		Non-convulsive status epilepticus with impaired consciousness
2612131000006110		Prolonged epileptic twilight state
2612141000006117		Epilepsia minoris continua
2622261000006115		Reading epilepsy
264634019	6674.00	Epilepsy associated problems
264637014	6677.00	Epilepsy drug side effect
2746121000006114		Benign focal epilepsy of childhood
2767851000006116		Musicogenic epilepsy
2811081000006110		Generalized epilepsy
2826531000006116		Secondarily generalised seizures
2826541000006114		Secondarily generalized seizures
297266016	F250200	Epileptic seizures - atonic
297267013	F250300	Epileptic seizures - akinetic
297281010	F251100	Neonatal myoclonic epilepsy
297282015	F251200	Epileptic seizures - clonic
297283013	F251300	Epileptic seizures - myoclonic
297284019	F251400	Epileptic seizures - tonic
297289012	F251y00	Other specified generalised convulsive epilepsy

297290015	F251z 00	Generalised convulsive epilepsy NOS
297295013	F254. 00	Partial epilepsy with impairment of consciousness
297296014	F2540 00	Temporal lobe epilepsy
297300010	F2542 00	Psychosensory epilepsy
297302019	F2544 00	Epileptic automatism
297303012	F254z 00	Partial epilepsy with impairment of consciousness NOS
297311019	F2552 00	Somatosensory epilepsy
297312014	F2553 11	Partial epilepsy with autonomic symptoms
297313016	F2553 00	Visceral reflex epilepsy
297315011	F2555 00	Unilateral epilepsy
297321010	F257. 00	Kojevnikov's epilepsy
297324019	F25y. 00	Other forms of epilepsy
297325018	F25y0 00	Cursive (running) epilepsy
297330019	F25yz 00	Other forms of epilepsy NOS
299334015	Fyu50 00	[X]Other generalized epilepsy and epileptic syndromes
299336018	Fyu51 00	[X]Other epilepsy
299337010	Fyu52 00	[X]Other status epilepticus
299345017	Fyu59 00	[X]Status epilepticus, unspecified
3093151000006111		Idiopathic generalised epilepsy
3093161000006113		Idiopathic generalized epilepsy
3093171000006118		Primary generalised epilepsy
3093181000006115		Primary generalized epilepsy
3130661000006113		Visual epilepsy
3139271000006111		Chronic progressive epilepsia partialis continua
3209051000006118		Postepileptic confusion
3210931000006115		Centralopathic epilepsy

32109410000 06113		Temporal-central focal epilepsy
32109510000 06110		Centrotemporal epilepsy
33008110000 06114		Postepileptic delirium
33058310000 06114		Centrencephalic epilepsy
33238510000 06114		Childhood absence epilepsy
33238710000 06116		Petit-mal epilepsy
33238910000 06115		Childhood - juvenile - absence epilepsy
34411710000 06118		Epileptic aura
345225017	F2550 11	Focal epilepsy
345275015	F2504 00	Juvenile absence epilepsy
345311015	F25G. 00	Severe myoclonic epilepsy in infancy
345318014	F25C. 00	Drug-induced epilepsy
345321011	F25D. 00	Menstrual epilepsy
345323014	667B. 00	Nocturnal epilepsy
345345010	F25y3 00	Complex partial status epilepticus
34795510000 06118		Light sensitive partial seizure
3506708010		Pyridoxine-dependent epilepsy
3510617017		Intractable idiopathic partial epilepsy
3511291019		Recurrent complex partial epilepsy
35587810000 06118		Generalized convulsive epilepsy
36081210000 06115		Myoclonic epilepsy with choreoathetosis
36137610000 06115		Myoclonus epilepsy AND ragged red fibres
36137710000 06110		Myoclonus epilepsy AND ragged red fibers
36184510000 06117		Epileptic vertigo
36184610000 06115		Vertiginous epilepsy
36216100000 6110	Eu803 00	Acquired epileptic aphasia
36683210000 06119		Symptomatic generalised epilepsy
36683310000 06116		Symptomatic generalized epilepsy

36683410000 06114		Secondary generalized epilepsy
36683510000 06111		Secondary generalised epilepsy
36983110000 06112		Epileptic cry
37196610000 06115		PTE - Post-traumatic epilepsy
37599310000 06112		Unclassified epileptic seizures
37968910000 06117		Reflex epilepsy
37969010000 06118		Sensory-induced epilepsy
37969110000 06115		Epilepsy associated with specific stimuli
38784810000 06113		Epileptic disorder
38784910000 06111		Epileptic
38785010000 06115		EP - Epilepsy
399395015	F1321 00	Progressive myoclonic epilepsy
399408014	F25z.0 0	Epilepsy NOS
40529210000 06118		Photogenic epilepsy
40529310000 06115		Television epilepsy
40529410000 06113		Photic epilepsy
40529510000 06110		Epilepsy only in relation to photic stimulation
40529710000 06117		Pure photosensitive epilepsy
41649010000 06116		Epilepsy, dementia and amelogenesis imperfecta
41649110000 06118		Epilepsy, mental deterioration and yellow teeth
41963810000 06118		Extratemporal epilepsy
42563100000 6118	Eu052 12	[X]Schizophrenia-like psychosis in epilepsy
450602015	F2550 12	Motor epilepsy
450603013	F2550 00	Jacksonian, focal or motor epilepsy
453344019	F25z.1 1	Epileptic seizure
45401410000 06110		History of epilepsy

4566371000006112		On examination - no fit/convulsion seen
459233015	667C.00	Epilepsy control good
459234014	667D.00	Epilepsy control poor
4636071000006111		Epilepsy drug side effect
472410017	F251500	Tonic-clonic epilepsy
472411018	F251099	Grand mal epilepsy
4755771000006114		Epileptic drop attack
4769481000006114		Generalized non-convulsive epilepsy
4769491000006112		Generalized nonconvulsive epilepsy
4769501000006116		Generalised nonconvulsive epilepsy
4769541000006119		Benign myoclonic epilepsy in infancy
4769611000006117		TLE - Temporal lobe epilepsy
4769641000006118		Mesiobasal limbic epilepsy
4769701000006110		Epileptic complex partial seizures with automatisms
4769771000006116		Cursive epilepsy
4769781000006118		Running epilepsy
4769801000006119		Localization-related(focal)(partial)idiopathic epilepsy and epileptic syndromes with seizures of localized onset
477355017	F254100	Psychomotor epilepsy
478024019	F25B.00	Alcohol-induced epilepsy
495804010	F250100	Pykno-epilepsy
500033015	F251.00	Generalised convulsive epilepsy
5007071000006118		Local epilepsy
5007091000006117		Localization-related epilepsy
5007111000006114		Benign psychomotor epilepsy of childhood
5007121000006118		Benign atypical partial epilepsy in childhood
5007131000006115		Epilepsy with recurrent unilateral seizures in children

5007141000006113		Childhood epilepsy with occipital paroxysms
5007151000006110		Benign occipital epilepsy of childhood
5007161000006112		BCEOP - Benign childhood epilepsy with occipital paroxysms
5007171000006117		Benign occipital epilepsy of childhood - early onset variant
5007191000006116		Benign occipital epilepsy of childhood - late onset variant
5007201000006118		Primary inherited reading epilepsy
5007211000006115		Localisation-related symptomatic epilepsy
5007221000006111		Localization-related symptomatic epilepsy
5007231000006114		Amygdalo-hippocampal epilepsy
5007241000006116		Rhinencephalic epilepsy
5007251000006119		Lateral temporal epilepsy
5007261000006117		Frontal lobe epilepsy
5007271000006112		Supplementary motor epilepsy
5007281000006110		Cingulate epilepsy
5007291000006113		Anterior frontopolar epilepsy
5007301000006114		Orbitofrontal epilepsy
5007311000006112		Dorsolateral epilepsy
5007321000006116		Opercular epilepsy
5007351000006113		Parietal lobe epilepsy
5007361000006110		Occipital lobe epilepsy
5007371000006115		Chronic progressive epilepsia partialis continua of childhood
5007391000006119		Localisation-related symptomatic epilepsy with specific precipitant
5007401000006117		Localization-related symptomatic epilepsy with specific precipitant
5007411000006119		Hemiplegia-hemiconvulsion-epilepsy syndrome
5007421000006110		Hemiconvulsion-hemiplegia-epilepsy syndrome
5007441000006115		Localisation-related cryptogenic epilepsy

50074510000 06118		Localization-related cryptogenic epilepsy
50074810000 06114		Benign neonatal epilepsy
50075210000 06114		Myoclonic epilepsy of early childhood
50075410000 06119		Epilepsy with grand mal seizures on awakening
50075510000 06117		Cryptogenic generalised epilepsy
50075610000 06115		Cryptogenic generalized epilepsy
50076210000 06113		Myoclonic astatic epilepsy
50076310000 06111		Epilepsy with myoclonic and astatic seizures
50076610000 06119		Myoclonic absence epilepsy
50076710000 06114		Epilepsy with myoclonic absences
50077010000 06110		Baltic myoclonus epilepsy
50077310000 06119		Myoclonic epilepsy - ragged red fibres
50077410000 06112		MERRF - Myoclonic epilepsy - ragged red fibres
50077510000 06114		MERRF - Myoclonic epilepsy - ragged red fibers
50077610000 06111		Myoclonic epilepsy - ragged red fibers
50077710000 06116		Cryptogenic myoclonic epilepsy
50077810000 06118		Idiopathic myoclonic epilepsy
50078110000 06116		Symptomatic myoclonic epilepsy
50078710000 06113		Epilepsy undetermined whether focal or generalised
50078810000 06111		Epilepsy undetermined whether focal or generalized
50079310000 06117		Acquired aphasia with epilepsy
50079410000 06110		Epilepsy with continuous spike wave during slow-wave sleep
50079710000 06119		Secondary reading epilepsy
50079910000 06118		Narcotic withdrawal epilepsy
50080110000 06114		Catamenial epilepsy
50080310000 06115		Sleep-related epilepsy

50080410000 06113		Sleep related epilepsy
50080910000 06116		Writing epilepsy
50081010000 06110		Eating epilepsy
50081210000 06117		Decision-making epilepsy
50081310000 06119		Aquagenic epilepsy
50081410000 06112		Bathing epilepsy
50081510000 06114		Immersion-related epilepsy
50081610000 06111		Self-induced non-photosensitive epilepsy
50081910000 06115		Non-convulsive status epilepticus with three per second spike wave
50082010000 06117		Non-convulsive status epilepticus with 3/s spike wave
50082110000 06119		Non-convulsive status epilepticus without three per second spike wave
50082210000 06110		Non-convulsive status epilepticus without 3/s spike wave
50082310000 06113		Non-convulsive simple partial status epilepticus
50235310000 06114		Epileptic psychosis
50235710000 06112		Limbic epilepsy personality syndrome
50677110000 06114		Triple X syndrome, epilepsy, and hypogammaglobulinaemia
50677210000 06118		Triple X syndrome, epilepsy, and hypogammaglobulinemia
512000013	F25F. 00	Photosensitive epilepsy
53002510000 06119		Generalised epileptiform discharges
53002610000 06117		Generalized epileptiform discharges
55012210000 06116		Motor cortex epilepsy
55012310000 06118		Jacksonian epilepsy
56141610000 06114		Localisation-related idiopathic epilepsy
56141710000 06119		Localization-related idiopathic epilepsy
56182010000 06113		Paranoid-hallucinatory epileptic psychosis
60286210000 06119		Epileptic attack

60286310000 06116		Epileptic convulsion
60286410000 06114		Epileptic fit
64730100000 6112	F2500 11	Epileptic absences
69911310000 06116		Epilepsy, not refractory
69911410000 06114		Epilepsy, not intractable
69947610000 06114		Refractory localisation-related epilepsy
69947710000 06119		Refractory localization-related epilepsy
69947810000 06116		Intractable localization-related epilepsy
69947910000 06118		Intractable localisation-related epilepsy
69974510000 06113		Refractory epilepsia partialis continua
69974610000 06110		Intractable epilepsia partialis continua
70014410000 06114		Simple partial status epilepticus
70376310000 06119		Refractory occipital lobe epilepsy
70376410000 06112		Intractable occipital lobe epilepsy
70409210000 06113		Refractory frontal lobe epilepsy
70409310000 06111		Intractable frontal lobe epilepsy
70429010000 06119		Refractory parietal lobe epilepsy
70429110000 06116		Intractable parietal lobe epilepsy
72049710000 06115		Anoxic epileptic seizure
72205310000 06114		Epilepsy assessment
72535210000 06119		Refractory generalised nonconvulsive epilepsy
72535310000 06116		Refractory generalized nonconvulsive epilepsy
72649510000 06112		Epilepsy characterised by intractable complex partial seizures
72649610000 06114		Epilepsy characterized by intractable complex partial seizures
72653810000 06118		Nonconvulsive status epilepticus
73027410000 06112		Epileptic seizure witnessed by provider of history other than subject

73068210000 06113		Refractory epilepsy
73068310000 06111		Intractable epilepsy
73622018	F25y4 00	Benign Rolandic epilepsy
73637100000 6112	F25y2 00	Localization-related(focal)(partial)idiopathic epilepsy and epileptic syndromes with seizures of localised onset
74346100000 6119	F2543 00	Limbic system epilepsy
75033610000 06117		Autosomal dominant nocturnal frontal lobe epilepsy
75124510000 06118		Generalised non-convulsive absence epilepsy
75124610000 06116		Generalized non-convulsive absence epilepsy
75124710000 06111		Refractory juvenile myoclonic epilepsy
75124810000 06114		Intractable juvenile myoclonic epilepsy
75124910000 06112		Refractory myoclonic epilepsy
75125010000 06116		Intractable myoclonic epilepsy
75125110000 06118		Postoperative status epilepticus
75125210000 06114		Post infectious grand mal epilepsy
75125810000 06113		Post-cerebrovascular accident epilepsy
75206610000 06114		Myoclonic epilepsy myopathy sensory ataxia
75206710000 06119		MEMSA - myoclonic epilepsy myopathy sensory ataxia
75206810000 06116		Spinocerebellar ataxia with epilepsy
75257710000 06118		Generalised epilepsy with febrile seizures plus
75257810000 06115		Generalized epilepsy with febrile seizures plus
75352710000 06112		EAST (Epilepsy, ataxia, sensorineural deafness, and tubulopathy) syndrome
75352810000 06110		Epilepsy, ataxia, sensorineural deafness, and tubulopathy syndrome
75580510000 06118		Progressive myoclonus epilepsy with ataxia
75580610000 06116		PRICKLE1-related progressive myoclonic epilepsy with ataxia
75580710000 06111		Progressive myoclonic epilepsy 1B
75769810000 06111		Spinal muscular atrophy with progressive myoclonic epilepsy

75770510000 06119		Progressive epilepsy with mental retardation
75770610000 06117		Northern epilepsy
76630610000 06112		Refractory idiopathic generalised epilepsy
76630710000 06117		Intractable idiopathic generalised epilepsy
76630810000 06119		Refractory idiopathic generalized epilepsy
76630910000 06116		Intractable idiopathic generalized epilepsy
77320210000 06112		Skeletal dysplasia with epilepsy and short stature syndrome
77350310000 06117		Generalised epilepsy and paroxysmal dyskinesia syndrome
77350410000 06110		Generalized epilepsy and paroxysmal dyskinesia syndrome
77509110000 06117		Female restricted epilepsy with intellectual disability syndrome
77580910000 06113		X-linked epilepsy with learning disability and behaviour disorder syndrome
77581010000 06119		X-linked epilepsy with learning disability and behavior disorder syndrome
77581310000 06110		Benign adult familial myoclonic epilepsy
77581410000 06117		BAFME - Benign adult familial myoclonic epilepsy
77581510000 06115		Autosomal dominant cortical myoclonus and epilepsy
77581610000 06118		Benign adult familial myoclonus epilepsy
77822210000 06114		X-linked Dandy-Walker malformation with intellectual disability, basal ganglia disease and seizure syndrome
77824710000 06114		X-linked intellectual disability and epilepsy with progressive joint contracture and facial dysmorphism syndrome
77866710000 06113		Sudden unexpected death in epilepsy
77912510000 06111		X-linked intellectual disability with seizure and psoriasis syndrome
78048610000 06110		Atherosclerosis, deafness, diabetes, epilepsy, nephropathy syndrome
78102810000 06113		Alopecia, psychomotor epilepsy, periodontal pyorrhoea, intellectual disability syndrome
78103010000 06112		Alopecia, psychomotor epilepsy, periodontal pyorrhea, intellectual disability syndrome
78127610000 06110		Intellectual disability, epilepsy, bulbous nose syndrome
78265610000 06110		MEHMO (mental retardation, epileptic seizures, hypogonadism and hypogenitalism, microcephaly, obesity) syndrome
78274610000 06112		Osteogenesis imperfecta, retinopathy, seizures, intellectual disability syndrome

78307610000 06119		Coeliac disease with epilepsy and cerebral calcification syndrome
78406710000 06115		Epileptic encephalopathy
78466510000 06111		Severe intellectual disability, epilepsy, anal anomaly, distal phalangeal hypoplasia syndrome
78539810000 06117		Infant epilepsy with migrant focal crisis
78577010000 06117		Epilepsy due to infectious disease of central nervous system
78580610000 06115		Pyridoxal 5-phosphate dependent epilepsy
78612810000 06116		Epilepsy due to perinatal stroke
78612910000 06118		Epilepsy due to perinatal anoxic-ischaemic brain injury
78613010000 06117		Epilepsy due to perinatal anoxic-ischemic brain injury
78613110000 06119		Epilepsy due to perinatal hypoxic ischemic encephalopathy
78613210000 06110		Epilepsy due to perinatal hypoxic ischaemic encephalopathy
78613310000 06113		Epilepsy due to cerebrovascular accident
78613410000 06115		Epilepsy due to stroke
78613510000 06118		Epilepsy due to and following traumatic brain injury
78613610000 06116		Epilepsy due to intracranial tumour
78613710000 06111		Epilepsy due to intracranial tumor
78613810000 06114		Epilepsy due to intracranial neoplasm
78636110000 06110		Epilepsy co-occurrent and due to degenerative brain disorder
78636210000 06119		Epilepsy with degenerative brain disorder
78636310000 06116		Epilepsy co-occurrent and due to mesial temporal sclerosis
78636410000 06114		Epilepsy with mesial temporal sclerosis
78636510000 06111		Epilepsy due to immune disorder
78636610000 06113		Epilepsy co-occurrent and due to demyelinating disorder
78636710000 06118		Epilepsy with demyelinating disorder
78636810000 06115		Epilepsy co-occurrent and due to dementia
78636910000 06117		Epileptic dementia

78637010000 06117		Epilepsy with dementia
78662010000 06117		X-linked spasticity, intellectual disability, epilepsy syndrome
78685310000 06112		Febrile infection related epilepsy syndrome
78685610000 06115		FIRES - fever-induced refractory epileptic encephalopathy in school-aged children
78817810000 06111		Epileptic encephalopathy with global cerebral demyelination
78821510000 06111		X-linked intellectual disability, nail dystrophy, seizures syndrome
79527410000 06112		Epilepsy, microcephaly, skeletal dysplasia syndrome
79527610000 06111		Epilepsy telangiectasia syndrome
79551810000 06111		Epilepsy of infancy with migrating focal seizures
79596710000 06118		Autism spectrum disorder, epilepsy, arthrogryposis syndrome
79763410000 06110		Refractory generalised convulsive epilepsy
79763510000 06112		Refractory generalized convulsive epilepsy
79893510000 06110		Atonic epilepsy
79927110000 06117		Refractory complex partial seizure with impairment of consciousness
80003410000 06117		Partial frontal lobe epilepsy
80072100000 6115	F250z 00	Generalised nonconvulsive epilepsy NOS
80078610000 06111		Partial occipital lobe epilepsy
80089510000 06118		Partial parietal lobe epilepsy
80174910000 06115		Primary generalised absence epilepsy
80175010000 06111		Primary generalized absence epilepsy
80214410000 06111		Intractable absence seizures
80246910000 06119		Epileptic dementia with behavioural disturbance
80247010000 06119		Epileptic dementia with behavioral disturbance
80249710000 06110		Partial epileptic seizure of parietal lobe with impairment of consciousness
80249910000 06111		Complex partial epileptic seizure of frontal lobe
80250010000 06116		Partial epileptic seizure of frontal lobe with impairment of consciousness

80250210000 06114		Complex partial epileptic seizure of occipital lobe
80250310000 06112		Partial epileptic seizure of occipital lobe with impairment of consciousness
80250510000 06117		Complex partial epileptic seizure of temporal lobe
80250610000 06115		Partial epileptic seizure of temporal lobe with impairment of consciousness
80250710000 06110		Intractable partial temporal lobe epilepsy with impairment of consciousness
80250910000 06111		Intractable complex partial parietal lobe epilepsy
80251010000 06117		Intractable partial parietal lobe epilepsy with impairment of consciousness
80251110000 06119		Intractable partial frontal lobe epilepsy with impairment of consciousness
80251210000 06110		Intractable partial occipital lobe epilepsy with impairment of consciousness
80316010000 06113		Epilepsy in mother complicating pregnancy
80316110000 06111		Epilepsy in pregnancy
80372910000 06111		Intractable simple partial epilepsy
80532610000 06111		Atypical absence epilepsy
80895510000 06118		Aphasia co-occurrent with epilepsy
80895710000 06111		Status epilepticus due to complex partial epileptic seizure
80895810000 06114		Status epilepticus due to refractory complex partial seizures
80895910000 06112		Status epilepticus due to intractable complex partial seizures
80896010000 06116		Status epilepticus due to generalised idiopathic epilepsy
80896110000 06118		Status epilepticus due to generalized idiopathic epilepsy
80896310000 06112		Status epilepticus due to intractable idiopathic generalised epilepsy
80896410000 06119		Status epilepticus due to intractable idiopathic generalized epilepsy
80896510000 06117		Status epilepticus due to refractory idiopathic generalized epilepsy
80896610000 06115		Status epilepticus due to refractory idiopathic generalised epilepsy
80896710000 06110		Status epilepticus due to refractory epilepsy
80896810000 06113		Status epilepticus due to intractable epilepsy
80896910000 06111		Status epilepticus due to refractory simple partial epilepsy

8089701000006111		Status epilepticus due to intractable simple partial epilepsy
8089721000006118		Infantile spasms co-occurrent with status epilepticus
8089731000006115		Infantile spasms with status epilepticus
8089741000006113		Refractory infantile spasms co-occurrent with status epilepticus
8089751000006110		Refractory infantile spasms with status epilepticus
8089791000006116		Status epilepticus in benign Rolandic epilepsy
8089801000006115		Rolandic seizures with status epilepticus
8191681000006117		Petit-mal epilepsy
8289851000006112		Self management of epilepsy
851121000006117		Partial epilepsy
883041000006118	F250099	Petit mal epilepsy
897431000006113	SC20099	Post traumatic epilepsy
918561000006111		Epilepsy associated problems
918591000006115		Epilepsy drug side effects
918601000006111		Nocturnal epilepsy
931711000006118	F259.00	Early infantile epileptic encephalopathy with suppression bursts
9323961000006117		Epilepsy in mother complicating childbirth
9323971000006112		Epilepsy in childbirth

Hearing Loss

medcode	readcode	desc
100850012	F591.00	Sensorineural hearing loss
100854015	F591.99	Sensorineural deafness
100856018	F591.14	Perceptive hearing loss
100857010	F591.13	Perceptive deafness
113719012	F591300	Central hearing loss
1173591000000110	F591800	Congenital prelingual deafness

1173611000000119	F597.00	Mild acquired hearing loss
1173631000000110	F598.00	Moderate acquired hearing loss
1173651000000115	F599.00	Severe acquired hearing loss
1173671000000112	F59A.00	Profound acquired hearing loss
11902971000006110		Unilateral deafness
11929311000006115		Mild acquired hearing loss
11929321000006111		Moderate acquired hearing loss
11929331000006114		Severe acquired hearing loss
11929341000006116		Profound acquired hearing loss
11929481000006118		Deafened
12012291000006113		Isolated postlingual genetic deafness
12012311000006112		Isolated prelingual genetic deafness
12027621000006116		Total visual and total hearing impairment
12027631000006118		Deaf-blind
12027651000006113		Completely deaf and blind
121849011	F591200	Neural hearing loss
121916015	F581200	Noise-induced hearing loss
1234118017	F592.00	Mixed conductive and sensorineural deafness
1234119013	F592.11	Mixed hearing loss
1234121015	F592.99	Mixed deafness
12726991000006111		Unspecified sudden hearing loss
12727231000006118		Deafness NOS
12728821000006116		Deaf mutism, NEC
141844010	F591100	Sensory hearing loss
148551000006115	F591700	Sensorineural hearing loss, unilateral with unrestricted hearing on the contralateral side
1668111000000114	F59A.11	Deafened

1780215014	1C18.0 0	Difficulty hearing with background noise
1780216010	1C19.0 0	Difficulty hearing whispers
1872761000006 118		Special education needs - hearing impairment
2079710000061 12	F5801 00	Presbyacusia
2226281000000 117	F591C 00	Moderate sensorineural hearing loss
2226301000000 116	F591E0 0	Severe sensorineural hearing loss
2226361000000 117	F591B 00	Profound sensorineural hearing loss
2226381000000 114	F591D 00	Mild sensorineural hearing loss
2264121000000 111	F5919 00	Bilateral profound sensorineural hearing loss
2264161000000 115	F591A 00	Bilateral congenital sensorineural hearing loss
2267571000000 111	Fy1..12	Deafblind
2267601000000 116	Fy1..11	Dual sensory impairment - deafblind
2410391000000 119	E2F321 1	Articulatory impairment due to conductive hearing loss
2531501000006 116		Conductive hearing loss of combined sites
253161014	1C131 00	Unilateral deafness
253163012	1C133 00	Bilateral deafness
253491010	1J2..00	Suspected deafness
2553041000006 114		Word deafness
255425013	2BL2.0 0	O/E - slightly deaf
255426014	2BL3.0 0	O/E - significantly deaf
255427017	2BL4.0 0	O/E - very deaf
255428010	2BL5.0 0	O/E - completely deaf
255433014	2BM2. 00	O/E -tune fork=conductive deaf
255434015	2BM2. 11	O/E - conductive deafness
255435019	2BM3. 11	O/E - perceptive deafness
255436018	2BM3. 00	O/E tune fork=perceptive deaf
255437010	2BM4. 00	O/E - High tone deafness

2559891000006 111		Inner ear conductive hearing loss
25785014	F59..1 1	Deafness
25788011	F59..0 0	Hearing loss
2636241000006 117		Complete deafness
2740731000006 110		Hearing impairment
2740741000006 117		Difficulty hearing
2740761000006 118		HL - Hearing loss
2740771000006 113		HI - Hearing impairment
2740811000006 113		Tympanic membrane conductive hearing loss
2773231012	Fy1..00	Combined visual and hearing impairment
2985691000006 112		NOHL - Non-organic hearing loss
2985701000006 112		Non-organic hearing loss
299203016	F5802 00	Transient ischaemic deafness
299208013	F582.0 0	Sudden hearing loss
299240012	F5900 00	Unspecified conductive hearing loss
299241011	F5901 00	Conductive hearing loss due to disorder of external ear
299242016	F5902 00	Conductive hearing loss due to disorder of tympanic membrane
299243014	F5903 00	Conductive hearing loss due to disorder of middle ear
299244015	F5904 00	Conductive hearing loss due to disorder of inner ear
299245019	F5905 00	Conductive hearing loss, bilateral
299247010	F590y0 0	Combined conductive hearing loss
299248017	F590z0 0	Conductive hearing loss NOS
299255015	F5910 00	Unspecified perceptive hearing loss
299259014	F5916 00	Sensorineural hearing loss, bilateral
299261017	F591y0 0	Combined perceptive hearing loss
299262012	F591z0 0	Perceptive hearing loss NOS
299264013	F5921 00	Mixed conductive and sensorineural hearing loss, bilateral

299265014	F593.0 0	Deaf mutism
299266010	F59y.0 0	Other specified forms of hearing loss
299550012	FyuU0 00	[X]Deaf mutism, not elsewhere classified
299551011	FyuU1 00	[X]Other specified hearing loss
3106061000006 113		Toxic deafness
313148014	P40..0 0	Ear anomalies with hearing impairment
313149018	P400.0 0	Ear anomalies with hearing impaired, unspecified
313159017	P402.0 0	Other external ear anomaly with hearing impairment
313164018	P402z0 0	Other external ear anomaly with hearing impairment NOS
313177010	P40zz0 0	Ear anomaly with hearing impaired NOS
3158591000006 119		Occupational deafness
3190881000006 112		Paradoxic hearing loss
3203211000006 111		Cortical deafness
3209511000006 111		CHL - Conductive hearing loss
3209521000006 115		CD - Conductive deafness
3243751000006 113		Bone conduction deafness
3261821000006 119		External ear conductive hearing loss
3283701000006 115		Articulatory defect secondary to hearing loss
3289101000006 117		Upper frequency deafness
3289111000006 119		High frequency hearing loss
3301531000006 113		Senile deafness
3301551000006 118		Age-related hearing loss
3425011000006 112		End organ deafness
348075014	F59z.1 1	Chronic deafness
348076010	F594.0 0	High frequency deafness
3486051000006 114		Neurosensory deafness

3486081000006118		SND - Sensorineural deafness
3486091000006115		Sensory-neural deafness
3486101000006114		Sensory-neural hearing loss
3486111000006112		SNHL - Sensorineural hearing loss
3486121000006116		PD - Perceptive deafness
3503111000006111		Middle ear conductive hearing loss
3506711000006117		Nonspeaking deaf
3527478013		Congenital conductive hearing loss
3527591011		Congenital mixed conductive and sensorineural hearing loss
3568041000006119		Sensorineural hearing loss of combined sites
3614171000006111		Central deafness
3654101000006113		Dissociative deafness
3655811000006119		Mid frequency deafness
3687041000006111		Traumatic deafness, non-occupational
3692171000006115		Air conduction deafness
3693051000006116		Nerve conduction deafness
3693061000006119		Retrocochlear hearing loss
3693771000006111		NIHL - Noise-induced hearing loss
3761211000006116		Mixed conductive AND sensorineural hearing loss
3761221000006112		Mixed type deafness
3761251000006115		MHL - Mixed hearing loss
3867531000006116		Traumatic deafness, occupational
3890851000006110		Cochlear hearing loss
3890861000006112		Inner ear hearing loss
3954651000006119		Traumatic deafness
399507018	F591500	Ototoxicity - deafness
399508011	F59z.00	Deafness NOS

400791015	P40z.0<br>0	Other and unspecified ear anomaly with hearing impaired
401826019	E2F301<br>1	Word deafness
4062711000006<br>117		Unilateral hearing loss
4062721000006<br>113		Bilateral hearing loss
4062731000006<br>111		Neonatal hearing loss
4062831000006<br>115		Congenital deafness
407075014	1C13.1<br>1	Deafness symptom
4088861000006<br>112		Hearing loss remits during vertigo attacks
411470013	F5915<br>11	Drug ototoxicity - deafness
411844019	2BL..1<br>1	O/E - deaf
4238810000061<br>17	Eu446<br>11	Psychogenic deafness
4328210000061<br>13	Eu802<br>14	[X]Word deafness
4428721000006<br>111		Birth trauma deafness
4574291000006<br>117		On examination - slightly deaf
4574311000006<br>118		On examination - significantly deaf
4574331000006<br>112		On examination - very deaf
4574351000006<br>117		On examination - completely deaf
4574411000006<br>111		O/E - conductive deafness
4574421000006<br>115		On examination - tune fork=conductive deaf
4574451000006<br>112		On examination - tune fork=perceptive deaf
4574471000006<br>119		On examination - High tone deafness
459324019	1C16.0<br>0	Deteriorating hearing
460635011	ZV412<br>00	[V]Problems with hearing
470343013	1C132<br>00	Partial deafness
477221017	F5A..0<br>0	Hearing impaired
4775211000006<br>113		Transient ischemic deafness

4775301000006119		Unilateral conductive hearing loss with unrestricted hearing on the contralateral side
4775341000006117		Unilateral sensorineural hearing loss with unrestricted hearing on the contralateral side
4775371000006113		Unilateral mixed conductive and sensorineural hearing loss with unrestricted hearing on the contralateral side
502417018	F591211	Nerve deafness
5034801000006117		Dominant sensorineural hearing loss
5034811000006119		X-linked sensorineural hearing loss
5034821000006110		Recessive sensorineural hearing loss
5034831000006113		Perinatal sensorineural hearing loss
5034841000006115		Postnatal acquired sensorineural hearing loss
512317011	F591400	Congenital sensorineural deafness
5230911000006111		Pattern of hearing loss
5577911000006115		On examination - deaf
5599091000006112		Postoperative profound sensorineural hearing loss
5599201000006111		Difficulty hearing in noise
578361000006118	F590600	Conductive hearing loss, unilateral with unrestricted hearing on the contralateral side
587171000006119	F581211	Noise induced deafness
608101000006118	1C13.00	Deafness
608121000006111	SJ15.12	Deafness - traumatic - NOS
622201000000110	F596.00	Maternally inherited deafness
7005171000006114		Difficulty hearing high frequency sounds
700951000006112	F592000	Mixed conductive and sensorineural hearing loss, unilateral with unrestricted hearing on the contralateral side
7031801000006119		Difficulty hearing speech in large group setting
7038351000006118		Difficulty hearing normal speech tones
7046801000006112		Unilateral neural hearing loss
7052361000006119		Unilateral sensory hearing loss
7076961000006116		Bilateral central hearing loss

7078511000006111		Asymmetrical hearing loss
7078521000006115		Asymmetric hearing loss
7089001000006115		Autoimmune sensorineural hearing loss
7095141000006116		Asymmetrical sensorineural hearing loss
7095151000006119		Asymmetric sensorineural hearing loss
70962017	F595.00	Low frequency deafness
7129341000006117		Bilateral neural hearing loss
7129481000006119		Bilateral sensory hearing loss
7174891000006117		Deafblindness
7254151000006115		Speech and language developmental delay due to hearing loss
7254161000006118		Speech, language developmental delay from hearing loss
7265651000006117		Unilateral conductive hearing loss
733941000006114	F591.12	Low frequency deafness
73470015	F590.00	Conductive hearing loss
73473018	F590.11	Conductive deafness
7480421000006118		History of hearing loss
7487941000006119		Deafness of right ear
7487951000006117		Deafness of left ear
7487961000006115		Hearing loss of right ear
7487971000006110		Hearing loss of left ear
7493431000006112		Acquired sensorineural hearing loss
7510621000006112		Acquired deaf mutism
7510631000006110		Congenital deaf mutism
7535361000006110		Congenital sensorineural hearing loss
7558911000006118		Congenital deafness with inner ear agenesis, microtia, and microdontia
7728971000006112		Sudden sensorineural hearing loss

7815121000006 111		Acquired hearing loss
7815131000006 114		Acquired hearing impairment
7832181000006 110		Thickened earlobe with conductive deafness syndrome
7844651000006 118		Neuropathy with hearing impairment
7858791000006 113		Sudden idiopathic hearing loss
7976771000006 115		Severe hearing loss
8009061000006 113		Mild to moderate hearing loss
80699013	E2F320 0	Articulatory defect due to conductive hearing loss
8094261000006 115		Registered deaf
8094571000006 113		Registered hearing impaired
8225781000006 118		Deaf and blind
8229810000061 12	F591.1 1	High frequency deafness
8306171000006 110		Profound BSNHL (bilateral sensorineural hearing loss)
8317121000006 110		Bilateral severe sensorineural hearing loss
8317131000006 113		Severe BSNHL (bilateral sensorineural hearing loss)
8317141000006 115		Bilateral moderate sensorineural hearing loss
8317151000006 118		Moderate BSNHL (bilateral sensorineural hearing loss)
8317161000006 116		Bilateral mild sensorineural hearing loss
8317171000006 111		Mild BSNHL (bilateral sensorineural hearing loss)
8838310000061 17	F582.9 9	Sudden hearing loss NOS
8838910000061 18	F593.9 9	Deaf mutism NOS
8839010000061 19	F59z.9 9	Hearing loss NOS
9152110000061 18		Difficulty hearing whispers
9186810000061 19		Deafness
9187010000061 16		Unilateral deafness
9187110000061 18		Partial deafness

918721000006114		Bilateral deafness
960271000006118		Difficulty hearing normal speech tones
9700951000006116		Perception of hearing loss
981241000006119		Difficulty hearing speech in large group settings
981251000006117		Difficulty hearing high frequency sounds
982621000006118		Hearing impaired
989081000006119	F59..99	Hearing loss NOS

Heart Failure

medcode	readcode	desc
12490061000006118		Admit heart failure emergency
139475013	G58..00	Heart failure
139482012	G58..11	Cardiac failure
141306010	G581.00	Left ventricular failure
147247018	G580100	Chronic congestive heart failure
1488591011	1J60.00	Suspected heart failure
1488804017	1O1..00	Heart failure confirmed
1495417010	G581.11	Cardiac asthma
1539381000006117		Heart failure lifestyle plan commenced
1576321000006113		Cause of Death- Congestive Cardiac Failure
1647701000000118	G583.00	Heart failure with normal ejection fraction
1661371000000112	G583.11	HFNEF - heart failure with normal ejection fraction
18472010	G580000	Acute congestive heart failure
206703015	G580.12	Right heart failure
2227501000000110	G583.12	Heart failure with preserved ejection fraction
223981000000118	G58z.12	Cardiac failure NOS
2504331000006114		Acute left-sided heart failure
2504341000006116		Acute left heart failure
251680018	14A6.00	H/O: heart failure
2549208013	8H2S.00	Emergency hospital admission for heart failure
2580181000006118		Heart failure due to prosthesis
2581861000006110		Hypertensive heart disease with congestive heart failure
2585431000006117		Chronic left-sided congestive heart failure
2660881000006116		High output heart failure
2664351000006113		Chronic right-sided heart failure
2675255018	G580400	Congestive heart failure due to valvular disease
2908651000006113		Low output heart failure
300179017	G580200	Decompensated cardiac failure
300180019	G580300	Compensated cardiac failure
300190010	G581000	Acute left ventricular failure
300217019	G5y4z00	Post cardiac operation heart failure NOS
305601017	L09y200	Cardiac failure following abortive pregnancy

316833010	Q48y100	Congenital cardiac failure
3182541000006117		Congestive cardiac failure
3182551000006115		CCF - Congestive cardiac failure
3182561000006118		CHF - Congestive heart failure
3213831000006118		Right heart failure secondary to left heart failure
3244761000006113		Hypertensive heart failure
3283871000006117		Chronic heart failure
3288811000006119		Miscarriage with cardiac arrest and/or cardiac failure
3402251000006117		Cardiac failure after obstetrical surgery AND/OR other procedure including delivery
3488571000006114		Heart failure following cardiac surgery
3489281000006119		Hypertensive heart disease without congestive heart failure
350484012	SP11111	Heart failure as a complication of care
3589241000006116		Chronic right-sided congestive heart failure
3718701000006116		Acute left-sided congestive heart failure
3768511000006114		Benign hypertensive heart disease without congestive heart failure
3809001000006112		Acute right-sided congestive heart failure
3842421000006111		Congestive rheumatic heart failure
3852501000006115		Malignant hypertensive heart disease with congestive heart failure
3855411000006110		Right heart failure due to disorder of lung
3855421000006119		Right heart failure due to pulmonary disease
3868341000006118		HF - Heart failure
3886041000006118		Left heart failure
3886061000006119		Left-sided heart failure
395772015	G58z.00	Heart failure NOS
3960001000006113		Heart failure during AND/OR resulting from a procedure
3974561000006115		Pleural effusion due to congestive heart failure
411506018	G581.13	Impaired left ventricular function
412678013	Q490.00	Neonatal cardiac failure
4193361000006119		Chronic left-sided heart failure
453099015	14AM.00	H/O: Heart failure in last year
4540521000006111		History of heart failure
490972013	G580.13	Right ventricular failure
493287011	G580.11	Congestive cardiac failure
504901000006118	G211100	Benign hypertensive heart disease with congestive cardiac failure
510016018	G580.14	Biventricular congestive heart failure
5586371000006119		Cardiac failure developing in the perinatal period
5990971000006113		History of heart failure in last year
6043761000006113		Refractory heart failure
6204381000006117		Acute right-sided heart failure
6679051000006119		Red half-moon nail in congestive heart failure
6895651000006113		Emergency hospital admission for heart failure
6914191000006115		Systolic heart failure
6919191000006119		Diastolic heart failure
7025691000006110		Decompensated chronic heart failure
7052811000006113		Right heart failure due to pulmonary hypertension
7056281000006118		Congestive heart failure due to left ventricular systolic dysfunction
70653017	G580.00	Congestive heart failure
7250571000006113		Chronic systolic heart failure

7251291000006119		Chronic diastolic heart failure
7274561000006113		Acute on chronic systolic heart failure
7274571000006118		Acute systolic heart failure
7275951000006115		Acute diastolic heart failure
7275961000006118		Acute on chronic diastolic heart failure
728671000006119	G210100	Malignant hypertensive heart disease with congestive cardiac failure
728681000006116	G210000	Malignant hypertensive heart disease without congestive heart failure
72934016	G1yz100	Rheumatic left ventricular failure
7321121000006119		Heart failure with preserved ejection fraction
7400171000006110		Foetal heart failure
7400181000006113		Fetal heart failure
7400191000006111		Foetal heart failure with myocardial hypertrophy
7400201000006114		Fetal heart failure with myocardial hypertrophy
7400221000006116		Fetal heart failure with redistribution of cardiac output
741701000006114	G232.00	Hypertensive heart and renal disease with (congestive) heart failure
7475721000006114		Heart failure due to end stage congenital heart disease
7497261000006113		High risk of heart failure, stage A
7497271000006118		High risk of heart failure, stage B
7499321000006117		Induced termination of pregnancy complicated by cardiac failure
7500101000006113		Diastolic heart failure stage A
7500111000006111		Systolic heart failure stage A
7507301000006110		Acute exacerbation of chronic congestive heart failure
7507311000006113		Acute on chronic congestive heart failure
7510341000006110		Symptomatic congestive heart failure
7573171000006116		Heart failure with reduced ejection fraction
7573181000006118		Heart failure with reduced ejection fraction due to coronary artery disease
7573191000006115		Heart failure with reduced ejection fraction due to myocarditis
7573201000006117		Heart failure with reduced ejection fraction due to cardiomyopathy
7573211000006119		Heart failure with reduced ejection fraction due to heart valve disease
7588221000006110		Fetal heart failure due to extracardiac disease
7588231000006113		Foetal heart failure due to extracardiac disease
7763221000006118		Congestive heart failure stage B
7838091000006110		Neonatal cardiac failure due to decreased left ventricular output
7857711000006119		Neonatal cardiac failure due to pulmonary overperfusion
789941000006117	G234.00	Hypertensive heart and renal disease with both (congestive) heart failure and renal failure
8001631000006110		Hypertensive heart AND chronic kidney disease with congestive heart failure
8011111000006111		Congestive heart failure with right heart failure
8021211000006111		Congestive heart failure stage D
8021221000006115		Congestive heart failure stage C
8022711000006111		Congestive heart failure as early postoperative complication
8030311000006111		Exacerbation of congestive heart failure
8031661000006114		Congestive heart failure due to cardiomyopathy
8037891000006112		Systolic heart failure stage D
8037901000006111		Systolic heart failure, stage D
8037911000006114		Systolic heart failure stage C
8037921000006118		Systolic heart failure, stage C

8037931000006115		Systolic heart failure stage B
8037941000006113		Diastolic heart failure stage D
8037951000006110		Diastolic heart failure, stage D
8037961000006112		Diastolic heart failure stage C
8037971000006117		Diastolic heart failure, stage C
8037981000006119		Diastolic heart failure stage B
8048051000006114		Acute combined systolic and diastolic heart failure
8048061000006111		Chronic combined systolic and diastolic heart failure
8048071000006116		Acute on chronic combined systolic and diastolic heart failure
905391000006119		[RFC] Cardiac failure
94251011	G582.00	Acute heart failure
9833971000006112		Congestive heart failure stage C due to ischaemic cardiomyopathy
9833981000006110		Congestive heart failure stage C due to Ischemic cardiomyopathy
9833991000006113		Congestive heart failure stage B due to ischaemic cardiomyopathy
9834001000006116		Congestive heart failure stage B due to ischemic cardiomyopathy
9834021000006114		Systolic heart failure stage B due to ischaemic cardiomyopathy
9834031000006112		Systolic heart failure stage B due to ischemic cardiomyopathy
9834051000006117		Systolic heart failure stage C due to ischaemic cardiomyopathy
9834061000006115		Systolic heart failure stage C due to ischemic cardiomyopathy

Hypertension

medcode	readcode	desc
1011861000006110		adverse reaction to antihypertensive drugs
1011871000006115		adverse reaction to vasodilator antihypertensive drugs
1011881000006117		adverse reaction to centrally acting antihypertensive drugs
108730018		malignant hypertensive renal disease
11777411000006113		renal disease due to hypertension
12487991000006113		hypertensive treatm.changed
12488211000006112		hypertension treatm. started
12488221000006116		hypertension treatm.stopped
12496011		hypertensive retinopathy
12726231000006115		adverse reaction to other antihypertensives
12726241000006113		adverse reaction to antihypertensives nos
131046010	G200.00	Malignant essential hypertension
1409014		benign hypertensive renal disease
146259010		secondary pulmonary hypertension
151161000006115	G240.00	Malignant secondary hypertension
1529013		blind hypertensive eye
158241000006117	G24z.00	Secondary hypertension NOS
1706551000000114		hypertension 9 month review
1749291000006113		thromboembolic pulmonary hypertension
1780252014		hypertension treatment refused
1780253016		hypertension six month review
1780318013		moderate hypertension control
1780319017		hypertension annual review
1806071000006118		stage 1 hypertension
1806081000006115		stage 2 hypertension

1806141000006113		severe hypertension
1823901000006112		hypertension confirmed
1846941000006119		stage 1 hypertension (nice - nat ins for hth clin excl 2011)
1846961000006115		severe hypertension (nat inst for health clinical ex 2011)
1846991000006111		stage 2 hypertension (nice - nat ins for hth clin excl 2011)
1908711000006115		stage 1 hyperten (nice 2011) without evidnce end organ damge
1908721000006111		stage 1 hyperten (nice 2011) with evidnce end organ damge
19411000006110		other pre-exist hypertension in preg/childb/puerp + p/n comp
19421000006119		other pre-exist hypertension in preg/childb/puerp-not deliv
19431000006116		other pre-existing hypertension in preg/childb/puerp - deliv
19441000006114		other pre-existing hypertension in preg/childb/puerp nos
19451000006111		other pre-existing hypertension in preg/childb/puerp unspec
1992471000006116		vte risk assessment - uncontrolled systolic hypertension
2011000033113		antihypertensive drugs
2115801000000110		hypertension self-management plan review
2117971000000119		hypertension self-management plan agreed
212961000006118		pre-eclampsia or eclampsia + pre-existing hypertension nos
213081000006118		pre-exist 2ndry hypertens comp preg childbth and puerperium
213111000006112		pre-exist hypertension compl preg childbirth and puerperium
2159168015		patient on maximal tolerated antihypertensive therapy
2193021000000110		severe hypertension
2193031000000112		stage 1 hypertension
2193971000000110		hypertension resistant to drug therapy
2297871000000111		pulmonary arterial hypertension drugs band 1
2297911000000113		pulmonary arterial hypertension drugs band 2
2297951000000112		pulmonary arterial hypertension drugs band 3
2297991000000116		pulmonary arterial hypertension drugs band 4
2474335018		hypertension clinical management plan
2478822013	G241000	Secondary benign renovascular hypertension
251674014		h/o: hypertension
264471012		good hypertension control
264472017		poor hypertension control
264473010		hypertension:follow-up default
264485019		hypertension treatm. started
264486018		hypertensive treatm.changed
264487010		hypertension treatm.stopped
2645973019		chronic peripheral venous hypertension
2681651000006116		pulmonary hypertensive arterial disease
2681661000006119		hypertensive pulmonary arterial disease
28311000033116		vasodilator antihypertensive drugs
28411000033112		centrally acting antihypertensive drugs
299676019	G240000	Secondary malignant renovascular hypertension
299677011	G240z00	Secondary malignant hypertension NOS
299678018	G241.00	Secondary benign hypertension
299680012	G241z00	Benign secondary hypertension
299681011	G244.00	Hypertension secondary to endocrine disorders

299682016	G24zz00	Secondary hypertension NOS
299683014	G24z000	Renovascular hypertension
299684015	G24z100	Hypertension secondary to drug
299686018	G2y..00	Other specified hypertensive disease
299687010	G2z..00	Hypertensive disorder
300869017	Gyu2.00	[X]Hypertensive diseases
300870016	Gyu2000	[X]Other secondary hypertension
300871017		[x]hypertension secondary to other renal disorders
305764016		other pre-existing hypertension in preg/childbirth/puerp
305818015		pre-eclampsia or eclampsia with pre-existing hypertension
3117411000006118		high blood pressure
3117421000006114		hypertensive vascular disease
3117431000006112		hypertensive vascular degeneration
3117451000006117		bp - high blood pressure
3117461000006115		systemic arterial hypertension
3117471000006110		hbp - high blood pressure
3117481000006113		ht - hypertension
3117491000006111		high blood pressure disorder
3117501000006115		bp+ - hypertension
3117511000006117		htn - hypertension
3117521000006113		hypertensive disorder, systemic arterial
3119661000006114		hypertensive nephropathy
3135013	G201.00	Benign essential hypertension
324338010		other hypertensive agent poisoning
324342013		hypertensive agent poisoning nos
331291010		adverse reaction to other antihypertensives
331302012		adverse reaction to antihypertensives nos
3468501000006117		systemic primary arterial hypertension
350517010		thromboembolic pulmonary hypertension
350601000000116		high cost hypertension drugs
351341000000118		high cost hypertension drugs nos
351361000000117		other specified high cost hypertension drugs
351381000000114		primary pulmonary hypertension drugs band 1
351401000000114		primary pulmonary hypertension drugs band 2
351421000000117		primary pulmonary hypertension drugs band 3
351441000000112		primary pulmonary hypertension drugs band 4
351481000006111		[x] adverse reaction to antihypertensives nos
356121000006111		[x] adverse reaction to other antihypertensives
3642801000006112		malignant hypertension
3654631000006113		pulmonary hypertension
3654641000006115		pht - pulmonary hypertension
3693711000006119		benign secondary renovascular hypertension
3784371000006115		accelerated essential hypertension
3898601000006112		pre-existing hypertension in obstetric context
3898611000006110		pre-existing hypertension complicating pregnancy
3950631000006119		accelerated secondary hypertension
395751018	G20z.00	Essential hypertension NOS
395753015		hypertensive renal disease nos

39871000006113		oth pre-exist hypert in preg/childb/puerp -del with p/n comp
404471000006113		[x]oth antihyperten drug caus advers eff in therap use, nec
4199551000006110		antihypertensive poisoning
4199561000006112		poisoning caused by antihypertensive agent
422681000006111		[x]poisoning by other antihypertensive drugs, nec
4356081000006115		hypertension due to renovascular disease
43850011		primary pulmonary hypertension
443764015		on treatment for hypertension
451424017		antihypertensive therapy
4540441000006117		history of hypertension
4634851000006119		treatment for hypertension started
4634871000006112		hypertensive treatment changed
4634891000006113		treatment for hypertension stopped
47076011		renal hypertension
4775791000006113		malignant secondary renovascular hypertension
4775831000006118		hypertension secondary to endocrine disorder
4775861000006110		hypertension caused by drug
5057801000006113		chronic thromboembolic pulmonary hypertension
523801000006119		bp - hypertensive disease
530161000000111		trial reduction of antihypertensive therapy
530221000000112		trial withdrawal of antihypertensive therapy
53452019	G24..00	Secondary hypertension
535591000000118		trial withdrawal of antihypertensive therapy declined
535651000000113		trial reduction of antihypertensive therapy declined
64168014	G2...00	Hypertensive disease
64172013	G20..11	Elevated blood pressure
64282015		hypertensive renal disease
790031000006119		hypertension annual review
790121000006116	G20z.11	Hypertension
80224019	G203.00	Diastolic hypertension
8286321000006117		resistant hypertension
8286581000006114		stage 2 hypertension
84111015		hypertensive encephalopathy
84112010		hypertensive crisis
884121000006111		malignant hypertension
887811000006117		preg.+ pre-existing hypertensn
905451000006118		[rfc] pulmonary embolism/pulmonary hypertension
909441000006118		[rfc] hypertension
93494011	G202.00	Systolic hypertension
940001000006110		hypertension medication review
99042012	G20..00	Essential hypertension
99047018		primary hypertension

IBS

medcode	readcode	desc

1495560015	Eu4532 4	[X]Psychogenic IBS
18666015	J521.11	Irritable bowel syndrome
1927491000006 111	J52120 0	Irritable bowel syndrome characterised by alternating bowel habit
2670911000006 112		IBS - Irritable bowel syndrome
2791650019	J52110 0	Irritable bowel syndrome characterised by constipation
3030710000001 18	14CF.00	History of irritable bowel syndrome
303172010	J52100 0	Irritable bowel syndrome with diarrhoea
4786241000006 112		Irritable bowel syndrome with diarrhea
5086151000006 112		Irritable bowel syndrome variant of childhood
5086161000006 114		Irritable bowel syndrome variant of childhood with diarrhoea
5086171000006 119		Irritable bowel syndrome variant of childhood with diarrhea
5086181000006 116		Irritable bowel syndrome variant of childhood with constipation
7238961000006 111		Irritable bowel syndrome characterized by alternating bowel habit
7240101000006 111		Constipation predominant irritable bowel syndrome
7240111000006 114		Irritable bowel syndrome characterized by constipation
7423510000061 19	J521.00	Irritable colon - Irritable bowel syndrome
8863610000061 14	J521.99	Irritable bowel - IBS
9061810000061 10		[RFC] Irritable bowel syndrome (IBS)
9326771000006 119		Merional 150unit powder and solvent for solution for injection vials (IBSA Farmaceutici Italia Srl)

IHD

medcodeid	readcode	desc
116992017		acute subendocardial infarction
1218860015		acute papillary muscle infarction
1234005010		acute inferoposterior infarction
1234306015		acute septal infarction
1780491019		acute st segment elevation myocardial infarction
1780501013		acute non-st segment elevation myocardial infarction
1786197015		coronary thrombosis
1786198013		thrombosis - coronary
219521000000119		attack - heart
219531000000117		mi - acute myocardial infarction
2663441000006117		acute infarction of papillary muscle
2855301000006112		myocardial infarction

2855311000006110		infarction of heart
2855321000006119		cardiac infarction
2855341000006114		mi - myocardial infarction
2855351000006111		myocardial infarct
299707016		other specified anterior myocardial infarction
299708014		acute anteroapical infarction
299709018		anterior myocardial infarction nos
299710011		posterior myocardial infarction nos
299711010		lateral myocardial infarction nos
299712015		true posterior myocardial infarction
299714019		inferior myocardial infarction nos
299718016		other acute myocardial infarction
299719012		acute atrial infarction
299720018		other acute myocardial infarction nos
299721019		acute myocardial infarction nos
3343471000006116		acute anteroapical myocardial infarction
3381601000006117		acute myocardial infarction of anterior wall
3427201000006111		ami - acute myocardial infarction
3452181000006112		acute myocardial infarction of lateral wall
3565871000006113		acute myocardial infarction of inferolateral wall
362461000006119		[x]acute transmural myocardial infarction of unspecif site
3641641000006116		acute myocardial infarction of anterolateral wall
3645351000006114		acute nontransmural infarction
3699911000006119		acute myocardial infarction of diaphragmatic wall
3699921000006110		acute inferior myocardial infarction
37443015		heart attack
3745741000006117		acute myocardial infarction of inferoposterior wall
3784911000006111		acute myocardial infarction of septum
3784921000006115		acute myocardial infarction of septum alone
447324018		acute q-wave infarct
450322013		acute non-q wave infarction
455641000006112		acute anterolateral infarction
455651000006114		acute anteroseptal infarction
457531000006110		acute inferolateral infarction
460681000006116		acute transmural myocardial infarction of unspecif site
4775881000006117		true posterior wall infarction
4775891000006119		acute myocardial infarction of atrium
5935321000006111		acute q wave myocardial infarction
6348651000006112		myocardial infarction with complication
6601131000006115		ct - coronary thrombosis
6651221000006117		stemi - st elevation myocardial infarction
6651391000006114		nstemi - non-st segment elevation mi
884141000006116		coronary thrombosis
884151000006119		myocardial infarction
905351000006113		[rfc] myocardial infarction (mi)
932081000006118		first myocardial infarction
94884017		acute myocardial infarction
967931000006114		acute posterolateral myocardial infarction

1488382011		acute coronary syndrome
257153100006110		intermediate coronary syndrome
257156100006118		pre-infarction syndrome
257157100006113		preinfarction angina
299741012		preinfarction syndrome nos
299745015		acute coronary insufficiency
482811000006113		angina at rest
498328016		angina at rest
654611100006118		acs - acute coronary syndrome
72571000006115		unstable angina
7844010		preinfarction syndrome
7845011		unstable angina
7847015		crescendo angina
854491000006113		unstable angina
931961000006117		acute coronary syndrome
2632221000006111		induced cardiac arrest

SMI

medcode	readcode	desc
10122017		brief reactive psychosis
105029017		paranoid schizophrenia in remission
107878010		paranoid schizophrenia
114616017		chronic catatonic schizophrenia
11912191000006111		[v]personal history of schizophrenia
11918351000006117		[x] manic-depressive psychosis, depressed type without psychotic symptoms
11921141000006115		[x]recurrent severe episodes/reactive depressive psychosis
119579013	E141.00	Disintegrative psychosis
12005681000006116		robotic reusable bipolar electrosurgical instrument
12005691000006118		robotic single-use bipolar electrosurgical instrument
12059201000006117		bipolar disorder, most recent episode depression
12059211000006119		bipolar disorder, most recent episode manic
12059221000006110		bipolar affective disorder, most recent episode mixed
12059241000006115		bipolar i disorder, most recent episode manic
12059251000006118		bipolar i disorder, most recent episode depression
1219653018		atypical schizophrenia
1227584015		[v]personal history of manic-depressive psychosis
1234861017		chronic schizophrenic

1235866100000 6114		x-linked intellectual disability-psychosis-macroorchidism syndrome
1236110000061 10		subchronic schizo-affective schizophrenia
1246336100000 6113		bipolar affective disorder, currently depressed, severe, with psychosis
1248045100000 6112		[x]schizophrenia, schizotypal and delusional disorders
1248199100000 6119		acute exacerbation of subchronic hebephrenic schizophrenia
1248200100000 6117		acute exacerbation of subchronic schizophrenia
1271759100000 6112		[x] (other nonorganic psychotic disorders) or (chronic hallucinatory psychosis)
1273672100000 6110		fep - first episode psychosis
1275911100000 6111		[x] chronic hallucinatory psychosis
1275912100000 6115		[x]chronic hallucinatory psychosis
132503015		subchronic paranoid schizophrenia
1551410000061 16		schizoaffective schizophrenia
1551510000061 19		schizoaffective schizophrenia in remission
1551610000061 17		schizo-affective schizophrenia nos
1552810000061 19		schizotypal personality
1667581000000 114		[x]paranoid state in remission
1667591000000 111		[x]nonorganic psychosis in remission
1690610000061 12		restzustand - schizophrenia
1715191000006 112	Eu328 00	Severe major depression with psychotic features
1755901000006 112		[x]single major depressive episode, severe, with psychosis, psychosis in remission
1755911000006 110		[x]recurrent major depressive episodes, severe, with psychosis, psychosis in remission
1780205015		h/o: manic depressive disorder
1785851000006 110		bipolar i disorder
1785861000006 112		bipolar ii disorder
1785871000006 117		[x]bipolar ii disorder
178723016		catatonic schizophrenia in remission
1828610000061 10		recurrent manic episodes, in partial remission
1828710000061 15		recurrent manic episodes, severe

1974351000006118		paranoid schizophrenia, continuous
1974391000006112		paranoid schizophrenia, episodic with progressive deficit
1974431000006118		paranoid schizophrenia, episodic with stable deficit
1974451000006113		paranoid schizophrenia, episodic remittent
1974481000006117		paranoid schizophrenia, incomplete remission
1974511000006113		paranoid schizophrenia, complete remission
1974541000006112		paranoid schizophrenia, course uncertain, period of observation too short
1974601000006111		hebephrenic schizophrenia, continuous
1974611000006114		hebephrenic schizophrenia, episodic with progressive deficit
1974621000006118		hebephrenic schizophrenia, episodic with stable deficit
1974641000006113		hebephrenic schizophrenia, episodic remittent
1974671000006117		hebephrenic schizophrenia, incomplete remission
1974691000006116		hebephrenic schizophrenia, complete remission
1974721000006114		hebephrenic schizophrenia, course uncertain, period of observation too short
1974751000006117		catatonic schizophrenia, continuous
1974781000006113		catatonic schizophrenia, episodic with progressive deficit
1974801000006112		catatonic schizophrenia, episodic with stable deficit
1974831000006116		catatonic schizophrenia, episodic remittent
1974861000006113		catatonic schizophrenia, incomplete remission
1974891000006117		catatonic schizophrenia, complete remission
1974921000006111		catatonic schizophrenia, course uncertain, period of observation too short
1974951000006119		undifferentiated schizophrenia, continuous
1974971000006112		undifferentiated schizophrenia, episodic with progressive deficit
1975001000006118		undifferentiated schizophrenia, episodic with stable deficit
1975021000006111		undifferentiated schizophrenia, episodic remittent
1975051000006119		undifferentiated schizophrenia, incomplete remission

1975081000006110		undifferentiated schizophrenia, complete remission
1975161000006118		undifferentiated schizophrenia, course uncertain, period of observation too short
1975191000006114		post-schizophrenic depression, continuous
1975211000006110		post-schizophrenic depression, episodic with progressive deficit
1975231000006116		post-schizophrenic depression, episodic with stable deficit
1975261000006113		post-schizophrenic depression, episodic remittent
1975281000006115		post-schizophrenic depression, incomplete remission
1975301000006116		post-schizophrenic depression, complete remission
1975321000006114		post-schizophrenic depression, course uncertain, period of observation too short
1975351000006117		residual schizophrenia, continuous
1975381000006113		residual schizophrenia, episodic with progressive deficit
1975401000006113		residual schizophrenia, episodic with stable deficit
1975431000006117		residual schizophrenia, episodic remittent
1975441000006110		residual schizophrenia, incomplete remission
1975471000006119		residual schizophrenia, complete remission
1975481000006116		residual schizophrenia, course uncertain, period of observation too short
1975491000006118		simple schizophrenia, continuous
1975501000006114		simple schizophrenia, episodic with progressive deficit
1975521000006116		simple schizophrenia, episodic with stable deficit
1975551000006113		simple schizophrenia, episodic remittent
1975571000006115		simple schizophrenia, incomplete remission
1975601000006110		simple schizophrenia, complete remission
1975641000006112		simple schizophrenia, course uncertain, period of observation too short
1975681000006118		acute polymorphic psychot disord with symp of schizophren, without associated acute stress
1975691000006115		acute polymorphic psychot disord with symp of schizophren, with associated acute stress
1975711000006117		acute schizophrenia-like psychotic disorder, without associated acute stress

1975731000006 111		acute schizophrenia-like psychotic disorder, with associated acute stress
1975751000006 116		other schizophrenia, continuous
1975771000006 114		other schizophrenia, episodic with progressive deficit
1975801000006 111		other schizophrenia, episodic with stable deficit
1975831000006 115		other schizophrenia, episodic remittent
1975841000006 113		other schizophrenia, incomplete remission
1975881000006 119		other schizophrenia, complete remission
1975911000006 119		other schizophrenia, course uncertain, period of observation too short
1975931000006 113		bipolar affect disorder cur epi mild or moderate depressn, without somatic syndrome
1975941000006 115		bipolar affect disorder cur epi mild or moderate depressn, with somatic syndrome
1976001000006 113		schizophrenia, unspecified, continuous
1976061000006 114		schizophrenia, unspecified, episodic with progressive deficit
1976101000006 112		schizophrenia, unspecified, episodic with stable deficit
1976121000006 119		schizophrenia, unspecified, episodic remittent
1976141000006 114		schizophrenia, unspecified, incomplete remission
1976151000006 111		schizophrenia, unspecified, complete remission
1976171000006 118		schizophrenia, unspecified, course uncertain, period of observation too short
1976881000006 112		paranoid schizophrenia, other
1976891000006 110		hebephrenic schizophrenia, other
1976901000006 114		catatonic schizophrenia, other
1976911000006 112		undifferentiated schizophrenia, other
1976921000006 116		post-schizophrenic depression, other
1976931000006 118		residual schizophrenia, other
1976941000006 111		simple schizophrenia, other
1976951000006 113		other schizophrenia, other
1976961000006 110		schizophrenia, unspecified, other

215691000006112	286..11	Poor insight into psychotic condition
2157096015	1S42.00	Manic mood
215841000000114	Eu22000	Delusional disorder
215851000000112		[x]paranoid psychosis
215871000000115		[x]paranoia
223601000000119		manic psychosis
223611000000117		non-organic psychosis
2501011000006119		severe bipolar i disorder, most recent episode manic, without psychotic features
251628010		h/o: schizophrenia
2517511000006119		chronic bipolar ii disorder, most recent episode major depressive
2522281000006115		bipolar i disorder, single manic episode with postpartum onset
25461000006115		other nonorganic psychoses
2555231000006113		bipolar i disorder, single manic episode, in full remission
2555241000006115		bipolar 1 disorder, single manic episode, full remission
2577941000006118		schizophrenia, in remission
2590511000006116		bipolar disorder, partial remission
2601091000006116		cocaine induced psychosis with hallucinations
2611981000006118		subchronic undifferentiated schizophrenia with acute exacerbations
2648981000006119		bipolar i disorder, single manic episode
2648991000006116		bipolar 1 disorder, single manic episode
2672961000006111		severe mixed bipolar i disorder with psychotic features, mood-incongruent
2672971000006116		severe bipolar i disorder, most recent episode mixed, with psychotic features, mood-incongruent
2674671000006119		severe mixed bipolar i disorder with psychotic features
2674681000006116		severe bipolar i disorder, most recent episode mixed, with psychotic features
2705561000006112		chronic disorganised schizophrenia
2705571000006117		chronic disorganized schizophrenia
2705961000006118		severe bipolar ii disorder, most recent episode major depressive, in full remission

2708271000006 111		factitious psychosis
2715201000006 111		severe bipolar i disorder, single manic episode with psychotic features, mood-congruent
2717981000006 118		mdi - manic-depressive illness
2724061000006 113		erotomanic delusion disorder
2724221000000 116		late onset substance-induced psychosis
2724261000000 112		late onset cocaine-induced psychosis
2724281000000 115		late onset lsd (lysergic acid diethylamide)-induced psychosis
2724301000000 119		late onset cannabinoid-induced psychosis
2724321000000 111		late onset amphetamine-induced psychosis
2726261000006 113		subchronic disorganised schizophrenia with acute exacerbations
2726271000006 118		subchronic disorganized schizophrenia with acute exacerbations
2729641000006 117		severe bipolar i disorder, single manic episode without psychotic features
2729691000000 119		cataleptic schizophrenia
2758341000006 117		bipolar ii disorder, most recent episode major depressive
2761681000006 119		bipolar i disorder, most recent episode mixed
2782051000006 112		bipolar i disorder, most recent episode manic with catatonic features
2806351000006 114		severe bipolar ii disorder, most recent episode major depressive with psychotic features, mood-congruent
2808601000006 117		opioid induced psychosis with hallucinations
2823981000006 116		opioid psychosis with delusions
2833511000006 116		severe bipolar ii disorder, most recent episode major depressive with psychotic features, mood-incongruent
2848341000006 118		bipolar i disorder, most recent episode depressed with catatonic features
2852381000006 111		depressed bipolar i disorder in full remission
2852391000006 114		bipolar i disorder, most recent episode depressed, in full remission
2857081000006 110		bipolar ii disorder, most recent episode major depressive with catatonic features
28758018		subchronic schizophrenia
2885171000006 112		bipolaris
2885181000006 110		genus bipolaris

2885191000006113		bipolaris species
2919421000006112		severe depressed bipolar i disorder with psychotic features, mood-incongruent
2919431000006110		severe bipolar i disorder, most recent episode depressed with psychotic features, mood-incongruent
2924901000006115		severe bipolar disorder with psychotic features, mood-incongruent
2927881000006111		cannabis psychosis with hallucinations
2938951000006113		subchronic disorganised schizophrenia
2938961000006110		subchronic disorganized schizophrenia
294660013	E00z.00	Senile or presenile psychoses NOS
294714016		postpartum psychosis
294724012		non-organic psychoses
294725013		schizophrenic disorders
294726014		schizophrenia simplex
294727017		simple schizophrenia
294728010		unspecified schizophrenia
294730012		acute exacerbation of subchronic schizophrenia
294731011		acute exacerbation of chronic schizophrenia
294734015		simple schizophrenia nos
294735019		unspecified hebephrenic schizophrenia
294736018		subchronic hebephrenic schizophrenia
294737010		chronic hebephrenic schizophrenia
294738017		acute exacerbation of subchronic hebephrenic schizophrenia
294739013		acute exacerbation of chronic hebephrenic schizophrenia
294740010		hebephrenic schizophrenia in remission
294741014		hebephrenic schizophrenia nos
294742019		catatonic schizophrenia
294743012		[x]schizophrenic flexibilatis cerea
294744018		unspecified catatonic schizophrenia
294750011		acute exacerbation of subchronic catatonic schizophrenia
294751010		acute exacerbation of chronic catatonic schizophrenia
294753013		catatonic schizophrenia nos
294754019		unspecified paranoid schizophrenia
294757014		acute exacerbation of subchronic paranoid schizophrenia
294758016		acute exacerbation of chronic paranoid schizophrenia
294760019		paranoid schizophrenia nos
294764011		latent schizophrenia
294765012		unspecified latent schizophrenia
294766013		subchronic latent schizophrenia
294767016		chronic latent schizophrenia
294768014		acute exacerbation of subchronic latent schizophrenia
294769018		acute exacerbation of chronic latent schizophrenia
294770017		latent schizophrenia in remission
294771018		latent schizophrenia nos

294773015		cyclic schizophrenia
294787015		coenesthopathic schizophrenia
294788013		cenesthopathic schizophrenia
294789017		other schizophrenia nos
294790014		schizophrenia
294802018		single manic episode, unspecified
294803011		single manic episode, mild
294804017		single manic episode, moderate
294805016		single manic episode, severe
294806015		single manic episode, severe, with psychosis
294807012		single manic episode in partial remission
294808019		single manic episode in full remission
294809010		manic disorder, single episode nos
294810017		recurrent manic episodes
294811018		recurrent manic episodes, unspecified
294812013		recurrent manic episodes, mild
294813015		recurrent manic episodes, moderate
294815010		recurrent manic episodes, severe, with psychosis
294817019		recurrent manic episodes, in full remission
294818012		recurrent manic episode nos
294828015		single major depressive episode, severe, with psychosis
294840015		recurrent major depressive episodes, severe, with psychosis
294847017		manic-depressive - now manic
294848010		bipolar affective disorder, currently manic, unspecified
294849019		bipolar affective disorder, currently manic, mild
294850019		bipolar affective disorder, currently manic, moderate
294858014		bipolar affective disorder, currently manic, nos
294860011		manic-depressive - now depressed
294861010		bipolar affective disorder, currently depressed, unspecified
294862015		bipolar affective disorder, currently depressed, mild
294863013		bipolar affective disorder, currently depressed, moderate
294868016		bipolar affective disorder, currently depressed, nos
294869012		mixed bipolar affective disorder
294870013		mixed bipolar affective disorder, unspecified
294871012		mixed bipolar affective disorder, mild
294872017		mixed bipolar affective disorder, moderate
294874016		mixed bipolar affective disorder, severe, with psychosis
294876019		mixed bipolar affective disorder, in full remission
294877011		mixed bipolar affective disorder, nos
294880012		unspecified bipolar affective disorder
294881011		unspecified bipolar affective disorder, unspecified
294882016		mild bipolar disorder
294883014		moderate bipolar disorder
294887010		bipolar disorder in full remission
294888017		unspecified bipolar affective disorder, nos
294891017		other and unspecified manic-depressive psychoses
294892012		unspecified manic-depressive psychoses
294893019		atypical manic disorder

294895014		mixed bipolar i disorder
294896010		other and unspecified manic-depressive psychoses nos
294897018		affective psychosis
294898011		unspecified affective psychoses nos
294902017		other affective psychosis nos
294904016		simple paranoid state
294910016		other paranoid states
294911017		paranoia querulans
294912012		other paranoid states nos
294913019		paranoid psychosis
294917018		reactive depressive psychosis
294919015		acute hysterical psychosis
294924017	E134.00	Psychogenic paranoid psychosis
294926015		other reactive psychoses
294929010		other reactive psychoses nos
294933015	E14..00	Psychoses with origin in childhood
294939016	E141100	Residual disintegrative psychoses
294949018		other specified non-organic psychoses
296022017		[x]schizophrenia, schizotypal and delusional disorders
296031017		undifferentiated schizophrenia
296040018		[x]schizophrenia, unspecified
2960491000006119		severe manic bipolar i disorder with psychotic features
2960501000006110		severe bipolar i disorder, most recent episode manic, with psychotic features
296066015	Eu22z00	[X]Persistent delusional disorder, unspecified
296095012	Eu25y00	[X]Other schizoaffective disorders
296096013	Eu25z00	[X]Schizoaffective disorder, unspecified
296110017	Eu30100	Mania
296118012		[x]other manic episodes
296130018		bipolar disorder in remission
296135011		[x]bipolar affective disorder, unspecified
2964521000006113		moderate bipolar i disorder, single manic episode
2976051000006113		chronic undifferentiated schizophrenia
2981801000006118		bipolar i disorder, most recent episode depressed with atypical features
2988481000006116		chronic residual schizophrenia with acute exacerbations
2991561000006115		severe bipolar ii disorder, most recent episode major depressive with psychotic features
2994631000006113		bipolar ii disorder, most recent episode major depressive with postpartum onset

2998701000006116		manic bipolar i disorder in full remission
2998711000006118		bipolar i disorder, most recent episode manic, in full remission
300411000000110		h/o: psychosis
3005061000006111		bipolaris hawaiiense
3005081000006118		bipolaris hawaiiensis
3005591000006114		disorganised schizophrenia in remission
3005601000006118		disorganized schizophrenia in remission
3005611000006115		disorganized schizophrenia, in remission
300931000000113		referral to psychosis early intervention service
3036151000006112		monosymptomatic hypochondriacal psychosis
3037071000006117		severe manic bipolar i disorder with psychotic features, mood-incongruent
3037081000006119		severe bipolar i disorder, most recent episode manic, with psychotic features, mood-incongruent
3052571000006110		bipolar ii disorder, most recent episode major depressive with melancholic features
3067411000006110		chronic disorganised schizophrenia with acute exacerbations
3067421000006119		chronic disorganized schizophrenia with acute exacerbations
3068121000006112		disorganised schizophrenia
3068131000006110		disorganized schizophrenia
3071521000006117		mixed bipolar i disorder in remission
3071531000006119		bipolar i disorder, most recent episode mixed, in remission
3075241000006112		severe bipolar ii disorder, most recent episode major depressive, in remission
3077201000006119		moderate bipolar ii disorder, most recent episode major depressive
3082681000006117		schizophreniform disorder with good prognostic features
3089781000006115		mixed bipolar i disorder in partial remission
3089791000006117		bipolar i disorder, most recent episode mixed, in partial remission
3118041000006112		schizoaffective disorder, bipolar type
3137261000006111		undifferentiated schizophrenia in remission

3137271000006116		undifferentiated schizophrenia, in remission
3159061000006113		moderate mixed bipolar i disorder
3159071000006118		moderate bipolar i disorder, most recent episode mixed
3169541000006113		mild bipolar i disorder, single manic episode
3174001000006110		severe bipolar i disorder, single manic episode with psychotic features
3174071000006116		bipolar disorder, full remission
3201661000006111		bipolar ii disorder, most recent episode major depressive with atypical features
3204761000006117		mild mixed bipolar i disorder
3204771000006112		mild bipolar i disorder, most recent episode mixed
3233851000006115		manic bipolar i disorder in remission
3233861000006118		bipolar i disorder, most recent episode manic, in remission
3246851000006112		severe mixed bipolar i disorder without psychotic features
3246861000006114		severe bipolar i disorder, most recent episode mixed, without psychotic features
3300541000006111		depressed bipolar i disorder
3300551000006113		bipolar i disorder, most recent episode depressed
3301331000006119		bipolar i disorder, most recent episode depressed, in partial remission
3328681000006114		residual schizophrenia in remission
3328691000006112		residual schizophrenia, in remission
3333471000006115		amphetamine psychosis with hallucinations
3336841000006115		chronic bipolar i disorder, most recent episode depressed
3369281000006111		depressed bipolar i disorder in remission
3369291000006114		bipolar i disorder, most recent episode depressed, in remission
3385511000006118		witigo psychosis
3388761000006117		severe depressed bipolar i disorder with psychotic features, mood-congruent
3388771000006112		severe bipolar i disorder, most recent episode depressed with psychotic feature, mood-congruent
3401501000006110		bipolar i disorder, most recent episode manic with postpartum onset

3405421000006118		schizophreniform disorder without good prognostic features
3417321000006119		infection by bipolaris hawaiiense
3417351000006111		infection caused by bipolaris hawaiiense
3468381000006118		severe depressed bipolar i disorder with psychotic features
3468391000006115		severe bipolar i disorder, most recent episode depressed with psychotic features
346896012		reactive psychoses
346897015	E00y.11	Presbyophrenic psychosis
3497641000006117		severe bipolar i disorder, most recent episode depressed without psychotic features
3504751000006114		induced psychosis
3504761000006111		symbiotic psychosis
3515351010		psychotic disorder with schizophreniform symptoms caused by cocaine
3515352015		cocaine-induced psychotic disorder with schizophreniform symptoms
3515942013		postpartum psychosis in remission
3526134018		bipolaris sorokiniana
3527411000006119		paranoid schizophrenia, in remission
3528681000006111		bipolar i disorder, most recent episode manic, in partial remission
3532400017		bipolaris cactivora
3540391000006118		inhalant induced psychosis with hallucinations
3552841000006115		severe mixed bipolar i disorder with psychotic features, mood-congruent
3552851000006118		severe bipolar i disorder, most recent episode mixed, with psychotic features, mood-congruent
3555731000006114		paraphrenic schizophrenia
3557751000006110		bipolar i disorder, most recent episode mixed with postpartum onset
3583611000006115		moderate depressed bipolar i disorder
3583621000006111		moderate bipolar i disorder, most recent episode depressed
3589421000006110		severe bipolar ii disorder, most recent episode major depressive, in partial remission
359091000006115	Eu02z12	[X] Presenile psychosis NOS
359161000006110	Eu02z15	[X] Senile psychosis NOS
3615761000006116		manic bipolar i disorder
3615771000006111		bipolar i disorder, most recent episode manic

362381000006115		acute polymorphic psychotic disorder co-occurrent with symptoms of schizophrenia
362391000006117		acute polymorphic psychotic disorder without symptoms of schizophrenia
362421000006113		acute schizophrenia-like psychotic disorder
3627341000006115		psychosis
362781000006116		[x]affective psychosis nos
3636801010		synthetic cathinone-induced psychotic disorder with schizophreniform symptoms
3636803013		psychotic disorder with schizophreniform symptoms caused by synthetic cathinone
3647231000006119		severe bipolar disorder with psychotic features, mood-congruent
3651771000006116		subchronic residual schizophrenia with acute exacerbations
3656651000006111		chronic residual schizophrenia
3659701000006111		mild bipolar ii disorder, most recent episode major depressive
366571000006114		[x]atypical schizophrenia
367051000006112		severe depressed bipolar i disorder without psychotic features
367061000006114		psychosis and severe depression co-occurrent and due to bipolar affective disorder
367071000006119		bipolar affective disorder, currently manic, severe, with psychosis
367081000006116		[x]bipolar affect disorder cur epi manic wout psychotic symp
367091000006118		[x]bipolar affect disorder cur epi mild or moderate depressn
367101000006112		bipolar affective disorder
3671041000006111		mild manic bipolar i disorder
3671051000006113		mild bipolar i disorder, most recent episode manic
367111000006110	Eu31000	Bipolar affective disorder, current episode hypomanic
367121000006119		[x]bipolar affective disorder, current episode mixed
367151000006111		[x]bipolar disorder, single manic episode
367161000006113		[x]bipolar ii disorder
3672261000006113		bipolar cells of retina
367951000006113		borderline schizophrenia
367971000006115		[x]bouffee delirante with symptoms of schizophrenia

3679910000061 19		[x]brief reactive psychosis nos
3680010000061 12		[x]brief schizophreniform disorder
3680110000061 10		[x]brief schizophrenifrm psych
3690811000006 118		destruction of lesion or structure by biterminal bipolar fulguration
3694651000006 113		bipolar i disorder, most recent episode mixed with catatonic features
3704510000061 10		[x]catatonic schizophrenia
3706310000061 17		[x]cenesthopathic schizophrenia
3709810000061 12		[x]chronic hallucinatory psychosis
3710310000061 15		[x]chronic undifferentiated schizophrenia
3713841000006 116		mild depressed bipolar i disorder
3713851000006 119		mild bipolar i disorder, most recent episode depressed
3721561000006 114		inhalant induced psychosis with delusions
3725431000006 118		bipolar i disorder, single manic episode, in remission
3725441000006 111		bipolar 1 disorder, single manic episode, in remission
3731661000006 112		bipolar i disorder, most recent episode depressed with melancholic features
3745281000006 119		subchronic residual schizophrenia
3762510000061 12		[x]cyclic schizophrenia
3762710000061 19		[x]cycloid psychosis
3762810000061 16		cycloid psychosis
3773101000006 118		bipolar i disorder, single manic episode, in partial remission
3773111000006 115		bipolar 1 disorder, single manic episode, in partial remission
3774610000061 13	Eu843 12	[X]Disintegrative psychosis
3779041000006 118		severe manic bipolar i disorder with psychotic features, mood-congruent
3779051000006 116		severe bipolar i disorder, most recent episode manic, with psychotic features, mood-congruent
3780510000061 19		[x]disorganised schizophrenia
3788101000006 118		chronic undifferentiated schizophrenia with acute exacerbations

3816191000006113		bipolaris australiensis
3822471000006114		severe bipolar ii disorder, most recent episode major depressive without psychotic features
3850471000006114		moderate manic bipolar i disorder
3850481000006112		moderate bipolar i disorder, most recent episode manic
3854401000006113		bipolar 2 disorder
3862661000006112		chronic schizophrenia
3878611000006116		schizophreniform psychosis, depressive type
3886291000006119		bipolar disorder, in remission
388741000006110		[x]hebephrenic schizophrenia
389401000006111	Eu30000	Hypomania
389451000006110		[x]hysterical psychosis
3895621000006112		subchronic undifferentiated schizophrenia
3898991000006114		severe bipolar i disorder, single manic episode with psychotic features, mood-incongruent
3917861000006119		bipolar i disorder, most recent episode depressed with postpartum onset
3929161000006110		bipolar i disorder, single manic episode with catatonic features
3933581000006112		bipolaris spicifera
394461000006110		[x]involutional paranoid state
3946141000006118		schizophreniform disorder
395021000006111		[x]latent schizophrenia
395031000006114		[x]latent schizophrenic reaction
396071000006119		[x]manic-depressive reaction
396691000006111	Eu30z11	[X]Mania NOS
396701000006111	Eu30211	[X]Mania with mood-congruent psychotic symptoms
396711000006114	Eu30212	[X]Mania with mood-incongruent psychotic symptoms
396741000006113		[x]manic episode
396771000006117		[x] manic-depressive psychosis, depressed type without psychotic symptoms

396781000006119		[x]manic-depress psychosis,depressed type+psychotic symptoms
396791000006116		manic-depressive illness
396801000006115		manic-depressive psychosis
397581000006111		cannabis-induced psychosis
397641000006116		cocaine-induced psychosis
397661000006117		opioid-induced psychosis
397691000006113		volatile inhalant-induced psychosis
398541000006116		bipolar affective disorder , current episode mixed
398631000006113		mixed schizophrenic and affective pschosis
401759010	E00y.00	Other senile and presenile organic psychoses
401763015		acute schizophrenic episode
401764014		other schizophrenia
401765010		manic disorder, single episode
401768012		chronic paranoid psychosis
401771016		non-organic psychosis nos
401855010		[x]other schizophrenia
401856011		schizotypal personality disorder
401857019	Eu22y00	[X]Other persistent delusional disorders
401862018		[x]unspecified nonorganic psychosis
401863011	Eu30200	[X]Mania with psychotic symptoms
401864017		[x]manic episode, unspecified
401865016		[x]other bipolar affective disorders
401869010	Eu32300	[X]Severe depressive episode with psychotic symptoms
402504012	285..11	Psychotic condition, insight present
4082931000006110		schizophrenic reaction
4119081000006111		schizophrenic relative
412201000006113	Eu2y.00	[X]Other nonorganic psychotic disorders
418181000006110		[x]paranoia querulans
418221000006118		[x]paranoid schizophrenia
418231000006115		[x]paranoid state
418251000006110	Eu22013	Late paraphrenia

418261000006112		[x]paraphrenic schizophrenia
4196151000006111		subchronic schizophrenia with acute exacerbations
4196171000006118		schizophrenia, catatonic, in remission
4196201000006119		mixed bipolar i disorder in full remission
4196211000006116		bipolar i disorder, most recent episode mixed, in full remission
419861000006117	Eu22.00	Persistent delusional disorder
423041000006119		[x]post-schizophrenic depression
423271000006116		prepsychotic schizophrenia
423471000006117		prodromal schizophrenia
423731000006114		[x]pseudoneurotic schizophrenia
423741000006116		[x]pseudopsychopathic schizophrenia
424071000006112	Eu23312	[X]Psychogenic paranoid psychosis
424231000006116	Eu2z.11	Psychotic
424511000006112		[x]reactive psychosis
424551000006113	Eu33314	[X]Recurr severe episodes/psychogenic depressive psychosis
424661000006111		[x]recurrent manic episodes
424671000006116	Eu33315	[X]Recurrent severe episodes of psychotic depression
424681000006118		recurrent reactive depressive episodes, severe, with psychosis
424841000006113		[x]residual schizophrenia
424931000006113		[x]restzustand schizophrenic
425501000006113	Eu25100	Schizoaffective disorder, depressive type
425511000006111	Eu25000	Schizoaffective disorder, manic type
425521000006115	Eu25200	Schizoaffective disorder, mixed type
425541000006110	Eu25.00	Schizoaffective disorder
425551000006112		[x]schizoaffective psychosis nos
425561000006114		[x]schizoaffective psychosis, depressive type

425571000006119		[x]schizoaffective psychosis, manic type
425601000006114		[x]schizophrenia
425641000006111		[x]schizophrenic catalepsy
425651000006113		[x]schizophrenic catatonia
425671000006115		[x]schizophrenic reaction
425681000006117		[x]schizophreniform disord nos
425691000006119		[x]schizophreniform psychosis, depressive type
425701000006119		[x]schizophreniform psychosis, manic type
425711000006116		[x]schizophrenifrm psychos nos
425731000006110		[x]schizotypal personality disorder
426881000006111		[x]simple schizophrenia
426981000006116		[x]single episode of reactive depressive psychosis
428441000006116		[x]symbiotic psychosis
428451000006119	Eu0z.12	Psychotic symptom present
43595011		residual schizophrenia
44335019	E122.00	Paraphrenia
4539931000006112		history of schizophrenia
456731000006115		acute exacerbation of chronic schizo-affective schizophrenia
456801000006115		acute exacerbation subchronic schizo-affective schizophrenia
460273017		[v]personal history of schizophrenia
473201000006114	E11..00	Mood disorder
4763611000006115		acute exacerbation of subchronic disorganised schizophrenia
4763631000006114		acute exacerbation of subchronic disorganized schizophrenia
4763641000006116		acute exacerbation of chronic disorganised schizophrenia
4763661000006117		acute exacerbation of chronic disorganized schizophrenia
4763691000006113		schizophrenia, catatonic
4763701000006113		schizophrenic flexibilatis cerea

4763731000006117		subchronic catatonic schizophrenia with acute exacerbation
4763751000006112		chronic catatonic schizophrenia with acute exacerbation
4763771000006119		subchronic paranoid schizophrenia with acute exacerbation
4763791000006118		chronic paranoid schizophrenia with acute exacerbation
4763861000006116		latent schizophrenia, in remission
4763891000006112		subchronic schizoaffective schizophrenia
4763911000006114		acute exacerbation of subchronic schizoaffective schizophrenia
4763921000006118		acute exacerbation of chronic schizoaffective schizophrenia
4763941000006113		schizoaffective schizophrenia, in remission
4763951000006110		schizophrenia, schizoaffective, in remission
4764391000006115		paranoia
4767311000006117		bipolar affective disorder, current episode mixed
492761000000114		bipolar affective disorder resolved
492821000000113		schizophrenia resolved
492881000000114		psychosis resolved
4997831000006110		schizophrenic language
5023391000006116		reactive psychosis
5023541000006116		psychosis associated with intensive care
5023551000006119		intensive care psychosis
5023901000006110		post-schizophrenic depression
5128631000006110		cutaneous monosymptomatic delusional psychosis
513691000006116		severe manic bipolar i disorder without psychotic features
513701000006116		bipolar affect disord, currently manic,severe with psychosis
513711000006118		depressed bipolar i disorder in partial remission
513721000006114		bipolar affect disord, now depressed, severe with psychosis
513731000006112		bipolar affect disord, now depressed, severe, no psychosis

5137410000061 19		manic bipolar i disorder in partial remission
5137510000061 17		bipolar affective disorder, current episode depression
5138010000061 12		bipolar affective disorder, current episode manic
5138110000061 10		bipolar affective disorder, currently manic, in full remission
5138610000061 13		bipolar affective disorder, currently depressed, in full remission
5138710000061 18		bipolar disorder
5248961000006 113		schizophrenic prodrome
52897013		chronic paranoid schizophrenia
5387561000006 118		bipolar diathermy
5435901000006 117		bipolar
5517971000006 118		mixed schizophrenic and affective psychosis
5524571000006 112		schizophreniform psychosis, manic type
5566310000061 16		chronic schizoaffective schizophrenia
5614121000006 115		involutional paranoid state
5933701000006 116		profile of mood states, bipolar
5933841000006 114		rust inventory of schizotypal cognitions
6137910000061 15		depressive psychoses
6357571000006 119		bipolar 1 disorder
6357621000006 116		severe bipolar i disorder
6357631000006 118		severe bipolar disorder
6357651000006 113		severe bipolar ii disorder
6515001000006 117		early intervention in psychosis
6519871000006 118		early intervention in psychosis - 24 hour intensive
6519881000006 115		early intervention in psychosis - 24 hour not intensive
6519891000006 117		early intervention in psychosis - part day : day care
6519901000006 118		early intervention in psychosis - daily intensive
6519911000006 115		early intervention in psychosis - 3-5 contacts/week

6519921000006111		early intervention in psychosis - 1-2 contacts/week
6519931000006114		early intervention in psychosis - 1-3 contacts/month
6519941000006116		early intervention in psychosis - <1 contact/month
6520581000006119		early intervention in psychosis hrgs
6647221000006111		history of manic depressive disorder
6890801000006117		late onset schizophrenia
6891981000006111		referral for bipolar disorder
701051000006118		mixed bipolar affective disorder, partial/unspec remission
701071000006111		mixed bipolar affective disorder, severe
71539017		subchronic catatonic schizophrenia
7443501000006113		bipolar partial shoulder prosthesis
7453771000006111		endotherapy electrosurgical coagulator/cutter, bipolar, reusable
7456141000006112		endotherapy electrosurgical coagulator/cutter, bipolar, refurbished
7461241000006118		humeral head bipolar component
7467941000006117		femoral head bipolar component
7472911000006112		laparoscopic electrosurgical forceps, bipolar
7488321000006114		atypical psychosis
7538311000006116		planar-resection electrosurgical diathermy bipolar conducting unit
7548121000006114		universal electrosurgical diathermy system bipolar electrode, reprocessed
7548151000006117		universal electrosurgical diathermy system bipolar electrode, single-use
7558781000006111		ppmx - mental retardation with psychosis, pyramidal signs, and macroorchidism
7567101000006113		bipolar clinic
7568231000006110		schizophrenia clinic
7696261000006113		acute polymorphic psychotic disorder with symptoms of schizophrenia
7707781000006115		partial hip replacement with bipolar prosthesis
7756771000006110		endoscopic electrosurgical electrode, bipolar, reprocessed
7756991000006113		endoscopic electrosurgical electrode, bipolar, reusable

7757001000006117		endoscopic electrosurgical electrode, bipolar, single-use
7757081000006114		percutaneous electrosurgical probe, spinal-denervation, bipolar
7760691000006114		bipolaris cynodontis
7767871000006111		laparoscopic electrosurgical forceps, bipolar, reprocessed
7790241000006117		psychosis co-occurrent and due to parkinson's disease
7822921000006111		provision of information about psychosis
7826381000006113		hysteroscopic bipolar electrosurgical excision of uterine fibroid
7849081000006116		bipolar type i disorder currently in full remission
7849091000006118		bipolar type ii disorder currently in full remission
7853100000611 6		unspecified schizo-affective schizophrenia
7860821000006114		manic symptoms co-occurrent and due to primary psychotic disorder
7860831000006112		manic symptoms with primary psychotic disorder
7880861000006118		h/o: postpartum psychosis
7880871000006113		history of postpartum psychosis
789221000006116	E110.11	Hypomanic psychoses
7965151000006112		severe depressed bipolar i disorder
7965161000006114		severe mixed bipolar i disorder
8009371000006115		phencyclidine psychosis
8011471000006113		severe manic bipolar i disorder
8019691000006113		bipolar ii disorder, most recent episode rapid cycling
8019701000006113		bipolar 2 disorder, most recent episode rapid cycling
8028531000006112		early intervention in psychosis team
8041781000006111		rapid cycling bipolar i disorder
8089081000006116		psychosis caused by inhalant
8089301000006110		reactive depressive psychosis, single episode
819351000006115		hebephrenic schizophrenia

82091000006116		bipolar disorder in partial remission
82151000006114		severe bipolar disorder without psychotic features
82171000006116		severe bipolar disorder with psychotic features
8232521000006119		paranoid state in remission
8232541000006114		non-organic psychosis in remission
8232571000006118		single major depressive episode, severe, with psychosis, psychosis in remission
8232591000006117		recurrent major depressive episodes, severe, with psychosis, psychosis in remission
8235161000006110		mixed bipolar affective disorder, in partial remission
8237821000006118		recurrent manic episodes, in remission
8237871000006117		single manic episode in remission
8302181000006114		hysteroscopic bipolar electrosurgical excision of uterine polyp
8342781000006115		psychoactive substance-induced psychosis
8365411000006116		family intervention for psychosis
8369811000006116		early intervention in psychosis procedures simple reference set
8434671000006112		schizophrenia tom (therapy outcome measure) activity score
8434681000006110		schizophrenia tom (therapy outcome measure) carer wellbeing score
8434701000006113		schizophrenia tom (therapy outcome measure) impairment score
8434711000006111		schizophrenia tom (therapy outcome measure) participation score
8434721000006115		schizophrenia tom (therapy outcome measure) wellbeing score
8464961000006115		recurrent reactive depressive episodes, severe, with psychosis
882281000006113		schizophrenic psychoses nos
882291000006111		schizophrenic psychoses
882301000006112		acute schizo affective psychosis
882311000006110		manic-depressive psychoses
882331000006116		paranoia
914461000006118	Eu22111	[X]Capgras syndrome

914471000006113	Eu22100	[X]Delusional misidentification syndrome
9225016		schizophrenia in remission
9326201000006116		psychosis in pregnancy
967791000006111	Eu22200	[X]Cotard syndrome
996171000006114		psychosis, schizophrenia and bipolar affective disorder resolved

CVA/TIA

medcode	readcode	desc
106392017		Basilar artery syndrome
106394016		Vertebrobasilar insufficiency
118689010		Cerebral thrombosis
11878511000006113		Cerebrovascular accident due to occlusion of cerebral artery
11903571000006110		Stroke due to intracerebral haemorrhage
11919571000006110		[X]Other intracerebral haemorrhage
11920121000006117		Transient cerebral ischemia
1212072018		Occlusive stroke
12223101000006118		Intracerebral haemorrhage
12223111000006115		Intracerebral hemorrhage
12223121000006111		ICH - intracerebral haemorrhage
12223131000006114		ICH - intracerebral hemorrhage
1222398015		Cerebral arterial occlusion
122361000006113		Stroke due to cerebral arterial occlusion
122371000006118		Stroke due to intracerebral haemorrhage
122401000006115		Stroke unspecified
1235534016		Binswanger's encephalopathy
125470015		Cerebral embolism
12722481000006116		Right sided intracerebral haemorrhage, unspecified
12762021000006116		Cerebral hemorrhage
13031191000006112		Cerebral hemorrhage
130375018		Lateral medullary syndrome
149347010		Binswanger's disease
158118014		Cerebellar infarction
1667741000000110		[V]Personal history of transient ischaemic attack
218511000000117		Infarction - cerebral
2474651019		Infarction of basal ganglia
2476647018		Vertebral artery compression syndrome
25897016		Subclavian steal syndrome
2622631000006114		Intrapontine haemorrhage
2622641000006116		Intrapontine hemorrhage
2622651000006119		Pontine hemorrhage
2818821000006111		Occlusion of cerebral artery
299342019		[X]Other lacunar syndromes
300276019		External capsule haemorrhage
300287010		Intracerebral haemorrhage NOS
300312010		Cerebral infarct due to thrombosis of precerebral arteries

300313017		Cerebral infarction due to embolism of precerebral arteries
300321011		Cerebral infarction due to thrombosis of cerebral arteries
300322016		Cerebral infarction due to embolism of cerebral arteries
300344014		Carotid artery syndrome hemispheric
300345010		Multiple and bilateral precerebral artery syndromes
300348012		Other transient cerebral ischaemia
300349016		Transient cerebral ischaemia NOS
300352012		Impending cerebral ischaemia
300353019		Intermittent cerebral ischaemia
300362017		Middle cerebral artery syndrome
300363010		Anterior cerebral artery syndrome
300364016		Posterior cerebral artery syndrome
300366019		Cerebellar stroke syndrome
300370010		Left sided CVA
300371014		Right sided CVA
300939013		[X]Other intracerebral haemorrhage
300941014		[X]Other cerebral infarction
300943012		[X]Occlusion and stenosis of other cerebral arteries
300956017		[X]Intracerebral haemorrhage in hemisphere, unspecified
3299621000006119		Cortical hemorrhage
3346051000006110		Internal capsule hemorrhage
345639010		Infarction - precerebral
345650013		Brainstem infarction NOS
345655015		Pure motor lacunar syndrome
345658018		Pure sensory lacunar syndrome
345675012		Lobar cerebral haemorrhage
345684012		Carotid territory transient ischaemic attack
3540781000006117		Vertebrobasilar arterial insufficiency
3662311000006113		Thrombosis of cerebral arteries
3662321000006117		Cerebral arterial thrombosis
3662331000006119		CT - Cerebral thrombosis
370661000006114		[X]Cereb infarct due unsp occlus/stenos precerebr arteries
370701000006118		[X]Cerebrl infarctn due/unspcf occlusn or sten/cerebrl artrs
3719851000006116		Cerebellar hemorrhage
3719861000006119		Haemorrhagic cerebellum
3719871000006114		Hemorrhagic cerebellum
3728451000006116		Cerebral arterial embolism
3777981000006119		Posterior inferior cerebellar artery syndrome
3778011000006112		Inferior cerebellar artery syndrome
3778021000006116		LMS - Lateral medullary syndrome
395780010		Cerebral infarction NOS
395783012		Transient ischaemic attack
395788015		Transient cerebral ischaemia
3964591000006117		Subcortical leucoencephalopathy
3964601000006113		Subcortical leukoencephalopathy
3964611000006111		Binswanger's dementia
3964621000006115		Encephalitis subcorticalis chronica
3964651000006112		Subcortical arteriosclerotic encephalopathy

3964661000006114		Subcortical atherosclerotic dementia
3964671000006119		Chronic progressive subcortical encephalopathy
405339016		Stroke and cerebrovascular accident unspecified
4057031000006117		Brain stem infarct
4057041000006110		Brain stem stroke
4057051000006112		Infarction of brain stem
411416011		Stroke in the puerperium
416991000006112		[X]Other transnt cerebral ischaemic attacks+related syndroms
451133011		Left sided cerebral infarction
451134017		Right sided cerebral infarction
4777811000006117		Basal ganglia hemorrhage
4777831000006111		External capsule hemorrhage
4777881000006112		Intracerebral haemorrhage, multiple localised
4777891000006110		Intracerebral hemorrhage, multiple localized
4778071000006118		Carotid artery syndrome
4778101000006111		Impending cerebral ischemia
4778121000006118		Intermittent cerebral ischemia
4778181000006119		Left sided cerebral hemisphere cerebrovascular accident
4778201000006118		Right sided cerebral hemisphere cerebrovascular accident
483988011		Bulbar haemorrhage
495394013		Cortical haemorrhage
496232015		Internal capsule haemorrhage
499739014		Insufficiency - basilar artery
5010981000006119		Stroke
5011021000006113		Cerebrovascular accident due to cerebral artery occlusion
5011161000006115		Lacunar stroke
5011171000006110		LACI - Lacunar infarction
5011181000006113		LI - Lacunar infarction
5011191000006111		Pure motor lacunar infarction
5011211000006112		Pure sensory lacunar infarction
5011401000006110		Lobar cerebral hemorrhage
5011491000006115		Anterior circulation transient ischaemic attack
5011501000006111		Anterior circulation transient ischemic attack
5011511000006114		Carotid territory transient ischemic attack
502878012		Cerebellar haemorrhage
503469016		Pontine haemorrhage
503791000006114		Basal nucleus haemorrhage
524511000006116		Brain stem stroke syndrome
524541000006117		Brainstem infarction
542251000006112		Cereb infarct due cerebral venous thrombosis, nonpyogenic
542261000006114		Cereb infarct due unsp occlus/stenos precerebr arteries
542831000006116		Cerebral embolus
543141000006110		Cerebrl infarctn due/unspcf occlusn or sten/cerebrl artrs
5492171000006112		TIA
5492181000006110		Temporary cerebral vascular dysfunction
5492201000006111		TIA - Transient ischaemic attack
5492221000006118		Transient ischemic attack
5560161000006111		Intracerebral hemorrhage (ICH)

57341000006119		Wallenberg syndrome
58046010		Vertebral artery syndrome
605461000006117		CVA - cerebral artery occlusion
605471000006112		CVA - cerebrovascular accid due to intracerebral haemorrhage
605481000006110		CVA - cerebrovascular accident in the puerperium
605491000006113		CVA - Cerebrovascular accident unspecified
605501000006117		CVA unspecified
67501000006115		Vertebro-basilar artery syndrome
67511000006117		Vertebro-basilar insufficiency
6837051000006119		Basal ganglion stroke
6837061000006117		Basal ganglion infarct
744901000006114		Intracerebral haemorrhage
744921000006116		Intracerebral haemorrhage in hemisphere, unspecified
746571000006116		Intracerebral haemorrhage, multiple localized
748941000006115		Left sided intracerebral haemorrhage, unspecified
7951271000006116		Haemorrhage of medulla oblongata
7951281000006118		Hemorrhage of medulla oblongata
8231151000006110		Personal history of transient ischaemic attack
884421000006119		Cerebral haemorrhage
95931000006111		Transient cerebral ischaemia NOS
988951000006117		Transient Ischaemic Attacks
989201000006117		Cerebral haemorrhage
989211000006119		Cerebral haemorrhage NOS

Learning disability

medcode	readcode	desc
315486012		Fragile X chromosome
378493013		Down's syndrome NOS
1224878018		Trisomy 21 NOS
398241000006118		[X]Mental retardation with autistic features
295661017		Other specified mental retardation
296592018		[X]Other mental retardation, other impairments of behaviour
401902015		[X]Unspecified mental retardatio
398231000006111		[X]Mental retardation
404861000006115		[X]Oth mental retard with statement no or min impairm behav
430061000006113		[X]Unsp mental retard with statement no or min impairm behav
430071000006118		[X]Unsp mental retardation without mention impairment behav
146051000006113		Severe mental retardation, IQ in range 20-34
201751000006110		Profound mental retardation with IQ less than 20

398821000006114		X]Moderate mental subnormality
417681000006116		[X]Overactive disorder assoc mental retard/stereotype movts
423521000006119		[X]Profound mental subnormality
296557014		[X]Mild mental retardation, other impairments of behaviour
296574014		[X]Severe mental retardation, other impairments of behaviour
398661000006116		[X]Mld mental retard with statement no or min impairm behav
423481000006119		[X]Profound ment retard sig impairmnt behav req attent/treat
420581000006111		Autism spectrum disorder
1129811000000119		Moderate learning disability
1550051000000113		Profound learning disability
398391000006116		Mild intellectual disability
423501000006112		Profound intellectual disability
426591000006111		Severe intellectual disability
426611000006117		[X]Severe mental subnormality
296565012		[X]Mod retard oth behav impair
398411000006116		[X]Mild mental subnormality
423361000006118		[X]Prfnd mental retardation without mention impairment behav
423511000006110		[X]Profound mental retardation, other impairments of behavr
222121000000113		Trisomy 21
2090010		Fragile X syndrome
507246016		Mild mental retardation, IQ in range 50-70
1550041000000110		Mild learning disability
296678017		[X]Other pervasive developmental disorders
398781000006115		[X]Mod mental retardation without mention impairment behav
426541000006119		[X]Sev mental retardation without mention impairment behav
151009017		Mental retardation
296586012		[X]Other mental retardation
398201000006115		[X]Mental deficiency NOS
398251000006116		[X]Mental subnormality NOS
404851000006117		[X]Oth mental retard sig impairment behav req attent/treatmt

431231000006115		[X]Unspecified mental retardatn, other impairments of behav
413177014		Educationally subnormal
1129781000000117		Severe learning disability
295664013		Intellectual disability
628281000006114		Down's syndrome
398651000006118		[X]Mld mental retard sig impairment behav req attent/treatmt
423491000006116		[X]Profound ment retrd wth statement no or min impairm behav
9881000006115		Other specified mental retardation
411791000006118		[X]Other mental retardation without mention impairment behav
430081000006115		[X]Unsp mentl retard sig impairment behav req attent/treatmt
296679013		[X]Pervasive developmental disorder, unspecified
700071000006118		Moderate mental retardation, IQ in range 35-49
398761000006113		[X]Mod mental retard sig impairment behav req attent/treatmt
398771000006118		[X]Mod mental retard with statement no or min impairm behav
426521000006114		[X]Sev mental retard sig impairment behav req attent/treatmt
426531000006112		[X]Sev mental retard with statement no or min impairm behav
295662012		Other specified mental retardation NOS
398381000006119		[X]Mild mental retardation
395061000006117		Learning disability
398811000006118		Moderate intellectual disability
10374011		Laurence-Moon-Biedl syndrome
108541018		hurler's syndrome
110901011		Acrodysostosis
11998101000006118		[X]Severe learning disability
12177531000006112		[X]Moderate learning disability
12177541000006119		Intellectual developmental disorder
12181611000006117		Profound intellectual disability
12181621000006113		Profound intellectual development disorder
12190771000006114		Severe intellectual disability
12190781000006112		Severe intellectual development disorder
12202441000006114		Moderate intellectual disability

1220245100000 6111		Moderate intellectual development disorder
1221474011		Mild intellectual disability
1224879014		Mild intellectual development disorder
1224880012		Deletion of short arm of chromosome 4
1224941015		Trisomy 21 NOS
1229637015		Trisomy 18 NOS
1229639017		Trisomy 13 NOS
1231577014		Dubowitz syndrome
1232189100000 6112		Degenerative amsterodamensis typus
1233229018		Bruck-de Lange syndrome
1233764100000 6113		Biedl-Bardet syndrome
1234038018		Intellectual disability, congenital heart disease, blepharophimosis, blepharoptosis and hypoplastic teeth
1235959100000 6114		Deletion of short arm of chromosome 5
1270221100000 6116		X-linked intellectual disability with marfanoid habitus
1270378100000 6112		Angelman's syndrome
1270379100000 6110		Hirschsprung disease-intellectual disability syndrome
1270392100000 6110		Specific learning difficulty
1270394100000 6115		Mild mental retardation (I.Q. 50-70)
127638013		Moderate mental retardation (I.Q. 35-49)
127639017		Profound mental retardation (I.Q. below 20)
148214012		Severe mental retardation (I.Q. 20-34)
1694831000006 112		Angelman syndrome
1705901000006 118		Happy puppet syndrome
1780502018		Severe mental retardation, IQ in range 20-34
1803731000006 114		Prader-Willi syndrome
1887331000006 119		Mental retardation
2114791000000 110		[X]Mild learning disability
2129110000061 16		[X]Profound learning disability
2129210000061 12		Pitt-Hopkins syndrome
2158210000001 19		Angelman syndrome
2399910000061 11		Smith-Magenis syndrome
2478440016		Acrodysostosis
2540251000006 114		[X]Specific learning disability
2548475019		Profound mental retardation with IQ less than 20
25776014		Fragile X syndrome
2589171000006 110		Kleefstra syndrome

2589191000006111		Prader - Willi syndrome
2740611000006112		Prader-Willi Syndrome
2771711000006112		Trisomy 21
2771721000006116		Patau's syndrome - trisomy 13
2771731000006118		Ohdo blepharophimosis syndrome
2835541000006116		Dubowitz's syndrome
2835561000006117		On learning disability register
2844171000006117		Coffin-Lowry syndrome
2844181000006119		Bardet-Biedl syndrome
297751000006119		LMBB - Laurence-Moon-Bardet-Biedl syndrome
3003301000006113		CLS - Coffin-Lowry syndrome
3003311000006111		4p partial monosomy syndrome
3003321000006115		Chromosome 4 short arm deletion syndrome
3003331000006117		4p minus syndrome
3003341000006110		Midline fusion defect syndrome
3003351000006112		Complete trisomy 13 syndrome
3003361000006114		D>1< trisomy syndrome
302051000000118		BFLS
3149211000006118		Borjeson syndrome
3149231000006112		Other specified mental retardation
3149251000006117		Other specified mental retardation NOS
3149271000006110		Mental retardation NOS
3155151000006112		[X]Mild mental retardation, other impairments of behaviour
3155171000006119		[X]Mod retard oth behav impair
3155181000006116		[X]Severe mental retardation, other impairments of behaviour
3155191000006118		[X]Other mental retardation
3155201000006115		[X]Other mental retardation, other impairments of behaviour
315625018		[X]Other pervasive developmental disorders
3161031000006112		[X]Pervasive developmental disorder, unspecified
3161041000006119		Noonan's syndrome
3161051000006117		Profound mental handicap

3161061000006<br>115		Profound mental retardation (Intelligence Quotient below 20)
3334491000006<br>116		Profound learning disability with intelligence quotient less than 20
3334501000006<br>112		Profound learning impairment with intelligence quotient less than 20
3493641000006<br>112		Profound learning disability, intelligence quotient less than 20
3493651000006<br>114		Profound learning impairment, intelligence quotient less than 20
3493661000006<br>111		Profound learning disability
3493671000006<br>116		Lujan-Fryns syndrome
3493681000006<br>118		De Lange syndrome
3562061000006<br>117		Brachmann-de Lange syndrome
3562071000006<br>112		Degenerative amstelodamensis typus
3562081000006<br>110		Typus degenerativus amstelodamensis
3562091000006<br>113		Fragile X chromosome
3562111000006<br>116		Severe learning disability
3562121000006<br>112		Severe mental handicap
3562131000006<br>110		Severe mental retardation (Intelligence Quotient 20-34)
3562141000006<br>117		Severe learning disability, intelligence quotient in range 20-34
3562151000006<br>115		Severe learning impairment, intelligence quotient in range 20-34
3562161000006<br>118		Noonan's syndrome
3585501000006<br>118		Complete trisomy 21 syndrome
3616531000006<br>110		Down syndrome
3616551000006<br>115		T21 - Trisomy 21
3616561000006<br>118		Downs syndrome
3616571000006<br>113		Complete trisomy 18 syndrome
3616581000006<br>111		Edwards syndrome
3616591000006<br>114		Moderate mental handicap
36300015		Moderate mental retardation (Intelligence Quotient 35-49)
3640931000006<br>110		Moderate learning disability, intelligence quotient in range 35-49
3640951000006<br>115		Moderate learning impairment, intelligence quotient in range 35-49
3640961000006<br>118		Moderate learning disability
3640971000006<br>113		mucopolysaccharidosis, mps-i-h
3750051000006<br>112		l-iduronidase deficiency, hurler type

378494019		lipochondrodystrophy
378496017		gargoylism
378499012		hurler-pfaundler syndrome
378500015		dysostosis multiplex syndrome
386751000006111		mps 1-h - mucopolysaccharidosis type i-h
3910901000006110		mucopolysaccharidosis type i-h
3910921000006117		hurler disease mps type 1h
3910931000006119		mucopolysaccharidosis type i severe form
3910941000006112		Peripheral dysostosis
3910951000006114		Rett's disorder
395051000006119		Cerebroatrophic hyperammonemia
424971000006111		Cerebroatrophic hyperammonaemia
4830941000006112		RTS - Rett syndrome
4830961000006111		Rett disorder
4830971000006116		Retts syndrome
4831981000006110		Borjeson-Forssman-Lehmann syndrome
4831991000006113		5p partial monosomy syndrome
4832001000006110		Lejeune syndrome
5339911000006112		5p minus syndrome
5339921000006116		Partial deletion of short arm of chromosome 5 syndrome
5339971000006115		Angelman syndrome
5339981000006117		Down's syndrome NOS
53891000006113		Partial trisomy 21 in Down's syndrome
557191000000119		Edward's syndrome NOS
600811000006111		Patau's syndrome NOS
636711000006113		Partial trisomy 13 in Patau's syndrome
63896011		[X]Feeble-mindedness
63898012		Mild mental handicap
667621000006119		Mild mental retardation (Intelligence Quotient 50-70)
6989771000006117		Mild learning disability, intelligence quotient in range 50-70
7558461000006113		Mild learning impairment, intelligence quotient in range 50-70
7577251000006111		Mild learning disability
7577261000006113		[X]Learn acquisition disab NOS

785941000006115		[X]Learning disability NOS
787151000006113		[X]Mental deficiency NOS
79051000006114		[X]Mental retardation
8191411000006110		[X]Mental retardation with autistic features
829441000006116		[X]Mental subnormality NOS
8337561000006119		[X]Mild mental retardation
8337571000006114		[X]Mild mental retardation without mention impairment behav
8337581000006112		[X]Mild mental subnormality
8337591000006110		[X]Mld mental retard sig impairment behav req attent/treatmt
88271000006113		[X]Mld mental retard with statement no or min impairm behav
882771000006119		[X]Mod mental retard sig impairment behav req attent/treatmt
882781000006116		[X]Mod mental retard with statement no or min impairm behav
882791000006118		[X]Mod mental retardation without mention impairment behav
88351000006114		[X]Moderate mental retardation
88361000006111		[X]Moderate mental subnormality
88371000006116		[X]Unspecified mental retardation
88381000006118		[X]Oth mental retard sig impairment behav req attent/treatmt
893481000006117		[X]Oth mental retard with statement no or min impairm behav
94131019		[X]Other mental retardation without mention impairment behav
968201000006114		Educationally subnormal

2 codes were identified by the searches but deemed grossly offensive and were excluded. These terms are no longer used in clinical practice or every day language. Any cases missed by excluding these terms will be minimal, and they represent clearly suboptimal coding.

3. **Outcomes**

For each outcome, a codelist was developed. These were developed from existing codelists, along with a search of relevant terms from the CPRD Aurum Data dictionary. The medication codelists were taken from existing codelists, available online, and further updated to ensure they were as sensitive as possible, searching the CPRD Aurum Data Dictionary using the drug generic name stem. The codelist for high anti-cholinergic burden drugs was taken from the UEA Aging Brain Care, Anticholinergic Cognitive Burden Scale (2012)(4).

Care Plan

medcode	readcode	desc
11930781000006119		Dementia care plan
1834091000006117		Dementia care plan
1949681000006119		Person centred dementia support plan (Scotland)
1949691000006116		Dementia support plan (Scot) in place at 12 months
1949701000006116		Dementia support plan (Scot) partly in place at 12 months

1949711000006118		Dementia support plan (Scot) absent at 12 months
2248021000000110	8CMZ.00	Dementia care plan
2373161000000116	8T05100	Referral to dementia support organisation declined
2373201000000112	8T05000	Referral to dementia support organisation
2439591000000113	8CMZ000	Dementia care plan agreed
2439631000000113	8CMZ100	Dementia care plan reviewed
2439671000000110	8CMZ200	Dementia care plan declined
2439711000000111	8CMZ300	Dementia care plan review declined
2714871000000112		Signposting to dementia support service
408401000000119	6AB..00	Dementia annual review
915111000006112		Dementia review
915121000006116		Dementia review with third party

Medication Review

medcode	readcode	desc
11635931000006111		Care planning medication review simple reference set
12117691000006117		Medication review
12620351000006114		STOMP (Stopping Over-Medication of People with Learning Disability, Autism or Both) medication review
12736811000006111		Medication review done by clinical pharmacist
13072961000006111		Structured medication review
137851000000113		Medication review with patient
1485178017		Medication review without patient
1488692014		Other medication review
1488739013		Medication review done by pharmacist
1562621000000119		Concordance and compliance level 2 medication review
1576381000006112		Tele. Reminder for Medication Review
1576401000006112		Verbal Reminder for Medication Review
1576581000006116		Tele. Reminder for Medication Review
1576671000006115		Verbal Reminder for Medication Review
158421000000116		Medication review of medical notes
1681361000000119		Medication review done by medicines management technician
1681401000000111		Medication review done by medicines management pharmacist
1753791000006115		Medication review - additional
1796041000000119		Medication review by practice nurse

1875461000006 115		Referral to general practitioner for medication review
1916981000006 111		Dementia medication review
1920671000006 114		Medication review with carer
1983571000006 112		Extended medication review by community pharmacist
2009961000006 117		Medication review done by clinical pharmacist
2010011000006 110		Polypharmacy medication review done by clinical pharmacist
2160114019		Medication review done by doctor
2249791000000 116		Polypharmacy medication review
2276751000000 115		Referral to general practitioner for medication review
2403101000000 116		Dementia medication review
2474282010		Medication review done by nurse
2474283017		Medication review declined
2474691016		Mental health medication review
2724411000000 114		Medication review invitation
282653015		Medication review
2989710000001 15		Medication review done by community pharmacist
4051410000001 16		Medication review done by pharmacy technician
458904017		Medication review due
458905016		Medication review done
7124110000061 17		Medication review
7484741000006 113		Neurological disorder medication review
8454801000006 118		Medication review by community nurse
9188310000061 16		Medication review
9402910000061 11		[RFC] Medication review
9403610000061 13		Medication review done by nurse

Z-drugs

prodcode	productname	dmdid	termfromemis	formulation	routeofadministration	drugsubstance
1386141000033114	Stilnoct 5mg tablets	3206111000001100	Stilnoct 5mg tablets (Sanofi)	Tablet	Oral	Zolpidem tartrate
1387141000033111	Stilnoct 10mg tablets	3199611000001100	Stilnoct 10mg tablets (Sanofi)	Tablet	Oral	Zolpidem tartrate

15579410 00033116	Zimovane LS 3.75mg tablets	178011000 001104	Zimovane LS 3.75mg tablets (Sanofi)	Tablet	Oral	Zopiclon e
15584410 00033110	Zimovane 7.5mg tablets	810511000 001108	Zimovane 7.5mg tablets (Sanofi)	Tablet	Oral	Zopiclon e
15621410 00033113	Zolpidem 5mg tablets	321183000	Zolpidem 5mg tablets	Tablet	Oral	Zolpidem tartrate
15623410 00033111	Zopiclone 3.75mg tablets	321177003	Zopiclone 3.75mg tablets	Tablet	Oral	Zopiclon e
15624410 00033117	Zolpidem 10mg tablets	321184006	Zolpidem 10mg tablets	Tablet	Oral	Zolpidem tartrate
15632410 00033113	Zopiclone 7.5mg tablets	321175006	Zopiclone 7.5mg tablets	Tablet	Oral	Zopiclon e
17135410 00033112	Zileze 3.75 tablets	224011000 001106	Zileze 3.75 tablets (Opus Pharmaceuticals Ltd)	Tablet	Oral	Zopiclon e
17136410 00033113	Zileze 7.5 tablets	562111000 001100	Zileze 7.5 tablets (Opus Pharmaceuticals Ltd)	Tablet	Oral	Zopiclon e
20037410 00033117	Zaleplon 5mg capsules	321190005	Zaleplon 5mg capsules	Capsule	Oral	Zaleplon
20038410 00033110	Zaleplon 10mg capsules	321191009	Zaleplon 10mg capsules	Capsule	Oral	Zaleplon
20039410 00033119	Sonata 5mg capsules	846011000 001109	Sonata 5mg capsules (Meda Pharmaceuticals Ltd)	Capsule	Oral	Zaleplon
20040410 00033117	Sonata 10mg capsules	435711000 001108	Sonata 10mg capsules (Meda Pharmaceuticals Ltd)	Capsule	Oral	Zaleplon
49572410 00033112	Zopiclone 3.75mg/5ml oral suspension	879901100 0001100	Zopiclone 3.75mg/5ml oral suspension	Oral suspens ion	Oral	Zopiclon e
59787410 00033112	Zopiclone 7.5mg/5ml oral suspension	879891100 0001110	Zopiclone 7.5mg/5ml oral suspension	Oral suspens ion	Oral	Zopiclon e
64566410 00033117	Zopiclone 3.75mg/5ml oral solution	196096110 00001100	Zopiclone 3.75mg/5ml oral solution	Oral solution	Oral	Zopiclon e
91779410 00033119	Zopiclone 7.5mg/5ml oral solution	245109110 00001100	Zopiclone 7.5mg/5ml oral solution	Oral solution	Oral	Zopiclon e

Benzodiazepines

prodcode	productname	termfromemis
102264100 0033113		Oxazepam Capsules 30 mg
102574100 0033113	Oxazepam 10mg tablets	Oxazepam 10mg tablets
102584100 0033115	Oxazepam 15mg tablets	Oxazepam 15mg tablets
102594100 0033111		Oxazepam Tablets 30 mg
103880410 00033115	Perizam 1mg/ml oral suspension	Perizam 1mg/ml oral suspension (Rosemont Pharmaceuticals Ltd)

103881410 00033116	Perizam 2mg/ml oral suspension	Perizam 2mg/ml oral suspension (Rosemont Pharmaceuticals Ltd)
112759410 00033119	Oxazepam 10mg/5ml oral suspension	Oxazepam 10mg/5ml oral suspension
115598410 00033118	Midazolam 10mg/2ml oromucosal solution pre-filled oral syringes sugar free	Midazolam 10mg/2ml oromucosal solution pre-filled oral syringes sugar free
115599410 00033114	Midazolam 2.5mg/0.5ml oromucosal solution pre-filled oral syringes sugar free	Midazolam 2.5mg/0.5ml oromucosal solution pre-filled oral syringes sugar free
115600410 00033112	Midazolam 5mg/1ml oromucosal solution pre-filled oral syringes sugar free	Midazolam 5mg/1ml oromucosal solution pre-filled oral syringes sugar free
115601410 00033111	Midazolam 7.5mg/1.5ml oromucosal solution pre-filled oral syringes sugar free	Midazolam 7.5mg/1.5ml oromucosal solution pre-filled oral syringes sugar free
116162410 00033110	Clonazepam 1mg/1ml solution for injection ampoules and diluent	Clonazepam 1mg/1ml solution for injection ampoules and diluent
116163410 00033117	Rivotril 1mg/1ml concentrate for solution for injection ampoules and diluent	Rivotril 1mg/1ml concentrate for solution for injection ampoules and diluent (Imported (France))
116474100 0033111		Remnos Tablets 10 mg
116484100 0033118		Remnos Tablets 5 mg
117334100 0033116		Rivotril Injection 1mg/ml, 1 ml ampoule
117724100 0033117	Rivotril 500microgram tablets	Rivotril 500microgram tablets (Roche Products Ltd)
117734100 0033110	Rivotril 2mg tablets	Rivotril 2mg tablets (Roche Products Ltd)
118414100 0033114	Rohypnol 1mg tablets	Rohypnol 1mg tablets (Roche Products Ltd)
122025410 00033114	Zacco 5mg/5ml oral suspension	Zacco 5mg/5ml oral suspension (Thame Laboratories Ltd)
122026410 00033110	Zacco 10mg/5ml oral suspension	Zacco 10mg/5ml oral suspension (Thame Laboratories Ltd)
123152410 00033112	Midazolam 10mg/1ml oromucosal solution pre-filled oral syringes sugar free	Midazolam 10mg/1ml oromucosal solution pre-filled oral syringes sugar free
123737410 00033114		Lorazepam Oral solution 1 mg/ml (5 mg/5 ml)
125033410 00033119	Lorazepam 1mg/ml oral solution sugar free	Lorazepam 1mg/ml oral solution sugar free
130581410 00033113	Lorazepam 500microgram tablets	Lorazepam 500microgram tablets
131497410 00033113	Midazolam 10mg/5ml oral solution unit dose ampoules sugar free	Midazolam 10mg/5ml oral solution unit dose ampoules sugar free
131498410 00033115	Ozalin 2mg/ml oral solution 5ml unit dose ampoules	Ozalin 2mg/ml oral solution 5ml unit dose ampoules (Primex Pharmaceuticals Oy)
131594410 00033115	Lorazepam 1mg orodispersible tablets	Lorazepam 1mg orodispersible tablets
131595410 00033119	Lorazepam 2.5mg orodispersible tablets	Lorazepam 2.5mg orodispersible tablets
134199410 00033117	Midazolam 100mg/50ml solution for infusion pre-filled syringes	Midazolam 100mg/50ml solution for infusion pre-filled syringes
134200410 00033119	Midazolam 50mg/50ml solution for infusion pre-filled syringes	Midazolam 50mg/50ml solution for infusion pre-filled syringes

135124100 0033119		Solis Capsules 2 mg
135134100 0033112		Solis Capsules 5 mg
136354100 0033112	Somnite 2.5mg/5ml oral suspension	Somnite 2.5mg/5ml oral suspension (Norgine Pharmaceuticals Ltd)
138324100 0033113	Stesolid 10mg rectal tube	Stesolid 10mg rectal tube (Accord Healthcare Ltd)
138334100 0033115	Stesolid 5mg rectal tube	Stesolid 5mg rectal tube (Accord Healthcare Ltd)
138924100 0033111		Surem Capsules 5 mg
141374100 0033113		Temazepam Capsules 10 mg
141384100 0033115		Temazepam Capsules 15 mg
141394100 0033111		Temazepam Capsules 20 mg
141404100 0033113		Temazepam Capsules 30 mg
141674100 0033119		Temazepam Elixir 10 mg/5 ml
141754100 0033113		Temazepam Gelthix Gel-filled capsules 15 mg
141764100 0033114		Temazepam Gelthix Gel-filled capsules 30 mg
141774100 0033117		Temazepam Gelthix Gel-filled capsules 10 mg
141784100 0033110		Temazepam Gelthix Gel-filled capsules 20 mg
141804100 0033116		Temazepam Hard gelatin capsules 10 mg
141814100 0033117		Temazepam Hard gelatin capsules 20 mg
142214100 0033111		Temazepam Planpak
142314100 0033116		Temazepam Single dose vials 10 mg/5 ml
142814100 0033113	Tensium 10mg tablets	Tensium 10mg tablets (DDSA Pharmaceuticals Ltd)
142824100 0033118	Tensium 2mg tablets	Tensium 2mg tablets (DDSA Pharmaceuticals Ltd)
142834100 0033111		Tensium Tablets 5 mg
142844100 0033117	Temazepam 10mg tablets	Temazepam 10mg tablets
142854100 0033116	Temazepam 20mg tablets	Temazepam 20mg tablets
145594100 0033116	Tropium 5mg capsules	Tropium 5mg capsules (Dr Reddy's Laboratories (UK) Ltd)
150294100 0033114	Valclair 10mg suppositories	Valclair 10mg suppositories (Durbin Plc)

1543541000033117	Xanax 250microgram tablets	Xanax 250microgram tablets (Upjohn UK Ltd)
1543641000033116	Xanax 500microgram tablets	Xanax 500microgram tablets (Upjohn UK Ltd)
163341000033112		Bromazepam Tablets 1.5 mg
163441000033118		Bromazepam Tablets 3 mg
1699841000033119	Temazepam 10mg/5ml oral solution sugar free	Temazepam 10mg/5ml oral solution sugar free
1728141000033116	Diazepam 5mg/5ml oral solution	Diazepam 5mg/5ml oral solution
2056341000033116	Midazolam 50mg/50ml solution for infusion vials	Midazolam 50mg/50ml solution for infusion vials
237641000033119		Chlordiazepoxide Hydrochloride Capsules 10 mg
238141000033111	Chlordiazepoxide 5mg capsules	Chlordiazepoxide 5mg capsules
238241000033116	Chlordiazepoxide 10mg capsules	Chlordiazepoxide 10mg capsules
248541000033115		Chlordiazepoxide Hydrochloride Tablets 10 mg
248641000033119		Chlordiazepoxide Hydrochloride Tablets 5 mg
250241000033117		Chlordiazepoxide Tablets 25 mg
250641000033119	Chlordiazepoxide 10mg tablets	Chlordiazepoxide 10mg tablets
250741000033111		Chlordiazepoxide Hydrochloride Tablets 25 mg
250841000033118	Chlordiazepoxide 5mg tablets	Chlordiazepoxide 5mg tablets
2601041000033112	Clonazepam 2.5mg/ml drops sugar free	Clonazepam 2.5mg/ml drops sugar free
2601141000033111	Rivotril 2.5mg/1ml drops	Rivotril 2.5mg/1ml drops (Imported (Switzerland))
2621141000033119	Tropium 10mg capsules	Tropium 10mg capsules (Dr Reddy's Laboratories (UK) Ltd)
2621241000033114	Tropium 5mg tablets	Tropium 5mg tablets (Dr Reddy's Laboratories (UK) Ltd)
2621341000033116	Tropium 10mg tablets	Tropium 10mg tablets (Dr Reddy's Laboratories (UK) Ltd)
262841000033110	Clobazam 10mg tablets	Clobazam 10mg tablets
2640041000033113	Clonazepam 125micrograms/5ml oral suspension	Clonazepam 125micrograms/5ml oral suspension
2667941000033119	Clonazepam 625micrograms/5ml oral suspension	Clonazepam 625micrograms/5ml oral suspension
2711041000033119	Clobazam 5mg/5ml oral solution	Clobazam 5mg/5ml oral solution
2719441000033110	Clonazepam 500micrograms/5ml oral solution	Clonazepam 500micrograms/5ml oral solution

2722341000033116	Clonazepam 500micrograms/5ml oral suspension	Clonazepam 500micrograms/5ml oral suspension
2752741000033119	Clonazepam 2mg/5ml oral solution	Clonazepam 2mg/5ml oral solution
275541000033114		Clonazepam Injection 1mg/ml, 1 ml ampoule
2762341000033113	Diazepam 2mg/5ml oral solution	Diazepam 2mg/5ml oral solution
2762441000033119	Diazepam 10mg/5ml oral suspension	Diazepam 10mg/5ml oral suspension
286741000033118	Clonazepam 500microgram tablets	Clonazepam 500microgram tablets
286841000033111	Clonazepam 2mg tablets	Clonazepam 2mg tablets
2905341000033111		Midazolam Buccal Liquid Sugar-Free 10 mg/ml
3033041000033117	Diazepam 10mg/2ml emulsion for injection ampoules	Diazepam 10mg/2ml emulsion for injection ampoules
3033141000033118	Diazepam 10mg/2ml solution for injection ampoules	Diazepam 10mg/2ml solution for injection ampoules
3135541000033116	Clonazepam 5mg/5ml oral suspension	Clonazepam 5mg/5ml oral suspension
3157541000033114	Clobazam 10mg/5ml oral suspension	Clobazam 10mg/5ml oral suspension
3188341000033110		Diazepam Syrup 5 mg/5 ml
3246441000033111	Klonopin 0.5mg oral lyophilisates	Klonopin 0.5mg oral lyophilisates (Imported (United States))
3308041000033119	Lorazepam 500micrograms/5ml oral suspension	Lorazepam 500micrograms/5ml oral suspension
3345241000033111	Diazepam 2.5mg/5ml oral suspension	Diazepam 2.5mg/5ml oral suspension
3923841000033119	Lorazepam 1mg/5ml oral suspension	Lorazepam 1mg/5ml oral suspension
3929241000033115	Midazolam 100mg/50ml solution for infusion vials	Midazolam 100mg/50ml solution for infusion vials
3956041000033115	Midazolam 5mg/5ml solution for injection ampoules	Midazolam 5mg/5ml solution for injection ampoules
396341000033111	Dalmane 15mg capsules	Dalmane 15mg capsules (Mylan)
396441000033117	Dalmane 30mg capsules	Dalmane 30mg capsules (Mylan)
3979041000033112	Diazepam 25mg/5ml oral solution	Diazepam 25mg/5ml oral solution
3993641000033114	Diazepam 2mg/5ml oral suspension	Diazepam 2mg/5ml oral suspension
41141000033110	Alprazolam 250microgram tablets	Alprazolam 250microgram tablets
41241000033115	Alprazolam 500microgram tablets	Alprazolam 500microgram tablets
4151341000033112	Midazolam 50mg/10ml solution for injection ampoules	Midazolam 50mg/10ml solution for injection ampoules

438241000 033115		Diazepam Capsules 2 mg
438341000 033113		Diazepam Capsules 5 mg
443414100 0033114	Chlordiazepoxide 5mg/5ml oral suspension	Chlordiazepoxide 5mg/5ml oral suspension
443641000 033112		Diazepam Elixir 2 mg/5 ml
446441000 033119	Diazemuls 10mg/2ml emulsion for injection ampoules	Diazemuls 10mg/2ml emulsion for injection ampoules (Accord Healthcare Ltd)
446641000 033117		Diazepam Injection 10 mg
446741000 033114		Diazepam Injection 5 mg/ml
457741000 033114		Diazepam Rectubes 10 mg
457841000 033116		Diazepam Rectubes 5 mg
457941000 033112	Diazepam 10mg RecTubes	Diazepam 10mg RecTubes (Wockhardt UK Ltd)
458041000 033110	Diazepam 2.5mg RecTubes	Diazepam 2.5mg RecTubes (Wockhardt UK Ltd)
458141000 033114		Diazepam Rectubes Rectal tubes 20 mg
458241000 033119	Diazepam 5mg RecTubes	Diazepam 5mg RecTubes (Wockhardt UK Ltd)
458341000 033112	Diazepam 2.5mg/1.25ml rectal solution tube	Diazepam 2.5mg/1.25ml rectal solution tube
458441000 033118		Diazepam Rectal tubes 20 mg
458541000 033117	Diazepam 10mg/2.5ml rectal solution tube	Diazepam 10mg/2.5ml rectal solution tube
458641000 033116	Diazepam 5mg/2.5ml rectal solution tube	Diazepam 5mg/2.5ml rectal solution tube
460041000 033115	Diazepam 10mg suppositories	Diazepam 10mg suppositories
460741000 033117		Diazepam Syrup 2 mg/5 ml
466334100 0033118	Lorazepam 250micrograms/5ml oral suspension	Lorazepam 250micrograms/5ml oral suspension
466741000 033115	Diazepam 10mg tablets	Diazepam 10mg tablets
466841000 033113	Diazepam 2mg tablets	Diazepam 2mg tablets
466941000 033117	Diazepam 5mg tablets	Diazepam 5mg tablets
509054100 0033119	Midazolam 2mg/2ml solution for injection ampoules	Midazolam 2mg/2ml solution for injection ampoules
541494100 0033113	Nitrazepam 5mg/5ml oral suspension	Nitrazepam 5mg/5ml oral suspension
542634100 0033114	Midazolam 2.5mg/0.5ml oromucosal solution pre-filled oral syringes	Midazolam 2.5mg/0.5ml oromucosal solution pre-filled oral syringes

542644100 0033115	Midazolam 5mg/1ml oromucosal solution pre-filled oral syringes	Midazolam 5mg/1ml oromucosal solution pre-filled oral syringes
542654100 0033119	Midazolam 7.5mg/1.5ml oromucosal solution pre-filled oral syringes	Midazolam 7.5mg/1.5ml oromucosal solution pre-filled oral syringes
542664100 0033118	Midazolam 10mg/2ml oromucosal solution pre-filled oral syringes	Midazolam 10mg/2ml oromucosal solution pre-filled oral syringes
542674100 0033110	Buccolam 2.5mg/0.5ml oromucosal solution pre-filled oral syringes	Buccolam 2.5mg/0.5ml oromucosal solution pre-filled oral syringes (Shire Pharmaceuticals Ltd)
542684100 0033117	Buccolam 5mg/1ml oromucosal solution pre-filled oral syringes	Buccolam 5mg/1ml oromucosal solution pre-filled oral syringes (Shire Pharmaceuticals Ltd)
542694100 0033113	Buccolam 7.5mg/1.5ml oromucosal solution pre-filled oral syringes	Buccolam 7.5mg/1.5ml oromucosal solution pre-filled oral syringes (Shire Pharmaceuticals Ltd)
542704100 0033114	Buccolam 10mg/2ml oromucosal solution pre-filled oral syringes	Buccolam 10mg/2ml oromucosal solution pre-filled oral syringes (Shire Pharmaceuticals Ltd)
558241000 033114		Evacalm Tablets 2 mg
558341000 033116		Evacalm Tablets 5 mg
574864100 0033110	Diazepam 2mg/5ml oral solution sugar free	Diazepam 2mg/5ml oral solution sugar free
578341000 033118	Flurazepam 15mg capsules	Flurazepam 15mg capsules
578441000 033112	Flurazepam 30mg capsules	Flurazepam 30mg capsules
589214100 0033113	Clobazam 5mg/5ml oral suspension	Clobazam 5mg/5ml oral suspension
589224100 0033118	Clobazam 10mg/5ml oral solution	Clobazam 10mg/5ml oral solution
589234100 0033111	Lorazepam 500micrograms/5ml oral solution	Lorazepam 500micrograms/5ml oral solution
589244100 0033117	Lorazepam 1mg/5ml oral solution	Lorazepam 1mg/5ml oral solution
589954100 0033113	Clobazam 1mg/5ml oral solution	Clobazam 1mg/5ml oral solution
589964100 0033114	Clobazam 1mg/5ml oral suspension	Clobazam 1mg/5ml oral suspension
589974100 0033117	Clobazam 2.5mg/5ml oral solution	Clobazam 2.5mg/5ml oral solution
589984100 0033110	Clobazam 2.5mg/5ml oral suspension	Clobazam 2.5mg/5ml oral suspension
589994100 0033119	Clobazam 25mg/5ml oral solution	Clobazam 25mg/5ml oral solution
590004100 0033117	Clobazam 25mg/5ml oral suspension	Clobazam 25mg/5ml oral suspension
590104100 0033114	Clonazepam 12.5mg/5ml oral solution	Clonazepam 12.5mg/5ml oral solution
590114100 0033113	Clonazepam 12.5mg/5ml oral suspension	Clonazepam 12.5mg/5ml oral suspension
590124100 0033118	Clonazepam 250micrograms/5ml oral solution	Clonazepam 250micrograms/5ml oral solution
590134100 0033111	Clonazepam 250micrograms/5ml oral suspension	Clonazepam 250micrograms/5ml oral suspension

595341000033118	Flunitrazepam 1mg tablets	Flunitrazepam 1mg tablets
5973041000033115	Midazolam 10mg/5ml oral solution	Midazolam 10mg/5ml oral solution
5973141000033116	Midazolam 10mg/5ml oral suspension	Midazolam 10mg/5ml oral suspension
5973241000033111	Midazolam 12.5mg/5ml oral solution	Midazolam 12.5mg/5ml oral solution
5973341000033118	Midazolam 12.5mg/5ml oral suspension	Midazolam 12.5mg/5ml oral suspension
5973441000033112	Midazolam 5mg/5ml oral solution	Midazolam 5mg/5ml oral solution
5973541000033113	Midazolam 5mg/5ml oral suspension	Midazolam 5mg/5ml oral suspension
5991341000033113	Clonazepam 2mg/5ml oral suspension	Clonazepam 2mg/5ml oral suspension
5991641000033117	Diazepam 10mg/5ml oral solution	Diazepam 10mg/5ml oral solution
5991741000033114	Diazepam 2.5mg/5ml oral solution	Diazepam 2.5mg/5ml oral solution
6001641000033114	Midazolam 10mg/ml oral solution	Midazolam 10mg/ml oral solution
6031941000033113		Xanax Tablets 1 mg
609541000033119	Frisium 10mg tablets	Frisium 10mg tablets (Sanofi)
62241000033118		Anxon Capsules 15 mg
62341000033111		Anxon Capsules 30 mg
6360941000033113	Clobazam 2mg/5ml oral suspension	Clobazam 2mg/5ml oral suspension
6361041000033115	Clobazam 2mg/5ml oral solution	Clobazam 2mg/5ml oral solution
6475441000033114	Midazolam 10mg/1ml oromucosal solution pre-filled oral syringes	Midazolam 10mg/1ml oromucosal solution pre-filled oral syringes
6529041000033112	Clonazepam 2mg/5ml oral solution sugar free	Clonazepam 2mg/5ml oral solution sugar free
6529741000033110	Clonazepam 500micrograms/5ml oral solution sugar free	Clonazepam 500micrograms/5ml oral solution sugar free
736941000033118	Hypnovel 10mg/5ml solution for injection ampoules	Hypnovel 10mg/5ml solution for injection ampoules (Roche Products Ltd)
737041000033117	Hypnovel 10mg/2ml solution for injection ampoules	Hypnovel 10mg/2ml solution for injection ampoules (Neon Healthcare Ltd)
7678541000033116	Epistatus 10mg/1ml oromucosal solution pre-filled oral syringes	Epistatus 10mg/1ml oromucosal solution pre-filled oral syringes (Veriton Pharma Ltd)
8262841000033115	Clobazam 5mg/5ml oral suspension sugar free	Clobazam 5mg/5ml oral suspension sugar free
8262941000033111	Clobazam 10mg/5ml oral suspension sugar free	Clobazam 10mg/5ml oral suspension sugar free
8263041000033118	Tapclob 5mg/5ml oral suspension	Tapclob 5mg/5ml oral suspension (Martindale Pharmaceuticals Ltd)

8263141000033119	Tapclob 10mg/5ml oral suspension	Tapclob 10mg/5ml oral suspension (Martindale Pharmaceuticals Ltd)
826541000033112		Lexotan Tablets 1.5 mg
826641000033113		Lexotan Tablets 3 mg
827841000033112	Librium 10mg capsules	Librium 10mg capsules (Mylan)
827941000033116	Librium 5mg capsules	Librium 5mg capsules (Mylan)
835041000033114		Librium Tablets 10 mg
835141000033113		Librium Tablets 25 mg
835241000033118		Librium Tablets 5 mg
844141000033111	Lorazepam 4mg/1ml solution for injection ampoules	Lorazepam 4mg/1ml solution for injection ampoules
850741000033117	Loprazolam 1mg tablets	Loprazolam 1mg tablets
851141000033111	Lorazepam 1mg tablets	Lorazepam 1mg tablets
851241000033116	Lorazepam 2.5mg tablets	Lorazepam 2.5mg tablets
851341000033114	Lormetazepam 1mg tablets	Lormetazepam 1mg tablets
851441000033115	Lormetazepam 500microgram tablets	Lormetazepam 500microgram tablets
872441000033113		Medazepam Capsules 5 mg
8870741000033111	Clonazepam 2.5mg/ml drops	Clonazepam 2.5mg/ml drops
88941000033110	Ativan 4mg/1ml solution for injection ampoules	Ativan 4mg/1ml solution for injection ampoules (Pfizer Ltd)
91241000033114		Atensine Tablets 10 mg
91341000033116		Atensine Tablets 2 mg
91441000033110		Atensine Tablets 5 mg
914741000033113	Midazolam 10mg/5ml solution for injection ampoules	Midazolam 10mg/5ml solution for injection ampoules
914841000033115	Midazolam 10mg/2ml solution for injection ampoules	Midazolam 10mg/2ml solution for injection ampoules
939441000033114	Mogadon 5mg tablets	Mogadon 5mg tablets (Mylan)
967841000033113		Nitrazepam Capsules 5 mg
972541000033115		Nitrazepam Mixture 150 ml
974341000033116	Nitrazepam 2.5mg/5ml oral suspension	Nitrazepam 2.5mg/5ml oral suspension

975141000 033118		Nitrados Tablets 5 mg
975241000 033113		Nitrazepam Tablets 10 mg
975341000 033115	Nitrazepam 5mg tablets	Nitrazepam 5mg tablets

Anti-psychotics (all)

prodcode	product name	dm did	termfromemis
1003641000 033113			Olanzapine 10mg tablets
1003741000 033116			Olanzapine 5mg tablets
1003841000 033114			Olanzapine 7.5mg tablets
1004114100 0033118			Biquelle XL 50mg tablets (Aspire Pharma Ltd)
1004124100 0033113			Biquelle XL 150mg tablets (Aspire Pharma Ltd)
1004134100 0033115			Biquelle XL 200mg tablets (Aspire Pharma Ltd)
1004144100 0033114			Biquelle XL 300mg tablets (Aspire Pharma Ltd)
1004154100 0033110			Biquelle XL 400mg tablets (Aspire Pharma Ltd)
1004174100 0033119			Zaluron XL 50mg tablets (Fontus Health Ltd)
1004184100 0033112			Zaluron XL 150mg tablets (Fontus Health Ltd)
1004194100 0033116			Zaluron XL 200mg tablets (Fontus Health Ltd)
1004204100 0033110			Zaluron XL 300mg tablets (Fontus Health Ltd)
1004214100 0033114			Zaluron XL 400mg tablets (Fontus Health Ltd)
1016341000 033111			Orap Tablets 10 mg
1016441000 033117			Orap 2mg tablets (Janssen-Cilag Ltd)
1016541000 033116			Orap 4mg tablets (Eumedica Pharmaceuticals)
1022741000 033116			Oxypertine 10mg capsules
1025384100 0033119			Melperone hydrochloride 100mg tablets
1025474100 0033110			Mintreleq XL 50mg tablets (Aristo Pharma Ltd)
1025484100 0033117			Mintreleq XL 150mg tablets (Aristo Pharma Ltd)
1025494100 0033113			Mintreleq XL 200mg tablets (Aristo Pharma Ltd)

1025504100 0033113			Mintreleq XL 300mg tablets (Aristo Pharma Ltd)
1025514100 0033112			Mintreleq XL 400mg tablets (Aristo Pharma Ltd)
1026941000 033117			Oxypertine 40mg tablets
1049334100 0033115			Haloperidol 500micrograms/5ml oral solution
1049344100 0033114			Haloperidol 500micrograms/5ml oral suspension
1052041000 033118			Perphenazine Injection 5 mg/ml
1061514100 0033115			Atrolak XL 50mg tablets (Accord Healthcare Ltd)
1061524100 0033110			Atrolak XL 200mg tablets (Accord Healthcare Ltd)
1061534100 0033117			Atrolak XL 300mg tablets (Accord Healthcare Ltd)
1061544100 0033111			Atrolak XL 400mg tablets (Accord Healthcare Ltd)
1063541000 033113			Pericyazine 10mg/5ml oral solution
1065141000 033117			Perphenazine 2mg tablets
1065241000 033112			Perphenazine 4mg tablets
1065341000 033119			Perphenazine Tablets 8 mg
1066741000 033111			Pericyazine 10mg tablets
1067041000 033110			Pericyazine 2.5mg tablets
1067141000 033114			Pericyazine Tablets 25 mg
1086741000 033114			Piportil Depot 50mg/1ml solution for injection ampoules (Sanofi)
1087341000 033110			Piportil Depot 100mg/2ml solution for injection ampoules (Sanofi)
1090841000 033118			Pimozide Tablets 10 mg
1090941000 033114			Pimozide 2mg tablets
1091041000 033116			Pimozide 4mg tablets
1094634100 0033116			Aripiprazole 400mg powder and solvent for suspension for injection pre-filled syringes
1094644100 0033110			Abilify Maintena 400mg powder and solvent for prolonged-release suspension for injection pre-filled syringes (Otsuka Pharmaceuticals (U.K.) Ltd)
1117541000 033112			Promazine Hydrochloride Injection 50 mg/ml
1129141000 033118			Promazine Hydrochloride Suspension 50 mg/5 ml

1129641000 033111			Promazine 25mg/5ml oral solution
1134241000 033114			Promazine Hydrochloride Tablets 100 mg
1134341000 033116			Promazine 25mg tablets
1134441000 033110			Promazine 50mg tablets
1150041000 033111			Quetiapine 100mg tablets
1150141000 033110			Quetiapine 200mg tablets
1150241000 033115			Quetiapine 25mg tablets
1150341000 033113			Quetiapine Fumarate Tablets (starter pack) 6 x 25 mg, 2 x 100 mg
1157894100 0033118			Psyquet XL 50mg tablets (Sandoz Ltd)
1157924100 0033119			Psyquet XL 150mg tablets (Sandoz Ltd)
1157934100 0033112			Psyquet XL 200mg tablets (Sandoz Ltd)
1157944100 0033118			Psyquet XL 300mg tablets (Sandoz Ltd)
1157954100 0033117			Psyquet XL 400mg tablets (Sandoz Ltd)
1158141000 033113			Redeptin Injection 2 mg/ ml
1158374100 0033116			Loxapine 9.1mg/dose inhalation powder
1158384100 0033114			Adasuve 9.1mg/dose inhalation powder (Galen Ltd)
1161241000 033115			Remoxipride Hydrochloride M/R capsules 150 mg
1161341000 033113			Remoxipride Hydrochloride M/R capsules 300 mg
1169434100 0033111			Paliperidone 175mg/0.875ml prolonged-release suspension for injection pre-filled syringes
1169444100 0033117			Paliperidone 263mg/1.315ml prolonged-release suspension for injection pre-filled syringes
1169454100 0033116			Paliperidone 350mg/1.75ml prolonged-release suspension for injection pre-filled syringes
1169464100 0033115			Paliperidone 525mg/2.625ml prolonged-release suspension for injection pre-filled syringes
1169474100 0033112			Trevicta 175mg/0.875ml prolonged-release suspension for injection pre-filled syringes (Janssen-Cilag Ltd)
1169484100 0033119			Trevicta 263mg/1.315ml prolonged-release suspension for injection pre-filled syringes (Janssen-Cilag Ltd)
1169494100 0033110			Trevicta 350mg/1.75ml prolonged-release suspension for injection pre-filled syringes (Janssen-Cilag Ltd)
1169504100 0033110			Trevicta 525mg/2.625ml prolonged-release suspension for injection pre-filled syringes (Janssen-Cilag Ltd)

1173004100 0033115			Quetiapine 200mg/5ml oral suspension
1173841000 033113			Risperdal 1mg/ml oral solution (Janssen-Cilag Ltd)
1173941000 033117			Risperidone 1mg/ml oral solution sugar free
1175364100 0033118			Quetiapine 20mg/ml oral suspension sugar free
1176241000 033115			Risperdal 1mg tablets (Janssen-Cilag Ltd)
1176341000 033113			Risperdal 2mg tablets (Janssen-Cilag Ltd)
1176441000 033119			Risperdal 3mg tablets (Janssen-Cilag Ltd)
1176541000 033118			Risperdal 4mg tablets (Janssen-Cilag Ltd)
1176641000 033117			Risperidone 1mg tablets
1176741000 033114			Risperidone 2mg tablets
1176841000 033116			Risperidone 3mg tablets
1176941000 033112			Risperidone 4mg tablets
1177641000 033119			Risperdal 6mg tablets (Janssen-Cilag Ltd)
1177741000 033111			Risperidone 6mg tablets
1178194100 0033118			Brancico XL 150mg tablets (Zentiva)
1178204100 0033112			Brancico XL 200mg tablets (Zentiva)
1178214100 0033111			Brancico XL 300mg tablets (Zentiva)
1178224100 0033116			Brancico XL 400mg tablets (Zentiva)
1178234100 0033114			Brancico XL 50mg tablets (Zentiva)
1179884100 0033118			Pimozide 2mg/5ml oral suspension
1182154100 0033119			Levomepromazine 62.5mg/5ml oral suspension
1187674100 0033116			Chlorpromazine 25mg/5ml oral solution sugar free
1242804100 0033114			Atrolak XL 150mg tablets (Accord Healthcare Ltd)
1266534100 0033110			Cariprazine 1.5mg capsules
1266544100 0033116			Cariprazine 3mg capsules
1266554100 0033115			Cariprazine 4.5mg capsules

1266564100 0033119			Cariprazine 6mg capsules
1266574100 0033111			Reagila 1.5mg capsules (Recordati Pharmaceuticals Ltd)
1266584100 0033118			Reagila 3mg capsules (Recordati Pharmaceuticals Ltd)
1266594100 0033114			Reagila 4.5mg capsules (Recordati Pharmaceuticals Ltd)
1266604100 0033116			Reagila 6mg capsules (Recordati Pharmaceuticals Ltd)
1266764100 0033113			Chlorpromazine 100mg suppositories
1268341000 033116			Serenace 500microgram capsules (Teva UK Ltd)
1269641000 033115			Serenace Elixir 2 mg/5 ml
1269741000 033112			Serenace 2mg/ml liquid (Teva UK Ltd)
1271541000 033112			Serenace 20mg/2ml solution for injection ampoules (IVAX Pharmaceuticals UK Ltd)
1271641000 033113			Serenace 5mg/1ml solution for injection ampoules (IVAX Pharmaceuticals UK Ltd)
1275941000 033115			Serenace 1.5mg tablets (Teva UK Ltd)
1276041000 033113			Serenace 10mg tablets (Teva UK Ltd)
1276141000 033112			Serenace 20mg tablets (Teva UK Ltd)
1276241000 033117			Serenace 5mg tablets (Teva UK Ltd)
1276841000 033118			Serdolect 12mg tablets (Lundbeck Ltd)
1276941000 033114			Serdolect 16mg tablets (Lundbeck Ltd)
1277041000 033110			Serdolect 20mg tablets (Lundbeck Ltd)
1277141000 033114			Serdolect 4mg tablets (Lundbeck Ltd)
1277241000 033119			Sertindole 12mg tablets
1277341000 033112			Sertindole 16mg tablets
1277441000 033118			Sertindole 20mg tablets
1277541000 033117			Sertindole 4mg tablets
1277641000 033116			Seroquel 100mg tablets (AstraZeneca UK Ltd)
1277741000 033113			Seroquel 200mg tablets (AstraZeneca UK Ltd)
1277841000 033115			Seroquel 25mg tablets (AstraZeneca UK Ltd)

1277941000 033111			Seroquel Tablets (starter pack) 6 x 25 mg, 2 x 100 mg
1366641000 033118			Solian 200 tablets (Sanofi)
1366741000 033110			Solian 50 tablets (Sanofi)
1370441000 033112			Sparine Injection 50 mg/ml
1372741000 033116			Sparine Suspension 50 mg/5 ml
1373141000 033110			Sparine Tablets 100 mg
1373241000 033115			Sparine Tablets 25 mg
1373341000 033113			Sparine Tablets 50 mg
1379241000 033117			Stelazine Injection 1 mg/ml
1381441000 033113			Stelazine Liquid 10mg/ml
1385341000 033116			Stelazine 10mg Spansules (Mercury Pharma Group Ltd)
1385441000 033110			Stelazine 15mg Spansules (Mercury Pharma Group Ltd)
1385541000 033111			Stelazine 2mg Spansules (Mercury Pharma Group Ltd)
1385941000 033117			Stelazine 1mg/5ml syrup (Mercury Pharma Group Ltd)
1386341000 033112			Stelazine 1mg tablets (Mercury Pharma Group Ltd)
1386441000 033118			Stelazine 5mg tablets (Mercury Pharma Group Ltd)
1396441000 033117			Sulpiride 200mg tablets
1396541000 033116			Sulpitil 200mg tablets (Pfizer Ltd)
1397641000 033117			Sulpiride 400mg tablets
1432341000 033111			Thioridazine 100mg/5ml oral suspension
1432441000 033117			Thioridazine 25mg/5ml oral suspension
1432541000 033116			Thioridazine 25mg/5ml oral solution
1432741000 033112			Thioridazine Syrup 12.5mg/5 ml
1433641000 033111			Thioridazine 10mg tablets
1433741000 033119			Thioridazine 100mg tablets
1433841000 033112			Thioridazine 25mg tablets

1433941000 033116			Thioridazine 50mg tablets
1451410000 33119			Benperidol 250microgram tablets
1459841000 033114			Trifluoperazine Injection 1 mg/ml
1462041000 033119			Trifluoperazine Liquid 10mg/ml
1464141000 033113			Trifluoperazine 5mg/5ml oral solution sugar free
1466941000 033117			Trifluoperazine 10mg modified-release capsules
1467041000 033116			Trifluoperazine 15mg modified-release capsules
1467141000 033117			Trifluoperazine 2mg modified-release capsules
1467641000 033110			Trifluoperazine 1mg/5ml oral solution sugar free
1470641000 033116			Trifluoperazine 1mg tablets
1470741000 033113			Trifluoperazine 5mg tablets
1471641000 033112			Triperidol Tablets 1 mg
1471741000 033115			Triperidol Tablets 500 mcg
1473341000 033117			Triptafen tablets (Advanz Pharma)
1473441000 033111			Triptafen-M tablets (Mercury Pharma Group Ltd)
1473741000 033116			Trifluperidol Tablets 1 mg
1474541000 033114			Trifluperidol Tablets 500 mcg
1512741000 033113			Nozinan 25mg tablets (Sanofi)
1563441000 033114			Zuclopenthixol acetate 100mg/2ml solution for injection ampoules
1563541000 033110			Zuclopenthixol acetate 50mg/1ml solution for injection ampoules
1563641000 033111			Zuclopenthixol decanoate 200mg/1ml solution for injection ampoules
1563741000 033119			Zuclopenthixol decanoate 500mg/1ml solution for injection ampoules
1564041000 033119			Zuclopenthixol 10mg tablets
1564141000 033115			Zuclopenthixol 2mg tablets
1564241000 033110			Zuclopenthixol 25mg tablets
1565141000 033119			Zyprexa 10mg tablets (Eli Lilly and Company Ltd)

1565241000 033114			Zyprexa 5mg tablets (Eli Lilly and Company Ltd)
1565341000 033116			Zyprexa 7.5mg tablets (Eli Lilly and Company Ltd)
1661141000 033114			Olanzapine 2.5mg tablets
1698441000 033110			Sulpiride 200mg/5ml oral solution sugar free
1713941000 033118			Zyprexa 2.5mg tablets (Eli Lilly and Company Ltd)
1719341000 033115			Chlorpromazine 100mg/5ml oral solution
1765741000 033116			Zoleptil 100 tablets (Movianto UK Ltd)
1765841000 033114			Zoleptil 25 tablets (Movianto UK Ltd)
1765941000 033118			Zoleptil 50 tablets (Movianto UK Ltd)
1766041000 033111			Zotepine 100mg tablets
1766141000 033110			Zotepine 25mg tablets
1766241000 033115			Zotepine 50mg tablets
1832841000 033113			Quetiapine Fumarate Tablets (starter pack) 6x25 mg, 2x100 mg, 2x150 mg
1832941000 033117			Seroquel Tablets (starter pack) 6x25 mg, 2x100 mg, 2x150 mg
1844641000 033118			Quetiapine 150mg tablets
1844741000 033110			Seroquel 150mg tablets (AstraZeneca UK Ltd)
2076441000 033112			Sulparex Tablets 200 mg
2077741000 033115			Olanzapine 5mg oral lyophilisates sugar free
2077841000 033113			Olanzapine 10mg oral lyophilisates sugar free
2077941000 033117			Zyprexa 5mg Velotabs (Eli Lilly and Company Ltd)
2078041000 033119			Zyprexa 10mg Velotabs (Eli Lilly and Company Ltd)
2188141000 033115			Risperdal 500microgram tablets (Janssen-Cilag Ltd)
2188241000 033110			Risperidone 500microgram tablets
2216641000 033113			Amisulpride 400mg tablets
2216741000 033116			Solian 400 tablets (Sanofi)
2400410000 33110			Chlorpromazine 25mg/5ml oral solution

2413410000 33116			Chlorpromazine 50mg/2ml solution for injection ampoules
2422410000 33115			Chlorpromazine Hydrochloride Injection 100 mg/5 ml
2423410000 33113			Chlorpromazine Hydrochloride Injection 25 mg/ml
2465410000 33119			Chlorpromazine Hydrochloride Suppositories 100 mg
2466410000 33118			Chlorpromazine 100mg/5ml oral suspension sugar free
2472410000 33118			Chlorpromazine Hydrochloride Syrup 25 mg/5 ml
2490410000 33117			Chlorpromazine 10mg tablets
2491410000 33118			Chlorpromazine 100mg tablets
2492410000 33113			Chlorpromazine 25mg tablets
2493410000 33115			Chlorpromazine 50mg tablets
2511410000 33117			Chloractil Tablets 100 mg
2512410000 33112			Chloractil Tablets 25 mg
2513410000 33119			Chloractil Tablets 50 mg
2640141000 033112			Promazine 50mg/5ml oral solution
2644841000 033114			Amisulpride 100mg/ml oral solution sugar free
2644941000 033118			Solian 100mg/ml oral solution (Sanofi)
2653741000 033117			Methotrimeprazine Maleate Tablets 6 mg
2653841000 033110			Levinan 6mg tablets (Kyowa Kirin Ltd)
2658941000 033110			Sulpor 200mg/5ml oral solution (Rosemont Pharmaceuticals Ltd)
2730641000 033111			Promazine Hydrochloride Syrup 50 mg/5 ml
2736410000 33114			Clopixol Acuphase 50mg/1ml solution for injection ampoules (Lundbeck Ltd)
2748410000 33112			Clopixol Acuphase 100mg/2ml solution for injection ampoules (Lundbeck Ltd)
2758141000 033116			Haloperidol Oral Solution, Sugar Free 1 mg/5 ml
2759641000 033117			Olanzapine 15mg tablets
2759741000 033114			Olanzapine 15mg oral lyophilisates sugar free
2759841000 033116			Zyprexa 15mg tablets (Eli Lilly and Company Ltd)

2759941000033112			Zyprexa 15mg Velotabs (Eli Lilly and Company Ltd)
276041000033113			Clopixol 200mg/1ml solution for injection ampoules (Lundbeck Ltd)
276141000033112			Clopixol Conc 500mg/1ml solution for injection ampoules (Lundbeck Ltd)
2779841000033118			Risperdal Consta 25mg powder and solvent for suspension for injection vials (Janssen-Cilag Ltd)
2779941000033114			Risperdal Consta 37.5mg powder and solvent for suspension for injection vials (Janssen-Cilag Ltd)
2780041000033113			Risperdal Consta 50mg powder and solvent for suspension for injection vials (Janssen-Cilag Ltd)
2780141000033112			Risperidone 25mg powder and solvent for suspension for injection vials
2780241000033117			Risperidone 37.5mg powder and solvent for suspension for injection vials
2780341000033110			Risperidone 50mg powder and solvent for suspension for injection vials
2835141000033114			Amisulpride 100mg tablets
2835241000033119			Solian 100 tablets (Sanofi)
2846441000033114			Benquil 250microgram tablets (Concord Pharmaceuticals Ltd)
284941000033113			Clozaril 25mg tablets (Mylan)
285041000033113			Clozaril 100mg tablets (Mylan)
2868841000033116			Risperidone 1mg orodispersible tablets sugar free
2868941000033112			Risperidone 2mg orodispersible tablets sugar free
2869041000033115			Risperdal Quicklet 1mg orodispersible tablets (Janssen-Cilag Ltd)
2869141000033116			Risperdal Quicklet 2mg orodispersible tablets (Janssen-Cilag Ltd)
286941000033115			Clopixol 10mg tablets (Lundbeck Ltd)
287041000033119			Clopixol 2mg tablets (Lundbeck Ltd)
287141000033115			Clopixol 25mg tablets (Lundbeck Ltd)
287541000033112			Clozapine 100mg tablets
287641000033113			Clozapine 25mg tablets
2929741000033118			Haloperidol 5mg/1ml solution for injection ampoules
2929841000033111			Haldol 5mg/1ml solution for injection ampoules (Janssen-Cilag Ltd)
2955541000033113			Terrazine 1mg tablets (Dr Reddy's Laboratories (UK) Ltd)

2955641000033114			Terrazine 5mg tablets (Dr Reddy's Laboratories (UK) Ltd)
2975841000033113			Quetiapine 300mg tablets
2975941000033117			Seroquel 300mg tablets (AstraZeneca UK Ltd)
3030841000033118			Olanzapine 10mg powder for solution for injection vials
3030941000033114			Zyprexa 10mg powder for solution for injection vials (Eli Lilly and Company Ltd)
3063441000033119			Levomepromazine 25mg/1ml solution for injection ampoules
3063541000033118			Levomepromazine 25mg tablets
3063641000033117			Levomepromazine 6mg tablets
3090541000033111			Flupentixol 50mg/0.5ml solution for injection ampoules
3090641000033112			Flupentixol 100mg/1ml solution for injection ampoules
3090741000033115			Flupentixol 200mg/1ml solution for injection ampoules
3090841000033113			Flupentixol 20mg/1ml solution for injection ampoules
3090941000033117			Flupentixol 40mg/2ml solution for injection ampoules
3091041000033110			Flupentixol 1mg tablets
3091141000033114			Flupentixol 500microgram tablets
3091241000033119			Flupentixol 3mg tablets
3103841000033110			Pipotiazine 100mg/2ml solution for injection ampoules
3104041000033117			Pipotiazine 50mg/1ml solution for injection ampoules
3137541000033113			Aripiprazole 10mg tablets
3137641000033114			Aripiprazole 15mg tablets
3137741000033117			Aripiprazole 30mg tablets
3137841000033110			Abilify 10mg tablets (Otsuka Pharmaceuticals (U.K.) Ltd)
3137941000033119			Abilify 15mg tablets (Otsuka Pharmaceuticals (U.K.) Ltd)
3138041000033116			Abilify 30mg tablets (Otsuka Pharmaceuticals (U.K.) Ltd)
3141141000033114			Stelazine Forte 1mg/ml oral solution (Mercury Pharma Group Ltd)
3158941000033116			Denzapine 25mg tablets (Britannia Pharmaceuticals Ltd)

3159041000 033113			Denzapine 100mg tablets (Britannia Pharmaceuticals Ltd)
3179941000 033111			Nortriptyline 10mg / Fluphenazine 500microgram tablets
3180441000 033118			Amitriptyline 25mg / Perphenazine 2mg tablets
3180541000 033117			Amitriptyline 10mg / Perphenazine 2mg tablets
3225241000 033118			Aripiprazole 5mg tablets
3225341000 033111			Abilify 5mg tablets (Otsuka Pharmaceuticals (U.K.) Ltd)
3226341000 033115			Zaponex 25mg tablets (Leyden Delta B.V.)
3226441000 033114			Zaponex 100mg tablets (Leyden Delta B.V.)
3246041000 033119			Risperidone 500microgram orodispersible tablets sugar free
3246141000 033115			Risperdal Quicklet 500microgram orodispersible tablets (Janssen-Cilag Ltd)
3849441000 033112			Chlorpromazine 50mg/5ml oral solution
3858941000 033110			Chlorpromazine 25mg/1ml solution for injection ampoules
3866541000 033110			Olanzapine 20mg oral lyophilisates sugar free
3866641000 033111			Zyprexa 20mg Velotabs (Eli Lilly and Company Ltd)
3892541000 033114			Olanzapine 2.5mg/5ml oral suspension
3950341000 033115			Aripiprazole 10mg orodispersible tablets sugar free
3950441000 033114			Aripiprazole 15mg orodispersible tablets sugar free
3950541000 033110			Aripiprazole 1mg/ml oral solution
3950641000 033111			Abilify 10mg orodispersible tablets (Otsuka Pharmaceuticals (U.K.) Ltd)
3950741000 033119			Abilify 15mg orodispersible tablets (Otsuka Pharmaceuticals (U.K.) Ltd)
3950841000 033112			Abilify 1mg/ml oral solution (Otsuka Pharmaceuticals (U.K.) Ltd)
3996241000 033117			Olanzapine 20mg tablets
3996341000 033110			Zyprexa 20mg tablets (Eli Lilly and Company Ltd)
4012041000 033116			Risperidone 3mg orodispersible tablets sugar free
4012141000 033117			Risperidone 4mg orodispersible tablets sugar free
4012241000 033112			Risperdal Quicklet 3mg orodispersible tablets (Janssen-Cilag Ltd)

4012341000033119			Risperdal Quicklet 4mg orodispersible tablets (Janssen-Cilag Ltd)
4133841000033118			Quetiapine Oral Suspension Sugar Free 12.5 mg/5 ml
4148441000033110			Chlorpromazine Capsules 10 mg
419941000033113			Depixol Low Volume 200mg/1ml solution for injection ampoules (Lundbeck Ltd)
420041000033111			Depixol Injection 200 mg/10 ml
4200641000033118			Paliperidone 3mg modified-release tablets
4200741000033110			Paliperidone 6mg modified-release tablets
4200841000033117			Paliperidone 9mg modified-release tablets
4200941000033113			Invega 3mg modified-release tablets (Janssen-Cilag Ltd)
4201041000033115			Invega 6mg modified-release tablets (Janssen-Cilag Ltd)
4201141000033116			Invega 9mg modified-release tablets (Janssen-Cilag Ltd)
420141000033110			Depixol 40mg/2ml solution for injection ampoules (Lundbeck Ltd)
420241000033115			Depixol Injection (syringe) 20 mg/1 ml
420341000033113			Depixol Injection (syringe) 40 mg/2 ml
420441000033119			Depixol Conc 50mg/0.5ml solution for injection ampoules (Lundbeck Ltd)
420541000033118			Depixol Conc. Injection 500 mg/5 ml
422941000033113			Depixol 20mg/1ml solution for injection ampoules (Lundbeck Ltd)
423041000033115			Depixol Conc 100mg/1ml solution for injection ampoules (Lundbeck Ltd)
431741000033118			Depixol 3mg tablets (Lundbeck Ltd)
4422541000033110			Quetiapine 100mg/5ml oral suspension
4424541000033119			Haloperidol 5mg/5ml oral solution
4456741000033117			Aripiprazole 9.75mg/1.3ml solution for injection vials
4456841000033110			Abilify 9.75mg/1.3ml solution for injection vials (Otsuka Pharmaceuticals (U.K.) Ltd)
4591741000033114			Quetiapine 50mg modified-release tablets
4591841000033116			Quetiapine 200mg modified-release tablets
4591941000033112			Quetiapine 300mg modified-release tablets

4592041000 033118			Quetiapine 400mg modified-release tablets
4592141000 033119			Seroquel XL 50mg tablets (AstraZeneca UK Ltd)
4592241000 033114			Seroquel XL 200mg tablets (AstraZeneca UK Ltd)
4592341000 033116			Seroquel XL 300mg tablets (AstraZeneca UK Ltd)
4592441000 033110			Seroquel XL 400mg tablets (AstraZeneca UK Ltd)
4765410000 33119			Dozic 5mg/5ml oral solution (Rosemont Pharmaceuticals Ltd)
4766410000 33118			Dozic Liquid 2mg/ml
4798410000 33112			Dolmatil 400mg tablets (Sanofi)
4809410000 33116			Dolmatil 200mg tablets (Sanofi)
4831410000 33112			Droperidol Injection 5mg/ml
4835410000 33115			Droperidol Liquid 1mg/ml
4850410000 33112			Droperidol Tablets 10 mg
4940141000 033119			Amisulpride 25mg/5ml oral solution
5054641000 033118			Haloperidol 1mg/5ml oral solution
5055241000 033117			Droperidol 2.5mg/1ml solution for injection ampoules
5055341000 033110			Xomolix 2.5mg/1ml solution for injection ampoules (Kyowa Kirin Ltd)
5077641000 033110			Clozapine 50mg/ml oral suspension sugar free
5077741000 033118			Denzapine 50mg/ml oral suspension (Britannia Pharmaceuticals Ltd)
5077941000 033115			Clozapine 200mg tablets
5078041000 033117			Clozapine 50mg tablets
5078141000 033118			Denzapine 200mg tablets (Britannia Pharmaceuticals Ltd)
5078241000 033113			Denzapine 50mg tablets (Britannia Pharmaceuticals Ltd)
5565041000 033117			Zypadhera 210mg powder and solvent for suspension for injection vials (Eli Lilly and Company Ltd)
5565141000 033118			Zypadhera 300mg powder and solvent for suspension for injection vials (Eli Lilly and Company Ltd)
5565241000 033113			Zypadhera 405mg powder and solvent for suspension for injection vials (Eli Lilly and Company Ltd)
5575741000 033110			Olanzapine embonate 210mg powder and solvent for suspension for injection vials

5575841000 033117			Olanzapine embonate 300mg powder and solvent for suspension for injection vials
5575941000 033113			Olanzapine embonate 405mg powder and solvent for suspension for injection vials
5671410000 33111			Fentazin Injection 5 mg/ml
5709410000 33117			Fentazin 2mg tablets (Advanz Pharma)
5710410000 33110			Fentazin 4mg tablets (Advanz Pharma)
5711410000 33114			Fentazin Tablets 8 mg
5814341000 033116			Levomepromazine 2.5mg/5ml oral suspension
5820410000 33112			Fluphenazine decanoate 25mg/1ml solution for injection pre-filled syringes
5821410000 33111			Fluphenazine decanoate 50mg/2ml solution for injection pre-filled syringes
5846410000 33111			Fluphenazine decanoate 25mg/1ml solution for injection ampoules
5847410000 33119			Fluphenazine Enanthate Injection 25 mg/ml
5848410000 33112			Fluspirilene Injection 2 mg/ ml
5866410000 33119			Fluphenazine decanoate 12.5mg/0.5ml solution for injection ampoules
5867410000 33111			Fluphenazine decanoate 250mg/10ml solution for injection vials
5868410000 33118			Fluphenazine decanoate 50mg/2ml solution for injection ampoules
5869410000 33114			Fluphenazine decanoate 50mg/0.5ml solution for injection ampoules
5877410000 33113			Fluphenazine decanoate 100mg/1ml solution for injection ampoules
5889741000 033118			Quetiapine 12.5mg/5ml oral solution
5889841000 033111			Quetiapine 12.5mg/5ml oral suspension
5890741000 033111			Haloperidol 1mg/5ml oral suspension
5890941000 033114			Amisulpride 25mg/5ml oral suspension
5891141000 033117			Quetiapine 100mg/5ml oral solution
5894100003 3117			Amisulpride 200mg tablets
5904100003 3114			Amisulpride 50mg tablets
5909341000 033112			Quetiapine 150mg modified-release tablets
5909441000 033118			Seroquel XL 150mg tablets (AstraZeneca UK Ltd)

5934410000 33111			Fluanxol 1mg tablets (Lundbeck Ltd)
5935410000 33112			Fluanxol 500microgram tablets (Lundbeck Ltd)
5954410000 33112			Fluphenazine 1mg tablets
5956410000 33114			Fluphenazine 2.5mg tablets
5957410000 33117			Fluphenazine 5mg tablets
5969441000 033117			Haloperidol 1.5mg/5ml oral solution
5969541000 033116			Haloperidol 1.5mg/5ml oral suspension
5969641000 033115			Haloperidol 250micrograms/5ml oral solution
5969741000 033112			Haloperidol 250micrograms/5ml oral suspension
5974841000 033112			Quetiapine 25mg/5ml oral solution
5974941000 033116			Quetiapine 25mg/5ml oral suspension
5975041000 033116			Quetiapine 50mg/5ml oral solution
5975141000 033117			Quetiapine 50mg/5ml oral suspension
5993541000 033117			Haloperidol 5mg/5ml oral suspension
5997341000 033118			Olanzapine 2.5mg/5ml oral solution
6077410000 33117			Fortunan Tablets 1.5 mg
6078410000 33110			Fortunan Tablets 10 mg
6079410000 33119			Fortunan Tablets 5 mg
6080410000 33116			Fortunan Tablets 500 micrograms
6359441000 033116			Paliperidone 50mg/0.5ml suspension for injection pre-filled syringes
6359541000 033115			Paliperidone 75mg/0.75ml suspension for injection pre-filled syringes
6359641000 033119			Paliperidone 100mg/1ml suspension for injection pre-filled syringes
6359741000 033111			Paliperidone 150mg/1.5ml suspension for injection pre-filled syringes
6359841000 033118			Xeplion 50mg/0.5ml suspension for injection pre-filled syringes (Janssen-Cilag Ltd)
6359941000 033114			Xeplion 75mg/0.75ml suspension for injection pre-filled syringes (Janssen-Cilag Ltd)
6360041000 033112			Xeplion 100mg/1ml suspension for injection pre-filled syringes (Janssen-Cilag Ltd)

6360141000033111			Xeplion 150mg/1.5ml suspension for injection pre-filled syringes (Janssen-Cilag Ltd)
6443041000033115			Asenapine 5mg sublingual tablets sugar free
6443141000033116			Asenapine 10mg sublingual tablets sugar free
6443241000033111			Sycrest 5mg sublingual tablets (Lundbeck Ltd)
6443341000033118			Sycrest 10mg sublingual tablets (Lundbeck Ltd)
6445641000033110			Levomepromazine 6mg/5ml oral suspension
6455741000033116			Ziprasidone 40mg capsules
6455841000033114			Ziprasidone 80mg capsules
6456741000033114			Zalasta 10mg tablets (Consilient Health Ltd)
6456841000033116			Zalasta 15mg tablets (Consilient Health Ltd)
6456941000033112			Zalasta 2.5mg tablets (Consilient Health Ltd)
6457041000033113			Zalasta 20mg tablets (Consilient Health Ltd)
6457141000033112			Zalasta 5mg tablets (Consilient Health Ltd)
6457241000033117			Zalasta 7.5mg tablets (Consilient Health Ltd)
6465341000033114			Zalasta 10mg orodispersible tablets (Consilient Health Ltd)
6465441000033115			Zalasta 15mg orodispersible tablets (Consilient Health Ltd)
6465541000033119			Zalasta 20mg orodispersible tablets (Consilient Health Ltd)
6465641000033118			Zalasta 5mg orodispersible tablets (Consilient Health Ltd)
6465941000033113			Olanzapine 5mg orodispersible tablets sugar free
6466041000033115			Olanzapine 10mg orodispersible tablets sugar free
6466141000033116			Olanzapine 15mg orodispersible tablets sugar free
6466241000033111			Olanzapine 20mg orodispersible tablets sugar free
6514941000033116			Arkolamyl 5mg orodispersible tablets (Mylan)
6515041000033116			Arkolamyl 10mg orodispersible tablets (Mylan)
6515141000033117			Arkolamyl 15mg orodispersible tablets (Mylan)
6515241000033112			Arkolamyl 20mg orodispersible tablets (Mylan)

6583410000 33110			Haloperidol 500microgram capsules
6592410000 33113			Haloperidol Elixir 2 mg/5 ml
6594410000 33114			Haldol decanoate 100mg/1ml solution for injection ampoules (Janssen-Cilag Ltd)
6595410000 33110			Haldol decanoate 50mg/1ml solution for injection ampoules (Janssen-Cilag Ltd)
6596410000 33111			Haloperidol 20mg/2ml solution for injection ampoules
6597410000 33119			Haloperidol decanoate 100mg/1ml solution for injection ampoules
6598410000 33112			Haloperidol decanoate 50mg/1ml solution for injection ampoules
6599410000 33116			Haloperidol Injection 5 mg/ml
6600410000 33118			Haldol Injection 5 mg/ml
6603410000 33116			Haloperidol 5mg/5ml oral solution sugar free
6604410000 33110			Haloperidol Liquid 2mg/ml
6613410000 33112			Haloperidol 10mg/5ml oral solution sugar free
6614410000 33118			Haloperidol Oral liquid conc. 10 mg/ml
6615410000 33117			Haldol 2mg/ml oral solution (Janssen-Cilag Ltd)
6616410000 33116			Haldol Oral liquid conc. 10 mg/ml
6623410000 33115			Haloperidol 1.5mg tablets
6624410000 33114			Haloperidol 20mg tablets
6625410000 33110			Haloperidol 5mg tablets
6632410000 33118			Haloperidol 10mg tablets
6633410000 33111			Haloperidol 500microgram tablets
6634410000 33117			Haldol 10mg tablets (Janssen-Cilag Ltd)
6635410000 33116			Haldol 5mg tablets (Janssen-Cilag Ltd)
6904100003 3115			Anquil 250microgram tablets (Kyowa Kirin Ltd)
7575410000 33111			Integrin 10mg capsules (Sterling-Winthrop)
7681641000 033116			Olanzapine 10mg orodispersible tablets
7681741000 033113			Olanzapine 15mg orodispersible tablets

7681841000 033115			Olanzapine 20mg orodispersible tablets
7681941000 033111			Olanzapine 5mg orodispersible tablets
7687841000 033114			Sondate XL 50mg tablets (Teva UK Ltd)
7687941000 033118			Sondate XL 200mg tablets (Teva UK Ltd)
7688041000 033115			Sondate XL 300mg tablets (Teva UK Ltd)
7688141000 033116			Sondate XL 400mg tablets (Teva UK Ltd)
7696410000 33110			Integrin Tablets 40 mg
8110410000 33116			Largactil 50mg/2ml solution for injection ampoules (Sanofi)
8112941000 033114			Tenprolide XL 50mg tablets (Actavis UK Ltd)
8113041000 033116			Tenprolide XL 200mg tablets (Actavis UK Ltd)
8113141000 033117			Tenprolide XL 300mg tablets (Actavis UK Ltd)
8133410000 33115			Largactil Suppositories 100 mg
8134410000 33114			Largactil Forte 100mg/5ml oral suspension (Sanofi)
8137410000 33119			Largactil 25mg/5ml syrup (Sanofi)
8157410000 33115			Largactil 10mg tablets (Sanofi)
8158410000 33113			Largactil 100mg tablets (Sanofi)
8159410000 33117			Largactil 25mg tablets (Sanofi)
8160410000 33110			Largactil 50mg tablets (Sanofi)
8246241000 033114			Sondate XL 150mg tablets (Teva UK Ltd)
8298341000 033111			Seotiapim XL 200mg tablets (Sandoz Ltd)
8298441000 033117			Seotiapim XL 300mg tablets (Sandoz Ltd)
8298541000 033116			Seotiapim XL 400mg tablets (Sandoz Ltd)
8349141000 033117			Tenprolide XL 400mg tablets (Actavis UK Ltd)
8407410000 33115			Loxapine 10mg capsules
8408410000 33113			Loxapine 25mg capsules
8409410000 33117			Loxapine 50mg capsules

842041000033117			Loxapac 10mg capsules (Wyeth Pharmaceuticals)
842141000033118			Loxapac 25mg capsules (Wyeth Pharmaceuticals)
842241000033113			Loxapac 50mg capsules (Wyeth Pharmaceuticals)
8540041000033111			Psytixol 50mg/0.5ml solution for injection ampoules (Mylan)
885941000033119			Methotrimeprazine Hydrochloride Injection 25mg/ml, 1 ml ampoule
8882441000033111			Chlorpromazine Hydrochloride Suppositories 25 mg
8885941000033111			Chlorpromazine 25mg suppositories
8886041000033118			Chlorpromazine 10mg capsules
892141000033114			Melleril 100mg/5ml oral suspension (Novartis Pharmaceuticals UK Ltd)
892241000033119			Melleril 25mg/5ml oral suspension (Novartis Pharmaceuticals UK Ltd)
893741000033115			Melleril 25mg/5ml syrup (Novartis Pharmaceuticals UK Ltd)
894141000033116			Melleril Syrup 3mg/10 ml
895841000033114			Melleril 10mg tablets (Novartis Pharmaceuticals UK Ltd)
895941000033118			Melleril 100mg tablets (Novartis Pharmaceuticals UK Ltd)
8960241000033117			Aripiprazole 400mg powder and solvent for suspension for injection vials
896041000033111			Melleril 25mg tablets (Novartis Pharmaceuticals UK Ltd)
8960441000033116			Abilify Maintena 400mg powder and solvent for prolonged-release suspension for injection vials (Otsuka Pharmaceuticals (U.K.) Ltd)
896141000033110			Melleril 50mg tablets (Novartis Pharmaceuticals UK Ltd)
902641000033111			Methotrimeprazine Maleate Tablets 25 mg
924241000033110			Modecate 25mg/1ml solution for injection pre-filled syringes (Sanofi-Synthelabo Ltd)
924341000033117			Modecate 50mg/2ml solution for injection pre-filled syringes (Sanofi-Synthelabo Ltd)
925341000033116			Modecate 50mg/2ml solution for injection ampoules (Sanofi)
925841000033113			Modecate 12.5mg/0.5ml solution for injection ampoules (Sanofi)
925941000033117			Modecate Concentrate 100mg/1ml solution for injection ampoules (Sanofi)
926041000033110			Moditen Enanthate Injection 25 mg/ml
927741000033110			Modecate 250mg/10ml solution for injection vials (Sanofi-Synthelabo Ltd)

9278410000 33117			Modecate Concentrate 50mg/0.5ml solution for injection ampoules (Sanofi)
9348410000 33111			Modecate 25mg/1ml solution for injection ampoules (Sanofi)
9350410000 33115			Moditen 1mg tablets (Sanofi)
9351410000 33116			Moditen 2.5mg tablets (Sanofi)
9352410000 33111			Moditen 5mg tablets (Sanofi-Synthelabo Ltd)
9388410000 33118			Motipress tablets (Sanofi-Synthelabo Ltd)
9389410000 33114			Motival 10mg/500microgram tablets (Sanofi)
9604441000 033112			Ebesque XL 50mg tablets (Ethypharm UK Ltd)
9605541000 033112			Ebesque XL 200mg tablets (Ethypharm UK Ltd)
9605941000 033118			Ebesque XL 300mg tablets (Ethypharm UK Ltd)
9606341000 033113			Ebesque XL 400mg tablets (Ethypharm UK Ltd)
9650410000 33112			Neulactil Forte syrup (Sanofi)
9655410000 33119			Neulactil 10mg tablets (Sanofi)
9656410000 33118			Neulactil 2.5mg tablets (Sanofi)
9657410000 33110			Neulactil Tablets 25 mg
9808941000 033118			Lurasidone 18.5mg tablets
9809041000 033110			Lurasidone 37mg tablets
9809141000 033114			Lurasidone 74mg tablets
9809241000 033119			Latuda 18.5mg tablets (Sunovion Pharmaceuticals Europe Ltd)
9809341000 033112			Latuda 37mg tablets (Sunovion Pharmaceuticals Europe Ltd)
9809441000 033118			Latuda 74mg tablets (Sunovion Pharmaceuticals Europe Ltd)
9816410000 33117			Nozinan 25mg/1ml solution for injection ampoules (Sanofi)

High Anti-cholinergic Burden Drugs

**ACB score 3 drugs Aurum codelist**			
ProdCode	ProdName	Term	Substance
89341000033116		Atropine Sulfate Injection 800 micrograms/ml	
593241000033110		Flavoxate Hydrochloride Tablets 100 mg	

912941000033119		Min-I-Jet Atropine Injection 100 micrograms/ml	
983141000033114		Nortriptyline Liquid 10mg/5 ml	
1007241000033118		Opulets Atropine Sulphate Eye drops 1 %	
1013341000033119		Orphenadrine Citrate Injection 30 mg/ml	
1134841000033113		Promethazine Theoclate Tablets 25 mg	
1138741000033113		Propantheline Tablets 15 mg	
1670041000033113		Promethazine Hydrochloride Injection 50 mg/2 ml ampoule	
1832841000033113		Quetiapine Fumarate Tablets (starter pack) 6x25 mg, 2x100 mg, 2x150 mg	
2190041000033118		Diphenhydramine Hydrochloride Caplets 25 mg	
929241000033111		Morphine And Atropine Injection 1 ml ampoule	
19241000033112		Adrenaline And Atropine Spray	
43441000033118		Amitriptyline Sr Capsules 75 mg	
43641000033116		Amitriptyline Hydrochloride Capsules 50 mg	
1669941000033112		Promethazine Hydrochloride Injection 25 mg/ml	
1065341000033119		Perphenazine Tablets 8 mg	
1017441000033119		Orphenadrine Citrate Tablets 100 mg	
1052041000033118		Perphenazine Injection 5 mg/ml	
430141000033112		Desipramine Tablets 25 mg	
163141000033114		Brompheniramine Maleate Tablets 4 mg	
51941000033114		Amitriptyline Hydrochloride Mixture Sugar Free 10 mg/5 ml	
61141000033117		Amoxapine Tablets 150 mg	
465041000033110		Dimenhydrinate Tablets 50 mg	
4133841000033118		Quetiapine Oral Suspension Sugar Free 12.5 mg/5 ml	
1432741000033112		Thioridazine Syrup 12.5mg/5 ml	
1013041000033116		Orphenadrine Hydrochloride Injection 20 mg/ml	
1146241000033119		Pyridoxine Hydrochloride Tablets 100 mg	
1150341000033113		Quetiapine Fumarate Tablets (starter pack) 6 x 25 mg, 2 x 100 mg	
50041000033116		Amitriptyline Hydrochloride Injection 10mg/ml	
162641000033112		Brompheniramine Maleate Tablets 12 mg	
460541000033113		Diphenhydramine Hydrochloride Syrup 10 mg/5 ml	
43541000033117		Amitriptyline Hydrochloride Capsules 25 mg	

43741000033113		Amitriptyline Hydrochloride Capsules 75 mg	
60841000033118		Amoxapine Tablets 25 mg	
92241000033115	Atropine 600microgram tablets	Atropine 600microgram tablets	Atropine sulfate
1139641000033113	Propantheline bromide 15mg tablets	Propantheline bromide 15mg tablets	Propantheline bromide
3086141000033116	Chlorphenamine 4mg tablets	Chlorphenamine 4mg tablets	Chlorphenamine maleate
285641000033119	Clemastine 1mg tablets	Clemastine 1mg tablets	Clemastine hydrogen fumarate
272841000033117	Clemastine 500micrograms/5ml oral solution sugar free	Clemastine 500micrograms/5ml oral solution sugar free	Clemastine hydrogen fumarate
462741000033116	Diphenhydramine 25mg tablets	Diphenhydramine 25mg tablets	Diphenhydramine hydrochloride
2189941000033111	Diphenhydramine 50mg tablets	Diphenhydramine 50mg tablets	Diphenhydramine hydrochloride
1134541000033111	Promethazine hydrochloride 10mg tablets	Promethazine hydrochloride 10mg tablets	Promethazine hydrochloride
1134641000033112	Promethazine hydrochloride 25mg tablets	Promethazine hydrochloride 25mg tablets	Promethazine hydrochloride
745241000033119	Hydroxyzine 10mg tablets	Hydroxyzine 10mg tablets	Hydroxyzine hydrochloride
745341000033112	Hydroxyzine 25mg tablets	Hydroxyzine 25mg tablets	Hydroxyzine hydrochloride
1065141000033117	Perphenazine 2mg tablets	Perphenazine 2mg tablets	Perphenazine
1065241000033112	Perphenazine 4mg tablets	Perphenazine 4mg tablets	Perphenazine
1433641000033111	Thioridazine 10mg tablets	Thioridazine 10mg tablets	Thioridazine hydrochloride
1433841000033112	Thioridazine 25mg tablets	Thioridazine 25mg tablets	Thioridazine hydrochloride
1433941000033116	Thioridazine 50mg tablets	Thioridazine 50mg tablets	Thioridazine hydrochloride
1433741000033119	Thioridazine 100mg tablets	Thioridazine 100mg tablets	Thioridazine hydrochloride
1432441000033117	Thioridazine 25mg/5ml oral suspension	Thioridazine 25mg/5ml oral suspension	Thioridazine
1432341000033111	Thioridazine 100mg/5ml oral suspension	Thioridazine 100mg/5ml oral suspension	Thioridazine
1432541000033116	Thioridazine 25mg/5ml oral solution	Thioridazine 25mg/5ml oral solution	Thioridazine hydrochloride
287641000033113	Clozapine 25mg tablets	Clozapine 25mg tablets	Clozapine

287541000033112	Clozapine 100mg tablets	Clozapine 100mg tablets	Clozapine
1003741000033116	Olanzapine 5mg tablets	Olanzapine 5mg tablets	Olanzapine
1003841000033114	Olanzapine 7.5mg tablets	Olanzapine 7.5mg tablets	Olanzapine
1003641000033113	Olanzapine 10mg tablets	Olanzapine 10mg tablets	Olanzapine
1661141000033114	Olanzapine 2.5mg tablets	Olanzapine 2.5mg tablets	Olanzapine
2077741000033115	Olanzapine 5mg oral lyophilisates sugar free	Olanzapine 5mg oral lyophilisates sugar free	Olanzapine
2077841000033113	Olanzapine 10mg oral lyophilisates sugar free	Olanzapine 10mg oral lyophilisates sugar free	Olanzapine
1150241000033115	Quetiapine 25mg tablets	Quetiapine 25mg tablets	Quetiapine fumarate
1150041000033111	Quetiapine 100mg tablets	Quetiapine 100mg tablets	Quetiapine fumarate
1150141000033110	Quetiapine 200mg tablets	Quetiapine 200mg tablets	Quetiapine fumarate
1844641000033118	Quetiapine 150mg tablets	Quetiapine 150mg tablets	Quetiapine fumarate
60441000033116	Amitriptyline 10mg tablets	Amitriptyline 10mg tablets	Amitriptyline hydrochloride
60641000033119	Amitriptyline 25mg tablets	Amitriptyline 25mg tablets	Amitriptyline hydrochloride
60541000033115	Amitriptyline 50mg tablets	Amitriptyline 50mg tablets	Amitriptyline hydrochloride
989141000033111	Nortriptyline 10mg tablets	Nortriptyline 10mg tablets	Nortriptyline hydrochloride
989241000033116	Nortriptyline 25mg tablets	Nortriptyline 25mg tablets	Nortriptyline hydrochloride
1454741000033115	Trimipramine 50mg capsules	Trimipramine 50mg capsules	Trimipramine maleate
1471341000033116	Trimipramine 10mg tablets	Trimipramine 10mg tablets	Trimipramine maleate
1471441000033110	Trimipramine 25mg tablets	Trimipramine 25mg tablets	Trimipramine maleate
60941000033114	Amoxapine 50mg tablets	Amoxapine 50mg tablets	Amoxapine
61041000033116	Amoxapine 100mg tablets	Amoxapine 100mg tablets	Amoxapine
3179941000033111	Nortriptyline 10mg / Fluphenazine 500microgram tablets	Nortriptyline 10mg / Fluphenazine 500microgram tablets	Fluphenazine hydrochloride/ Nortriptyline hydrochloride
3180441000033118	Amitriptyline 25mg / Perphenazine 2mg tablets	Amitriptyline 25mg / Perphenazine 2mg tablets	Amitriptyline hydrochloride/ Perphenazine
3180541000033117	Amitriptyline 10mg / Perphenazine 2mg tablets	Amitriptyline 10mg / Perphenazine 2mg tablets	Amitriptyline hydrochloride/ Perphenazine

1042141000033117	Paroxetine 20mg tablets	Paroxetine 20mg tablets	Paroxetine hydrochloride
1042241000033112	Paroxetine 30mg tablets	Paroxetine 30mg tablets	Paroxetine hydrochloride
3109641000033112	Promethazine teoclate 25mg tablets	Promethazine teoclate 25mg tablets	Promethazine teoclate
13395041000033113	Paracetamol 500mg / Diphenhydramine 25mg tablets	Paracetamol 500mg / Diphenhydramine 25mg tablets	Diphenhydramine hydrochloride/ Paracetamol
3063241000033115	Trihexyphenidyl 2mg tablets	Trihexyphenidyl 2mg tablets	Trihexyphenidyl hydrochloride
3063341000033113	Trihexyphenidyl 5mg tablets	Trihexyphenidyl 5mg tablets	Trihexyphenidyl hydrochloride
1011941000033119	Orphenadrine 25mg/5ml oral solution sugar free	Orphenadrine 25mg/5ml oral solution sugar free	Orphenadrine hydrochloride
1016841000033119	Orphenadrine 50mg tablets	Orphenadrine 50mg tablets	Orphenadrine hydrochloride
594741000033118	Flavoxate 200mg tablets	Flavoxate 200mg tablets	Flavoxate hydrochloride
1701641000033110	Tolterodine 1mg tablets	Tolterodine 1mg tablets	Tolterodine tartrate
1701741000033118	Tolterodine 2mg tablets	Tolterodine 2mg tablets	Tolterodine tartrate
2191141000033114	Trospium chloride 20mg tablets	Trospium chloride 20mg tablets	Trospium chloride
1670641000033119	Propiverine 15mg tablets	Propiverine 15mg tablets	Propiverine hydrochloride
1146141000033114	Pyridoxine 10mg tablets	Pyridoxine 10mg tablets	Pyridoxine hydrochloride
1146341000033112	Pyridoxine 20mg tablets	Pyridoxine 20mg tablets	Pyridoxine hydrochloride
1146441000033118	Pyridoxine 50mg tablets	Pyridoxine 50mg tablets	Pyridoxine hydrochloride
88741000033112	Atropine 1% eye drops	Atropine 1% eye drops	Atropine sulfate
88841000033119	Atropine 1% eye ointment	Atropine 1% eye ointment	Atropine sulfate
1818741000033114	Atropine 0.5% eye drops	Atropine 0.5% eye drops	Atropine sulfate
2922241000033119	Promethazine hydrochloride 20mg tablets	Promethazine hydrochloride 20mg tablets	Promethazine hydrochloride
3996241000033117	Olanzapine 20mg tablets	Olanzapine 20mg tablets	Olanzapine
2759641000033117	Olanzapine 15mg tablets	Olanzapine 15mg tablets	Olanzapine
377241000033112	Co-phenotrope 2.5mg/0.025mg tablets	Co-phenotrope 2.5mg/0.025mg tablets	Atropine sulfate/ Diphenoxylate hydrochloride
3866541000033110	Olanzapine 20mg oral lyophilisates sugar free	Olanzapine 20mg oral lyophilisates sugar free	Olanzapine

3178241000033110	Solifenacin 10mg tablets	Solifenacin 10mg tablets	Solifenacin succinate
3178141000033115	Solifenacin 5mg tablets	Solifenacin 5mg tablets	Solifenacin succinate
5078041000033117	Clozapine 50mg tablets	Clozapine 50mg tablets	Clozapine
4079041000033115	Paroxetine 10mg tablets	Paroxetine 10mg tablets	Paroxetine hydrochloride
11755441000033119	Paroxetine 40mg tablets	Paroxetine 40mg tablets	Paroxetine hydrochloride
4591941000033112	Quetiapine 300mg modified-release tablets	Quetiapine 300mg modified-release tablets	Quetiapine fumarate
4591841000033116	Quetiapine 200mg modified-release tablets	Quetiapine 200mg modified-release tablets	Quetiapine fumarate
4592041000033118	Quetiapine 400mg modified-release tablets	Quetiapine 400mg modified-release tablets	Quetiapine fumarate
5270941000033111	Trospium chloride 60mg modified-release capsules	Trospium chloride 60mg modified-release capsules	Trospium chloride
2975841000033113	Quetiapine 300mg tablets	Quetiapine 300mg tablets	Quetiapine fumarate
1844741000033110	Seroquel 150mg tablets	Seroquel 150mg tablets (AstraZeneca UK Ltd)	Quetiapine fumarate
148241000033115	Biorphen 25mg/5ml oral solution	Biorphen 25mg/5ml oral solution (Rosemont Pharmaceuticals Ltd)	Orphenadrine hydrochloride
2643641000033111	Detrusitol XL 4mg capsules	Detrusitol XL 4mg capsules (Upjohn UK Ltd)	Tolterodine tartrate
442941000033111	Dimotane 2mg/5ml elixir	Dimotane 2mg/5ml elixir (Mercury Pharma Group Ltd)	Brompheniramine maleate
1686141000033119	Seroxat 20mg/10ml liquid	Seroxat 20mg/10ml liquid (GlaxoSmithKline UK Ltd)	Paroxetine hydrochloride
1090541000033115	Piriton 2mg/5ml syrup	Piriton 2mg/5ml syrup (GlaxoSmithKline Consumer Healthcare)	Chlorphenamine maleate
599041000033117	Forceval capsules	Forceval capsules (Forum Health Products Ltd)	Ascorbic acid/ Biotin/ Calcium/ Chromium/ Copper/ Cyanocobalamin/ Ergocalciferol/ Folic acid/ Iodine/ Iron/ Magnesium/ Manganese/ Molybdenum/ Nicotinamide/ Pantothenic acid/ Phosphorus/ Potassium/ Pyridoxine/ Riboflavin/ Selenium/ Thiamine/ Tocopheryl acetate/ Vitamin A/ Zinc
450941000033113	Dimotane Plus oral solution	Dimotane Plus oral solution (Mercury Pharma Group Ltd)	Brompheniramine maleate/ Pseudoephedrine hydrochloride

892241000033119	Melleril 25mg/5ml oral suspension	Melleril 25mg/5ml oral suspension (Novartis Pharmaceuticals UK Ltd)	Thioridazine
2929641000033110	Phenergan 25mg/1ml solution for injection ampoules	Phenergan 25mg/1ml solution for injection ampoules (Sanofi)	Promethazine hydrochloride
2077941000033117	Zyprexa 5mg Velotabs	Zyprexa 5mg Velotabs (Eli Lilly and Company Ltd)	Olanzapine
1389441000033112	Surmontil 50mg capsules	Surmontil 50mg capsules (Sanofi)	Trimipramine maleate
456341000033111	Dimotane Plus Paediatric elixir	Dimotane Plus Paediatric elixir (Mercury Pharma Group Ltd)	Brompheniramine maleate/ Pseudoephedrine hydrochloride
469941000033114	Disipal 50mg tablets	Disipal 50mg tablets (Astellas Pharma Ltd)	Orphenadrine hydrochloride
938941000033114	Motival 10mg/500microgram tablets	Motival 10mg/500microgram tablets (Sanofi)	Fluphenazine hydrochloride/ Nortriptyline hydrochloride
1071241000033117	Phenergan 5mg/5ml elixir	Phenergan 5mg/5ml elixir (Sanofi)	Promethazine hydrochloride
896041000033111	Melleril 25mg tablets	Melleril 25mg tablets (Novartis Pharmaceuticals UK Ltd)	Thioridazine hydrochloride
1080741000033112	Phenergan Nightime 25mg tablets	Phenergan Nightime 25mg tablets (Sanofi)	Promethazine hydrochloride
1601641000033117	Detrusitol 2mg tablets	Detrusitol 2mg tablets (Upjohn UK Ltd)	Tolterodine tartrate
2191241000033119	Regurin 20mg tablets	Regurin 20mg tablets (Mylan)	Trospium chloride
1713941000033118	Zyprexa 2.5mg tablets	Zyprexa 2.5mg tablets (Eli Lilly and Company Ltd)	Olanzapine
1277641000033116	Seroquel 100mg tablets	Seroquel 100mg tablets (Luye Pharma Ltd)	Quetiapine fumarate
1079541000033116	Phenergan 25mg tablets	Phenergan 25mg tablets (Sanofi)	Promethazine hydrochloride
1495041000033113	Urispas 200 tablets	Urispas 200 tablets (Recordati Pharmaceuticals Ltd)	Flavoxate hydrochloride
1091541000033114	Piriton 4mg tablets	Piriton 4mg tablets (GlaxoSmithKline Consumer Healthcare)	Chlorphenamine maleate
2759941000033112	Zyprexa 15mg Velotabs	Zyprexa 15mg Velotabs (Eli Lilly and Company Ltd)	Olanzapine
90941000033111	Atarax 25mg tablets	Atarax 25mg tablets (Alliance Pharmaceuticals Ltd)	Hydroxyzine hydrochloride
1565241000033114	Zyprexa 5mg tablets	Zyprexa 5mg tablets (Eli Lilly and Company Ltd)	Olanzapine
1277741000033113	Seroquel 200mg tablets	Seroquel 200mg tablets (Luye Pharma Ltd)	Quetiapine fumarate
1278341000033111	Seroxat 20mg tablets	Seroxat 20mg tablets (GlaxoSmithKline UK Ltd)	Paroxetine hydrochloride
1473441000033111	Triptafen-M tablets	Triptafen-M tablets (Mercury Pharma Group Ltd)	Amitriptyline hydrochloride/ Perphenazine
1565141000033119	Zyprexa 10mg tablets	Zyprexa 10mg tablets (Eli Lilly and Company Ltd)	Olanzapine
938841000033118	Motipress tablets	Motipress tablets (Sanofi-Synthelabo Ltd)	Fluphenazine hydrochloride/ Nortriptyline hydrochloride

1396841000033119	Surmontil 25mg tablets	Surmontil 25mg tablets (Sanofi)	Trimipramine maleate
90841000033115	Atarax 10mg tablets	Atarax 10mg tablets (Alliance Pharmaceuticals Ltd)	Hydroxyzine hydrochloride
2759841000033116	Zyprexa 15mg tablets	Zyprexa 15mg tablets (Eli Lilly and Company Ltd)	Olanzapine
1473341000033117	Triptafen tablets	Triptafen tablets (Advanz Pharma)	Amitriptyline hydrochloride/ Perphenazine
1079441000033117	Phenergan 10mg tablets	Phenergan 10mg tablets (Sanofi)	Promethazine hydrochloride
892141000033114	Melleril 100mg/5ml oral suspension	Melleril 100mg/5ml oral suspension (Novartis Pharmaceuticals UK Ltd)	Thioridazine
624141000033116	Galpseud Plus linctus	Galpseud Plus linctus (Thornton & Ross Ltd)	Chlorphenamine maleate/ Pseudoephedrine hydrochloride
1277841000033115	Seroquel 25mg tablets	Seroquel 25mg tablets (Luye Pharma Ltd)	Quetiapine fumarate
38841000033112	Allegron 25mg tablets	Allegron 25mg tablets (King Pharmaceuticals Ltd)	Nortriptyline hydrochloride
1601741000033114	Detrunorm 15mg tablets	Detrunorm 15mg tablets (Advanz Pharma)	Propiverine hydrochloride
895841000033114	Melleril 10mg tablets	Melleril 10mg tablets (Novartis Pharmaceuticals UK Ltd)	Thioridazine hydrochloride
1278441000033117	Seroxat 30mg tablets	Seroxat 30mg tablets (GlaxoSmithKline UK Ltd)	Paroxetine hydrochloride
1565341000033116	Zyprexa 7.5mg tablets	Zyprexa 7.5mg tablets (Eli Lilly and Company Ltd)	Olanzapine
1601541000033118	Detrusitol 1mg tablets	Detrusitol 1mg tablets (Upjohn UK Ltd)	Tolterodine tartrate
893741000033115	Melleril 25mg/5ml syrup	Melleril 25mg/5ml syrup (Novartis Pharmaceuticals UK Ltd)	Thioridazine hydrochloride
2130441000033116	Nytol One-A-Night 50mg tablets	Nytol One-A-Night 50mg tablets (Omega Pharma Ltd)	Diphenhydramine hydrochloride
2956341000033114	Agitane 2mg tablets	Agitane 2mg tablets (Dr Reddy's Laboratories (UK) Ltd)	Trihexyphenidyl hydrochloride
2956441000033115	Agitane 5mg tablets	Agitane 5mg tablets (Dr Reddy's Laboratories (UK) Ltd)	Trihexyphenidyl hydrochloride
1481741000033112	Ucerax 25mg tablets	Ucerax 25mg tablets (UCB Pharma Ltd)	Hydroxyzine hydrochloride
1137841000033116	Pro-Banthine 15mg tablets	Pro-Banthine 15mg tablets (Kyowa Kirin Ltd)	Propantheline bromide
896141000033110	Melleril 50mg tablets	Melleril 50mg tablets (Novartis Pharmaceuticals UK Ltd)	Thioridazine hydrochloride
895941000033118	Melleril 100mg tablets	Melleril 100mg tablets (Novartis Pharmaceuticals UK Ltd)	Thioridazine hydrochloride
38741000033119	Allegron 10mg tablets	Allegron 10mg tablets (King Pharmaceuticals Ltd)	Nortriptyline hydrochloride
2078041000033119	Zyprexa 10mg Velotabs	Zyprexa 10mg Velotabs (Eli Lilly and Company Ltd)	Olanzapine
1396741000033112	Surmontil 10mg tablets	Surmontil 10mg tablets (Sanofi)	Trimipramine maleate
1481641000033115	Ucerax 10mg/5ml syrup	Ucerax 10mg/5ml syrup (UCB Pharma Ltd)	Hydroxyzine hydrochloride

96341000033114	Avomine 25mg tablets	Avomine 25mg tablets (Manx Healthcare Ltd)	Promethazine teoclate
402541000033113	Dalivit oral drops	Dalivit oral drops (Boston Healthcare Ltd)	Ascorbic acid/ Ergocalciferol/ Nicotinamide/ Pyridoxine/ Riboflavin/ Thiamine/ Vitamin A
804241000033113	Ketovite tablets	Ketovite tablets (Essential Pharmaceuticals Ltd)	Acetomenaphthone/ Alpha tocopheryl acetate/ Ascorbic acid/ Biotin/ Calcium pantothenate/ Folic acid/ Inositol/ Nicotinamide/ Pyridoxine hydrochloride/ Riboflavin/ Thiamine hydrochloride
2616441000033113	Nightcalm 25mg tablets	Nightcalm 25mg tablets (Galpharm International Ltd)	Diphenhydramine hydrochloride
997041000033116	Nytol 25mg tablets	Nytol 25mg tablets (Omega Pharma Ltd)	Diphenhydramine hydrochloride
2970241000033117	Dreemon 25mg tablets	Dreemon 25mg tablets (E M Pharma)	Diphenhydramine hydrochloride
1441000033111	Abidec Multivitamin drops	Abidec Multivitamin drops (Omega Pharma Ltd)	Ascorbic acid/ Ergocalciferol/ Nicotinamide/ Pyridoxine hydrochloride/ Riboflavin/ Thiamine hydrochloride/ Vitamin A palmitate
2759741000033114	Olanzapine 15mg oral lyophilisates sugar free	Olanzapine 15mg oral lyophilisates sugar free	Olanzapine
3978241000033112	Pseudoephedrine 30mg/5ml / Chlorphenamine 2mg/5ml oral solution sugar free	Pseudoephedrine 30mg/5ml / Chlorphenamine 2mg/5ml oral solution sugar free	Chlorphenamine maleate/ Pseudoephedrine hydrochloride
1408941000033117	Tavegil 1mg tablets	Tavegil 1mg tablets (GlaxoSmithKline Consumer Healthcare)	Clemastine hydrogen fumarate
1404541000033117	Tavegil 500micrograms/5ml elixir	Tavegil 500micrograms/5ml elixir (Novartis Consumer Health UK Ltd)	Clemastine hydrogen fumarate
570941000033117	Fentazin 2mg tablets	Fentazin 2mg tablets (Advanz Pharma)	Perphenazine
571041000033110	Fentazin 4mg tablets	Fentazin 4mg tablets (Advanz Pharma)	Perphenazine
663141000033113	Haymine tablets	Haymine tablets (Chemidex Pharma Ltd)	Chlorphenamine maleate/ Ephedrine hydrochloride
12910041000033116	Ephedrine hydrochloride 15mg / Chlorphenamine 10mg tablets	Ephedrine hydrochloride 15mg / Chlorphenamine 10mg tablets	Chlorphenamine maleate/ Ephedrine hydrochloride
161041000033111	Broflex 5mg/5ml syrup	Broflex 5mg/5ml syrup (Rosemont Pharmaceuticals Ltd)	Trihexyphenidyl hydrochloride
87941000033111	Asendis 50mg tablets	Asendis 50mg tablets (Mercury Pharma Group Ltd)	Amoxapine
284941000033113	Clozaril 25mg tablets	Clozaril 25mg tablets (Mylan)	Clozapine

285041000033113	Clozaril 100mg tablets	Clozaril 100mg tablets (Mylan)	Clozapine
777741000033117	Isopto Atropine 1% eye drops	Isopto Atropine 1% eye drops (Alcon Eye Care Ltd)	Atropine sulfate
87641000033116	Asendis 100mg tablets	Asendis 100mg tablets (Mercury Pharma Group Ltd)	Amoxapine
909941000033112	Minims atropine sulfate 1% eye drops 0.5ml unit dose	Minims atropine sulfate 1% eye drops 0.5ml unit dose (Bausch & Lomb UK Ltd)	Atropine sulfate
4434641000033116	Tixylix Night Cough oral solution	Tixylix Night Cough oral solution (Novartis Consumer Health UK Ltd)	Pholcodine/ Promethazine hydrochloride
1034841000033117	Pamergan P100 solution for injection 2ml ampoules	Pamergan P100 solution for injection 2ml ampoules (Martindale Pharmaceuticals Ltd)	Pethidine hydrochloride/ Promethazine hydrochloride
2971641000033110	Benadryl Skin Allergy Relief cream	Benadryl Skin Allergy Relief cream (McNeil Products Ltd)	Diphenhydramine hydrochloride/ Racemic camphor/ Zinc oxide
4434541000033117	Pholcodine 1.5mg/5ml / Promethazine 1.5mg/5ml oral solution sugar free	Pholcodine 1.5mg/5ml / Promethazine 1.5mg/5ml oral solution sugar free	Pholcodine/ Promethazine hydrochloride
1514541000033113	Vivioptal capsules	Vivioptal capsules (Bausch & Lomb UK Ltd)	Adenosine/ Alpha tocopheryl acetate/ Biotin/ Calcium ascorbate/ Calcium hydrogen phosphate anhydrous/ Choline hydrogen tartrate/ Cobaltous sulfate monohydrate/ Colecalciferol/ Copper sulfate monohydrate/ Cyanocobalamin/ Dexpanthenol/ Ethyl linoleate/ Ferrous sulfate dried/ Folic acid/ Inositol/ Lecithin/ L-Lysine monohydrochloride/ Magnesium glycerophosphate/ Manganese sulfate monohydrate/ Nicotinamide/ Orotic acid anhydrous/ Potassium sulfate/ Pyridoxine hydrochloride/ Riboflavin/ Rutoside/ Sodium molybdate anhydrous/ Thiamine nitrate/ Vitamin A palmitate/ Zinc oxide
9293641000033112	Berocca effervescent tablets orange	Berocca effervescent tablets orange (Bayer Plc)	Ascorbic acid/ Biotin/ Calcium/ Cyanocobalamin/ Folic acid/ Magnesium/ Nicotinic acid/ Pantothenic acid/ Pyridoxine/ Riboflavin/ Thiamine/ Zinc

1367741000033112	Sominex 20mg tablets	Sominex 20mg tablets (Teva UK Ltd)	Promethazine hydrochloride
9304041000033113	Valupak Vitamin B6 10mg tablets	Valupak Vitamin B6 10mg tablets (BR Pharmaceuticals Ltd)	Pyridoxine hydrochloride
2971541000033114	Benadryl Skin Allergy Relief lotion	Benadryl Skin Allergy Relief lotion (Pfizer Consumer Healthcare Ltd)	Diphenhydramine hydrochloride/ Racemic camphor/ Zinc oxide
2613141000033113	Covonia night time formula oral solution	Covonia night time formula oral solution (Thornton & Ross Ltd)	Dextromethorphan hydrobromide/ Diphenhydramine hydrochloride
5576941000033119	Panadol NightPain tablets	Panadol NightPain tablets (GlaxoSmithKline Consumer Healthcare)	Diphenhydramine hydrochloride/ Paracetamol
5263741000033114	Diphenhydramine 10mg/5ml / Dextromethorphan 6.65mg/5ml oral solution sugar free	Diphenhydramine 10mg/5ml / Dextromethorphan 6.65mg/5ml oral solution sugar free	Dextromethorphan hydrobromide/ Diphenhydramine hydrochloride
819841000033110	Lentizol 50mg modified-release capsules	Lentizol 50mg modified-release capsules (Pfizer Ltd)	Amitriptyline hydrochloride
819741000033117	Lentizol 25mg modified-release capsules	Lentizol 25mg modified-release capsules (Pfizer Ltd)	Amitriptyline hydrochloride
4060841000033113	Atropine 500micrograms/5ml solution for injection Minijet pre-filled syringes	Atropine 500micrograms/5ml solution for injection Minijet pre-filled syringes (UCB Pharma Ltd)	Atropine sulfate
4060941000033117	Atropine 1mg/10ml solution for injection Minijet pre-filled syringes	Atropine 1mg/10ml solution for injection Minijet pre-filled syringes (UCB Pharma Ltd)	Atropine sulfate
4060741000033115	Atropine 3mg/30ml solution for injection Minijet pre-filled syringes	Atropine 3mg/30ml solution for injection Minijet pre-filled syringes (UCB Pharma Ltd)	Atropine sulfate
1140541000033115	Propain caplets	Propain caplets (Ceuta Healthcare Ltd)	Caffeine/ Codeine phosphate/ Diphenhydramine hydrochloride/ Paracetamol
670341000033110	Histalix Syrup	Histalix Syrup (Wallace Manufacturing Chemists Ltd)	Ammonium chloride/ Diphenhydramine hydrochloride/ Menthol
296241000033113	Concavit capsules	Concavit capsules (Wallace Manufacturing Chemists Ltd)	Alpha tocopheryl acetate/ Ascorbic acid/ Calcium pantothenate/ Ergocalciferol/ Nicotinamide/ Pyridoxine hydrochloride/ Riboflavin/ Thiamine nitrate/ Vitamin A
325741000033112	Concavit drops	Concavit drops (Wallace Manufacturing Chemists Ltd)	Ascorbic acid/ Dexpanthenol/ Ergocalciferol/

			Nicotinamide/ Pyridoxine hydrochloride/ Riboflavin sodium phosphate/ Sodium ascorbate/ Thiamine hydrochloride/ Vitamin A
369041000033117	Concavit syrup	Concavit syrup (Wallace Manufacturing Chemists Ltd)	Ascorbic acid/ Dexpanthenol/ Ergocalciferol/ Nicotinamide/ Pyridoxine hydrochloride/ Riboflavin sodium phosphate/ Sodium ascorbate/ Thiamine hydrochloride/ Vitamin A
2975941000033117	Seroquel 300mg tablets	Seroquel 300mg tablets (Luye Pharma Ltd)	Quetiapine fumarate
228841000033115	Cernevit powder for solution for injection vials	Cernevit powder for solution for injection vials (Baxter Healthcare Ltd)	Alpha tocopherol/ Ascorbic acid/ Biotin/ Colecalciferol/ Cyanocobalamin/ Dexpanthenol/ Folic acid/ Nicotinamide/ Pyridoxine hydrochloride/ Retinol/ Riboflavin sodium phosphate/ Thiamine
3030941000033114	Zyprexa 10mg powder for solution for injection vials	Zyprexa 10mg powder for solution for injection vials (Eli Lilly and Company Ltd)	Olanzapine
3030841000033118	Olanzapine 10mg powder for solution for injection vials	Olanzapine 10mg powder for solution for injection vials	Olanzapine
3078141000033112	Dymotil 2.5mg/25microgram tablets	Dymotil 2.5mg/25microgram tablets (Mercury Pharma Group Ltd)	Atropine sulfate/ Diphenoxylate hydrochloride
1362641000033117	Solivito N powder for solution for infusion vials	Solivito N powder for solution for infusion vials (Fresenius Kabi Ltd)	Biotin/ Cyanocobalamin/ Folic acid/ Nicotinamide/ Pyridoxine hydrochloride/ Riboflavin sodium phosphate/ Sodium ascorbate/ Sodium pantothenate/ Thiamine nitrate
1403141000033111	Syndol caplets	Syndol caplets (Sanofi)	Caffeine/ Codeine phosphate/ Doxylamine succinate/ Paracetamol
2779741000033111	Allerief 2mg/5ml oral solution	Allerief 2mg/5ml oral solution (Crescent Pharma Ltd)	Chlorphenamine maleate
4158641000033114	Hayleve 4mg tablets	Hayleve 4mg tablets (Genesis Pharmaceuticals Ltd)	Chlorphenamine maleate
3158941000033116	Denzapine 25mg tablets	Denzapine 25mg tablets (Britannia Pharmaceuticals Ltd)	Clozapine
3159041000033113	Denzapine 100mg tablets	Denzapine 100mg tablets (Britannia Pharmaceuticals Ltd)	Clozapine
3178341000033117	Vesicare 5mg tablets	Vesicare 5mg tablets (Astellas Pharma Ltd)	Solifenacin succinate

3178441000033111	Vesicare 10mg tablets	Vesicare 10mg tablets (Astellas Pharma Ltd)	Solifenacin succinate
602141000033119	Forceval Junior capsules	Forceval Junior capsules (Alliance Pharmaceuticals Ltd)	Alpha tocopheryl acetate/ Ascorbic acid/ Betacarotene/ Chromium/ Copper/ Cyanocobalamin/ D-biotin/ Ergocalciferol/ Folic acid/ Iodine/ Iron/ Magnesium/ Manganese/ Molybdenum/ Nicotinamide/ Pantothenic acid/ Phytomenadione/ Pyridoxine/ Riboflavin/ Selenium/ Thiamine/ Zinc
9141000033119	Actonorm oral powder	Actonorm oral powder (Wallace Manufacturing Chemists Ltd)	Aluminium hydroxide gel dried/ Atropine sulfate/ Calcium carbonate/ Magnesium carbonate/ Magnesium trisilicate/ Peppermint oil/ Sodium bicarbonate
208741000033116	Calcimax syrup	Calcimax syrup (Wallace Manufacturing Chemists Ltd)	Ascorbic acid/ Calcium chloride dihydrate/ Calcium levulinate/ Calcium pantothenate/ Nicotinamide/ Pyridoxine hydrochloride/ Riboflavin/ Thiamine hydrochloride
5888641000033118	Amitriptyline 10mg/5ml oral suspension	Amitriptyline 10mg/5ml oral suspension	Amitriptyline hydrochloride
4309141000033112	Atropine 500micrograms/5ml oral solution	Atropine 500micrograms/5ml oral solution	Atropine sulfate
6124441000033110	Propantheline bromide 15mg/5ml oral suspension	Propantheline bromide 15mg/5ml oral suspension	Propantheline bromide
5974241000033113	Pyridoxine 100micrograms/5ml oral solution	Pyridoxine 100micrograms/5ml oral solution	Pyridoxine hydrochloride
5974341000033115	Pyridoxine 100micrograms/5ml oral suspension	Pyridoxine 100micrograms/5ml oral suspension	Pyridoxine hydrochloride
6065841000033113	Pyridoxine 10mg/5ml oral suspension	Pyridoxine 10mg/5ml oral suspension	Pyridoxine hydrochloride
3984841000033113	Pyridoxine 250mg/5ml oral solution	Pyridoxine 250mg/5ml oral solution	Pyridoxine hydrochloride
5974641000033111	Pyridoxine 25mg/5ml oral solution	Pyridoxine 25mg/5ml oral solution	Pyridoxine hydrochloride
5974741000033119	Pyridoxine 25mg/5ml oral suspension	Pyridoxine 25mg/5ml oral suspension	Pyridoxine hydrochloride
5974441000033114	Pyridoxine 50mg/5ml oral solution	Pyridoxine 50mg/5ml oral solution	Pyridoxine hydrochloride
5974541000033110	Pyridoxine 50mg/5ml oral suspension	Pyridoxine 50mg/5ml oral suspension	Pyridoxine hydrochloride

4429141000033110	Trihexyphenidyl 2mg/5ml oral solution	Trihexyphenidyl 2mg/5ml oral solution	Trihexyphenidyl hydrochloride
8868041000033115	Trihexyphenidyl 2mg/5ml oral suspension	Trihexyphenidyl 2mg/5ml oral suspension	Trihexyphenidyl hydrochloride
3226341000033115	Zaponex 25mg tablets	Zaponex 25mg tablets (Leyden Delta B.V.)	Clozapine
3226441000033114	Zaponex 100mg tablets	Zaponex 100mg tablets (Leyden Delta B.V.)	Clozapine
4020441000033117	Histergan 10mg/5ml syrup	Histergan 10mg/5ml syrup (Norma Chemicals Ltd)	Diphenhydramine hydrochloride
140841000033118	Benylin with Codeine oral solution	Benylin with Codeine oral solution (Pfizer Consumer Healthcare Ltd)	Codeine phosphate/ Diphenhydramine hydrochloride/ Levomenthol
1522341000033119	Vigranon-B syrup	Vigranon-B syrup (Wallace Manufacturing Chemists Ltd)	Dexpanthenol/ Nicotinamide/ Pyridoxine hydrochloride/ Riboflavin sodium phosphate/ Thiamine hydrochloride
3866641000033111	Zyprexa 20mg Velotabs	Zyprexa 20mg Velotabs (Eli Lilly and Company Ltd)	Olanzapine
3938941000033117	Detrunorm XL 30mg capsules	Detrunorm XL 30mg capsules (Advanz Pharma)	Propiverine hydrochloride
3938841000033113	Propiverine 30mg modified-release capsules	Propiverine 30mg modified-release capsules	Propiverine hydrochloride
3996341000033110	Zyprexa 20mg tablets	Zyprexa 20mg tablets (Eli Lilly and Company Ltd)	Olanzapine
3993341000033118	Emselex 7.5mg modified-release tablets	Emselex 7.5mg modified-release tablets (Aspire Pharma Ltd)	Darifenacin hydrobromide
3993441000033112	Emselex 15mg modified-release tablets	Emselex 15mg modified-release tablets (Aspire Pharma Ltd)	Darifenacin hydrobromide
4021041000033117	Arlevert tablets	Arlevert tablets (Hennig Arzneimittel GmbH & Co. KG)	Cinnarizine/ Dimenhydrinate
4020941000033110	Cinnarizine 20mg / Dimenhydrinate 40mg tablets	Cinnarizine 20mg / Dimenhydrinate 40mg tablets	Cinnarizine/ Dimenhydrinate
4079141000033116	Seroxat 10mg tablets	Seroxat 10mg tablets (GlaxoSmithKline UK Ltd)	Paroxetine hydrochloride
12880341000033112	Hydroxyzine 2.5mg/5ml oral solution	Hydroxyzine 2.5mg/5ml oral solution	Hydroxyzine hydrochloride
5889741000033118	Quetiapine 12.5mg/5ml oral solution	Quetiapine 12.5mg/5ml oral solution	Quetiapine fumarate
5889841000033111	Quetiapine 12.5mg/5ml oral suspension	Quetiapine 12.5mg/5ml oral suspension	Quetiapine fumarate
5974841000033112	Quetiapine 25mg/5ml oral solution	Quetiapine 25mg/5ml oral solution	Quetiapine fumarate

5974941000033116	Quetiapine 25mg/5ml oral suspension	Quetiapine 25mg/5ml oral suspension	Quetiapine fumarate
9776541000033118	Pyridoxine 100mg/5ml oral solution	Pyridoxine 100mg/5ml oral solution	Pyridoxine hydrochloride
11755341000033113	Pyridoxine 300mg/5ml oral solution	Pyridoxine 300mg/5ml oral solution	Pyridoxine hydrochloride
13117441000033119	Trihexyphenidyl 5mg/5ml oral suspension	Trihexyphenidyl 5mg/5ml oral suspension	Trihexyphenidyl hydrochloride
4593941000033111	ALLERcalm Allergy Relief 4mg tablets	ALLERcalm Allergy Relief 4mg tablets (Actavis UK Ltd)	Chlorphenamine maleate
4518741000033119	Toviaz 4mg modified-release tablets	Toviaz 4mg modified-release tablets (Pfizer Ltd)	Fesoterodine fumarate
4518841000033112	Toviaz 8mg modified-release tablets	Toviaz 8mg modified-release tablets (Pfizer Ltd)	Fesoterodine fumarate
5490141000033118	Amitriptyline 10mg/5ml oral solution	Amitriptyline 10mg/5ml oral solution	Amitriptyline hydrochloride
5997341000033118	Olanzapine 2.5mg/5ml oral solution	Olanzapine 2.5mg/5ml oral solution	Olanzapine
3892541000033114	Olanzapine 2.5mg/5ml oral suspension	Olanzapine 2.5mg/5ml oral suspension	Olanzapine
5891141000033117	Quetiapine 100mg/5ml oral solution	Quetiapine 100mg/5ml oral solution	Quetiapine fumarate
4422541000033110	Quetiapine 100mg/5ml oral suspension	Quetiapine 100mg/5ml oral suspension	Quetiapine fumarate
4592241000033114	Seroquel XL 200mg tablets	Seroquel XL 200mg tablets (Luye Pharma Ltd)	Quetiapine fumarate
4592441000033110	Seroquel XL 400mg tablets	Seroquel XL 400mg tablets (Luye Pharma Ltd)	Quetiapine fumarate
4592341000033116	Seroquel XL 300mg tablets	Seroquel XL 300mg tablets (Luye Pharma Ltd)	Quetiapine fumarate
4592141000033119	Seroquel XL 50mg tablets	Seroquel XL 50mg tablets (Luye Pharma Ltd)	Quetiapine fumarate
4591741000033114	Quetiapine 50mg modified-release tablets	Quetiapine 50mg modified-release tablets	Quetiapine fumarate
4957441000033113	Tolterodine 2mg/5ml oral suspension	Tolterodine 2mg/5ml oral suspension	Tolterodine tartrate
2643541000033110	Tolterodine 4mg modified-release capsules	Tolterodine 4mg modified-release capsules	Tolterodine tartrate
11072841000033114	Nortriptyline 10mg/5ml oral solution	Nortriptyline 10mg/5ml oral solution	Nortriptyline hydrochloride
12257841000033117	Nortriptyline 10mg/5ml oral suspension	Nortriptyline 10mg/5ml oral suspension	Nortriptyline hydrochloride
5975041000033116	Quetiapine 50mg/5ml oral solution	Quetiapine 50mg/5ml oral solution	Quetiapine fumarate

5975141000033117	Quetiapine 50mg/5ml oral suspension	Quetiapine 50mg/5ml oral suspension	Quetiapine fumarate
5078241000033113	Denzapine 50mg tablets	Denzapine 50mg tablets (Britannia Pharmaceuticals Ltd)	Clozapine
5078141000033118	Denzapine 200mg tablets	Denzapine 200mg tablets (Britannia Pharmaceuticals Ltd)	Clozapine
5077741000033118	Denzapine 50mg/ml oral suspension	Denzapine 50mg/ml oral suspension (Britannia Pharmaceuticals Ltd)	Clozapine
5077941000033115	Clozapine 200mg tablets	Clozapine 200mg tablets	Clozapine
5077641000033110	Clozapine 50mg/ml oral suspension sugar free	Clozapine 50mg/ml oral suspension sugar free	Clozapine
5271041000033118	Regurin XL 60mg capsules	Regurin XL 60mg capsules (Contura Ltd)	Trospium chloride
5314641000033110	Flotros 20mg tablets	Flotros 20mg tablets (Galen Ltd)	Trospium chloride
5575741000033110	Olanzapine embonate 210mg powder and solvent for suspension for injection vials	Olanzapine embonate 210mg powder and solvent for suspension for injection vials	Olanzapine embonate monohydrate
5565041000033117	Zypadhera 210mg powder and solvent for suspension for injection vials	Zypadhera 210mg powder and solvent for suspension for injection vials (Eli Lilly and Company Ltd)	Olanzapine embonate monohydrate
5575841000033117	Olanzapine embonate 300mg powder and solvent for suspension for injection vials	Olanzapine embonate 300mg powder and solvent for suspension for injection vials	Olanzapine embonate monohydrate
5565141000033118	Zypadhera 300mg powder and solvent for suspension for injection vials	Zypadhera 300mg powder and solvent for suspension for injection vials (Eli Lilly and Company Ltd)	Olanzapine embonate monohydrate
5575941000033113	Olanzapine embonate 405mg powder and solvent for suspension for injection vials	Olanzapine embonate 405mg powder and solvent for suspension for injection vials	Olanzapine embonate monohydrate
5565241000033113	Zypadhera 405mg powder and solvent for suspension for injection vials	Zypadhera 405mg powder and solvent for suspension for injection vials (Eli Lilly and Company Ltd)	Olanzapine embonate monohydrate
5909441000033118	Seroquel XL 150mg tablets	Seroquel XL 150mg tablets (Luye Pharma Ltd)	Quetiapine fumarate
5909341000033112	Quetiapine 150mg modified-release tablets	Quetiapine 150mg modified-release tablets	Quetiapine fumarate
6003041000033116	Solifenacin 10mg/5ml oral solution	Solifenacin 10mg/5ml oral solution	Solifenacin succinate
6003141000033117	Solifenacin 10mg/5ml oral suspension	Solifenacin 10mg/5ml oral suspension	Solifenacin succinate
6456941000033112	Zalasta 2.5mg tablets	Zalasta 2.5mg tablets (Consilient Health Ltd)	Olanzapine
6457141000033112	Zalasta 5mg tablets	Zalasta 5mg tablets (Consilient Health Ltd)	Olanzapine

6457241000033117	Zalasta 7.5mg tablets	Zalasta 7.5mg tablets (Consilient Health Ltd)	Olanzapine
6456741000033114	Zalasta 10mg tablets	Zalasta 10mg tablets (Consilient Health Ltd)	Olanzapine
6456841000033116	Zalasta 15mg tablets	Zalasta 15mg tablets (Consilient Health Ltd)	Olanzapine
6457041000033113	Zalasta 20mg tablets	Zalasta 20mg tablets (Consilient Health Ltd)	Olanzapine
6465641000033118	Zalasta 5mg orodispersible tablets	Zalasta 5mg orodispersible tablets (Consilient Health Ltd)	Olanzapine
6466041000033115	Olanzapine 10mg orodispersible tablets sugar free	Olanzapine 10mg orodispersible tablets sugar free	Olanzapine
6466141000033116	Olanzapine 15mg orodispersible tablets sugar free	Olanzapine 15mg orodispersible tablets sugar free	Olanzapine
6466241000033111	Olanzapine 20mg orodispersible tablets sugar free	Olanzapine 20mg orodispersible tablets sugar free	Olanzapine
6465941000033113	Olanzapine 5mg orodispersible tablets sugar free	Olanzapine 5mg orodispersible tablets sugar free	Olanzapine
6465341000033114	Zalasta 10mg orodispersible tablets	Zalasta 10mg orodispersible tablets (Consilient Health Ltd)	Olanzapine
6465441000033115	Zalasta 15mg orodispersible tablets	Zalasta 15mg orodispersible tablets (Consilient Health Ltd)	Olanzapine
6465541000033119	Zalasta 20mg orodispersible tablets	Zalasta 20mg orodispersible tablets (Consilient Health Ltd)	Olanzapine
7681641000033116	Olanzapine 10mg orodispersible tablets	Olanzapine 10mg orodispersible tablets	Olanzapine
7681741000033113	Olanzapine 15mg orodispersible tablets	Olanzapine 15mg orodispersible tablets	Olanzapine
7681841000033115	Olanzapine 20mg orodispersible tablets	Olanzapine 20mg orodispersible tablets	Olanzapine
7681941000033111	Olanzapine 5mg orodispersible tablets	Olanzapine 5mg orodispersible tablets	Olanzapine
6515041000033116	Arkolamyl 10mg orodispersible tablets	Arkolamyl 10mg orodispersible tablets (Mylan)	Olanzapine
6515141000033117	Arkolamyl 15mg orodispersible tablets	Arkolamyl 15mg orodispersible tablets (Mylan)	Olanzapine
6514941000033116	Arkolamyl 5mg orodispersible tablets	Arkolamyl 5mg orodispersible tablets (Mylan)	Olanzapine
6515241000033112	Arkolamyl 20mg orodispersible tablets	Arkolamyl 20mg orodispersible tablets (Mylan)	Olanzapine
2848741000033118	Pyridoxine 100mg/5ml oral suspension	Pyridoxine 100mg/5ml oral suspension	Pyridoxine hydrochloride
7687841000033114	Sondate XL 50mg tablets	Sondate XL 50mg tablets (Teva UK Ltd)	Quetiapine fumarate
7687941000033118	Sondate XL 200mg tablets	Sondate XL 200mg tablets (Teva UK Ltd)	Quetiapine fumarate
7688041000033115	Sondate XL 300mg tablets	Sondate XL 300mg tablets (Teva UK Ltd)	Quetiapine fumarate

7688141000033116	Sondate XL 400mg tablets	Sondate XL 400mg tablets (Teva UK Ltd)	Quetiapine fumarate
7861041000033118	Uraplex 20mg tablets	Uraplex 20mg tablets (Contura Ltd)	Trospium chloride
8112541000033115	Forceval Soluble tablets	Forceval Soluble tablets (Alliance Pharmaceuticals Ltd)	Alpha tocopheryl acetate/ Ascorbic acid/ Biotin/ Calcium/ Chromium/ Copper/ Cyanocobalamin/ Ergocalciferol/ Folic acid/ Iodine/ Iron/ Magnesium/ Manganese/ Molybdenum/ Nicotinamide/ Pantothenic acid/ Phosphorus/ Potassium/ Pyridoxine/ Riboflavin/ Selenium/ Thiamine/ Vitamin A/ Zinc
8114141000033119	Forceval Soluble Junior tablets	Forceval Soluble Junior tablets (Alliance Pharmaceuticals Ltd)	Alpha tocopheryl acetate/ Ascorbic acid/ Biotin/ Chromium/ Copper/ Cyanocobalamin/ Ergocalciferol/ Folic acid/ Iodine/ Iron/ Magnesium/ Manganese/ Molybdenum/ Nicotinamide/ Pantothenic acid/ Phytomenadione/ Potassium/ Pyridoxine/ Riboflavin/ Selenium/ Thiamine/ Vitamin A/ Zinc
8112841000033118	Santizor XL 4mg capsules	Santizor XL 4mg capsules (Upjohn UK Ltd)	Tolterodine tartrate
8112941000033114	Tenprolide XL 50mg tablets	Tenprolide XL 50mg tablets (Actavis UK Ltd)	Quetiapine fumarate
8113041000033116	Tenprolide XL 200mg tablets	Tenprolide XL 200mg tablets (Actavis UK Ltd)	Quetiapine fumarate
8113141000033117	Tenprolide XL 300mg tablets	Tenprolide XL 300mg tablets (Actavis UK Ltd)	Quetiapine fumarate
8128041000033117	Mariosea XL 4mg capsules	Mariosea XL 4mg capsules (Teva UK Ltd)	Tolterodine tartrate
8232241000033111	Neditol XL 4mg capsules	Neditol XL 4mg capsules (Aspire Pharma Ltd)	Tolterodine tartrate
8246241000033114	Sondate XL 150mg tablets	Sondate XL 150mg tablets (Teva UK Ltd)	Quetiapine fumarate
8263241000033114	Preblacon XL 4mg capsules	Preblacon XL 4mg capsules (Accord Healthcare Ltd)	Tolterodine tartrate
8274041000033113	Blerone XL 4mg capsules	Blerone XL 4mg capsules (Zentiva)	Tolterodine tartrate
8315941000033110	Neditol XL 2mg capsules	Neditol XL 2mg capsules (Aspire Pharma Ltd)	Tolterodine tartrate
8298341000033111	Seotiapim XL 200mg tablets	Seotiapim XL 200mg tablets (Sandoz Ltd)	Quetiapine fumarate
8298541000033116	Seotiapim XL 400mg tablets	Seotiapim XL 400mg tablets (Sandoz Ltd)	Quetiapine fumarate
8298441000033117	Seotiapim XL 300mg tablets	Seotiapim XL 300mg tablets (Sandoz Ltd)	Quetiapine fumarate

8300841000033111	Inconex XL 4mg capsules	Inconex XL 4mg capsules (Sandoz Ltd)	Tolterodine tartrate
8315741000033112	Tolterodine 2mg modified-release capsules	Tolterodine 2mg modified-release capsules	Tolterodine tartrate
8349141000033117	Tenprolide XL 400mg tablets	Tenprolide XL 400mg tablets (Actavis UK Ltd)	Quetiapine fumarate
11730041000033115	Quetiapine 200mg/5ml oral suspension	Quetiapine 200mg/5ml oral suspension	Quetiapine fumarate
850341000033118	Lomotil 2.5mg/25microgram tablets	Lomotil 2.5mg/25microgram tablets (Imported (Ireland))	Atropine sulfate/ Diphenoxylate hydrochloride
8960741000033111	Vesomni 6mg/0.4mg modified-release tablets	Vesomni 6mg/0.4mg modified-release tablets (Astellas Pharma Ltd)	Solifenacin succinate/ Tamsulosin hydrochloride
8960641000033119	Solifenacin 6mg / Tamsulosin 400microgram modified-release tablets	Solifenacin 6mg / Tamsulosin 400microgram modified-release tablets	Solifenacin succinate/ Tamsulosin hydrochloride
9604441000033112	Ebesque XL 50mg tablets	Ebesque XL 50mg tablets (Ethypharm UK Ltd)	Quetiapine fumarate
9605541000033112	Ebesque XL 200mg tablets	Ebesque XL 200mg tablets (Ethypharm UK Ltd)	Quetiapine fumarate
9605941000033118	Ebesque XL 300mg tablets	Ebesque XL 300mg tablets (Ethypharm UK Ltd)	Quetiapine fumarate
9606341000033113	Ebesque XL 400mg tablets	Ebesque XL 400mg tablets (Ethypharm UK Ltd)	Quetiapine fumarate
9703041000033111	Detrunorm XL 45mg capsules	Detrunorm XL 45mg capsules (Advanz Pharma)	Propiverine hydrochloride
9702941000033118	Propiverine 45mg modified-release capsules	Propiverine 45mg modified-release capsules	Propiverine hydrochloride
10615141000033115	Atrolak XL 50mg tablets	Atrolak XL 50mg tablets (Accord Healthcare Ltd)	Quetiapine fumarate
10615241000033110	Atrolak XL 200mg tablets	Atrolak XL 200mg tablets (Accord Healthcare Ltd)	Quetiapine fumarate
10615341000033117	Atrolak XL 300mg tablets	Atrolak XL 300mg tablets (Accord Healthcare Ltd)	Quetiapine fumarate
10615441000033111	Atrolak XL 400mg tablets	Atrolak XL 400mg tablets (Accord Healthcare Ltd)	Quetiapine fumarate
10041141000033118	Biquelle XL 50mg tablets	Biquelle XL 50mg tablets (Aspire Pharma Ltd)	Quetiapine fumarate
10041241000033113	Biquelle XL 150mg tablets	Biquelle XL 150mg tablets (Aspire Pharma Ltd)	Quetiapine fumarate
10041441000033114	Biquelle XL 300mg tablets	Biquelle XL 300mg tablets (Aspire Pharma Ltd)	Quetiapine fumarate
10041541000033110	Biquelle XL 400mg tablets	Biquelle XL 400mg tablets (Aspire Pharma Ltd)	Quetiapine fumarate
10041341000033115	Biquelle XL 200mg tablets	Biquelle XL 200mg tablets (Aspire Pharma Ltd)	Quetiapine fumarate

10041741000033119	Zaluron XL 50mg tablets	Zaluron XL 50mg tablets (Fontus Health Ltd)	Quetiapine fumarate
10041841000033112	Zaluron XL 150mg tablets	Zaluron XL 150mg tablets (Fontus Health Ltd)	Quetiapine fumarate
10041941000033116	Zaluron XL 200mg tablets	Zaluron XL 200mg tablets (Fontus Health Ltd)	Quetiapine fumarate
10042041000033110	Zaluron XL 300mg tablets	Zaluron XL 300mg tablets (Fontus Health Ltd)	Quetiapine fumarate
10042141000033114	Zaluron XL 400mg tablets	Zaluron XL 400mg tablets (Fontus Health Ltd)	Quetiapine fumarate
10254741000033110	Mintreleq XL 50mg tablets	Mintreleq XL 50mg tablets (Aristo Pharma Ltd)	Quetiapine fumarate
10254841000033117	Mintreleq XL 150mg tablets	Mintreleq XL 150mg tablets (Aristo Pharma Ltd)	Quetiapine fumarate
10254941000033113	Mintreleq XL 200mg tablets	Mintreleq XL 200mg tablets (Aristo Pharma Ltd)	Quetiapine fumarate
10255041000033113	Mintreleq XL 300mg tablets	Mintreleq XL 300mg tablets (Aristo Pharma Ltd)	Quetiapine fumarate
10255141000033112	Mintreleq XL 400mg tablets	Mintreleq XL 400mg tablets (Aristo Pharma Ltd)	Quetiapine fumarate
10332441000033118	Amitriptyline 10mg/5ml oral solution sugar free	Amitriptyline 10mg/5ml oral solution sugar free	Amitriptyline hydrochloride
10675541000033115	Mariosea XL 2mg capsules	Mariosea XL 2mg capsules (Teva UK Ltd)	Tolterodine tartrate
11578941000033118	Psyquet XL 50mg tablets	Psyquet XL 50mg tablets (Sandoz Ltd)	Quetiapine fumarate
11579241000033119	Psyquet XL 150mg tablets	Psyquet XL 150mg tablets (Sandoz Ltd)	Quetiapine fumarate
11579341000033112	Psyquet XL 200mg tablets	Psyquet XL 200mg tablets (Sandoz Ltd)	Quetiapine fumarate
11579441000033118	Psyquet XL 300mg tablets	Psyquet XL 300mg tablets (Sandoz Ltd)	Quetiapine fumarate
11579541000033117	Psyquet XL 400mg tablets	Psyquet XL 400mg tablets (Sandoz Ltd)	Quetiapine fumarate
11753641000033118	Quetiapine 20mg/ml oral suspension sugar free	Quetiapine 20mg/ml oral suspension sugar free	Quetiapine fumarate
11782141000033111	Brancico XL 300mg tablets	Brancico XL 300mg tablets (Zentiva)	Quetiapine fumarate
11782041000033112	Brancico XL 200mg tablets	Brancico XL 200mg tablets (Zentiva)	Quetiapine fumarate
11781941000033118	Brancico XL 150mg tablets	Brancico XL 150mg tablets (Zentiva)	Quetiapine fumarate
11782341000033114	Brancico XL 50mg tablets	Brancico XL 50mg tablets (Zentiva)	Quetiapine fumarate
11782241000033116	Brancico XL 400mg tablets	Brancico XL 400mg tablets (Zentiva)	Quetiapine fumarate
12292341000033116	Nortriptyline 50mg tablets	Nortriptyline 50mg tablets	Nortriptyline hydrochloride
1522841000033111	Vitamin B compound strong tablets	Vitamin B compound strong tablets	Nicotinamide/ Pyridoxine hydrochloride/ Riboflavin/ Thiamine hydrochloride

12428041000033114	Atrolak XL 150mg tablets	Atrolak XL 150mg tablets (Accord Healthcare Ltd)	Quetiapine fumarate
13133141000033113	Hydroxyzine 10mg/5ml oral suspension	Hydroxyzine 10mg/5ml oral suspension	Hydroxyzine hydrochloride
12605841000033119	Xonvea 10mg/10mg gastro-resistant tablets	Xonvea 10mg/10mg gastro-resistant tablets (Alliance Pharmaceuticals Ltd)	Doxylamine succinate/ Pyridoxine hydrochloride
12605741000033112	Doxylamine 10mg / Pyridoxine 10mg gastro-resistant tablets	Doxylamine 10mg / Pyridoxine 10mg gastro-resistant tablets	Doxylamine succinate/ Pyridoxine hydrochloride
12667541000033112	Vesicare 1mg/ml oral suspension	Vesicare 1mg/ml oral suspension (Astellas Pharma Ltd)	Solifenacin succinate
12667341000033117	Solifenacin 1mg/ml oral suspension sugar free	Solifenacin 1mg/ml oral suspension sugar free	Solifenacin succinate
45841000033119	Amitriptyline 25mg modified-release capsules	Amitriptyline 25mg modified-release capsules	Amitriptyline hydrochloride
55441000033119	Amitriptyline 25mg/5ml oral solution sugar free	Amitriptyline 25mg/5ml oral solution sugar free	Amitriptyline hydrochloride
45941000033110	Amitriptyline 50mg modified-release capsules	Amitriptyline 50mg modified-release capsules	Amitriptyline hydrochloride
55541000033118	Amitriptyline 50mg/5ml oral solution sugar free	Amitriptyline 50mg/5ml oral solution sugar free	Amitriptyline hydrochloride
2919841000033115	Atropine 1% eye drops 0.5ml unit dose preservative free	Atropine 1% eye drops 0.5ml unit dose preservative free	Atropine sulfate
2014541000033112	Atropine 1mg/10ml solution for injection pre-filled syringes	Atropine 1mg/10ml solution for injection pre-filled syringes	Atropine sulfate
89041000033118	Atropine 1mg/1ml solution for injection ampoules	Atropine 1mg/1ml solution for injection ampoules	Atropine sulfate
2014941000033118	Atropine 1mg/5ml solution for injection pre-filled syringes	Atropine 1mg/5ml solution for injection pre-filled syringes	Atropine sulfate
2015141000033119	Atropine 3mg/10ml solution for injection pre-filled syringes	Atropine 3mg/10ml solution for injection pre-filled syringes	Atropine sulfate
2014641000033113	Atropine 3mg/30ml solution for injection pre-filled syringes	Atropine 3mg/30ml solution for injection pre-filled syringes	Atropine sulfate
89141000033119	Atropine 400micrograms/1ml solution for injection ampoules	Atropine 400micrograms/1ml solution for injection ampoules	Atropine sulfate

2014341000033117	Atropine 500micrograms/5ml solution for injection pre-filled syringes	Atropine 500micrograms/5ml solution for injection pre-filled syringes	Atropine sulfate
89241000033114	Atropine 600micrograms/1ml solution for injection ampoules	Atropine 600micrograms/1ml solution for injection ampoules	Atropine sulfate
157441000033118	Brompheniramine 2mg/5ml oral solution	Brompheniramine 2mg/5ml oral solution	Brompheniramine maleate
2929541000033114	Promethazine 25mg/1ml solution for injection ampoules	Promethazine 25mg/1ml solution for injection ampoules	Promethazine hydrochloride
741541000033119	Hydroxyzine 10mg/5ml oral solution	Hydroxyzine 10mg/5ml oral solution	Hydroxyzine hydrochloride
4020341000033111	Diphenhydramine 10mg/5ml oral solution	Diphenhydramine 10mg/5ml oral solution	Diphenhydramine hydrochloride
3085841000033117	Chlorphenamine 10mg/1ml solution for injection ampoules	Chlorphenamine 10mg/1ml solution for injection ampoules	Chlorphenamine maleate
3063141000033110	Trihexyphenidyl 5mg/5ml oral solution	Trihexyphenidyl 5mg/5ml oral solution	Trihexyphenidyl hydrochloride
1662441000033114	Paroxetine 10mg/5ml oral suspension sugar free	Paroxetine 10mg/5ml oral suspension sugar free	Paroxetine hydrochloride
12988741000033118	Olanzapine 2.5mg orodispersible tablets sugar free	Olanzapine 2.5mg orodispersible tablets sugar free	Olanzapine
12988841000033111	Olanzapine 7.5mg orodispersible tablets sugar free	Olanzapine 7.5mg orodispersible tablets sugar free	Olanzapine
3085941000033113	Chlorphenamine 2mg/5ml oral solution	Chlorphenamine 2mg/5ml oral solution	Chlorphenamine maleate
3086041000033115	Chlorphenamine 2mg/5ml oral solution sugar free	Chlorphenamine 2mg/5ml oral solution sugar free	Chlorphenamine maleate
13158941000033115	Zaponex 12.5mg orodispersible tablets	Zaponex 12.5mg orodispersible tablets (Leyden Delta B.V.)	Clozapine
13159141000033111	Zaponex 25mg orodispersible tablets	Zaponex 25mg orodispersible tablets (Leyden Delta B.V.)	Clozapine
13159241000033116	Zaponex 50mg orodispersible tablets	Zaponex 50mg orodispersible tablets (Leyden Delta B.V.)	Clozapine
13158841000033111	Zaponex 100mg orodispersible tablets	Zaponex 100mg orodispersible tablets (Leyden Delta B.V.)	Clozapine
13159041000033112	Zaponex 200mg orodispersible tablets	Zaponex 200mg orodispersible tablets (Leyden Delta B.V.)	Clozapine
13158341000033119	Clozapine 100mg orodispersible tablets sugar free	Clozapine 100mg orodispersible tablets sugar free	Clozapine

13158441000033113	Clozapine 12.5mg orodispersible tablets sugar free	Clozapine 12.5mg orodispersible tablets sugar free	Clozapine
13158541000033114	Clozapine 200mg orodispersible tablets sugar free	Clozapine 200mg orodispersible tablets sugar free	Clozapine
13158641000033110	Clozapine 25mg orodispersible tablets sugar free	Clozapine 25mg orodispersible tablets sugar free	Clozapine
13158741000033118	Clozapine 50mg orodispersible tablets sugar free	Clozapine 50mg orodispersible tablets sugar free	Clozapine
13300841000033111	Nortriptyline 10mg/5ml oral solution sugar free	Nortriptyline 10mg/5ml oral solution sugar free	Nortriptyline hydrochloride
13300941000033115	Nortriptyline 25mg/5ml oral solution sugar free	Nortriptyline 25mg/5ml oral solution sugar free	Nortriptyline hydrochloride
13353141000033117	Tolthen XL 2mg capsules	Tolthen XL 2mg capsules (Northumbria Pharma Ltd)	Tolterodine tartrate
13353341000033119	Tolthen XL 4mg capsules	Tolthen XL 4mg capsules (Northumbria Pharma Ltd)	Tolterodine tartrate
13421541000033118	Tolterma XL 4mg capsules	Tolterma XL 4mg capsules (Macleods Pharma UK Ltd)	Tolterodine tartrate
13421441000033119	Tolterma XL 2mg capsules	Tolterma XL 2mg capsules (Macleods Pharma UK Ltd)	Tolterodine tartrate
979241000033110	Nortriptyline 10mg capsules	Nortriptyline 10mg capsules	Nortriptyline hydrochloride
979341000033117	Nortriptyline 25mg capsules	Nortriptyline 25mg capsules	Nortriptyline hydrochloride
1661841000033115	Orphenadrine 50mg/5ml oral solution sugar free	Orphenadrine 50mg/5ml oral solution sugar free	Orphenadrine hydrochloride
1114141000033111	Promethazine 5mg/5ml oral solution sugar free	Promethazine 5mg/5ml oral solution sugar free	Promethazine hydrochloride
4518641000033111	Fesoterodine 8mg modified-release tablets	Fesoterodine 8mg modified-release tablets	Fesoterodine fumarate
4518541000033110	Fesoterodine 4mg modified-release tablets	Fesoterodine 4mg modified-release tablets	Fesoterodine fumarate
3993241000033111	Darifenacin 15mg modified-release tablets	Darifenacin 15mg modified-release tablets	Darifenacin hydrobromide
3993141000033116	Darifenacin 7.5mg modified-release tablets	Darifenacin 7.5mg modified-release tablets	Darifenacin hydrobromide
13734941000033119	Biquelle XL 600mg tablets	Biquelle XL 600mg tablets (Aspire Pharma Ltd)	Quetiapine fumarate
13734841000033110	Quetiapine 600mg modified-release tablets	Quetiapine 600mg modified-release tablets	Quetiapine fumarate

Appendix: codelists used in the study

List created by first author 20/12/2023 – using pcd search command, for each of ACB_3 score drugs listed here: file (westessexccg.nhs.uk)

1. Davidson JaW-G, C. . Clinical codelist - Dementia [Internet]. *London School of Hygiene & Tropical Medicine*. 2021.
2. Kontopantelis E. PCDSEARCH: Stata module to extract code lists from primary care databases Statistical Software Component. 2015.
3. Mok PLH, Carr MJ, Guthrie B, Morales DR, Sheikh A, Elliott RA, et al. Multiple adverse outcomes associated with antipsychotic use in people with dementia: population based matched cohort study. *BMJ*. 2024;385:e076268.
4. Anglia UoE. Drugs on the ACB Scale 2012 [Available from: https://www.uea.ac.uk/documents/746480/2855738/Anticholinergics.pdf.
